# Large-scale data analysis for robotic yeast one-hybrid platforms and multi-disciplinary studies using GateMultiplex

**DOI:** 10.1186/s12915-021-01140-y

**Published:** 2021-09-24

**Authors:** Ni-Chiao Tsai, Tzu-Shu Hsu, Shang-Che Kuo, Chung-Ting Kao, Tzu-Huan Hung, Da-Gin Lin, Chung-Shu Yeh, Chia-Chen Chu, Jeng-Shane Lin, Hsin-Hung Lin, Chia-Ying Ko, Tien-Hsien Chang, Jung-Chen Su, Ying-Chung Jimmy Lin

**Affiliations:** 1 Department of Life Science and Institute of Plant Biology, College of Life Science, National Taiwan University, Taipei, 10617 Taiwan; 2 Department of Pharmacy, National Yang Ming Chiao Tung University, Taipei, 11221 Taiwan; 3 Genome and Systems Biology Degree Program, National Taiwan University and Academia Sinica, Taipei, 10617 Taiwan; 4 Biotechnology Division, Taiwan Agricultural Research Institute, Taichung, 41362 Taiwan; 5 Genomics Research Center, Academia Sinica, Taipei, 11529 Taiwan; 6 Department of Life Sciences, National Chung Hsing University, Taichung, 40227 Taiwan; 7 Department of Horticulture and Biotechnology, Chinese Culture University, Taipei, 11114 Taiwan; 8 Department of Life Sciences and Institute of Fisheries Science, National Taiwan University, Taipei, 10617 Taiwan

**Keywords:** Yeast one-hybrid, C++, Preclinical drug discovery, Precision agriculture, Deep-sea fishery

## Abstract

**Background:**

Yeast one-hybrid (Y1H) is a common technique for identifying DNA-protein interactions, and robotic platforms have been developed for high-throughput analyses to unravel the gene regulatory networks in many organisms. Use of these high-throughput techniques has led to the generation of increasingly large datasets, and several software packages have been developed to analyze such data. We previously established the currently most efficient Y1H system, meiosis-directed Y1H; however, the available software tools were not designed for processing the additional parameters suggested by meiosis-directed Y1H to avoid false positives and required programming skills for operation.

**Results:**

We developed a new tool named GateMultiplex with high computing performance using C++. GateMultiplex incorporated a graphical user interface (GUI), which allows the operation without any programming skills. Flexible parameter options were designed for multiple experimental purposes to enable the application of GateMultiplex even beyond Y1H platforms. We further demonstrated the data analysis from other three fields using GateMultiplex, the identification of lead compounds in preclinical cancer drug discovery, the crop line selection in precision agriculture, and the ocean pollution detection from deep-sea fishery.

**Conclusions:**

The user-friendly GUI, fast C++ computing speed, flexible parameter setting, and applicability of GateMultiplex facilitate the feasibility of large-scale data analysis in life science fields.

**Supplementary Information:**

The online version contains supplementary material available at 10.1186/s12915-021-01140-y.

## Background

The physical interactions between transcription factors (TFs) and their cis-DNA elements fine-tune the differential expression of the target genes [1–5]. Such transcriptional regulations carry out the prokaryotic and eukaryotic organismal development and response to the environmental cues [6]. Yeast one-hybrid (Y1H) has been used extensively to identify the interactions between TFs with DNA fragments [2, 7–12]. Integration of numerous TF-DNA interactions forms complex gene regulatory networks (GRN), which provides comprehensive insights to interpret phenotypes involved in different biological processes [2, 7–12]. To efficiently discover large-scale TF-DNA interactions for GRN constructions, Y1H has been evolved into high-throughput platforms operated through robotic arraying [2, 7–10, 12] or liquid-handling [11] machineries. In animal and plant kingdoms, many GRNs have been established to explore human disease [9], fly eye development [11], worm digestive tract [13], herbaceous root development [12], crop phenolic biosynthesis [14], and tree wood formation [2].

High-throughput Y1Hs are performed in high-density-formatted (HDF) plates. HDF plates allow the robotic machines to perform the experiments in a high-throughput manner. The formats of HDF plates are formed through a geometric sequence with [3 × 2^(*n* + 1)^] as the length and [2 × 2^(*n* + 1)^] as the width (Additional file 1: Fig. S1a). When 0 is substituted into the *n* of [3 × 2^(*n* + 1)^] × [2 × 2^(*n* + 1)^], then the format results as [3 × 2] × [2 × 2], which is a 24-format (6 × 4). When 1 to 4 are substituted individually into the *n*, then the formats become 96 (Additional file 1: Fig. S1b), 384 (Additional file 1: Fig. S1c), 1536 (Additional file 1: Fig. S1d), and 6144, respectively. The robotic platforms usually output text or CSV files containing the sample names, treatment categories, and quantified colony size/signal [2, 15–19]. The following analysis usually starts with the identification of signal information from the output files. The signal is then categorized into different groups using their sample names, such as reference (negative control) and TF (experimental groups), with different treatments, e.g., reporters and incubation times. A positive TF-DNA interaction is identified through the application of the signals from the experimental groups with several cutoff settings [2, 7–12]. *Background noise cutoff* was used to eliminate the signal from background noise caused by reporter self-activation. *Reference cutoff* was applied to compare the signal between the reference (negative control) and the experimental groups, and a fold-change was usually set to adjust the selection stringency. For example, the fold-change can be set as 2. If the signals from an experimental group are two times higher than its reference, then this experimental group can then be regarded as a positive colony. Each reference and experimental groups are usually represented by multiple biological replicates and technical replicates (colonies) to increase the robustness and reproducibility. *Biological/technical replicate cutoff* is usually set to determine a positive TF-DNA interaction event. A TF-DNA combination, for example, can be tested using 16 replicates. If biological/technical replicate cutoff is set as 8, then it means that the positive colonies need to be more than 8 out of 16 to evaluate such TF-DNA interaction as a positive event. Once the signals pass all cutoffs, then such TF and DNA would be regarded to interact with each other using yeast one-hybrid screening.

High-throughput platforms coupled with HDF plates usually generate hundreds of thousands sets of text-based signal [2, 15–19]. Many tools have been developed to analyze the subsequent multiple-step cutoff analysis. However, most of these tools were developed for specific projects, such as SpotOn [10] and TIDY [11] for yeast one-hybrid (Y1H), BASE for microarray [20], CellMissy for single-cell migration [21], DMAN for scanning fluorimetry [22], and DRfit for preclinical lead compound identification [23]. Most of the high-throughput analyzing tools were established in scripting languages, which are not able to provide efficient and timely analysis for the large-scale datasets. Tools developed in compiling languages exhibited much faster speed and lower memory and hardware requirement than scripting languages [24–27]. C++, as a compiling language, has been shown with the best performance among the programming languages commonly used in bioinformatics field [24–27]. However, most of the current tools were not written in C++, instead, in Java or scripting languages.

In this study, we developed GateMultiplex using C++ to analyze the data generated widely from high-throughput platforms using HDF plates or even more complexed format plates with high flexibility for cutoff settings. GateMultiplex is composed of three parts: GM_Converter, GM_Basic, and GM_Advanced. GM_Converter can transform different data formats into a fixed format for GM_Basic or GM_Advanced to perform analysis. GM_Basic or GM_Advanced can process text-mining from the text-based data sets and allow the users to select flexible cutoff settings, in which GM_Advanced provides more advanced options of the cutoff settings and output file categories. We incorporated an easy-to-use graphical user interface (GUI) for GateMultiplex to allow the users to customize their needs while designing their experiments and analyzing the data, and this GUI allows the users to operate without the requirement of programming skills. In addition, GateMultiplex can also be applied to analyze large-scale data from numerous fields in life science beyond the limitation of HDF plates. Four high-throughput data sets from different fields were used to demonstrate the analyzing flexibility of GateMultiplex. (1) Y1H screening for the identification of TF-DNA interactions; (2) lead identification in preclinical cancer drug discovery; (3) precision agriculture for crop line selection and harvesting time decision; (4) detecting ocean pollution generated from deep-sea fishery. In summary, fast speed, flexibility, and applicability of GateMultiplex increase the research project feasibility with large-scale data in life science fields.

## Implementation

### GateMultiplex: a C++ package to analyze large-scale HDF plates generated from Y1H

**Fig. 1:**
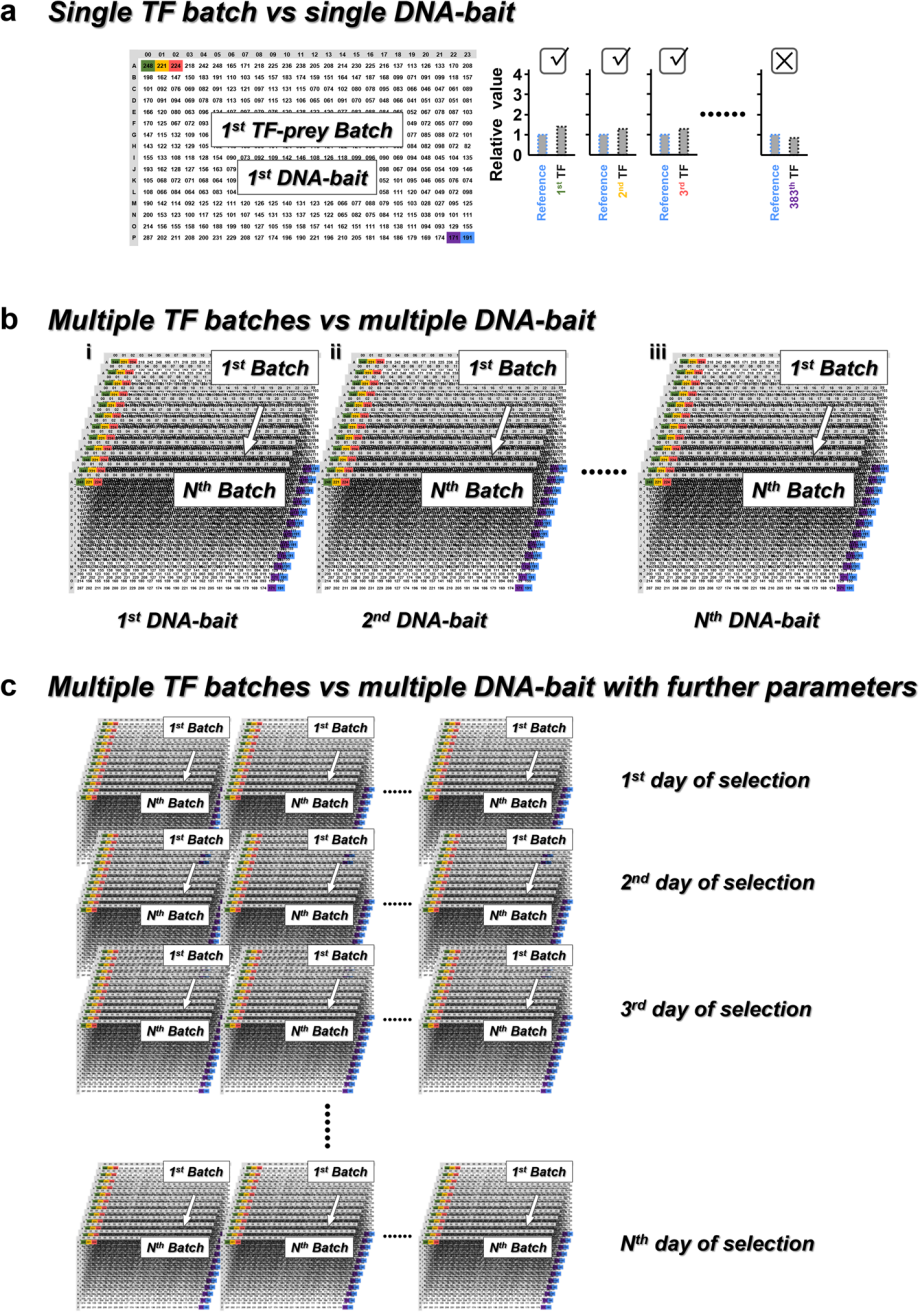
Large-scale analysis. **a** Take a 384-format plate with quantified yeast colony sizes for example, this plate could be a result from the screening of a 383 TF-prey batch (from green, yellow, red, to purple) with a negative control/reference (blue) against a DNA-bait. The numbers on the plate represent the size values of each colony for the TF-prey batch against the DNA-bait. The value of each TF-DNA combination would be compared to the reference value. If the value of a TF-DNA combination is higher than the reference value, the TF-prey would be regarded as a positive, such as the 1st TF. On the opposite, when the value of a TF-DNA combination is lower than the reference value, the TF-prey would be interpreted as a negative, such as the 383th TF. The TF-DNA combinations can be increased into **b** multiple TF-prey batches against multiple DNA-baits (from i to iii). **c** Multiple TF-prey batches against multiple DNA-baits can be integrated with different parameters, such as different culturing days to generate dramatically increased numbers of colony size values

We first used Y1H screening platforms to demonstrate the analyzing concept and the essential elements of GateMultiplex. In general, Y1H requires the incorporation of TF-preys and DNA-baits into yeast cells for the selection of TF-DNA interactions. TF-preys are first transformed into yeast cells to form a TF-prey library. The DNA-baits are then integrated with the TF-prey library by mating or transformation. Since high-throughput Y1H screenings usually involve the TF-prey library with hundreds or thousands of TFs, the TFs are arranged into different batches in HDF plates. Take our recently developed meiosis-directed Y1H platform as an example, different TF batches were arranged in 96-well liquid plates and transferred four times to solid agar plates into 384-format (96 × 4 technical replicates = 384) using the robotic arms with 96 high-density long pins (Additional file 1: Fig. S2a). Such 384 yeast colonies were then transferred among different plates using 384 high-density short pins (Additional file 1: Fig. S2b and S3) for the following meiosis and selection steps. The final colony sizes on the selection plates were quantified (Additional file 1: Fig. S4). An empty vector without TF is usually used as a reference representing the negative control (Fig. 1a, blue sample). Take one TF-prey batch in one 384-format HDF plate for example, the result signal from the combination of the first-batch TF-preys with the first DNA-bait (Fig. 1a, the green, orange, pink, …, and purple samples) would be compared to that from the reference (Fig. 1a, the blue sample). If the signal from a TF-DNA combination is higher than the reference, then such TF-prey colony can be interpreted as a positive (Fig. 1a, the green, orange and pink samples). The data amount generated from the screenings for a single (Fig. 1a) or multiple (Fig. 1b i) TF-prey batches against a single DNA-bait can be easily analyzed by Excel. When the screening scale increases, Excel can no longer process, for example, the result signal from multiple TF-prey batches against multiple DNA-baits (Fig. 1b ii, iii). Many programming scripting languages, such as R or Python, can be used for analyzing the enlarged data scale, which only requires easier script writing skills. The data scale would be even exponentially increased with more parameters incorporated, such as incubation days during selection (Fig. 1c). Under such scale, the computing speed of scripting language would be obviously slower than compiled language, such as C++. We therefore developed a C++-based software for Windows operating system, GateMultiplex, to analyze the large-scale data generated from Y1H screening.

### Identifying information from the input files

**Fig. 2:**
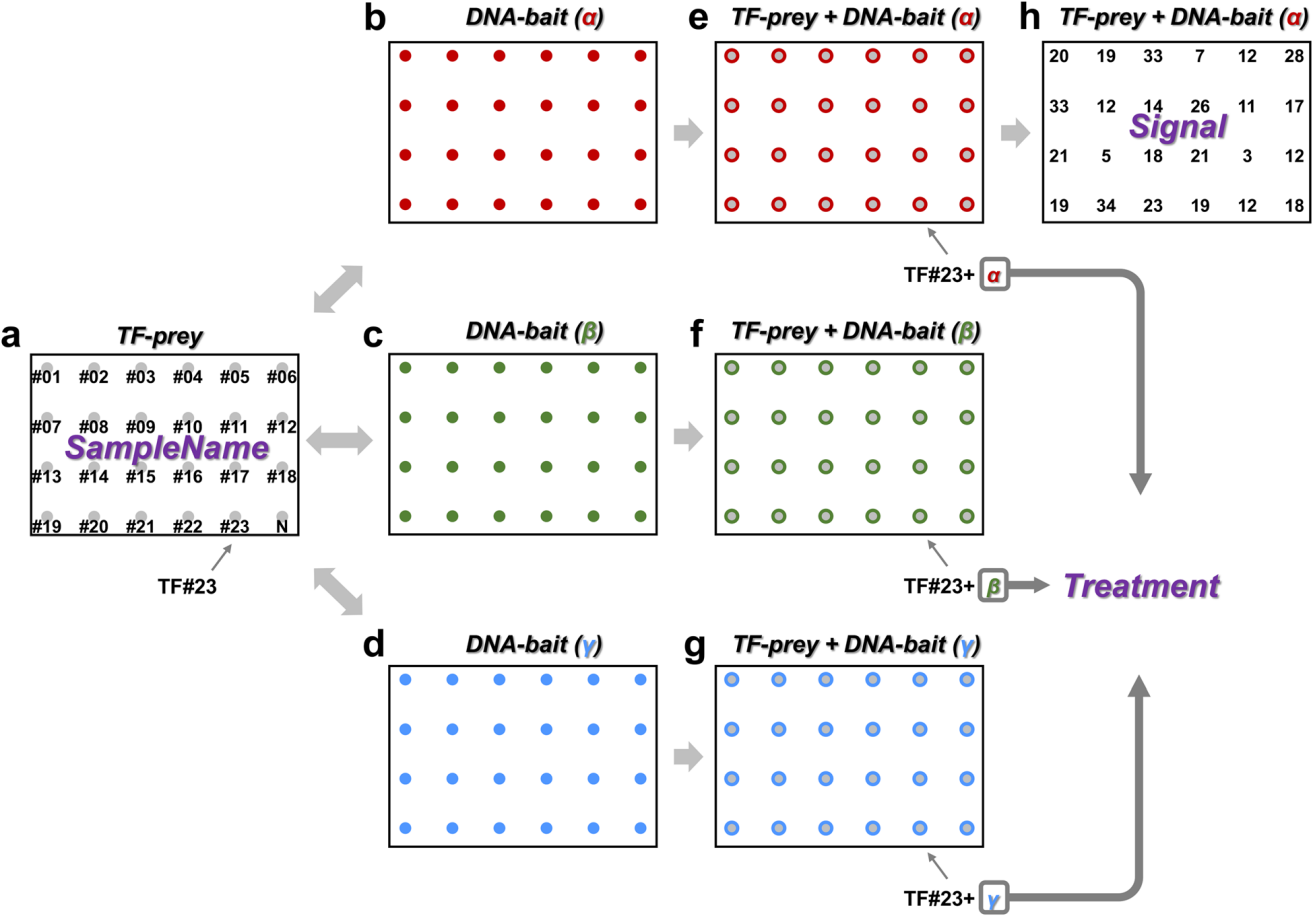
SampleName, Treatment and Signal. **a** An Y1H screening example of a simple TF-prey batch containing 23 TF-preys (named from TF#01 to TF#23) and 1 negative control (shown as *N* in this figure). The TF name and the *N* are defined as “SampleName”. **b**–**g** The TF-prey batch would be mated with **b** DNA-bait(α), **c** DNA-bait(β), and **d** DNA-bait(γ), which would respectively result in the yeast cells with both **e** TF-prey and DNA-bait(α), **f** TF-prey and DNA-bait(β), and (**g**) TF-prey and DNA-bait(γ). DNA-bait is defined as “Treatment,” and different DNA-baits represent different “Treatment” types. **h** The yeast cells would grow into colonies and be further quantified into colony size values as “Signal”

**Fig. 3:**
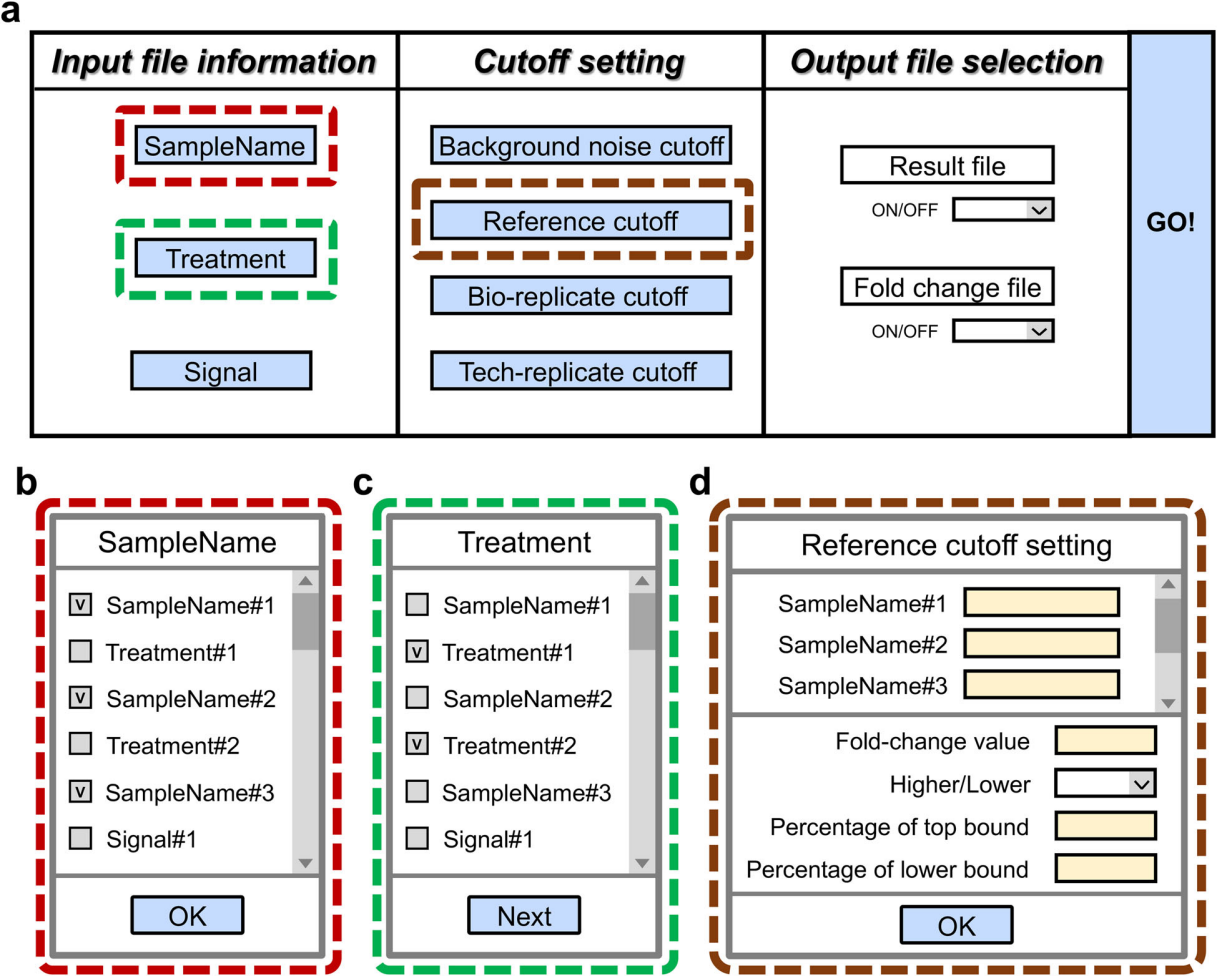
Graphical user interface (GUI). **a** The diagram of the GUI of GM_Basic (see Additional file 2 for the exact appearance of GUIs). The GUI contains three main areas, including the input file information, the cutoff setting and the output file selection. The input file information area is used to identify “SampleName,” “Treatment,” and “Signal” from the input files. The cutoff setting area includes background noise cutoff, reference cutoff, bio-replicate cutoff, and tech-replicate cutoff. The output file selection area offers two kinds of output files, the result file, and the fold-change file. The input file information area and the cutoff setting area were designed with clicking buttons (colored in blue). The output file selection area was designed with drop-down lists to provide on or off options. When the buttons are clicked, **b–d** the corresponding windows would pop up. Three pop-up windows are shown here as examples, including (**b**) the “SampleName” window, (**c**) the “Treatment” window, and (**d**) the reference cutoff setting window. The required information can be selected in (**b**) the “SampleName” window and (**c**) the “Treatment” window. **d** In reference cutoff setting window, dialog boxes (colored in yellow) are used for entering required numbers or words. After inputting all required information into GUI, pressing the “GO!” button (**a**) would start the data analyzing process

In Y1H, a TF-prey batch (Fig. 2a) would be mated with many DNA-baits (α, β, and γ in Fig. 2b–d), resulting in the yeast cells containing both TF-preys and DNA-baits (Fig. 2e–g). The names of each TF-prey and negative control, TF#01 to TF#23 then to N (Fig. 2a), are the sample names. The DNA-bait names, α, β, and γ (Fig. 2e–g), represent different treatment categories, because the same TF-prey can be mated with different DNA-baits as different treatments (Fig. 2e–g, TF#23 + α, TF#23 + β, TF#23 + γ). The colony sizes would then be quantified into different values as the signal (Fig. 2 h and Additional file 1: Fig. S4). In the Input file information part of the GUI (Fig. 3), the users can identify and categorize the information from their input CSV files into the sample names, treatments, and signal (Additional file 1: Fig. S5. See details in the Additional file 2). Among the sample names, the user can select a sample name as the reference/negative control (blue color in Additional file 1: Fig. S5).

### Flexible and different types of cutoff settings

**Fig. 4:**
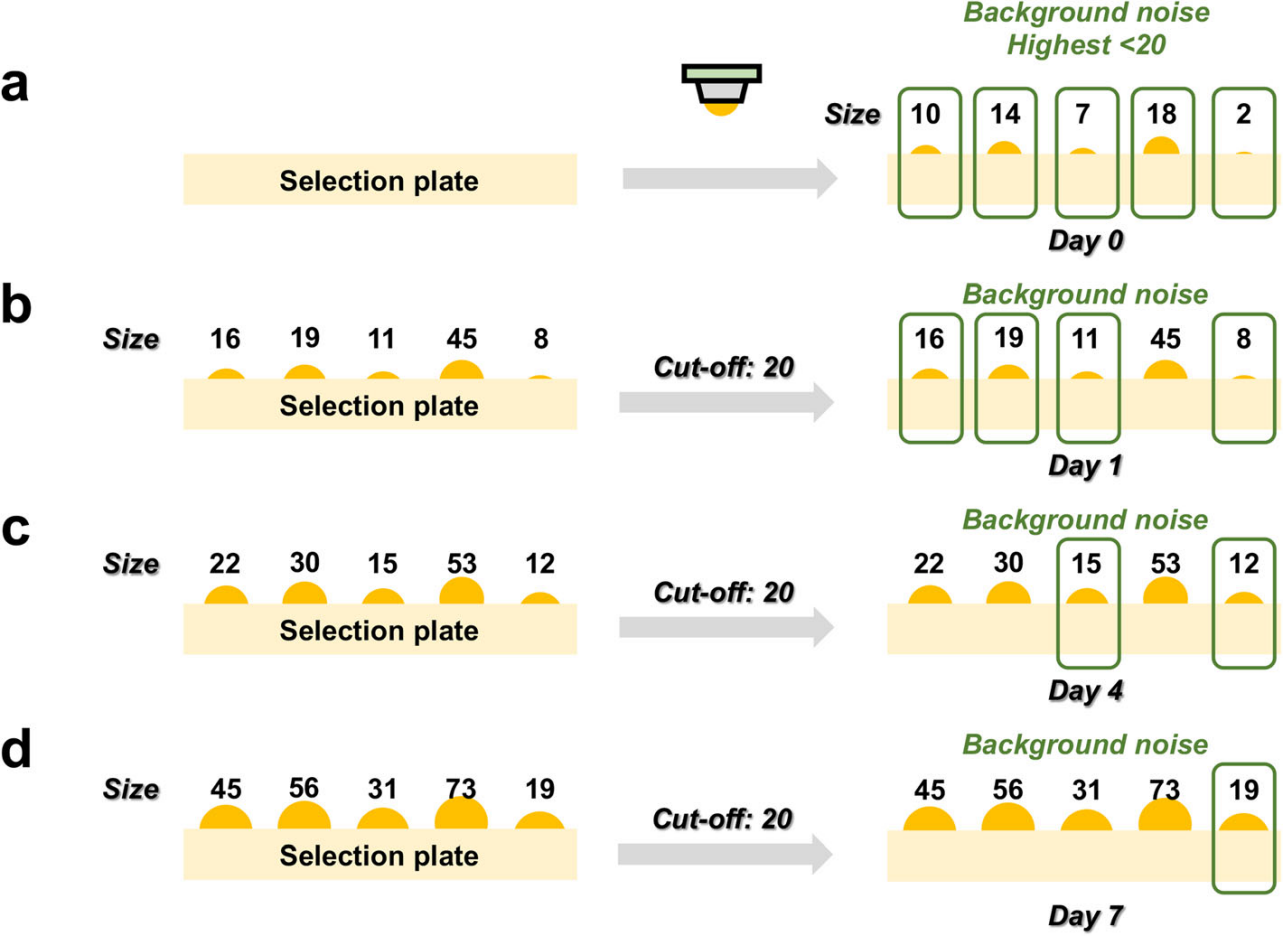
Background noise cutoff. **a** The colonies were transferred onto the selection plate and further quantified on different culturing day, including day 0, **b** day 1, **c** day 4, and **d** day 7. During (**a**) the pinning process, the transferred cell amount of each yeast colony would not be exactly the same. The numbers above the colonies represent the colony size. In this case, all colony sizes are smaller than 20, and thus 20 is used as the cutoff for background noise. If a colony is not larger than cutoff value 20, the signal from this colony would be regarded as the signal from background noise. For example, the colony signal with size 7 on **a** day 0 would be interpreted as the background noise as well as on **b** day 1 (size 11), and **c** day 4 (size 15). **d** On day 7, the colony signal increased to 31, larger than the cutoff value 20 and would no longer be regarded as the background noise

In the cutoff setting part (Fig. 3a), *we designed three types of cutoff*, and each cutoff type can be enabled or disabled to meet the requirements of various experimental workflows. *First is the background noise cutoff*: In Y1H, the background noise represents the yeast cell amounts transferred to the selection plates (Fig. 4a), namely, the signal of the starting yeast cells. Since the pins of the robotic platform cannot transfer the exact same cell amounts, the cell numbers may vary on the selection plates (Fig. 4a). In one possible scenario, the starting TF yeast cells are higher than the reference cells due to the uneven cell transferring. If none of the cells grow after several days, the TFs would then be counted as positive events because of their higher starting cell numbers. Thus, the setting of background noise cutoff prevents the bias caused by such uneven yeast cell transferring. Background noise cutoff can be set as a value, which is higher than the largest starting yeast size on day 0 (e.g., the value 20 in Fig. 4a). The yeast colonies on the selection plates would then grow along with the incubation days, and their size increased as well. The signals would be further computed only when they passed background noise cutoff. For example, the fourth colony (Fig. 4a) showed a result signal as 18 on day 0 (Fig. 4a), and increased to 45 on day 1 (Fig. 4b). Since 45 is higher than the cutoff as 20, the signal of this colony on day 1 would be processed. Instead, even the signal of the third colony raised from 7 to 15 on day 4 (Fig. 4a–c), the signal of this colony would still be eliminated as background noise. Once the third colony signal becomes higher than the cutoff on day 7 as 31 (Fig. 4d), its value would be collected for the analysis.

**Fig. 5:**
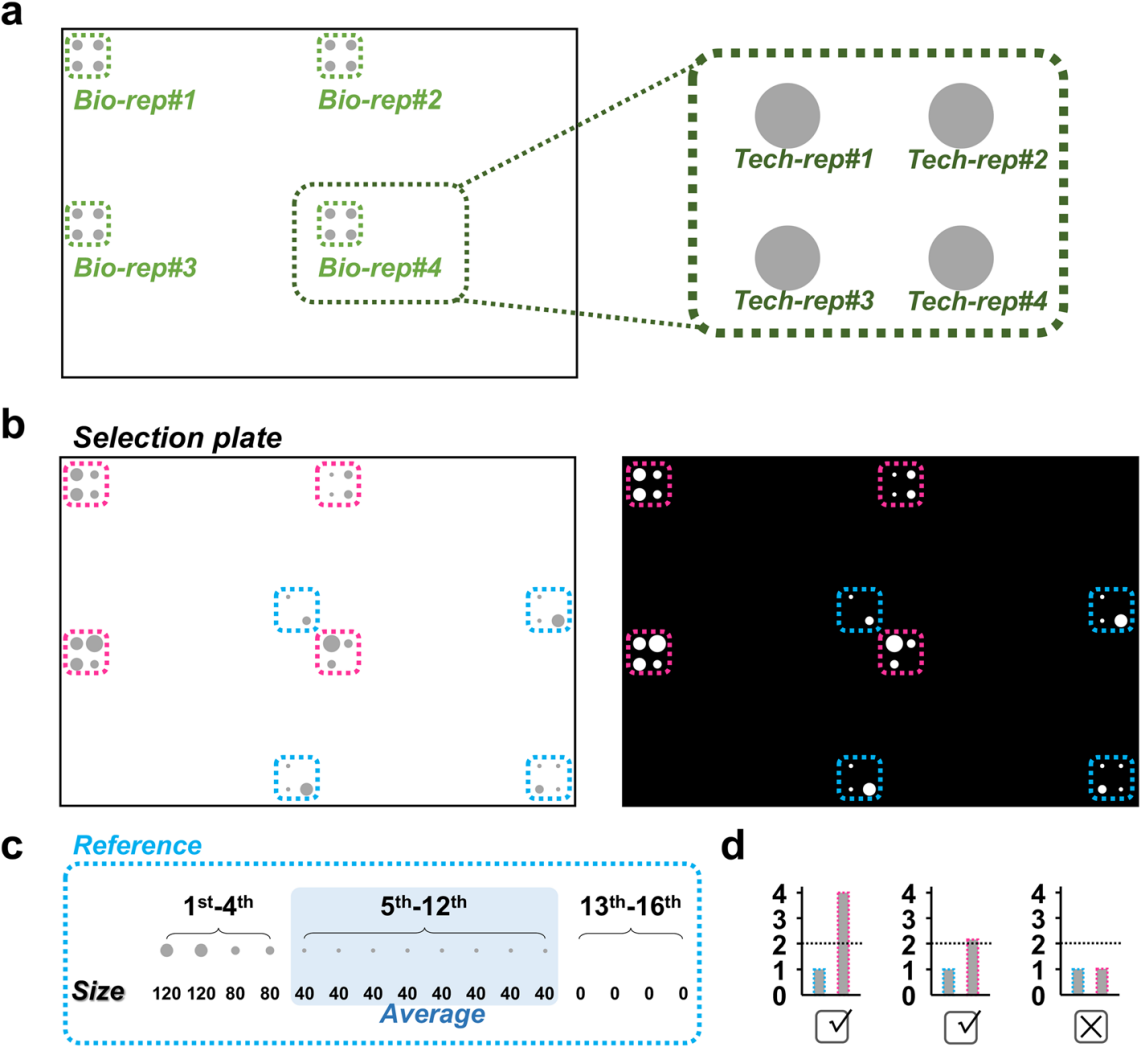
Reference cutoff. **a** One TF-DNA combination would be composed of 4 biological replicates (from bio-rep#1 to bio-rep#4). Each biological replicate is composed of 4 technical replicate colonies (from tech-rep#1 to tech-rep#4), resulting in 16 replicate colonies. **b** The 16 colonies in pink dashed frames belong to an experimental sample, and the other 16 colonies in blue dashed frames represent the reference sample. Through the black-and-white image, the size of each colony can be quantified. **c** The 16 colonies of reference were ranked based on the sizes. The size range from the 5th to the 12th (in blue background) were averaged. **d** The averaged value was defined as 1, and the fold-change value was set as 2 folds of the averaged value. As a result, reference cutoff includes the averaging ranges of the reference and the fold-change values. If the relative size of one experimental colony is larger than the cutoff value 2, then the colony would be regarded as a positive. If not, then the colony would be regarded as a negative.

*Second is the reference cutoff*: We also examine whether the signals of each TF-prey-DNA-bait colony signals are significantly higher than that of the reference colonies (empty vector-DNA-bait) to define the positive TF-prey-DNA-bait colonies. Here we take our currently established meiosis-directed Y1H platform as an example. In each TF-prey (or empty vector) and DNA-bait combination, we performed four biological replicates each with four technical replicates into total 16 replicates (Fig. 5a). GM_Basic provides two adjustable parameters to fine-tune the signals from the reference for the users to determine the selecting stringency. Since 16 reference colonies may result in different sizes (Fig. 5b, the gray dots in the blue dashed circles), their signals would be ranked from high to low (Fig. 5c). One parameter allows the users to choose the range to average the signals (Fig. 5c), and we then used the average signals as a cutoff. The other parameter can set a further fold-change of this cutoff to increase or decrease the stringency. Such cutoff with the fold-change setting is called as reference cutoff, and the users can select the signals higher or lower reference cutoff as positives (Additional file 1: Fig. S6). Take our previous study for an example, we compared the signals from each TF-prey-DNA-bait (Fig. 5b, the gray dots in the dashed pink circles) and reference colonies (Fig. 5b). To set reference cutoff, we ranked the signals of the reference colonies, and averaged the values from the 5th to 12th (Fig. 5c). The signals of each TF-prey-DNA-bait colony (Fig. 5d, the gray bars in pink dashed lines) were then compared to the averaged values of the reference (Fig. 5d, the gray bars in blue dashed lines). We further chose the fold-change as 2, and the TF-prey-DNA-bait colonies would be counted as positive colonies as their signals higher than 2-fold of the reference (Fig. 5d).

**Fig. 6:**
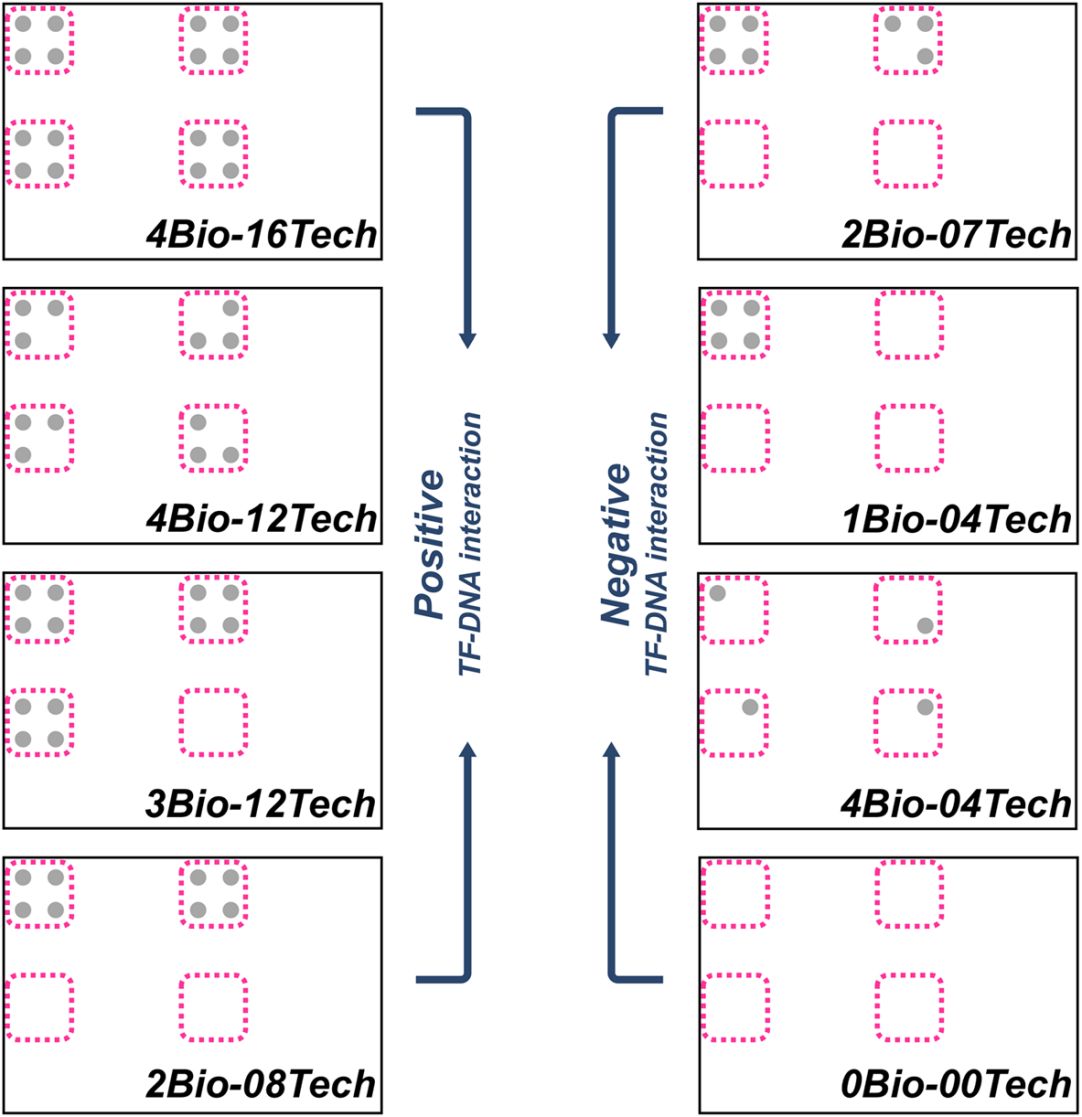
Biological and technical replicate cutoff. A TF-DNA combination contains 16 colonies as their biological and technical replicates. The 16 replicates would be processed into positives or negatives. Positive and negative replicates are shown as gray dots and empties, respectively. If biological and technical replicate cutoff is set as 8 replicates. The TF-DNA combination with more than or equal to 8 positive replicates would be identified as a positive TF-DNA interaction event. For examples, 4Bio-16Tech (as 16 positive technical replicates from 4 biological replicates), 4Bio-12Tech, 3Bio-12Tech, and 2Bio-08Tech represent the positive TF-DNA interaction events. Instead, the TF-DNA combination with less than 8 positive replicates would be regarded as a negative interaction event, such as 2Bio-07Tech, 1Bio-04Tech, 4Bio-04Tech, and 0Bio-00Tech

*Third is the biological and technical replicate cutoff*: GM_Basic then sums the number of the positive colonies from each TF-Prey-DNA-bait combination. If all 16 colonies were positive, then such TF-Prey-DNA-bait combination would be regarded as 4Bio-16Tech (Fig. 6). If only four technical replicates in one biological replicate pass, this TF-DNA combination would be counted as 1Bio-04Tech (Fig. 6). If we set bio-/tech-replicate cutoff as 2Bio and 08Tech, then only the TF-DNA combinations with more than 2 biological and 8 technical replicates can pass this cutoff. We then define these combinations as positive TF-DNA interactions (Fig. 6, left panel). In contrast, the combinations with less than 2 biological and 8 technical replicates would then be regarded as no TF-DNA interactions (Fig. 6, right panel).

### Output file format and selection

After the identification of positive TF-DNA interactions, GM_Basic can convert the results into a CSV file (Additional file 1: Fig. S7). The positive and negative TF-DNA interactions would be shown as P and N, respectively. The sample names are listed on the left column and the treatment category is placed on the title row (Additional file 1: Fig. S7b). Beside the result files, the users can also output a fold-change file, showing the signal fold-changes between each sample and the reference (see Additional file 2). Outputting the result files and the fold-change files are optional, and the users can use the drop-down lists to activate or inactivate the options (Fig. 3).

## Results

### GateMultiplex-GUI: a friendly graphical user interface (GUI)

We designed two GUIs for GM_Basic and GM_Advanced of GateMultiplex to allow the users to analyze their data without the requirement of programming skills. In the following, we first describe the GUI (Fig. 3) and the functions of GM_Basic. *The GUI is mainly composed of three parts: Input file information, Cutoff setting, and Output files* (Fig. 3). We implemented several characters to simplify the use of our GUI. (1) Buttons (Fig. 3a, blue boxes). Clicking the buttons would pop up the corresponding windows (Fig. 3b–d). The GUI can identify different factors from the user-provided files, and then compile the factor information into the pop-up windows to allow the users to choose based on their own needs. Once the parameters are all set, clicking the “GO!” button can start the analysis (Fig. 3a). (2) Drop-down lists. This list provides several fixed options and allows user to easily recognize their needed ones (Fig. 3a, d). For example, the users can activate or inactivate the output file types (Fig. 3a). (3) Dialog boxes. Our users can directly enter numbers or words into the boxes (Fig. 3d, orange boxes). The operating details are described in the Additional file 2, 3, and 4. A warning system was implemented into our GUI to prevent the users from entering incorrect information. For example, the GUI would show the total file numbers imported into GateMultiplex to allow the users to ensure the incorporation of all required files. Some boxes only allow entering numbers, and the GUI would pop out a warning if the users accidentally input characters (please see the details in the Additional file 2).

### The workflow of GateMultiplex

**Fig. 7:**
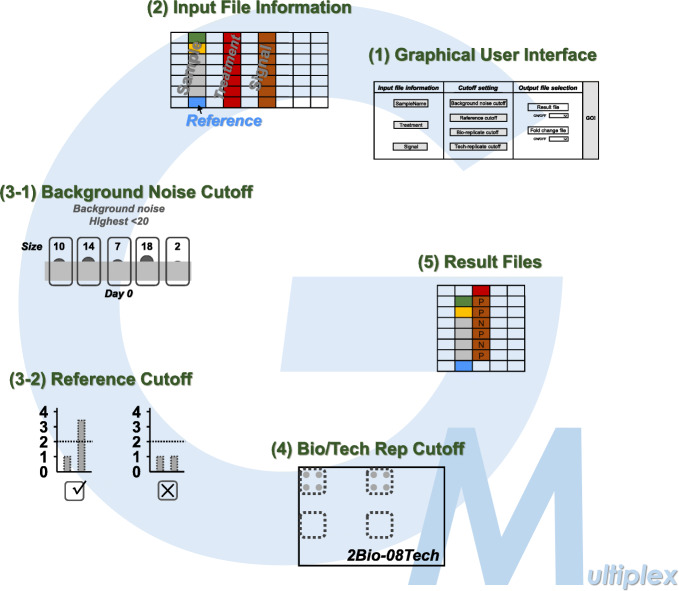
GateMultiplex workflow. In general, the workflow of GateMultiplex includes five steps. (Step1) The operation of the graphical user interface. (Step2) The identification of required information from the input files. (Step3-4) Different cutoff value settings. (Step5) The generation of result files

In summary, the whole analyzing procedure of GateMultiplex is composed of 5 steps (Fig. 7). Step1: Using GUI to enter the parameters; Step2: Extracting the information from the input files; Step3-1: Removing the result signals lower than background noise cutoff; Step3-2: Exerting reference cutoff through selecting the range of the ranking and the fold-change; Step4: Determining the positive TF-DNA interactions using bio-/tech-replicate cutoff; Step5: Generating the output files. The operating details are described in the Additional file 2, 3, and 4.

Beside Y1H analysis, GateMultiplex can also be applied to analyze large-scale data from numerous fields in life science. We next use three fields to demonstrate the analyzing power of GateMultiplex. (1) Lead identification in preclinical cancer drug discovery; (2) precision agriculture for crop line selection and harvesting time decision; (3) detecting ocean pollution generated from deep-sea fishery. Along with the description of the analysis of these fields, we will demonstrate more complicated analyzing situations, which can also be processed by GM_Basic. The drug discovery part will be used to show more complex signal format from HDF plates. Precision agriculture and deep-sea fishery analysis can explain the situation of encountering the input source beyond HDF plates, generating multiple signals, and multiple sample names from a single sample.

### Preclinical lead compound identification—complex signal source formats

**Fig. 8:**
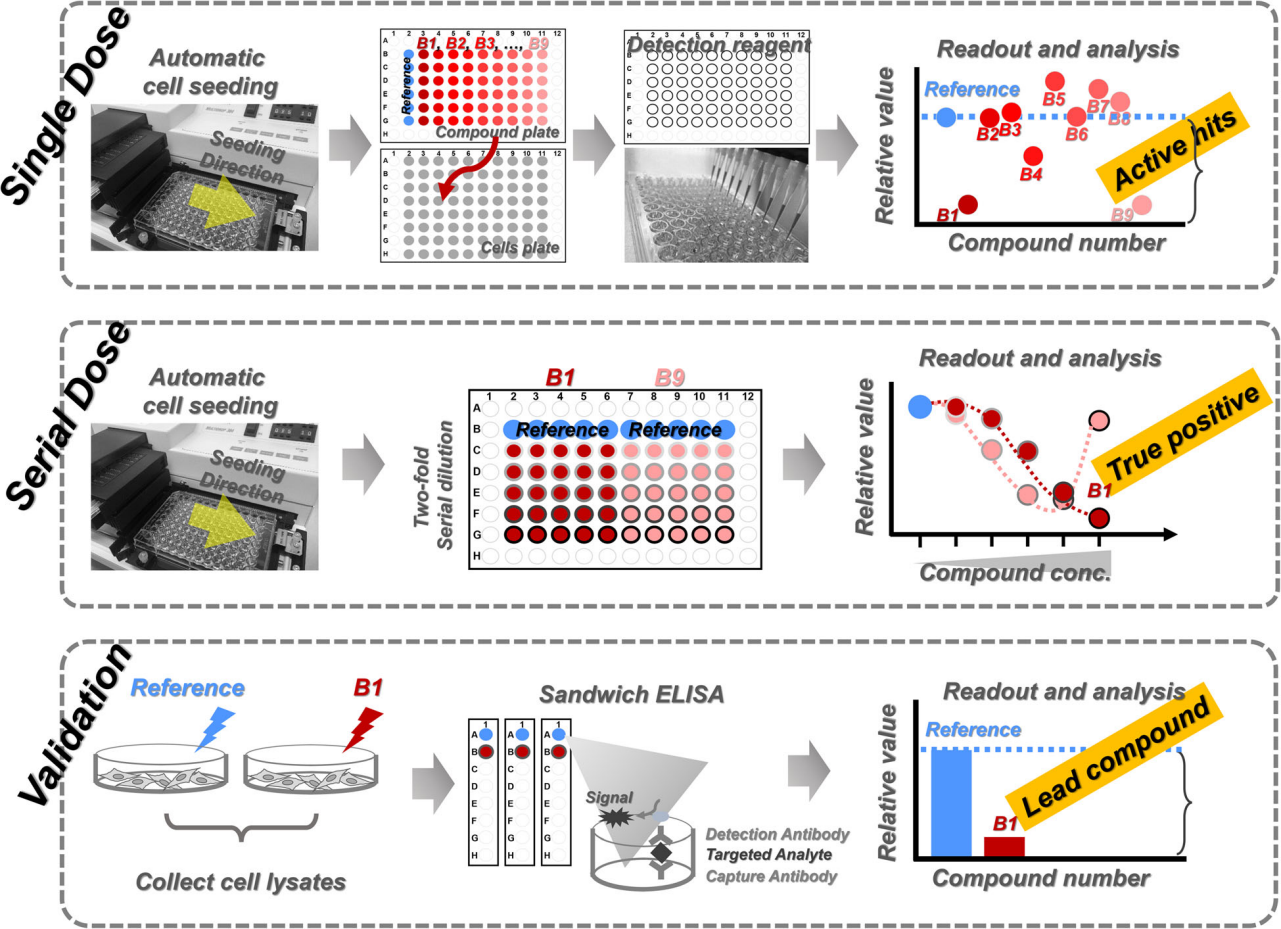
Preclinical lead compound identification. The process of lead compound identification includes three phases, single-dose screening, serial dose screening, and potential compound validation. In single-dose screening phase, cells are automatically seeded into each well on the plates. The 9 different compounds (compound B1 to B9 colored from red to pink) would be used to treat the cells. The wells with blue color represented the reference as no compound treatment. After culturing, the detection reagent would then be added. The relative cell viability of each well would be detected. If a compound can suppress the cell viability, these compounds would be regarded as active hits. The active hits, here as B1 and B9, would further be proceeded to the serial dose phase. The B1 (red) and B9 (pink) compounds were treated to the cells using serial dose. The darker circles around the red or pink dots represented the higher compound concentration. The B1 compound showed inhibited the cell viability along with the increased concentration, which would be determined as a true positive. The B1 would then be validated in the potential compound validation phase. The cell lysates from B1 treatment or reference were then collected for the sandwich ELISA. The cells treated with B1 showed lower value than reference, demonstrating B1 as a lead compound

**Fig. 9:**
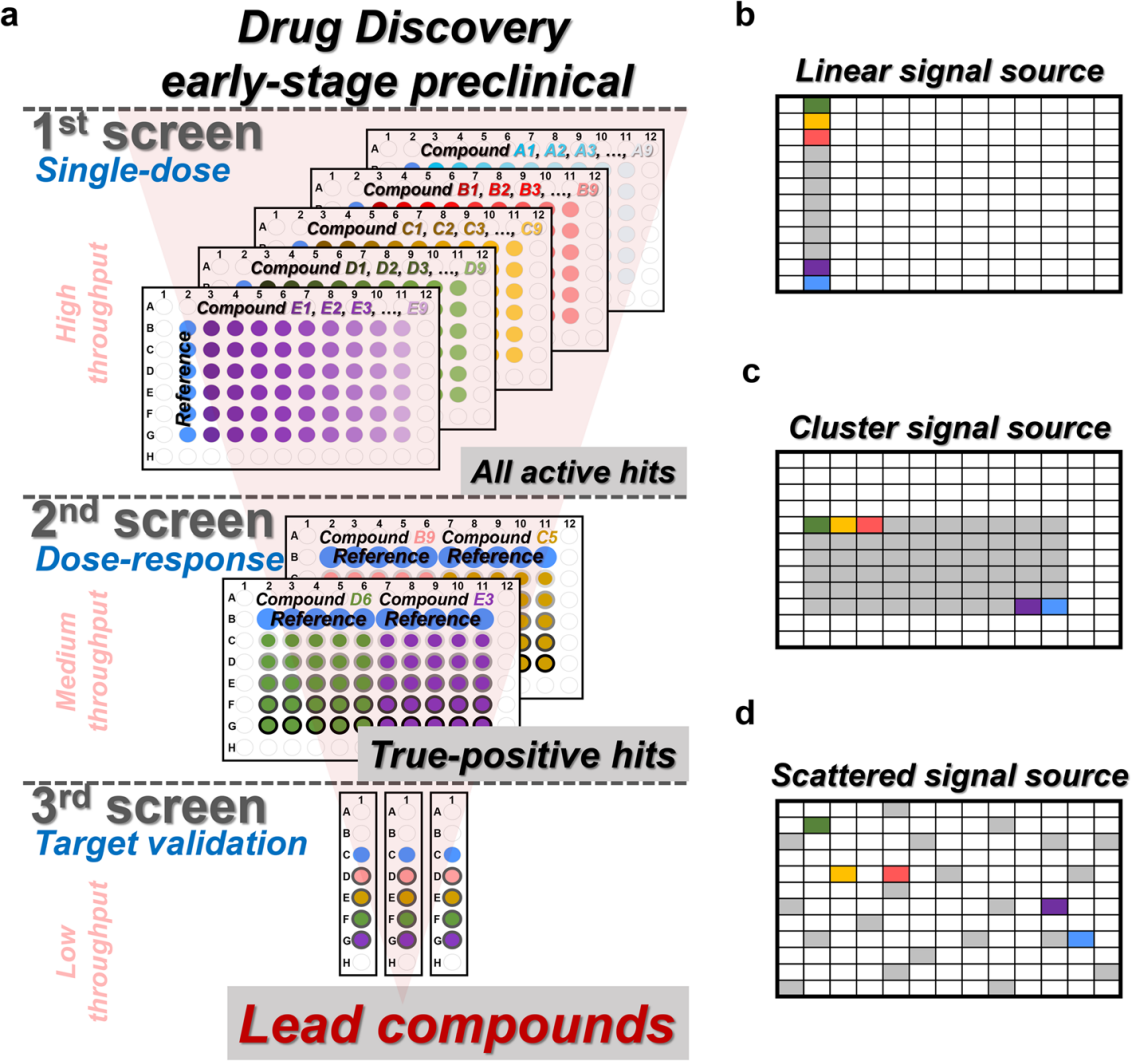
Signal source formats. **a** Preclinical lead compound identification is the early stage in drug discovery and is composed of three screening phases. The first phase is a high-throughput screening process. Large compound types are detected in single dose, and the active hits would be selected. The second phase is a medium-throughput screening. The active hits selected from single-dose screening would be tested in serial doses to select the true-positive hits. The third phase is a low-throughput screening for validation of lead compound identification. **b–d** Different platforms, such as the high-throughput, the medium-throughput, and the low-throughput platforms, can generate different signal formats. Common formats include **b** the linear signal source and **c** the clustered signal source. The signal files generated from preclinical lead compound identification are usually in the clustered format. **d** Scattered signal source is another kind of signal format

New drug development is basically composed of the discovery phase and development phase. In the discovery phase, the identification of lead compounds is the initiating stage and plays a crucial role to determine the whole-time frame of the drug development [28, 29]. The identification process incorporates three assays (Fig. 8), and here we used the drug development for cancer treatment as an example: (1) Single-dosage screening to obtain the active hits (Additional file 1: Fig. S8 and S9). The cancer cell suspension was seeded in 96-well plates from 2nd to 11th columns (Additional file 1: Fig. S8a) followed by the treatment of different compounds (Compound A1 to A9 in Additional file 1: Fig. S8b). The amounts of living cells in each well were then quantified to obtain the cell viability (Additional file 1: Fig. S8c). Through the comparison between the reference and compound treatment, the active hits were identified (Fig. 8). The sample names represented different compounds (Additional file 1: Fig. S8b), the cell amounts in each well were quantified as signal (Additional file 1: Fig. S8c), and the treatments are defined as different compound plates (Additional file 1: Fig. S8b and S8d). Total six technical replicates were carried out for each compound and reference (without compound) (Additional file 1: Fig. S9a). Through comparing to the reference (Additional file 1: Fig. S9b), the compound with more than or equal to four positive technical replicates were determined as an active hit (Additional file 1: Fig. S9c); (2) Serial-dose screening for the true-positive hits (Additional file 1: Fig. S10 and S11). The active hits were used to treat the cells by serial dosages (Additional file 1: Fig. S10). We seeded the cells again in 96-well plates (Additional file 1: Fig. S10a), and each plate was used to test two active hits (Compound A1 and A9 in Additional file 1: Fig. S10b and S10d). Five different dosages were used for each tested active hit, and each dosage was carried out with 5 technical replicates (Additional file 1: Fig. S10b, S10c and S11a). During the GateMultiplex calculation, these five technical replicates of each dosage were normalized by the average of the reference (Additional file 1: Fig. S11b). If the cell viability decreases along with the increased compound concentration (Compound B1, Fig. 8), then such compound would be considered as the true positives (Fig. 8 and Additional file 1: Fig. S11c); (3) Potential drug target validation (Additional file 1: Fig. S12 and S13). The true positives were further proceeded to functional examination using, for example, the enzyme-linked immunosorbent assay (ELISA) (Additional file 1: Fig. S12). Three biological replicates/batches each with two technical replicates were used (Additional file 1: Fig. S12a and S13a) followed by the quantification of protein phosphorylation level (Additional file 1: Fig. S12b and S13a). Through the comparison with reference (without compound) (Additional file 1: Fig. S13b), a true positive would be determined as a potential lead if all two technical replicates and three biological replicates are positive (Additional file 1: Fig. S13c and 13d). Since up to 100,000 compounds per day can be involved in the single-dosage screening, high-throughput and robotic platforms, including automated cell seeding machine (Fig. 8, automatic cell seeding), were extensively used among pharma companies and academics. These three assays serve as a hit/lead-finding strategy and narrow down the large-scale compound libraries to hits or even leads (Fig. 9a).

This hit/lead-finding strategy is also performed using HDF plates, such as 96- or 384-well plates. Unlike the linear signal source format from other high-throughput platforms (Fig. 9b and Additional file 1: Fig. S5), the machines for three hit/lead-finding assays usually provide cluster signal source format (Fig. 9c). Besides processing the linear format, GateMultiplex can also recognize the cluster format. Moreover, even the scattered signal source format can be processed (Fig. 9d, see Additional file 2). *GateMultiplex can intake various signal sources (linear, cluster, scattered formats) from HDF plates.* In the next two fields, agriculture and fishery, we will demonstrate the analysis of even more complicated signal source formats beyond HDF plates. Comparing to Y1H and drug development using fixed format plates, the data in agriculture and fishery are outputted in numerous formats due to the sources from different machineries or databases. GM_Basic can process the signal sources from both fixed format as HDF plates and various formats demonstrated in the followings.

### Phenomic screening in precision agriculture—multiple signals from one sample

**Fig. 10:**
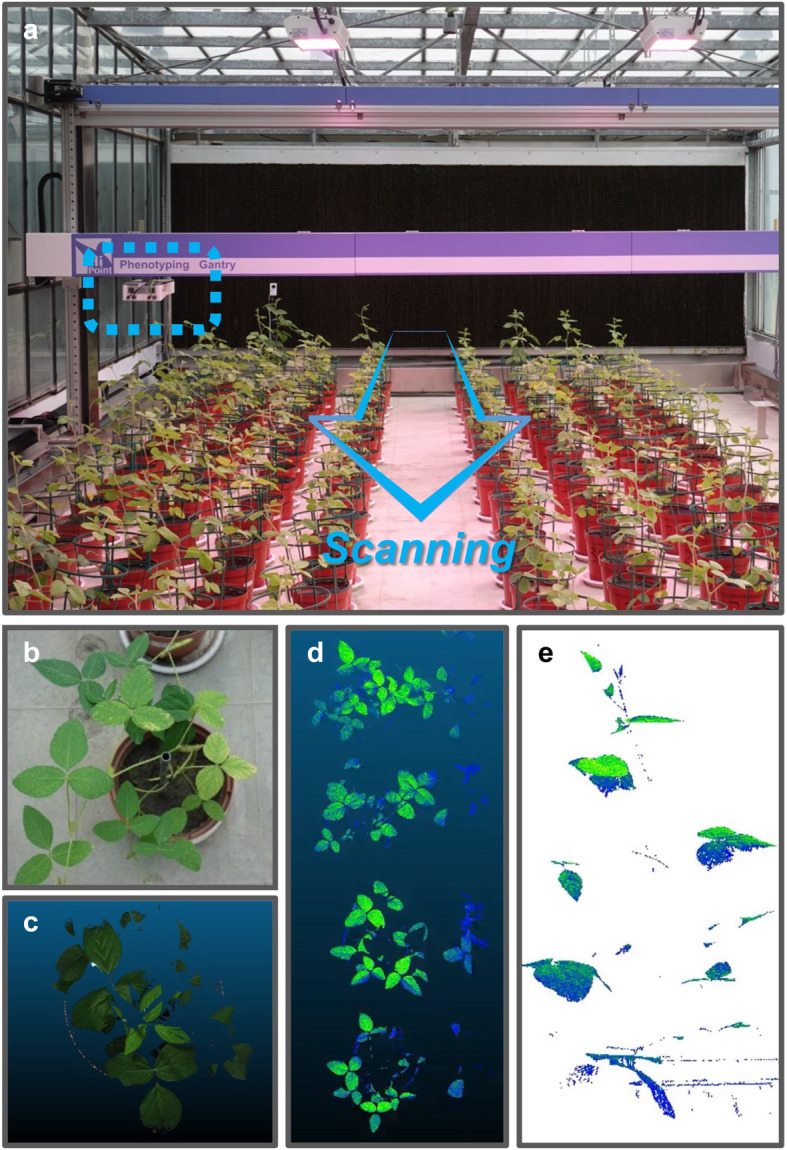
Multiple signals in precision agriculture. **a** Different soybean lines were arranged in a greenhouse. The high-throughput phenomic screening system is composed of a mobile camera (indicated by the blue dashed box) and the automatic gantry. The system scanned through the arranged soybean plants along the column direction (blue arrow), and recorded different kinds of images, including **b** RGB photos, **c** 3D scanning images for individual plants, **d** the images with the whole rows of plants, or **e** with further processing

**Fig. 11:**
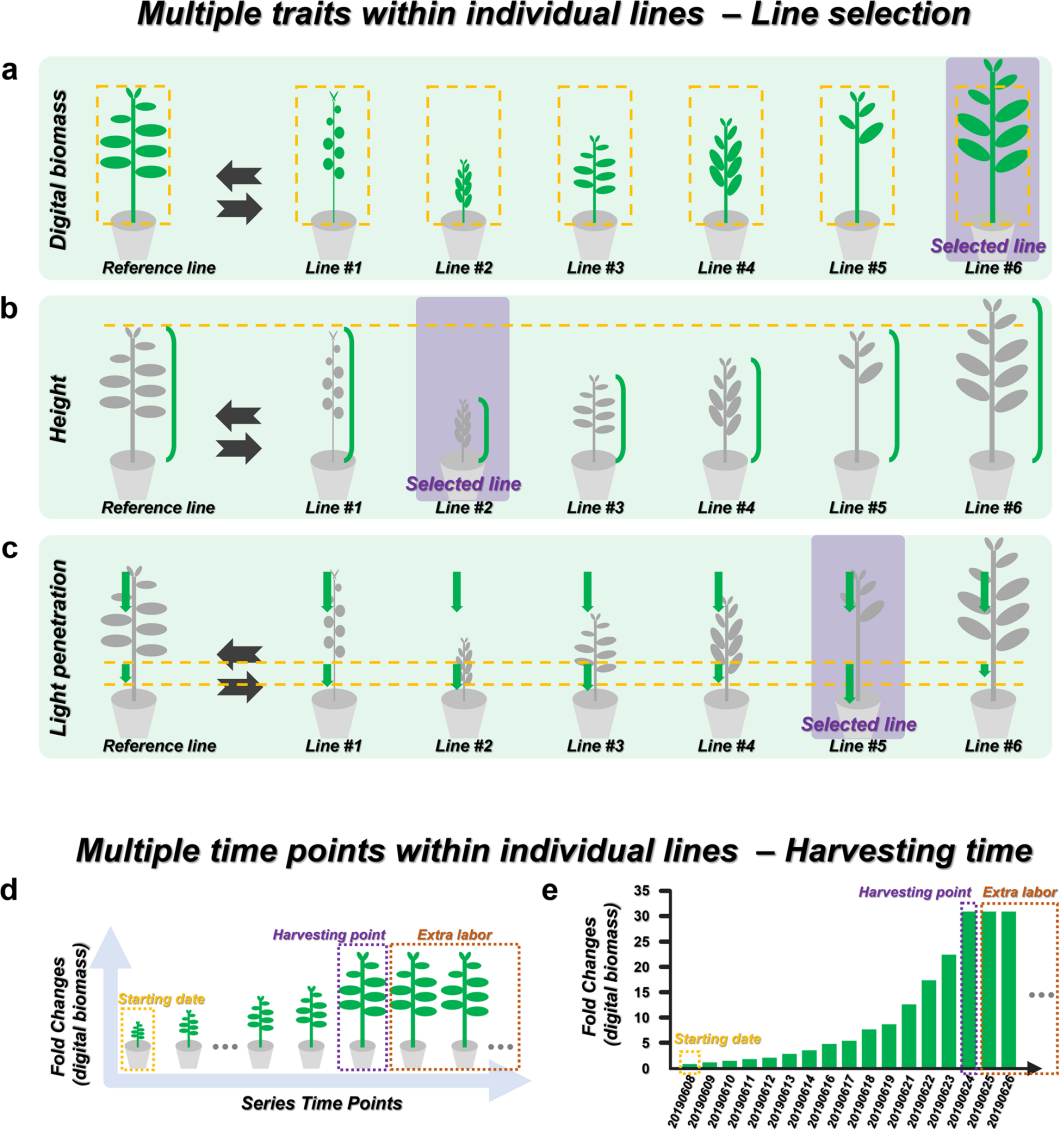
Plant line selection and harvesting time. **a**–**c** Multiple traits can be used for plant line selection, including **a** digital biomass, **b** height, and **c** light penetration. The lines (from line #1 to line #6) would be compared to a reference line to select the lines with required phenotypes. The plant lines with purple background were the selected lines. For example, **a** line #6 was selected because of its largest digital biomass. **b** Based on the height, line #2 with the shortest stem was selected. **c** In the light penetration trait, light remained the most after penetrating through the leaves of line #5, so line #5 was selected. **d**, **e** During **d** the plant growth period, **e** the plant biomass would enlarge throughout the growing stage. When the plants reach their harvesting point (purple dashed box), the biomass would no longer increase. If the plants are not harvested after this harvesting point (purple dashed box), extra labor (orange dashed box) would increase the cost and decrease the farming efficiency

Precision agriculture integrates series intelligent technologies, including phenomic screening, agrochemical management, and decision support systems to efficiently optimize the crop yield [30–32]. Phenomic screening usually uses a high-throughput sensor composed of a mobile camera (Fig. 10a, marked in the blue dashed box) and its supporting device to allow this camera scanning through the arranged plants (Fig. 10a). The sensor can record the morphological and architectural parameters from the plants, such as the RGB photos (Fig. 10b) and 3D scanning images from individual plants (Fig. 10c), a whole row of plants (Fig. 10d), or with further image processing (Fig. 10e). The device would then incorporate the parameters and images and convert into different critical traits for the crop breeding to select better lines. The traits include digital biomass [33–35] (Fig. 11a), plant height [36, 37] (Fig. 11b), light penetration depth [38–40] (Fig. 11c). Take a schematic example, different traits of each crop line would be quantified throughout the culturing period (Additional file 1: Fig. S14). In line selection, the crop names would serve as sample names, the quantified traits represent signal, and different dates are defined as treatments (Additional file 1: Fig. S15a and 15b). Comparing to the reference line, the lines with better traits would be selected by user-assigned cutoff (Additional file 1: Fig. S15c). Phenomic screening can also instruct the harvesting time (Fig. 11d, e, Additional file 1: Fig. S16). By scanning through the plant growth period (Fig. 11d), we would record the earliest time point of the maximum plant biomass or crop yield (Fig. 11d, e). In this time-based case, culturing dates can be regarded as sample names and crop line names can be treatments (Additional file 1: Fig. S16a). Through the fold-change output file (Additional file 1: Fig. S16b), an optimal harvesting time can be decided by the users (Additional file 1: Fig. S16c). With the information of optimized harvesting time, extra labor and farming can be avoided (Fig. 11e) to increase the agricultural efficiency.

Both line selection and harvesting time decision would output multiple traits or time points from each plant, which means that one sample may have multiple signals. GM_Basic can compute multiple signal sources from one sample (Additional file 1: Fig. S17a, dark and light brown columns) and provide the corresponding results (Additional file 1: Fig. S17b).

### Geographical tracking for marine ecosystems—multiple sample names for one sample

Ocean environment has suffered from unsustainable pressures, including pollution and overfishing, for years due to rapidly evolving human activities, such as seaborne trade, fishing, and recreation [41, 42]. One of the major pollution sources is from long vessel sailing time which results in shipping emissions of pollutants, and regions with frequent high shipping pollutants are under threat of climate change, low air quality, and health effect [43, 44]. These pollutants, such as noise and artificial light, severely increase the fish mortality and disturb the seabed habitat [45–49]. Overfishing, as the other global issue, causes substantial declines of fish species abundancy and individual fish populations, which would further jeopardize the marine ecosystems and economy [50, 51]. Deep-sea fishery is a long-term commercial fishing activity sailing far away from the shore with high economic value. The targets of deep-sea fishery are usually the less population fishes which have lower productivity and slower growth rates, playing crucial roles in marine ecosystems [52, 53]. For sustainable deep-sea fishery economy, deep-sea fishing activity is detected and recorded globally. From an environmental and management perspective, understanding vessel hours and fishing hours and their relationships may provide an opportunity to estimate the strength of potential fishing activities and pollution production.

**Fig. 12:**
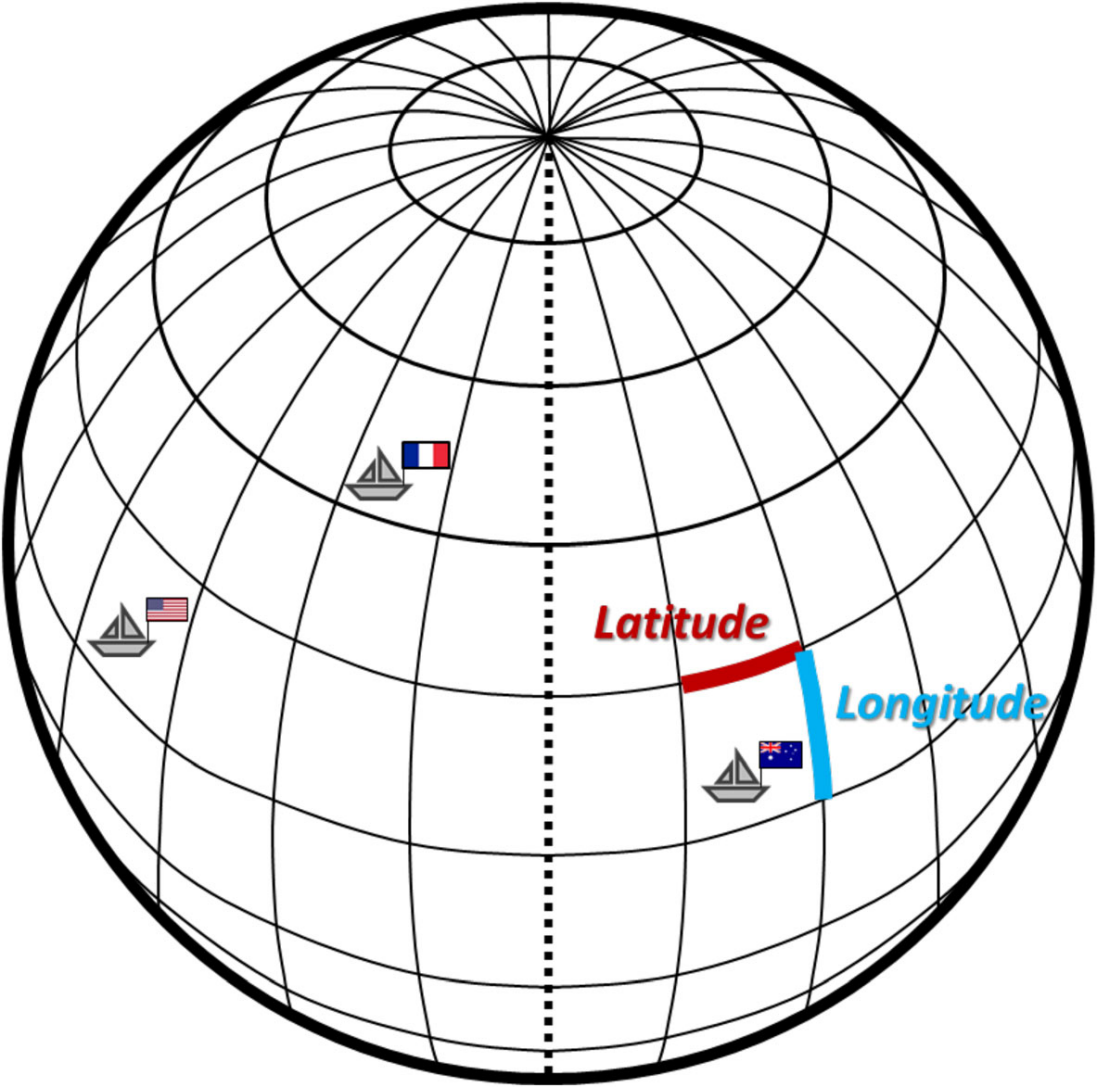
Multiple sample names in deep-sea fishery. The earth surface can be divided into different grids. Each grid is represented by a set of latitude (colored in red) and longitude (colored in blue). In deep-sea fishery, the ships are fishing on the offshore area which is far away from the shore. Through the latitude and longitude combination, the grids can be used to report the ship location

Global recording of deep-sea fishery parameters includes fishing hours, positively related to fishing amounts, and vessel hours, resulting in shipping emissions, as well as fishing date, and country names for each earth grid. Every grid can be identified by providing its latitude and longitude (Fig. 12), which means multiple sample names (latitude and longitude) for one sample (each grid). GM_Basic can integrate multiple sample names (sample-1 and sample-2 columns in Additional file 1: Fig. S17c) into one name (Additional file 1: Fig. S17d). In each grid (Fig. 12), the activity of all ships from different countries using different gears was recorded (Additional file 1: Fig. S18), so the sample names would be even more complicated when taking the consideration of the ships from different countries loaded with different fishing gear types (Additional file 1: Fig. S19a). Comparing to a user-assigned reference (Additional file 1: Fig. S19b, c), the positive results represent the grids, which might be polluted by the high vessel hours of the ships from certain countries using certain fishing gears (Additional file 1: Fig. S19c). The processed results thus can serve as the informing of overexploitation risks to marine resources.

We provided the real experimental results of these four fields (Additional file 5), preclinical lead compound identification, phenomic screening in precision agriculture, geographic vessel tracking of deep-sea fishery, and the Y1H screening [2], for the users to test the functions and operation of GateMultiplex.

### Yeast one-hybrid for TF-DNA interaction—multiple treatments for one sample

Beside multiple sample names and signals for one sample, the users may also encounter multiple treatment categories for one sample. In Y1H, different DNA-baits, incubation time, and screening methods could be used to compare different platforms. Within one treatment, different “conditions” would be used. For example, incubation time is a type of treatment, and incubating for 1, 2, and 3 days are the three conditions for this treatment (Additional file 1: Fig. S20). More treatments can always be incorporated to the existing treatments to create further combinations (Additional file 1: Fig. S21 and S22). In summary, GM_Basic can process different input source format, multiple sample names, treatments, and signals for a single sample to adapt more complicated experimental design (Additional file 1: Fig. S17e, f).

### GM_Advanced—more cutoff types and functions for advanced analyzing needs

**Fig. 13:**
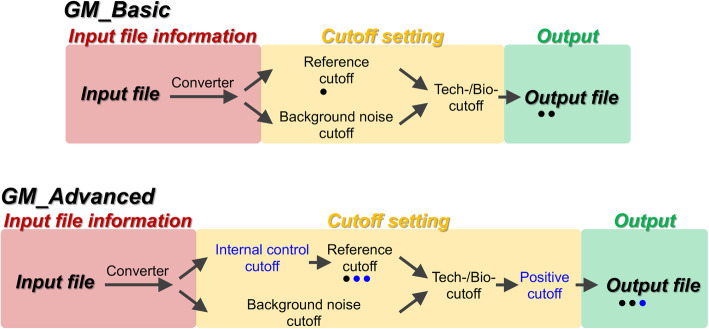
Procedure of GateMultiplex operation. The operation of GM_Basic and GM_Advanced can be divided into three parts, including input file information (in red background), cutoff setting (in yellow background), and output (in green background). The black dots under reference cutoff and the output file represent the numbers of the options. In GM_Basic, the operation starts from an input file, which would be converted into the required data format. The data would then be respectively analyzed by two cutoffs, reference cutoff, and background noise cutoff. The results from two cutoffs would be combined and further processed by tech-/bio-cutoff. The final results would be outputted as the result files. The operation of GM_Advanced is similar to that of GM_Basic. The blue words and dots in GM_Advanced represent the additional cutoffs or options. Internal control cutoff exerts before reference cutoff, and reference cutoff contains two more options (see Additional file 2 for the details). After being analyzed by tech-/bio- cutoff, the data could further be processed by positive cutoff. In the output file, one more file option is available

We further designed GM_Advanced for the users to apply more types of cutoffs and functions to fulfill their advanced experimental designs. Comparing to GM_Basic, GM_Advanced provides two additional cutoffs: internal control cutoff and positive cutoff (Fig. 13), and also has its own GUI to match each of the functions (Additional file 1: Fig. S23). *Using internal control cutoff*, the users can eliminate more bias caused by the inconsistency among different experimental batches. In Y1H screening, the experimental groups are the yeast colonies growing on the selection plates using, for example, antibiotics (Additional file 1: Fig. S24, right panel). An internal control group could be conducted with no antibiotic selection (Additional file 1: Fig. S24, left panel). Without any selection pressure, all yeast colonies were supposed to grow successfully on the internal control plates. If a yeast colony could not grow on the internal control plates, then the experimental results of this yeast colony should be excluded (Additional file 1: Fig. S24c) due to potential operating errors or inconsistent experimental performance. *Using positive cutoff*, the users can further adjust the selection stringency. In Y1H, the selection plates are usually incubated and recorded for several days. The results from each incubation day may vary according to the growth difference between the yeast with TF and empty vector (Additional file 1: Fig. S25). A same TF-DNA combination may be regarded as a positive interaction on day 1 and day 2, but also could switch to a negative on day 3 (Additional file 1: Fig. S25a). If positive cutoff was set as 2, then a TF-DNA combination would be evaluated as a positive, as long as this combination is counted as a positive in at least two incubation days (Additional file 1: Fig. S25). In precision agriculture, the farmers would select crop lines based on many important phenotypes (Additional file 1: Fig. S26). Different crop lines would have different required phenotypes, such as large biomass, short height, high light penetration, high greenness, small leaf angle, or large lead area (Additional file 1: Fig. S26), and one crop line may have more than one required phenotype (Additional file 1: Fig. S26, line#2 and #6). If a farmer wants to have a crop line with at least two required phenotypes, then positive cutoff could be set as 2. We also added two more options for reference cutoff and one more output file format in GM_Advanced (Fig. 13, blue dots) (see Additional file 2 for the details). To facilitate the operation of GateMultiplex, we provided detailed and step-by-step manuals for the users to conduct the analysis using GM_Converter, GM_Basic, and GM_Advanced for different fields (Additional files 3, 4, and 5). With 5 types of the cutoffs, 3 different output file formats, and an input file format converter, we wish GateMultiplex can meet the needs of the users from various fields in life science research (Fig. 13).

## Discussion

HDF plates are useful and necessary tools in life science field and have been applied extensively to numerous techniques and organism culture [2, 54–60]. The techniques adopted HDF have been developed to handle nucleic acids and proteins, such as yeast one-hybrid [2, 7–12] and enzyme-linked immunosorbent assay [61–63]. Various platforms have been established to culture different organisms in HDF plates, including virus [55], bacteria [57], fungi [58], yeast [2], algae [60], and cells from plant [56], animal [54], and human [59]. In addition, HDF plates have been coupled with many high-throughput platforms to further enlarge the screening scales, e.g., the 384- and 1536-liquid-handling system [64, 65], and the 384-, 1536-, and even 6144-arraying system [2, 66, 67].

Due to the scale rising dramatically from many HDF-high-throughput coupled systems, the amounts of their output data, usually in text-based formats, have also increased to a scale that the analysis is too difficult to perform manually. Thus, programming languages have become necessary to assist the analysis to avoid the errors created by manual operation, to maintain the robustness, and to accelerate the analyzing speed. C++ is one of the fastest common programming languages used in bioinformatics, comparing to Java, Perl, and Python [24–27]. Besides, C++ requires the least memory while performing the computation [27]. Using C++ for processing the data can provide a faster platform without high demand on the computing power. We used the data generated from our previous Y1H study [2] to test the analyzing speed of our C++ programs. We used 840 384-format plates to compare three Y1H systems, and used 80 384-format plates for the reproducibility test, yielding a total of 920 plates needed for analysis (840 + 80). The analysis of 920 plates represents the cross-comparison among 353,280 text-based numbers (920 × 384). Using C++ programs, we only need ~ 25 s to compute these data (an Intel Core i5 CPU at 1.7 GHz and 4 GB RAM). In contrast, if Java, Perl, or Python were used, then the estimated processing time would increase to approximately 40-fold based [27], which is up to ~ 17 min. The 920 plates of our previous Y1H screening were used to analyze the TF-DNA interactions to construct a relatively small-scale gene regulatory network (GRN) in a woody model plant, *Populus trichocarpa*. This GRN is composed of the results from screening 92 xylem-specific TFs against 7 secondary cell wall biosynthesis genes. In human, worms, flies, and herbaceous plants, GRNs involved in different developmental and differentiation pathways have been studied using Y1H to screening the interactions among thousands of TF-preys against hundreds of DNA-baits [2, 7–12]. In this large scale, we estimate to finish the data processing in 7 h using C++ programs, but other programming languages may need around 12 days (around 40 folds [27]). The results generated from such large-scale screening apparently require heavy computing power and time, and our newly developed GateMultiplex would serve as a convenient tool for such analysis.

In addition to the high processing speed, GateMultiplex also provides high flexibility for the users to customize the cutoff values based on the requirement of their experimental design. If any of the cutoffs are not necessary or available, the users can disable the cutoff functions by entering 0 as their values. For example, in our previous studies on drug screening using cell proliferation assay, the internal controls were not necessary. The cells were first seeded equally in each well of every plate. Once the cells all grew evenly in the plates, the drugs were then treated. The growth of the cells, the internal control, can be easily visualized by eyes, and further detection or analysis is not necessary. Furthermore, each plate had their own negative control groups, so inter-plate comparisons are also not required, which also shows that the internal controls are not required in this case. GateMultiplex also allows the users to locate their negative control groups in different places on the HDF plates to avoid the bias caused by the plate types and material. HDF plates are manufactured into many different types, such as well plates sealed using caps or films, and arraying plates using the formats by robotic pinning machine. Same types of HDF plates may also be produced using different material, leading to diverse application. Transparent, white, and black 384-well polystyrene plates are used for the detection optical density, luminescence, and fluorescence, respectively. In the HDF plates, the signal or the liquid evaporation are affected severely by their spatial locations of the plates, especially at the plate edge [68–70]. For example, yeast colonies grown on the agar plate edges without the surrounding by other colonies tend to grow much faster due to the access to more nutrients from the medium [71]. Such phenomenon is known as the “edge effect,” causing inaccurate analysis especially when the negative control groups locate at the edge. HDF plates with different types and material are affected by the edge effect in different ways. The flexibility of GateMultiplex on the negative control groups assists the users to freely assign the negative location to minimize the data instability created from edge effect.

GateMultiplex also has high applicability to be converted into a version fitted to user needs. Since the most common used HDF plates in high-throughput systems are 384- and 1536 formats, in this study, we designed GateMultiplex to support these two formats. Other HDF formats, such as 24, 96, and 6144, can be easily incorporated into GateMultiplex. Within each format, the experimental design can also be customized by the users. If 4 biological replicates each with 4 technical replicates are not necessary, the replicate numbers can be reduced in GateMultiplex into 4 biological replicates each with only 1 technical replicate or 1 biological replicates with 4 technical replicates. The stringency of the control fold-change cutoff can also be adjusted easily. In our Y1H example, we applied the average of 5th to 12th rank of the negative yeast colonies as the cutoff value (Fig. 5c). The stringency can be increased by using the average of the high rank negatives, e.g., 1st to 12th or even 1st to 6th. Instead, the cutoff values can be reduced to lower the stringency then allow more replicates to pass the cutoff as positives.

We have demonstrated the high speed, flexibility, and applicability of GateMultiplex with user-friendly GUIs. We compared GateMultiplex to the two most widely used software, SpotOn [10] and TIDY [11], from different aspects, including their programming languages, GUI, input data format, parameter setting, output file, and their application (Table 1). For the programming languages, GateMultiplex with high computing performance using C++ to deal with nowadays fast-growing data amounts generated from various high-throughput platforms. SpotOn and TIDY were written in scripting language Perl and Matlab, respectively, with much slower working speed than C++. In addition to the speed, being user-friendly is another critical requirement for the software development. We designed GUI in GateMultiplex to facilitate the operation, so the users would not need to dig into the source code to adjust any of the parameters. In contrast, coding ability is required to use SpotOn, and Matlab installation is required to operate TIDY. These two software tools were designed for a fixed input data format, and the incapability of processing diverse data formats generated from different experimental systems is one of the main reasons to limit their application to other systems. Another limiting main reason is the lack of flexibility for parameter setting, because the data from various experimental designs would require different types of parameter cutoff and their corresponding cutoff values for the analysis. SpotOn was used to process the data from Y1H as well as Y2H, and TIDY is a Y1H-specific analyzing tool due to limited input data format as well as low flexibility of parameter setting. For the output result files, TIDY does not provide a result file, and the users need to copy the results from their programming command lines, and SpotOn only generates one kind of result file. To cope with the limitation of fixed input data format, GateMultiplex was coupled with GM_Converter to transform the input data from various sources into a recognizable format for GateMultiplex. Comparing to one or two adjustable parameters in SpotOn and TIDY, GateMultiplex provides additional three types of parameters, which can be optional and adjustable through GUI. GateMultiplex provides three kinds of output files, including the result files, the fold-change files and the PNE files (see Additional file 2 for the details), which can be utilized easily or even for the further analysis. The high flexibility of input data format and parameter setting with many output file types of GateMultiplex allow the users, even with no programming skills, to customize the analysis of their experimental design from Y1H, drug development, precision agriculture, and deep-sea fishery. Such high flexibility further suggests the potential applications of GateMultiplex to other fields, such as yeast two-hybrid to screen protein-protein interactions and synthetic genetic array analysis to investigate genetic interactions.

**Table 1:** The comparison of GateMultiplex to two most widely used software

	Language	GUI^a^	Input file format	Adjustable parameters to identify positive events	Output files	Application
GateMultiplex	C++ (fastest)	Yes	Format-free	(1) Background noise cutoff (2) Reference cutoff (3) Bio/Tech replicates cutoff (4) Internal control cutoff (5) Positive cutoff	(1) Result file (2) Fold-change file (3) PNE file	(1) Yeast one-hybrid (2) Yeast two-hybrid (3) Drug development (4) Precision agriculture (5) Deep-sea fishery
SpotOn	Perl	NA	HDF plate format	(1) Reference cutoff^b^	(1) Result file	(1) Yeast one-hybrid (2) Yeast two-hybrid
TIDY	Matlab	NA	HDF plate format	(1) Reference cutoff^c^ (2) Bio/Tech replicates cutoff^d^	NA^e^	(1) Yeast one-hybrid

^a^The users can operate the GUI without further installing additional programming language user interface

^b^Named as *z* score cutoff in the original paper. The negative control value could be adjusted, but the fold-change value is not adjustable

^c^Named as background threshold in the original paper with the function similar to reference cutoff in this article. The fold-change value could be adjusted, but the negative control value is not adjustable

^d^Named as uniformity coefficient in the original paper with the function similar to Bio/Tech replicates cutoff. Only technical replicates can be processed but not biological replicates

^e^The output results only showed on the command lines

GateMultiplex is available on GitHub as the execution files. GateMultiplex can be operated by simple double-clicking without further installation requirement. Therefore, GateMultiplex can be used on the computers, which are under restricted user permissions for software installation, such as in the workspace or school. For some public computers, even if software is allowed to be installed, but would be removed after the operating system restart. The users would then need to install the software every time before their operation. In personal computers, the new installation of software may not be compatible with the already installed software, causing the inconvenience for the revision of current personal settings. GateMultiplex, without installation requirement, offers our users a convenient tool for the analysis.

## Conclusions

High-throughput platforms have been applied to various experimental design throughout life science researches. With the increasing output data, the analysis of million- or billion-scale text-based number sets can no longer be performed manually. In this study, we developed a fast-processing software, GateMultiplex, with high computing performance using C++ to provide a task-oriented platform for the analysis of the results generated by high-throughput platforms. We provide GUIs for the users to easily operate the analysis even without any programming skills. With many flexible parameter settings, the users from various fields can customize the analysis based on their experimental designs. User-friendly GUI, fast speed, flexibility, and applicability of GateMultiplex increase the project feasibility of the large-scale data analysis in life science fields.

## Methods

### Drug development: cell viability assay using single- or serial-dose drugs

The 3 steps of early-stage drug discovery, single-dose treatment, serial-dose treatment, and target validation, were used to identify the leads of signal transducer and activator of transcription 3 (Stat3) inhibitor. For single-dose treatment, the cell suspension of SNU-449, a hepatocellular carcinoma cell line, were prepared for automatic cell seeding process using MultiDrop® 384 (5840157, Thermo Fisher Scientific, Waltham, MA, USA). Approximately 1.6 × 10^3^ cells within 95 μl 5% fetal bovine serum (FBS) supplemented medium per well were evenly injected into a 96-well plate from column 2 to column 11 as a “cell plate.” After 24 h incubation, one reference (1% DMSO) and nine of 100 μM compounds (sorafenib, lenvatinib, nilotinib, tranylcypromine hydrochloride, A769662, GSK2879552, GSK-LSD1, BIX01254, and UNC0638) were freshly prepared in 5% FBS supplemented medium. Each compound was dispensed into 6 wells within one column as a “compound plate.” Each 10 μl compound solution in compound plate was then transferred to the “cell plate” using multichannel pipettes to generate the 10 μM working concentration. After 24 h compound treatment, 10 μl PrestoBlue™ cell viability reagent (A13262, Thermo Fisher Scientific, Waltham, MA, USA) was added in the middle 60 wells for 3.5 h. The cell growth of indicated compounds was compared to that of the reference to obtain the cell viability. For serial-dose treatment, sorafenib and BIX01254 were further chosen to analyze the dose-escalation effect. Approximately 1.6 × 10^3^ SNU-449 cells were seeded by MultiDrop 384 and incubated for 24 h. These two compounds with 0.9375, 1.875, 3.75, 7.5, and 15 μM or 0.1% DMSO were treated for further 24 h. PrestoBlue reagent was used for viability detection, and the cell viability results were shown in a dose-response curve.

### Drug development: drug target validation by ELISA

Approximately 3 × 10^5^ SNU-449 cells were seeded in 10-cm dishes with 10% FBS supplemented medium. After 24 h, culture medium was aspirated and replaced by 5% FBS supplemented medium. The cells were pretreated with interleukin-6 (25 ng/ml) for 30 min, and subsequently treated with the reference (0.1% DMSO) or indicated compounds (sorafenib and BIX01254, respectively) at 5 μM for another 24 h. The whole cell lysates were collected, and 10 μg proteins from each sample were used for analyzing the phosphorylation status of Stat3 using PathScan Phospho-Stat3 (Tyr705) Sandwich ELISA (#7300, Cell Signaling, Danvers, MA) according to the manufacturer’s manual.

### Precision agriculture: PlantEye high-throughput sensor phenotyping

The soybean lines used in this study were selected from the core collection developed by Taiwan Agricultural Research Institute. Plants were grown and arranged in the greenhouse at the temperature between 25 and 35 °C. The greenhouse was equipped with an automatic phenotyping system, including a 3D laser scanner, PlantEye F500 (Phenospex, Heerlen, The Netherlands), an image processing software, “HortControl” (ver. 3.3), and an automatic gantry. The system scanned the arranged plant lines at a speed of 30 mm s^−1^, and the distance between the plant and the scanner was about 40 to 50 cm. The recorded plant traits included leaf area, leaf area index, the projected leaf area, leaf inclination, plant height, greenness, hue, light penetration depth, digital biomass, Normalized Difference Vegetation Index (NDVI), Normalized Pigments Chlorophyll Ratio Index (NPCI), and Plant Senescence Reflectance Index (PSRI).

### Deep-sea fishery: vessel hours and fishing hours

The global dataset of fishing effort at a spatial resolution of 0.01° was compiled by the Global Fishing Watch Research program, collecting over 300,000 unique vessels in a given year with available automatic identification system (AIS) data, of which more than 60,000 are likely fishing vessels [72]. Date, location of the vessel, the flag state of the vessel, and gear type used by the vessel were recorded and recognized based on a manual review or from matching the vessels to registries. Vessel characteristics and activities, represented separately by vessel and fishing hours, were classified by the convolutional neural networks (CNN). The former was calculated as hours that vessels of this geartype and flag were present in this gridcell on this day while the latter was calculated as hours that vessels of this gear type and flag were fishing in this gridcell on this day.

## Availability and requirements

Project name: GateMultiplex Project home page: https://github.com/Woodformation1136/GateMultiplex Operating system(s): Windows 10 (64-bit) Programming language: C++ and Python Other requirements: None License: Free for academic use Any restrictions to use by non-academics: Commercial users please contact ycjimmylin@ntu.edu.tw

## Supplementary Information

**Additional file 1.** Supplementary figure S1-S26.

**Additional file 2.** Detailed concept illustrations during the operating steps of GateMultiplex.

**Additional file 3.** Manual-Y1H. A step-by-step manual for operating GateMultiplex on Y1H analysis.

**Additional file 4.** Manual-Agriculture/Drug discovery/Geographical tracking. A step-by-step manual for operating GateMultiplex on Agriculture/Drug discovery/Geographical tracking analysis.

**Additional file 5.** Dataset. Real experimental results of the four fields to demonstrate the operation of GateMultiplex.

## Data Availability

The GateMultiplex package, the source code and the additional file 5 are available at GitHub, and can be accessed using the following link. https://github.com/Woodformation1136/GateMultiplex [73]. The dataset supporting the conclusions of this article is included within the article and the additional file 5.

## Abbreviations

AIS: Automatic identification system
CNN: Convolutional neural networks
ELISA: Enzyme-linked immunosorbent assay
FBS: Fetal bovine serum
GRN: Gene regulatory network
GUI: Graphical user interface
HDF: High-density-formatted
NDVI: Normalized Difference Vegetation Index
NPCI: Normalized Pigments Chlorophyll Ratio Index
PSRI: Plant Senescence Reflectance Index
Stat3: Signal transducer and activator of transcription 3
TF: Transcription factor
Y1H: Yeast one-hybrid
Y2H: Yeast two-hybrid
