# Supplementary material for: Large-scale data analysis for robotic yeast one-hybrid platforms and multi-disciplinary studies using GateMultiplex
Source: BMC Biol. 2021 Sep 24;19:214. doi: 10.1186/s12915-021-01140-y (PMC8461970; doi:10.1186/s12915-021-01140-y)
Supplement: Supplementary file 1 — Additional file 1. Supplementary figure S1-S26. [file 12915_2021_1140_MOESM1_ESM.pdf]

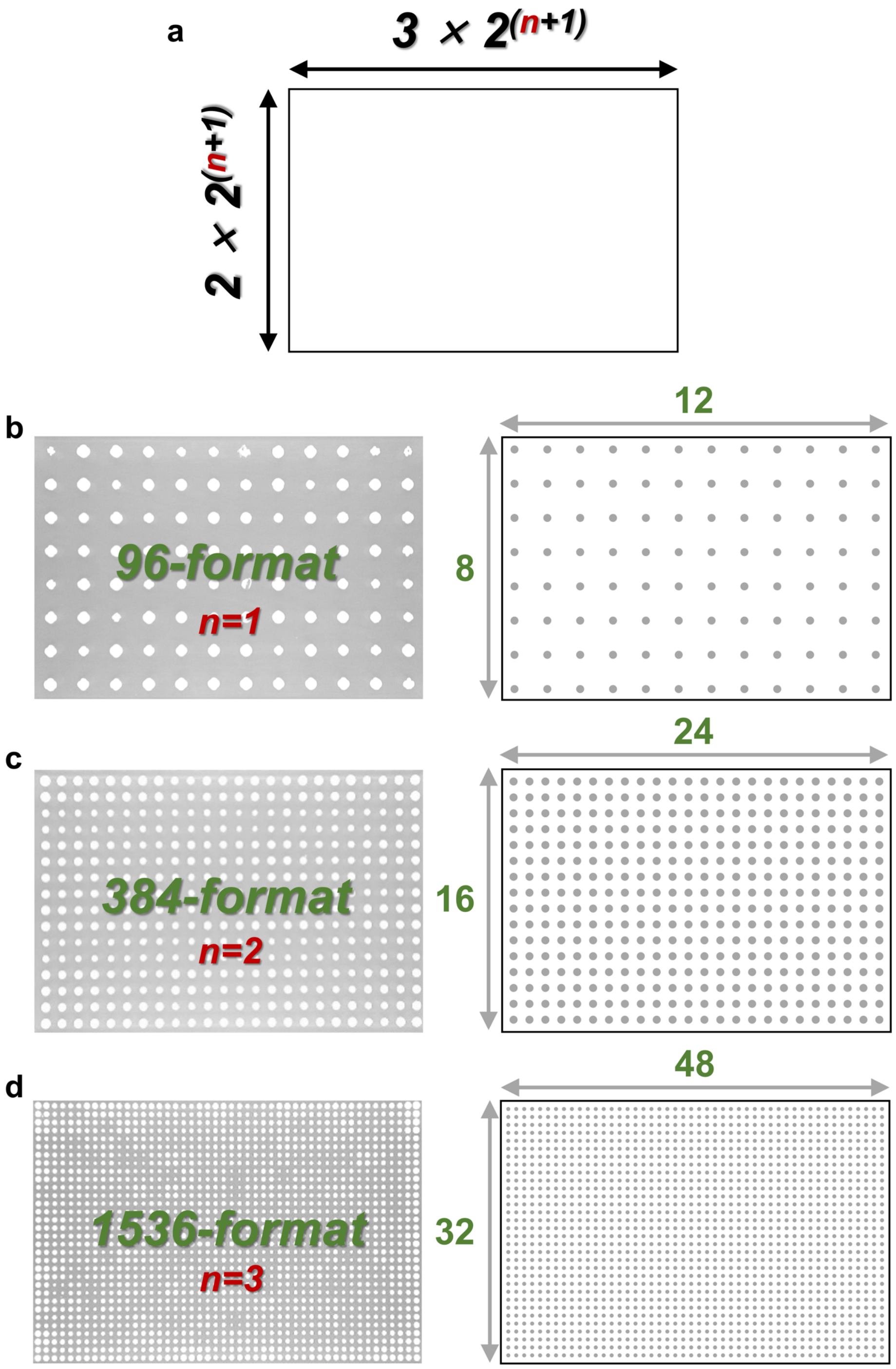

Figure S1. High-density-formatted (HDF) plates. a A HDF plate can be represented as the [3×2(n+1)] (length) × [2×2(n+1)] (width) format. **b-d** For example, (**b**) when 1 is substituted into the n, the result is [3×4] × [2×4] as a 96-format HDF plate (12 × 8). **c** When 2 is substituted into the n, the result is [3×8] × [2×8] as a 384-format HDF plate (24 × 16). **d** When 3 is substituted into the n, the result is [3×16] × [2×16] as a 1536-format HDF plate (48 × 32).

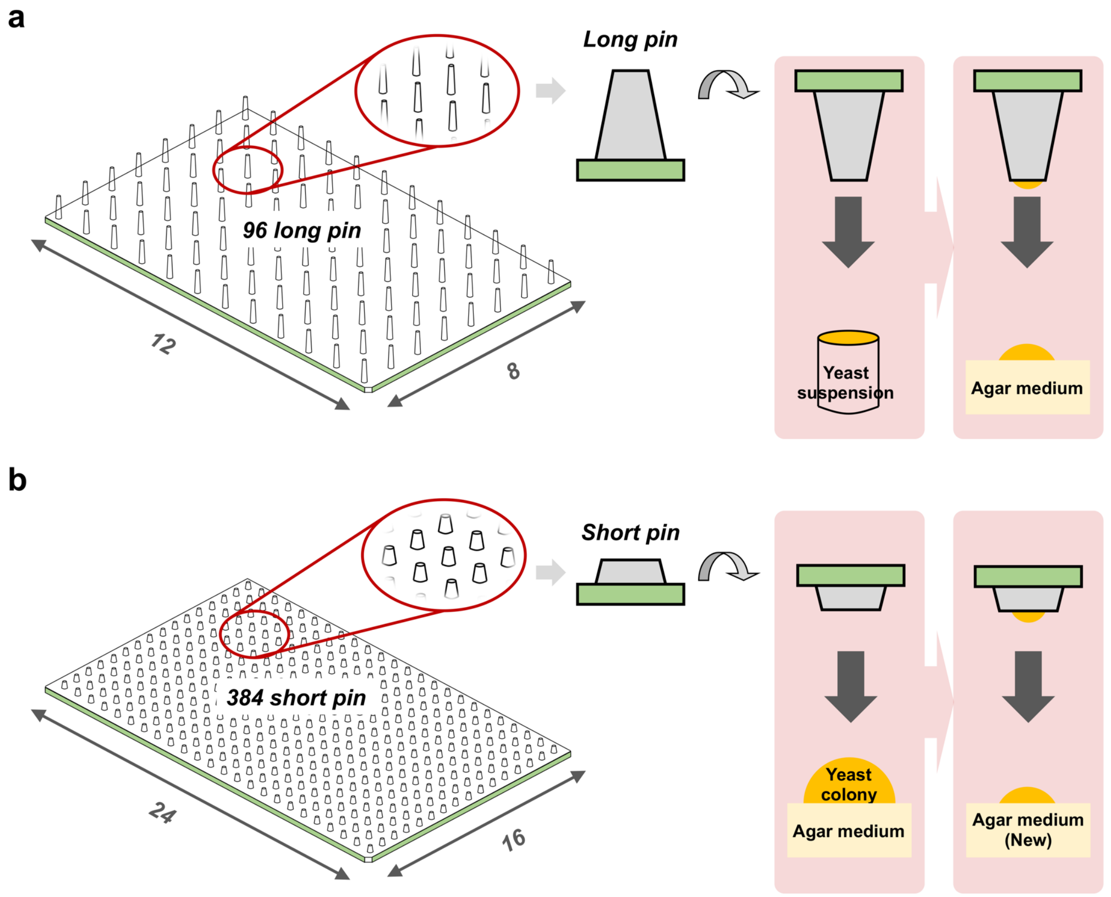

Figure S2. Robotic pinning system. a The 96 long pin is a high-density-formatted tool with 12 long pins as the length and 8 long pins as the width, which was used to transfer the liquid yeast cells. These Long pins would dip into the yeast suspension and carry the yeast cells to agar medium, resulting in solid yeast colonies. **b** The 384 short pin is also a high-density-formatted tool with 24 short pins as the length and 16 short pins as the width. Short pin was used to carry the solid yeast colony to pin on new agar medium.

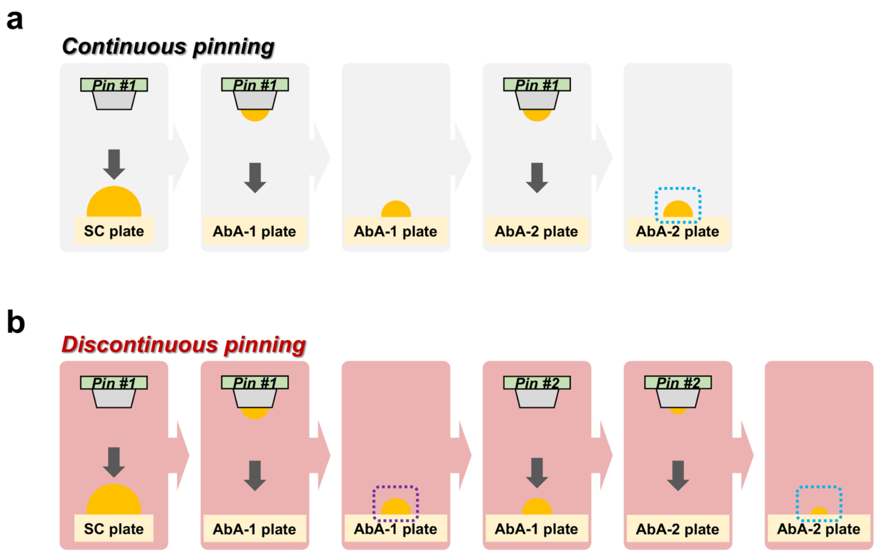

Figure S3. Continuous and discontinuous pinning. Two pinning processes were tested for yeast colony transferring. **a** One is the continuous pinning. During the continuous pinning, the colonies on the SC plates would be transferred to the AbA-1 plates through the pin #1. The pin #1 would continuously pin on the AbA-2 plates without replacing this pin #1. **b** The other pinning process is the discontinuous pinning. After the colonies was transferred from the SC plates onto the AbA-1 plates by the pin #1, the pin #1 would be discarded. A new pin (pin #2) was then used to transfer the colonies from the AbA-1 plates to the AbA-2 plates. The colonies on the AbA-2 plate (blue dashed frame in (**b**)) would be much smaller than that on the AbA-1 plates (purple dashed frame). The difference between two pinning processes is the transferred yeast colony amount on the AbA-2 plates. The colony size on the AbA-2 plate of (**a**) continuous pinning is larger that of (**b**) discontinuous pinning.

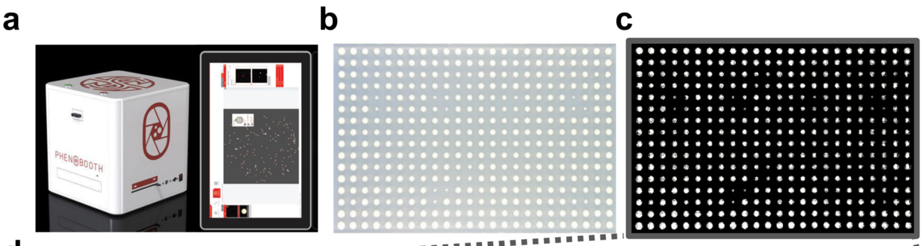

d

	01	02	03	04	05	06	07	08	09	10	11	12	13	14	15	16	17	18	19	20	21	22	23	24
A	248	221	224	218	242	248	165	171	218	225	236	238	205	208	214	230	225	216	137	113	126	133	170	208
B	198	162	147	150	183	191	110	103	145	157	183	174	159	151	164	147	187	168	099	071	091	099	118	157
C	101	092	076	069	082	091	123	121	097	113	131	115	070	074	102	080	078	095	083	066	046	047	061	089
D	170	091	094	069	078	078	113	105	097	115	123	106	065	061	091	070	057	048	066	041	051	037	051	081
E	166	120	080	063	096	124	107	097	079	076	120	128	122	120	077	083	088	084	065	052	067	078	087	103
F	170	125	067	072	093	093	103	091	061	062	144	118	115	121	077	083	075	087	051	049	072	065	077	090
G	147	115	132	109	106	123	150	144	082	080	128	098	118	134	133	117	085	113	098	077	085	088	072	101
H	143	122	132	129	105	132	148	139	090	101	124	095	118	121	122	121	090	077	092	084	082	098	072	82
I	155	133	108	118	128	154	090	073	092	109	142	146	108	126	118	099	096	090	069	094	048	045	104	135
J	193	162	128	127	156	163	079	080	079	092	174	129	123	111	131	089	113	098	067	094	056	054	109	146
K	105	068	072	071	068	064	109	083	061	077	094	089	050	049	078	044	048	052	101	095	046	065	076	074
L	108	066	084	064	063	083	104	102	073	074	089	095	049	050	069	071	058	058	111	120	047	049	072	098
M	190	142	114	092	125	122	111	120	126	125	140	131	123	126	066	077	088	079	105	103	028	027	095	125
N	200	153	123	100	117	125	101	107	145	131	133	137	125	122	065	082	075	084	112	115	038	019	101	111
O	214	156	155	158	160	188	199	180	127	105	159	158	157	141	162	151	111	118	138	111	094	093	129	155
P	287	202	211	208	200	231	229	208	127	174	196	190	221	196	210	205	181	184	186	179	169	174	171	191

Figure S4. Quantification of yeast colony sizes. a The PhenoBooth Colony Counter (Singer) was used to capture the images of the yeast colonies on the plates (**b**), which were converted into black-and-white images (**c**). The sizes of each colony on the HDF plates were then quantified into the corresponding values (**d**).

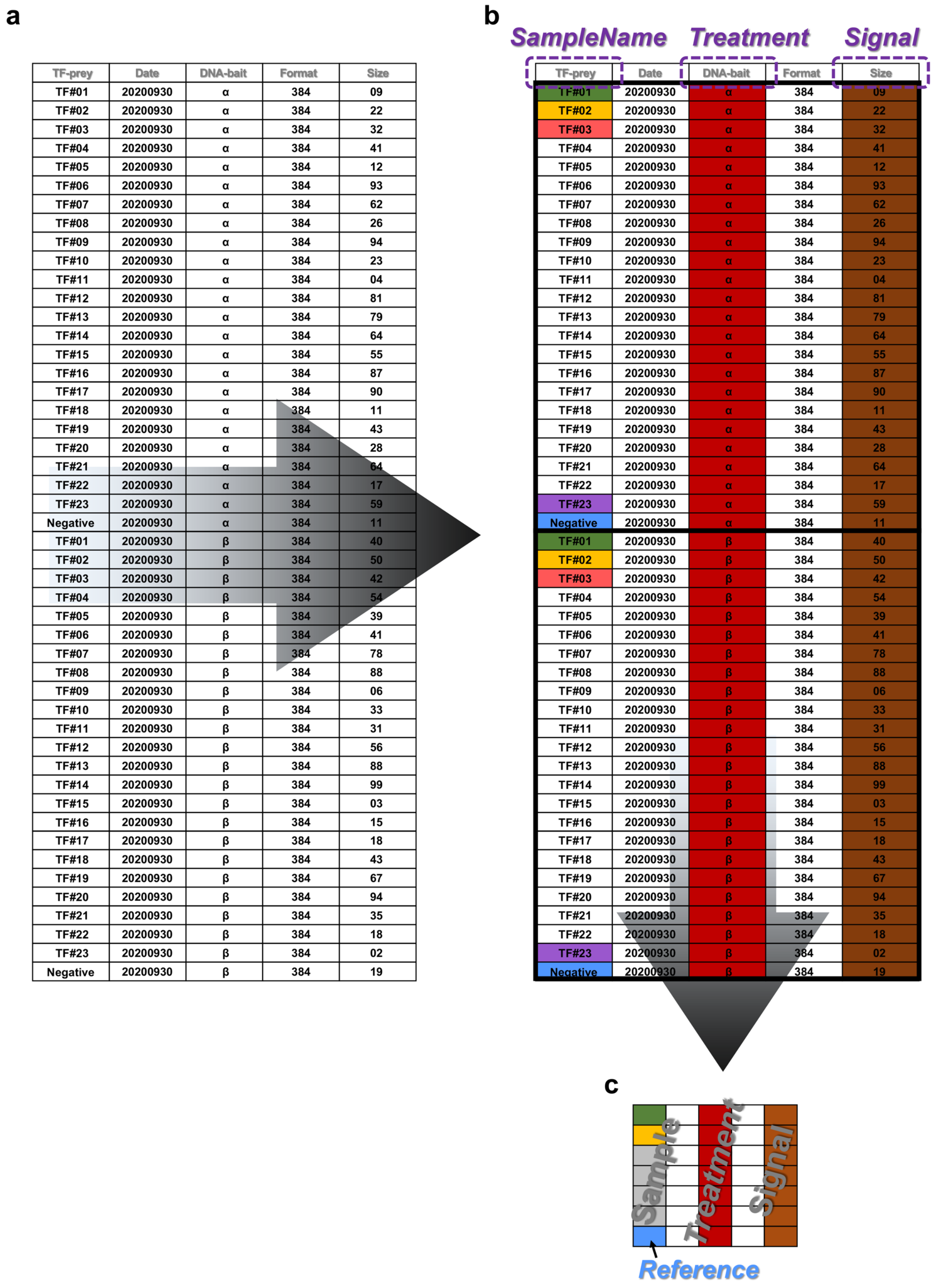

Figure S5. Identification of information from input file. a An input file example as a table with columns and rows. The information in the input file includes TF-prey, date, DNA-bait, format and size. **b** The required data can be selected and identified through the column titles of the input file in

GUI. The TF-prey column was selected as “SampleName”. The DNA-bait column was selected as “Treatment”. The size column was selected as “Signal”. **c** The identified columns would be extracted for further processing.

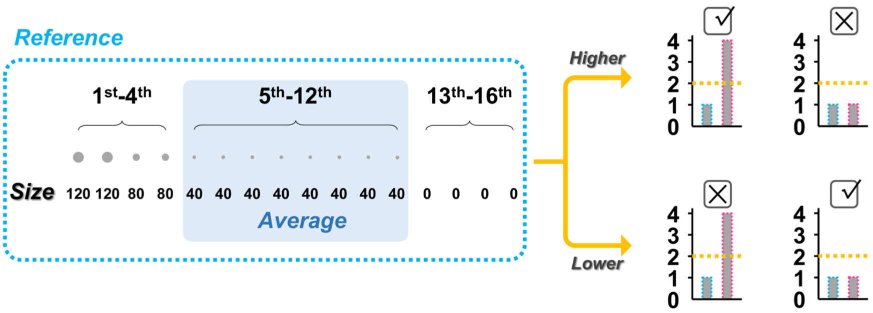

Figure S6. Selection of higher or lower reference cutoff. In reference cutoff, four kinds of selecting options are available: higher, higher or equal, lower, and lower or equal. When the higher option is used, a colony from the experimental samples (pink dashed frame) with higher values than cutoff (orange dashed lines) would be regarded as a positive. On the opposite, if the lower option is selected, a colony with higher size value would be regarded as a negative.

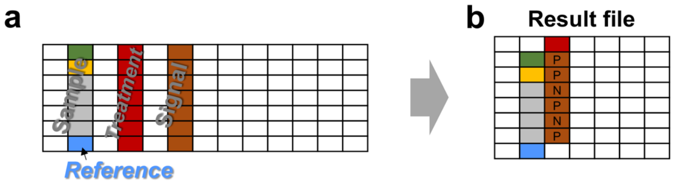

Figure S7. Result file format. a The identified and extracted input data is shown as a sample name column (with green, yellow, grey and blue cells), a treatment column (in red) and a signal column (in brown). **b** After GM analysis, the results would be integrated and output as a result file. Each signal would be processed into a positive or negative result (P/N in brown) in the result column, and the treatment would be the title of the result column. The sample names were listed beside the P/N results. The reference (blue) would not obtain a P/N result.

Single Dose

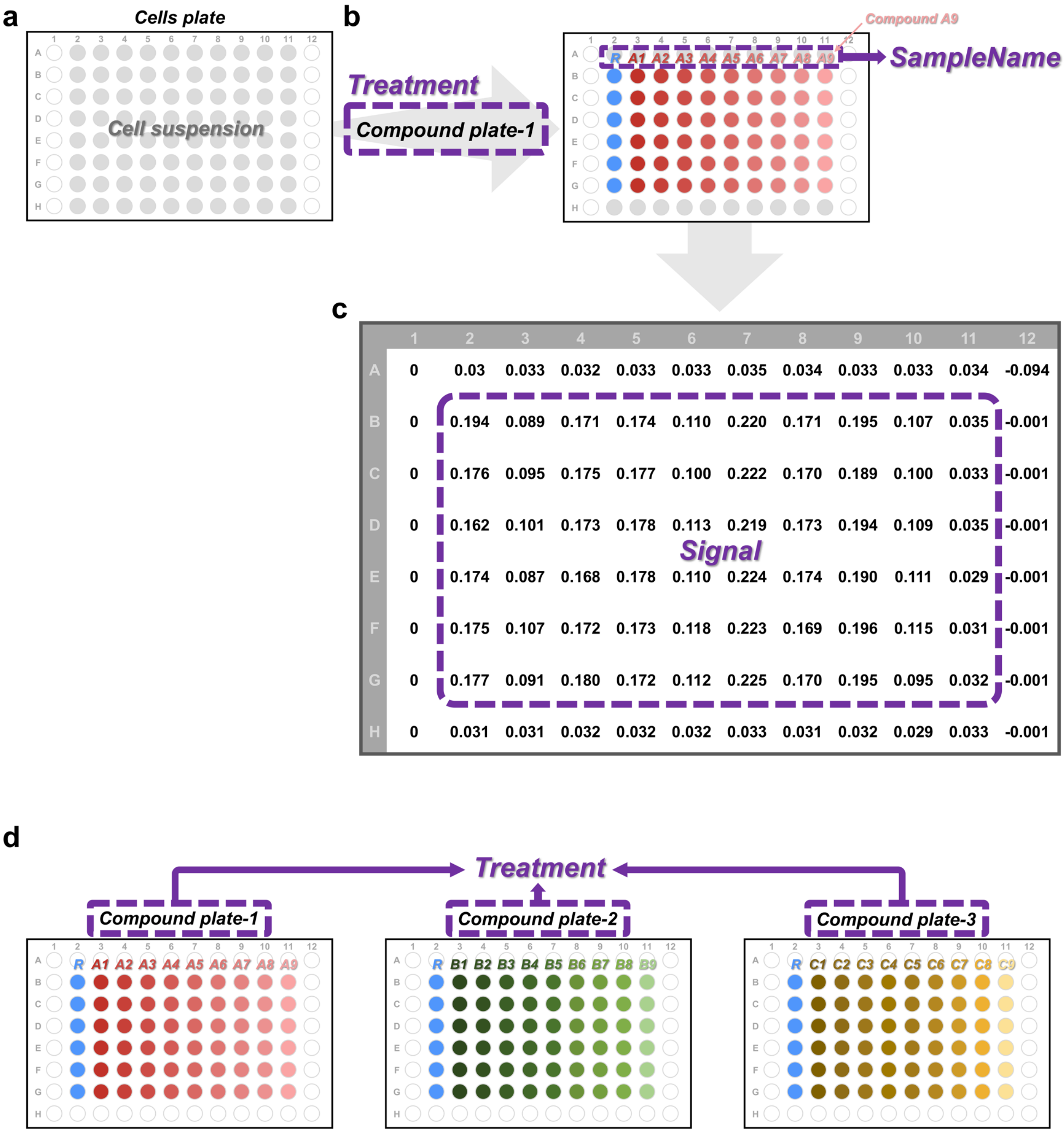

Figure S8. SampleName, Treatment and Signal of single-dose screening in drug development. a A schematic 96-well plate seeded with cell suspension (grey wells) was defined as a “cells plate”. **b** The cells were incubated with various reagents in a compound plate (compound plate-1), including 1 reference (“R” represented the solvent control and were shown in blue) and 9 different compounds (compound A1 to A9). The different reagents were defined as “SampleName”. The compound plate-1 was defined as “Treatment”. The relative viability of cells in each well would further be detected and

quantified into corresponding values (**c**), and these values circled by purple dashed frame were defined as “Signal”. **d** In single-dose screening, the numbers of compound plates could be multiple, such as compound plate-1 (compound A1 to A9, red to pink), compound plate-2 (compound B1 to B9, dark green to light green), and compound plate-3 (compound C1 to C9, brown to light yellow). The compound plate-1, compound plate-2 and compound plate-3 were defined as “Treatment”.

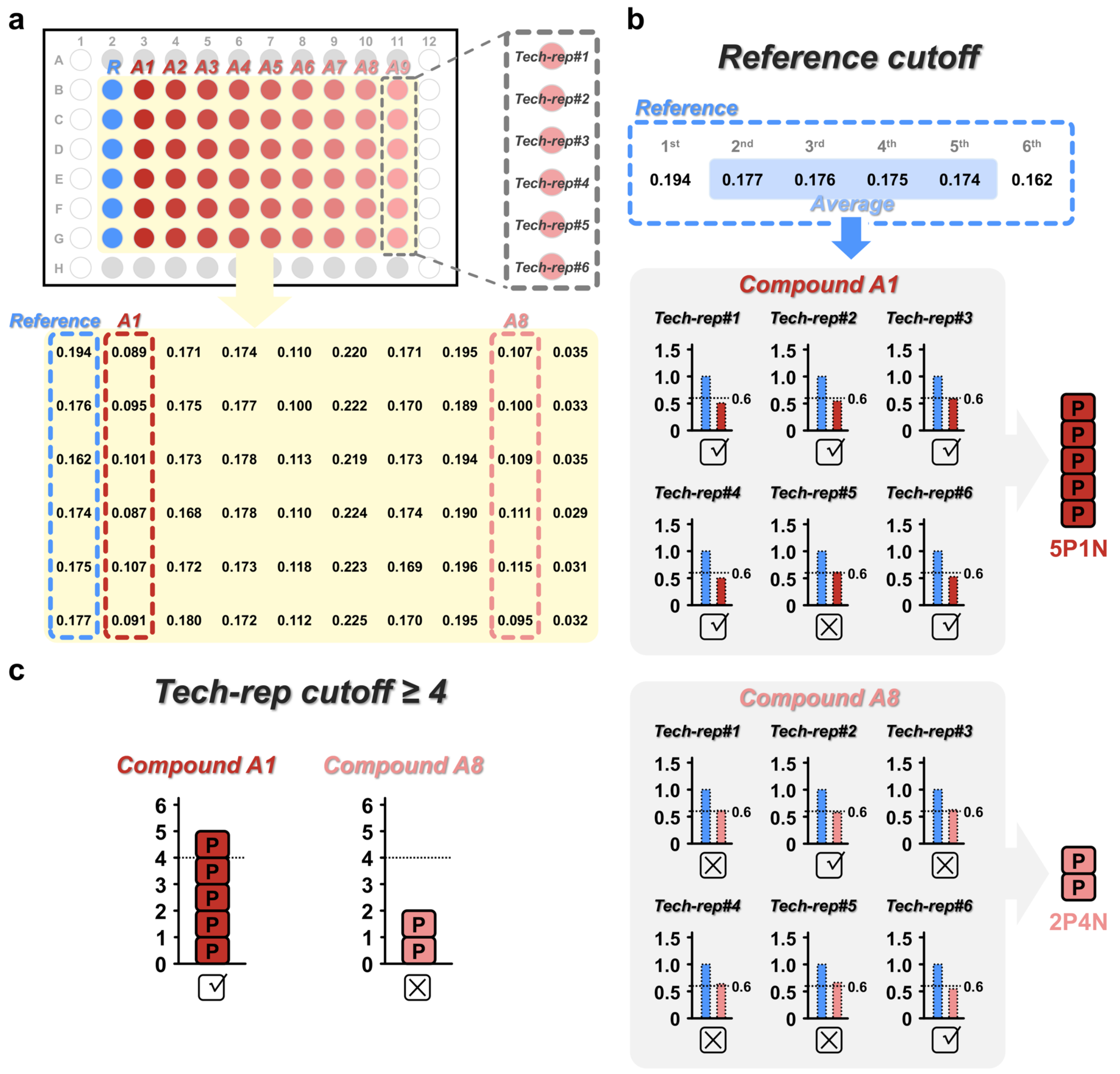

Figure S9. Cutoff setting of single-dose screening in drug development. a In single-dose screening, the reference or each compound were conducted with 6 technical replicates (tech-rep#1 to tech-rep#6, circled in the grey dashed frame). The schematic values from relative cell viability were highlighted in yellow background. The values circled by a blue frame were from the reference. Two compounds, compound A1 (in red dashed frame) and compound A8 (in pink dashed frame), were used as the examples to demonstrate the following cutoff setting. **b** In reference cutoff, the 6 reference values (in blue dashed frame) were ranked from the highest to the lowest. The reference values ranged from 2nd to 5th (in blue background) were averaged. Such averaged value of reference was normalized to 1, and the fold change cutoff was set as 0.6-fold lower than the reference. If the relative fold change value

from one technical replicate of a compound is lower than the cutoff value 0.6, then this technical replicate would be regarded as a positive. If not, then this technical replicate would be regarded as a negative. The compound A1 (in red) showed 5 positives and 1 negative (5P1N), while the compound A8 (in pink) exhibited 2 positives and 4 negatives (2P4N). **c** The tech-rep cutoff was set as higher than or equal to 4. In this case, the compound A1 included 5 positives (P, positive), which was greater than the tech-rep cutoff and would be regarded as a positive.

Serial Dose

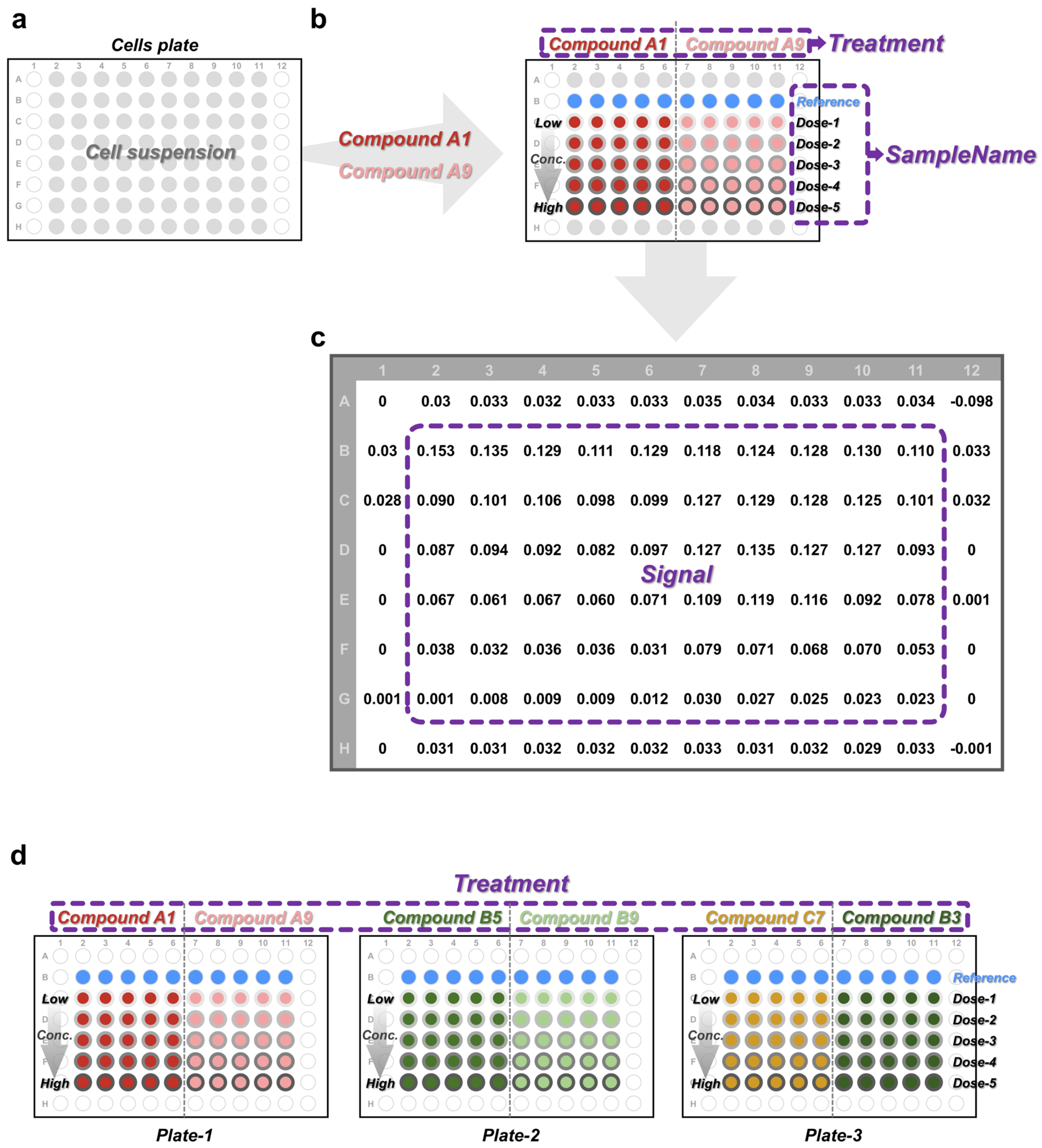

Figure S10. SampleName, Treatment and Signal of serial-dose screening in drug development. a The cells were seeded in a 96-well plate, (**b**) and then were incubated with the reference (R, solvent control, blue) and two compounds (compound A1, red; compound A9, pink). The compound-treated experiments were performed in serial dosages from low (Dose-1) to high (Dose-5). The compound dosages and the reference were defined as "SampleName". The names of compounds, such as

compound A1 and compound A9, were defined as “Treatment”. The relative viability of cells in each well would further be detected into schematic corresponding values (**c**), and these values circled by purple dashed frame were defined as “Signal”. **d** The numbers of compounds increased with the plate numbers (plate-1 to plate-3), and the names of compounds were defined as “Treatment” (compound A1, compound A9, compound B5, compound B9, compound C7, compound B3).

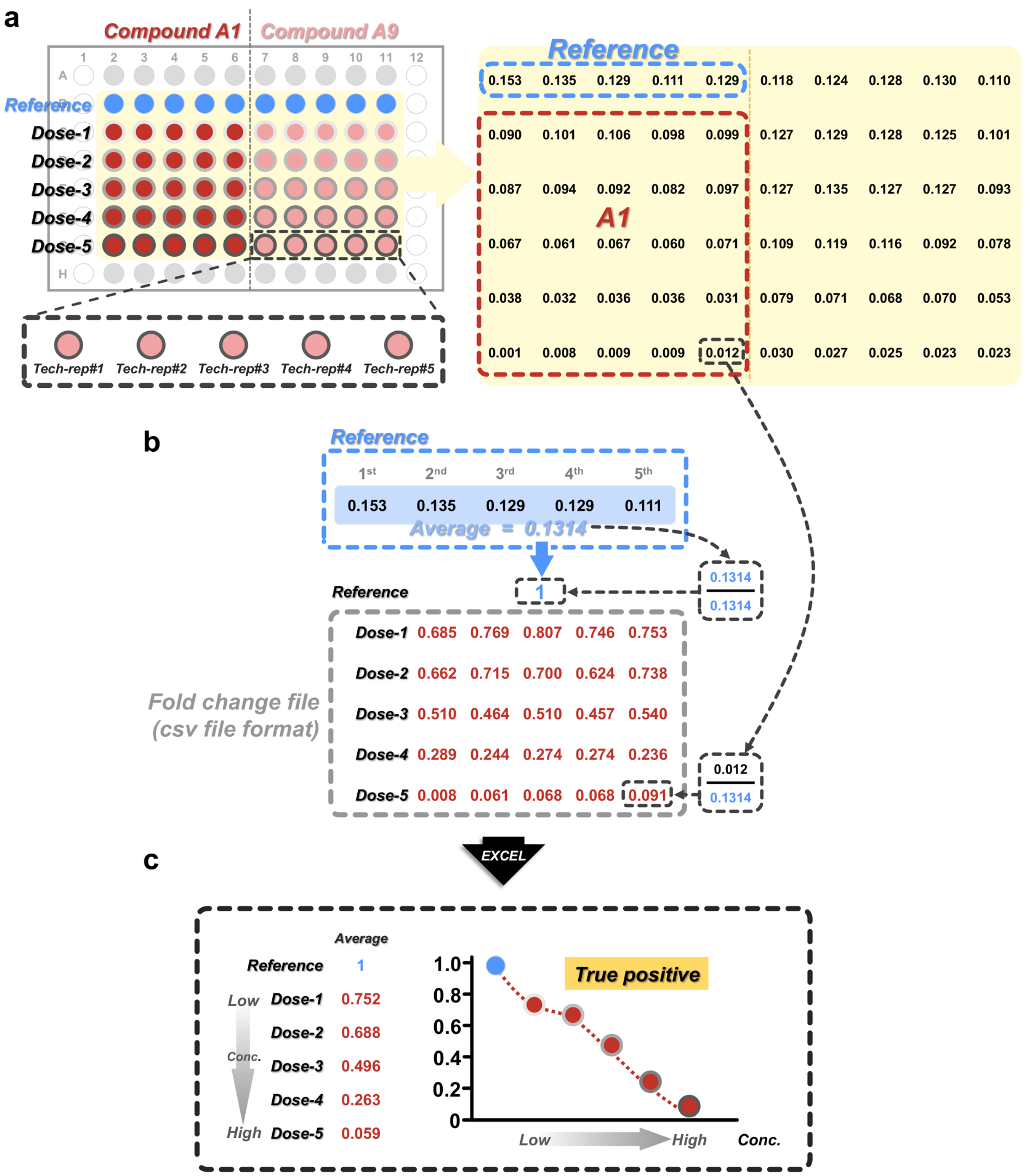

Figure S11. Data processing of serial-dose screening in drug development. a In serial-dose screening, each dosage of a compound or reference was conducted by 5 technical replicates (tech-rep#1 to tech-rep#5 in black dashed frame). The schematic values of relative cell viability were highlighted in yellow background. The values in blue dashed frame represented the reference, and the values in red dashed frame were the treatment results of compound A1. **b** The 5 technical replicates of reference values were averaged and normalized to the 1 (0.1314/0.1314). The normalized values of each compound dosages were compared to the value of averaged reference to generate a fold change

file (circled by grey dashed frame). The value of tech-rep#5 in the highest concentration of compound A1 was illustrated as an example (the value was circled in black dashed frame on the yellow background). Such value was 0.012, which was divided by the averaged reference value 0.1314, resulting in 0.091 (0.012/0.1314) in the fold change file. The fold change file was in csv file format which could be easily further processed by (**c**) Excel to generate the dose-response curve based on the user requirements.

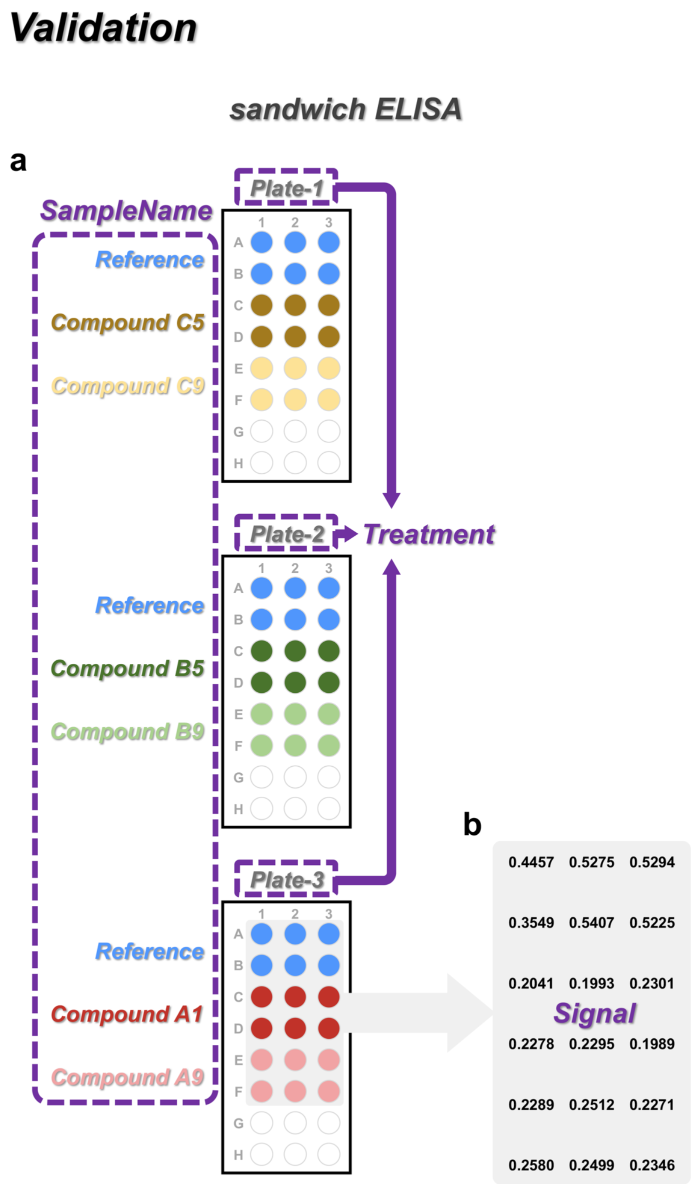

Figure S12. SampleName, Treatment and Signal of target validation in drug development. a The compound candidates were validated by sandwich ELISA. Each plate was composed of 1 reference group (in blue) and 2 compound groups. The reference (blue) and different names of compounds (brown, yellow, dark green, light green, red, and pink) were defined as "SampleName". The different plates, including plate-1, plate-2 and plate-3, were defined as "Treatment". **b** The corresponding schematic values in the reference group and compound groups were defines as "Signal" (in grey background).

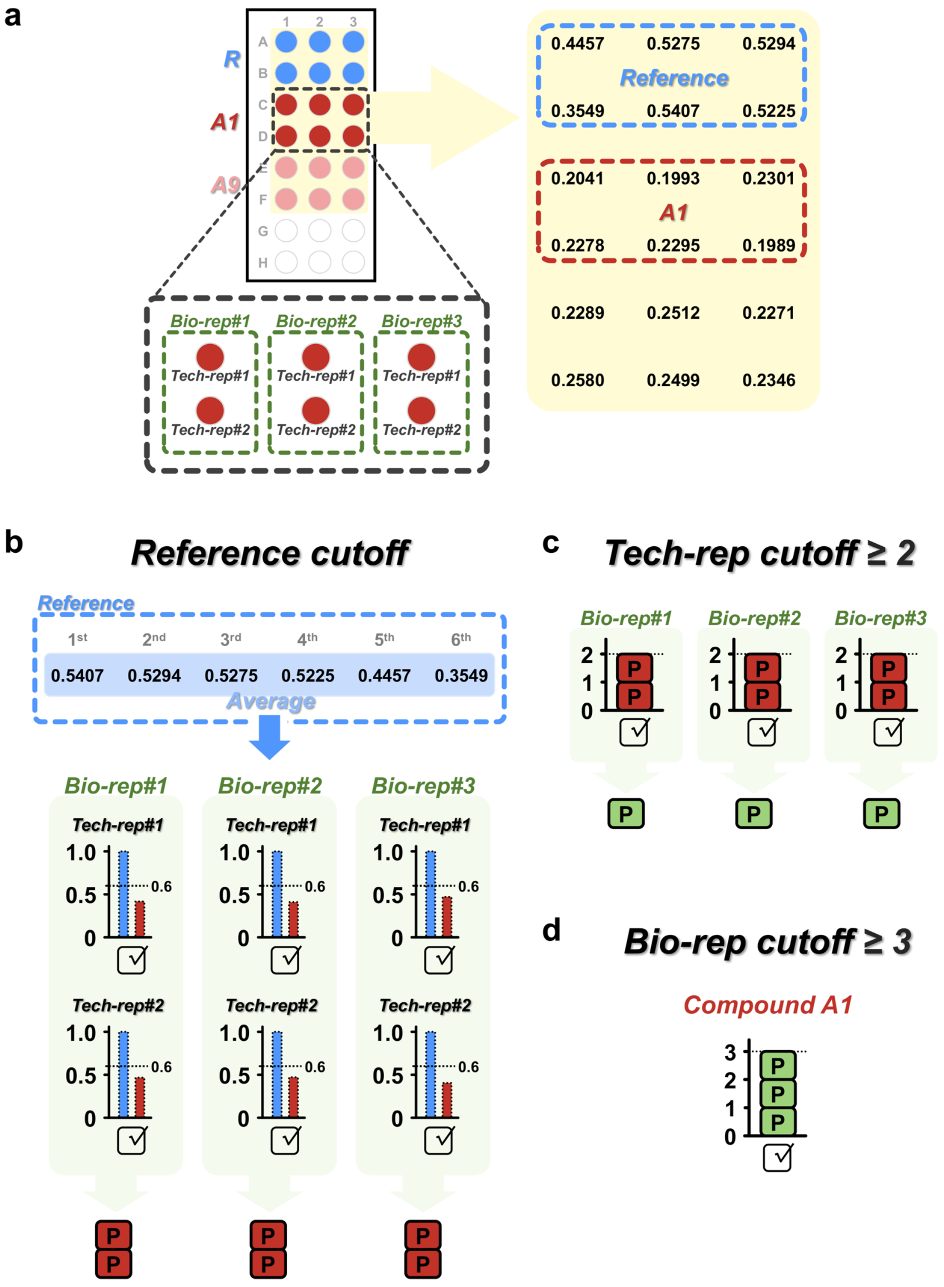

Figure S13. Cutoff setting of target validation in lead compound identification. a The compound-treated group or reference group each was composed of 3 biological replicates (bio-rep#1 to bio-rep#3 in black dashed frame), and each biological replicate included 2 technical replicates (tech-rep#1 and tech-rep#2 in green dashed frame). The corresponding schematic values of reference group and compound-treated group were shown within the yellow background. The values in blue dashed frame represented the reference, and the values in red dashed frame belonged to compound A1. **b** In reference cutoff, the values of 6 references (3 biological replicates × 2 technical replicates) were

averaged. The averaged value was normalized to 1, and the fold change cutoff was set as 0.6-fold lower than the reference. If the relative value of compound-treated group is lower than the cutoff value 0.6, then the compound-treated group would be regarded as a positive. In the case of compound A1, two technical replicates in each biological replicates (bio-rep#1, bio-rep#2 and bio-rep#3) were all regarded as positives (P, positive). **c** The tech-rep cutoff was set as higher than or equal to 2. For compound A1, each biological replicate showed 2 positive technical replicates, and then each biological replicate was regarded as a positive (P, positive). **d** The bio-rep cutoff was set as higher than or equal to 3. The compound A1 with 3 positive biological replicates passed the bio-rep cutoff.

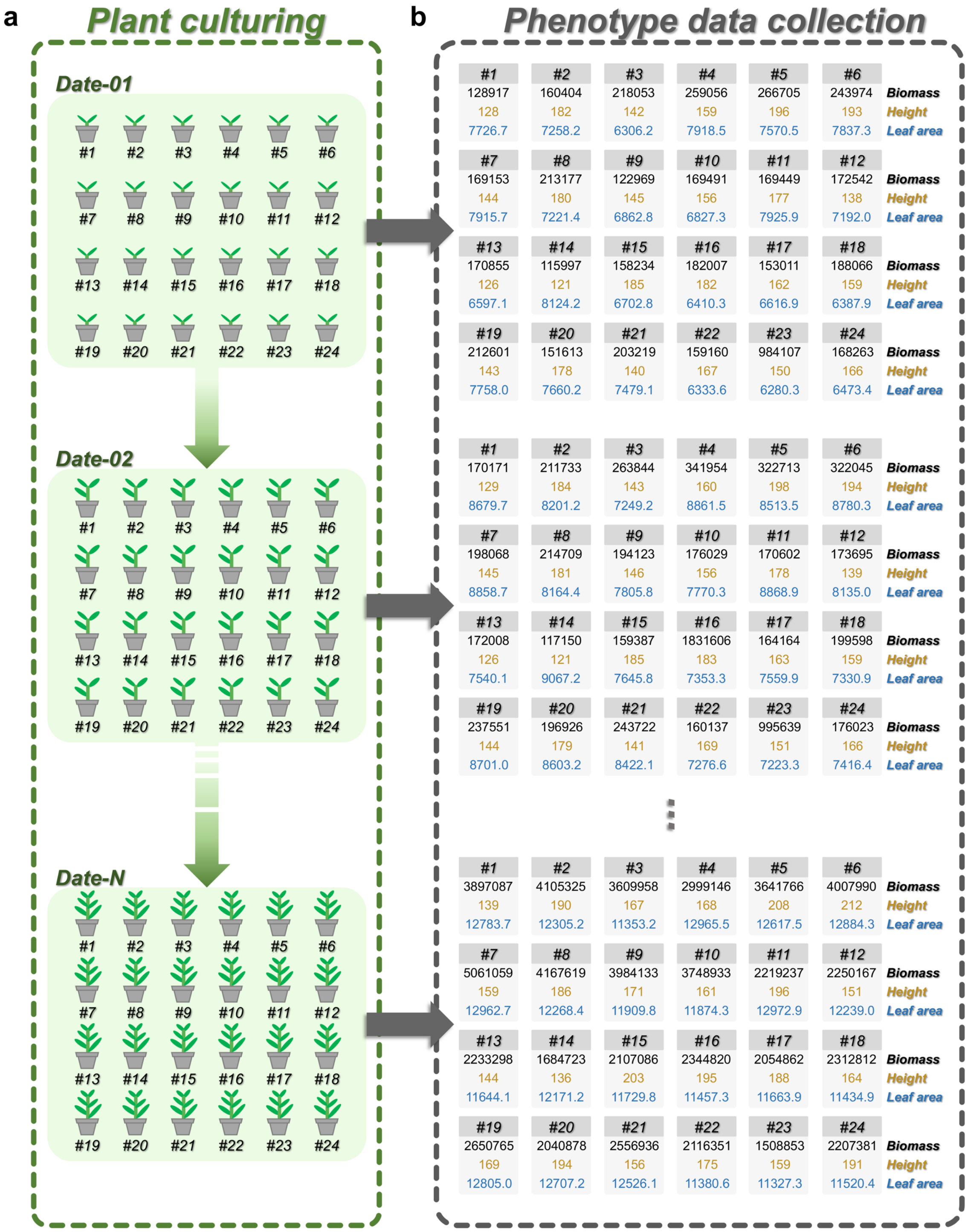

Figure S14. Data collection of precision agriculture. a A schematic group of plant lines was comprised of 24 plant lines (from #1 to #24). **b** During plant growth, the different phenotypic data of each plant line was collected on each day, including biomass (black), height (brown) and leaf area (blue).

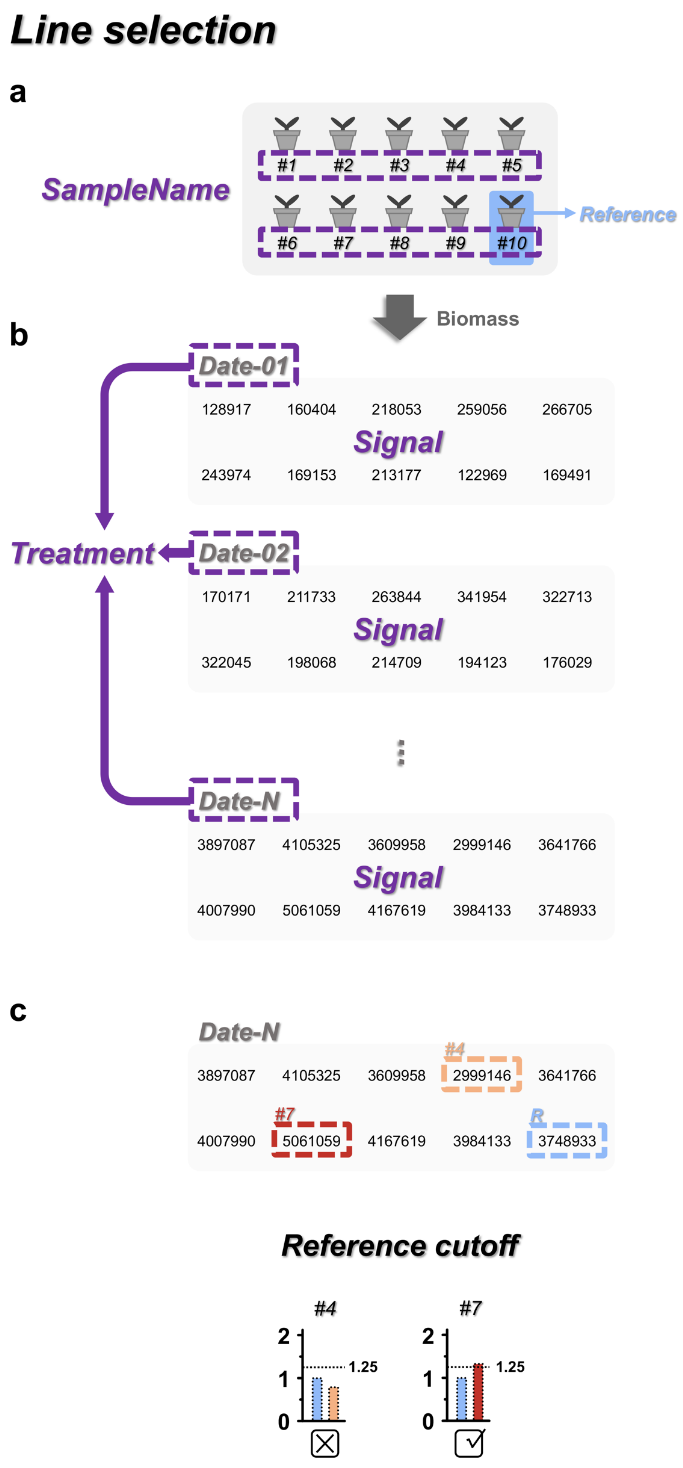

Figure S15. Line selection of precision agriculture. **a** A schematic group of plant lines, including 9 experimental lines (#1 to #9) and 1 reference line (#10), was used to demonstrate the line selection. The names of plant lines were defined as "SampleName". **b** The biomass data of plant lines were collected on each day. The dates (from Date-01 to Date-N) of biomass data were defined as "Treatment". The biomass values of plant lines were defined as "Signal". **c** In reference cutoff, the

biomass values of two plant lines (#4 in orange and #7 in red) in Date-N were used as examples. The biomass value of the reference line (R, blue) was normalized as 1 (blue bars), and the fold change cutoff was set as 1.25-fold higher than the reference. The relative biomass fold-change value of line#4 was lower than the cutoff value 1.25, and then defined as a negative. The relative biomass fold-change value of line#7 was higher than the cutoff value 1.25, and then defined as a positive.

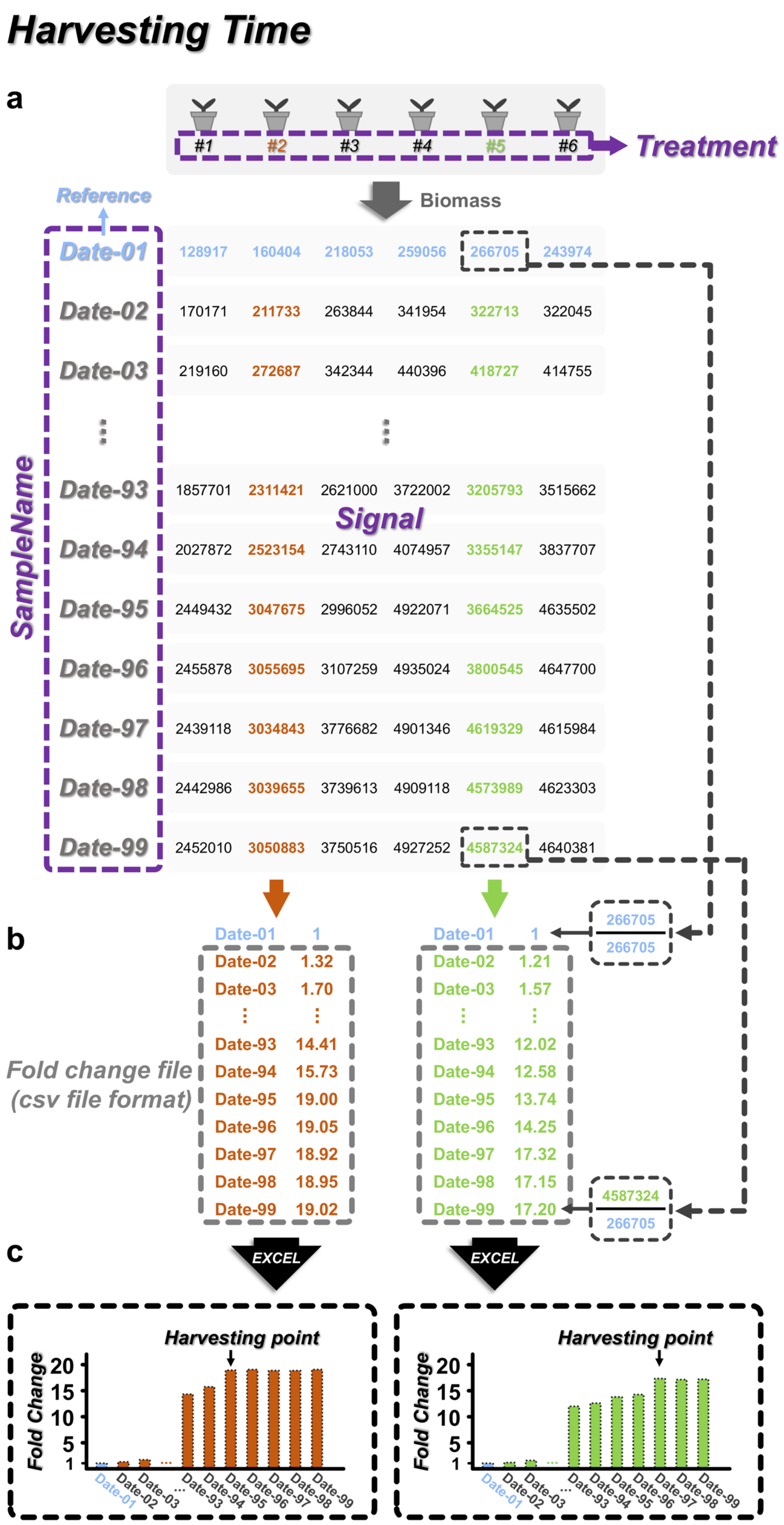

Figure S16. Harvesting time of precision agriculture. a A schematic group of 6 plant lines (#1 to #6) were used to collect the biomass data from Date-01 to Date-99. To decide the harvesting time, the dates of biomass values were defined as "SampleName". The names of plant lines were defined as

"Treatment". The biomass values were defined as "Signal". The biomass values from Date-01 in each plant lines were selected as the reference (blue). **b** The plant line#2 (brown) and line#5 (green) were used as examples. For plant line#5, the biomass values of Date-01 were normalized to 1 (blue, calculated from 266705/266705), and the relative values of biomass in other dates were calculated and outputted into the fold change file in the csv file format (grey frame). **c** The output results in fold change file could easily be further processed into figures by Excel based on user requirements.

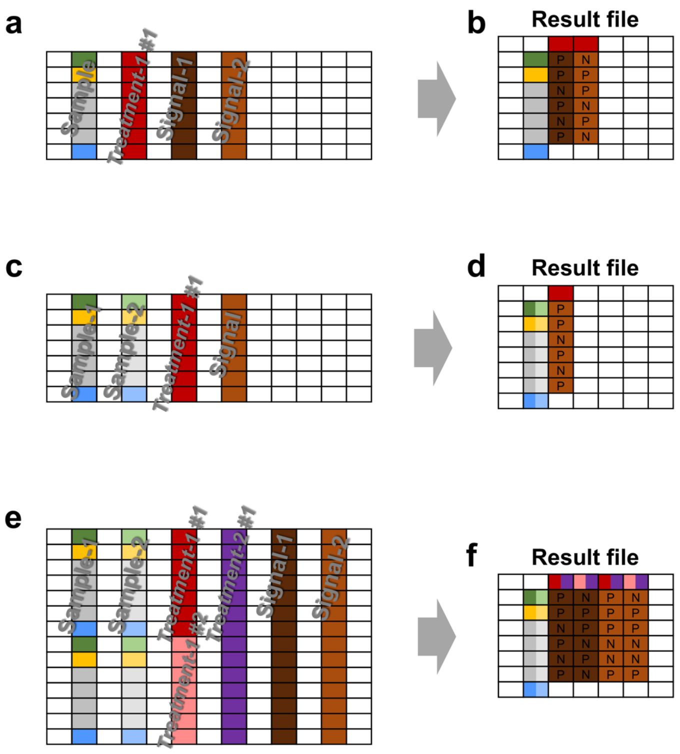

Figure S17. Input and output file format. a The input file includes multiple signal columns, for example, two signal columns (brown and light brown) are shown in this figure. **b** The output result file would contain two P/N result columns with their corresponding treatment as the column title. **c** The input file can also contain multiple sample name columns. The sample names from different sample name columns would be combined into one integrated sample name in (**d**) the output result file. **e** The most complicated situation is an input file containing multiple sample name columns, multiple treatment columns and multiple signal columns. **f** In the output result file, different sample name columns were combined into one column. The treatments from different treatment columns were also combined and used as the titles of each P/N result column. The P/N results of signals were listed on the output result file based on different treatment combinations and sample name combinations.

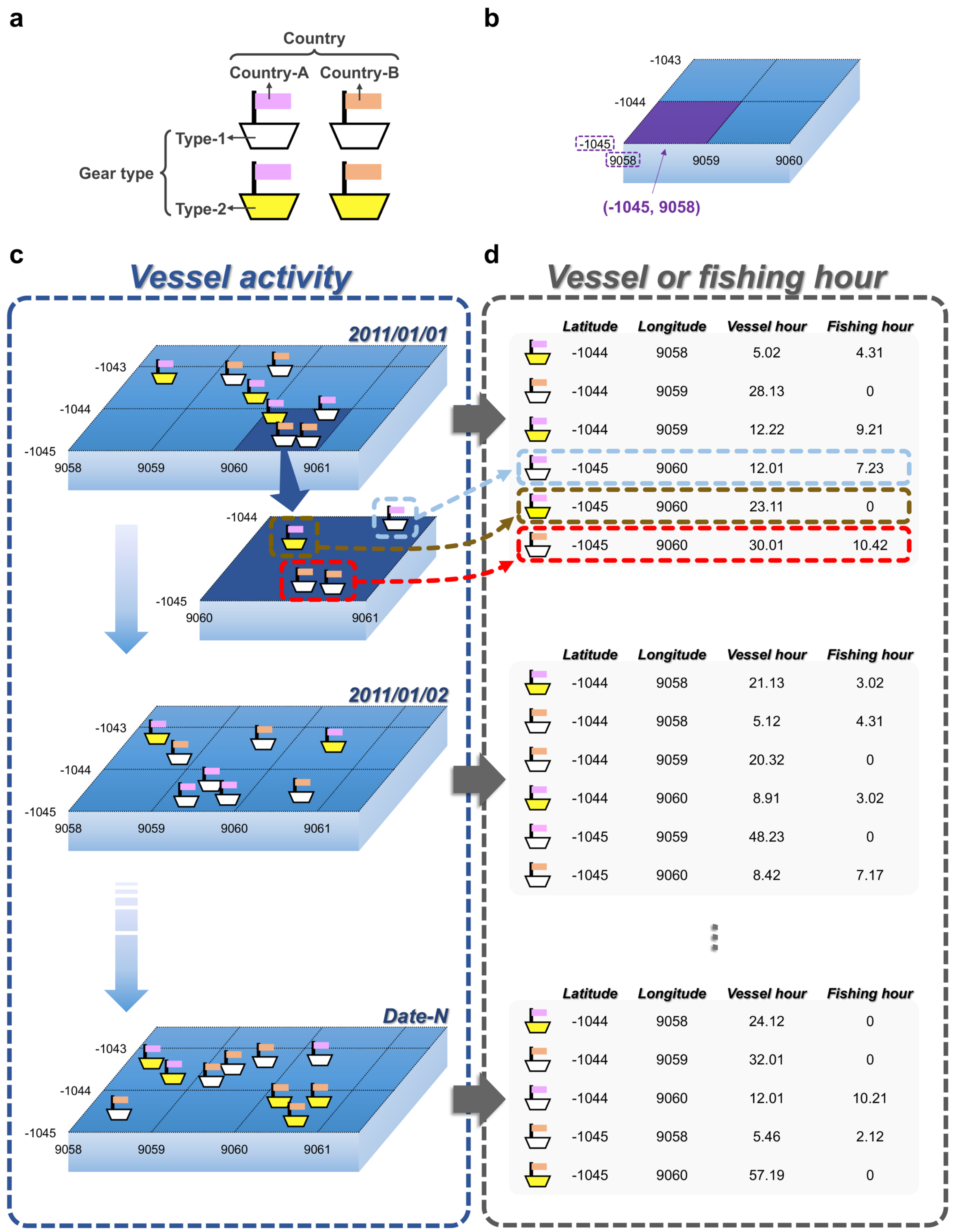

Figure S18. Data collection of marine ecosystems. a Each schematic ship belonged to one country and was equipped with one gear type. Different countries were represented by the color of flags, Country-A with pink flags and Country-B with orange flags. Different gear types were represented by the color of boats, Type-1 in white boats and Type-2 in yellow boats. **b** The location of a grid could

be represented by a set of latitude and longitude. For example, the coordinate of the purple grid could be represented by the latitude coordinate as -1045 and the longitude coordinate as 9058. **c, d** The vessel or fishing hour of vessel activity were collected and recorded on each day. In a grid, take dark blue grid as an example, the vessel activities would be recorded as different data sets if the ships belonged to the different countries or equipped with different gear types. For example, the light blue and dark brown dashed frame represented two ships both belonged to the Country-A (pink flags) but equipped with different gear types, then the vessel activities of these two ships would be recorded as two data sets. If the ships were from the same country and equipped with the same gear type in a grid, the recorded vessel activities would be the sum from the ships. In red dashed frame, the 2 ships on the same grid were both from Country-B and equipped with type-1 gear type, so the vessel activities of both ships would be recorded into one data set.

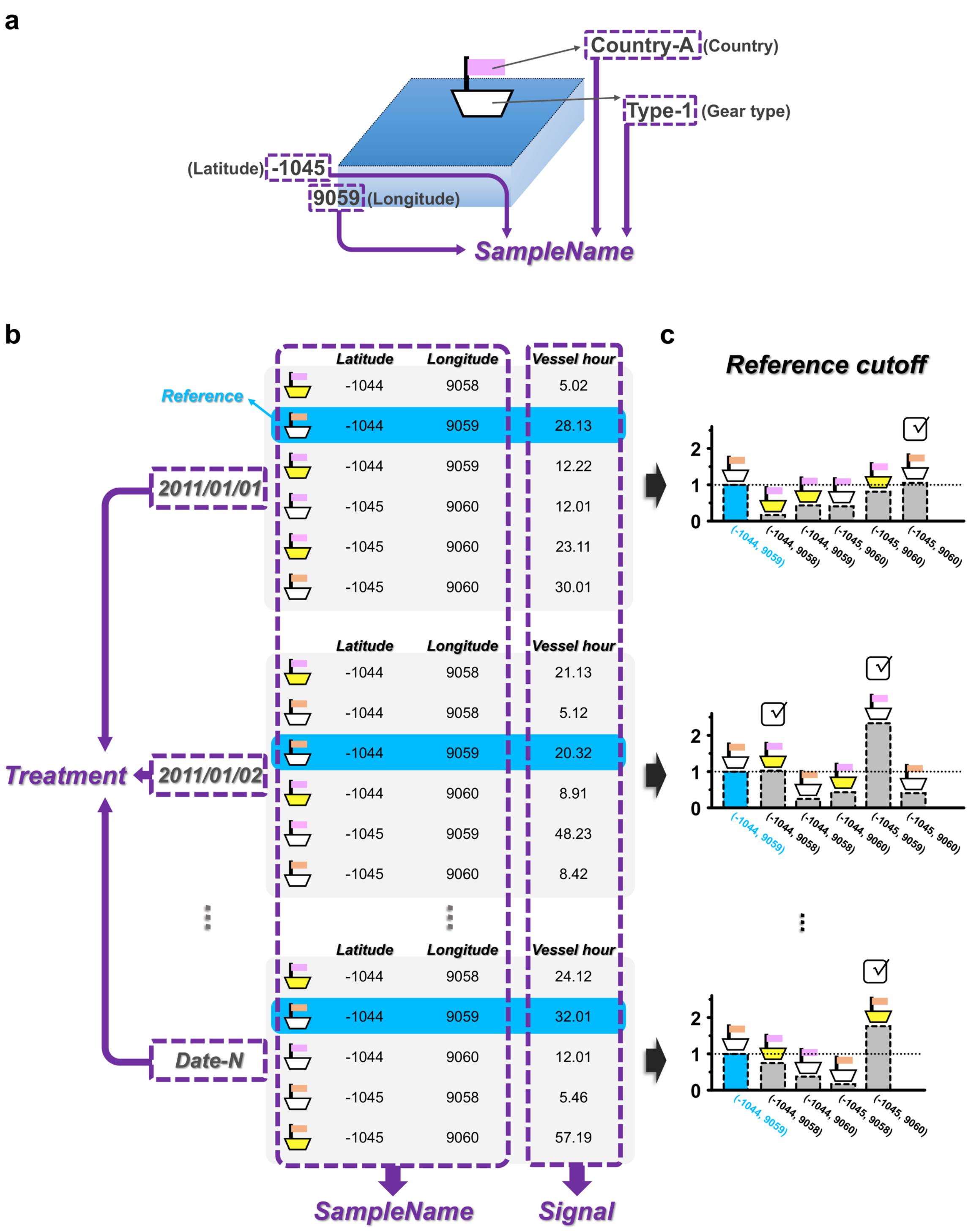

Figure S19. SampleName, Treatment, Signal and cutoff setting of marine ecosystem data. a The "SampleName" is composed of gear type, country, latitude and longitude of marine ecosystem data. **b** The dates of marine ecosystem data were defined as "Treatment". The vessel activity, here as the vessel hour, was defined as "Signal". The SampleName combination of orange flag (Country-B), white boat (gear type-1), -1044 (latitude) and 9059 (longitude) was selected as the reference (in blue

background). **c** In reference cutoff, the vessel hour value of the reference was normalized to 1 and the fold-change cutoff setting was set as 1-fold greater than that the reference. Take the schematic data on 2011/01/01 as an example, the relative vessel hour value of orange flag (Country-B) with white boat (gear type-1) on the grid (-1045, 9060) was higher than the reference cutoff value 1, and then defined as a positive.

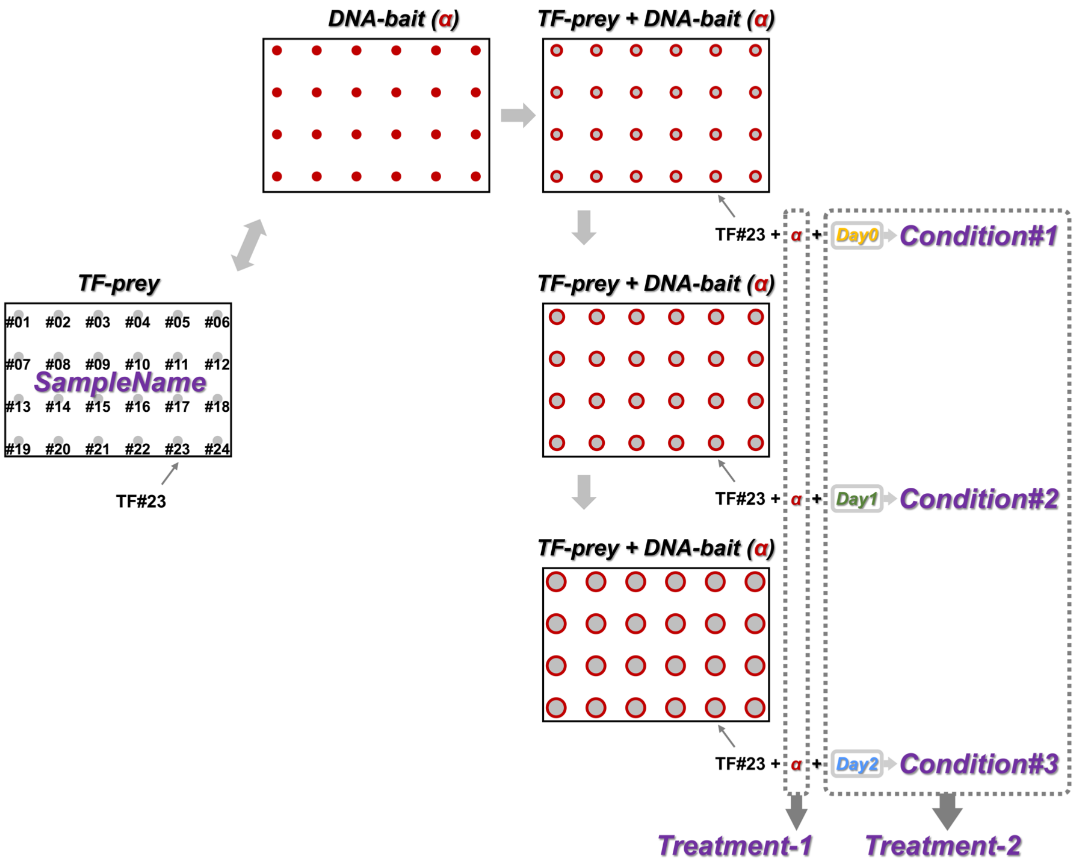

Figure S20. Multiple treatments in Y1H – two treatments. The TF-prey batch can be mated with a DNA-bait(α), resulting in the combination of the TF-prey and DNA-bait(α) in yeast cells. The yeast cells can further be cultured for Day0 (colored in yellow), Day1 (colored in green), and Day2 (colored in blue). Here the DNA-bait and culturing days are defined as different "Treatment". One "Treatment" can contain different conditions. For example, Day0, Day1 and Day2 all belongs to "Treatment-2". Day0 is "Condition#1", Day1 is "Condition#2" and Day2 is "Condition#3".

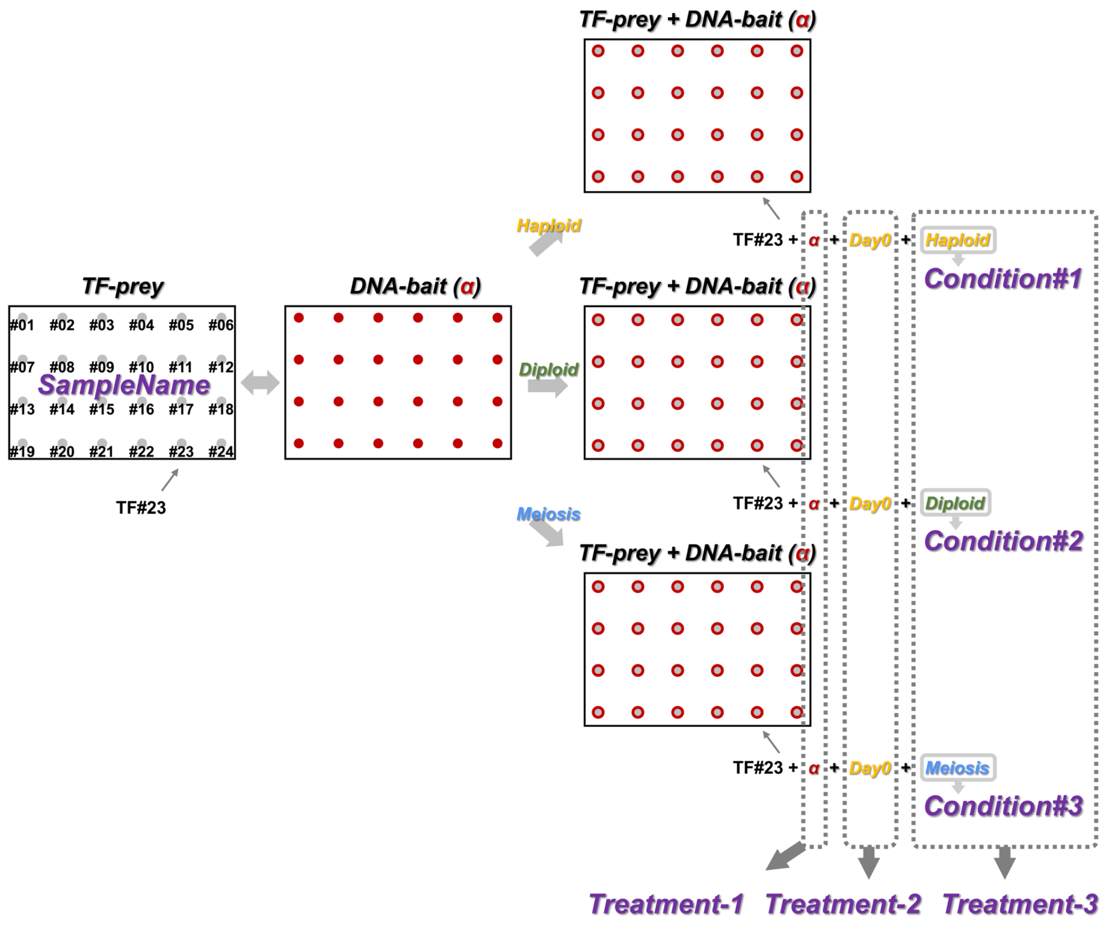

Figure S21. Multiple treatments in Y1H – three treatments. Following the design in Figure S20, the yeast cells can be examined in three different experimental methods, including Haploid (colored in yellow), Diploid (colored in green) and Meiosis (colored in blue). These experimental methods belong to "Treatment-3", and the Haploid, Diploid and Meiosis are "Condition#1", "Condition#2" and "Condition#3", respectively.

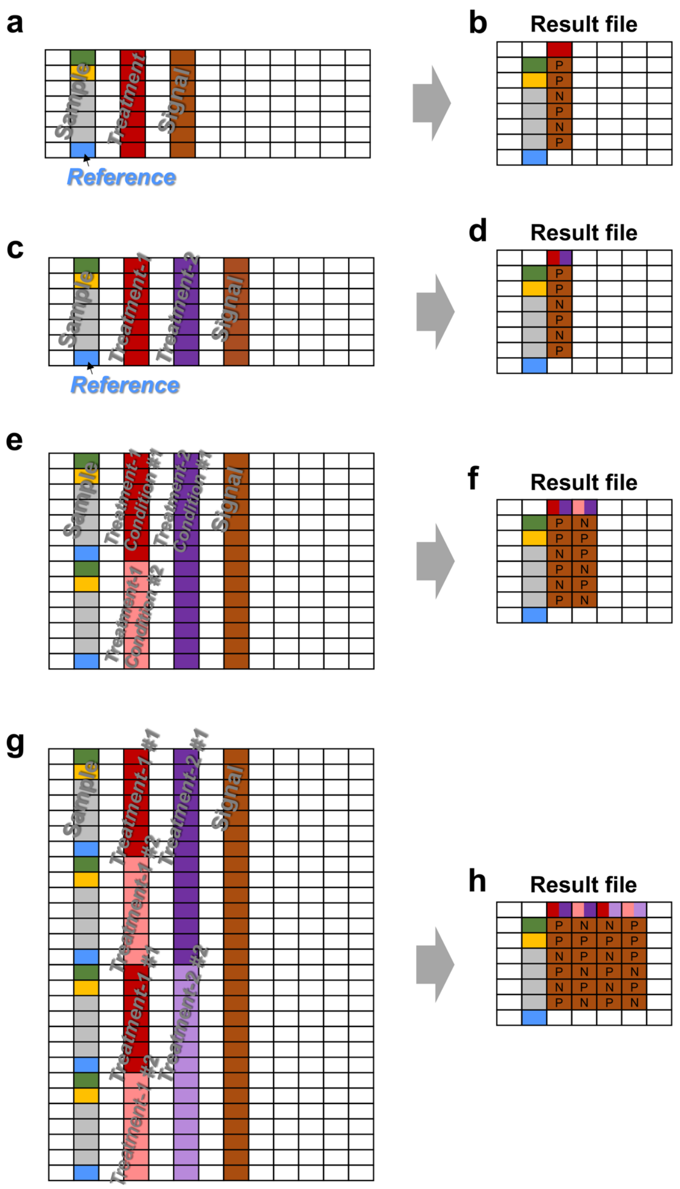

Figure S22. Input and output files format. a The input file included one treatment column (red). **b** The output result file contained one result column (brown) with the P/N results, and the treatment (red) was the title of the result column. **c** The input file included two treatment columns, “Treatment-1” (red) and “Treatment-2” (purple). **d** In the output result file, the treatment would be combined as the title for the result column (half red and half purple). **e** The input file contained two treatment columns, “Treatment-1” and “Treatment-2”. “Treatment-1” contained two conditions, “Condition#1” (red) and “Condition#2” (pink). “Treatment-2” contained one condition, “Condition#1” (purple). **f** In the output result file, the combinations of the conditions from each treatment column would be used as the titles

for the result columns. For example, the “Condition#1” of “Treatment-1” (red) was combined with the “Condition#1” of “Treatment-2” (purple). Such combination represented one of the result column title (half red and half purple) in the result file. **g** The input file, a more complicated situation, contained multiple treatments each with multiple conditions. In the input file, “Treatment-1” (red and pink) and “Treatment-2” (dark purple and light purple) both had two conditions. **h** The conditions from each treatment column would be combined into four combinations as the titles of four result columns.

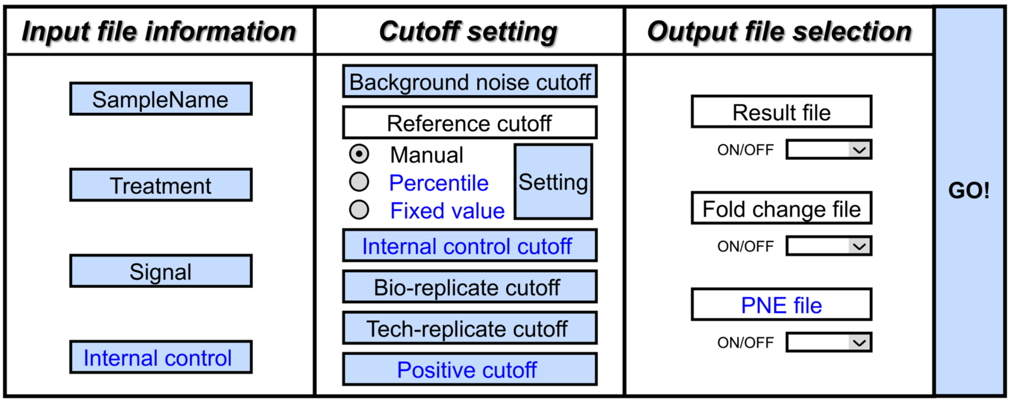

Figure S23. Graphical user interface (GUI) of GM_Advanced. The diagram of the GUI of GM_Advanced (see manual for the exact appearance of GUIs). The GUI contains three main parts, including the input file information, the cutoff setting and the output file selection. The blue words represent additional cutoffs and options compared to the GUI of GM_Basic. The input file information area can be used to identify “SampleName”, “Treatment”, “Signal” and internal control (additional). The cutoff setting area contains background noise cutoff, reference cutoff, internal control cutoff (additional), bio-replicate cutoff, tech-replicate cutoff and positive cutoff (additional). In reference cutoff, besides the manual option, two additional options were added, including the percentile option and the fixed value option. The output file selection area provides the result file, the fold change file and the PNE file (additional). After inputting all required information into GUI, pressing the “GO!” button would start the data analyzing process.

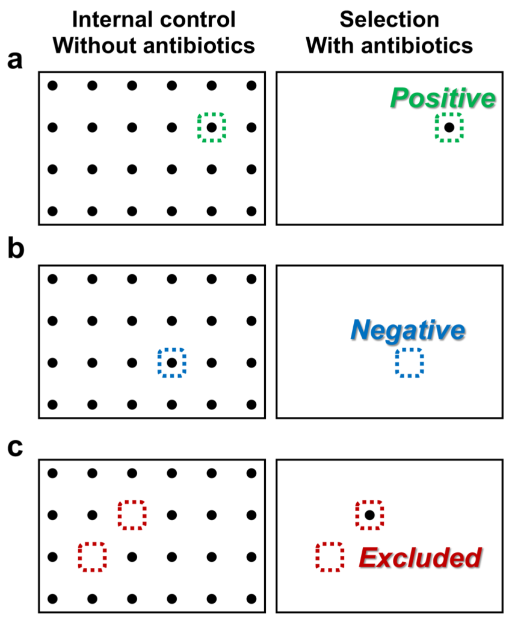

Figure S24. Internal control cutoff. Two kinds of plates were used for culturing yeast colonies, including the non-antibiotic plates and the antibiotic plates. The non-antibiotic plates were used as the internal control plates. All yeast colonies should be able to grow on the internal control plates. The antibiotic plates were used as selection plates. Each plate contains 24 colonies. Grown colonies are shown as black dots and colonies without growing are shown as empties. Colonies on relative positions between the internal control plates and the selection plate contain the same TF-DNA combination. **a**, **b** If a colony can grow on the internal control plates (green dashed frame and blue dashed frame), then (**a**) the growth of the colony on the selection plate would be regarded as a positive (green dashed frame) and (**b**) the colony without growing would be regarded as a negative (blue dashed frame). **c** The colonies without growing on the internal control plate (red frames) would be regarded as experimental errors during sample preparation. Upon this situation, the colonies with or without growing on the selection plate would both be counted as excluded (red frames).

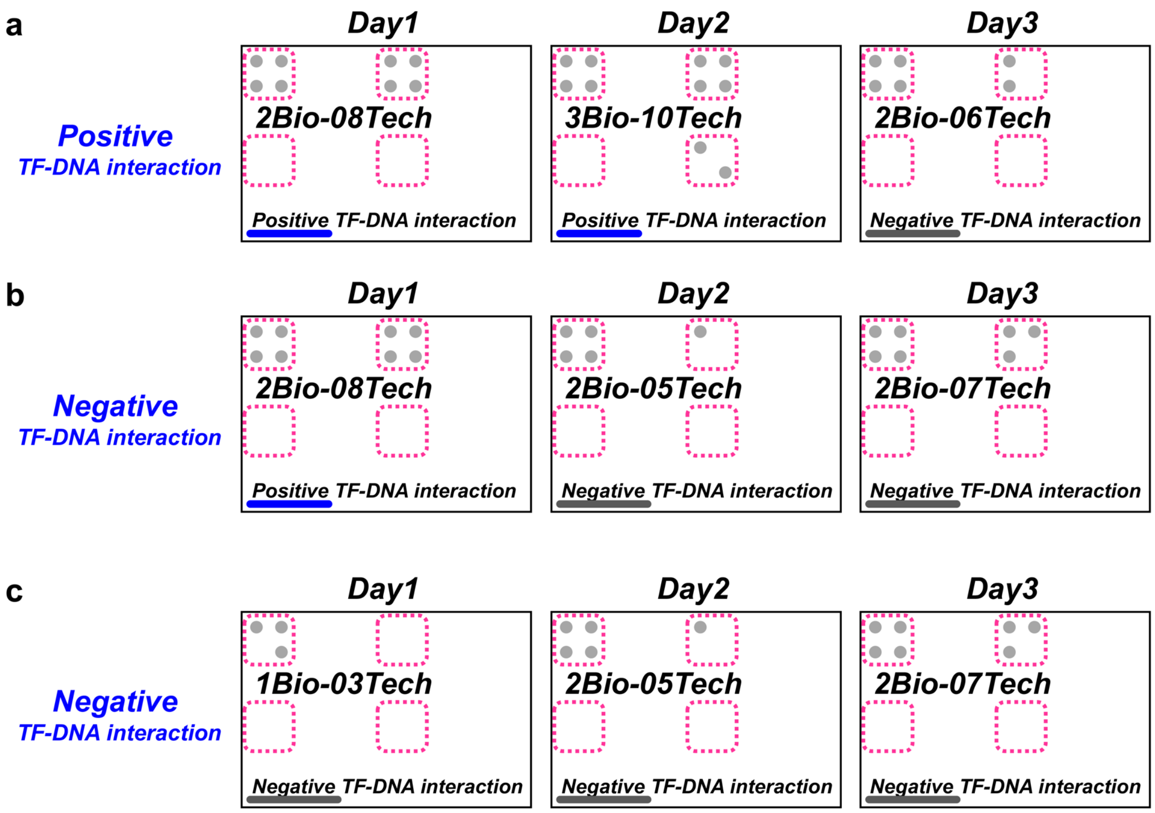

Figure S25. Positive cutoff. A TF-DNA combination contains 16 colonies as their biological and technical replicates on a selection plate. The 16 colonies were analyzed on 3 culturing days, Day1, Day2 and Day3, into positives or negatives. Positive and negative colonies are shown as grey dots and empties, respectively. Biological and technical replicate cutoff is set as 8 replicates. The TF-DNA combination with more than or equal to 8 positive replicates would be regarded as a positive interaction. For example, in (**a**), 2Bio-08Tech (as 8 positive technical replicates from 2 biological replicates) in Day1 and 3Bio-10Tech in Day2 were both counted as positives (black words with a blue underline), but 2Bio-06Tech in Day3 was a negative (black words with a black underline). To increase the analysis stringency, positive cutoff of culturing days can be set as 2. If a TF-DNA combination is regarded as a positive in more than or equal to 2 of the 3 culturing days, this TF-DNA combination would be interpreted as a positive TF-DNA interaction. For example, (**a**) The TF-DNA combination containing 2 positives on Day1 and Day2 was regarded as a positive TF-DNA interaction (blue words). **b, c** The TF-DNA combination containing (**b**) 1 positive on Day1 or (**c**) no positive were regarded as negative TF-DNA interactions (blue words).

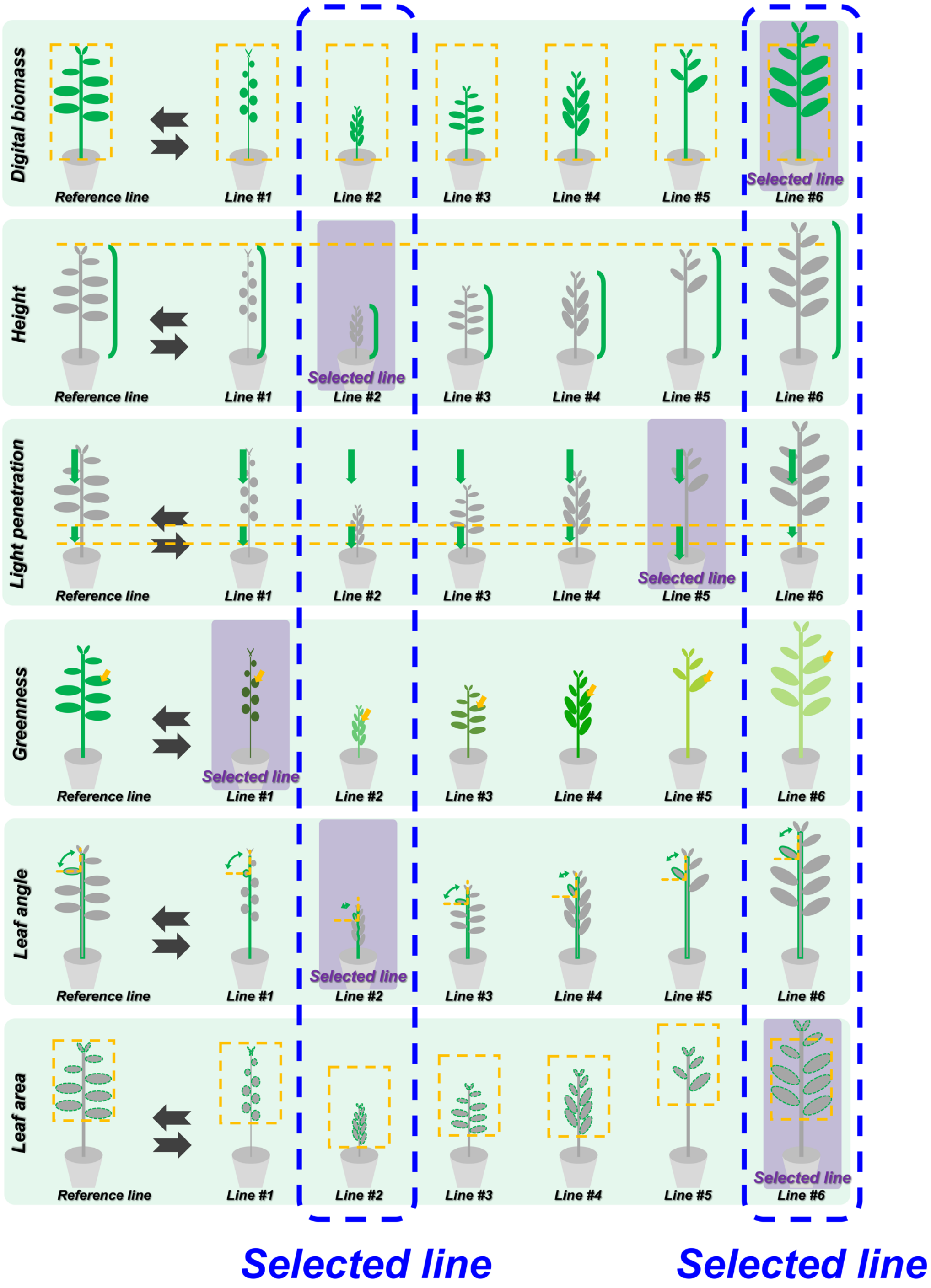

Figure S26. Positive cutoff in plant line selection. Multiple traits can be used for selecting plant lines in breeding, including digital biomass, height, light penetration, greenness, leaf angle, and leaf area. The lines, from line #1 to line #6, would be compared to a reference line to select the lines with required phenotypes. The plant lines with purple background were the selected lines for each trait. A plant line with more required phenotypes would be a better selection for breeding. In this case,

positive cutoff of traits was set as 2. If a plant line is selected in more than or equal to 2 traits (purple background), the line would be the final selected line (in blue words and blue dashed frame). For example, the line #2 was the selected line in both height and leaf angle (in purple background for two times), and was the final selected line (in blue words and blue dashed frame).
