# Supplementary material for: Large-scale data analysis for robotic yeast one-hybrid platforms and multi-disciplinary studies using GateMultiplex
Source: BMC Biol. 2021 Sep 24;19:214. doi: 10.1186/s12915-021-01140-y (PMC8461970; doi:10.1186/s12915-021-01140-y)
Supplement: Supplementary file 2 — Additional file 2. Detailed concept illustrations during the operating steps of GateMultiplex. [file 12915_2021_1140_MOESM2_ESM.pdf]

***NOTICE: To fully understand this manual, please read through the main text first**

# — Index —


Figure A1-A11. GM_Converter ········ 2-12

Figure B1-B4. Concepts about the input file ········ 13-16

Figure C. Background noise cutoff ········ 17

Figure D1-D4. Internal control cutoff ········ 18-21

Figure E1-E4. Reference cutoff (manual setting option) ········ 22-25

Figure F1-F2. Reference cutoff (percentile setting option) ········ 26-27

Figure G. Reference cutoff (fixed value setting option) ········ 28

Figure H. Combination of results after reference cutoff and background noise cutoff ········ 29

Figure I1-I3. Technical replicate cutoff ········ 30-32

Figure J. Biological replicate cutoff ········ 33

Figure K1-K3. Positive cutoff ········ 34-36

Figure L1-L3. Fold change file ········ 37-39

Figure M. PNE file ········ 40

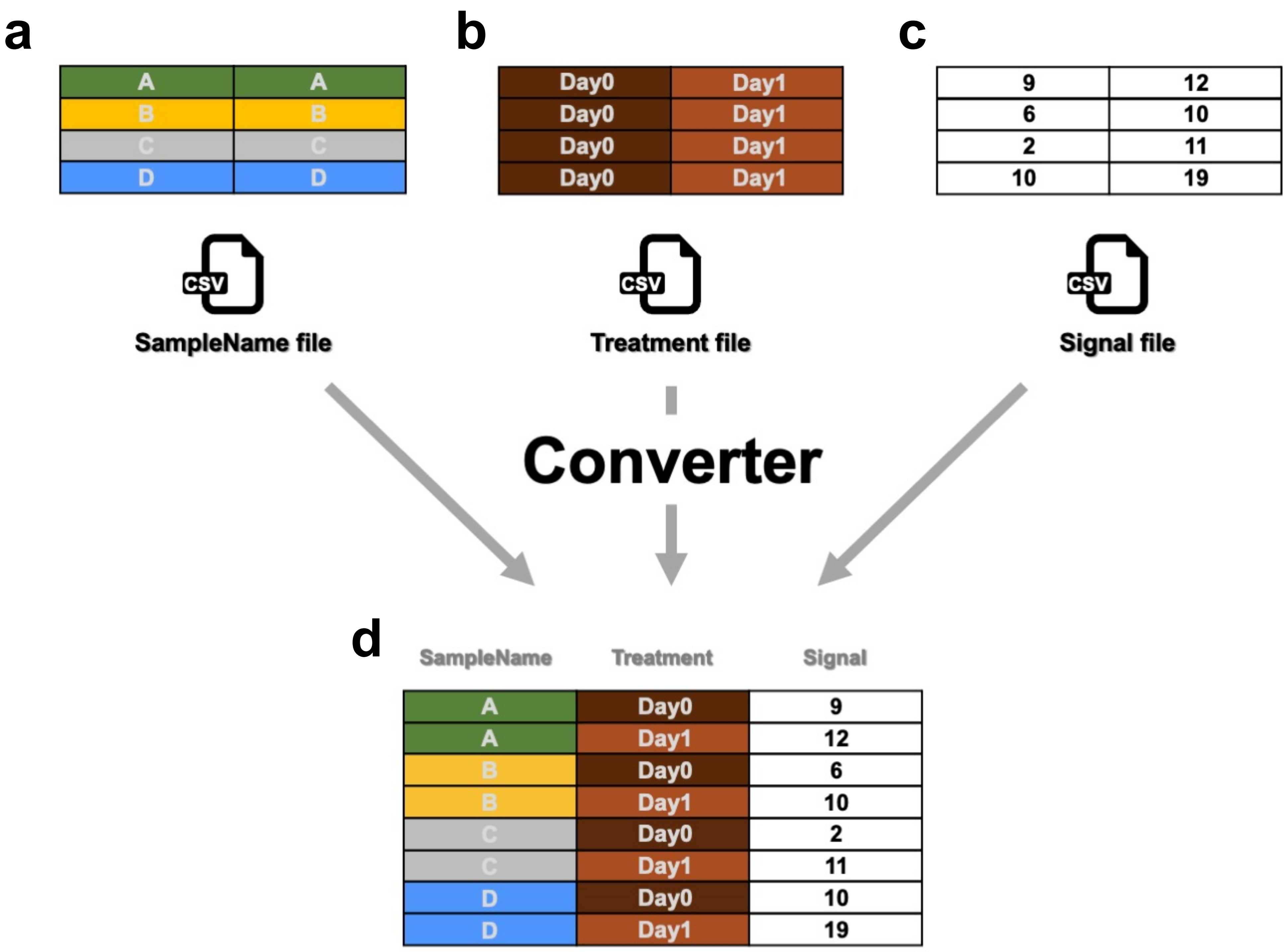


**Figure A1. The concept of GM_Converter**

The output experimental result files from high-throughput platforms may only contain signal data (shown as the “Signal” file (**c**)). To fit the required data format of the input file for GM (**d**), GM_Converter was designed to convert and merge the “Signal” file with other files provided by the users, including the (**a**) “SampleName” file and (**b**) “Treatment” file.

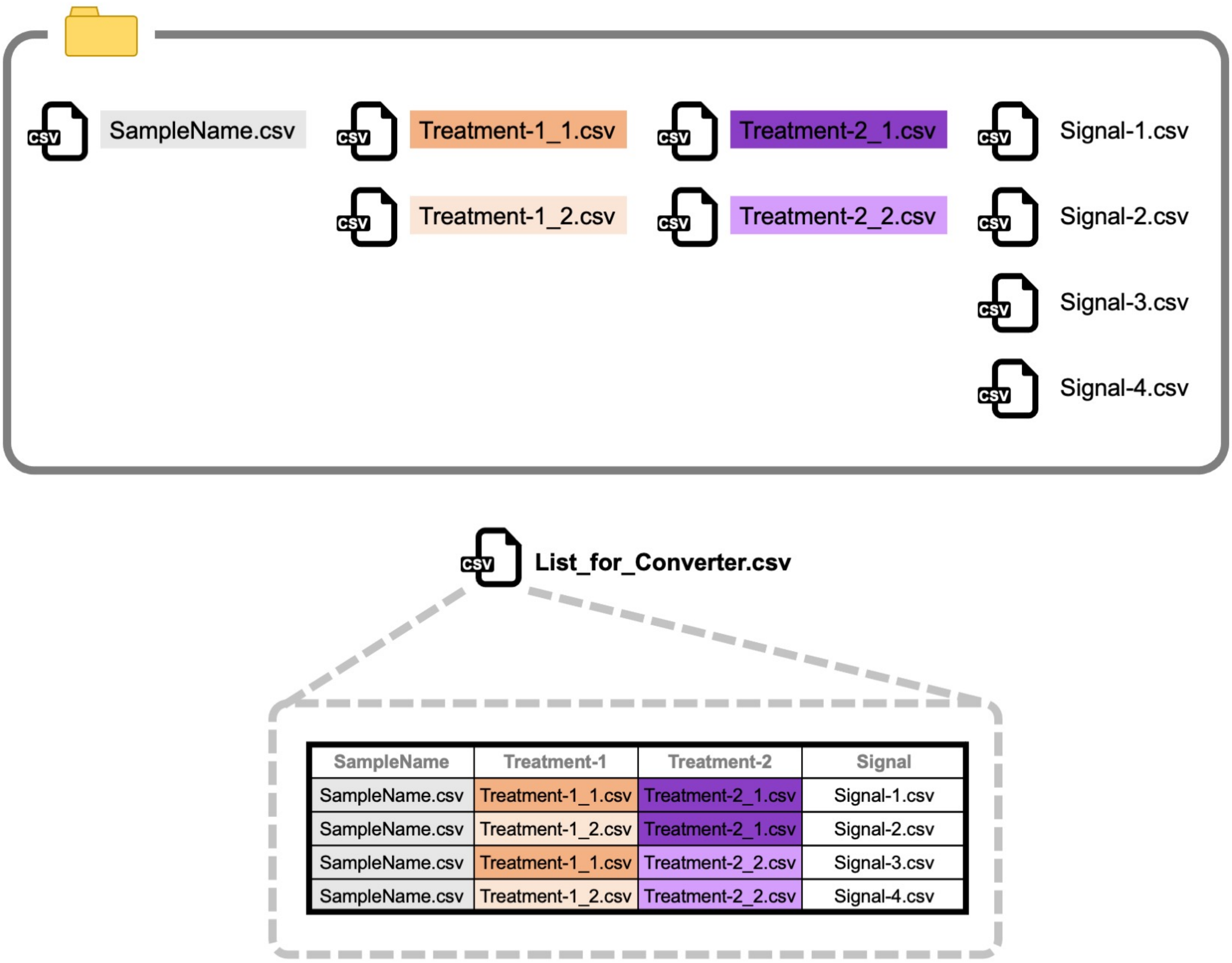


**Figure A2. Files needed for GM_Converter**

To fit the required data format for GM, four file types need to be prepared for GM_Converter. The file types include “Signal” files provided by high-throughput platforms, “Treatment” files, “SampleName” files, and a “List_for_Converter” file provided by the users. All files should be prepared in csv file format. “List_for_Converter” file contains the file names of “Signal” files, “Treatment” files and “SampleName” files. The file names listed on the same row of “List_for_Converter” file would be integrated. Take the second row of the “List_for_Converter” file for example, “SampleName.csv” (grey) contains the sample name information for “Signal-1.csv”. “Treatment-1_1.csv” (orange) and “Treatment-2_1.csv” (purple) contain the treatment information for “Signal-1.csv”. The file names listed on the same column belong to the same category. Take the second column for example, ”Treatment-1_1.csv” (orange) and “Treatment-1_2.csv” (light orange) both belong to “Treatment-1”, which is the title of the second column. “List_for_Converter” file would be used as the index for GM_Converter. The file names listed on “List_for_Converter.csv” must include the file format “.csv”. The name of “List_for_Converter” file has to be fixed.

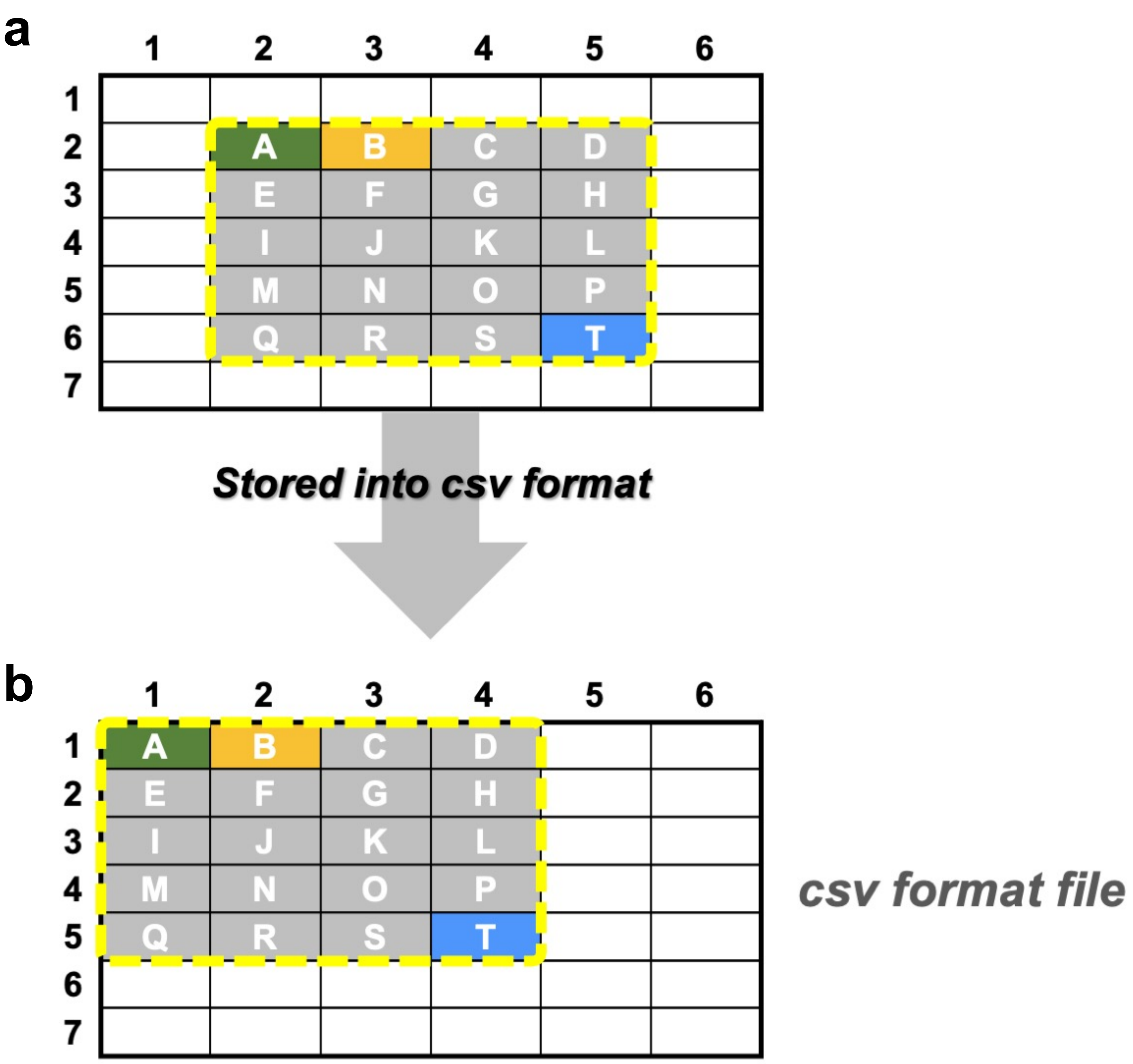


**Figure A3. Data shift after format changing (cluster data)**

**a** If the cells on upper rows or left columns of the data area (shown in the yellow dashed line frame) are empty, (**b**) the data area will be shifted to the most top and left boundaries after being stored into csv format.

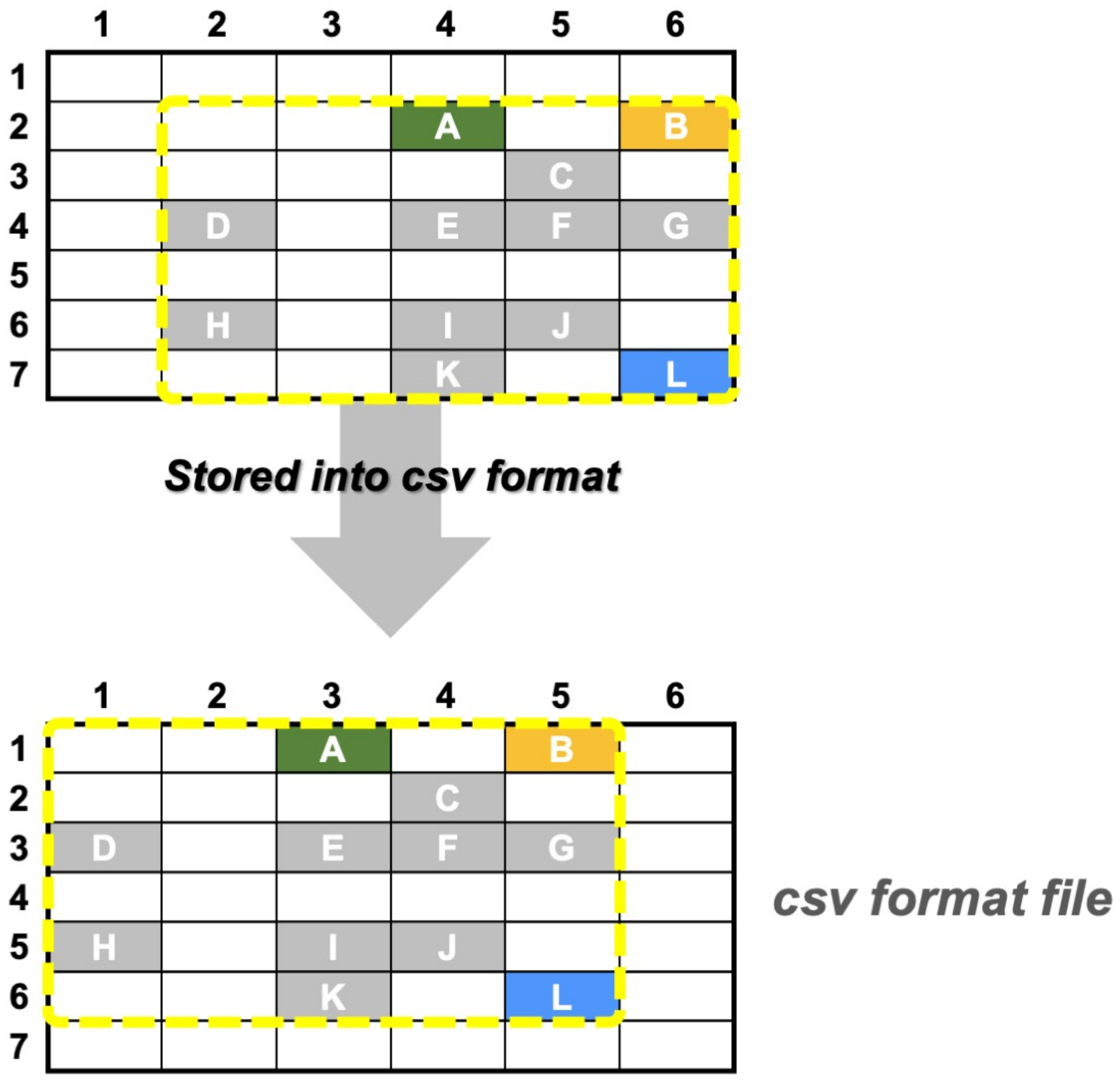


**Figure A4. Data shift after format changing (scattered data)**

Another example in addition to Figure A3.

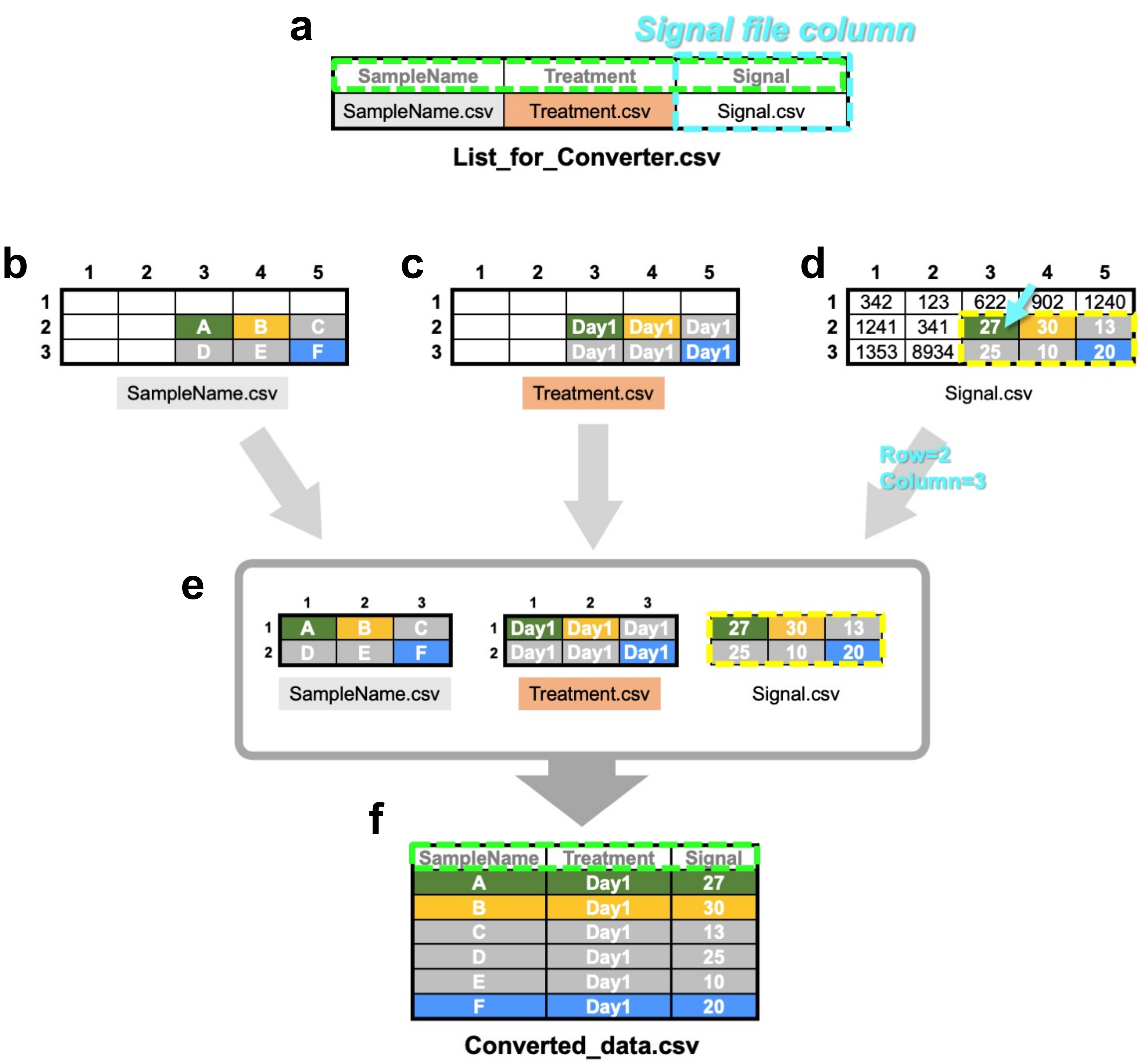


**Figure A5. The Function of GM_Converter ( for single “Signal file”)**

During the operation of GM_Converter, two input information are needed to be identified by the users. **a** The first information is the column title of “Signal” file in the “List_for_Converter” file (light blue dashed line). For example, the column title of “Signal” file here is shown as Signal. **d** The second information is the coordinate of upper left hand corner of the signal data in the “Signal” file (the data in green box indicated by the light blue arrow), which would be used as the starting coordinate for converting the signal data. Because of the shifting event explained in Figure A3 and A4, both starting coordinates of “SampleName” file (**b**) and “Treatment” file (**c**) would be row=1 and column=1 (**e**). GM_Converter can then integrate the data from “Signal” file, “Treatment” file and “SampleName” file into the “Converted_data” (**f**) in csv format. The data in the first row of “Converted_data” file is from the first row of “List_for_Converter” file (green dashed lines). This “Converted_data” file would then be used as the input file for GateMultiplex operation.

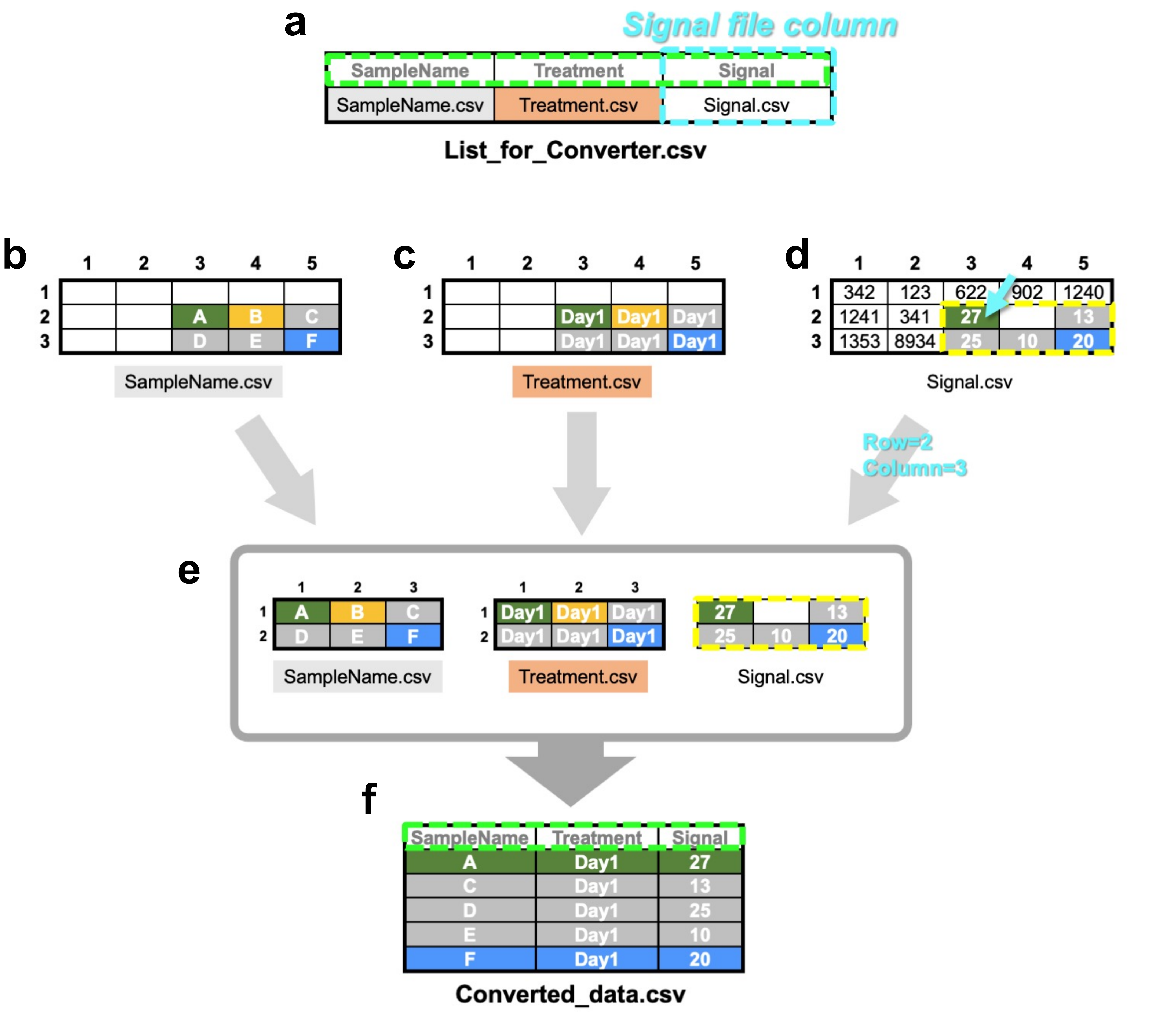


**Figure A6. The Function of GM_Converter ( for single "Signal file") – skipped data point**

During the operation of GM_Converter, if any of the cells is empty in the corresponding positions among the "Signal" file, "Treatment" file and "SampleName" file, then the data in other relative cells will be ignored. For example, the "Treatment" file (**b**) and "SampleName" file (**c**) both have data in their orange cells. In contrast, no data is on the relative cell in the "Signal" file (**d**), so the data in the orange cells would be excluded in the "Converted_data" file.

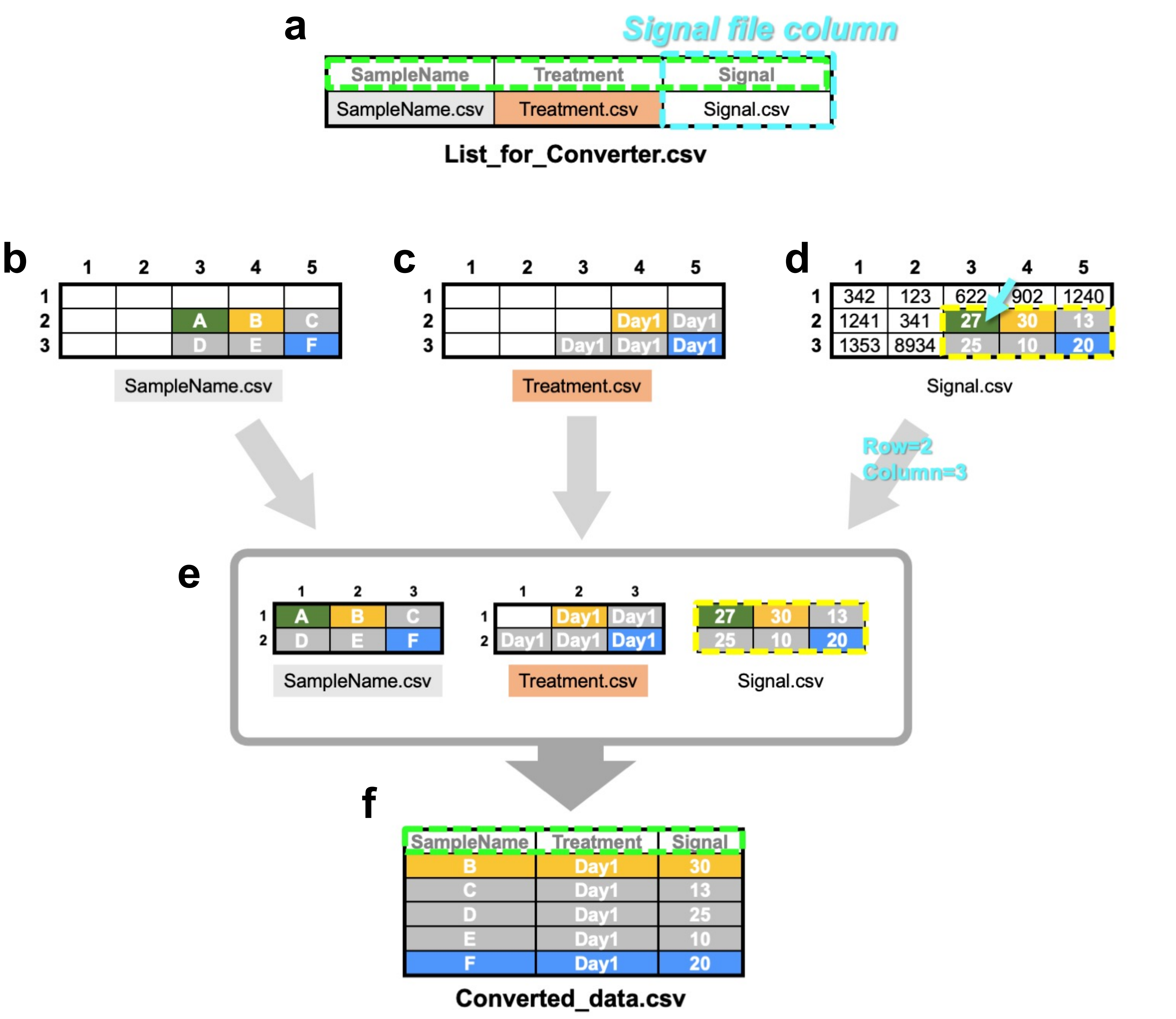


**Figure A7. The Function of GM_Converter ( for single "Signal file") – skipped data point**

Another example for skipped data point situation.

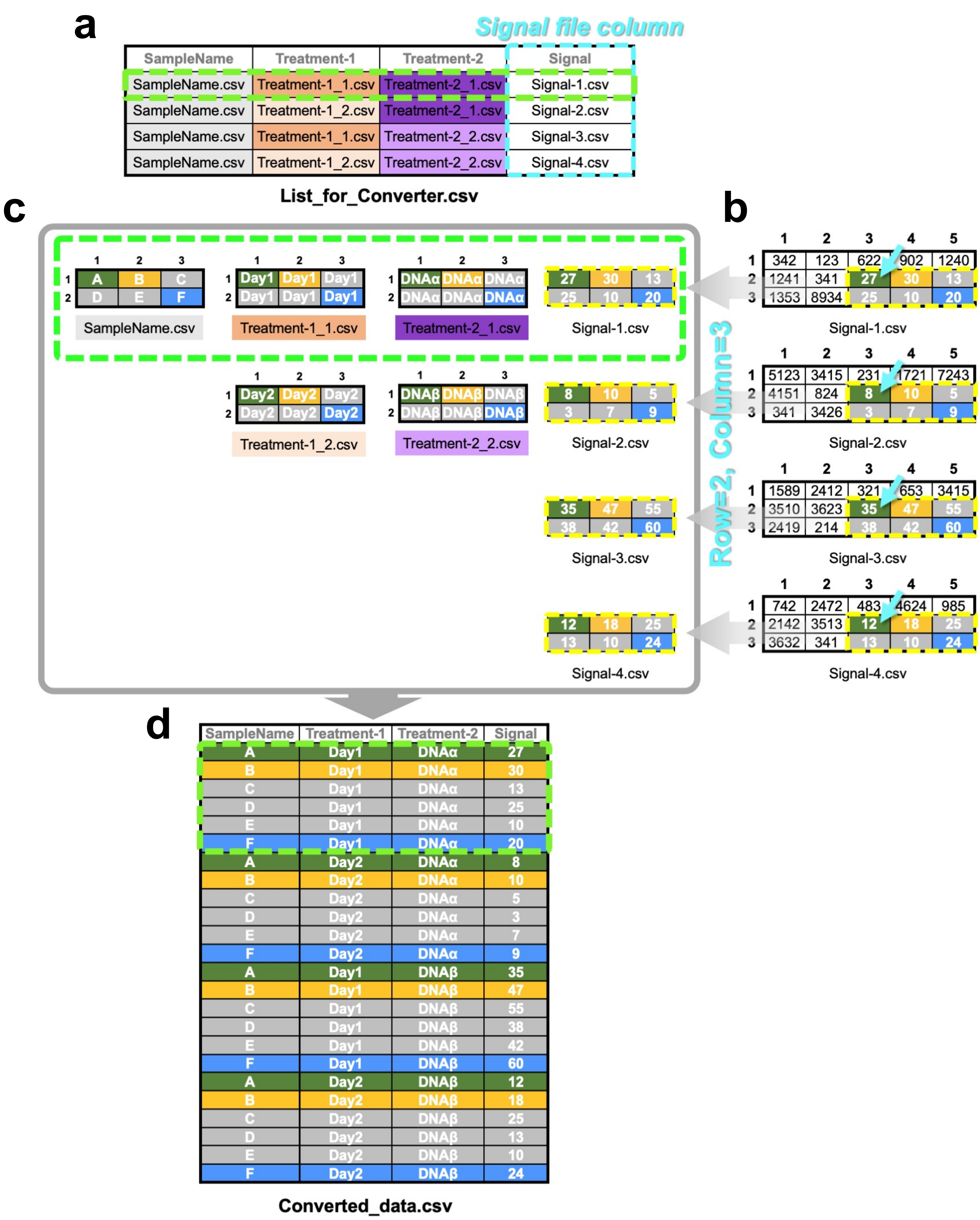


**Figure A8. The Function of GM_Converter (Multiple Signal files) – (1)**

High-throughput platforms sometimes generate their signal data into multiple files ((**a**) light blue dashed line) in same format (**b**). Similar to operating single “Signal” file, the users also need to provide two information, the coordinate in the “Signal” files and the column title of the “Signal” files in the “List_for_Converter” file. Because all “Signal” files are in the same format, they can be applied with the same starting coordinate ((**b**) light blue arrow). Through identifying the column title of the “Signal” files in the “List_for_Converter” file, the data in the “Signal” files, the “Treatment” files and the “SampleName” files would be integrated and converted into the “Converted_data” file (**d**). We take the conversion of the data of the first row in the “List_for_Converter” file as an example ((**a**) marked in the green dashed box). The data from each file would be extracted (**c**) and converted into the “Converted_data” file (**d**).

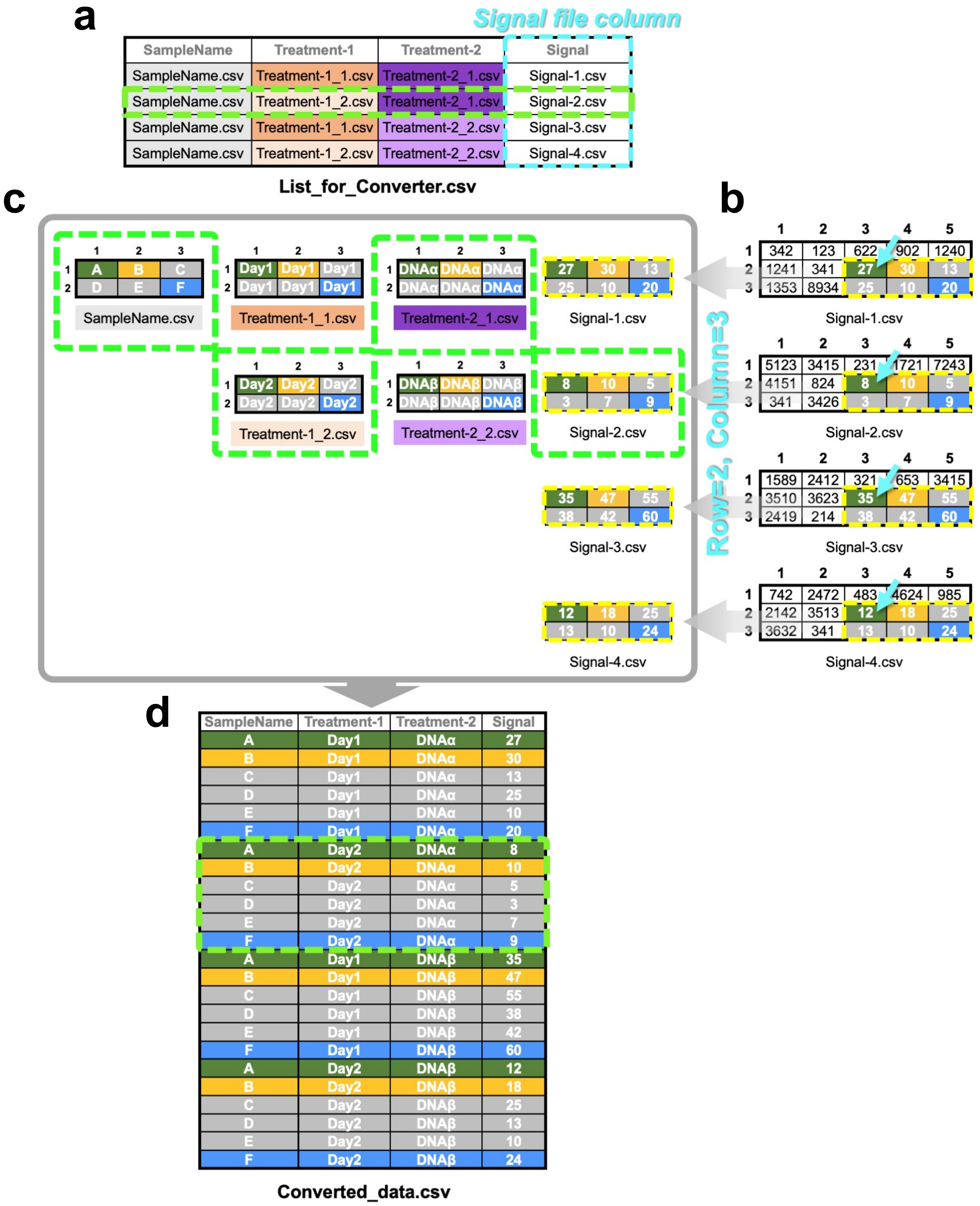


## Figure A9. The Function of GM_Converter (Multiple Signal files) – (2)

Another example for multiple "Signal" files.

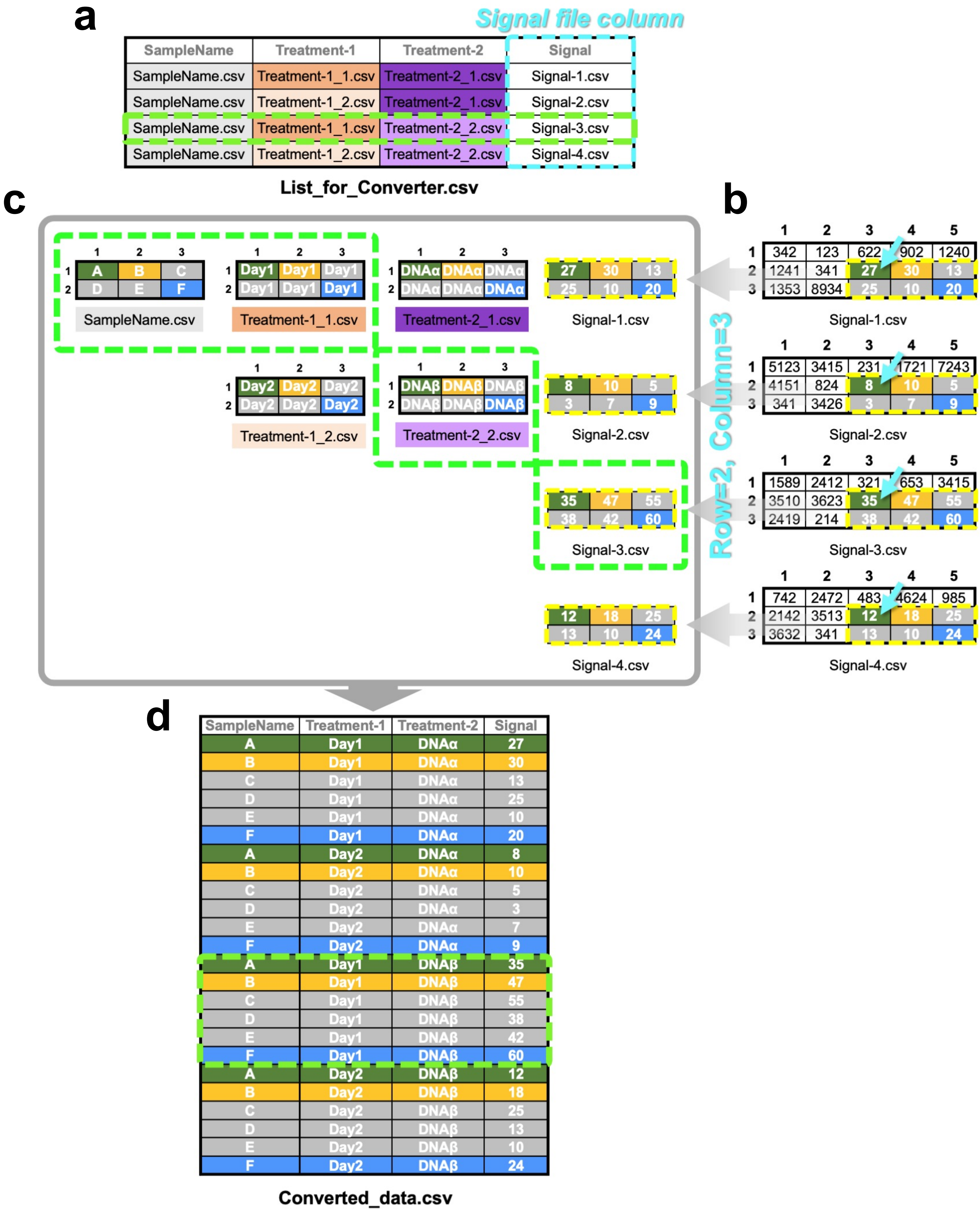


**Figure A10. The Function of GM_Converter (Multiple Signal files) – (3)**

Another example for multiple "Signal" files.

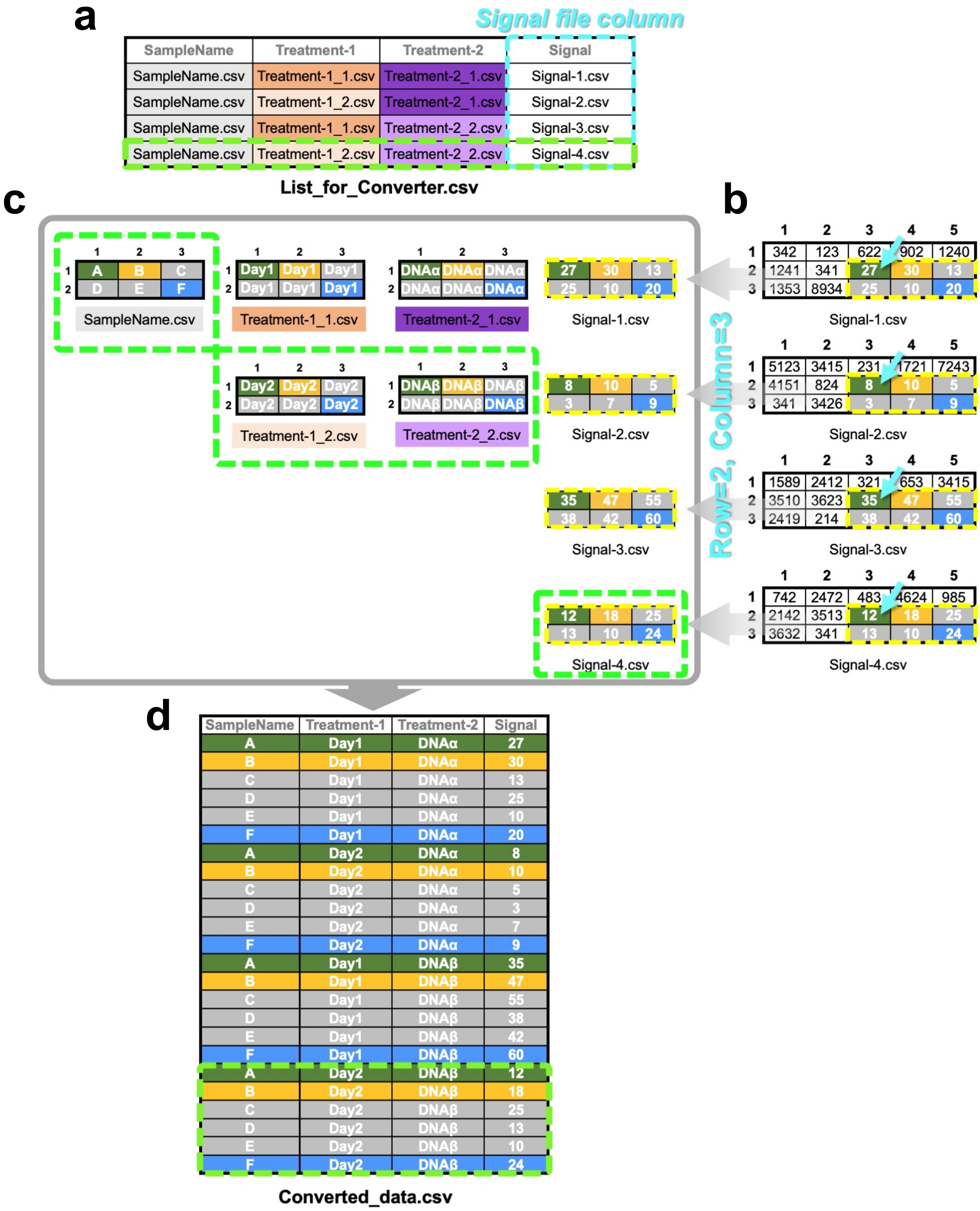


**Figure A11. The Function of GM_Converter (Multiple Signal files) – (4)**

Another example for multiple "Signal" files.

| SampleName | Treatment-1 | Treatment-2 | Signal |
|---|---|---|---|
| A | DNAα | Day1 | 5 |
| B | DNAα | Day1 | 12 |
| C | DNAα | Day1 | 8 |
| D | DNAα | Day1 | 14 |
| A | DNAβ | Day1 | 15 |
| B | DNAβ | Day1 | 20 |
| C | DNAβ | Day1 | 18 |
| D | DNAβ | Day1 | 2 |

| SampleName | Treatment-1 | Treatment-2 | Signal |
|---|---|---|---|
| A | DNAα | Day2 | 15 |
| B | DNAα | Day2 | 24 |
| C | DNAα | Day2 | 31 |
| D | DNAα | Day2 | 19 |
| A | DNAβ | Day2 | 24 |
| B | DNAβ | Day2 | 28 |
| C | DNAβ | Day2 | 30 |
| D | DNAβ | Day2 | 17 |

| SampleName | Treatment-1 | Treatment-2 | Signal |
|---|---|---|---|
| A | DNAα | Day3 | 30 |
| B | DNAα | Day3 | 45 |
| C | DNAα | Day3 | 37 |
| D | DNAα | Day3 | 44 |
| A | DNAβ | Day3 | 51 |
| B | DNAβ | Day3 | 58 |
| C | DNAβ | Day3 | 36 |
| D | DNAβ | Day3 | 41 |

| SampleName | Treatment-1 | Treatment-2 | Signal |
|---|---|---|---|
| A | DNAα | Day1 | 5 |
| B | DNAα | Day1 | 12 |
| C | DNAα | Day1 | 8 |
| D | DNAα | Day1 | 14 |
| A | DNAβ | Day1 | 15 |
| B | DNAβ | Day1 | 20 |
| C | DNAβ | Day1 | 18 |
| D | DNAβ | Day1 | 2 |
| A | DNAα | Day2 | 15 |
| B | DNAα | Day2 | 24 |
| C | DNAα | Day2 | 31 |
| D | DNAα | Day2 | 19 |
| A | DNAβ | Day2 | 24 |
| B | DNAβ | Day2 | 28 |
| C | DNAβ | Day2 | 30 |
| D | DNAβ | Day2 | 17 |
| A | DNAα | Day3 | 30 |
| B | DNAα | Day3 | 45 |
| C | DNAα | Day3 | 37 |
| D | DNAα | Day3 | 44 |
| A | DNAβ | Day3 | 51 |
| B | DNAβ | Day3 | 58 |
| C | DNAβ | Day3 | 36 |
| D | DNAβ | Day3 | 41 |

**Figure B1. Multiple input files**

When the users have multiple input files, the data from different files will be integrated into one file. And the first row of the integrated file, containing column titles, will be incorporated using that of the alphabetically first input files.

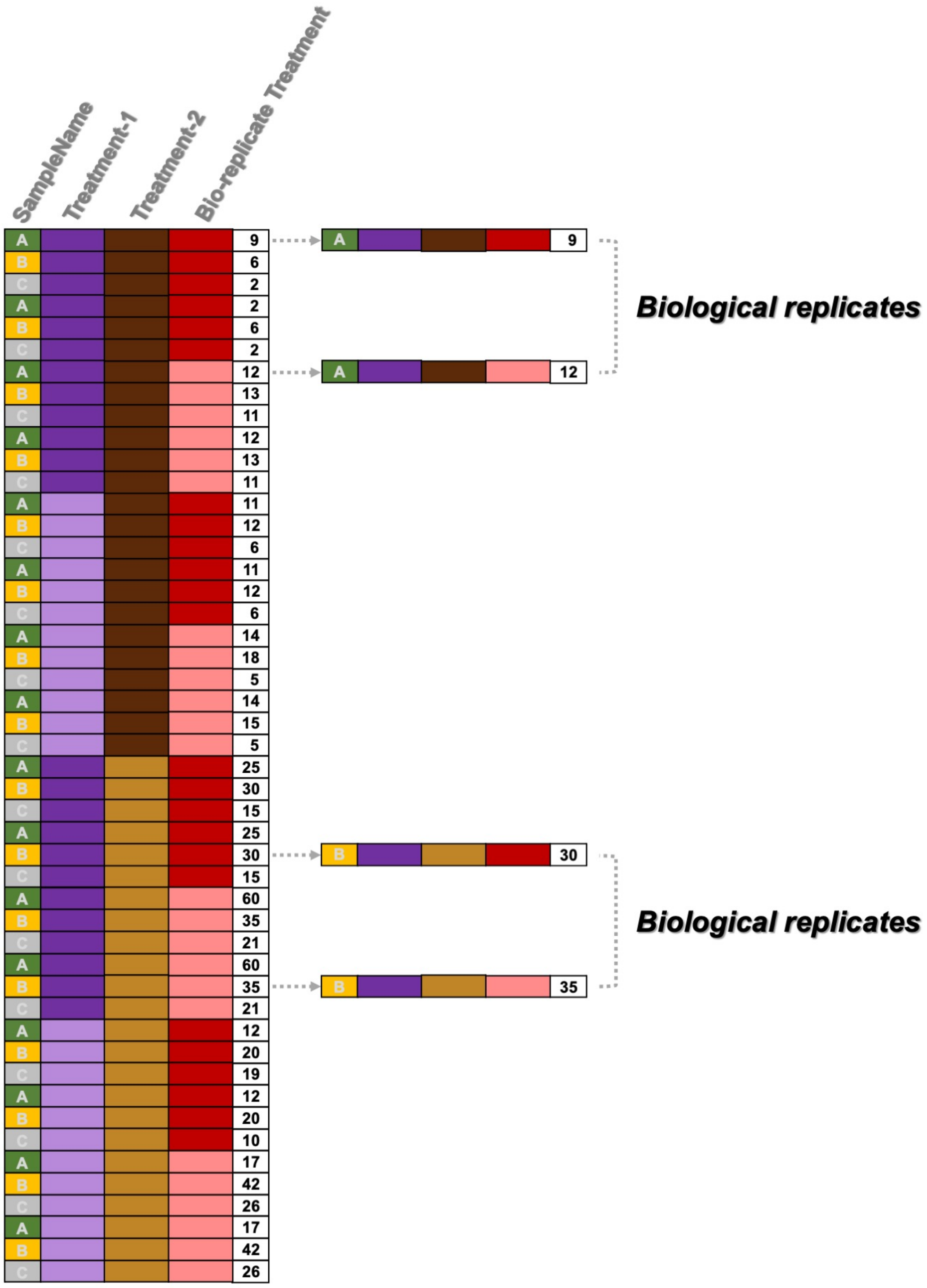


**Figure B2. Functions of Treatment and Bio-replicate Treatment**

During the identification of the required data column in the input file using GUI, two types of treatments can be used, the "Treatment" and the "Bio-replicate Treatment". The "Treatment" is used to divide the signals into different groups during reference cutoff analysis. In this figure, the "Treatment" includes "Treatment-1" and "Treatment-2". The "Bio-replicate Treatment" would not divide the signal, but is used as a tag to identify the biological replicates. Take SampleName A as an example, it contains several "Condition", including dark/light and dark/light brown. In SampleName A with dark purple and dark brown "Condition", the signal of A-dark purple-dark brown-red and A-dark purple-dark brown-pink will be recognized as biological replicates.

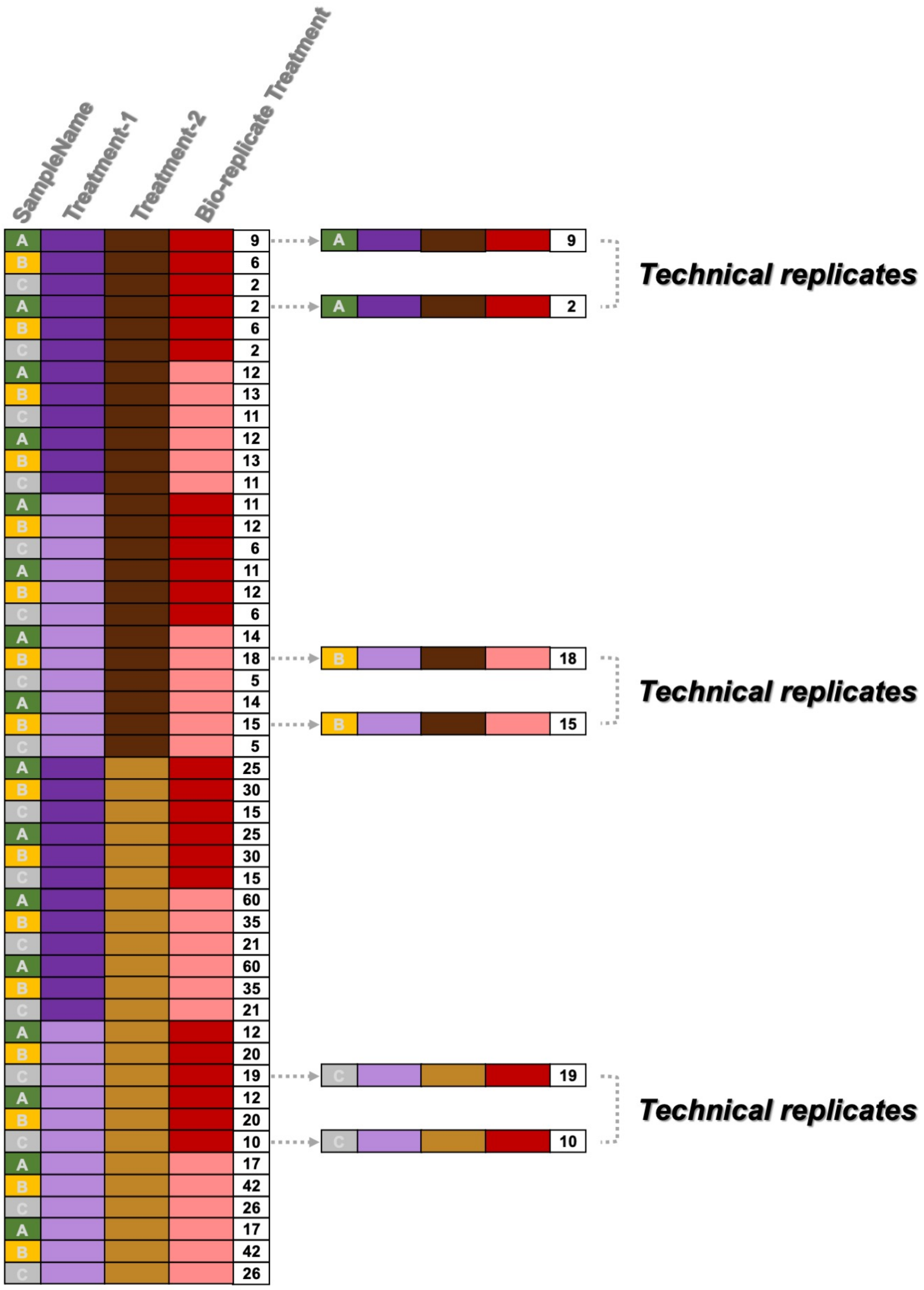


**Figure B3. Technical replicates**

The signals with exactly same “SampleName”, “Condition” of “Treatment”, including Treatment-1 and Treatment-2, and “Condition” of “Bio-replicate Treatment” would be recognized as technical replicates.

**a**

| SampleName | Treatment-1 | Treatment-2 | Signal-1 Day0 | Signal-2 Day1 |
|---|---|---|---|---|
| A | DNA-bait (α) | Diploid | 9 | 25 |
| B | DNA-bait (α) | Diploid | 6 | 30 |
| C | DNA-bait (α) | Diploid | 2 | 15 |
| D | DNA-bait (α) | Diploid | 10 | 13 |
| A | DNA-bait (β) | Diploid | 12 | 60 |
| B | DNA-bait (β) | Diploid | 10 | 35 |
| C | DNA-bait (β) | Diploid | 11 | 21 |
| D | DNA-bait (β) | Diploid | 19 | 31 |
| A | DNA-bait (α) | Meiosis | 3 | 12 |
| B | DNA-bait (α) | Meiosis | 8 | 20 |
| C | DNA-bait (α) | Meiosis | 6 | 19 |
| D | DNA-bait (α) | Meiosis | 7 | 26 |
| A | DNA-bait (β) | Meiosis | 14 | 17 |
| B | DNA-bait (β) | Meiosis | 18 | 42 |
| C | DNA-bait (β) | Meiosis | 5 | 26 |
| D | DNA-bait (β) | Meiosis | 8 | 11 |

**b**

| SampleName | Treatment-1 | Treatment-2 | Treatment-3 (signal_type) | Signal |
|---|---|---|---|---|
| A | DNA-bait (α) | Diploid | Day0 | 9 |
| B | DNA-bait (α) | Diploid | Day0 | 6 |
| C | DNA-bait (α) | Diploid | Day0 | 2 |
| D | DNA-bait (α) | Diploid | Day0 | 10 |
| A | DNA-bait (β) | Diploid | Day0 | 12 |
| B | DNA-bait (β) | Diploid | Day0 | 10 |
| C | DNA-bait (β) | Diploid | Day0 | 11 |
| D | DNA-bait (β) | Diploid | Day0 | 19 |
| A | DNA-bait (α) | Meiosis | Day0 | 3 |
| B | DNA-bait (α) | Meiosis | Day0 | 8 |
| C | DNA-bait (α) | Meiosis | Day0 | 6 |
| D | DNA-bait (α) | Meiosis | Day0 | 7 |
| A | DNA-bait (β) | Meiosis | Day0 | 14 |
| B | DNA-bait (β) | Meiosis | Day0 | 18 |
| C | DNA-bait (β) | Meiosis | Day0 | 5 |
| D | DNA-bait (β) | Meiosis | Day0 | 8 |
| A | DNA-bait (α) | Diploid | Day1 | 25 |
| B | DNA-bait (α) | Diploid | Day1 | 30 |
| C | DNA-bait (α) | Diploid | Day1 | 15 |
| D | DNA-bait (α) | Diploid | Day1 | 13 |
| A | DNA-bait (β) | Diploid | Day1 | 60 |
| B | DNA-bait (β) | Diploid | Day1 | 35 |
| C | DNA-bait (β) | Diploid | Day1 | 21 |
| D | DNA-bait (β) | Diploid | Day1 | 31 |
| A | DNA-bait (α) | Meiosis | Day1 | 12 |
| B | DNA-bait (α) | Meiosis | Day1 | 20 |
| C | DNA-bait (α) | Meiosis | Day1 | 19 |
| D | DNA-bait (α) | Meiosis | Day1 | 26 |
| A | DNA-bait (β) | Meiosis | Day1 | 17 |
| B | DNA-bait (β) | Meiosis | Day1 | 42 |
| C | DNA-bait (β) | Meiosis | Day1 | 26 |
| D | DNA-bait (β) | Meiosis | Day1 | 11 |

**Figure B4. Multiple signal columns and signal_type**

When an input data contains multiple signal columns (**a**), the multiple signal columns would be converted into one signal column with an additional new “Treatment” column titled “signal_type” (**b**). The “Condition” of the new “Treatment” would be the original titles of the multiple signal columns.

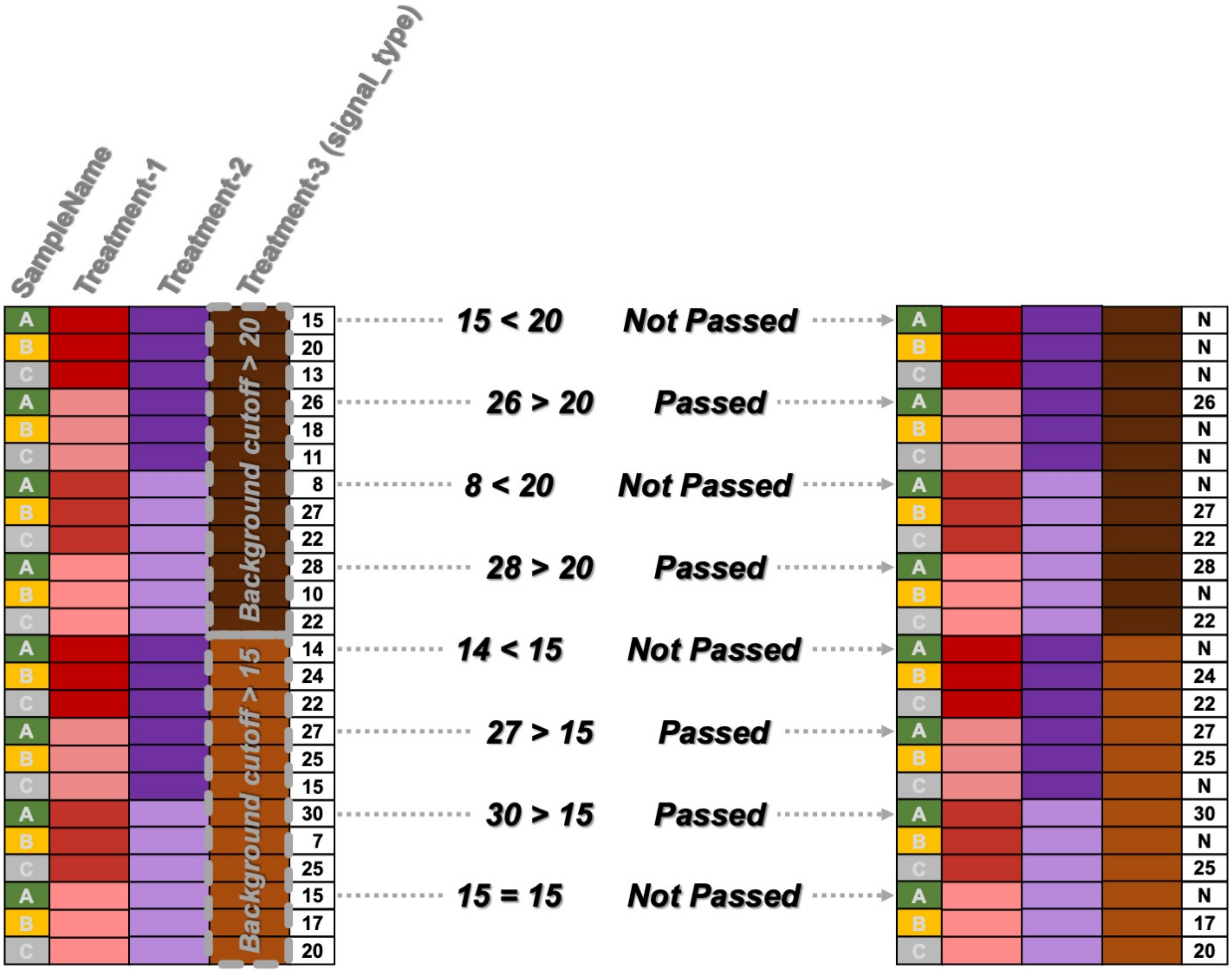


**Figure C. Background noise cutoff**

In background noise cutoff analysis, the users can provide a fixed value as the cutoff value. If the input data has more than one "Condition" of "signal_type" (the dark and light brown), the users can set different values for each "Condition" of "signal_type" as background noise cutoff values. For example, in this figure, the cutoff value of dark brown "Condition" of Treatment-3 (signal_type) was set as 20, while the light brown "Condition" of Treatment-3 (signal_type) was set as 15. If a signal passes background noise cutoff, then the signal value is remained; if not, then this signal would be processed into an N as a negative. Background noise cutoff is an optional function.

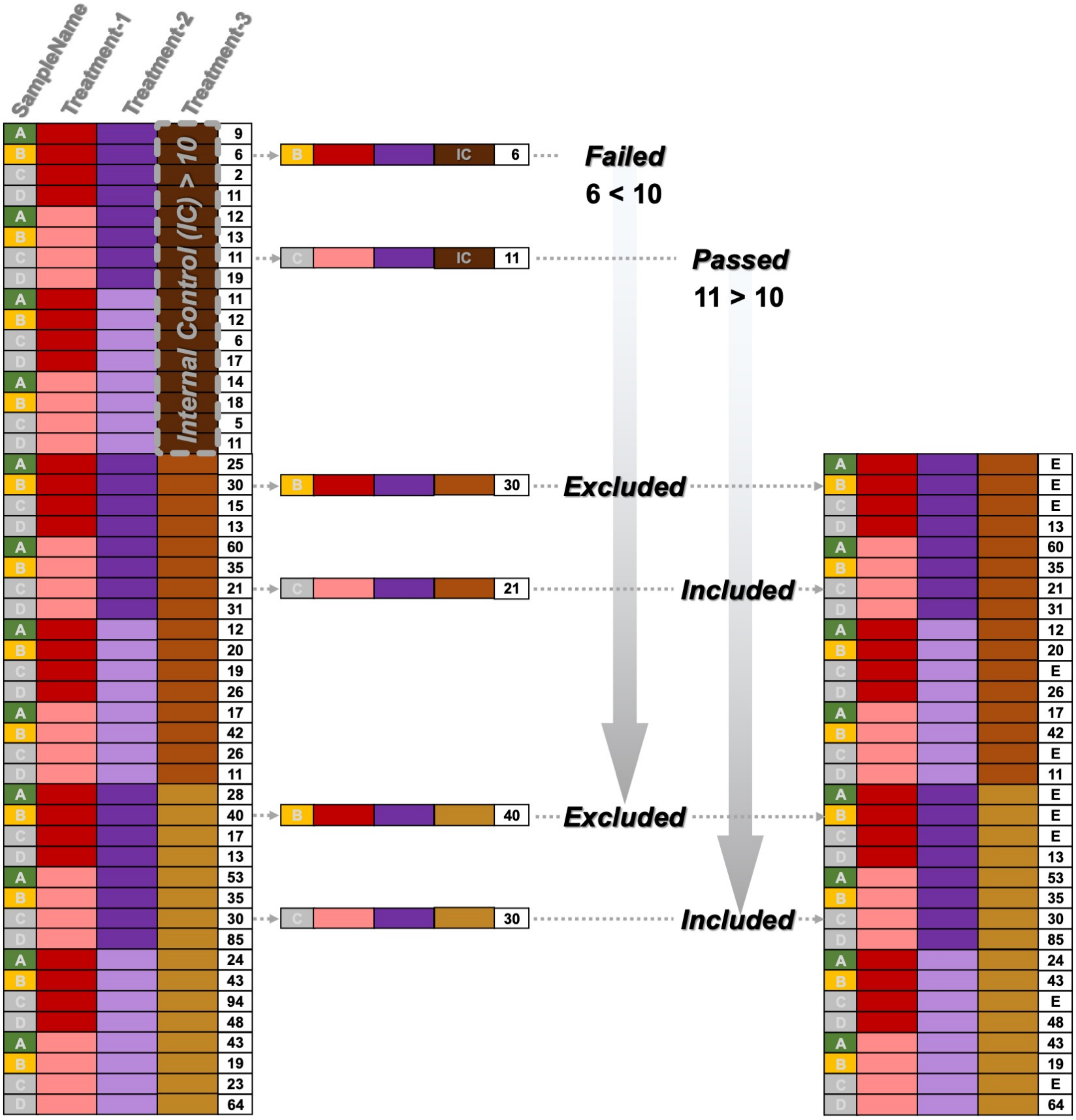


**Figure D1. The function of internal control cutoff**

The users can select one “Condition” in one “Treatment” as the internal control “Condition”. In this figure, the dark brown “Condition” in Treatment-3 was selected as the internal control “Condition”, and internal control cutoff was set as 10. Take the B-red-dark purple sample for example, the signal of B-red-dark purple-dark brown was 6, and was lower than the cutoff value 10, which would cause the exclusion (E) of the signal from B-red-dark purple-red brown and B-red-dark purple-light brown. Another example, the signal of C-pink-dark purple-dark brown passed the cutoff, and then allowed the signal of C-red-dark purple-red brown and C-red-dark purple-light brown to be further processed. Internal control cutoff is an optional function.

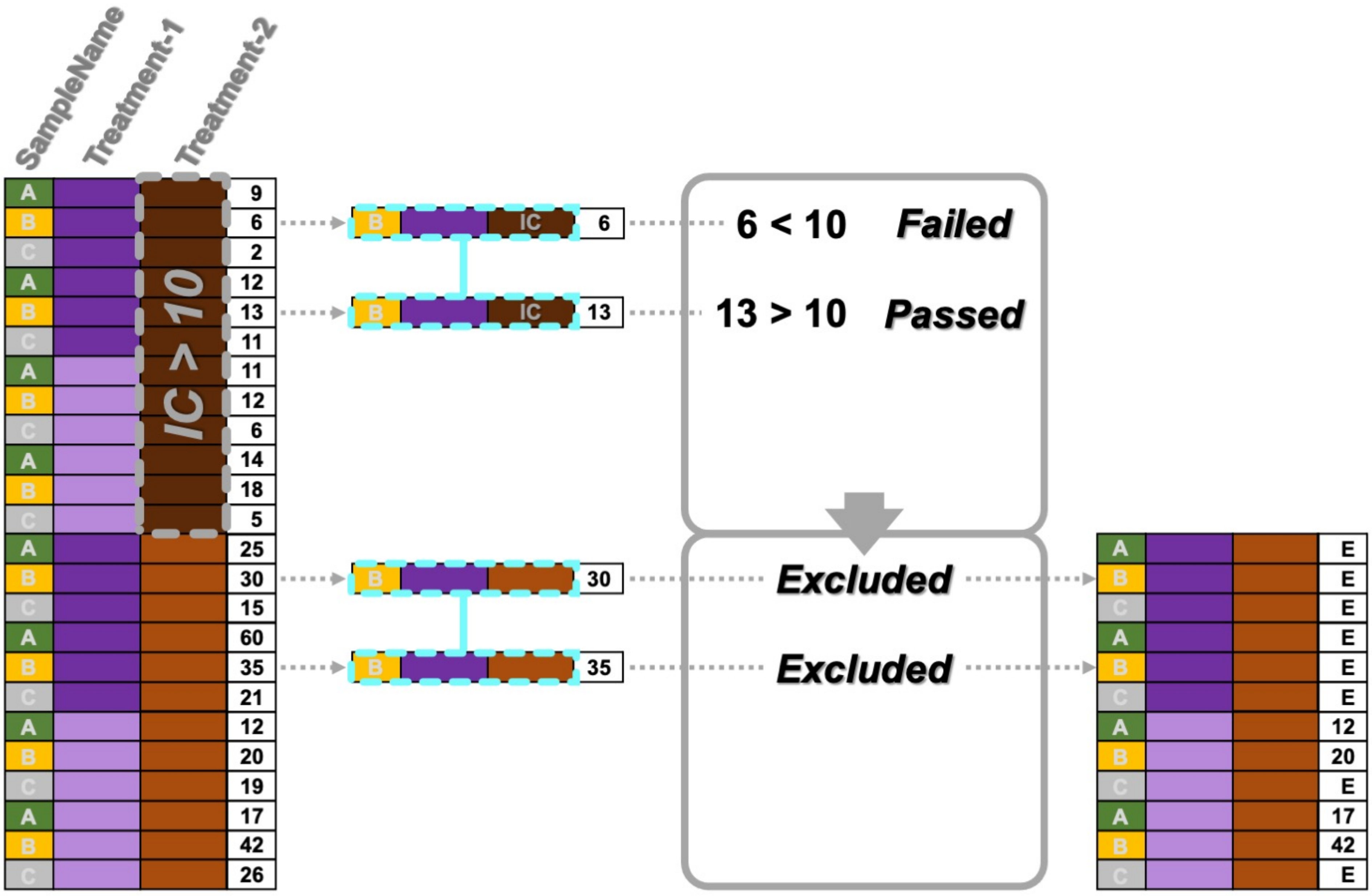


**Figure D2. Internal control cutoff with technical replicates**

Technical replicates were clustered as a group (light blue dashed lines frames) during internal control cutoff analysis. The group selected as internal control "Condition" would affect the results of the corresponding non-internal control "Condition" group. Take B-dark purple for example, when one of the two technical replicates, was lower than the cutoff value 10 (here as B-dark purple-dark brown), then the signals in relative group, B-dark purple-red brown, were both excluded (E).

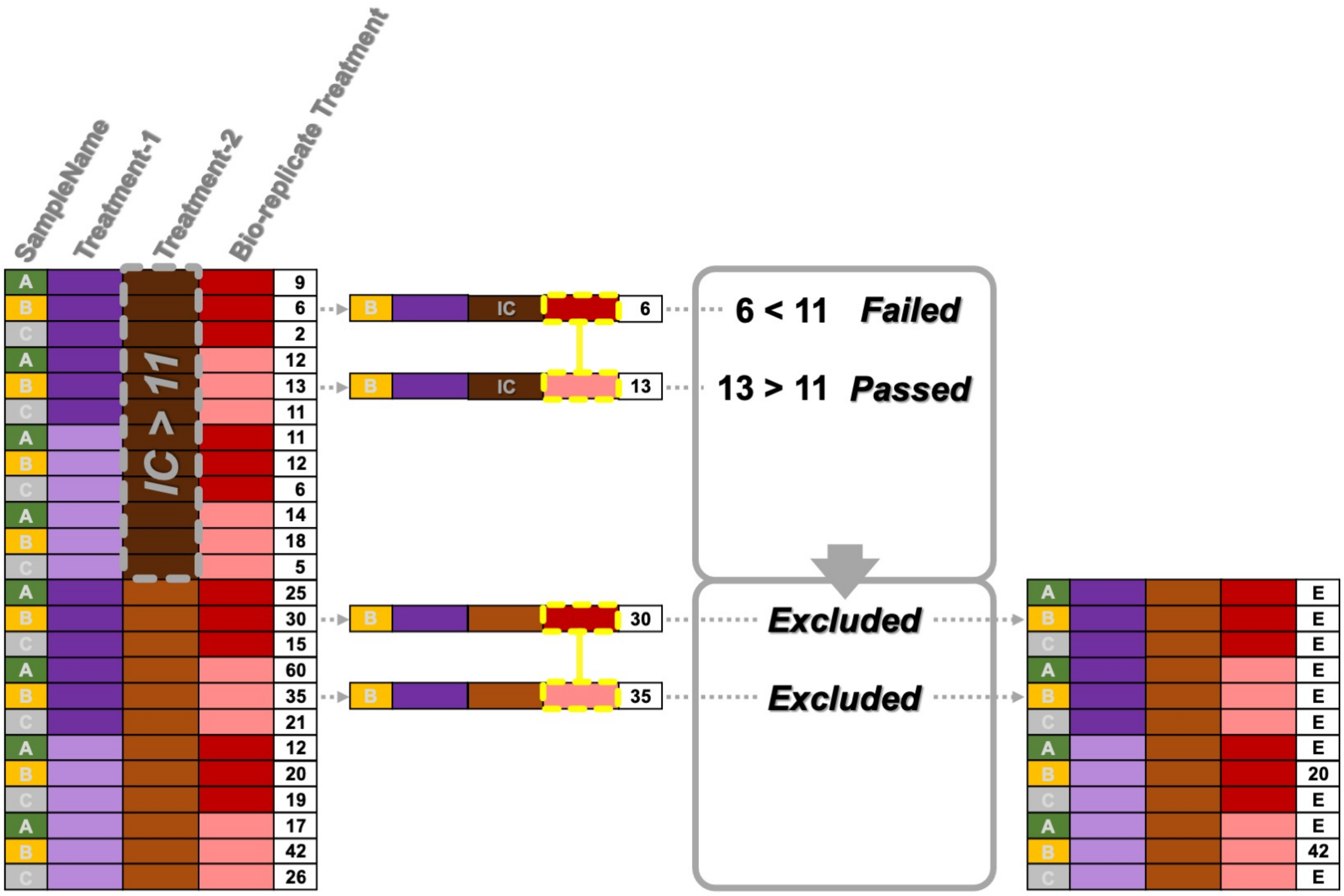


**Figure D3. Internal control cutoff with biological replicates**

Biological replicates involved in internal control cutoff in the same way as the technical replicates.

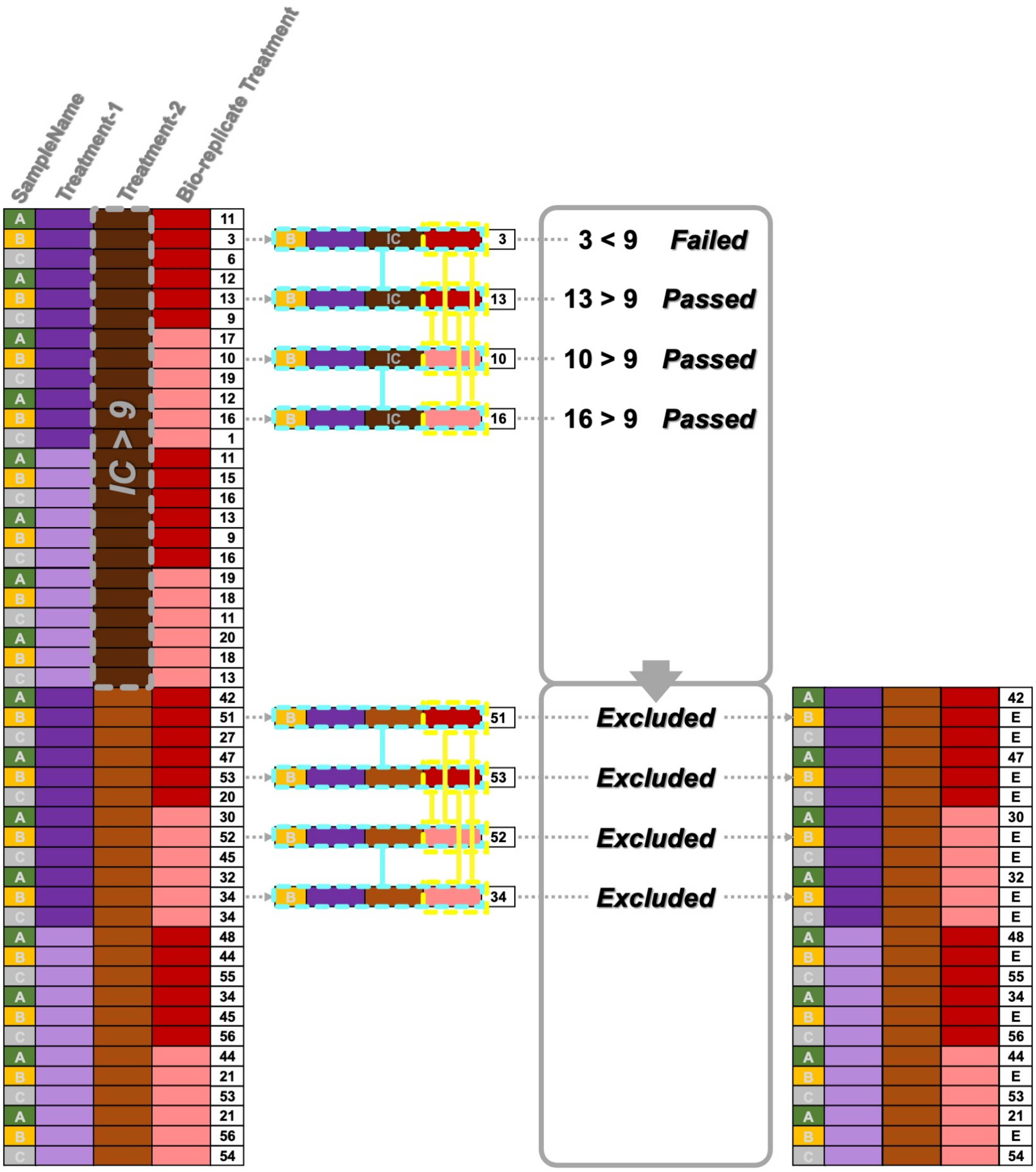


**Figure D4. Internal control cutoff with both technical replicates and biological replicates**

The samples contain both biological replicates and technical replicates were processed with internal control cutoff in the same way as Figure D2 and D3.

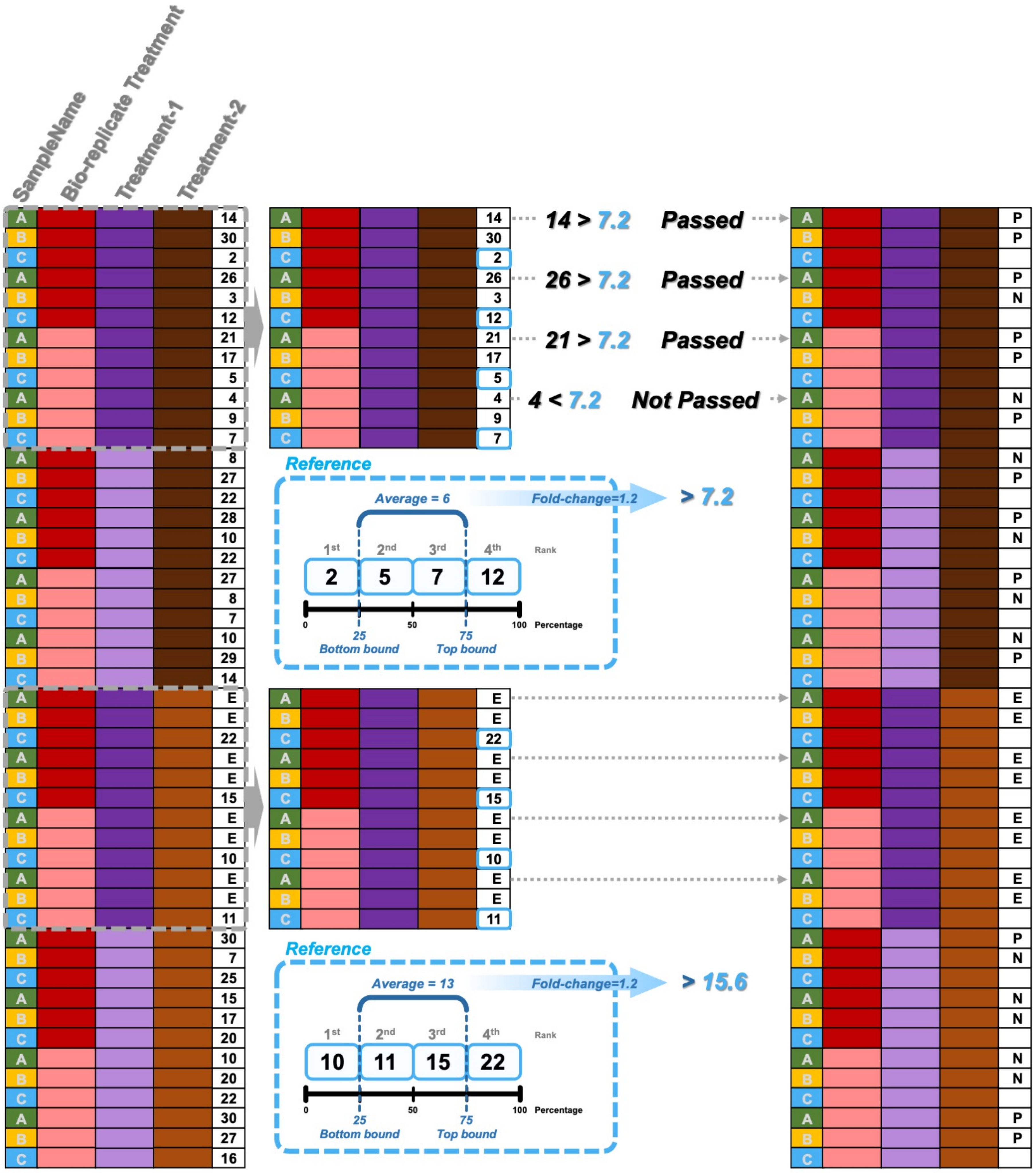


**Figure E1. Reference cutoff (manual setting option) – (1)**

Reference cutoff compares each signal to the reference cutoff value. This figure explained the manual setting option of reference cutoff. "SampleName" C was selected as the reference (in blue cell). The signals with the same "Condition" of "Treatment" were classified into one group, and each group had its own reference cutoff value. Take dark purple-dark brown group for example, the signals selected as reference from "SampleName" C were ranked. The signals in the range of bottom bound to top bound were averaged and multiplied by the fold-change value as 1.2, resulting in the reference cutoff value as 7.2 (blue dashed line frame). Reference cutoff was then set as 7.2. Take some signals for example, the A-red-dark purple-dark brown passed the cutoff, and obtained a P (positive) result. The A-pink-dark purple-dark brown did not pass the cutoff, so obtained a N result. The signals selected as reference, the signals with "SampleName" C, would not be analyzed by reference cutoff. Those signals already as an E result after internal control cutoff would remain as E.

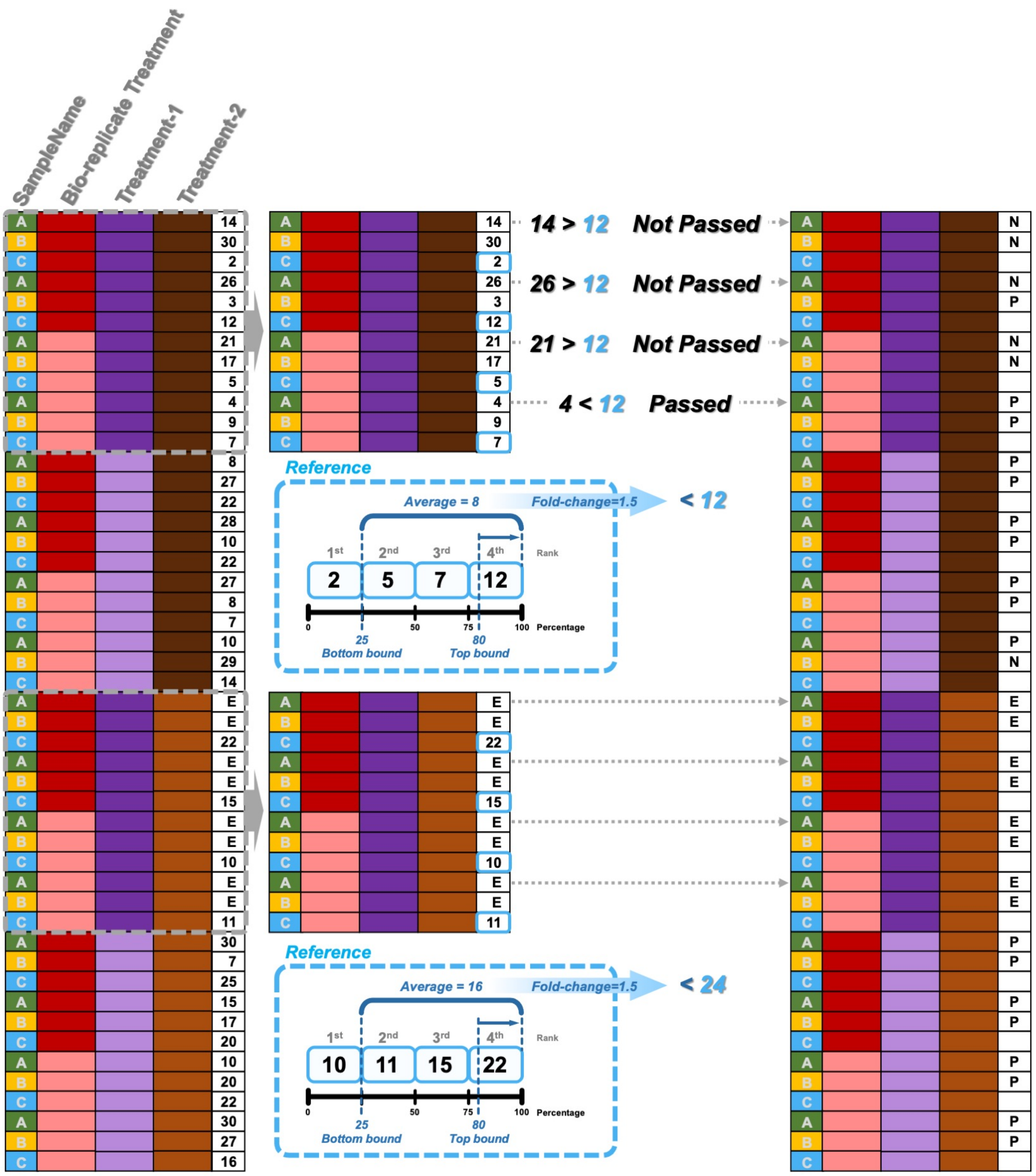


**Figure E2. Reference cutoff (manual setting option) – (2)**

This figure showed another example to give more details about the calculation of reference cutoff value in the manual setting option. If the top bound or bottom bound falls into the range of a signal, then the signal was also involved in the calculation of cutoff value. In the upper part of this figure, the top bound was 80, falling on the range of signal 12, and the bottom bound was 25, exactly cutting on the edge between signal 2 and 5. The signal 5, 7 and 12 were selected, averaged and multiplied by fold-change value 1.5, resulting in the reference cutoff value as 12 (blue dashed line frame).

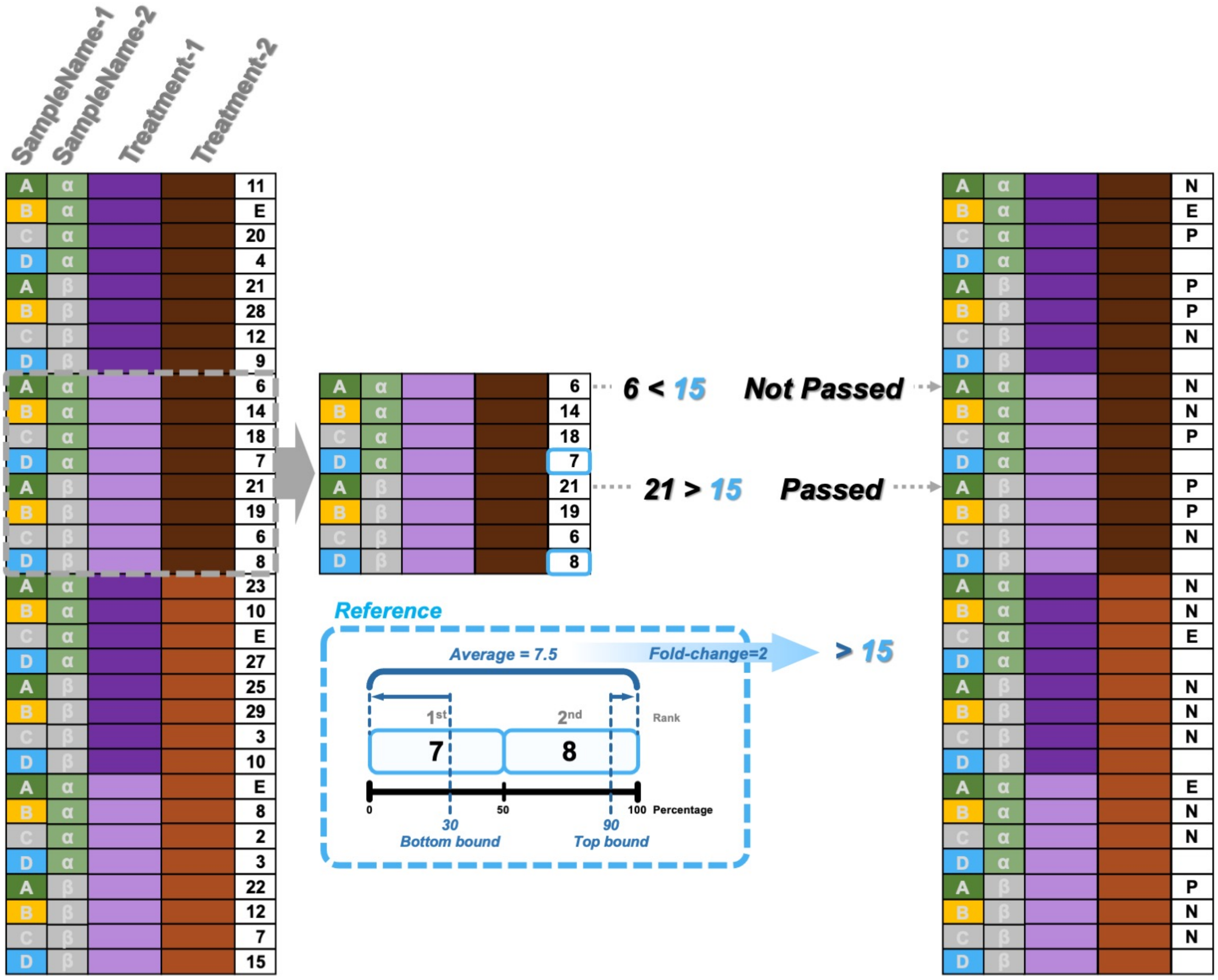


**Figure E3. Reference cutoff (manual setting with multiple SampleName columns) – (1)**

The figure provided more details about the selection of the "SampleName" of reference. When an input file contained multiple "SampleName" columns, the users can only select a "SampleName" in one "SampleName" column, such as D in SampleName-1 (light blue cells), and then the signals with D on SampleName-1 were selected to be processed for the reference cutoff value (light blue frame).

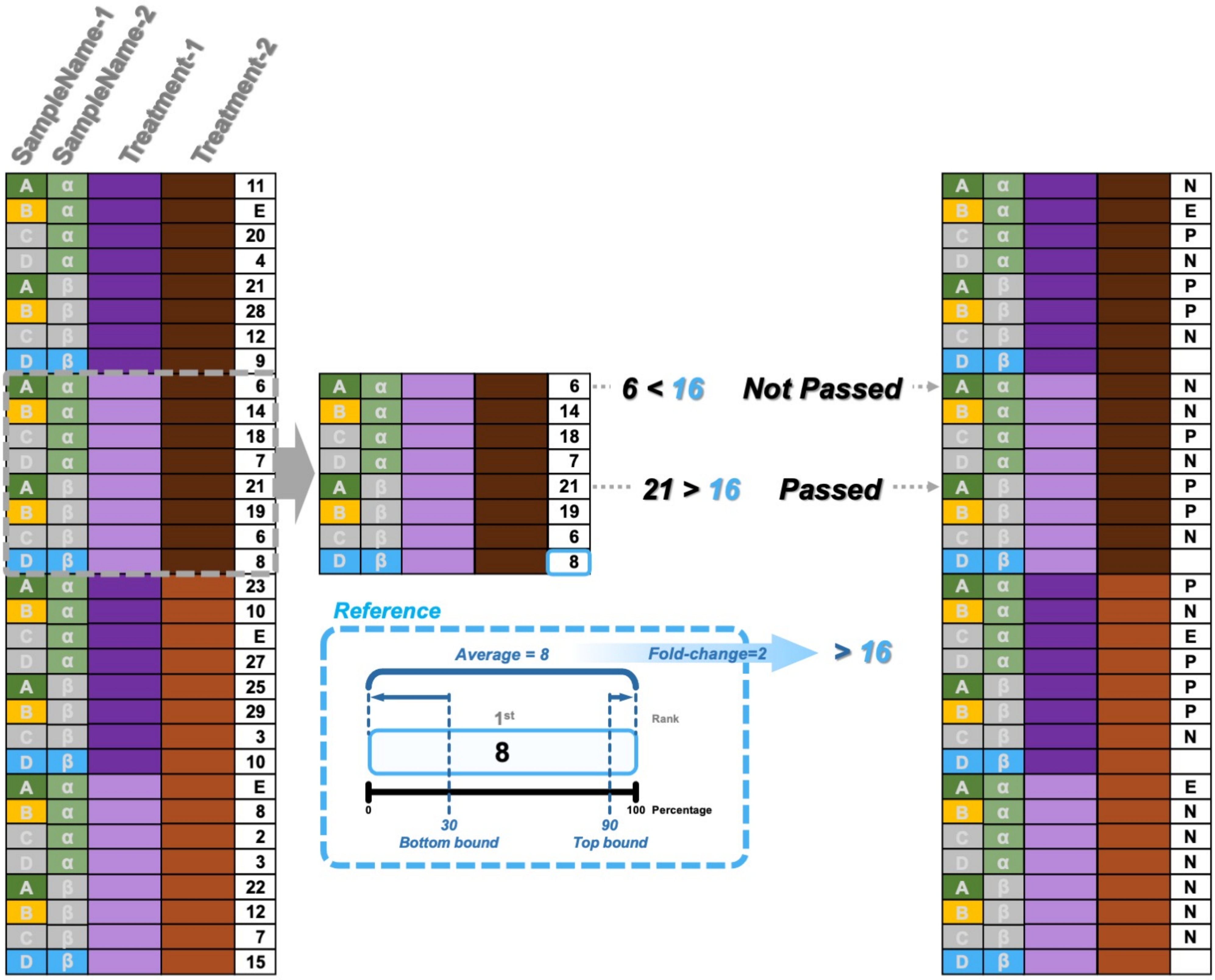


**Figure E4. Reference cutoff (manual setting with multiple SampleName columns) – (2)**

The users can also select one "SampleName" from each "SampleName" column. In this figure, the D in SampleName-1 and the β in SampleName-2 were both selected as reference. During reference cutoff analysis, only the signal 8 with both SampleName D and SampleName β would be used as reference.

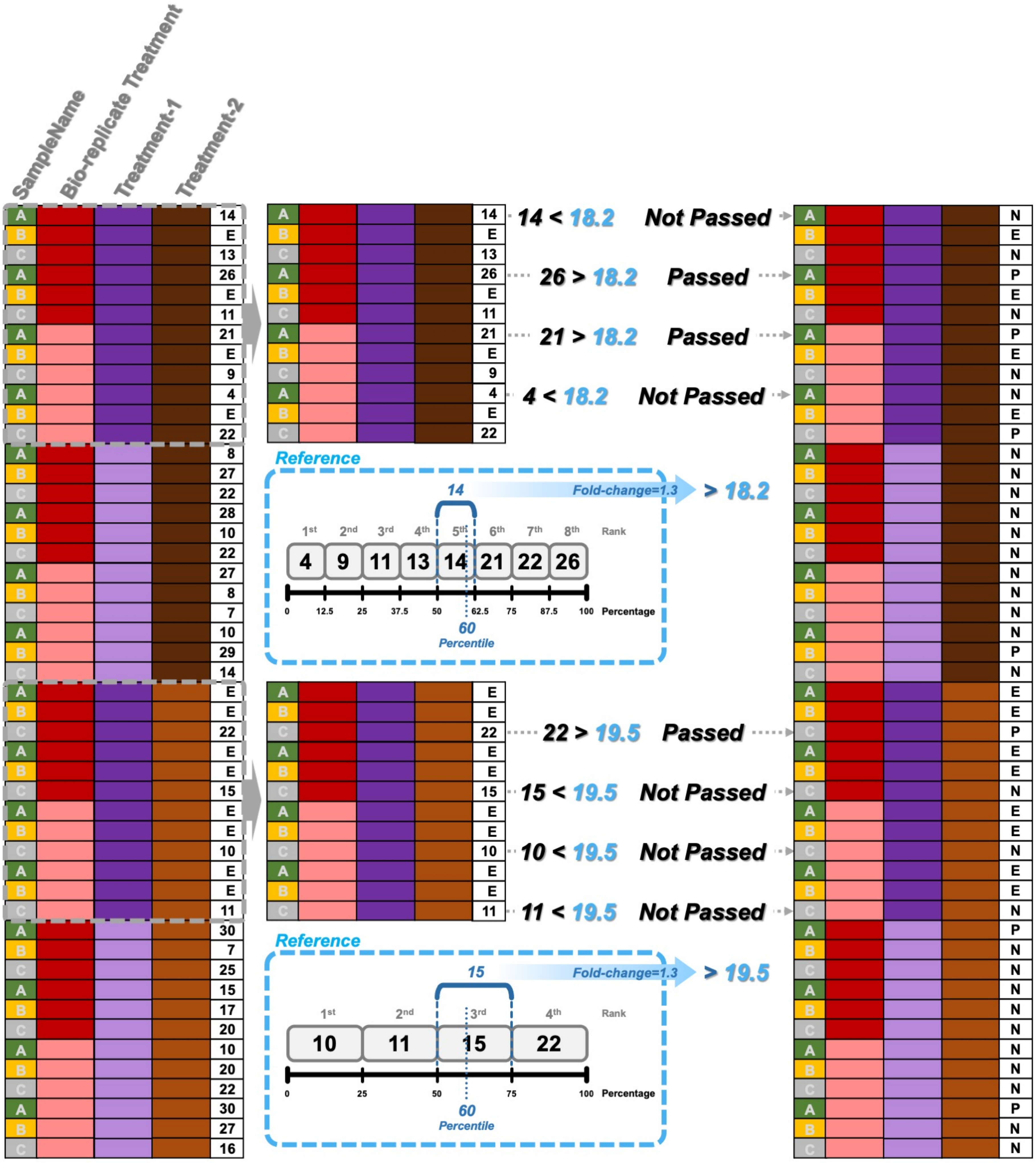

**Figure F1. Reference cutoff (percentile setting option) – (1)**

The figure showed the processing of reference cutoff value using the percentile setting option. All signals in one group except the signals with E results were ranked based on their values. Take the dark purple-dark brown group for example, the 60 percentile falls into the range of signal 14, so 14 was selected and multiplied with the fold change value 1.3, resulting in a reference cutoff value 18.2. Reference cutoff was then set as 18.2. If the signal value was higher than 18.2, then the result would be P. If the signal value was lower than or equal to 18.2, then the result would be N. The signals with E results would remain as E after reference cutoff analysis.

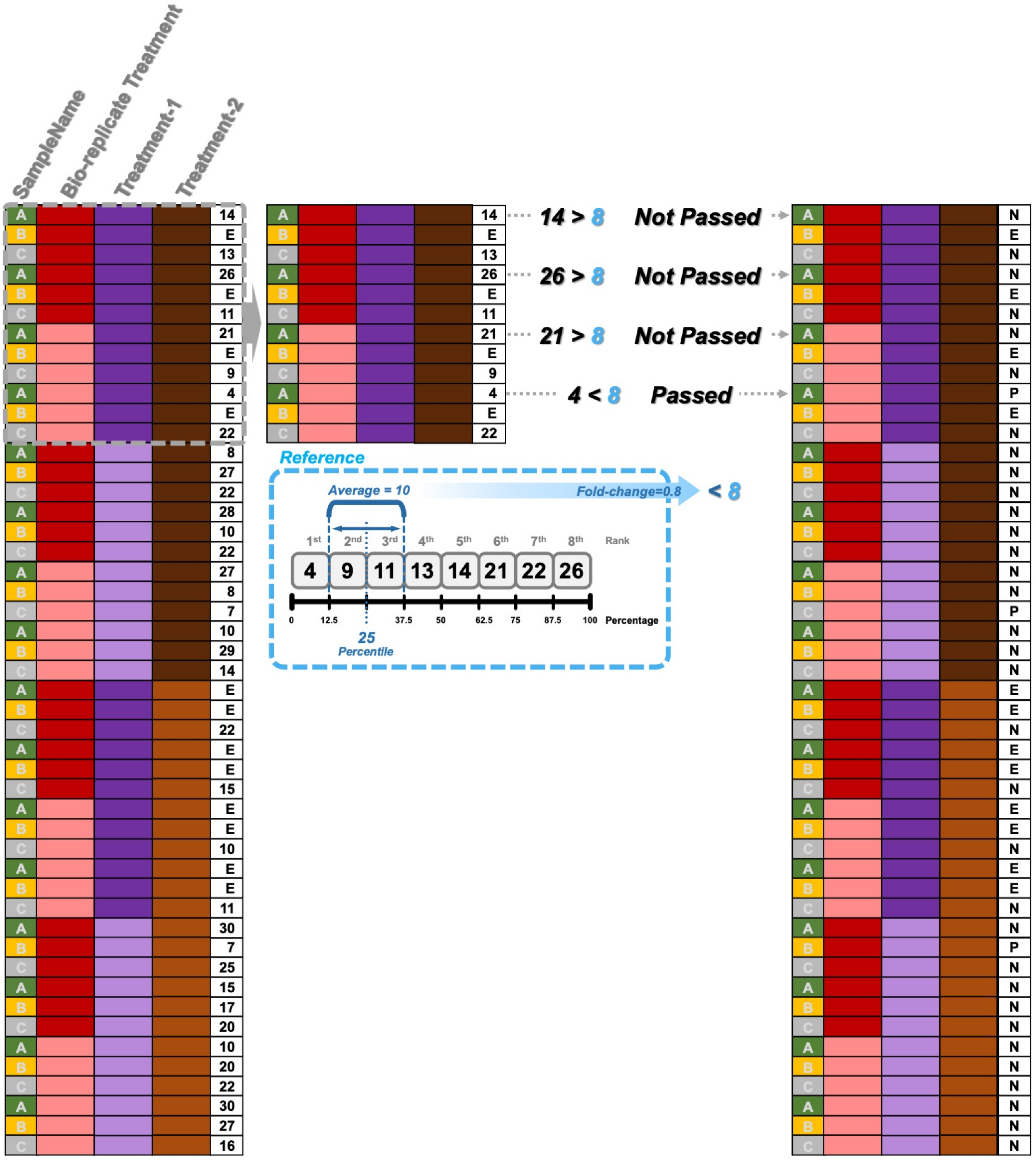


**Figure F2. Reference cutoff (percentile setting option) – (2)**

This figure provided additional details about the calculation of reference cutoff value in percentile setting option. If the percentile value located exactly on the edge of two signals, both signals would involve in the calculation of the reference cutoff value. In the figure, 25 percentile was between signal 9 and 11, and the average of the two signals was 10, which was further multiplied with the fold-change value 0.8, resulting in the reference cutoff value as 8.

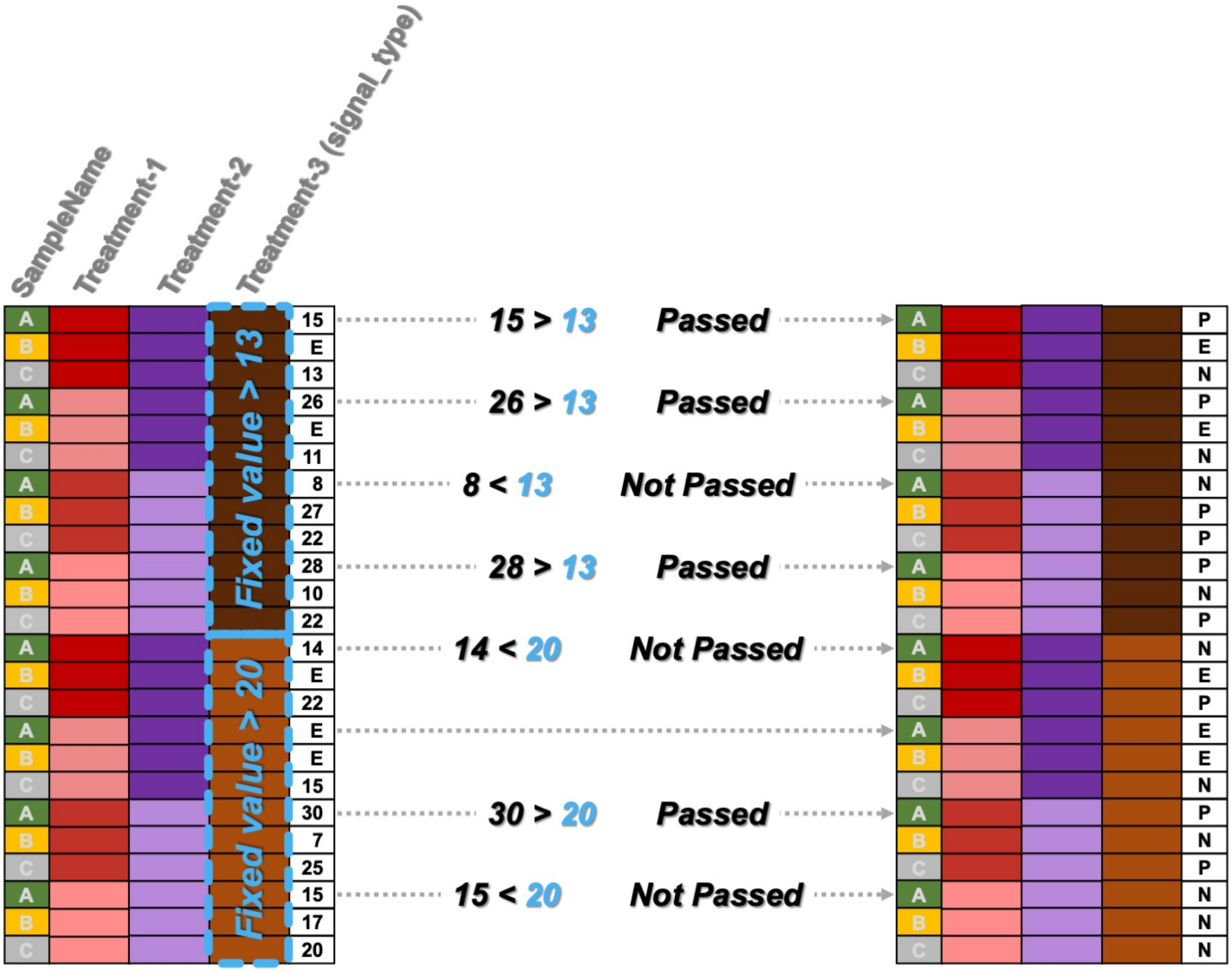


**Figure G. Reference cutoff (fixed value option)**

The fixed value setting option allowed the users to enter a fixed value as the reference cutoff value. If the input data contained more than one signal columns, as shown in Figure B4, an additional "Treatment" called "signal_type" would be generated. The users can set different values for each "Condition" of "signal_type" as the reference cutoff value. For example, the dark brown "Condition" of Treatment-3 was set as 13, and the light brown "Condition" of Treatmen-3 was set as 20. The signals with E results would remain as E after reference cutoff analysis.

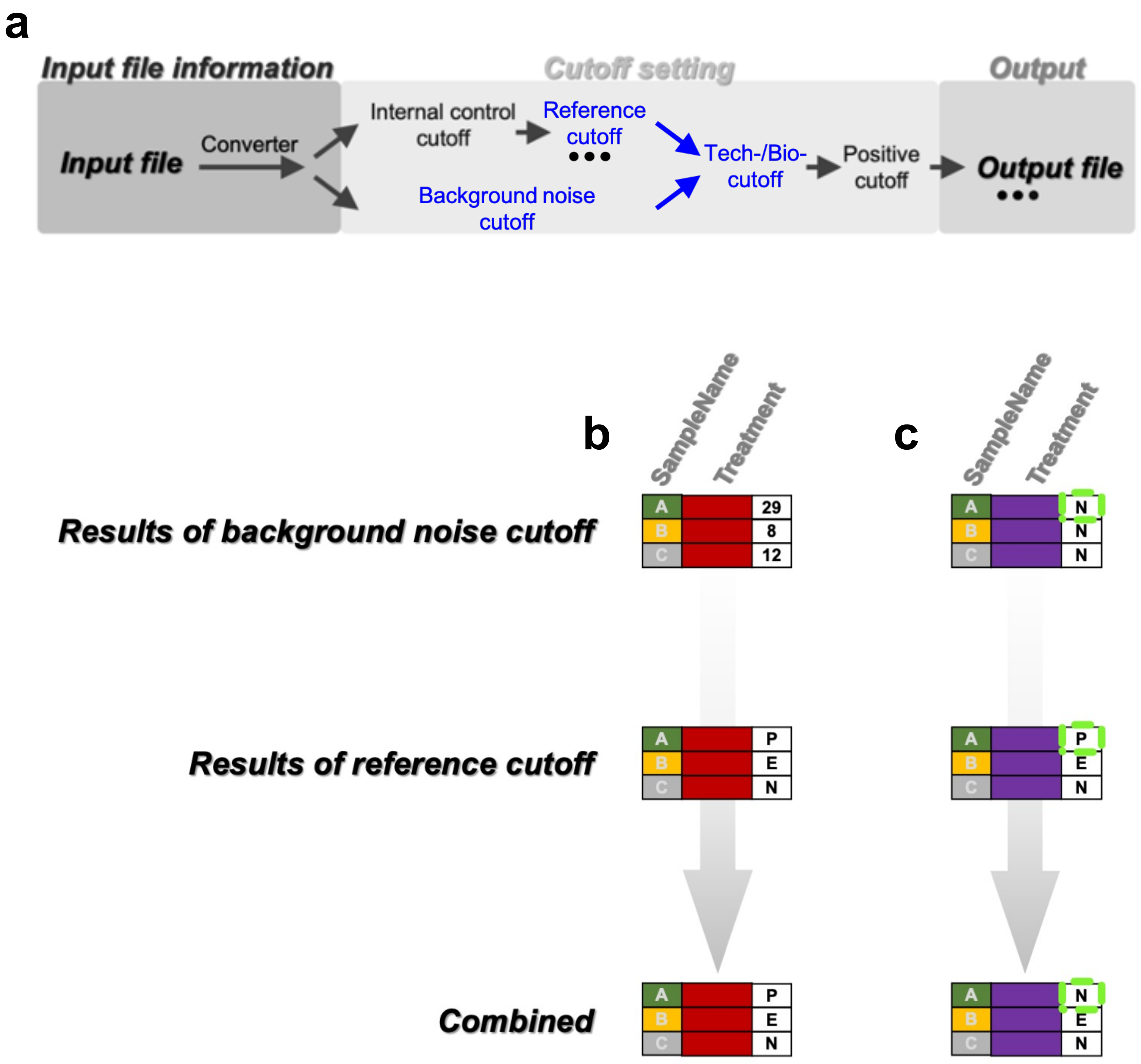


**Figure H. Combination of results after reference cutoff and background noise cutoff**

This figure described the (**a**) integration of the results after background noise cutoff and reference cutoff (in blue). **b** If the signals passed background noise cutoff, then the P/N/E of the reference cutoff results remained the same in combined results. **c** If not, then the P from the reference cutoff result became N in the combined results (light green dashed line frame), but E and N remained the same. This step was performed before bio-/tech-replicate cutoff.

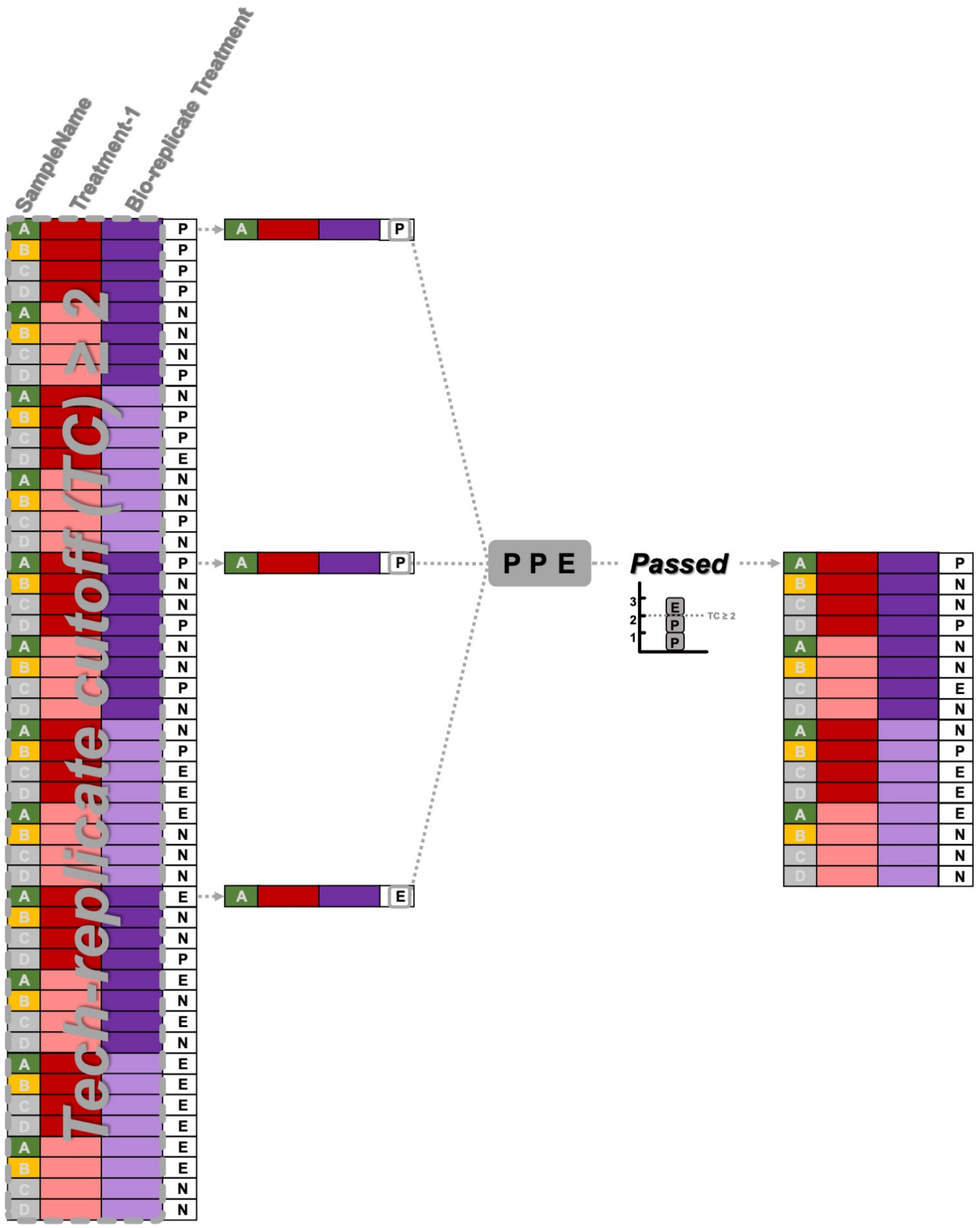


**Figure I1. Technical replicate cutoff – (1)**

The function of technical replicate cutoff worked on the P/N/E results. Technical replicate cutoff was set as 2. If the numbers of P were higher than or equal to 2, then the result after technical replicate cutoff would still be P. If not, the next step would calculate the numbers of P and E. If the numbers of P plus E can pass the cutoff, then the result would be E. Instead, the result would be N. Take the A-red-dark purple sample for example, three technical replicates of A-red-dark purple were 2Ps and 1E, and the numbers of P were equal to the technical replicate cutoff value as 2. This A-red-dark purple sample passed technical replicate cutoff, so the result would be P. Tech-replicate cutoff is not an optional function and the default is 1.

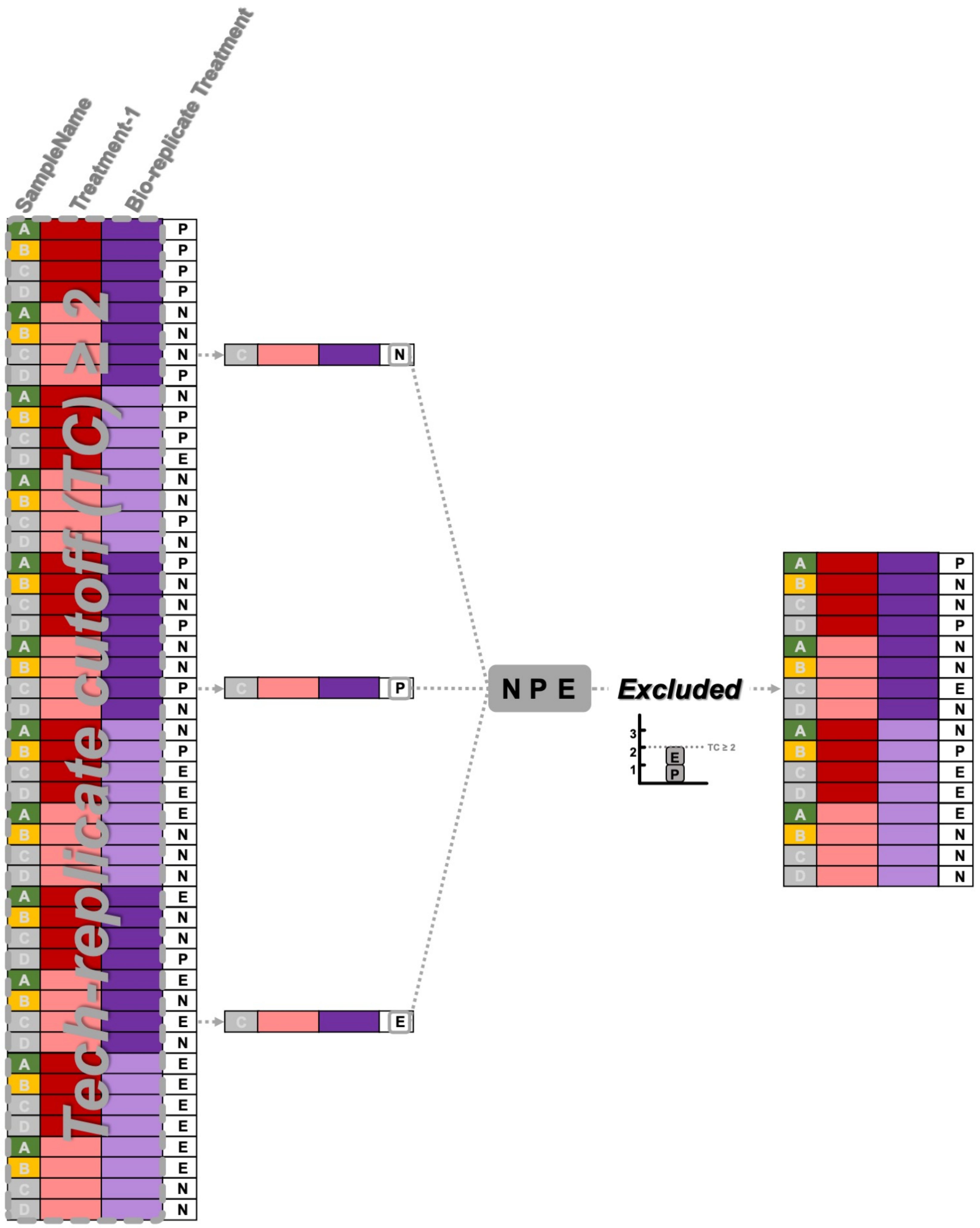


**Figure I2. Technical replicate cutoff – (2)**

Another example of technical replicate cutoff using the same cutoff setting as Figure I1. Three technical replicates of C-pink-dark purple sample were 1N, 1P and 1E. The number of P was lower than the cutoff value, so the next step generated the number of P and E as 2. The number 2 could pass the cutoff, so the C-pink-dark purple sample obtained an E result.

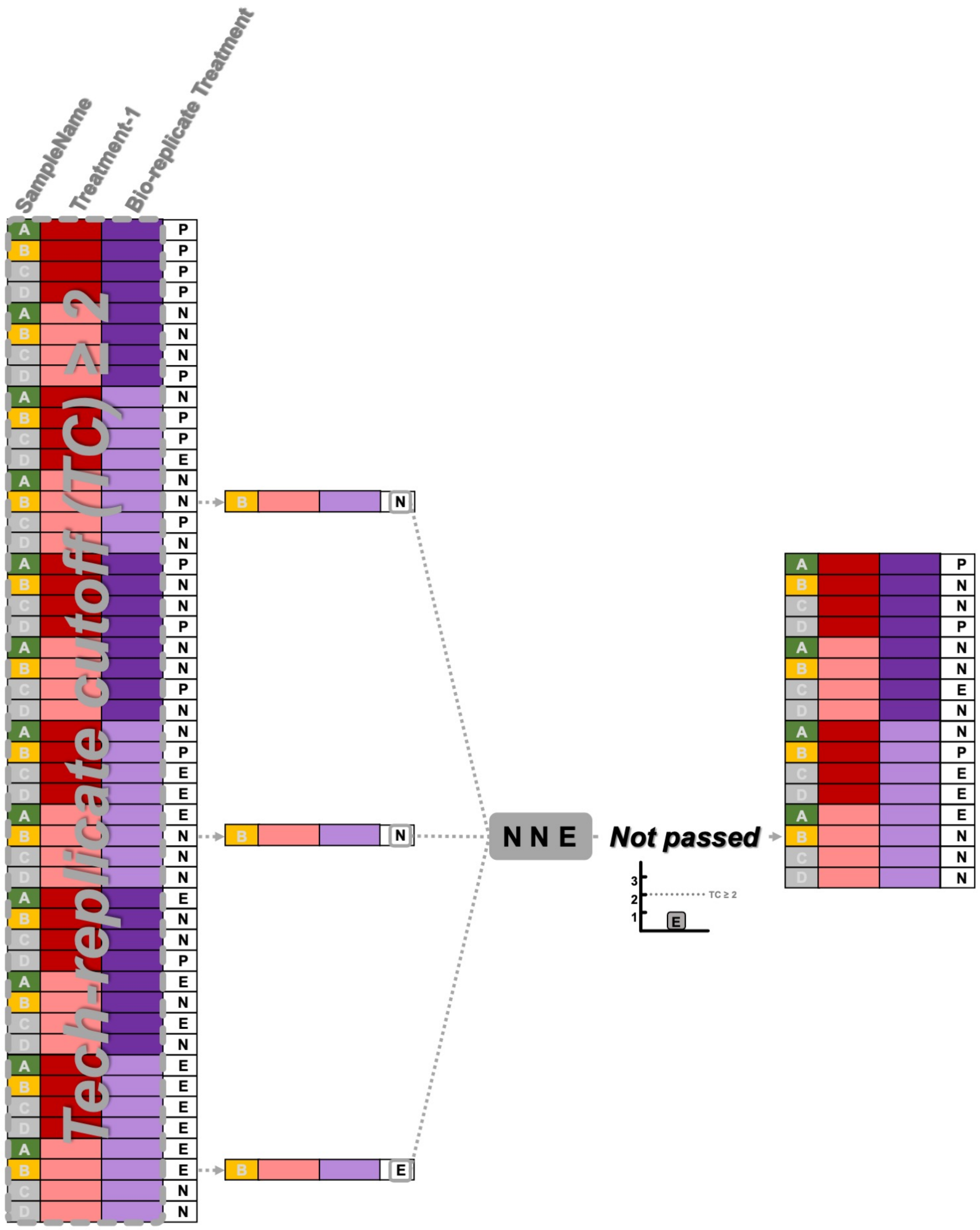


**Figure I3. Technical replicate cutoff – (3)**

Another example of technical replicate cutoff under the same cutoff setting as Figure I1. Three technical replicates of the B-pink-light purple sample were 2Ns and 1E. The number of P was lower than the cutoff value, and the number of P plus E was also lower than the cutoff value. The B-pink-light purple then obtained a N as the result.

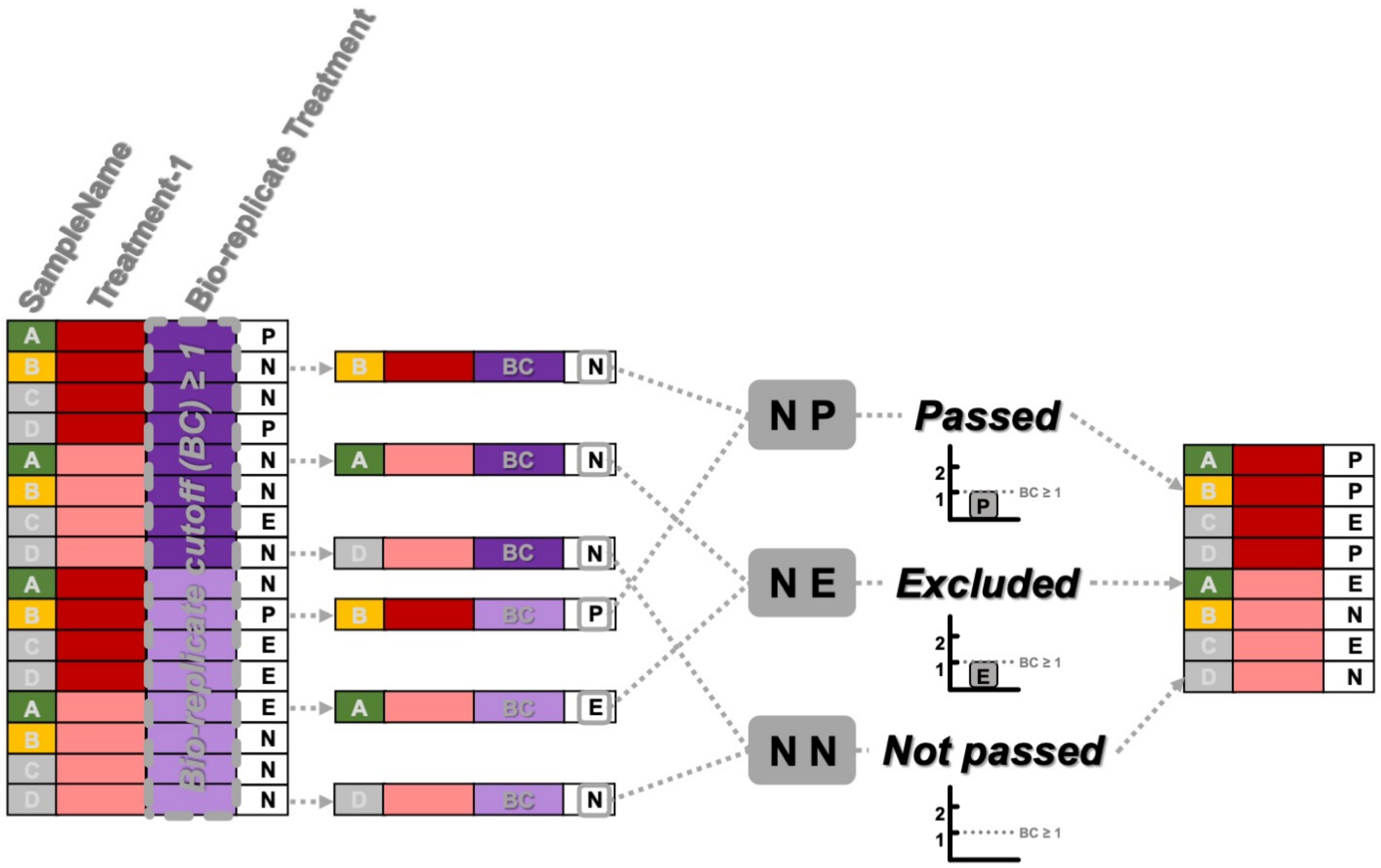


**Figure J. Biological replicate cutoff**

Biological replicate cutoff was executed after technical replicate cutoff. The analysis of biological replicate cutoff was similar to that of technical replicate cutoff (shown in Figure I) using an additional “Condition” of “Bio-replicate Treatment”. For example, the B-red-dark purple and the B-red-light purple samples were the two biological replicates of the B-red group. After the analysis, the B-red samples obtained the P result. Once the bio-replicate column was selected, biological replicate cutoff would become a required function, and the default is 1.

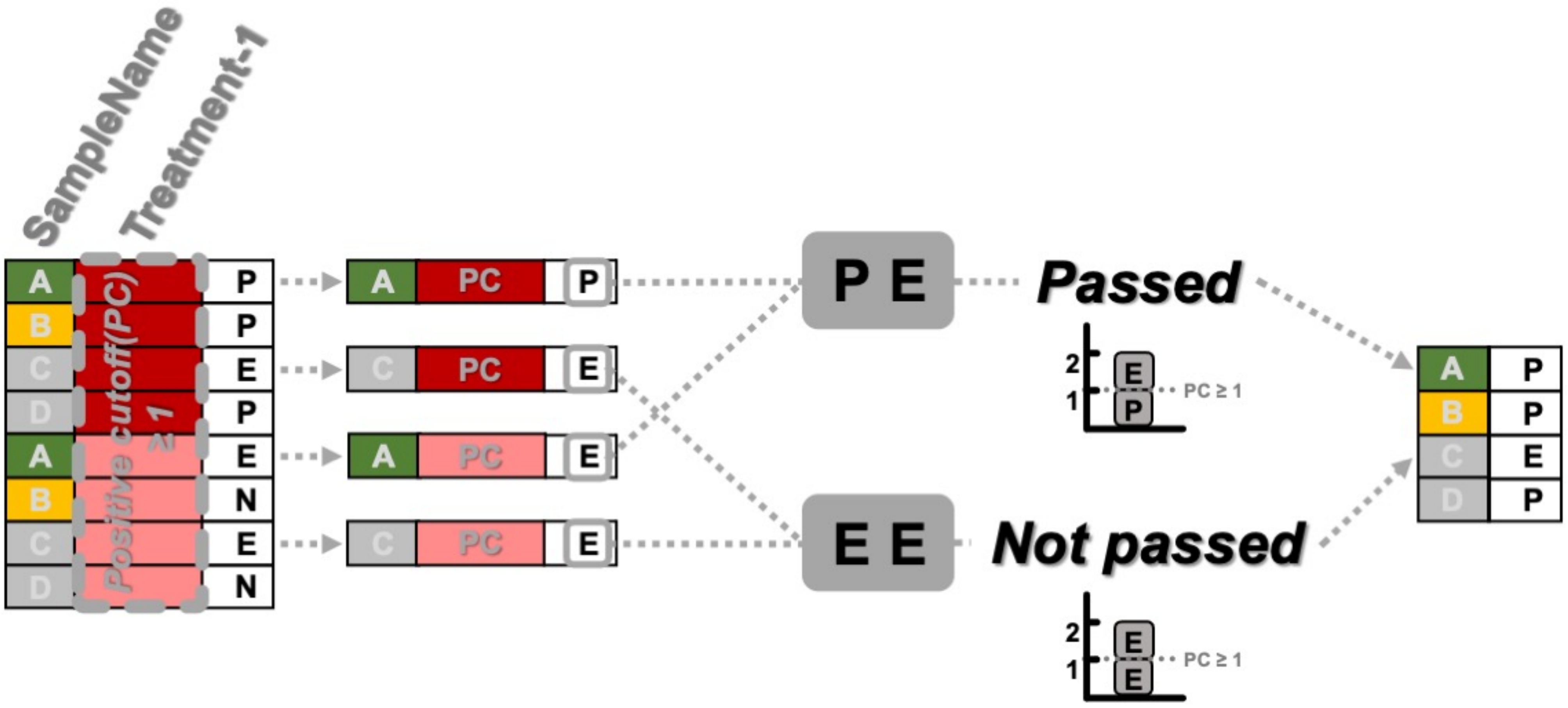


**Figure K1. Positive cutoff**

Positive cutoff was executed after biological replicate cutoff. The analysis of positive cutoff was similar to technical replicate cutoff (shown in Figure I), but positive cutoff worked on the "Treatment" selected by the users. In this figure, positive cutoff of Treatment-1 was set as 1. For example, the numbers of the P in the A group (A-red and A-pink) passed the cutoff, so the A samples obtained a P as the result. Positive cutoff is an optional function.

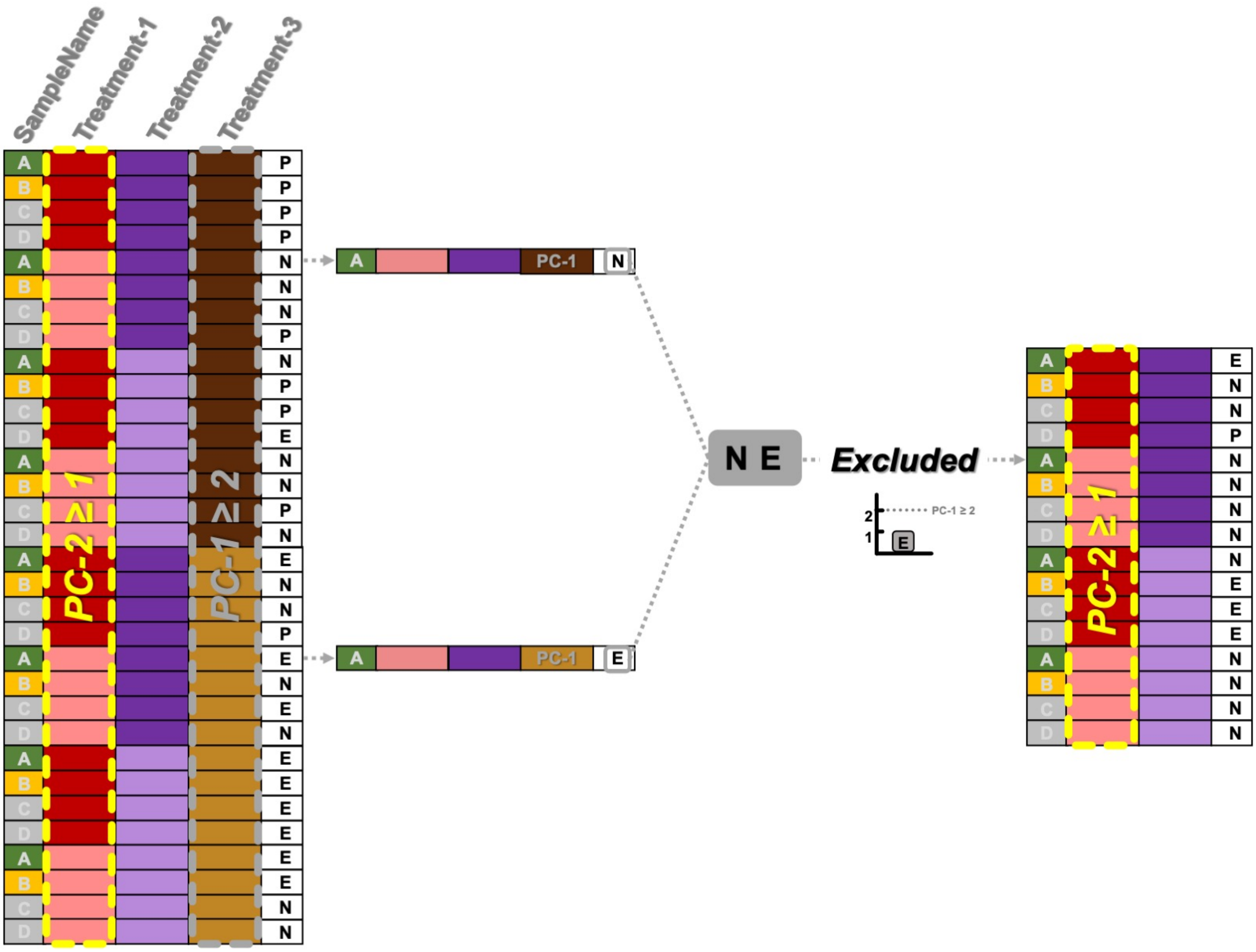


**Figure K2. Positive cutoff (multiple) – (1)**

The users can set multiple positive cutoffs. In this figure, Treatment-3 was selected as the first positive cutoff as 2, and Treament-1 was selected as the second positive cutoff as 1. During the first round of positive cutoff, the signals with different “Condition” of Treatment-3 were clustered and analyzed. The P/N/E numbers of A-pink-dark purple-dark brown and A-pink-dark purple-light brown obtained an E as the result. See the next figure for the second round analysis.

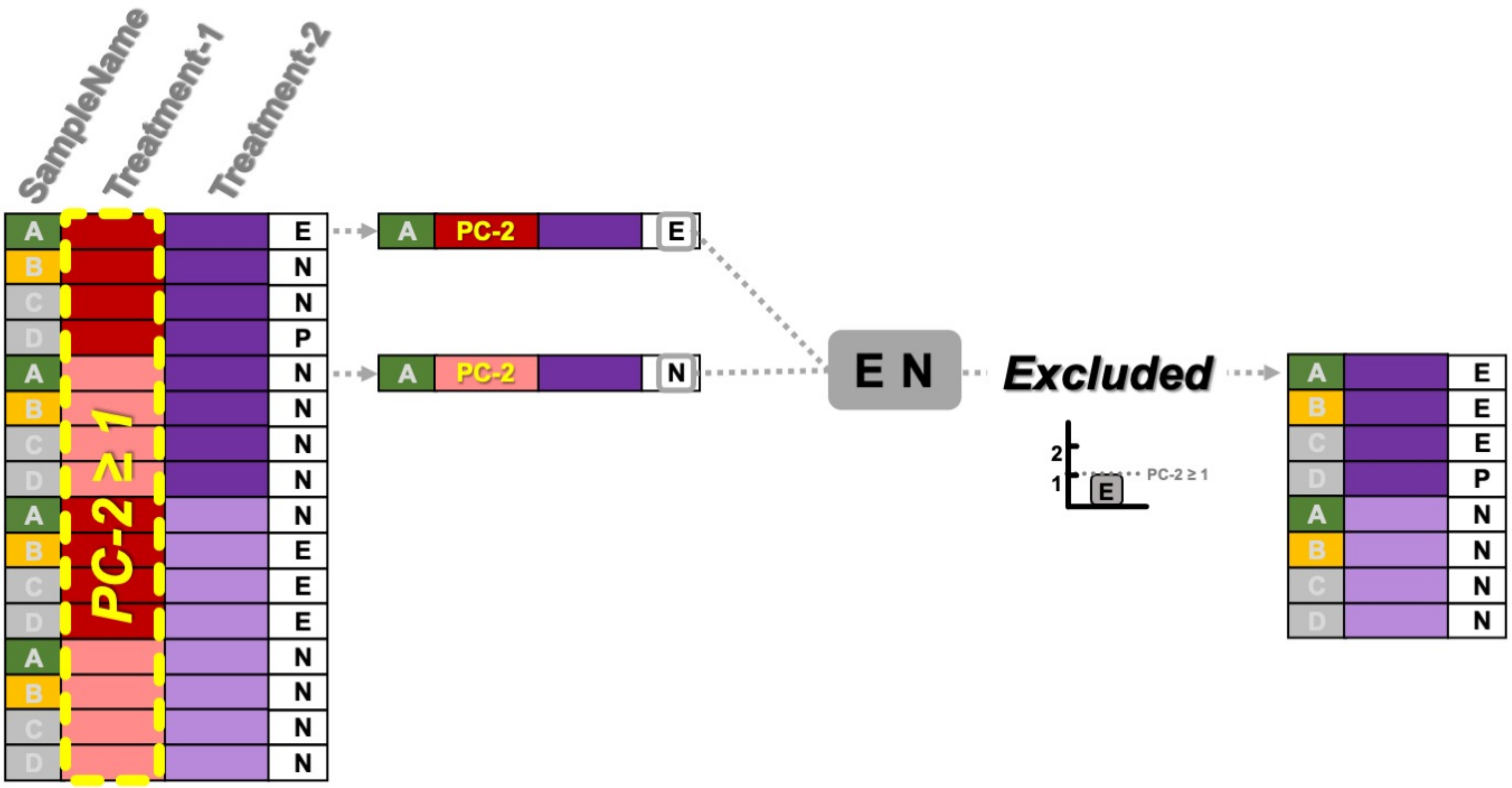


**Figure K3. Positive cutoff (multiple) – (2)**

On the second round of positive cutoff operation, the signals with different “Condition” of Treatment-1 were clustered and analyzed. The P/N/E number of A-red-dark purple and A-pink-dark purple obtained an E result.

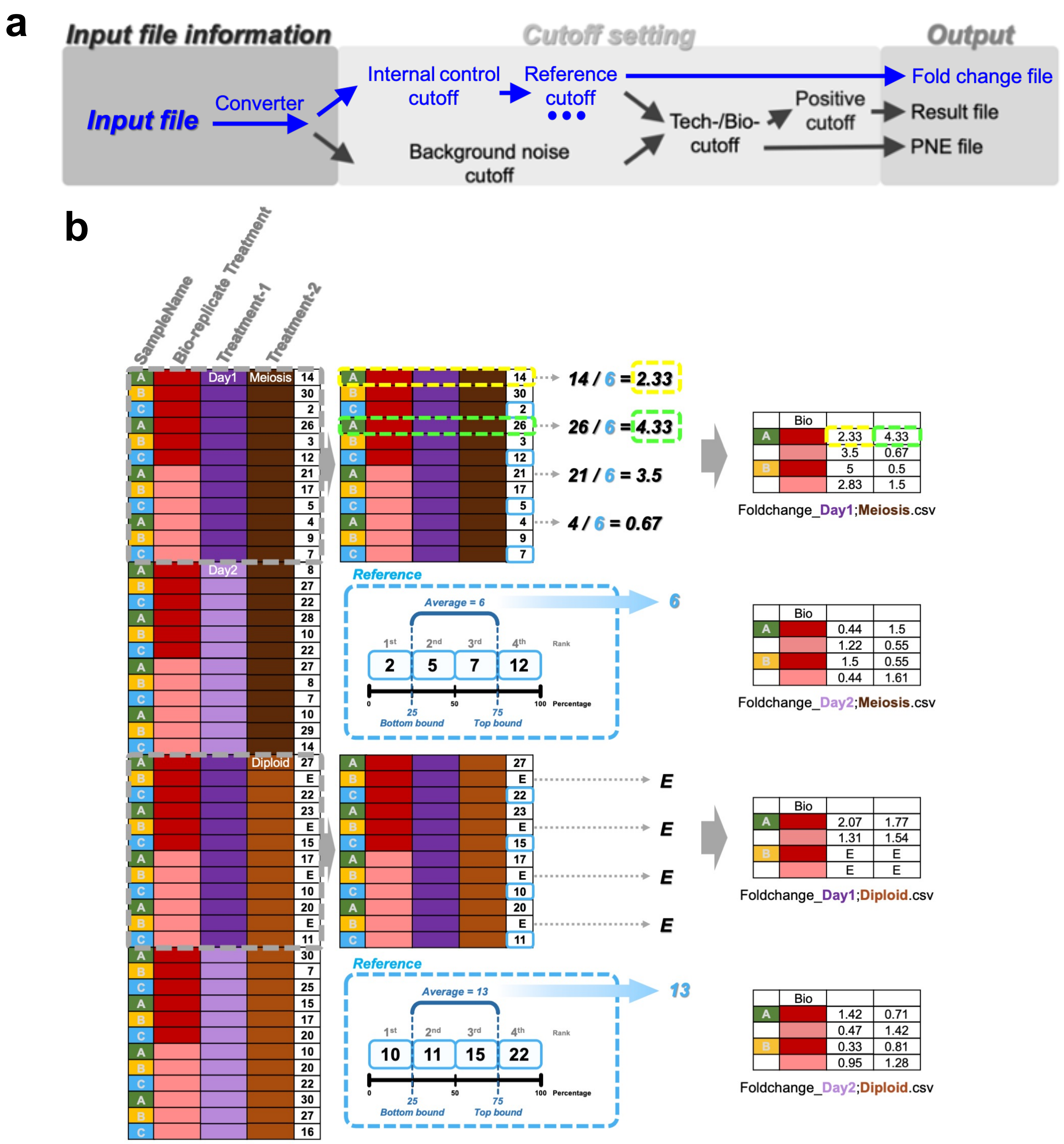


**Figure L1. Fold change file (manual setting option of reference cutoff)**

**a** After internal control cutoff, the samples would respectively be analyzed in two different ways using the reference value. One is for processing the “Fold change file” (in blue words). The other one is for outputting the “Result file” and “PNE file”. **b** The “Fold change file” contains the ratio results of the signals to the reference value, or the E results. The figure showed the processing for “Fold change file” using the manual setting option of reference cutoff. The calculation of the reference value is the same as Figure E without being multiplied by fold change value. Those signals already shown as an E result after internal control cutoff would remain as E in the “Fold change file”. The results with the same “Condition” of “Treatment” would be outputted in the same “Fold change file”. The file name would be the combination of “Condition” from different “Treatment”. For example, the results of Day1-Meiosis group (dark purple-dark brown group) samples would be outputted in the “Fold change file” named “Foldchange_Day1;Meiosis.csv”.

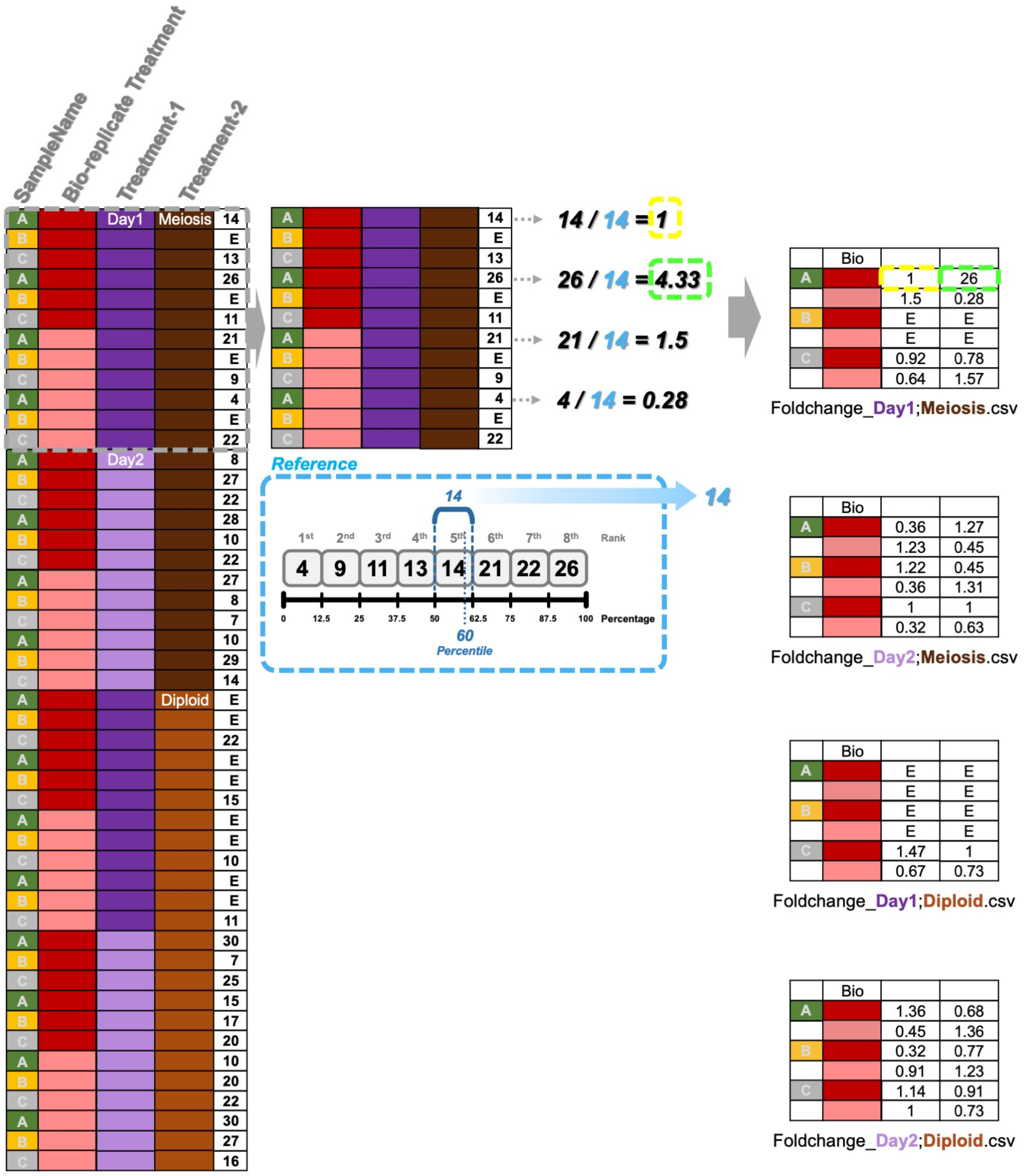


**Figure L2. Fold change file (percentile setting option of reference cutoff)**

The figure showed the processing for the “Fold change file” using the percentile setting option in reference cutoff. The calculation of reference value is the same as figure F without being multiplied by fold change value. The integration of results and the naming of files are the same as Figure L1.

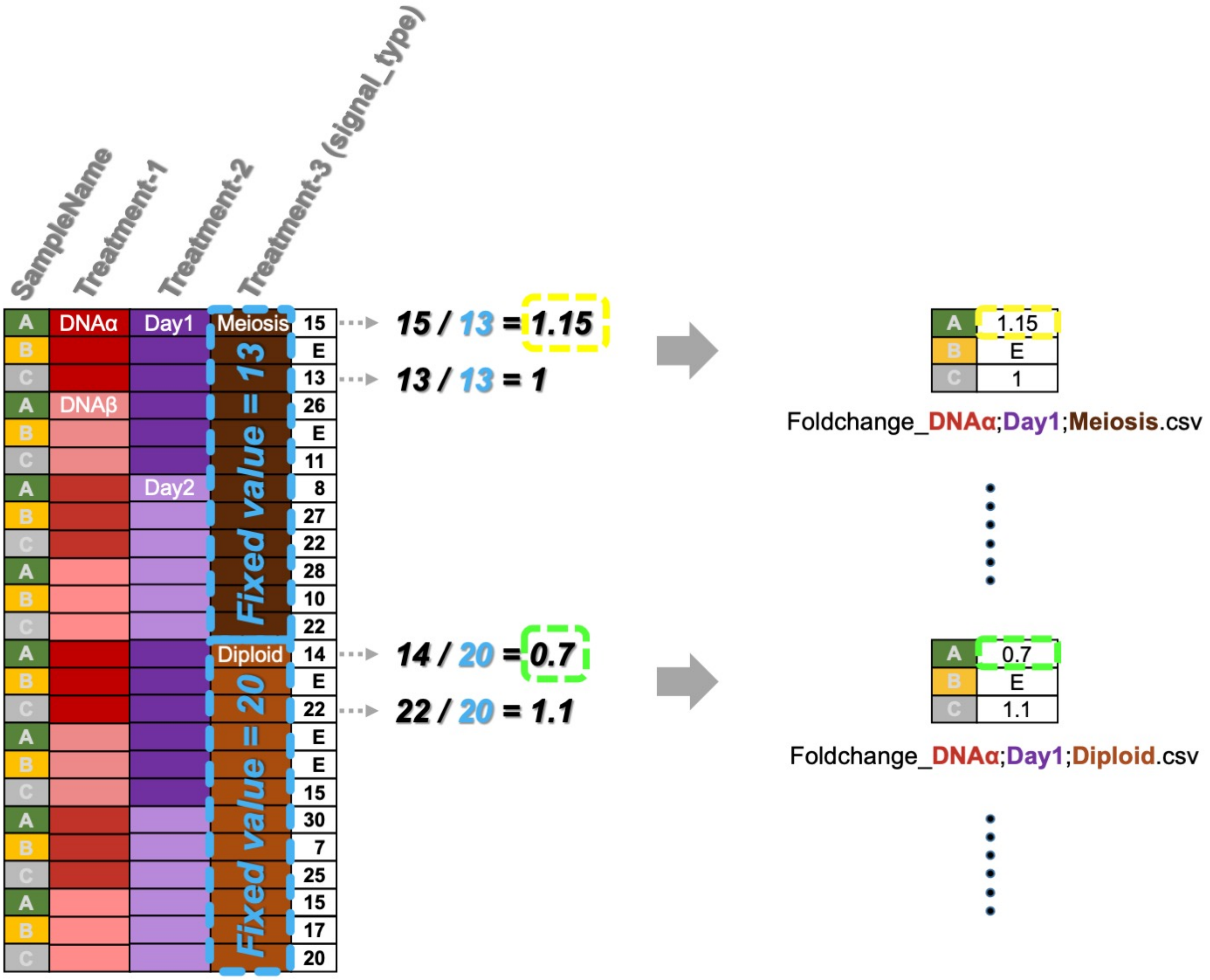


**Figure L3. Fold change file (fixed value option of reference cutoff)**

The figure showed the processing for the “Fold change file” using the fixed value setting option in reference cutoff. The reference value for the “Fold change file” is the same as reference cutoff value in Figure G. The integration of results and the naming of files are the same as Figure L1.

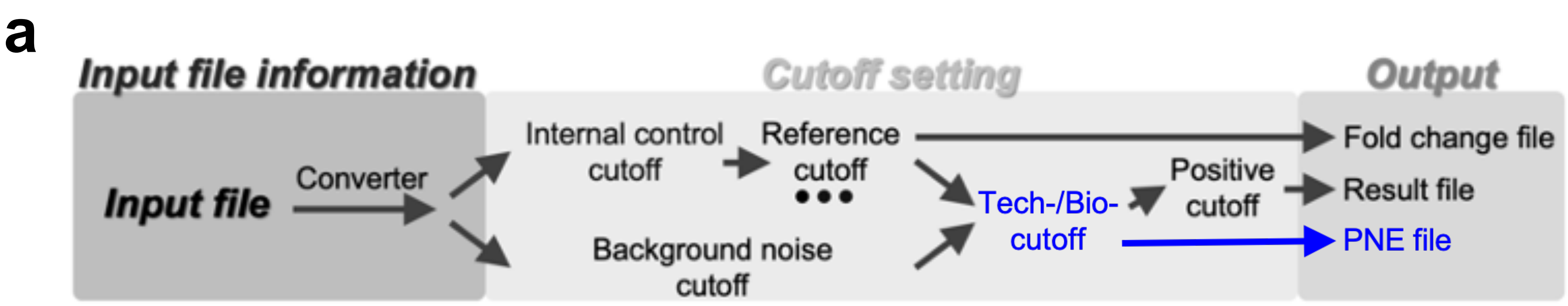


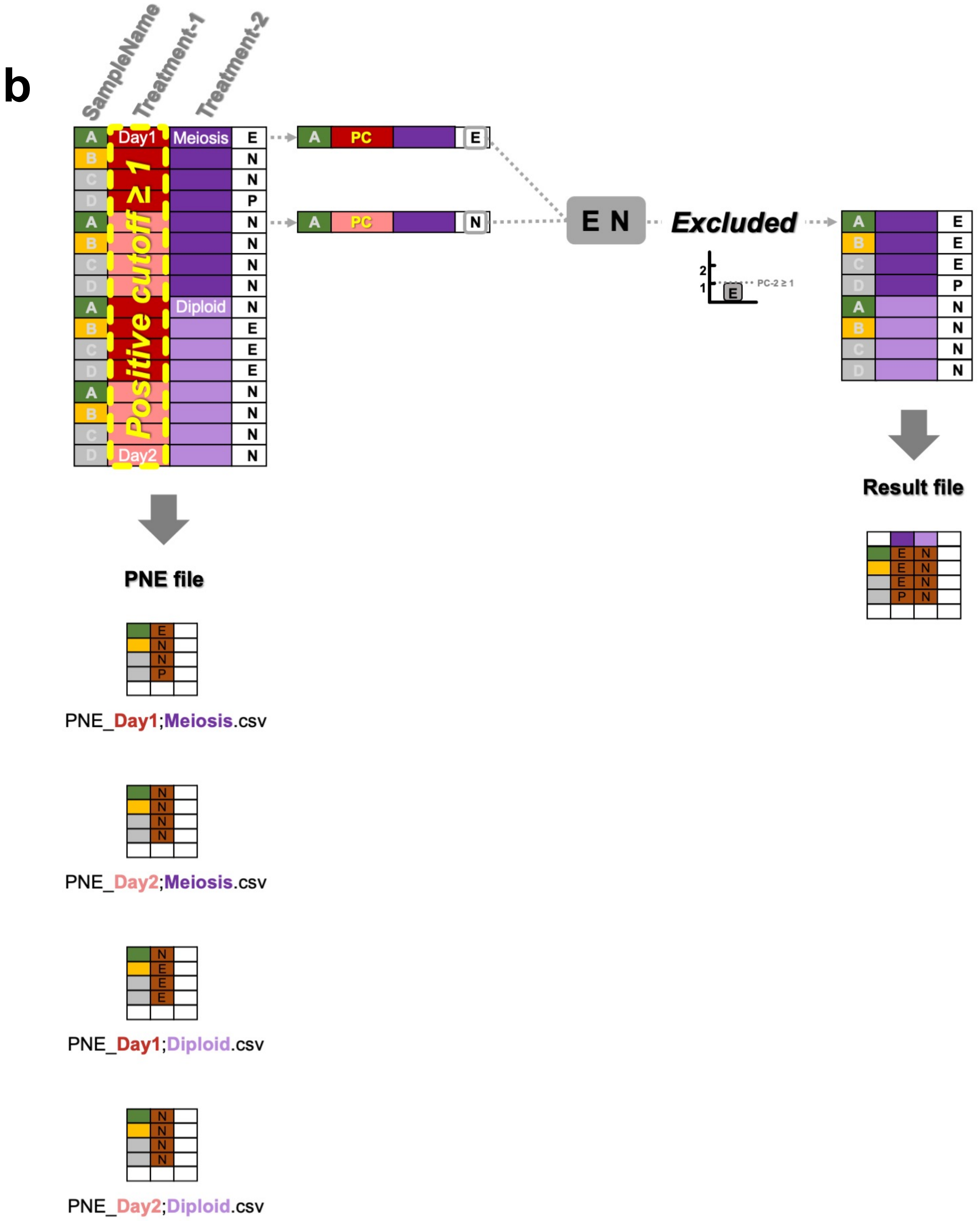


**Figure M. PNE file**

**a** The results in “PNE file” are the same as in “Result file” without being analyzed by positive cutoff (“Result file” was described in the main text). **b** The integration of the results and naming of files are the same as “Fold change file” in figure L-1.
