# Supplementary material for: Large-scale data analysis for robotic yeast one-hybrid platforms and multi-disciplinary studies using GateMultiplex
Source: BMC Biol. 2021 Sep 24;19:214. doi: 10.1186/s12915-021-01140-y (PMC8461970; doi:10.1186/s12915-021-01140-y)

***NOTICE: To fully understand this manual, please read through the main text first**

— Index —

Notes for using GateMultiplex 2

Symbols in output files 3

Download GateMultiplex files 4-5

Yeast one-hybrid (Y1H) for TF-DNA interaction (GM_Converter + GM_Basic) 6-23

Yeast one-hybrid (Y1H) for TF-DNA interaction (GM_Converter + GM_Advanced) 24-48

Warning of GateMultiplex 49

Warning of GM_Converter 50

Warning of GM_Basic 51-57

Warning of GM_Advanced 58-66

Notes for using GateMultiplex

- ❖ GateMultiplex is only compatible with Windows operation system.

- ❖ A reminder before using GateMultiplex, the maximum path length of Excel (usually as the default software to open CSV files) is 218 characters. To prevent unexpected issues, please avoid too long path length. To test GateMultiplex, we used eight computers with five different versions of Windows (Windows 10 Home x4, Windows 10 Education x1, Windows 10 Pro x1, Windows 10 Pro Education x1, Windows 7 Home Premium x1). One of the four computers with Windows 10 Home needed to close other programming software/interfaces before operating GateMultiplex, and another computer (Windows 7 Home Premium) needed to inactivate the anti-virus software to perform GateMultiplex. Overall, all eight computers could successfully operate GateMultiplex.

- ❖ The input files for GateMultiplex should be in csv format.

- ❖ The data of input files for GateMultiplex should not include comma "," and semicolon ";" (except the field separator commas for the csv file).

- ❖ The directory of input files for GateMultiplex should only contain English characters.

- ❖ SampleName files, Treatment files and List_for_Converter file need to be prepared and generated in Windows operation system.

- ❖ Only for GM_Basic and GM_Advanced: if "Fold change file" or "PNE file" options on the output file selection is enabled, the data in input files should not include any characters invalid for Windows filenames (listed in the following):

  - < (less than)
  - > (greater than)
  - : (colon)
  - " (double quote)
  - / (forward slash)
  - \ (backslash)
  - | (vertical bar or pipe)
  - ? (question mark)
  - * (asterisk)

Symbols in output files

❖ In result file (“Results.csv”)

- P: Positive
- N: Negative
- E: Excluded
- - : Not exist

	A	B	C	D	E	F	G	H	I	J	K
1		Diploid;AE	Diploid;AE	Diploid;AE	Diploid;AE	Diploid;AE	Diploid;AE	Diploid;AE	Diploid;AE	Diploid;AE	Diploi
2	TF#001	N	-	-	-	N	-	-	-	E	-
3	TF#002	P	-	-	-	N	-	-	-	E	-
4	TF#005	N	-	-	-	N	-	-	-	E	-
5	TF#006	P	-	-	-	N	-	-	-	E	-
6	TF#008	N	-	-	-	N	-	-	-	E	-
7	TF#012	-	-	-	N	-	-	-	N	-	-
8	TF#013	N	-	-	-	N	-	-	-	E	-
9	TF#014	N	-	-	-	N	-	-	-	E	-
10	TF#017	N	-	-	-	N	-	-	-	E	-
11	TF#018	N	-	-	-	N	-	-	-	E	-
12	TF#019	-	-	N	-	-	-	N	-	-	-
13	TF#022	-	-	N	-	-	-	N	-	-	-
14	TF#025				N				N		

❖ In fold change file

- nan (red frame) : zero divided by zero
- inf (blue frame): non-zero number divided by zero
- E: Excluded

	A	B	C	D	E	F	G	H
1		Bio						
2	TF#026	Bio1	nan	nan	nan	nan		
3		Bio2	nan	inf	nan	nan		
4		Bio3	nan	nan	nan	nan		
5		Bio4	nan	nan	nan	nan		
6	TF#031	Bio1	nan	nan	nan	nan		
7		Bio2	nan	nan	nan	nan		
8		Bio3	nan	nan	nan	nan		
9		Bio4	nan	nan	nan	nan		
10	TF#081	Bio1	nan	nan	nan	nan		
11		Bio2	nan	nan	nan	nan		
12		Bio3	nan	nan	nan	nan		

❖ PNE files use the same symbols as result file.

Download GateMultiplex files

- Download the files of GateMultiplex from GitHub

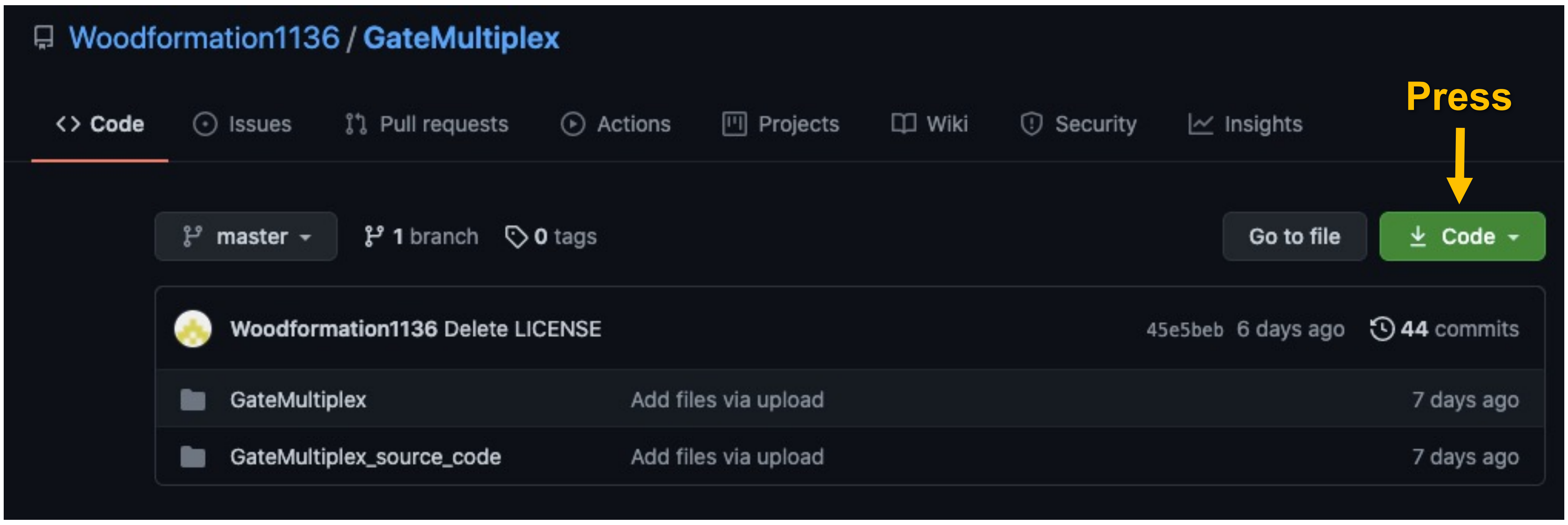

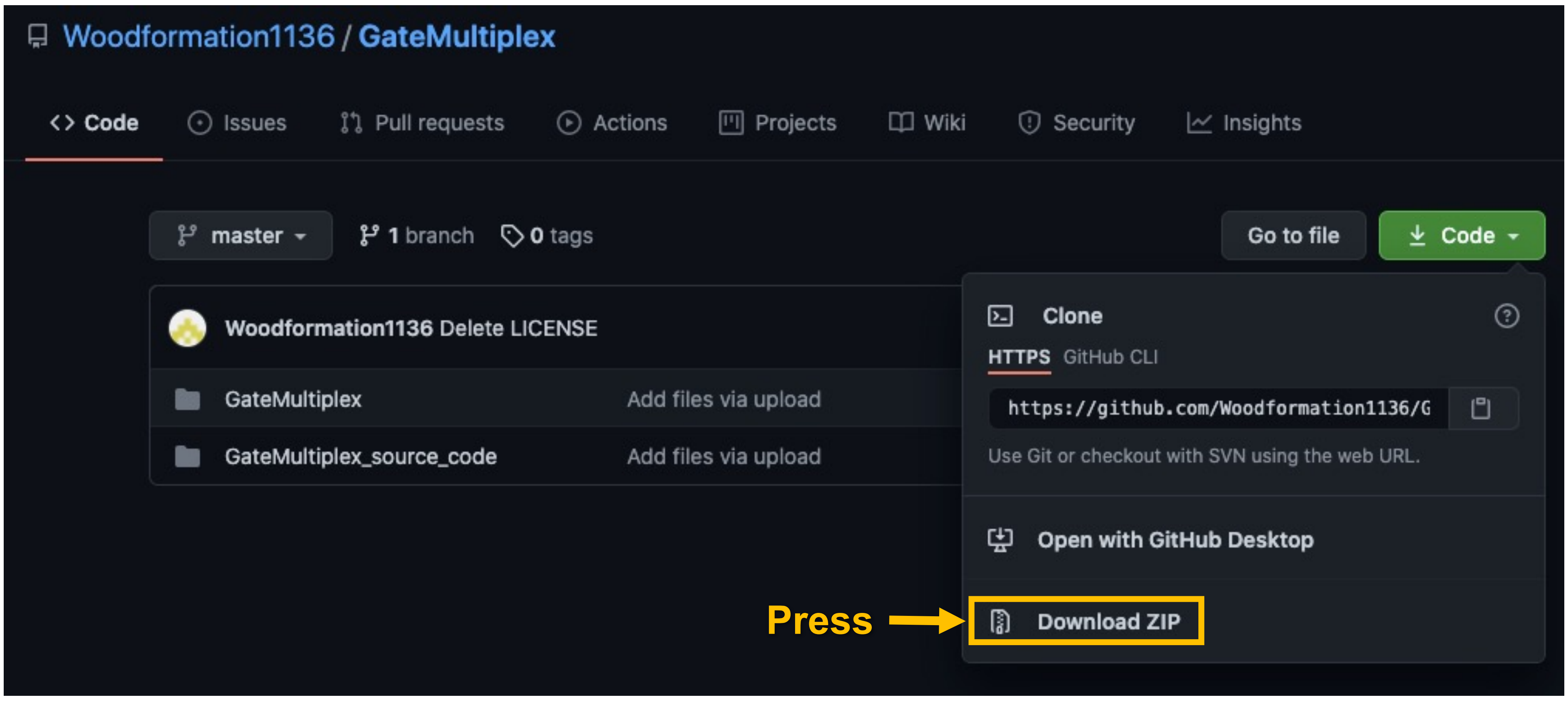

- Contents of downloaded GateMultiplex folder
- The files or folders downloaded from GitHub would be compressed into a zip file. This zip file would be automatically added with “-master” at the end of the file name. Therefore, our uploaded folders (“GateMultiplex” and “GateMultiplex_source_code”) will be downloaded into a zip file with the file name as “GateMultiplex-master”.

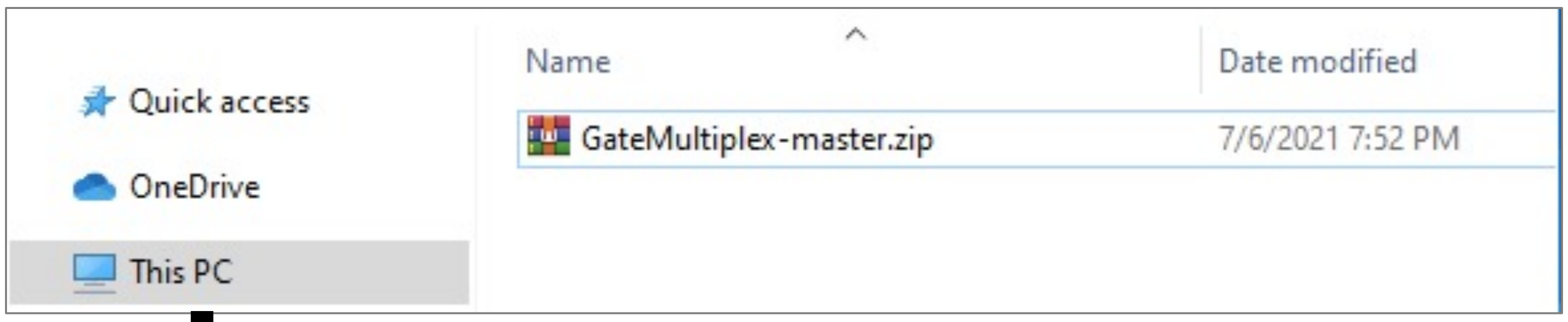

Decompress the Zipped file “GateMultiplex-master.zip”

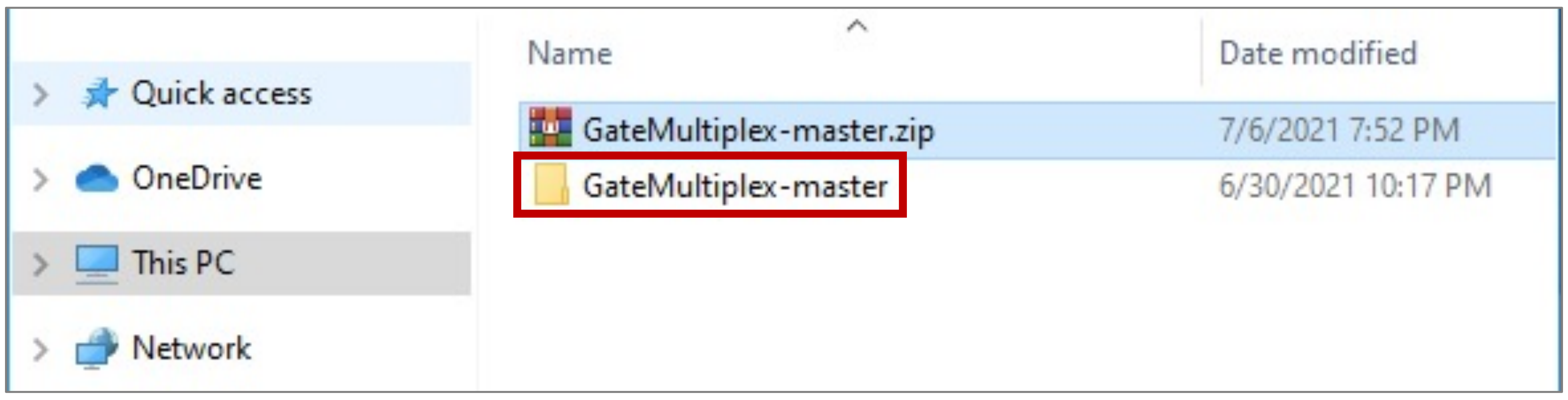

Open the folder “GateMultiplex-master” (red frame)

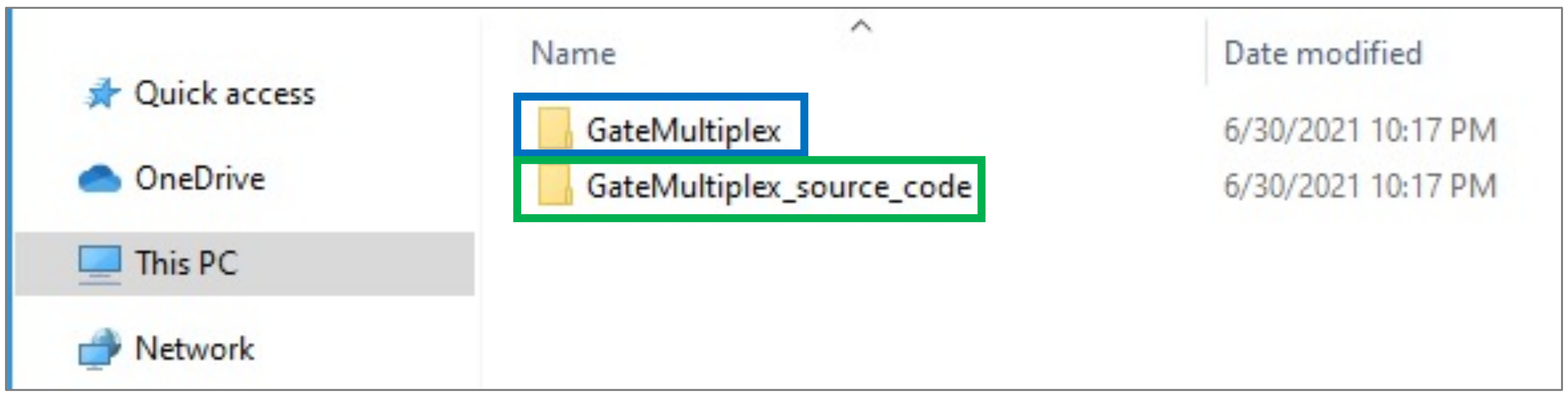

*The folder “GateMultiplex_source_code” (green frame) includes the source code of GateMultiplex.

Open the folder “GateMultiplex” (in blue frame)

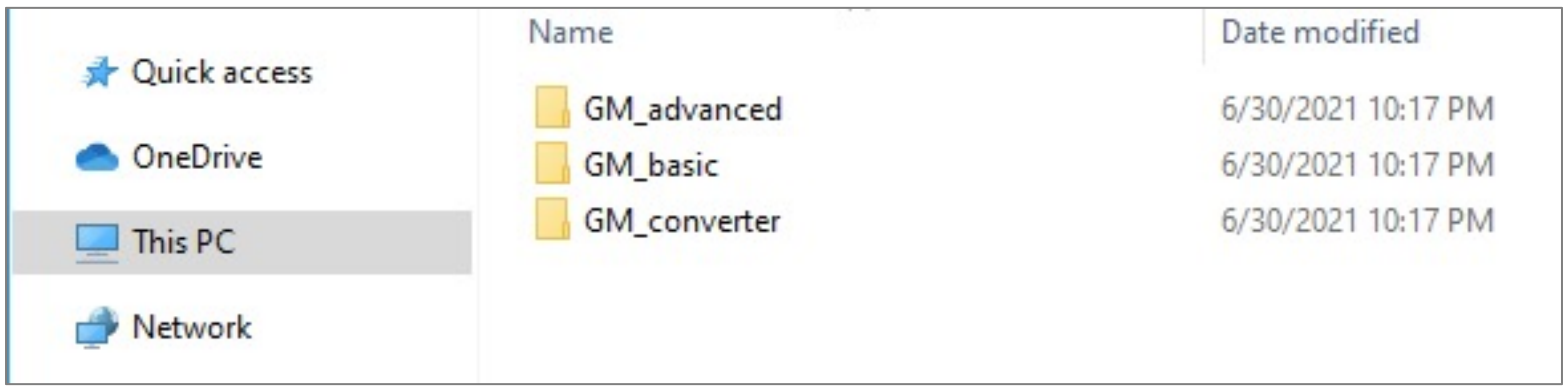

*The executive files of GM_Basic, GM_Advanced and GM_Converter are stored in the folder “GM_basic”, folder “GM_advanced” and “GM_converter”, respectively.

Yeast one-hybrid (Y1H) for TF-DNA interaction (GM_Converter + GM_Basic)

Yeast one-hybrid (Y1H) for TF-DNA interaction (GM_Converter + GM_Basic)

- *The provided demo data is from our previous study (PMID: 31186303, 2019 Genome Research). Due to the different version of PhenoBooth, the output values are different and thus the analyzed results might be different to the previous results.*

- The location of “Y1H_for GM_Basic_demo” data.

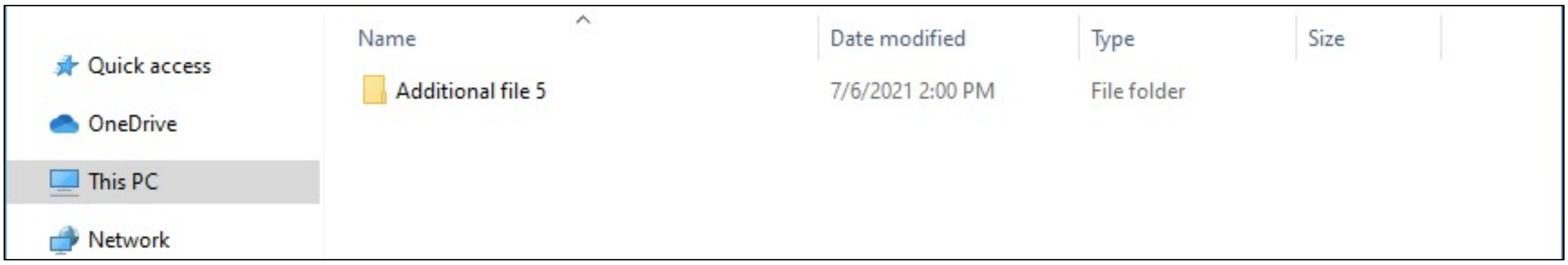

Open the “Additional file 5” folder

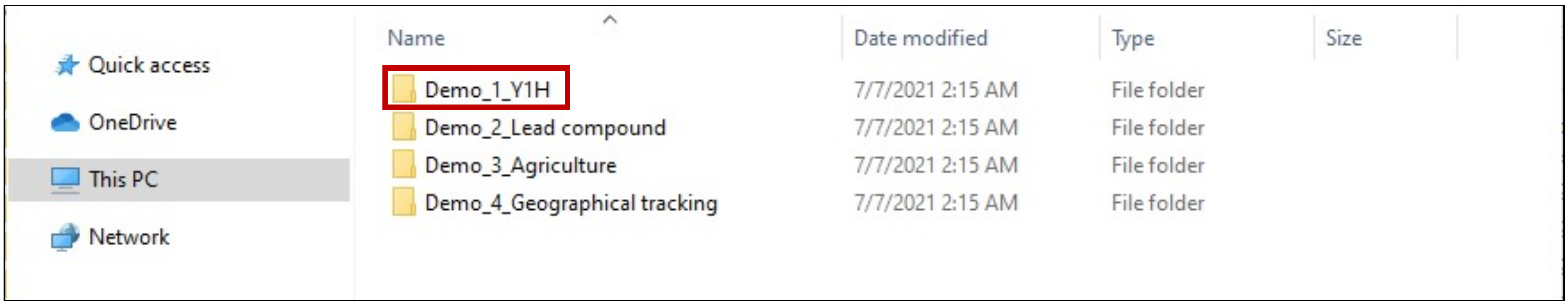

Open the “Demo_1_Y1H” folder (red frame)

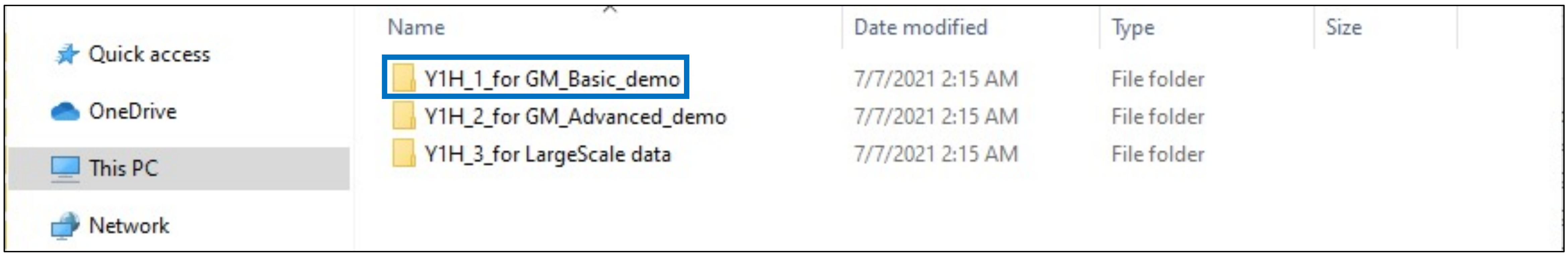

Open the “Y1H_1_for GM_Basic_demo” folder (blue frame)

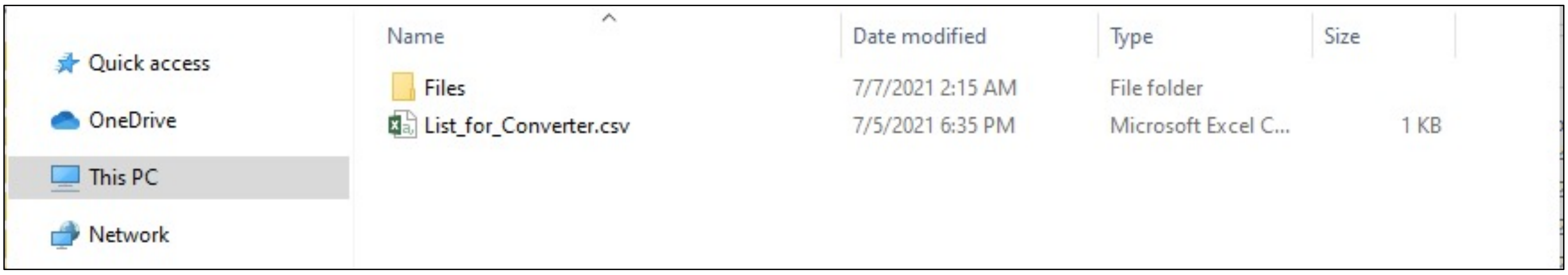

*The “Files” folder and “List_for_Converter.csv” are the demo data for “Y1H_1_for GM_Basic_demo”.

❖ Y1H (GM_Converter)

- The demo data of Y1H for GM_Basic is stored in “Y1H_1_for GM_Basic_demo” folder (green frame) of “Demo_1_Y1H” folder (red frame).
- Please see the Figure A1-A11 in Additional file 2 for the detailed concept of GM_Converter.

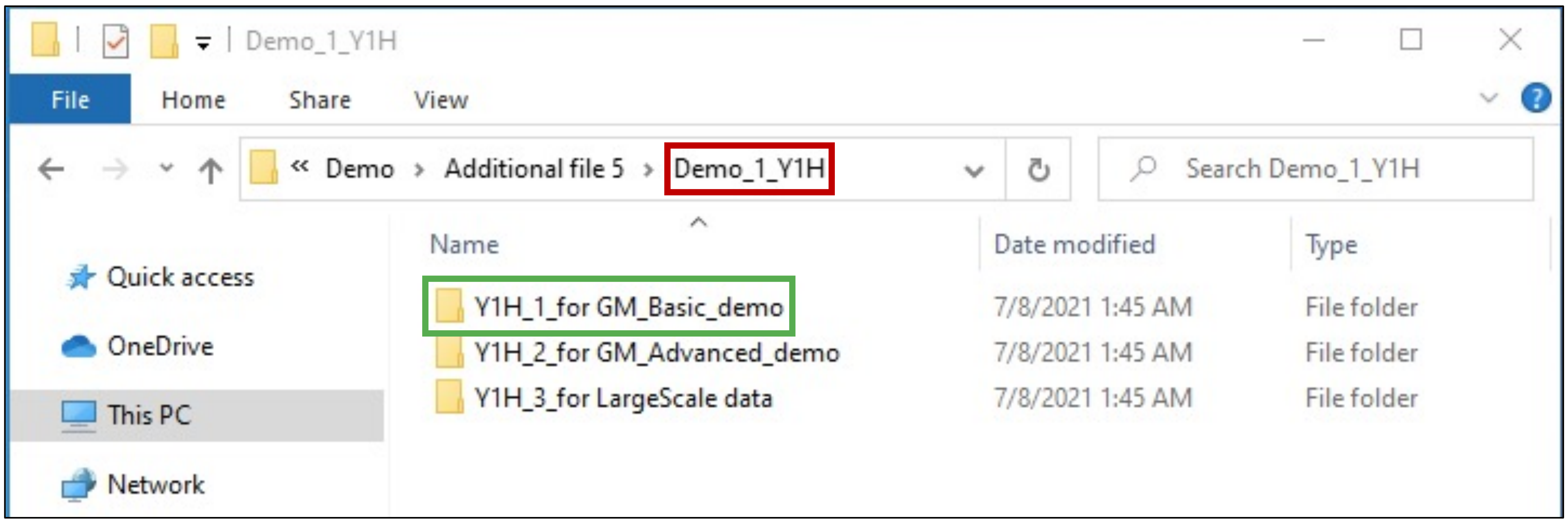

Open the “Y1H_1_ for GM_Basic_demo” folder (green frame)

- All SampleName files, Treatment files and Signal files need to be stored in one folder (in purple frame).
- Only the provided Signal file is the raw data. The other files, such as SampleName files and Treatment files should be provided by users (Here, the prepared SampleName files and Treatment files are provided).

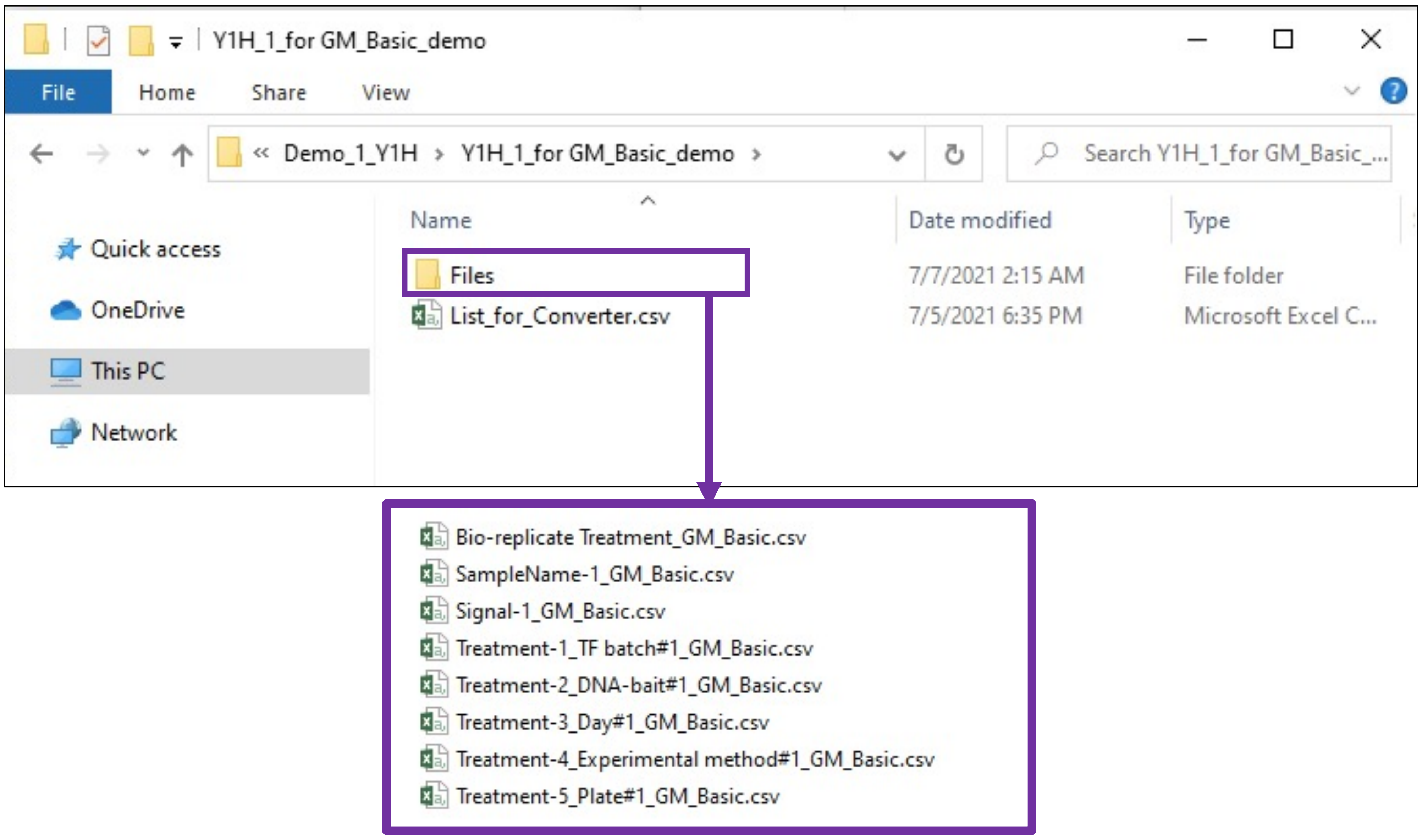

- Files in “Files” folder for using converter (Y1H for GM_Basic)
  The content of files are shown below

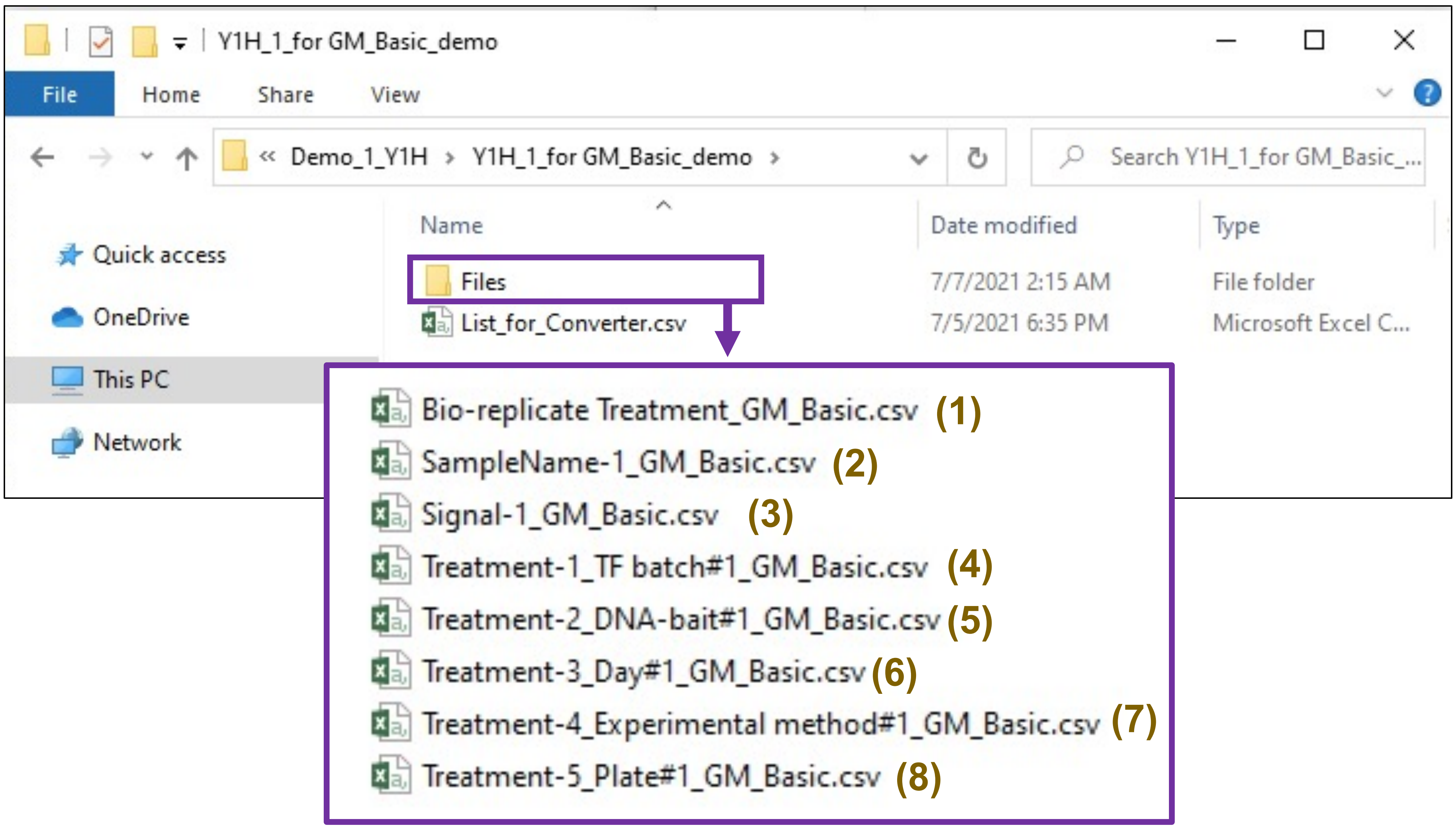

*TF batch#1: Batch1
*DNA-bait#1: CCoAOMT1 (Promoter)
*Day#1: Day4
*Experimental method#1: Meiosis
*Plate#1: ABA2 (ABA is an antibiotic, and here means that the plate is with antibiotics)

	(1) A	(2) A	(4) A	(5) A	(6) A	(7) A	(8) A
1	Bio-1	TF#001	Batch1	CCoAOMT1	Day4	Meiosis	ABA2
2	Bio-1	TF#001	Batch1	CCoAOMT1	Day4	Meiosis	ABA2
3	Bio-1	TF#002	Batch1	CCoAOMT1	Day4	Meiosis	ABA2
4	Bio-1	TF#002	Batch1	CCoAOMT1	Day4	Meiosis	ABA2
5	Bio-1	EV	Batch1	CCoAOMT1	Day4	Meiosis	ABA2
6	Bio-1	EV	Batch1	CCoAOMT1	Day4	Meiosis	ABA2
7	Bio-1	TF#005	Batch1	CCoAOMT1	Day4	Meiosis	ABA2
8	Bio-1	TF#005	Batch1	CCoAOMT1	Day4	Meiosis	ABA2
9	Bio-1	TF#006	Batch1	CCoAOMT1	Day4	Meiosis	ABA2
10	Bio-1	TF#006	Batch1	CCoAOMT1	Day4	Meiosis	ABA2

(3)

	A	B	C	D	E	F	G	H	I	J	K	L	M	N
1	Run	Plate	Type	Row	Col	Size	Circularity	Brightnes:	Redness	Avg..Red	Avg..Blue	Avg..Gree	Multi	Gene
2	1	9	None	A	1	0	0	0	0	0	0	0		
3	1	9	None	A	2	0	0	0	0	0	0	0		
4	1	9	None	A	3	255	0.9216	250.5935	0.7	252	252	247		
5	1	9	None	A	4	302	0.9238	250.9592	0.7	252	252	248		
6	1	9	None	A	5	0	0	0	0	0	0	0		
7	1	9	None	A	6	0	0	0	0	0	0	0		
8	1	9	None	A	7	0	0	0	0	0	0	0		
9	1	9	None	A	8	0	0	0	0	0	0	0		
10	1	9	None	A	9	67	0.8507	249.1244	0.4	250	250	246		
11	1	9	None	A	10	73	0.8356	249.6164	0.2	250	250	247		

Signal

List_for_Converter.csv”

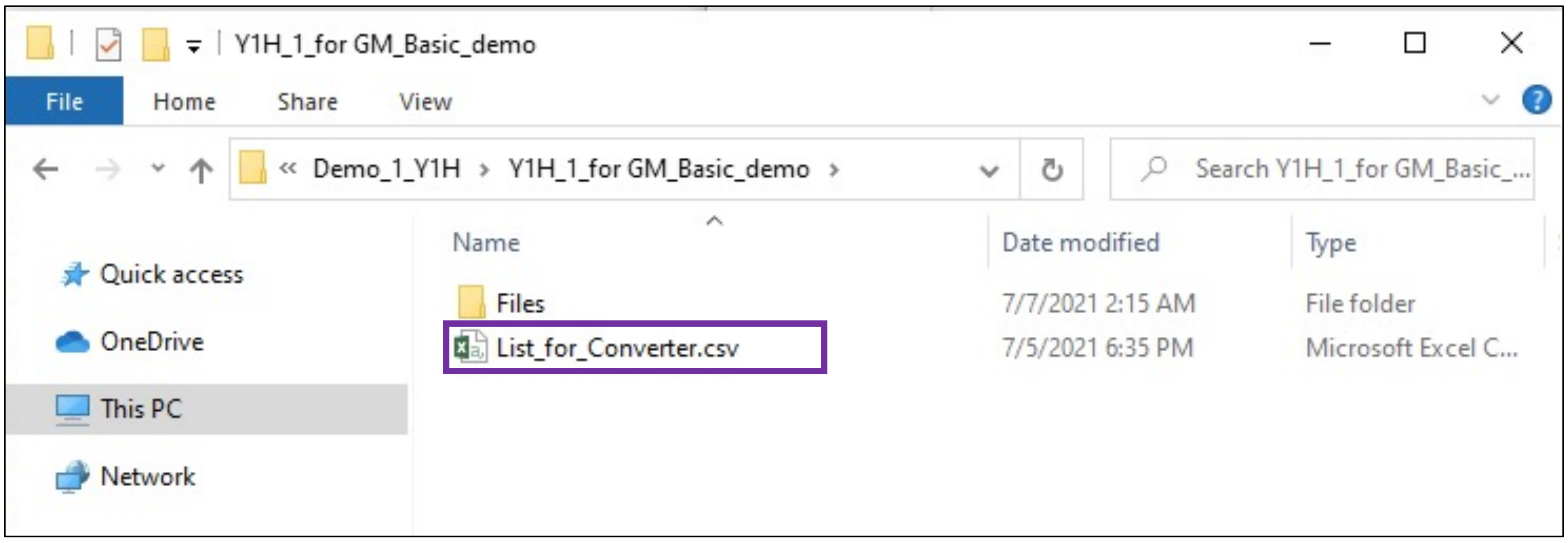

- The data title in the first row of “Converted_data.csv” file (the result file output from GM_Converter) is from the first row of “List_for_Converter” file (red frame). Please see the Figure A1-A11 in Additional file 2 for the detailed concept of GM_Converter.

- The data of files listed in the same row of ”List_for_Converter.csv” (blue frame) will be integrated. Please see the Figure A1-A11 in Additional file 2 for the detailed concept of GM_Converter.

“Converted_data.csv”

First row →

	A	B	C	D	E	F	G	H
1	SampleName_TF-prey	Treatment-1_TF batch	Treatment-2_DNA-bait	Treatment-3_Day	Treatment-4_Experimental method	Treatment-5_Plate	Bio-replicate Treatment_Bio-rep	Signal
2	TF#001	Batch1	CCoAOMT1	Day4	Meiosis	ABA2	Bio-1	0
3	TF#001	Batch1	CCoAOMT1	Day4	Meiosis	ABA2	Bio-1	0
4	TF#002	Batch1	CCoAOMT1	Day4	Meiosis	ABA2	Bio-1	255
5	TF#002	Batch1	CCoAOMT1	Day4	Meiosis	ABA2	Bio-1	302
6	EV	Batch1	CCoAOMT1	Day4	Meiosis	ABA2	Bio-1	0
7	EV	Batch1	CCoAOMT1	Day4	Meiosis	ABA2	Bio-1	0
8	TF#005	Batch1	CCoAOMT1	Day4	Meiosis	ABA2	Bio-1	0
9	TF#005	Batch1	CCoAOMT1	Day4	Meiosis	ABA2	Bio-1	0
10	TF#006	Batch1	CCoAOMT1	Day4	Meiosis	ABA2	Bio-1	67
11	TF#006	Batch1	CCoAOMT1	Day4	Meiosis	ABA2	Bio-1	73
12	TF#008	Batch1	CCoAOMT1	Day4	Meiosis	ABA2	Bio-1	0
13	TF#008	Batch1	CCoAOMT1	Day4	Meiosis	ABA2	Bio-1	0
14	TF#001	Batch1	CCoAOMT1	Day4	Meiosis	ABA2	Bio-2	0
15	TF#001	Batch1	CCoAOMT1	Day4	Meiosis	ABA2	Bio-2	3
16	TF#002	Batch1	CCoAOMT1	Day4	Meiosis	ABA2	Bio-2	614
17	TF#002	Batch1	CCoAOMT1	Day4	Meiosis	ABA2	Bio-2	282

“List_for_Converter.csv”

	A	B		G	H
1	SampleName_TF-prey	Treatment-1_TF batch		Bio-replicate Treatment_Bio-rep	Signal
2	SampleName-1_GM_Basic.csv	Treatment-1_TF batch#1_GM_Basic.csv	......	Bio-replicate Treatment_GM_Basic.csv	Signal-1_GM_Basic.csv
3					
4					

- Operation steps

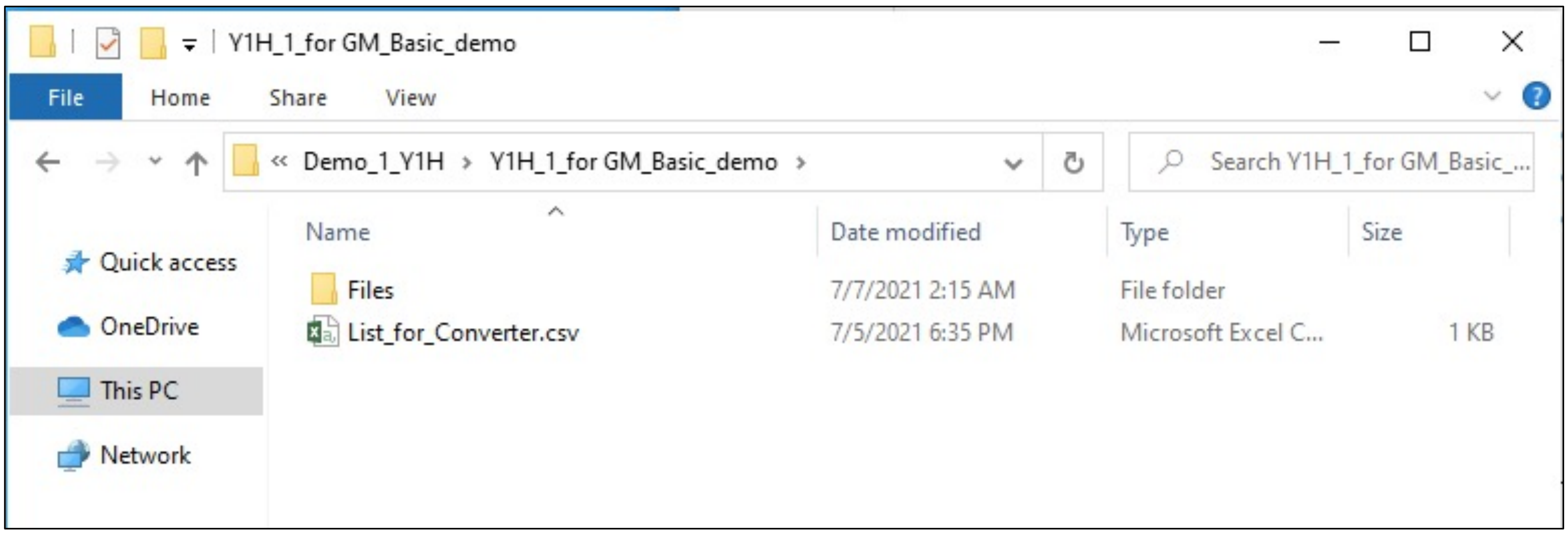

Copy and paste the two executive files of GM_Converter (GateMultiplex_converter.exe and Converter.exe) into "Y1H_1_for GM_Basic_demo" folder (purple frames and purple arrows)

*The two executive files should be placed in the same folder

- The "List_for_Converter.csv" (indicated by the green arrow) should be placed in the same folder with the two executive files of GM_Converter (GateMultiplex_converter.exe and Converter.exe).

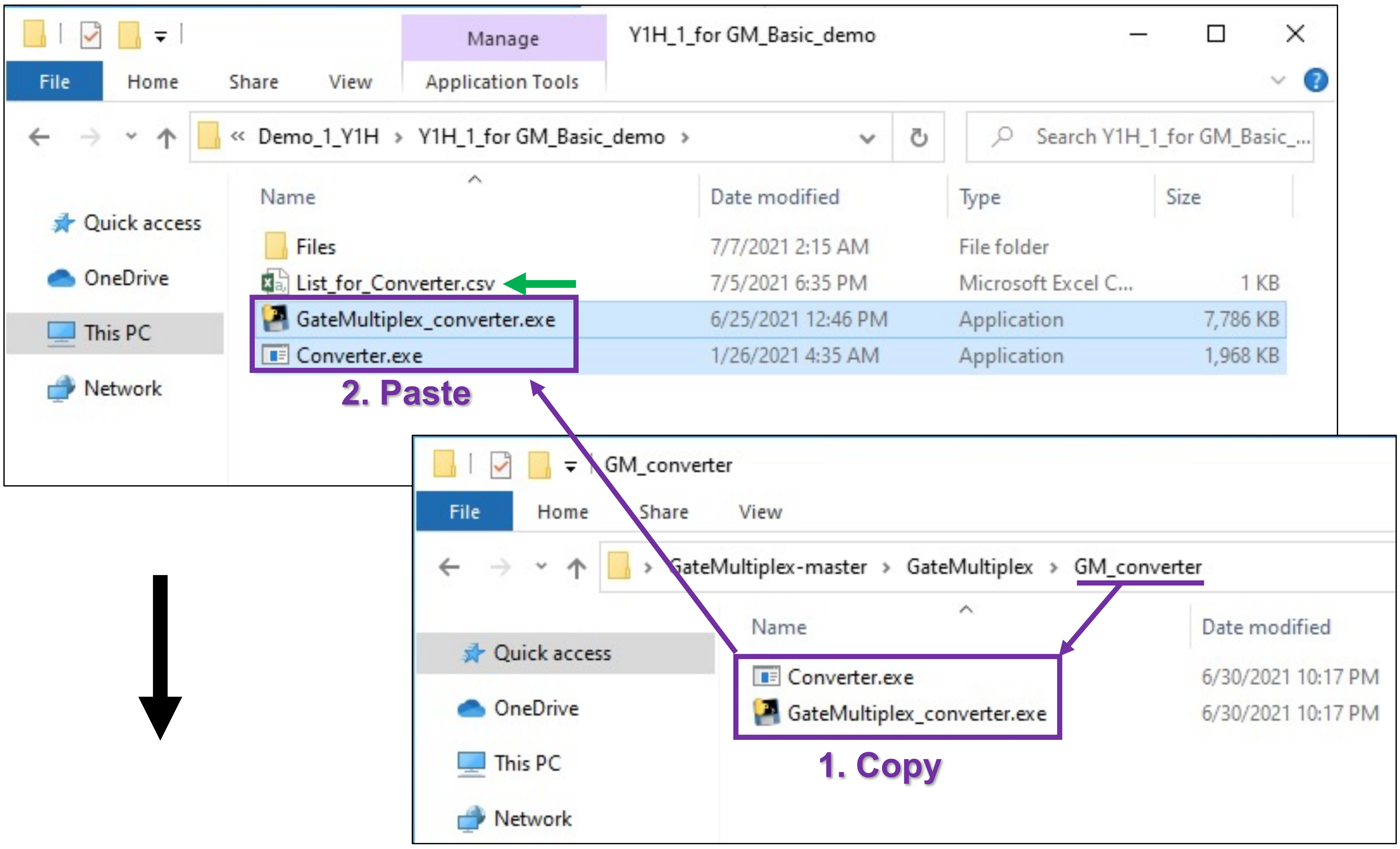

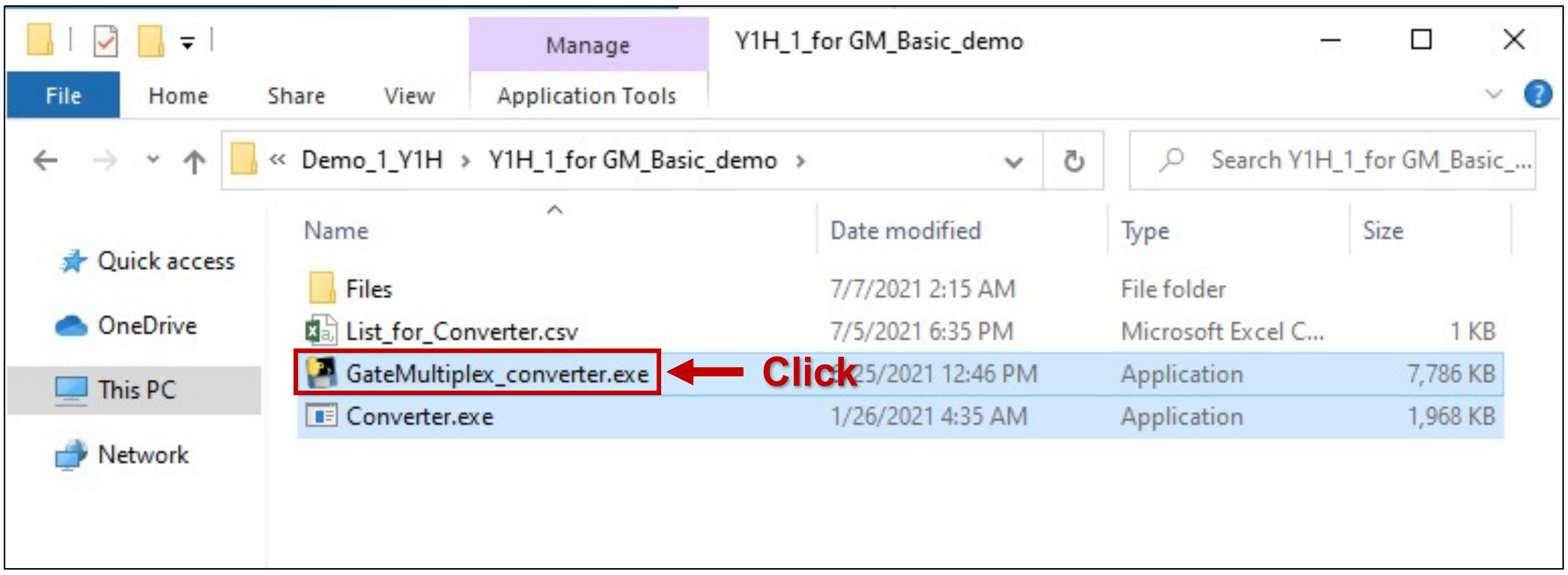

Activate the GM_Converter by a double-clicking on "GateMultiplex_converter.exe" (red frame)

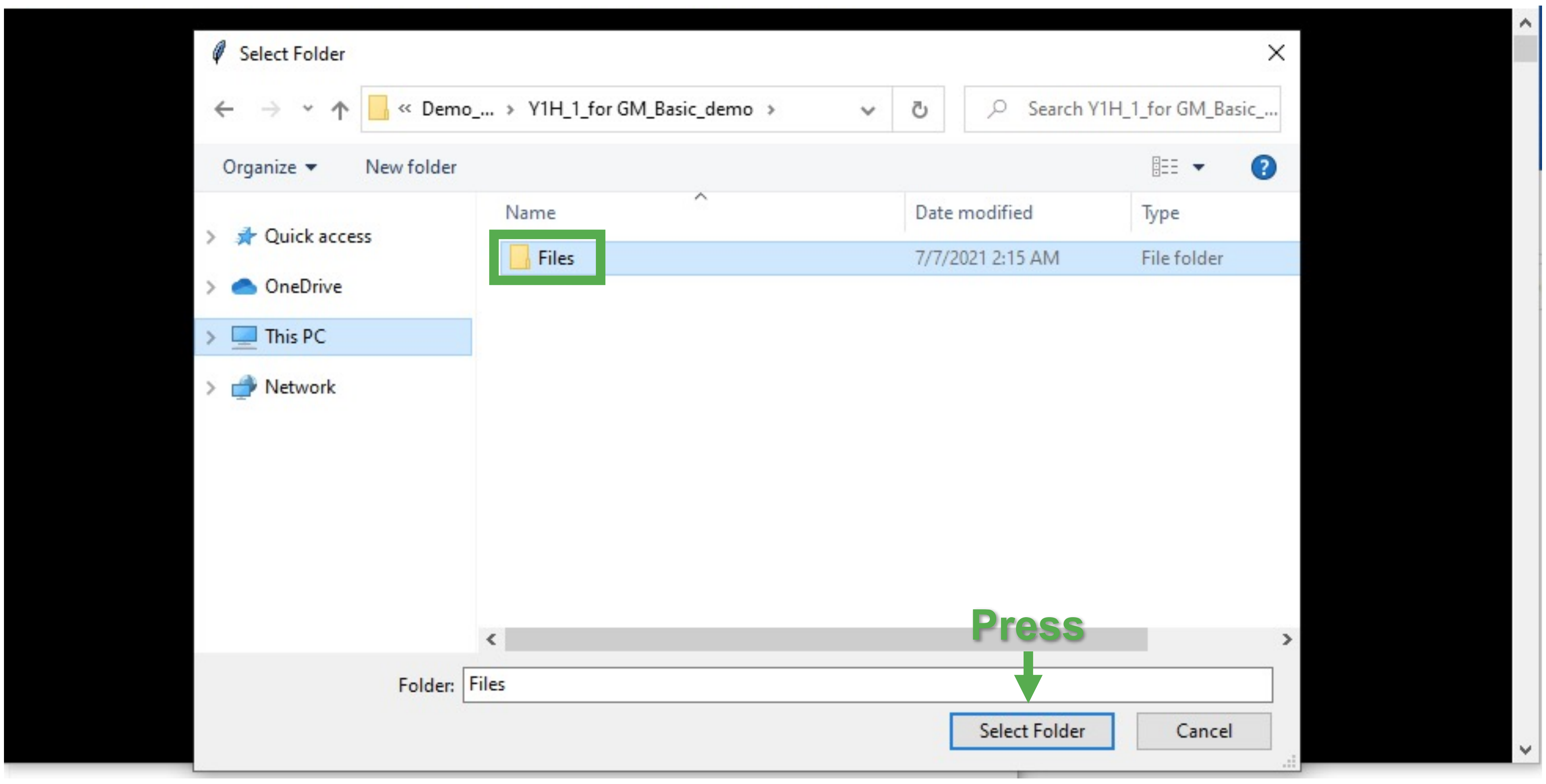

Select the folder "Files" (Additional file 5 > Demo_1_Y1H > Y1H_1_for GM_Basic_demo > Files) (green frame) and press "Select Folder" (indicated by a green arrow)

- Please see the Figure A5-A11 in Additional file 2 for the detailed parameter setting of GM_Converter.

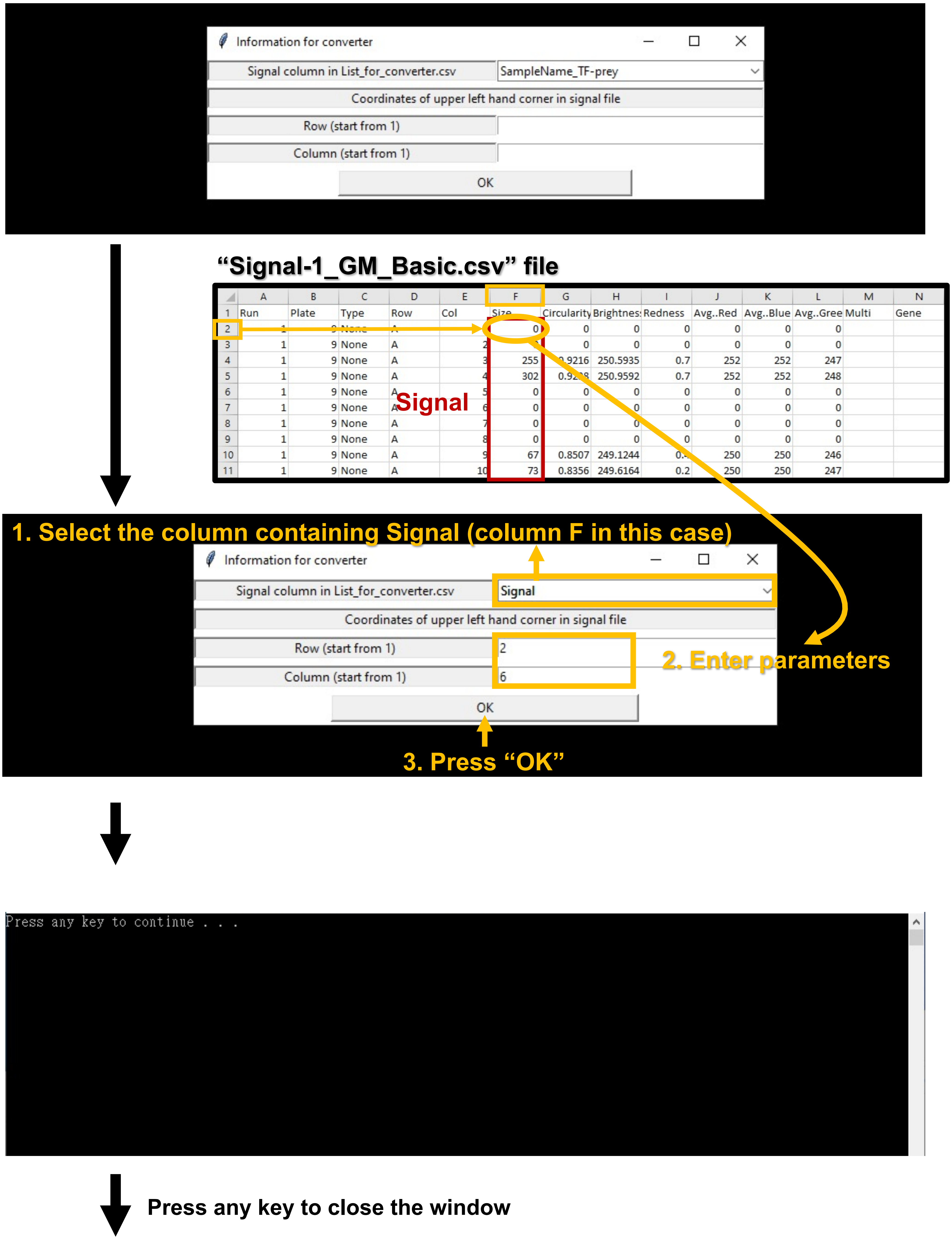

- The converted file “Converted_data.csv” (green frame) is stored in the “GM_converted_data” folder (red frame)

- The title of “Converted_data.csv” (circled by blue frame) is from the title in “List_for_Converter.csv”. Please see Figure A5-A11 in Additional file 2.

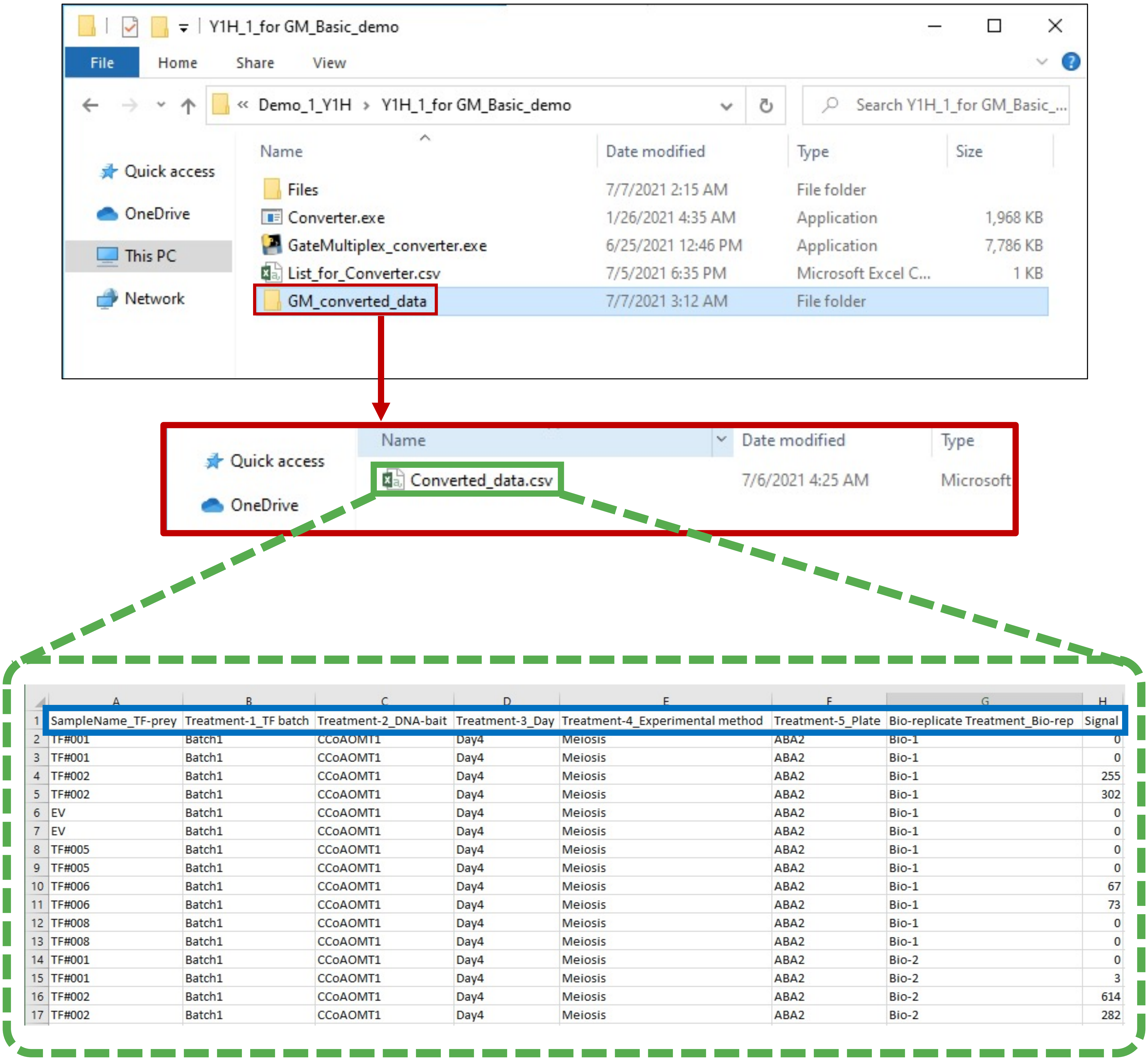

	A	B	C	D	E	F	G	H
1	SampleName_TF-prey	Treatment-1_TF batch	Treatment-2_DNA-bait	Treatment-3_Day	Treatment-4_Experimental method	Treatment-5_Plate	Bio-replicate Treatment_Bio-rep	Signal
2	TF#001	Batch1	CCoAOMT1	Day4	Meiosis	ABA2	Bio-1	0
3	TF#001	Batch1	CCoAOMT1	Day4	Meiosis	ABA2	Bio-1	0
4	TF#002	Batch1	CCoAOMT1	Day4	Meiosis	ABA2	Bio-1	255
5	TF#002	Batch1	CCoAOMT1	Day4	Meiosis	ABA2	Bio-1	302
6	EV	Batch1	CCoAOMT1	Day4	Meiosis	ABA2	Bio-1	0
7	EV	Batch1	CCoAOMT1	Day4	Meiosis	ABA2	Bio-1	0
8	TF#005	Batch1	CCoAOMT1	Day4	Meiosis	ABA2	Bio-1	0
9	TF#005	Batch1	CCoAOMT1	Day4	Meiosis	ABA2	Bio-1	0
10	TF#006	Batch1	CCoAOMT1	Day4	Meiosis	ABA2	Bio-1	67
11	TF#006	Batch1	CCoAOMT1	Day4	Meiosis	ABA2	Bio-1	73
12	TF#008	Batch1	CCoAOMT1	Day4	Meiosis	ABA2	Bio-1	0
13	TF#008	Batch1	CCoAOMT1	Day4	Meiosis	ABA2	Bio-1	0
14	TF#001	Batch1	CCoAOMT1	Day4	Meiosis	ABA2	Bio-2	0
15	TF#001	Batch1	CCoAOMT1	Day4	Meiosis	ABA2	Bio-2	3
16	TF#002	Batch1	CCoAOMT1	Day4	Meiosis	ABA2	Bio-2	614
17	TF#002	Batch1	CCoAOMT1	Day4	Meiosis	ABA2	Bio-2	282

❖ Y1H (GM_Basic)

- After Converted_data.csv completing, GM_Basic is further applied for analysis.
- GM_Basic includes two executive files (GateMultiplex_basic.exe and GateMultiplex_forWindows.exe) which should be stored in the same folder.
- Activate the GM_basic by a double-clicking on “GateMultiplex_basic.exe” (red frame).

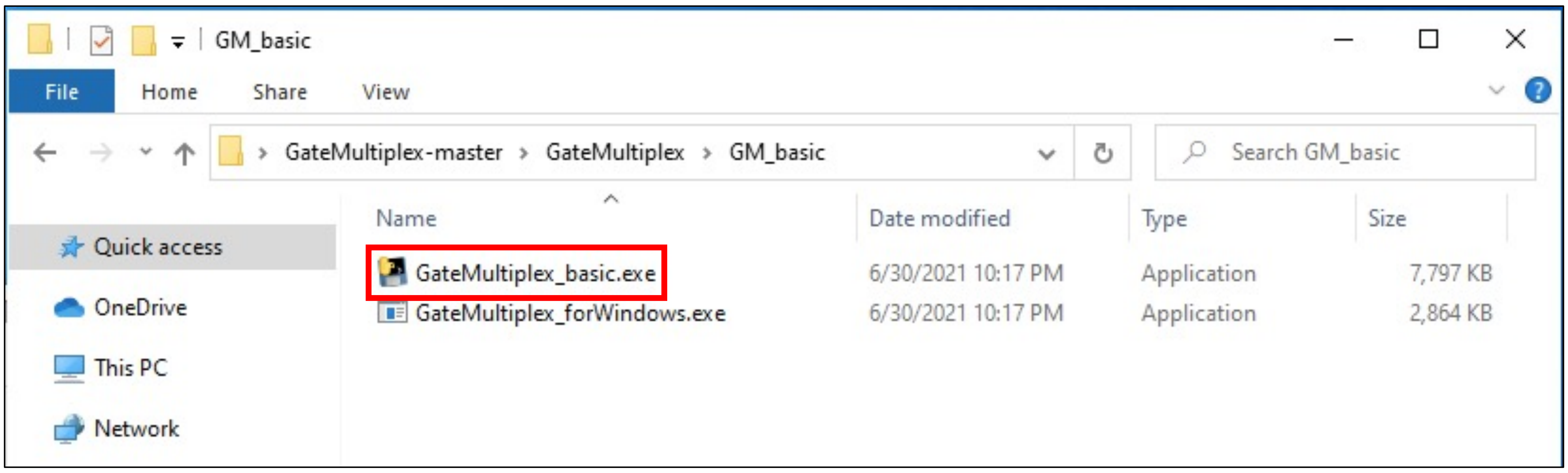

- Select the folder “GM_converted_data” (red frame) and press “Select Folder” (red arrow).
- Directory: Additional file 5 > Demo_1_Y1H > Y1H_1_for GM_Basic_demo > GM_converted_data

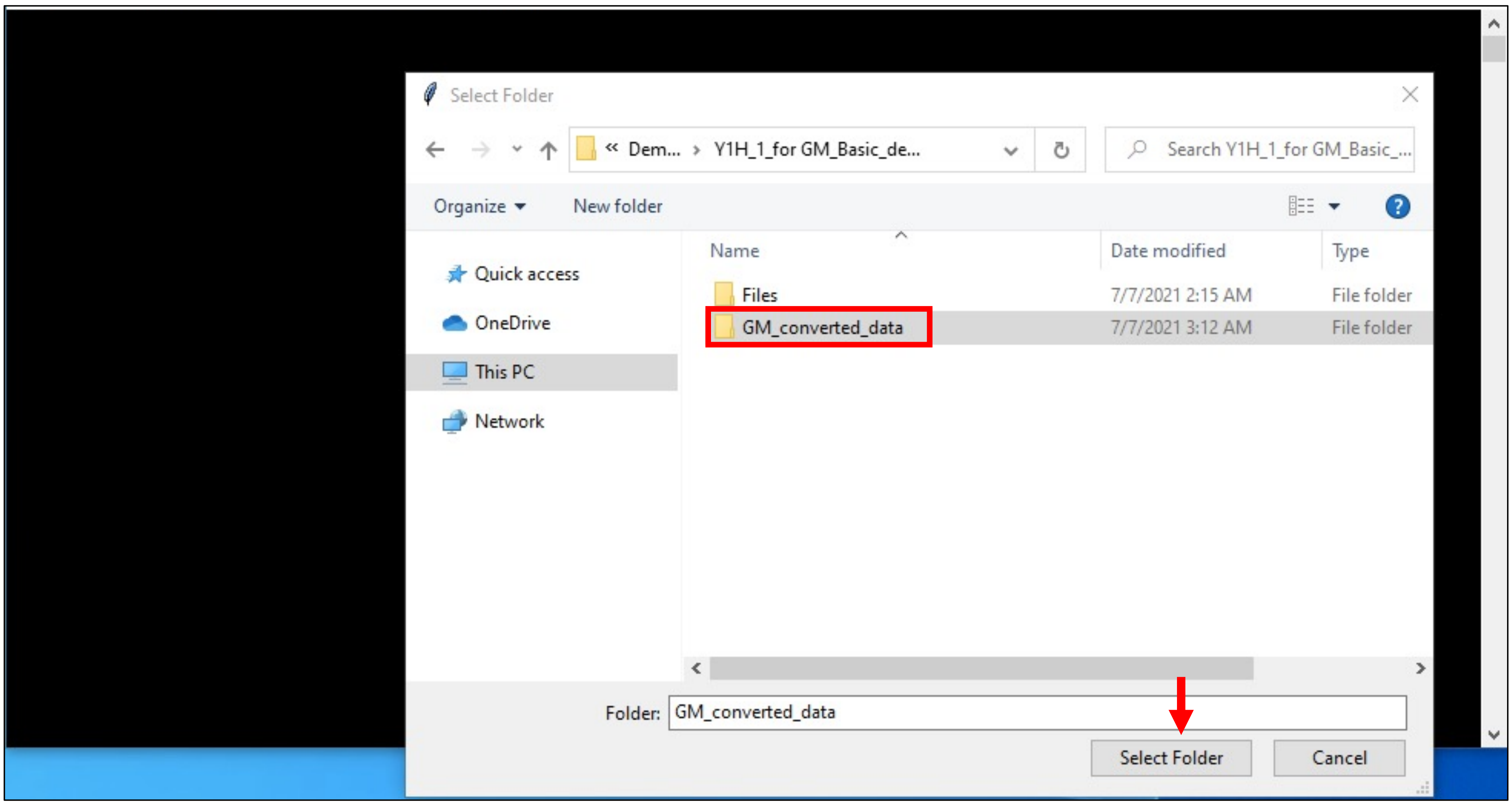

❖ Operation of GUI

✱ The GUI of GM_Basic

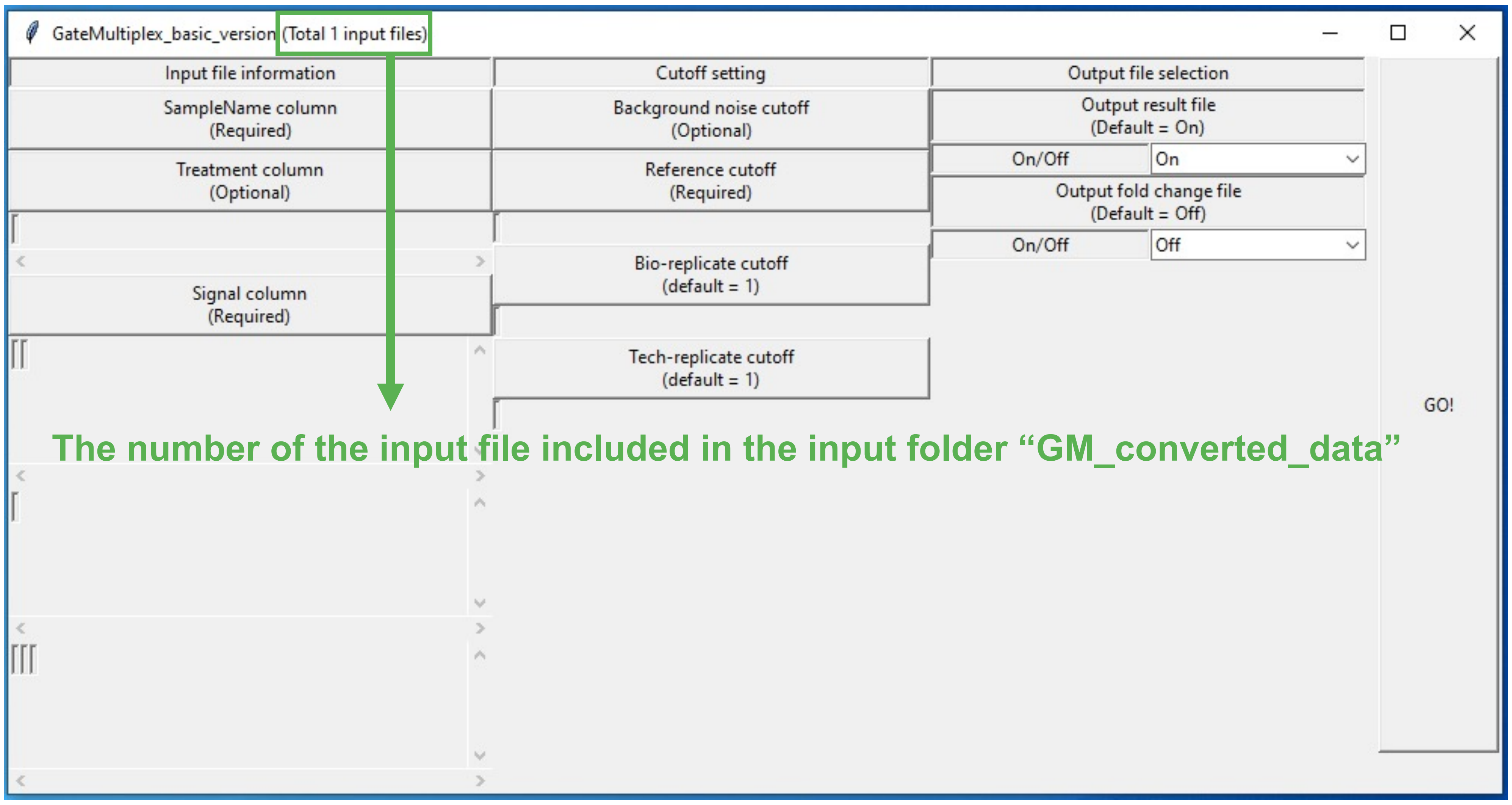

✱ SampleName selection

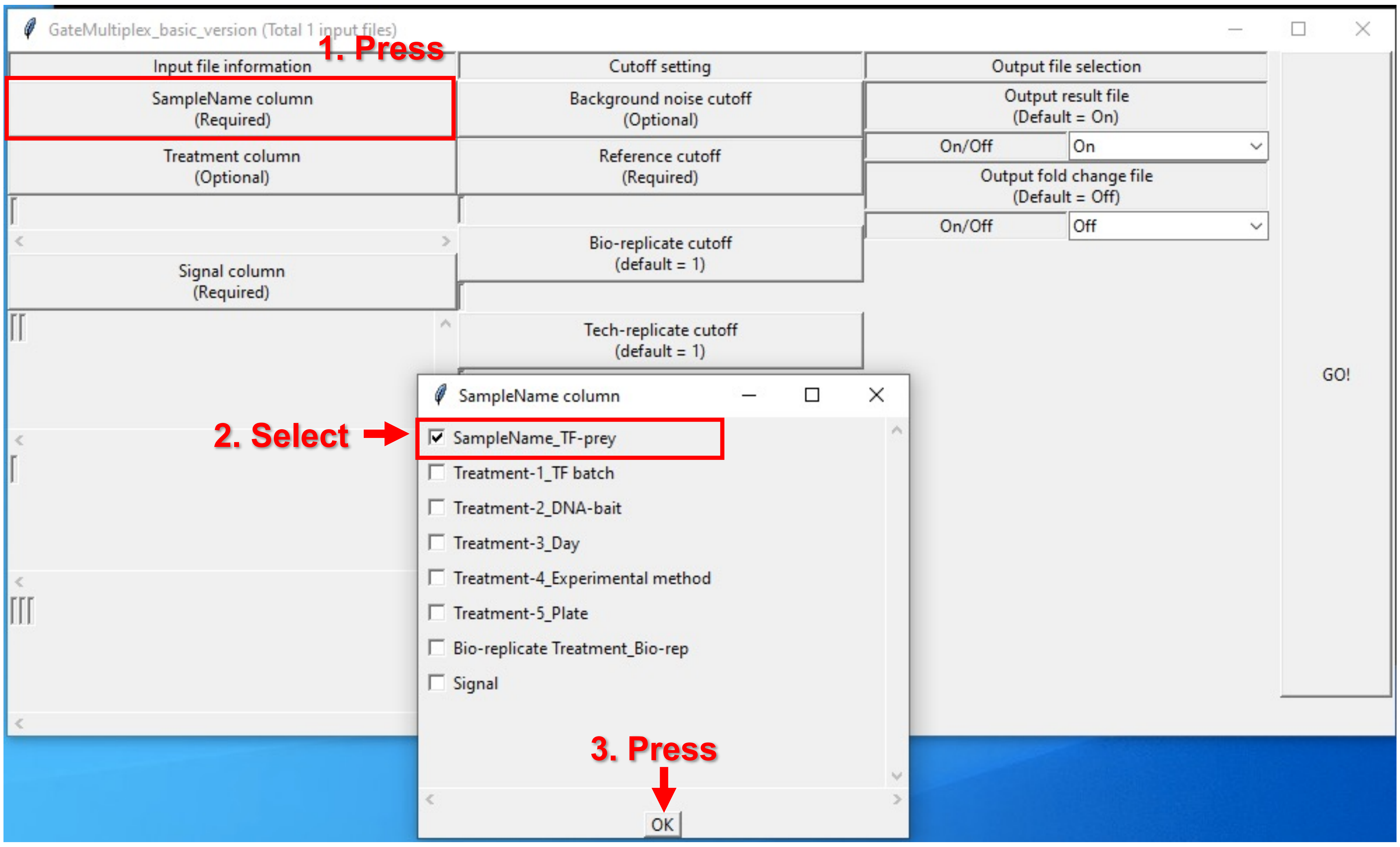

✱ Treatment selection

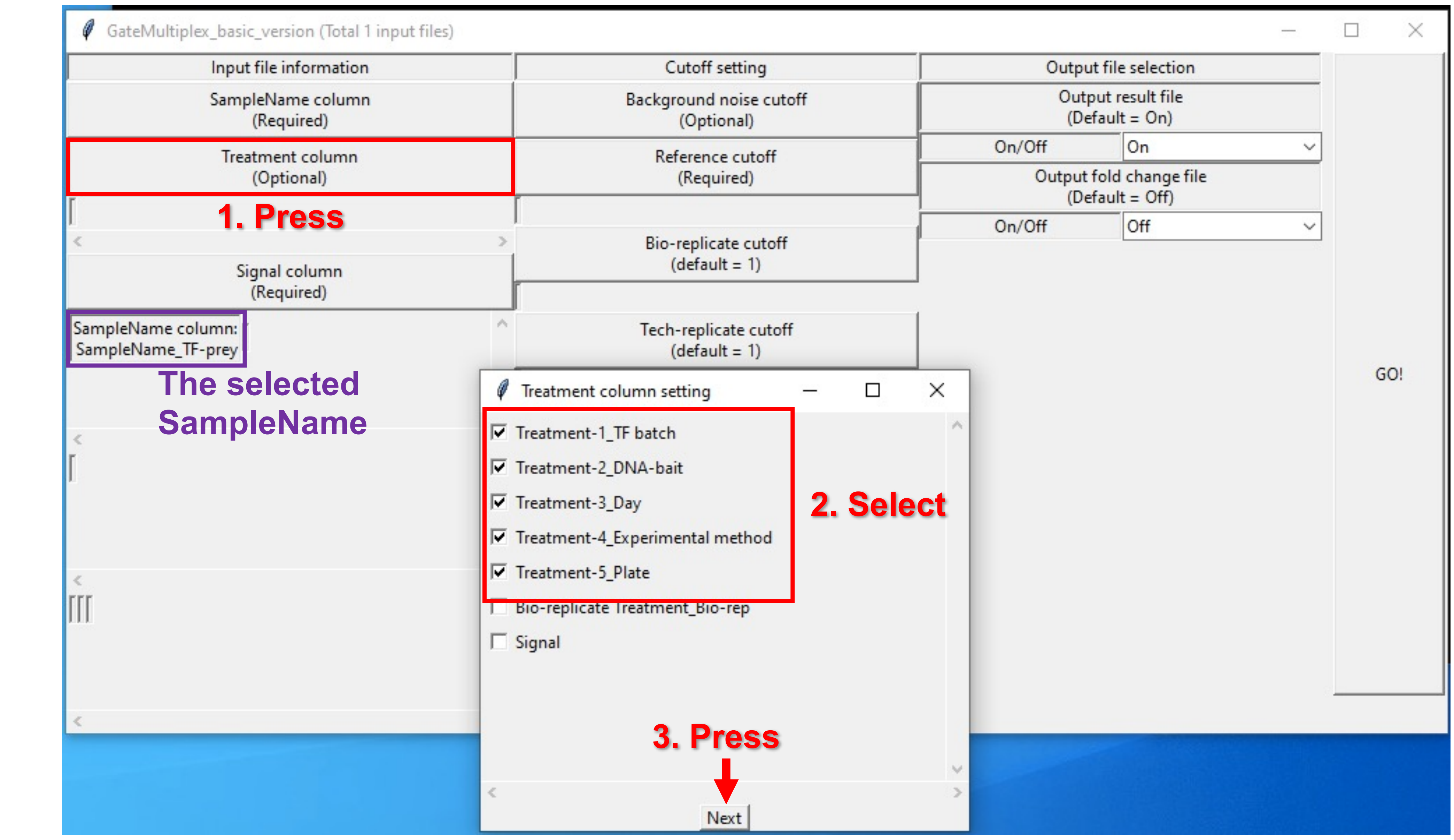

✱ Bio-replicate Treatment selection
(Please see Figure B2 in Additional file 2 for the concept of Bio-replicate Treatment)

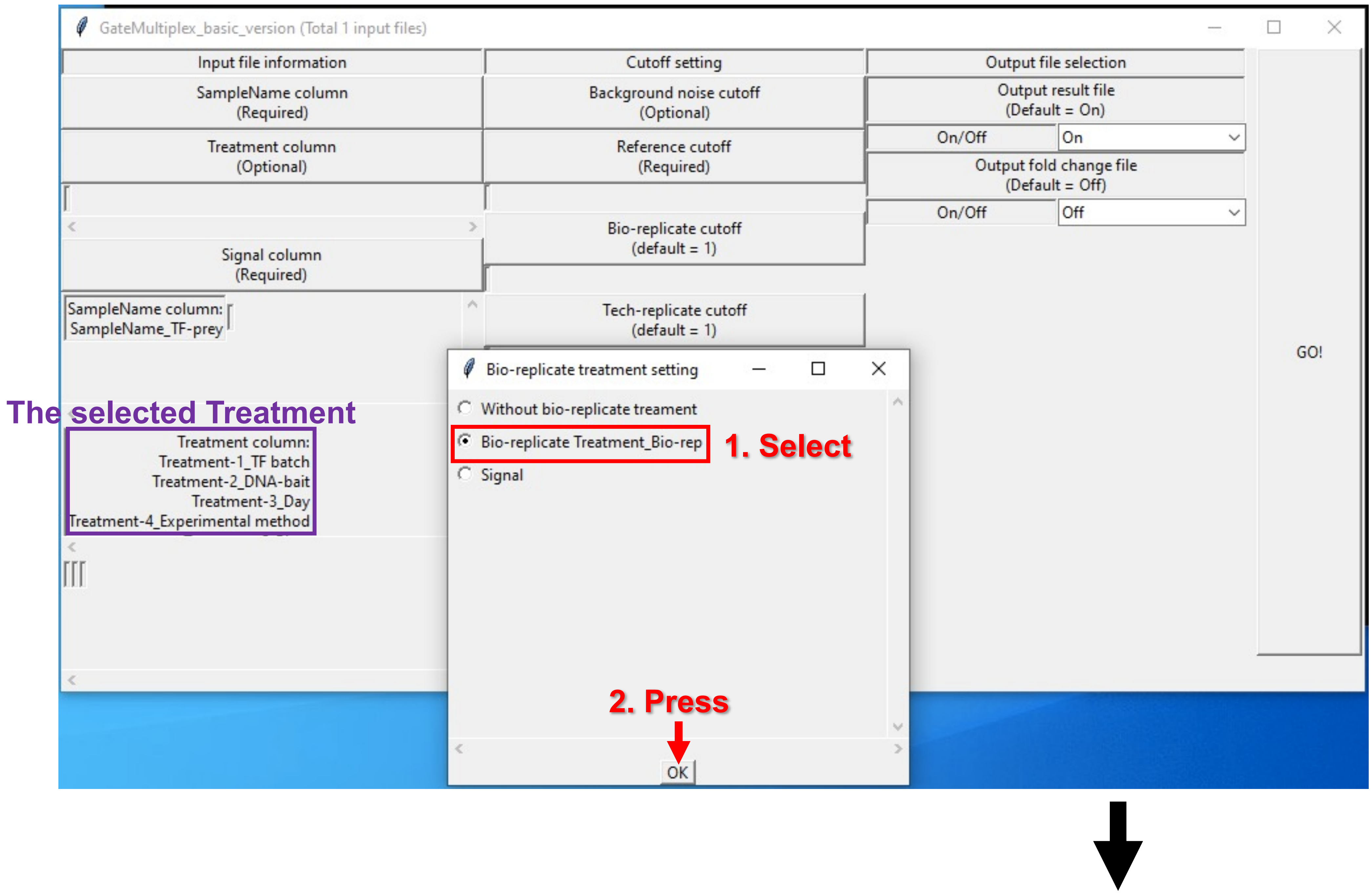

✱ Signal selection

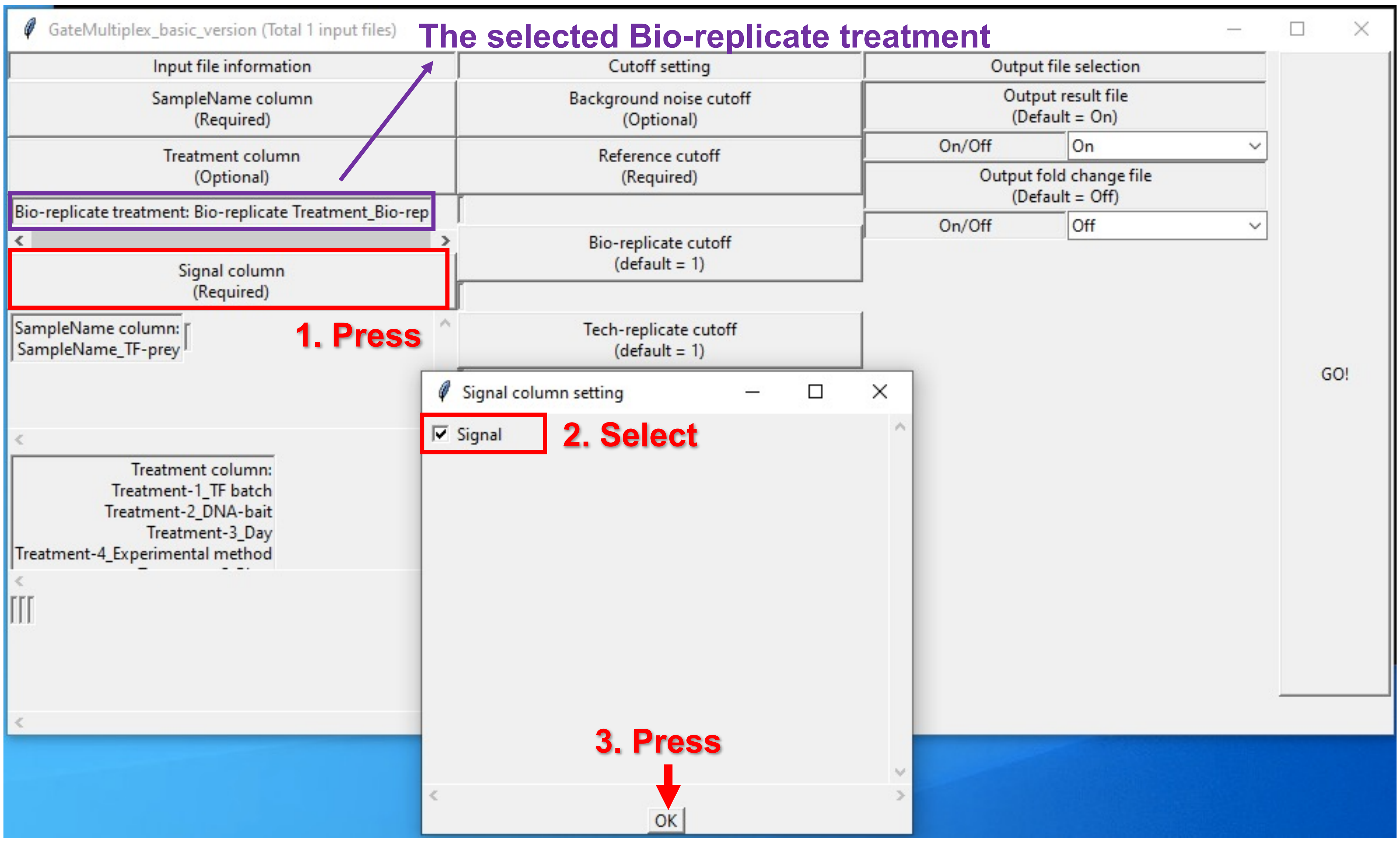

✱ Background noise cutoff setting

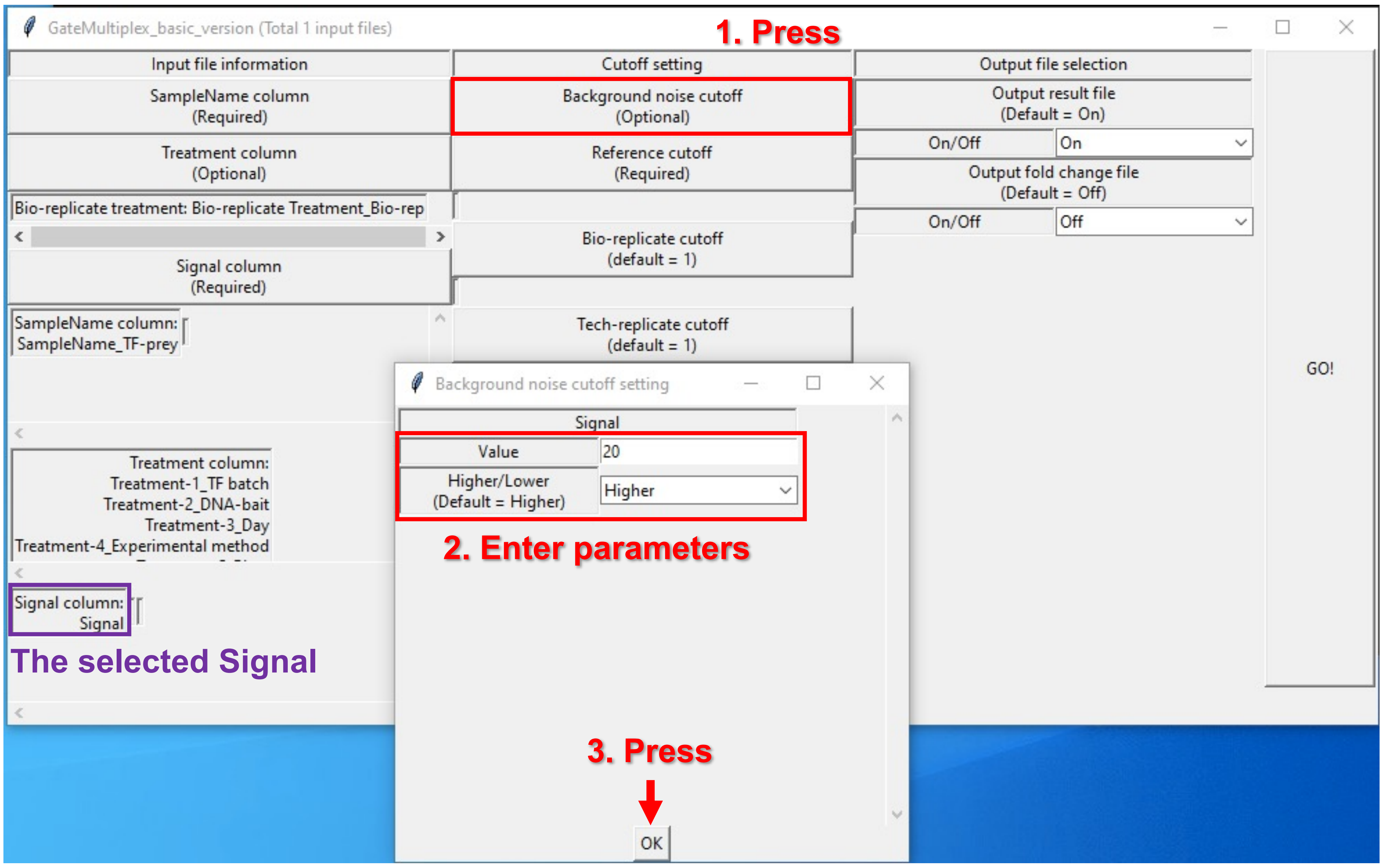

✱ Reference cutoff selection

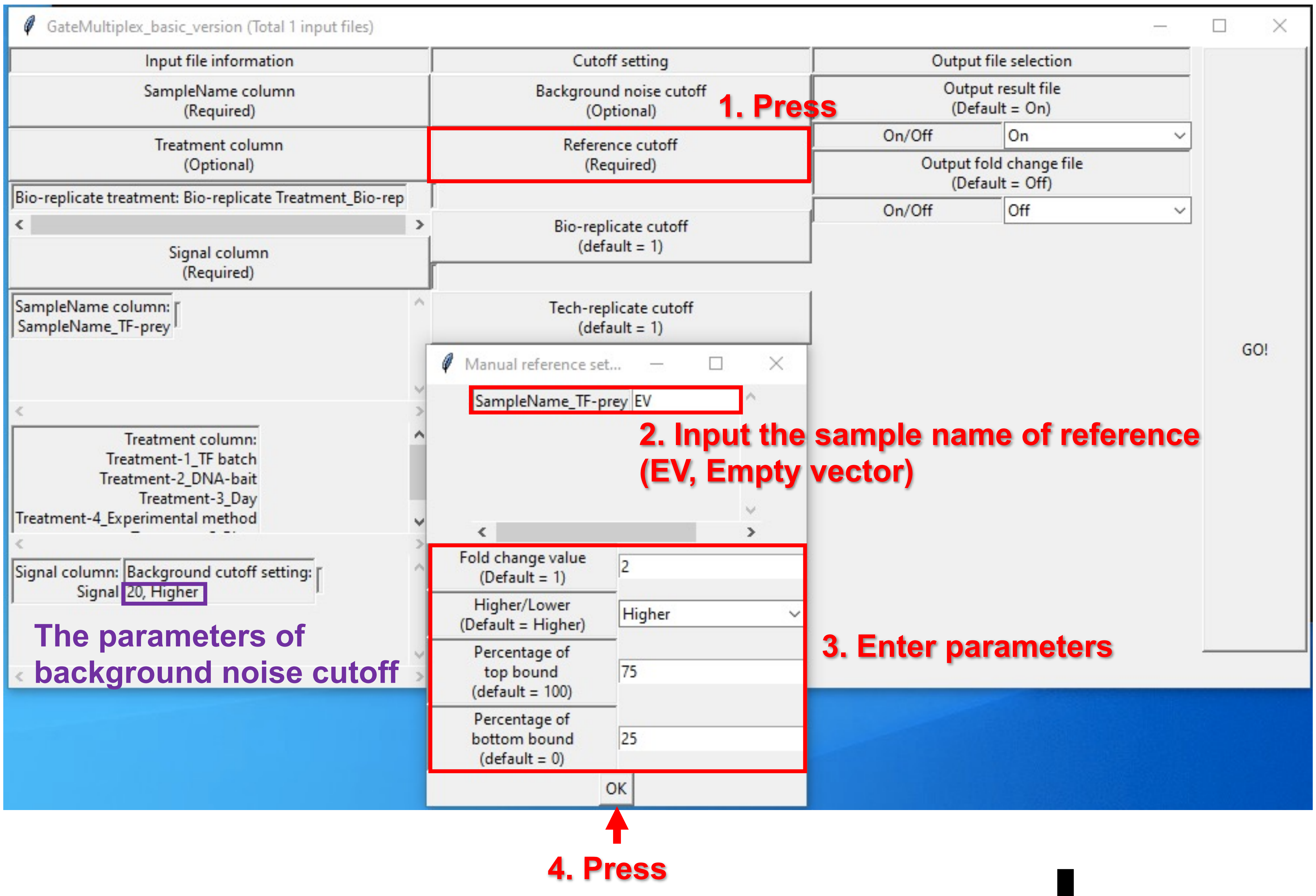

✱ Bio-replicate cutoff setting

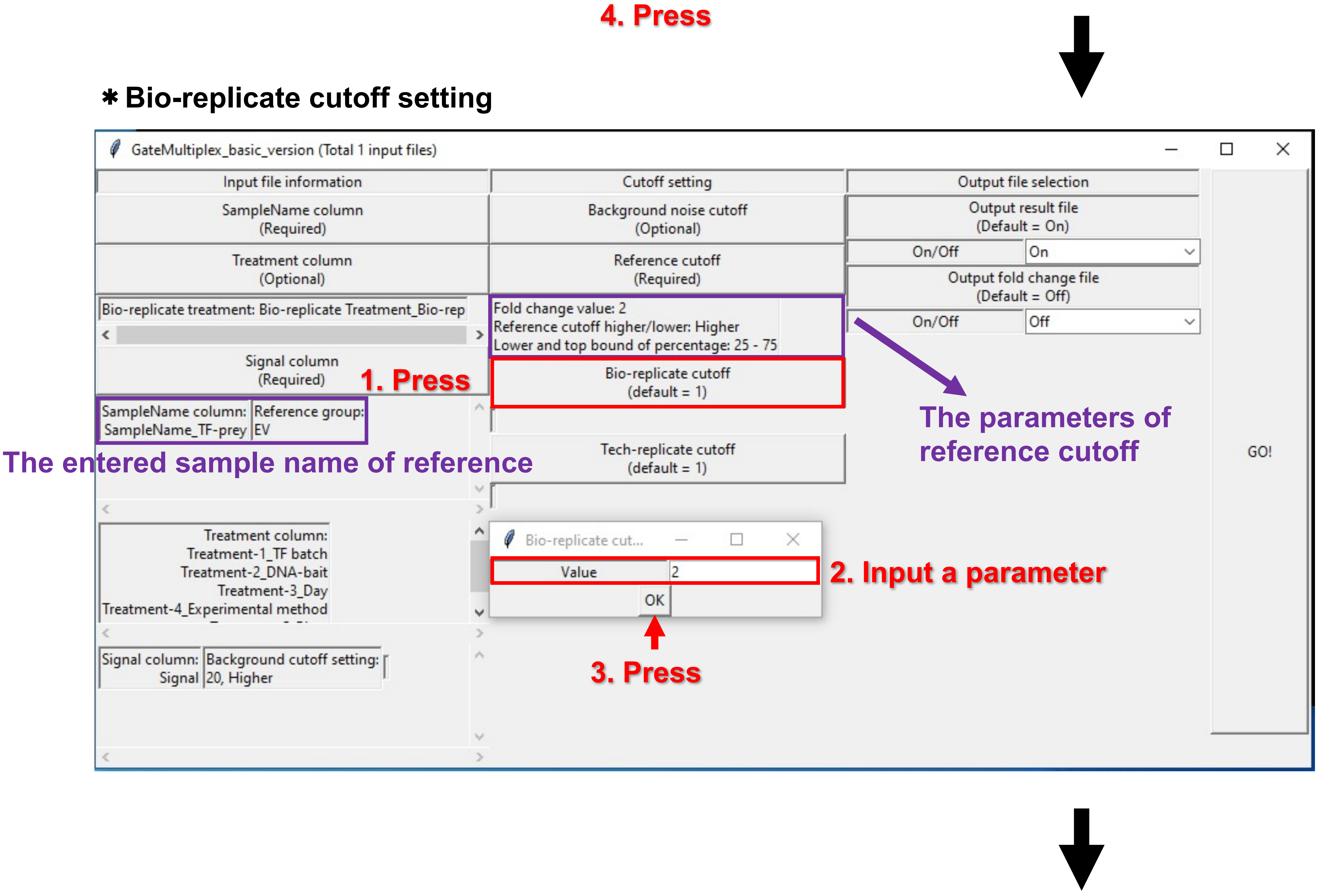

✱ Tech-replicate cutoff setting

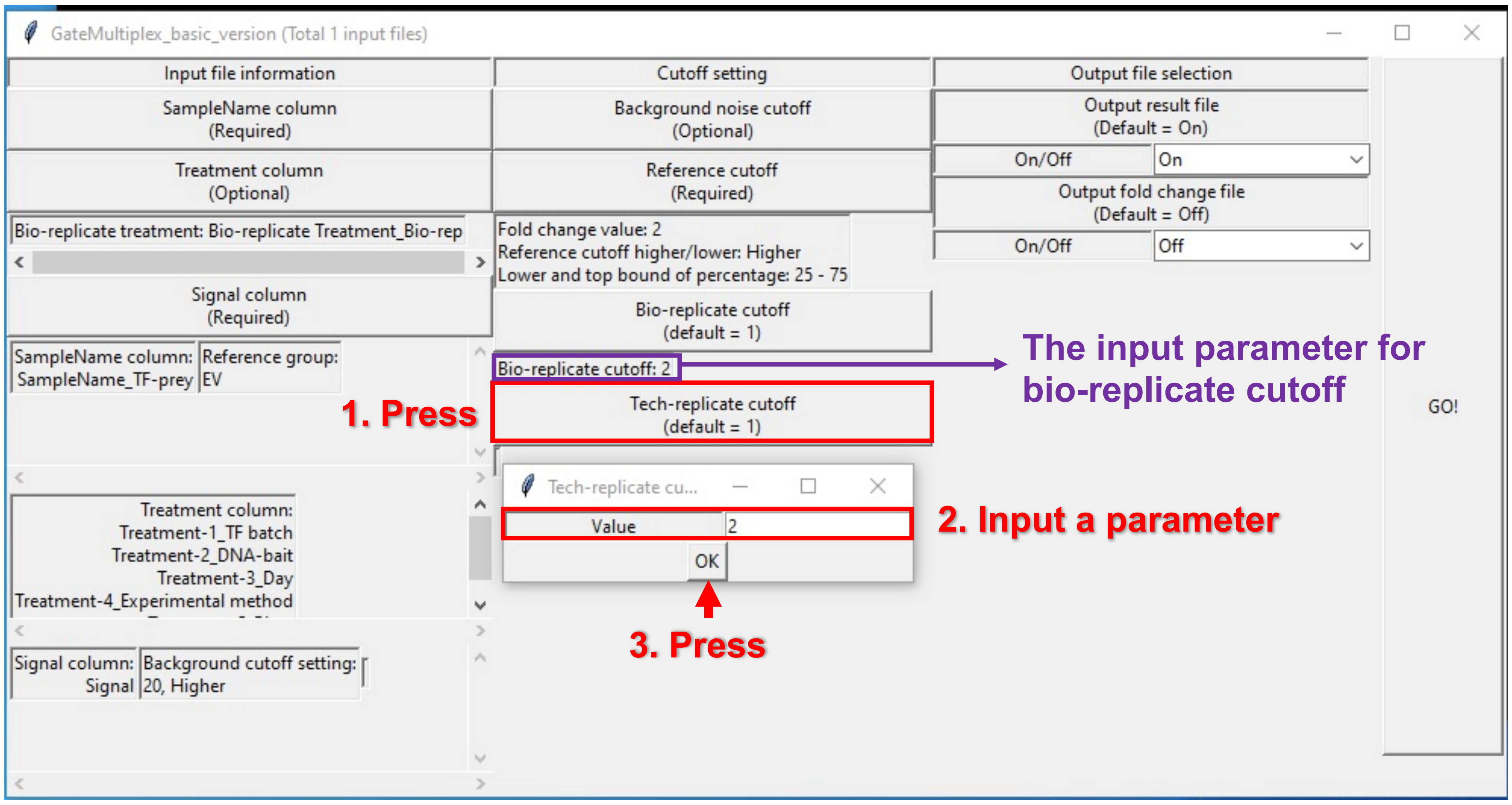

✱ Output file selection

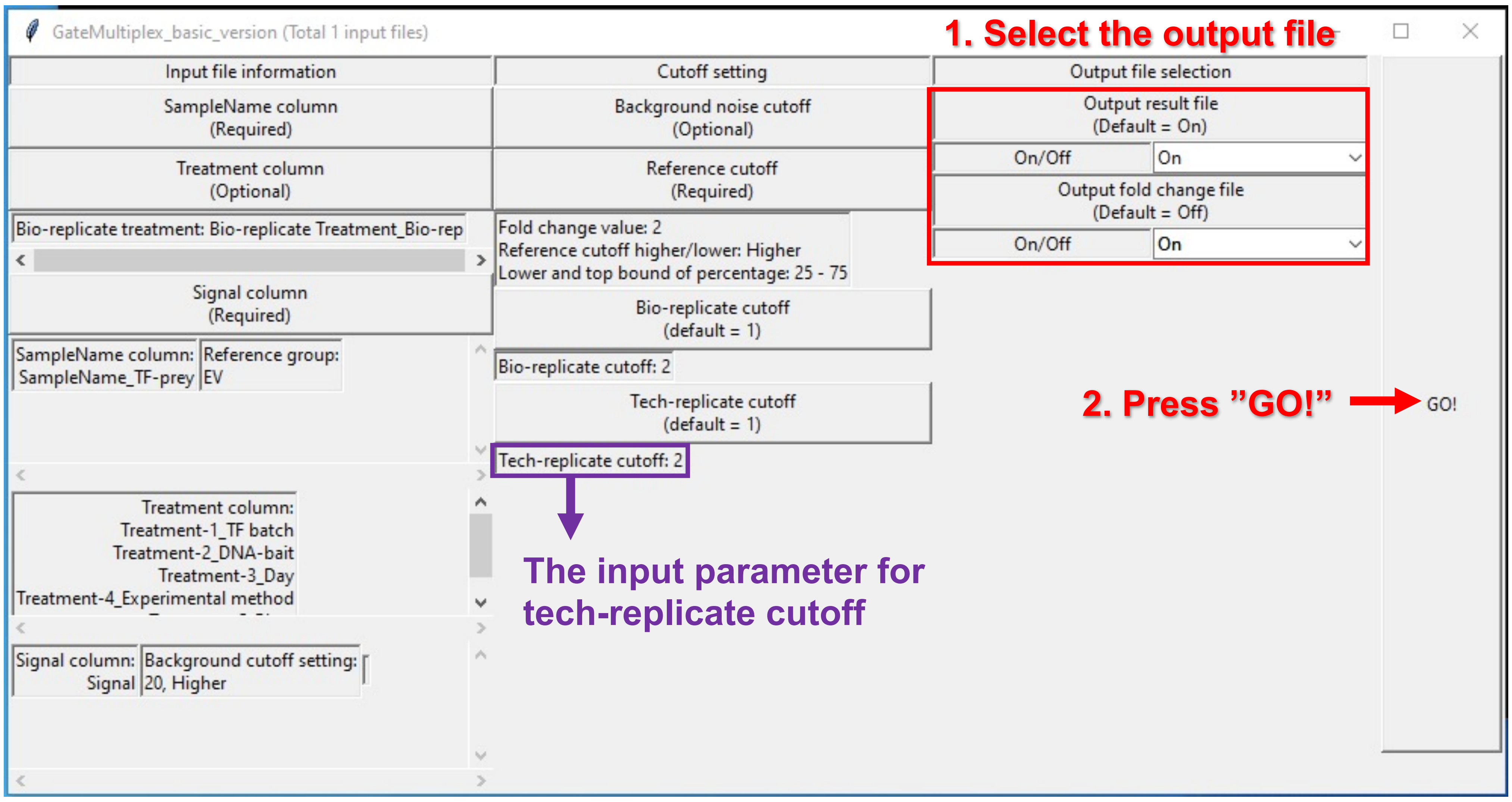

- The analysis will be completed after “Press any key to continue...” (red arrow) showing on the command line.

- Press any key to close the window.

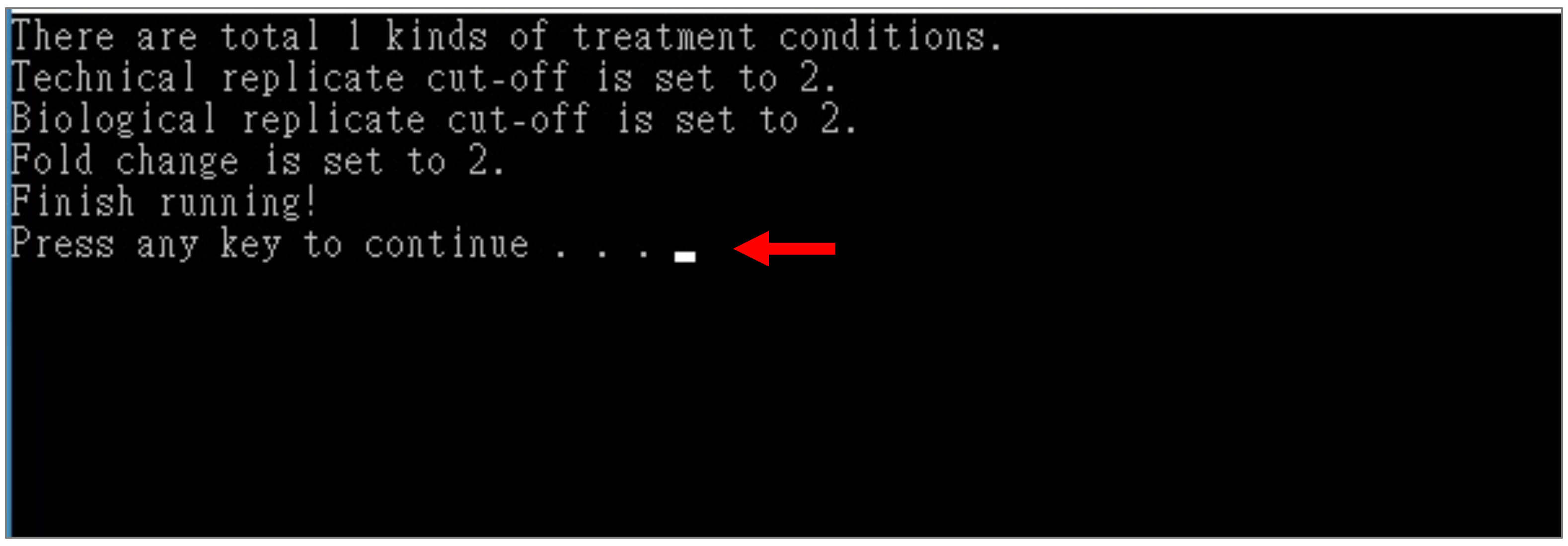

- The result file, named “Results. csv” (red frame), will be stored in the same folder with the executive files of GM_Basic.

- The fold change files will be stored in the folder named “GM_outputfiles” (green frame).

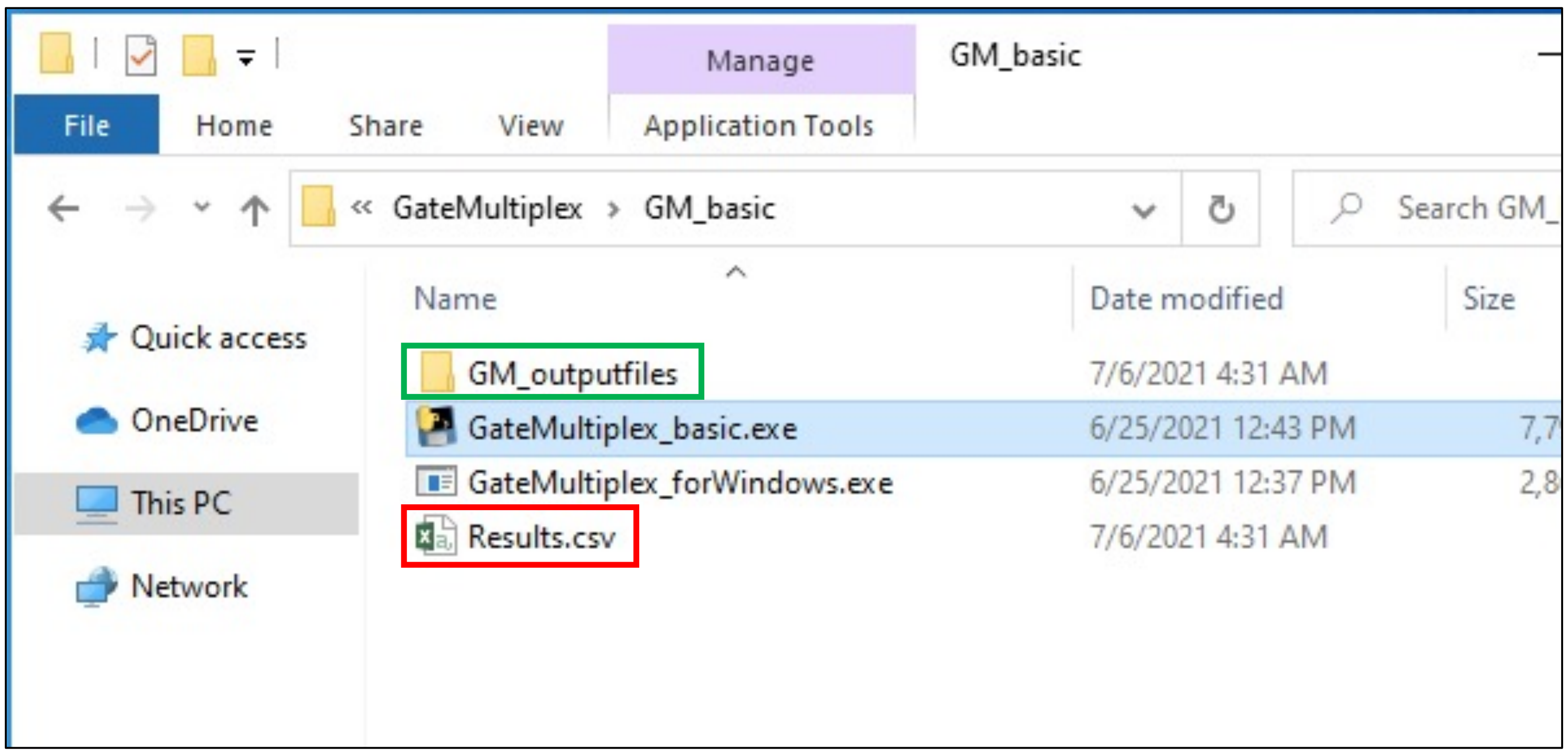

- The content of “Results.csv”.
- The symbol ”N” means negative, and the symbol “P” means positive. Please the “Symbols in output files” section in Additional file 3.

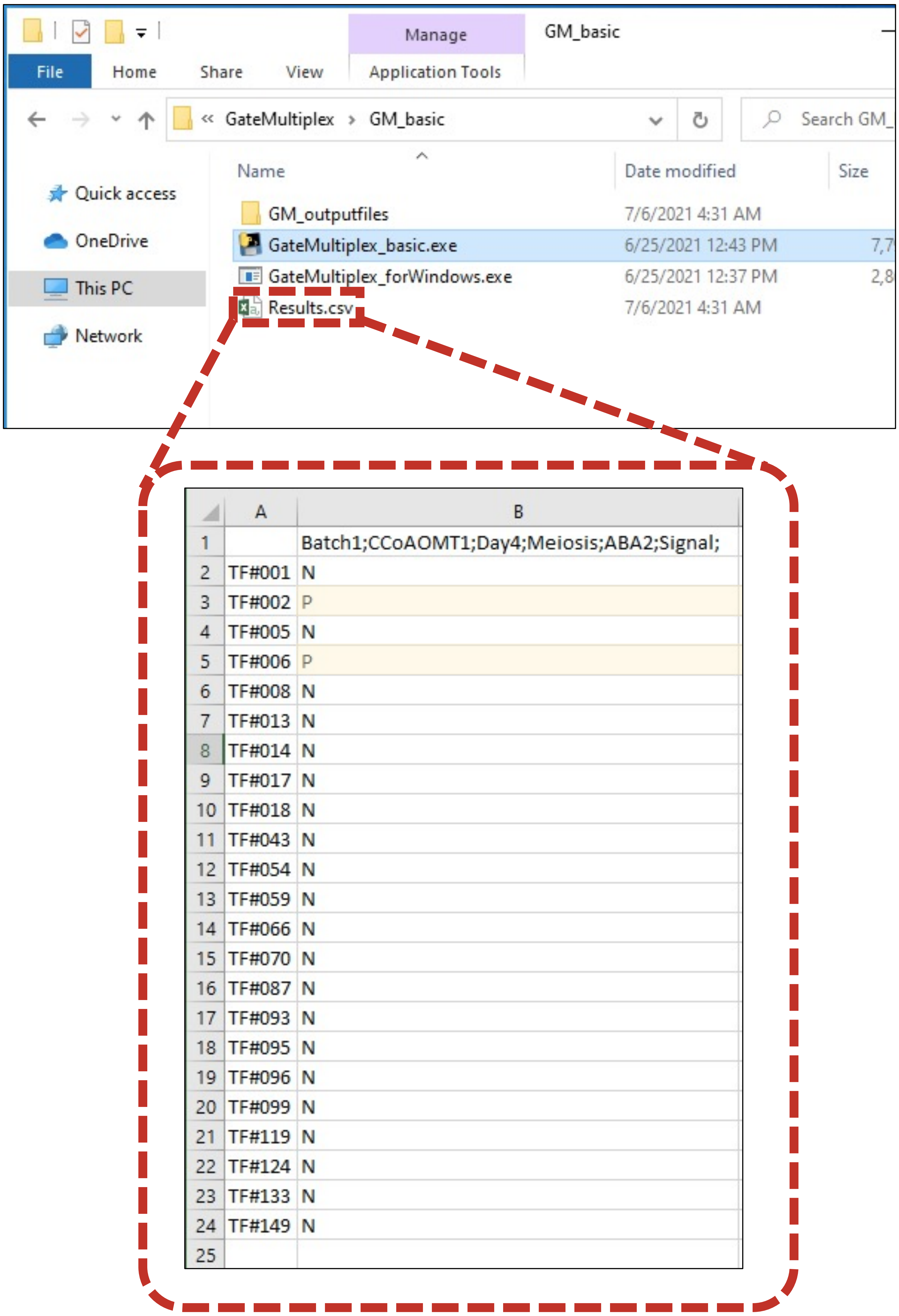

	A	B
1		Batch1;CCoAOMT1;Day4;Meiosis;ABA2;Signal;
2	TF#001	N
3	TF#002	P
4	TF#005	N
5	TF#006	P
6	TF#008	N
7	TF#013	N
8	TF#014	N
9	TF#017	N
10	TF#018	N
11	TF#043	N
12	TF#054	N
13	TF#059	N
14	TF#066	N
15	TF#070	N
16	TF#087	N
17	TF#093	N
18	TF#095	N
19	TF#096	N
20	TF#099	N
21	TF#119	N
22	TF#124	N
23	TF#133	N
24	TF#149	N
25		

- The content of fold change file. Please see Figure L1-L3 for the concept of fold change file.
- The symbol ”nan” means “zero divided by zero”, and the symbol “inf” means “non-zero number divided by zero”. Please the “Symbols in output files” section in Additional file 3.

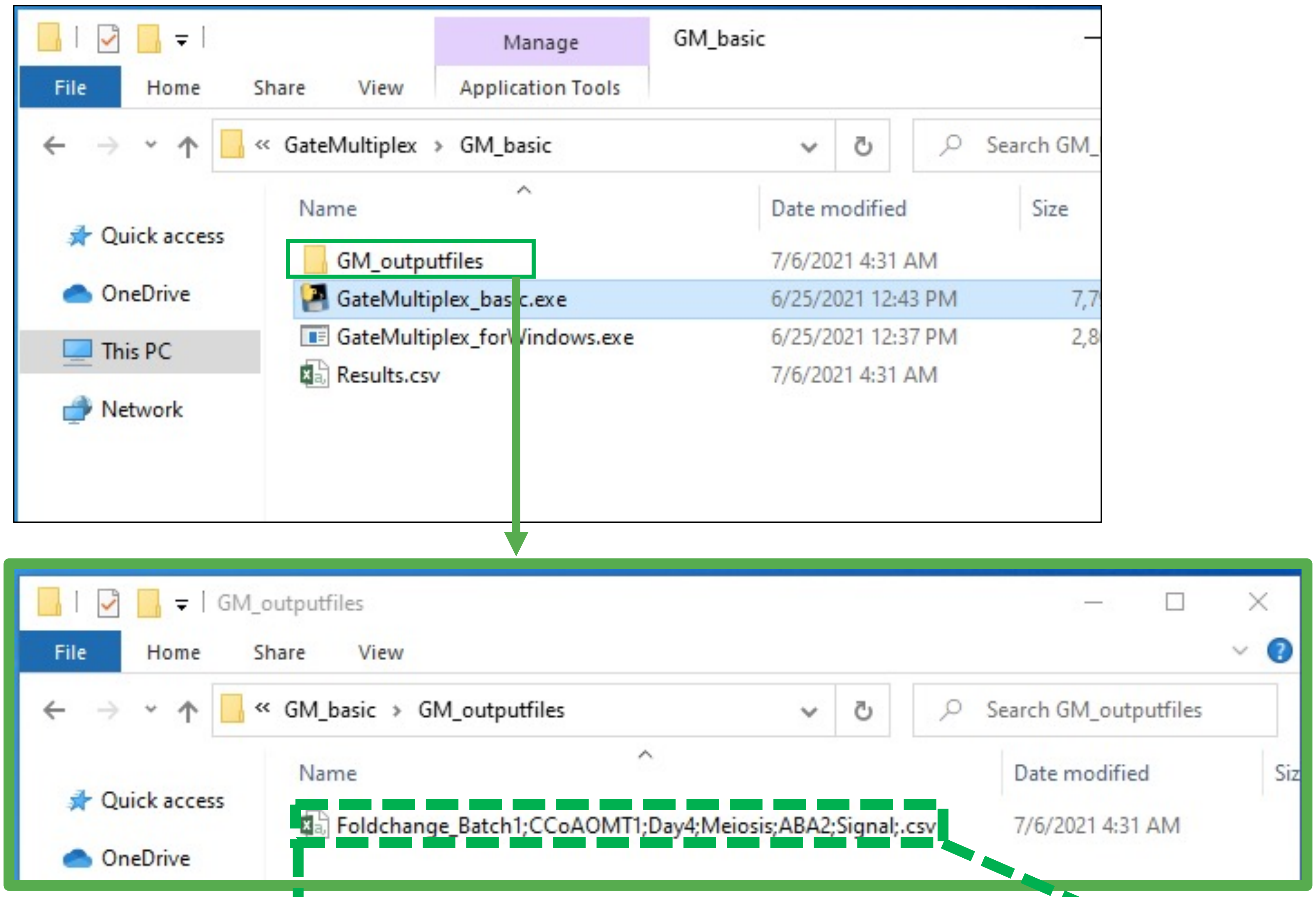

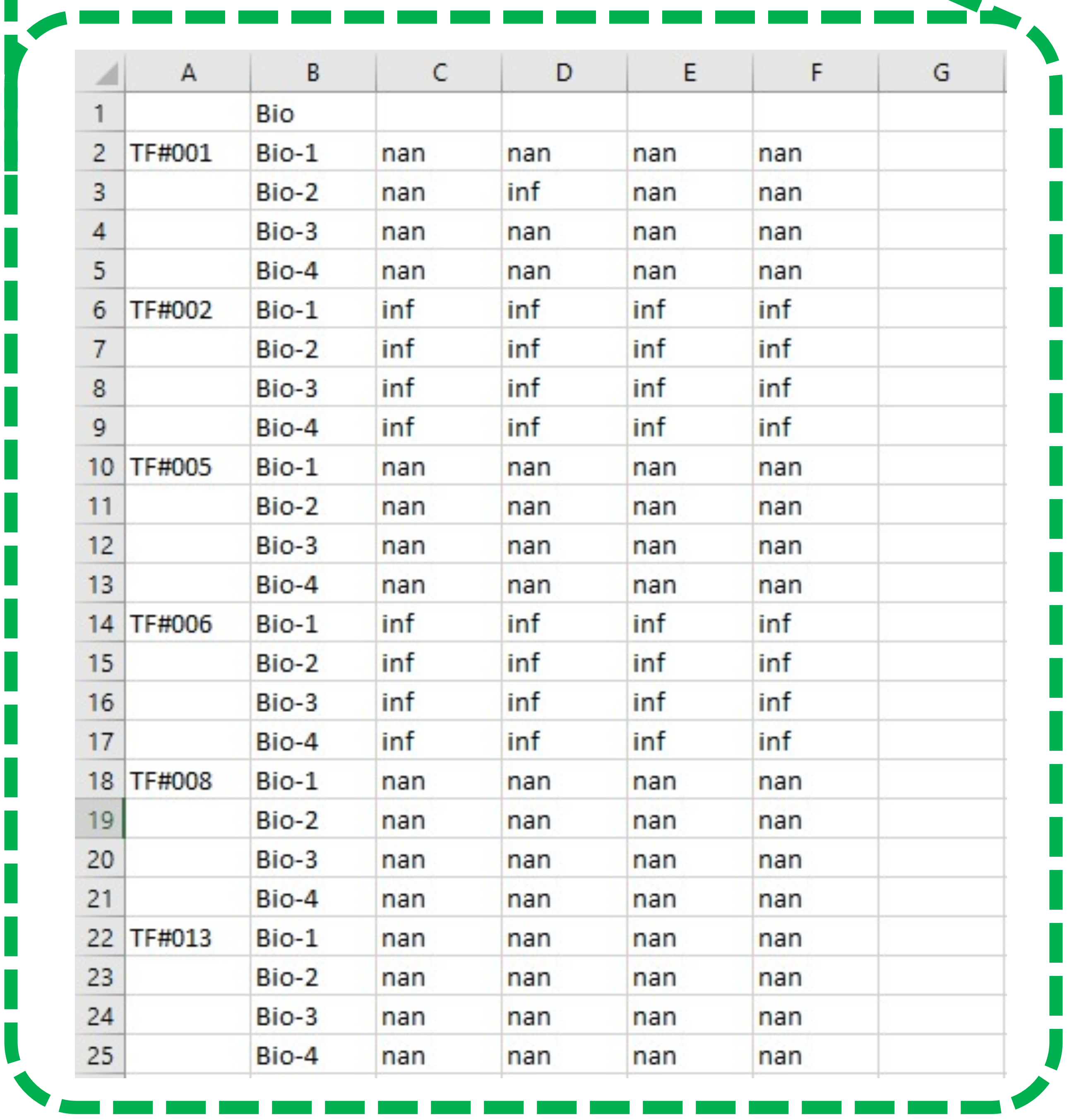

	A	B	C	D	E	F	G
1		Bio					
2	TF#001	Bio-1	nan	nan	nan	nan	
3		Bio-2	nan	inf	nan	nan	
4		Bio-3	nan	nan	nan	nan	
5		Bio-4	nan	nan	nan	nan	
6	TF#002	Bio-1	inf	inf	inf	inf	
7		Bio-2	inf	inf	inf	inf	
8		Bio-3	inf	inf	inf	inf	
9		Bio-4	inf	inf	inf	inf	
10	TF#005	Bio-1	nan	nan	nan	nan	
11		Bio-2	nan	nan	nan	nan	
12		Bio-3	nan	nan	nan	nan	
13		Bio-4	nan	nan	nan	nan	
14	TF#006	Bio-1	inf	inf	inf	inf	
15		Bio-2	inf	inf	inf	inf	
16		Bio-3	inf	inf	inf	inf	
17		Bio-4	inf	inf	inf	inf	
18	TF#008	Bio-1	nan	nan	nan	nan	
19		Bio-2	nan	nan	nan	nan	
20		Bio-3	nan	nan	nan	nan	
21		Bio-4	nan	nan	nan	nan	
22	TF#013	Bio-1	nan	nan	nan	nan	
23		Bio-2	nan	nan	nan	nan	
24		Bio-3	nan	nan	nan	nan	
25		Bio-4	nan	nan	nan	nan	

Yeast one-hybrid (Y1H) for TF-DNA interaction (GM_Converter + GM_Advanced)

Yeast one-hybrid (Y1H) for TF-DNA interaction (GM_Converter + GM_Advanced)

- *The provided demo data is from our previous study (PMID: 31186303, 2019 Genome Research). Due to the different version of PhenoBooth, the output values are different and thus the analyzed results might be different to the previous results.*

- The location of "Y1H_2_for GM_Advanced_demo" data.

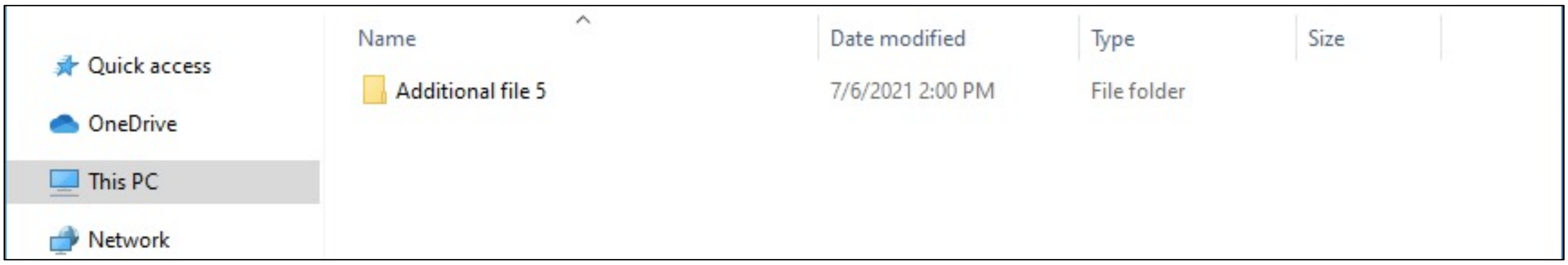

Open the "Additional file 5" folder

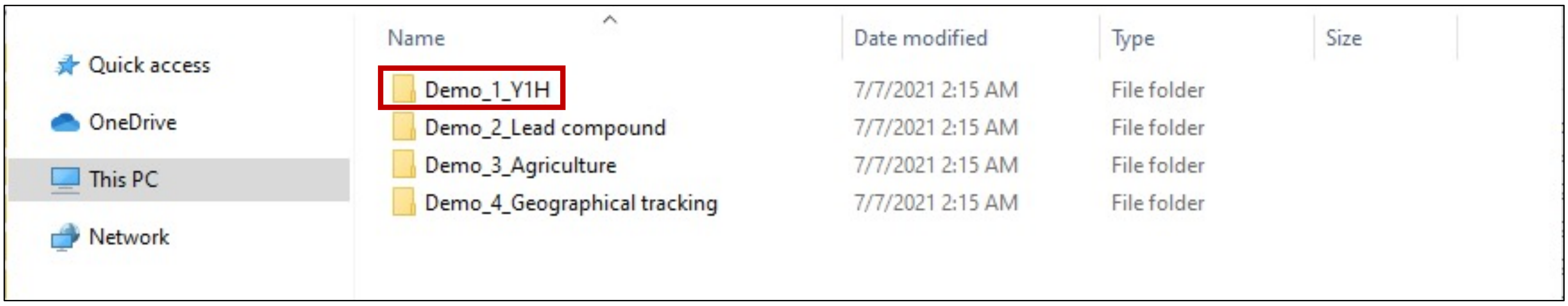

Open the "Demo_1_Y1H" folder (red frame)

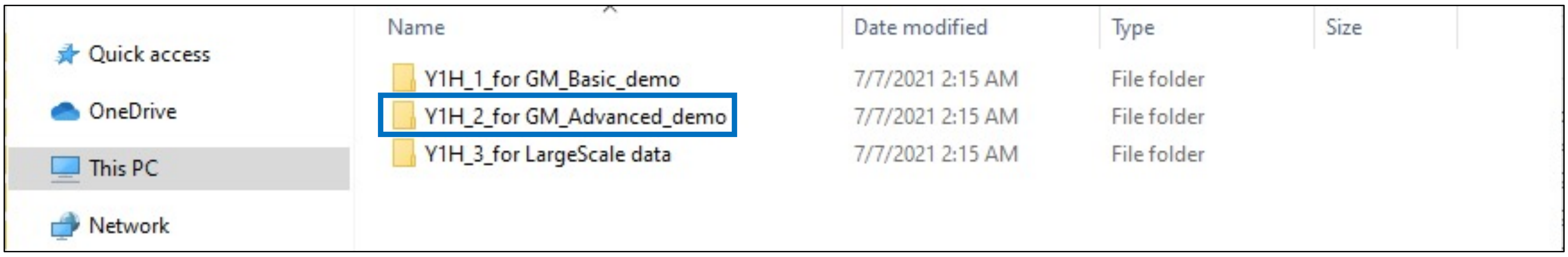

Open the "Y1H_2_for GM_Advanced_demo" folder (blue frame)

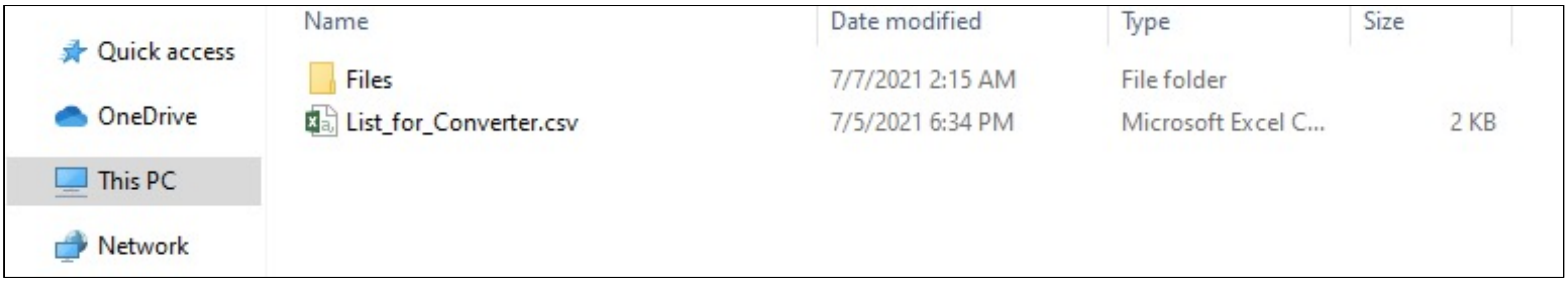

*The "Files" folder and "List_for_Converter.csv" are the demo data for "Y1H_2_for GM_Advanced_demo".

❖ Y1H (GM_Converter)

- The demo data of GM_Advanced in Y1H is stored in “Y1H_1_for GM_Advanced_demo” (green frame) folder of “Demo_1_Y1H” folder (red frame).

- Please see the Figure A1-A11 in Additional file 2 for the detailed concept of GM_Converter.

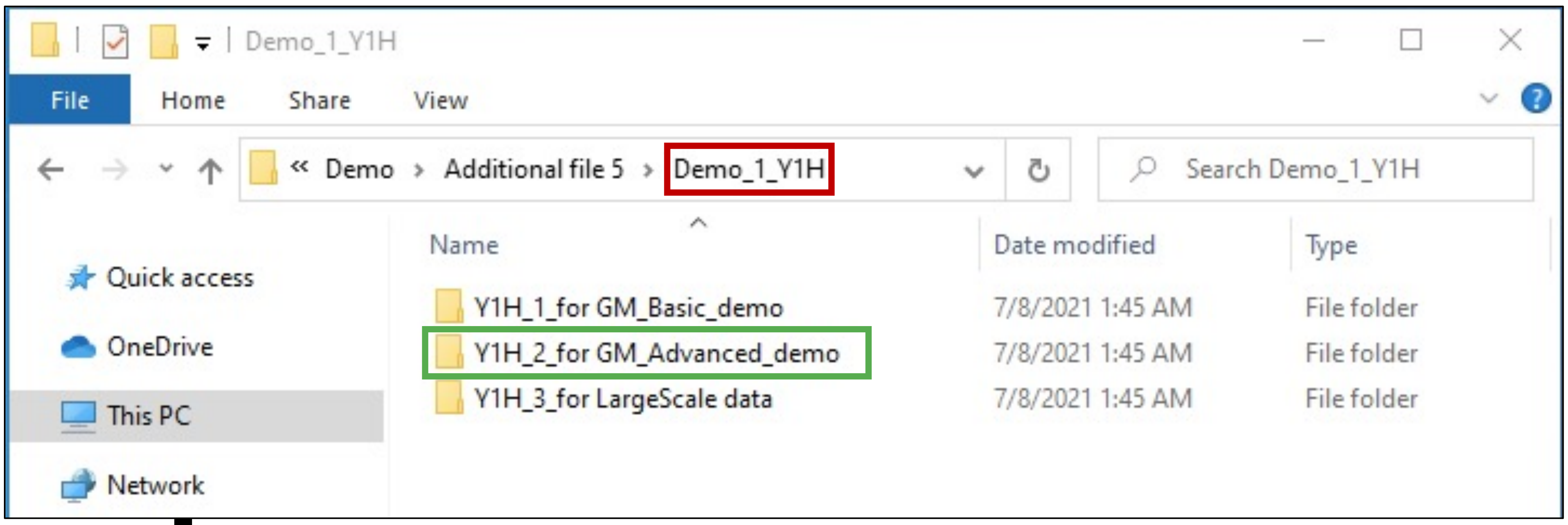

Open the “Y1H_2_for GM_Advanced_demo” folder (green frame)

- All SampleName files, Treatment files and Signal files need to be stored in one folder (in purple frame).

- Only the provided Signal file is the raw data. The other files, such as SampleName files and Treatment files should be provided by users (Here, the prepared SampleName files and Treatment files are provided).

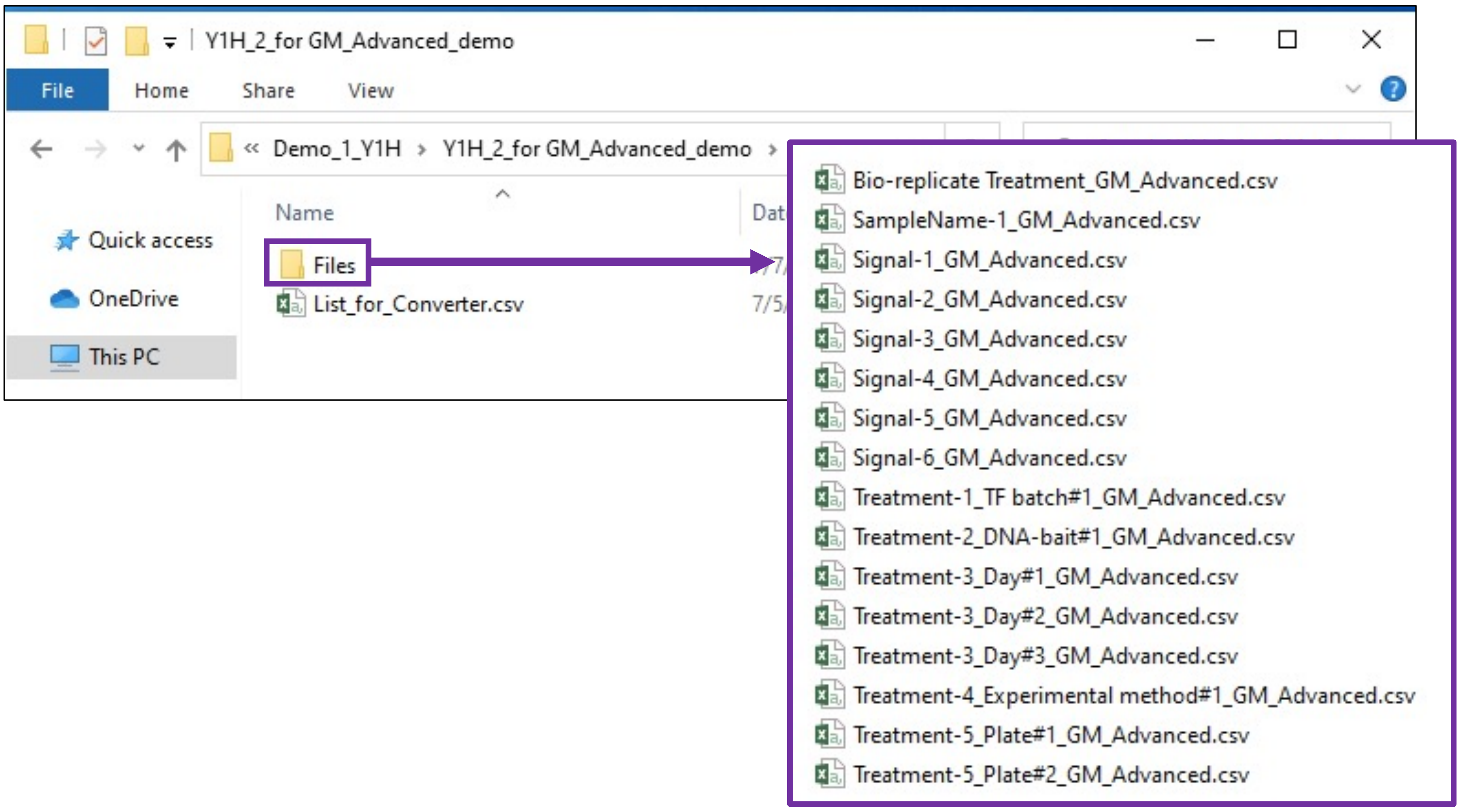

- Files in “Files” (Additional file 5 > Demo_1_Y1H > Y1H_2_for GM_Advanced_demo > Files) folder for converter (Y1H_GM_Advanced).
- Files circled by red frames are the additional Treatment files compared to the files “Y1H_1_for GM_Basic_demo”

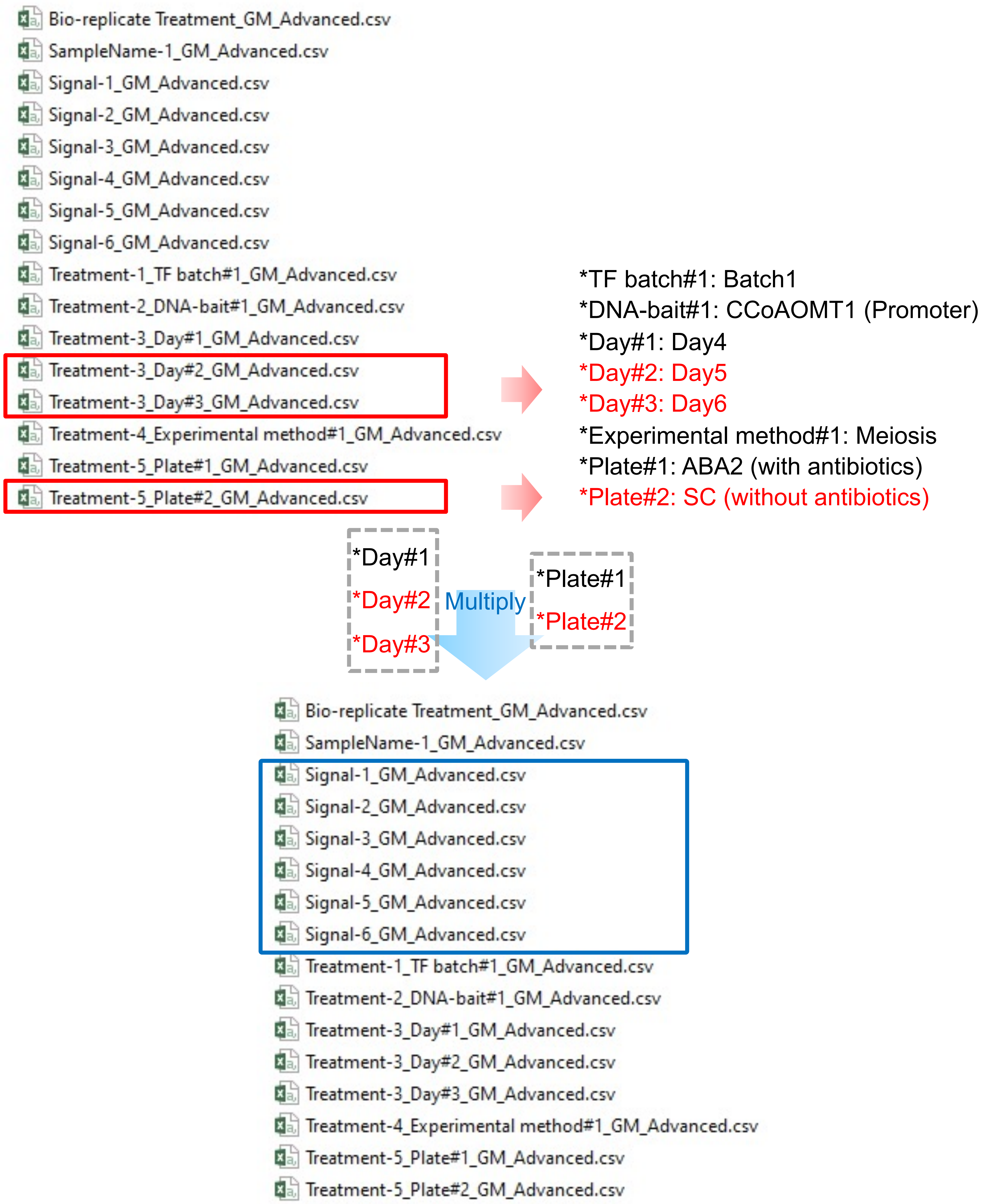

List_for_Converter.csv”

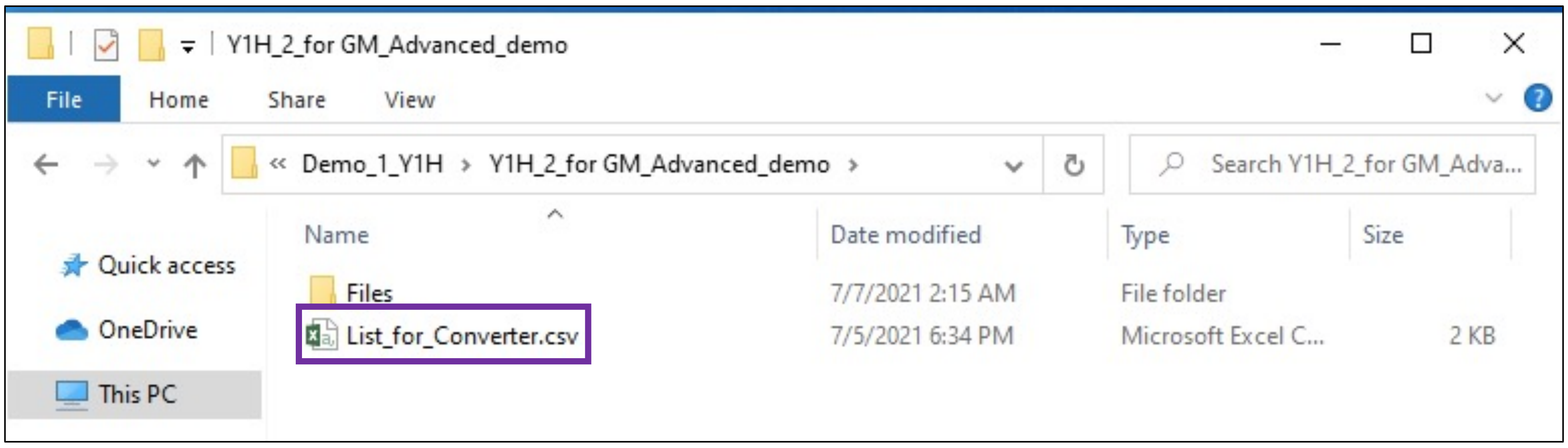

- The data in the first row of “Converted_data.csv” file (the result file output from GM_Converter) is from the first row of “List_for_Converter” file (circled by red frame). Please see the Figure A1-A11 in Additional file 2 for the detailed concept of GM_Converter.

- The data of files listed in the same row of ”List_for_Converter.csv” (take the third row circled by blue frame for example) will be integrated. Please see the Figure A1-A11 in Additional file 2 for the detailed concept of GM_Converter.

“Converted_data.csv”

First row →

	A	B	C	D	E	F	G	H
1	SampleName_TF-prey	Treatment-1_TF batch	Treatment-2_DNA-bait	Treatment-3_Day	Treatment-4_Experimental method	Treatment-5_Plate	Bio-replicate Treatment_Bio-rep	Signal
2	TF#001	Batch1	CCoAOMT1	Day4	Meiosis	ABA2	Bio-1	0
3	TF#001	Batch1	CCoAOMT1	Day4	Meiosis	ABA2	Bio-1	0
4	TF#002	Batch1	CCoAOMT1	Day4	Meiosis	ABA2	Bio-1	255
5	TF#002	Batch1	CCoAOMT1	Day4	Meiosis	ABA2	Bio-1	302
6	EV	Batch1	CCoAOMT1	Day4	Meiosis	ABA2	Bio-1	0
7	EV	Batch1	CCoAOMT1	Day4	Meiosis	ABA2	Bio-1	0
8	TF#005	Batch1	CCoAOMT1	Day4	Meiosis	ABA2	Bio-1	0
9	TF#005	Batch1	CCoAOMT1	Day4	Meiosis	ABA2	Bio-1	0
10	TF#006	Batch1	CCoAOMT1	Day4	Meiosis	ABA2	Bio-1	67
11	TF#006	Batch1	CCoAOMT1	Day4	Meiosis	ABA2	Bio-1	73
12	TF#008	Batch1	CCoAOMT1	Day4	Meiosis	ABA2	Bio-1	0
13	TF#008	Batch1	CCoAOMT1	Day4	Meiosis	ABA2	Bio-1	0
14	TF#001	Batch1	CCoAOMT1	Day4	Meiosis	ABA2	Bio-2	0
15	TF#001	Batch1	CCoAOMT1	Day4	Meiosis	ABA2	Bio-2	3
16	TF#002	Batch1	CCoAOMT1	Day4	Meiosis	ABA2	Bio-2	614
17	TF#002	Batch1	CCoAOMT1	Day4	Meiosis	ABA2	Bio-2	282

“List_for_Converter.csv”

	A	B	......	H
1	SampleName_TF-prey	Treatment-1_TF batch		Signal
2	SampleName-1_GM_Advanced.csv	Treatment-1_TF batch#1_GM_Advanced.csv		Signal-1_GM_Advanced.csv
3	SampleName-1_GM_Advanced.csv	Treatment-1_TF batch#1_GM_Advanced.csv		Signal-2_GM_Advanced.csv
4	SampleName-1_GM_Advanced.csv	Treatment-1_TF batch#1_GM_Advanced.csv		Signal-3_GM_Advanced.csv
5	SampleName-1_GM_Advanced.csv	Treatment-1_TF batch#1_GM_Advanced.csv		Signal-4_GM_Advanced.csv
6	SampleName-1_GM_Advanced.csv	Treatment-1_TF batch#1_GM_Advanced.csv		Signal-5_GM_Advanced.csv
7	SampleName-1_GM_Advanced.csv	Treatment-1_TF batch#1_GM_Advanced.csv		Signal-6_GM_Advanced.csv
8				

- Operation steps

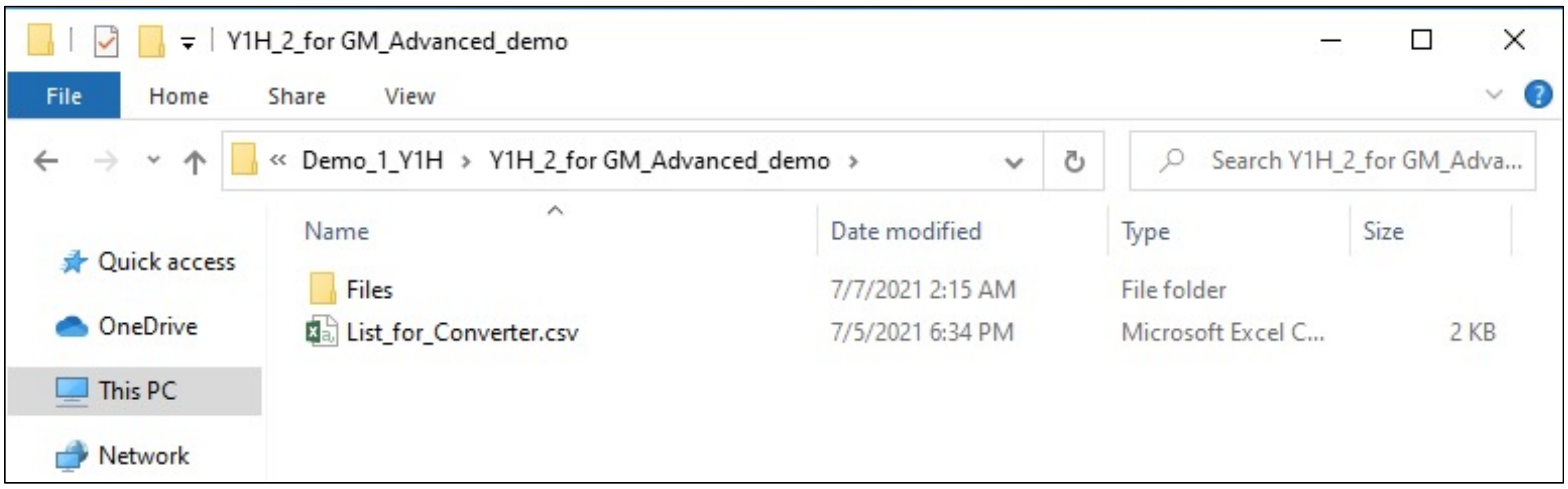

Copy and paste the two executive files of GM_Converter (GateMultiplex_converter.exe and Converter.exe) into "Y1H_1_for GM_Advanced_demo" folder (purple frames and purple arrows)

*The two executive files should be placed in the same folder

- The "List_for_Converter.csv" (indicated by the green arrow) should be placed in the same folder with the executive files of GM_Converter (GateMultiplex_converter.exe and Converter.exe).

.

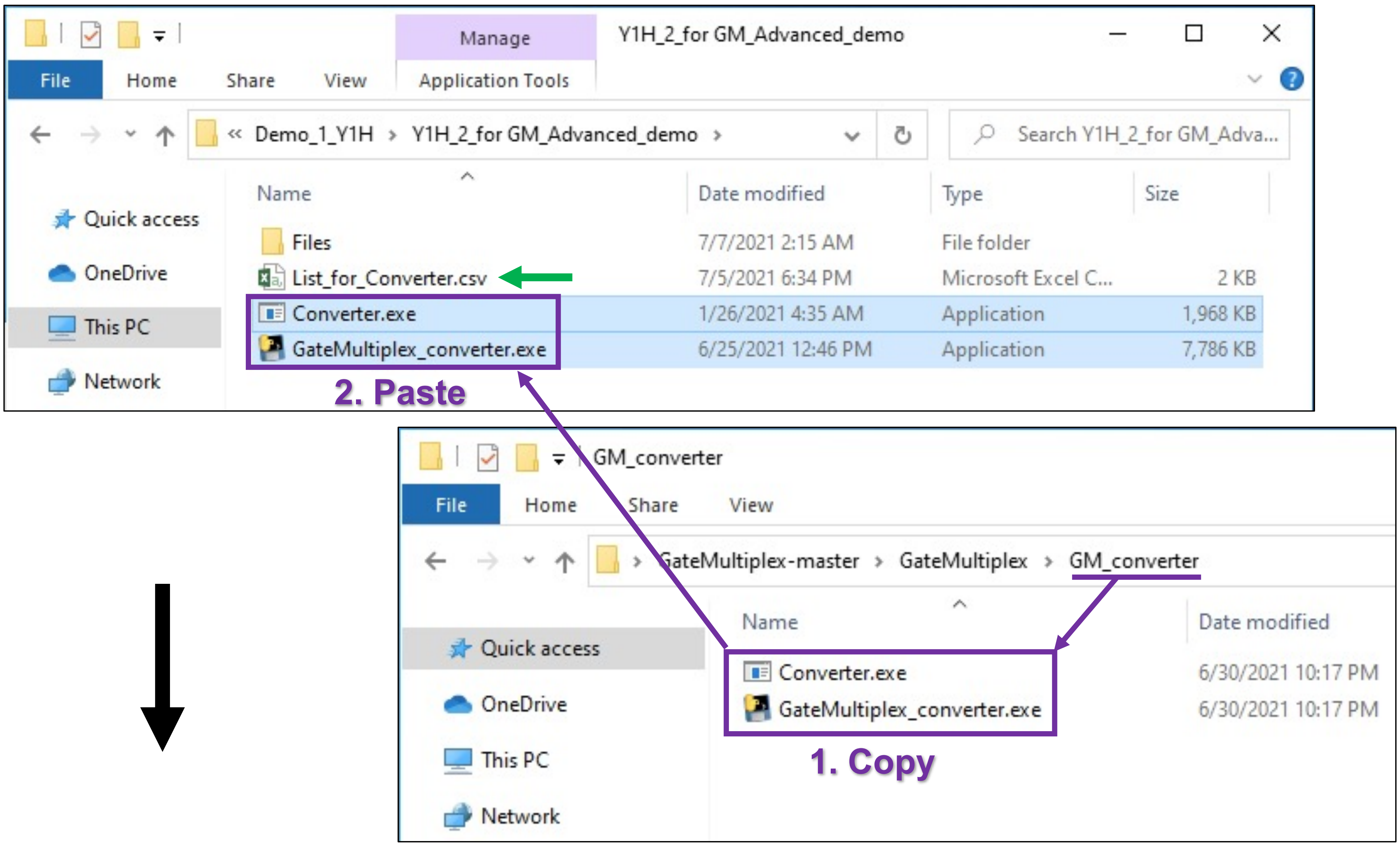

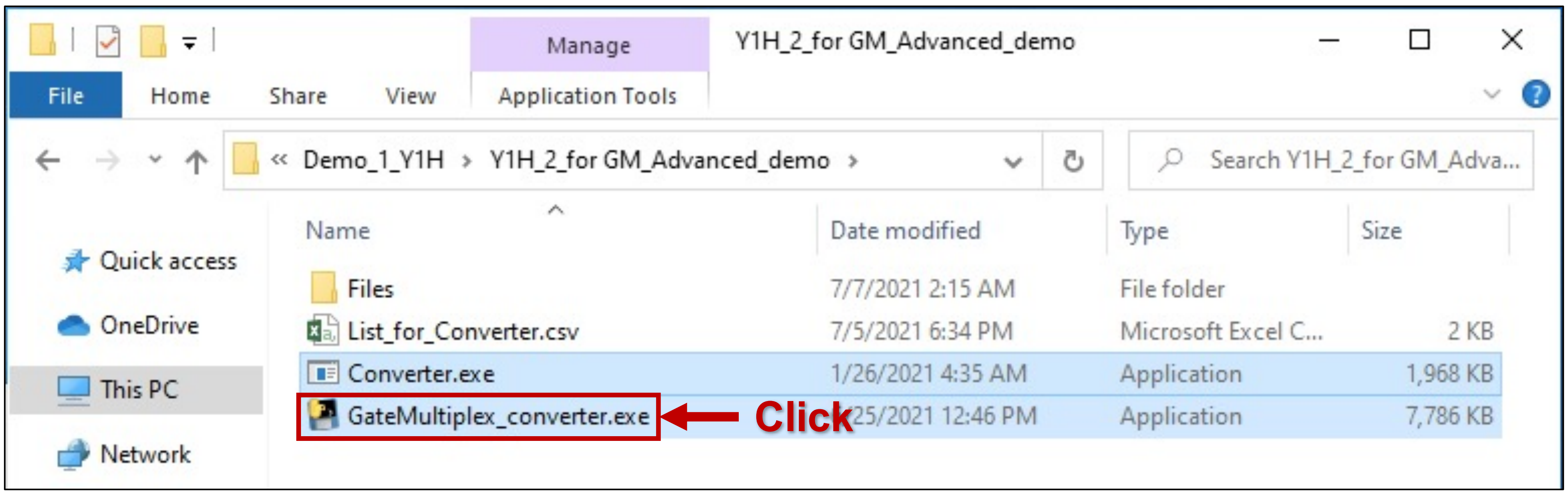

Activate the GM_Converter by a double-clicking on "GateMultiplex_converter.exe" (red frame)

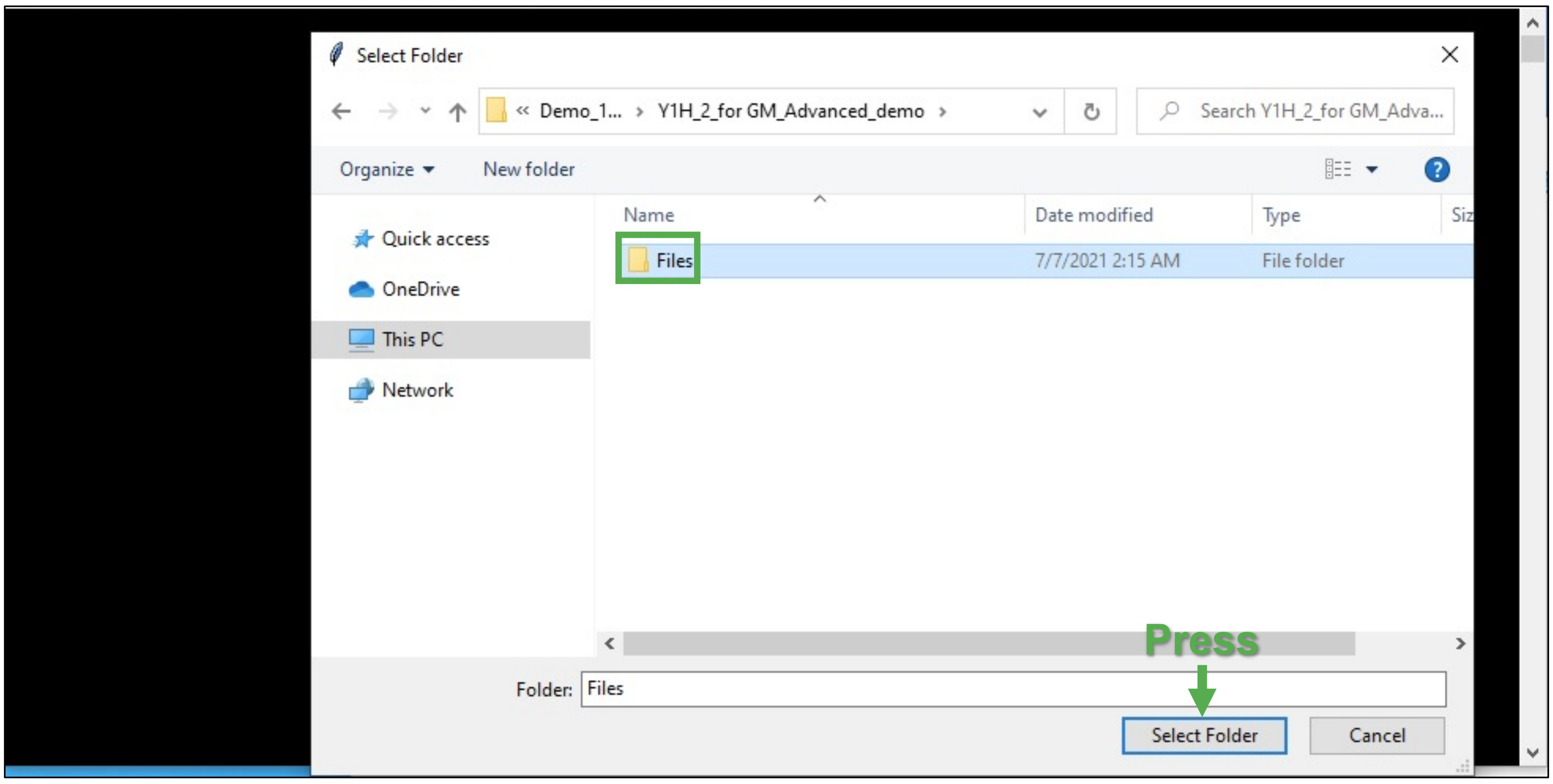

Select the folder "Files" (Additional file 5 > Demo_1_Y1H > Y1H_2_for GM_Advanced_demo > Files) (green frame) and press "Select Folder" (indicated by a green arrow)

- Please see the Figure A5-A11 in Additional file 2 for the detailed parameter setting of GM_Converter.

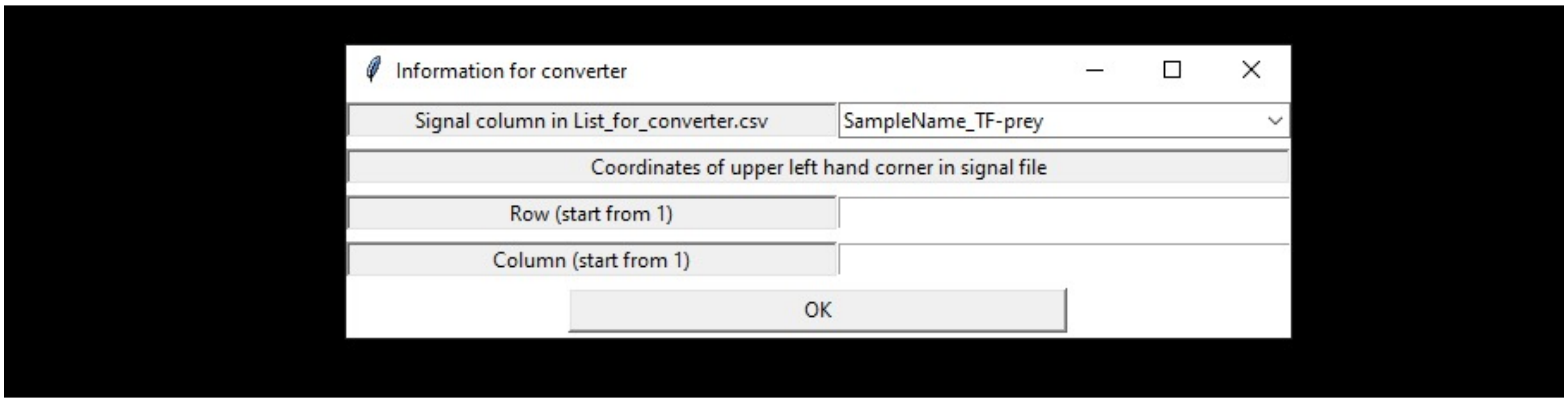

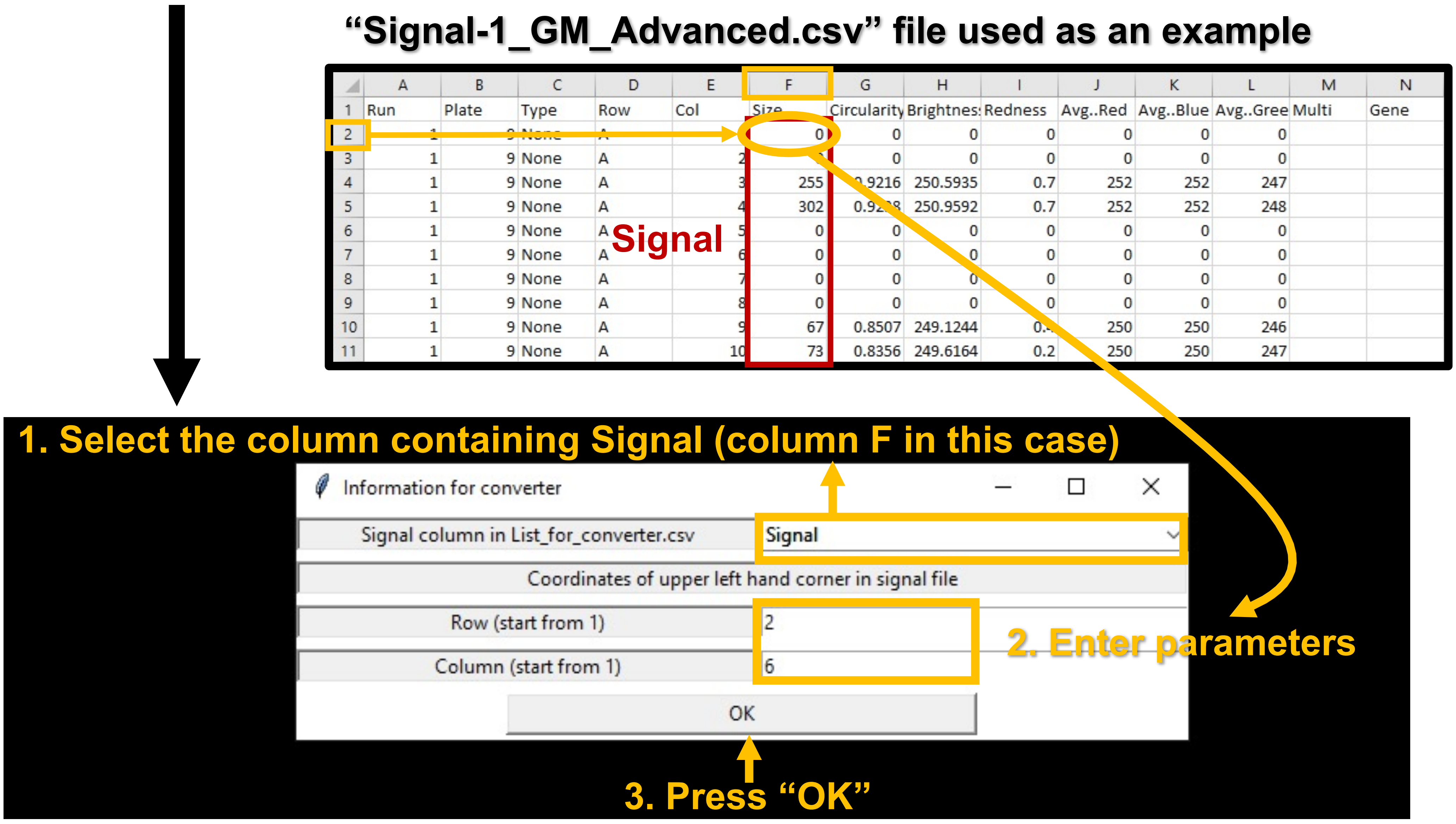

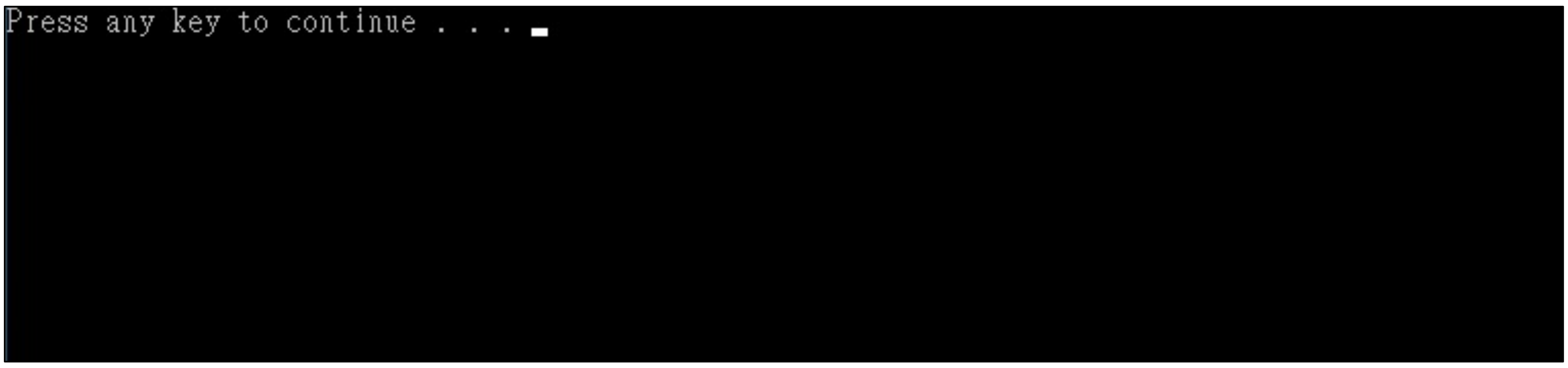

Press any key to close the window

- The converted file “Converted_data.csv” (green frame) is stored in the “GM_converted_data” folder (red frame)

- The title of “Converted_data.csv” (circled by blue frame) is the same as the title in “List_for_Converter.csv”. Please see Figure A5-A11 in Additional file 2.

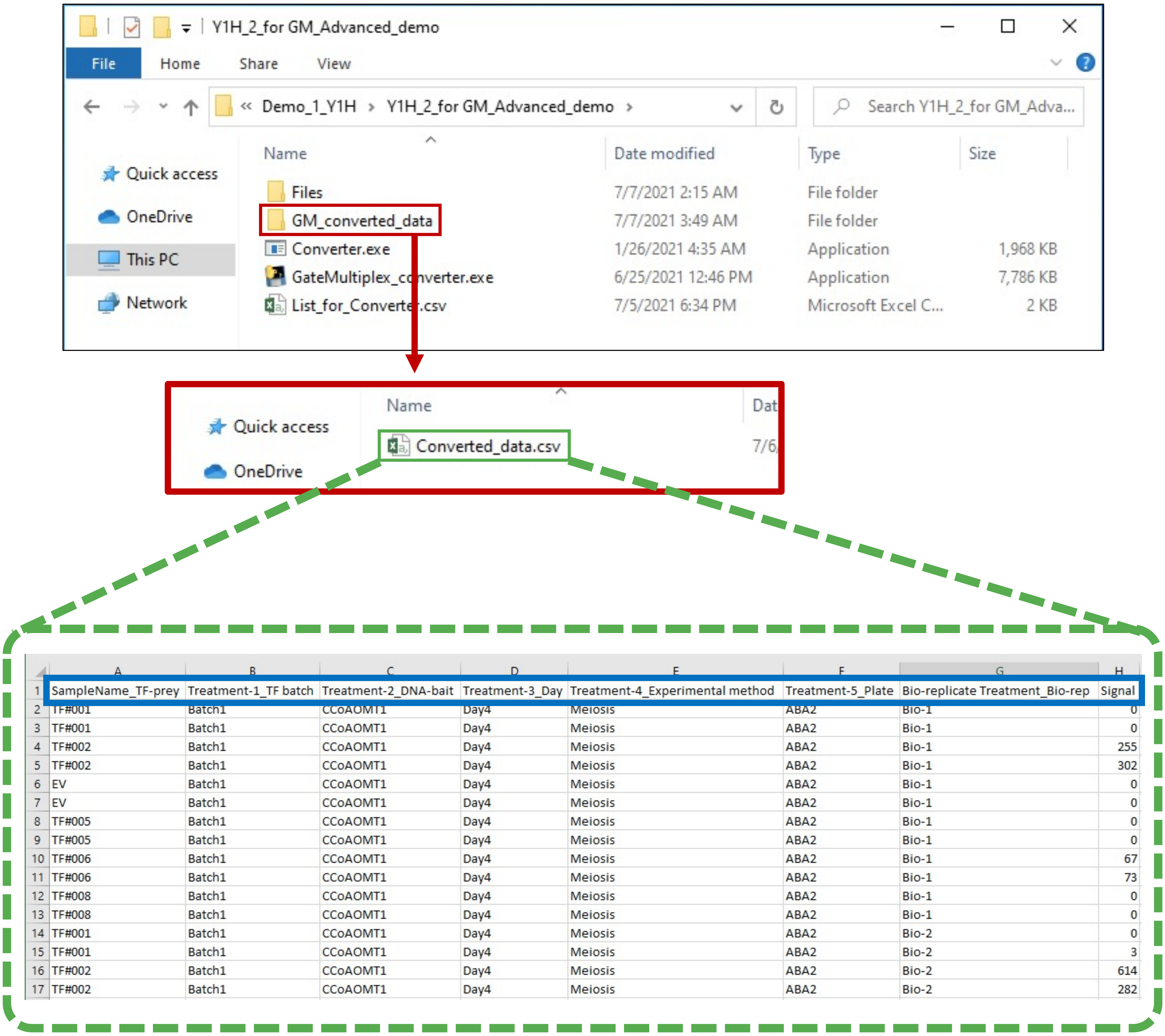

	A	B	C	D	E	F	G	H
1	SampleName_TF-prey	Treatment-1_TF batch	Treatment-2_DNA-bait	Treatment-3_Day	Treatment-4_Experimental method	Treatment-5_Plate	Bio-replicate Treatment_Bio-rep	Signal
2	TF#001	Batch1	CCoAOMT1	Day4	Meiosis	ABA2	Bio-1	0
3	TF#001	Batch1	CCoAOMT1	Day4	Meiosis	ABA2	Bio-1	0
4	TF#002	Batch1	CCoAOMT1	Day4	Meiosis	ABA2	Bio-1	255
5	TF#002	Batch1	CCoAOMT1	Day4	Meiosis	ABA2	Bio-1	302
6	EV	Batch1	CCoAOMT1	Day4	Meiosis	ABA2	Bio-1	0
7	EV	Batch1	CCoAOMT1	Day4	Meiosis	ABA2	Bio-1	0
8	TF#005	Batch1	CCoAOMT1	Day4	Meiosis	ABA2	Bio-1	0
9	TF#005	Batch1	CCoAOMT1	Day4	Meiosis	ABA2	Bio-1	0
10	TF#006	Batch1	CCoAOMT1	Day4	Meiosis	ABA2	Bio-1	67
11	TF#006	Batch1	CCoAOMT1	Day4	Meiosis	ABA2	Bio-1	73
12	TF#008	Batch1	CCoAOMT1	Day4	Meiosis	ABA2	Bio-1	0
13	TF#008	Batch1	CCoAOMT1	Day4	Meiosis	ABA2	Bio-1	0
14	TF#001	Batch1	CCoAOMT1	Day4	Meiosis	ABA2	Bio-2	0
15	TF#001	Batch1	CCoAOMT1	Day4	Meiosis	ABA2	Bio-2	3
16	TF#002	Batch1	CCoAOMT1	Day4	Meiosis	ABA2	Bio-2	614
17	TF#002	Batch1	CCoAOMT1	Day4	Meiosis	ABA2	Bio-2	282

❖ Y1H (GM_Advanced)

- After Converted_data.csv completing, GM_Advanced is further applied for analysis.
- GM_Advanced includes two executive files (GateMultiplex_advanced.exe and GateMultiplex_forWindows.exe), which should be placed in the same folder.
- Activate the GM_Advanced by a double-clicking on “GateMultiplex_advanced.exe” (red frame).

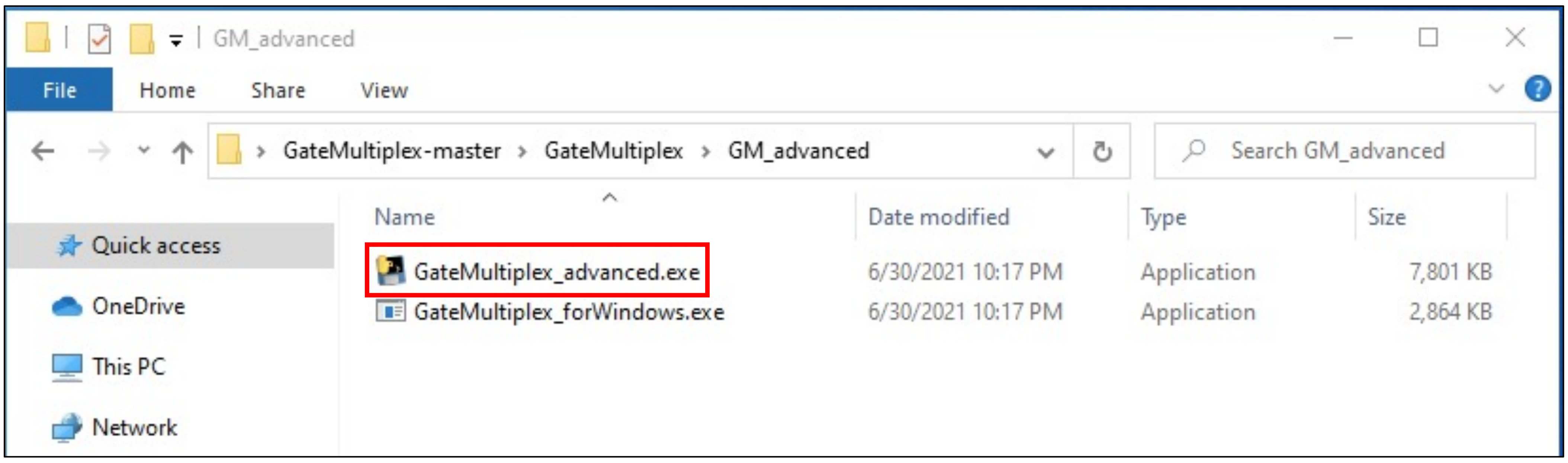

- Select the folder “GM_converted_data” (red frame) and press “Select Folder” (red arrow).
- Directory: Additional file 5 > Demo_1_Y1H > Y1H_2_for GM_Advanced_demo > GM_converted_data

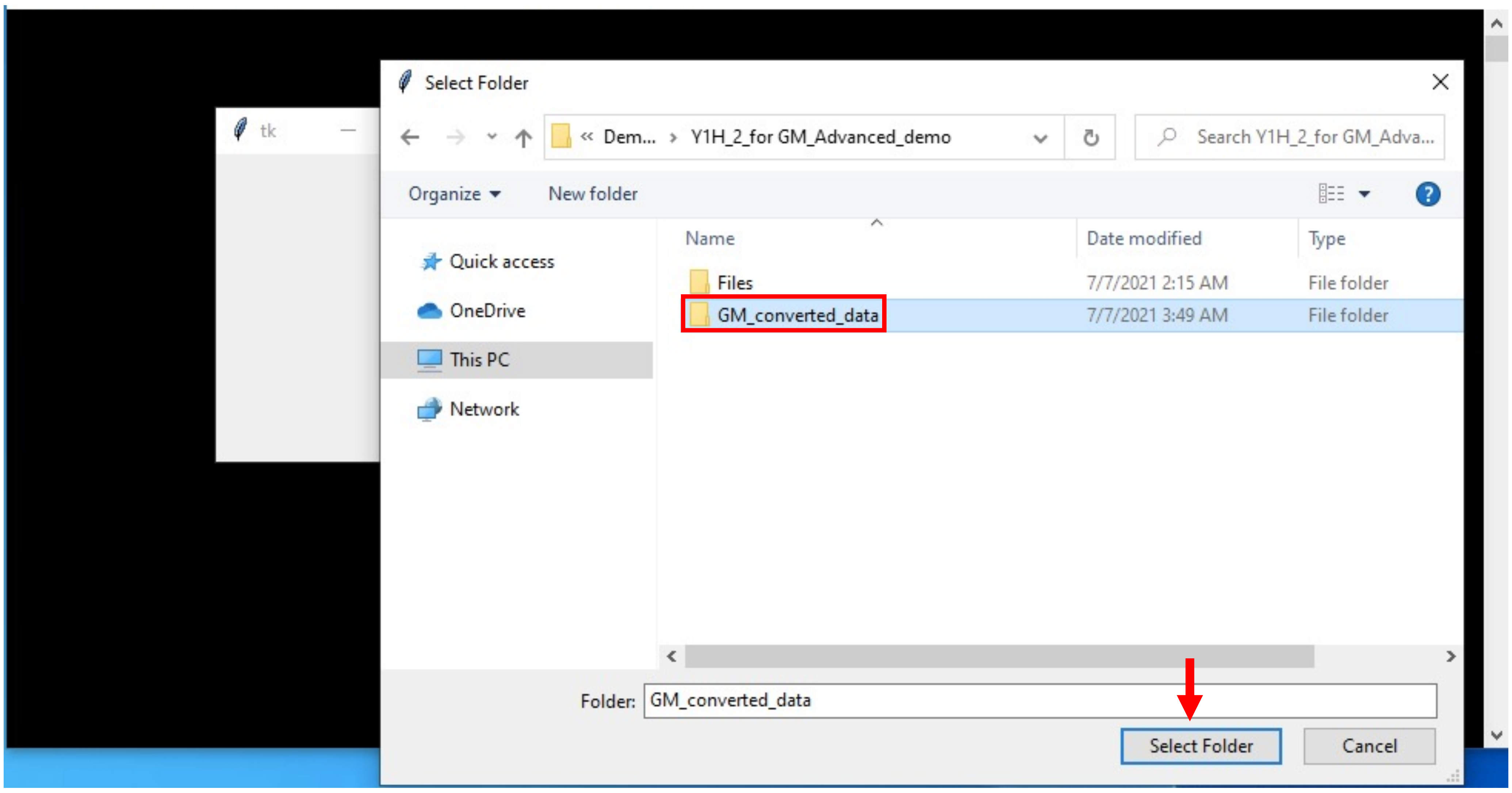

✱ The GUI of GM_Advanced

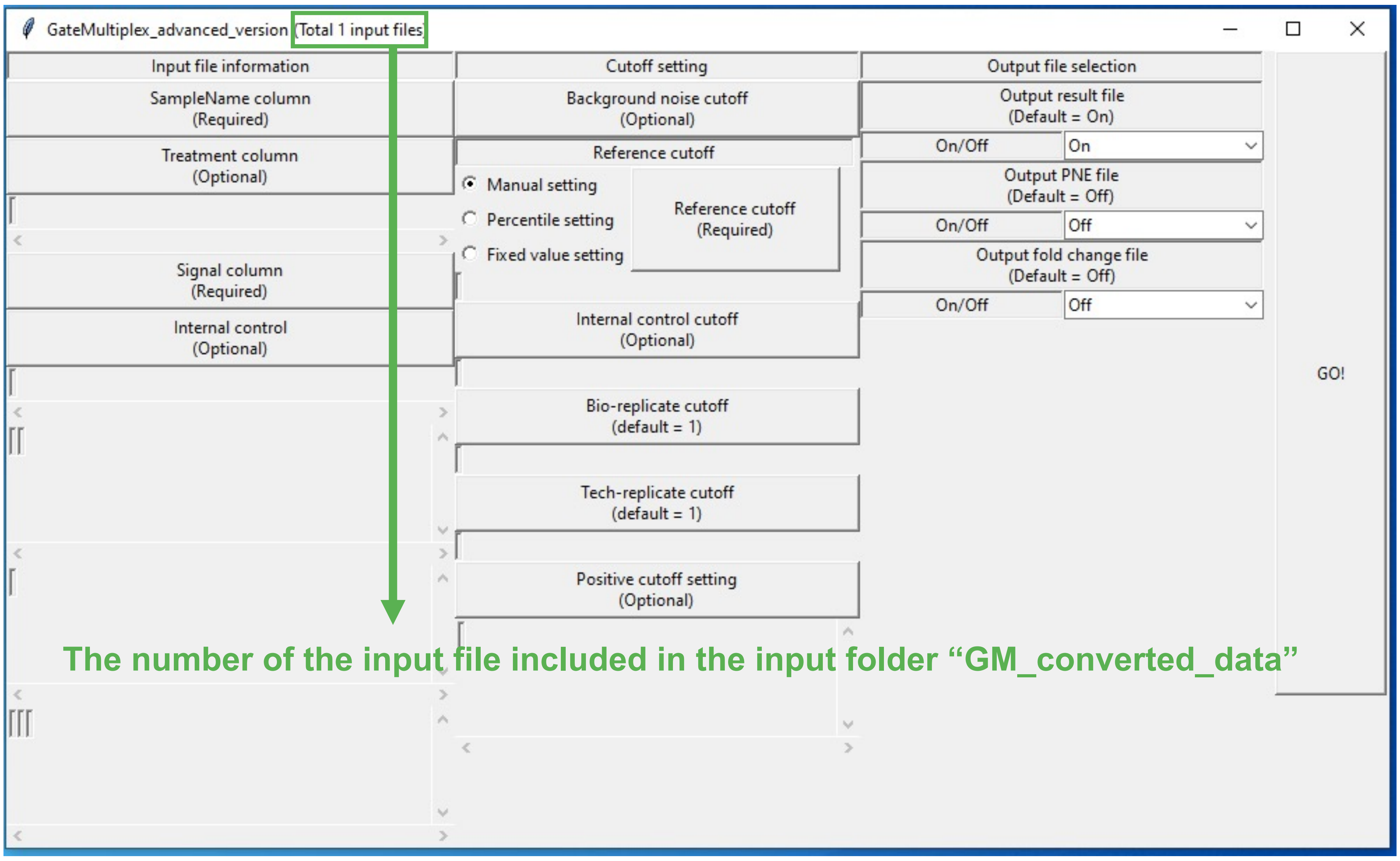

✱ SampleName selection

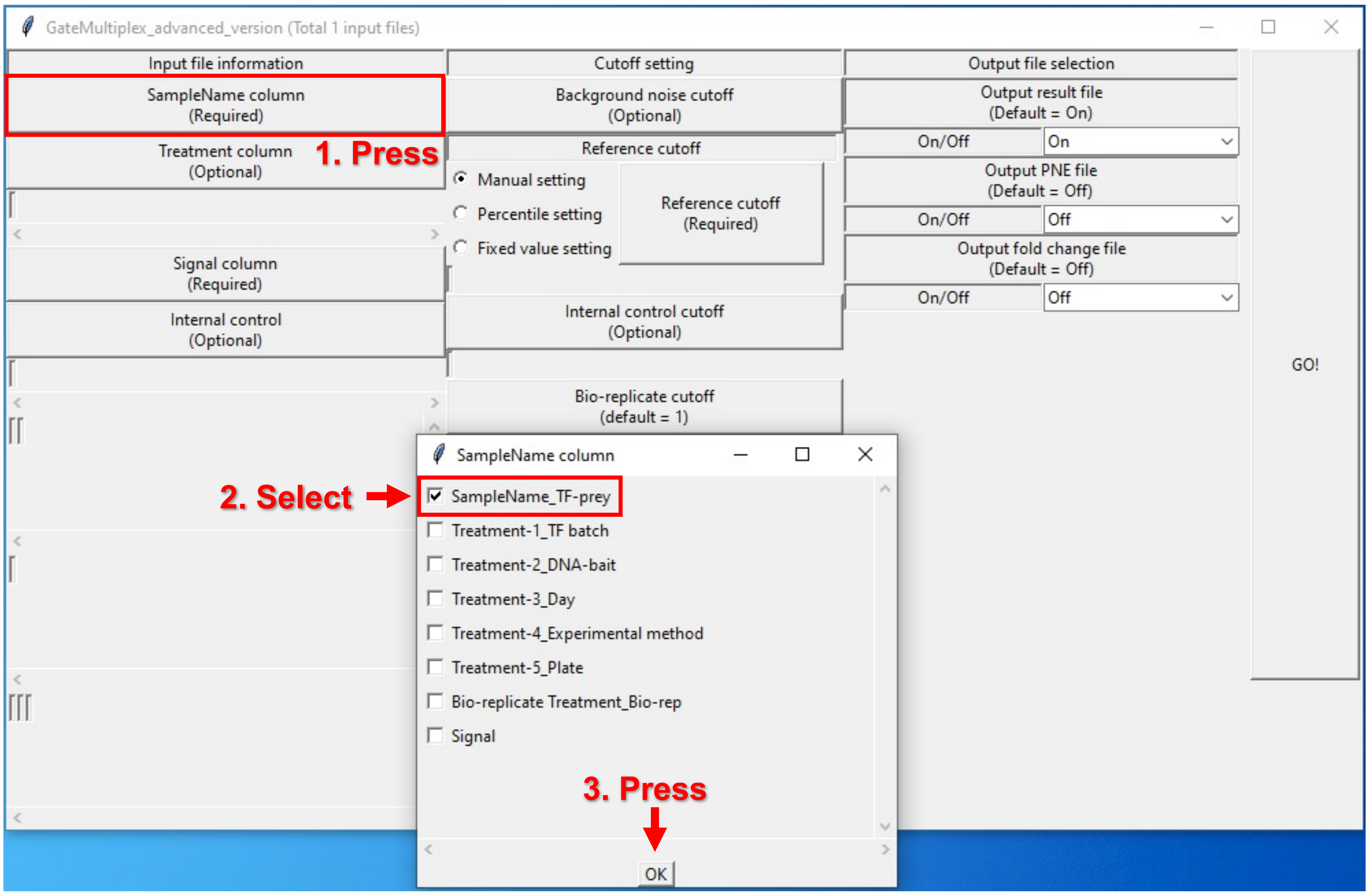

✱ Treatment selection

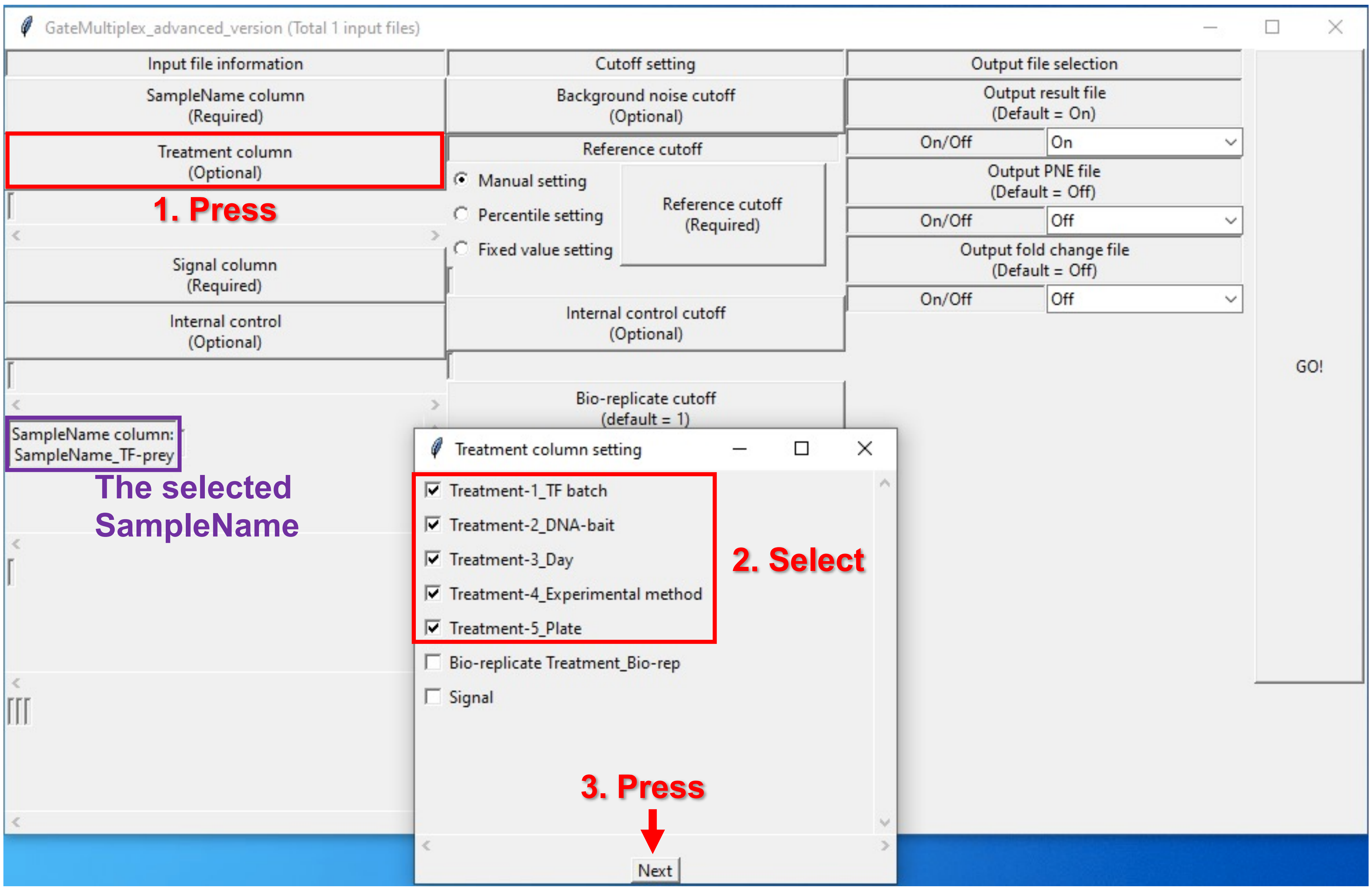

✱ Bio-replicate Treatment selection
(Please see Figure B2 in Additional file 2 for the concept of Bio-replicate Treatment)

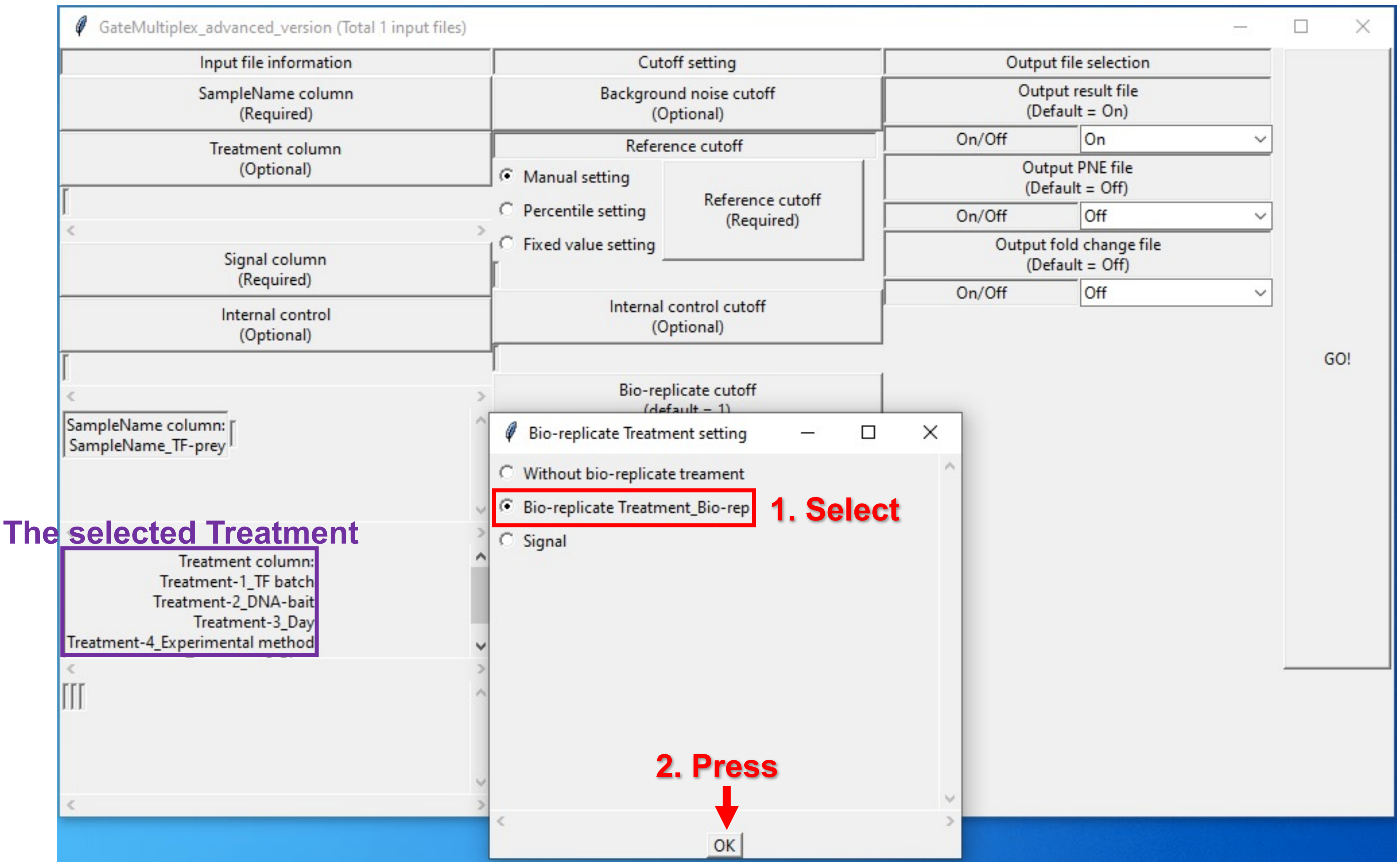

✱ **Signal selection**

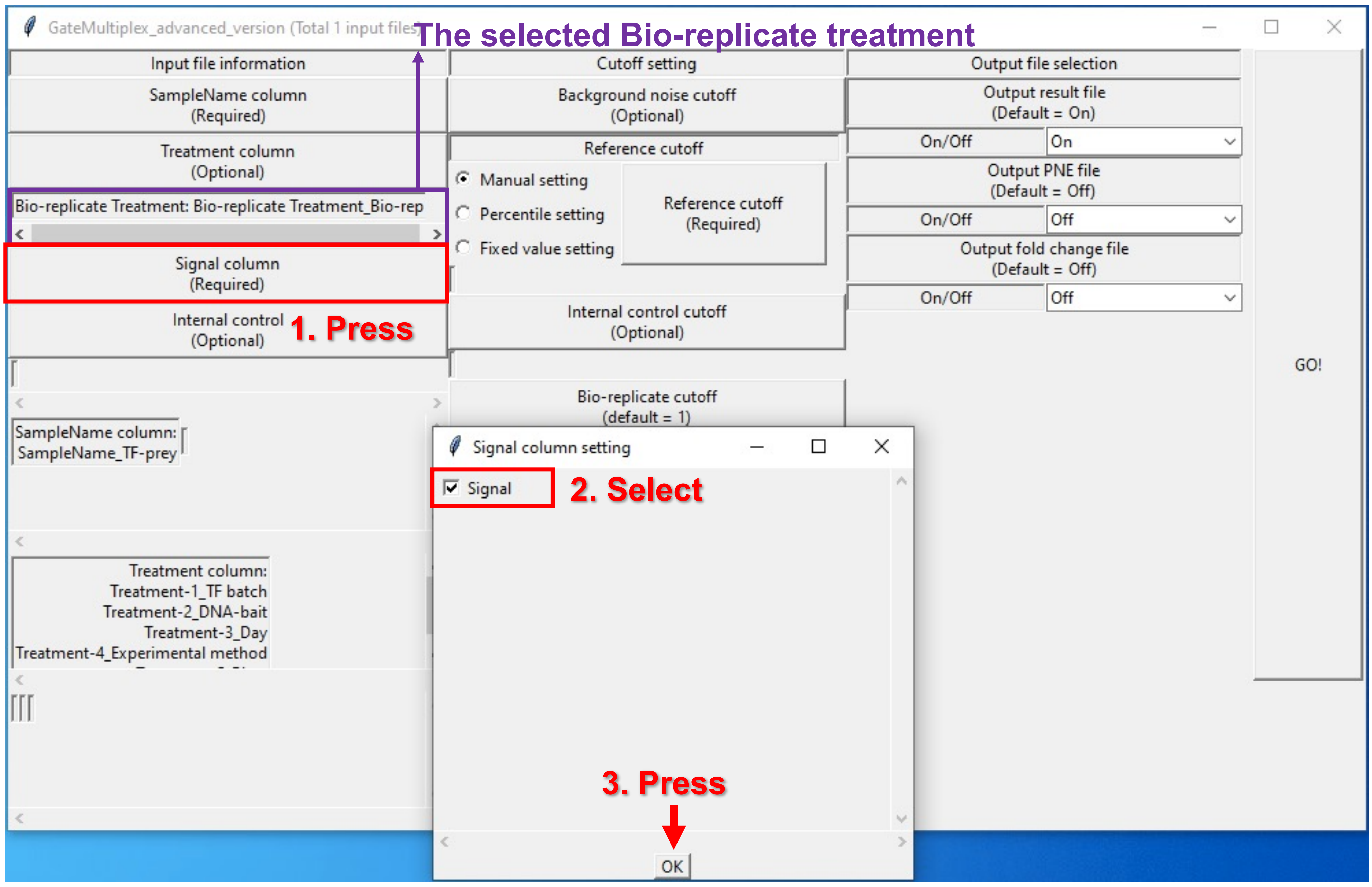

✱ **Internal control “Treatment” and “Condition” setting**

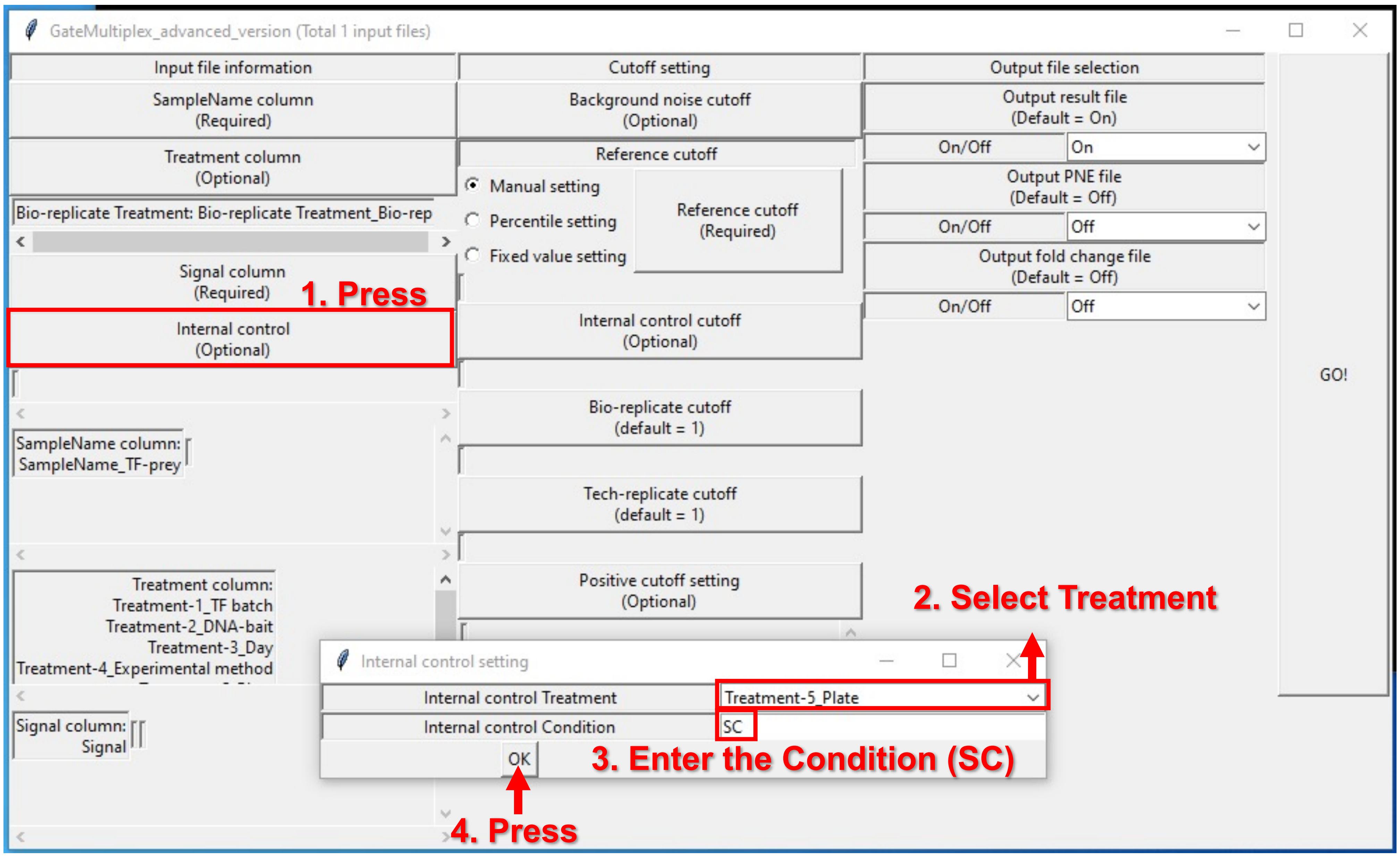

✱Please see Additional file 2 Figure. D1 for the detail explanation of the internal control (here, the “Condition” and “Treatment” setting)

✱ Background noise cutoff setting

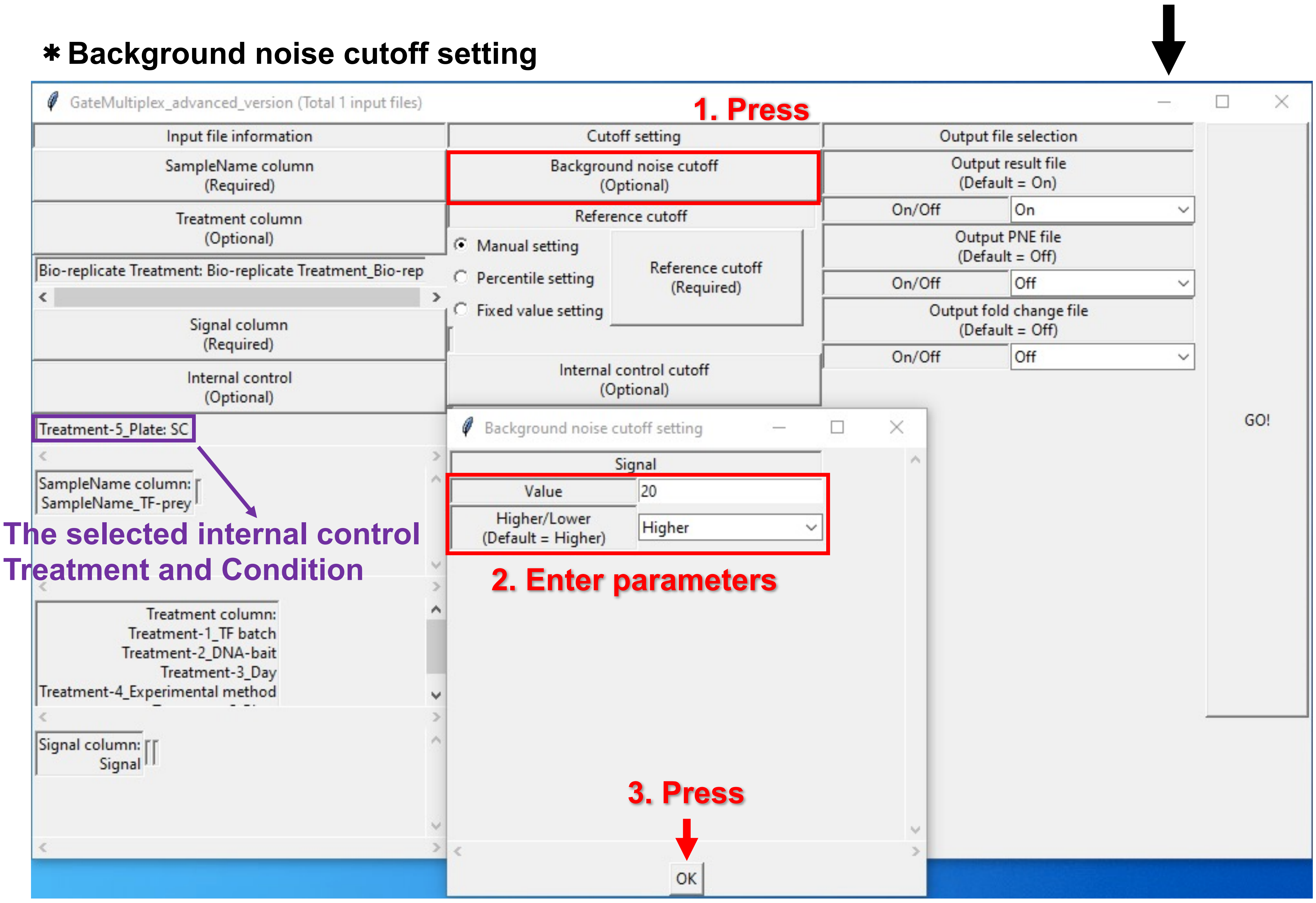

✱ Reference cutoff setting

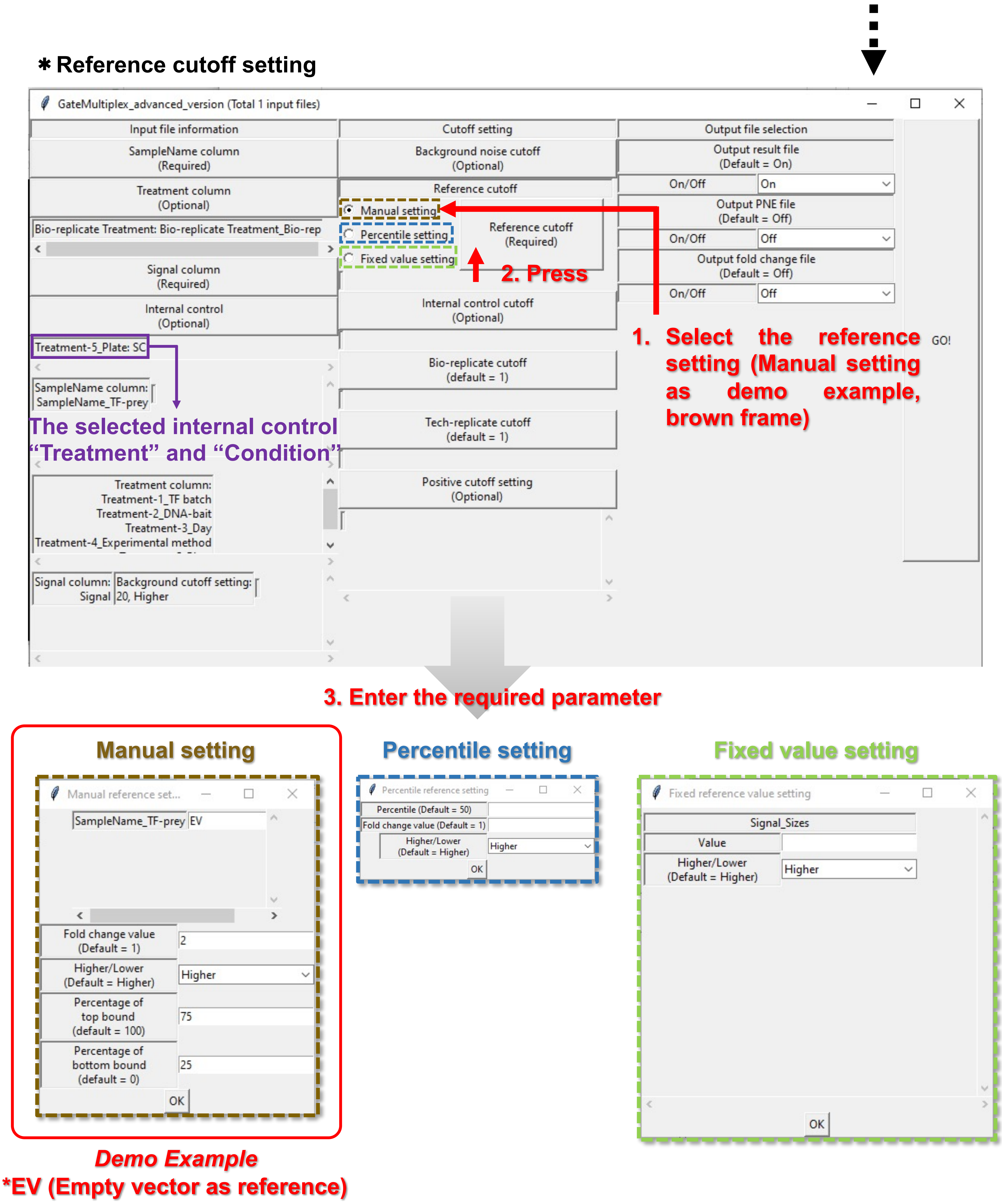

✱ Internal control cutoff setting

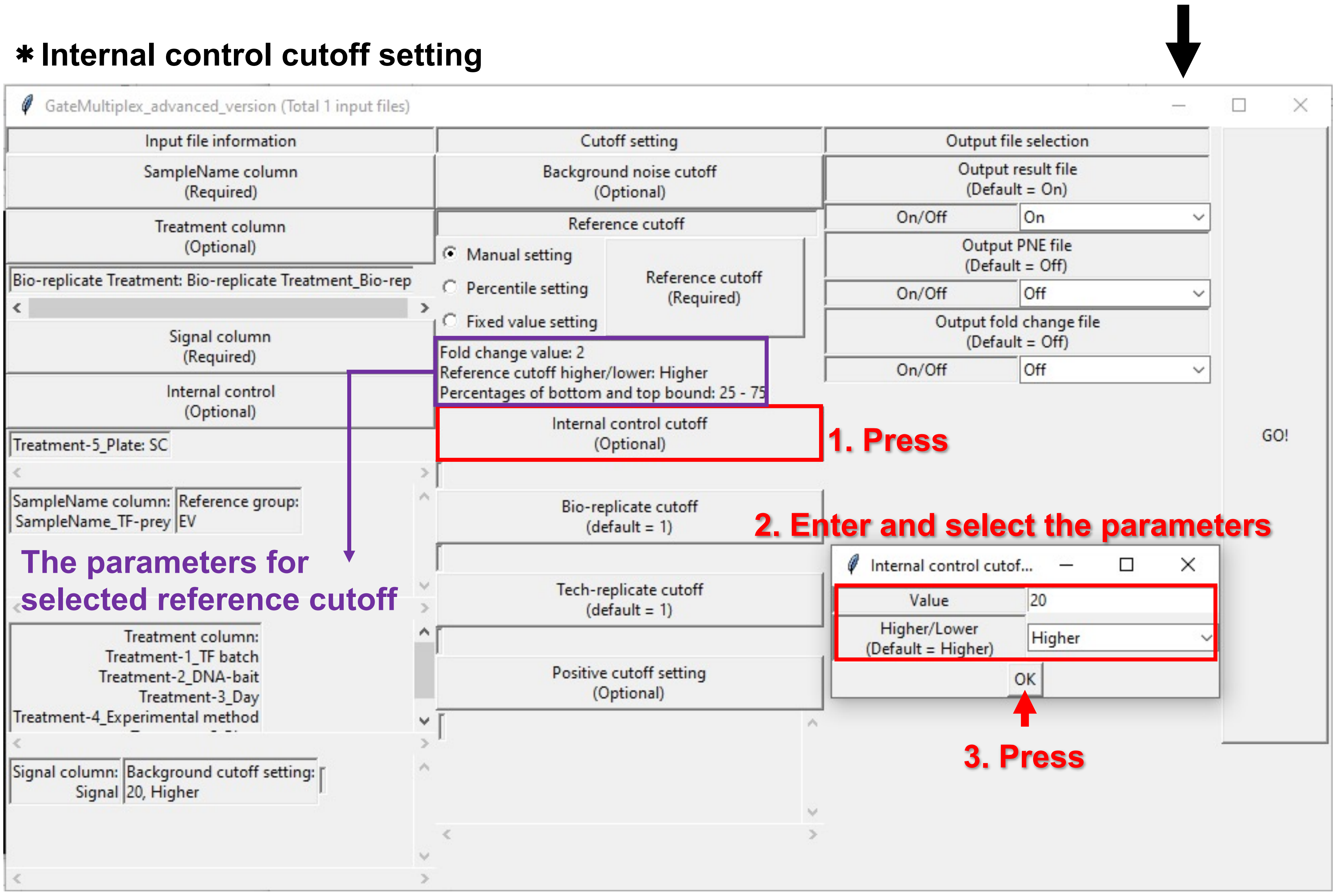

✱ Bio-replicate cutoff setting

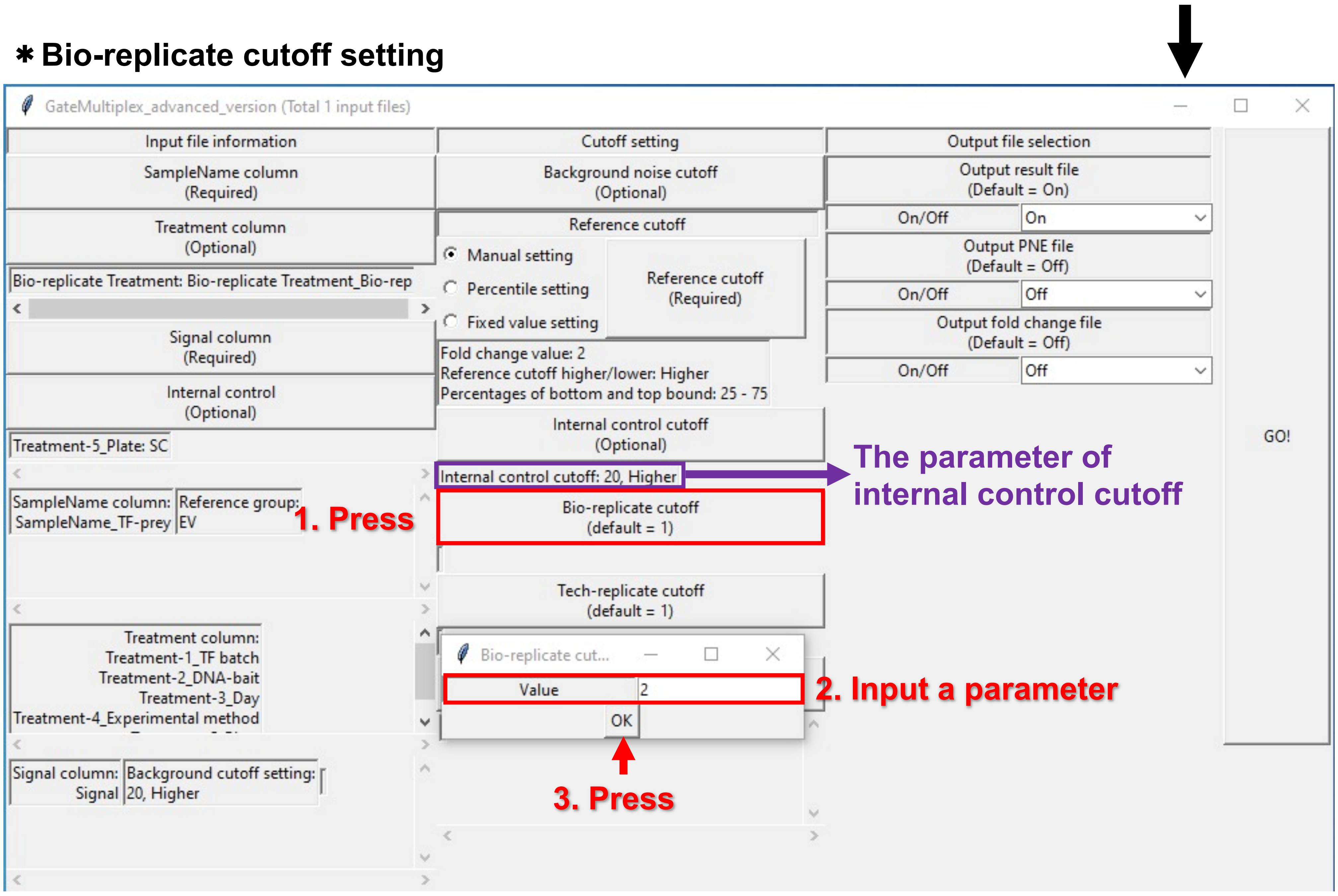

✱ Tech-replicate cutoff setting

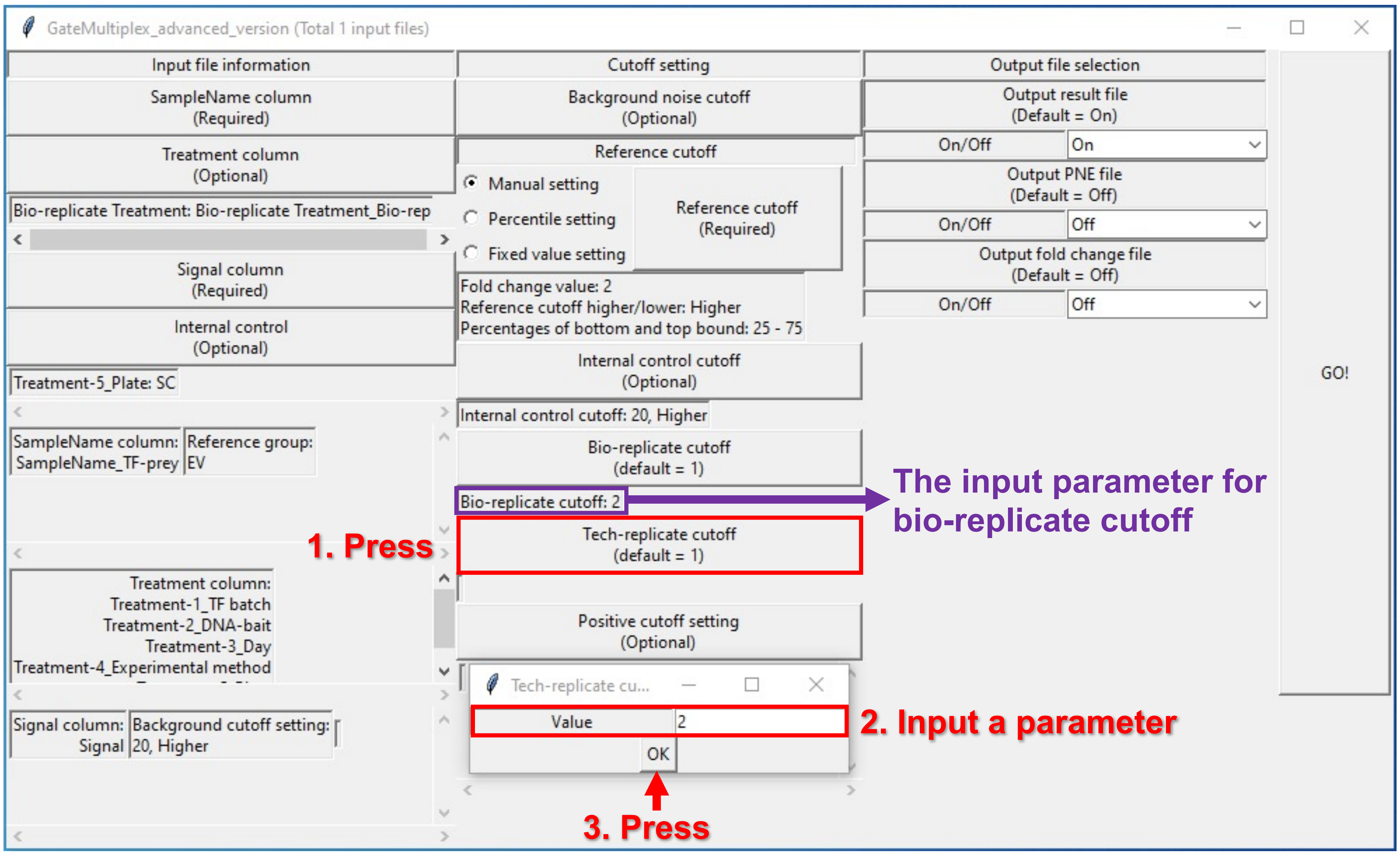

✱ Positive cutoff setting

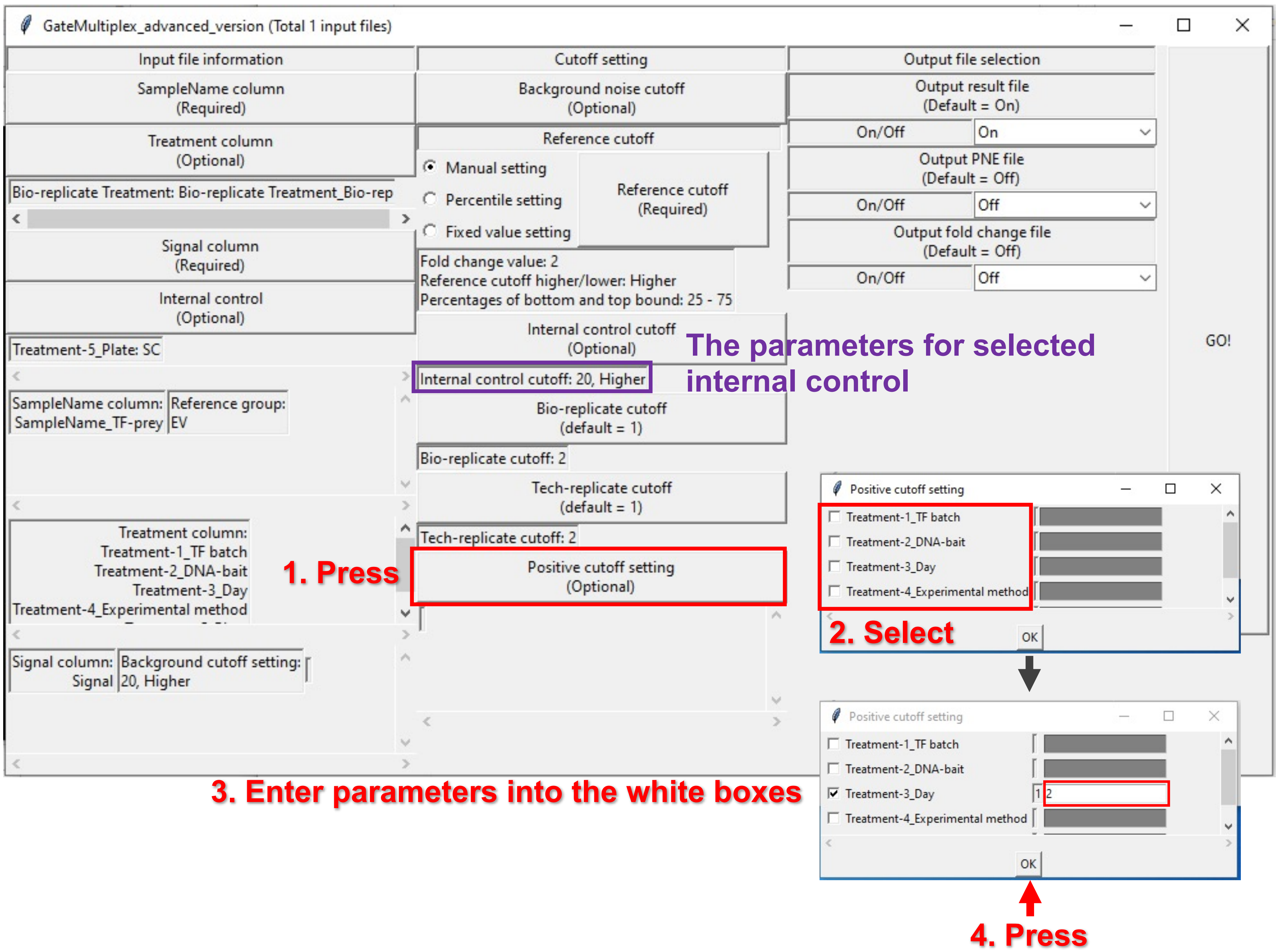

✱Positive cutoff setting (Advanced function)

✱ The Positive cutoff selection order is used as the operation order of positive cutoff. Please see Figure K1-K3 in Additional file 2 for detailed explanation.

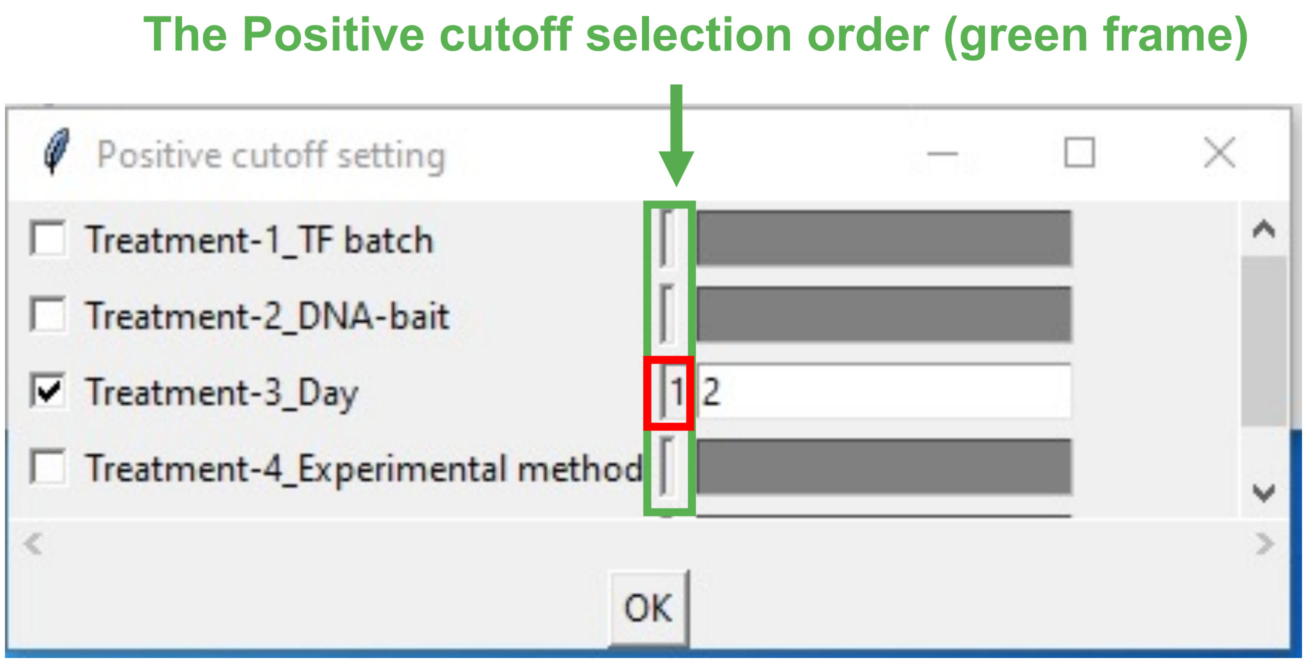

✱In our demonstrated example, "Treatment-3_Day" is the only Positive cutoff setting. Therefore, the Positive cutoff selection order of "Treatment-3_Day" is "1" (red frame)

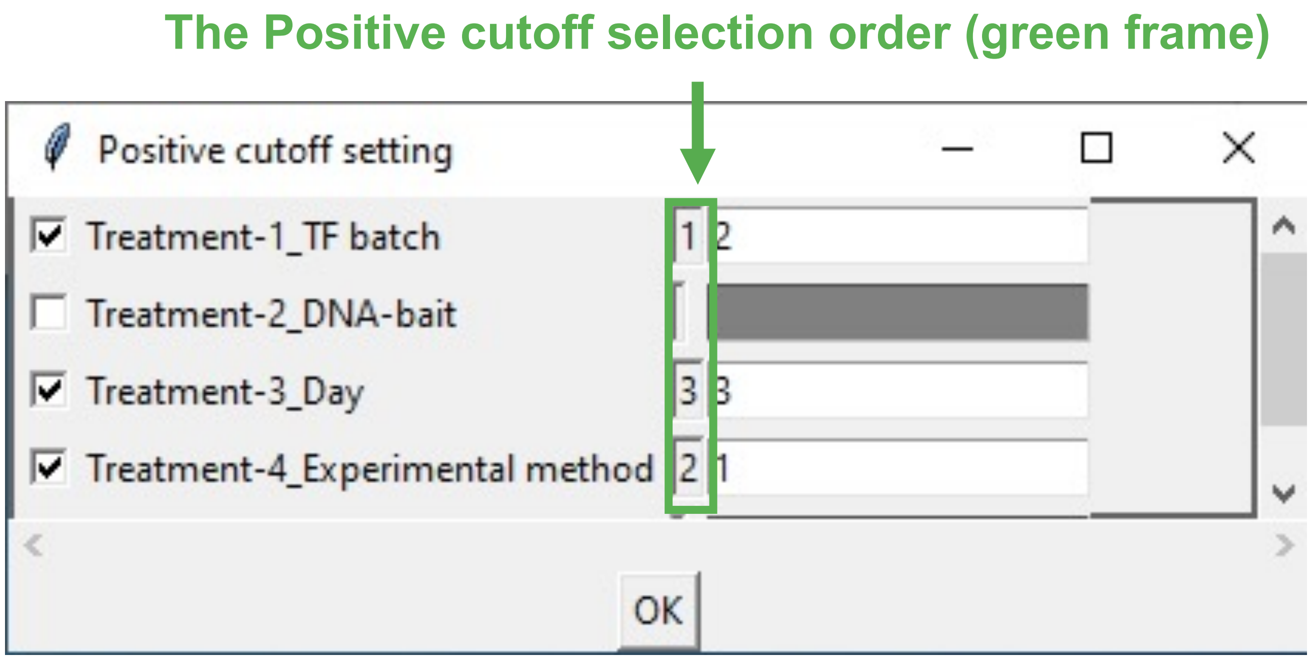

✱If the number of the Positive cutoff settings is increased, the Positive cutoff operation order will be the selection order.

✱ Output file selection

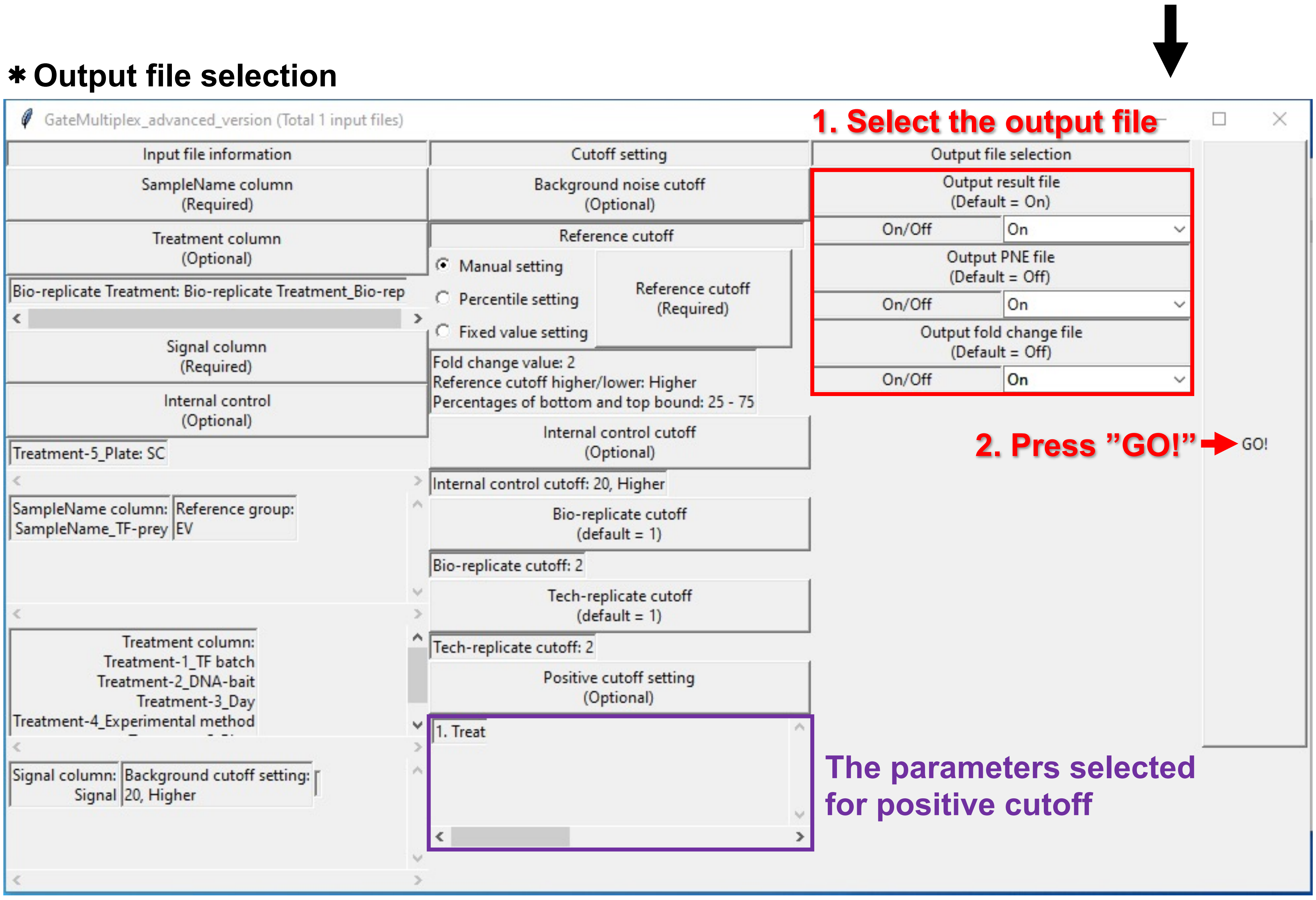

❖ The analysis will be completed after “Press any key to continue...” (red arrow) showing on the command line.

❖ Press any key to close the window.

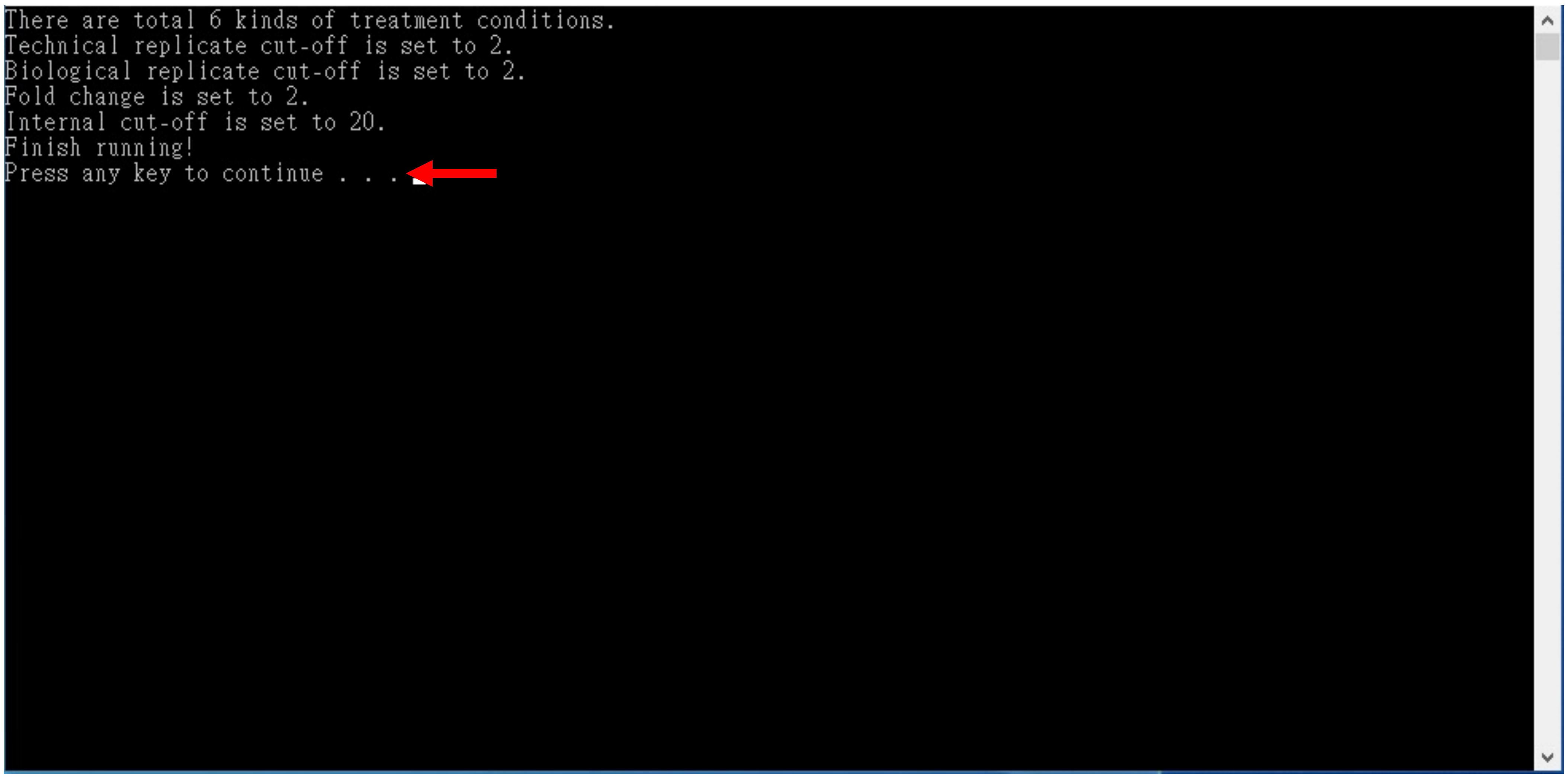

❖ The result file, named “Results” (red frame), is stored in the same folder with executive files of GM_Advanced.

❖ The fold change files (in purple frame) and the PNE files (in blue frame) will all be stored in the folder named “GM_outputfiles” (in green frame).

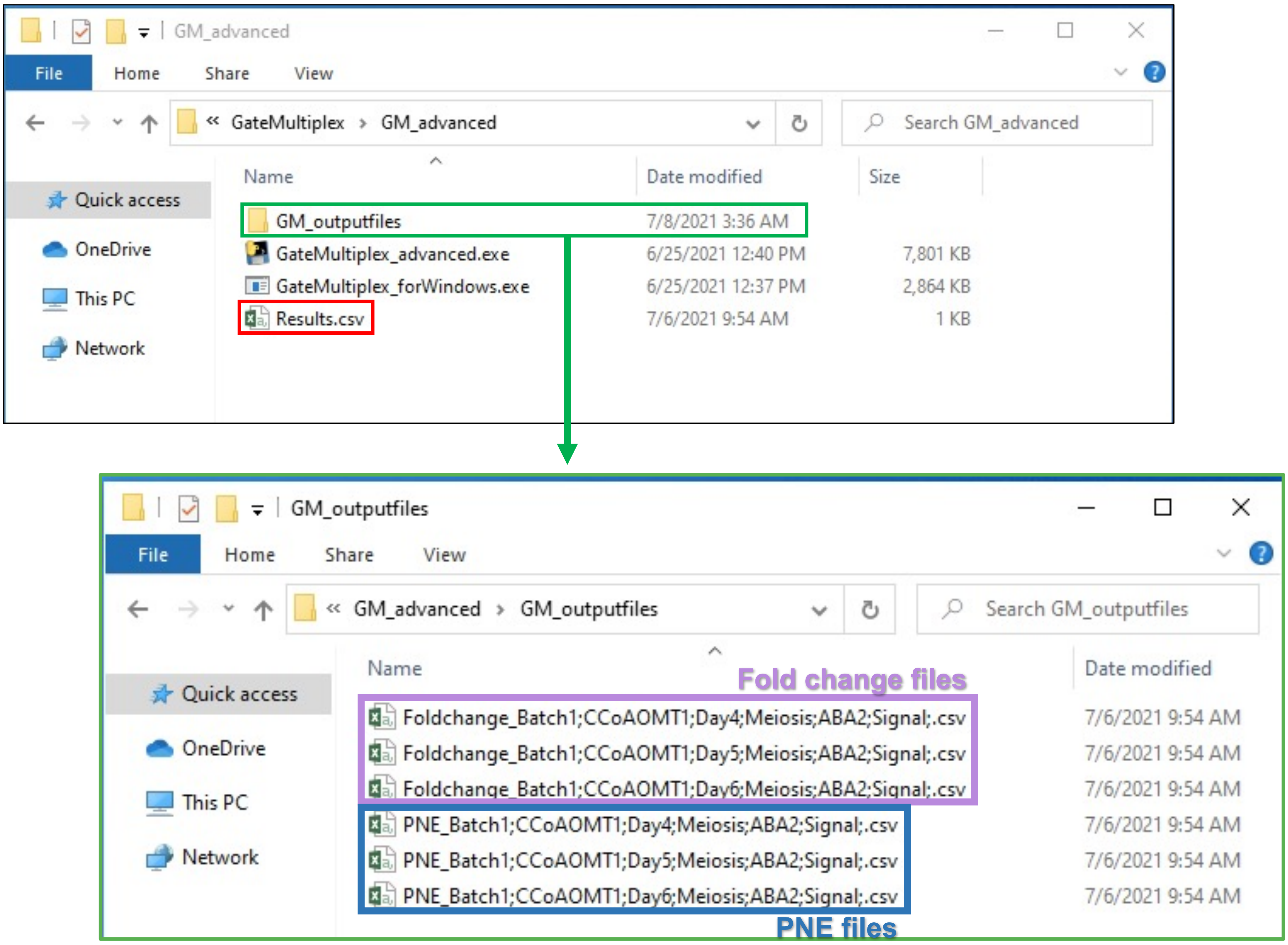

❖ The content of “Result.csv” file

- The symbol ”N” means negative, the symbol “P” means positive, and the symbol “E” means excluded. Please see the “Symbols in output files” section in Additional file 3.

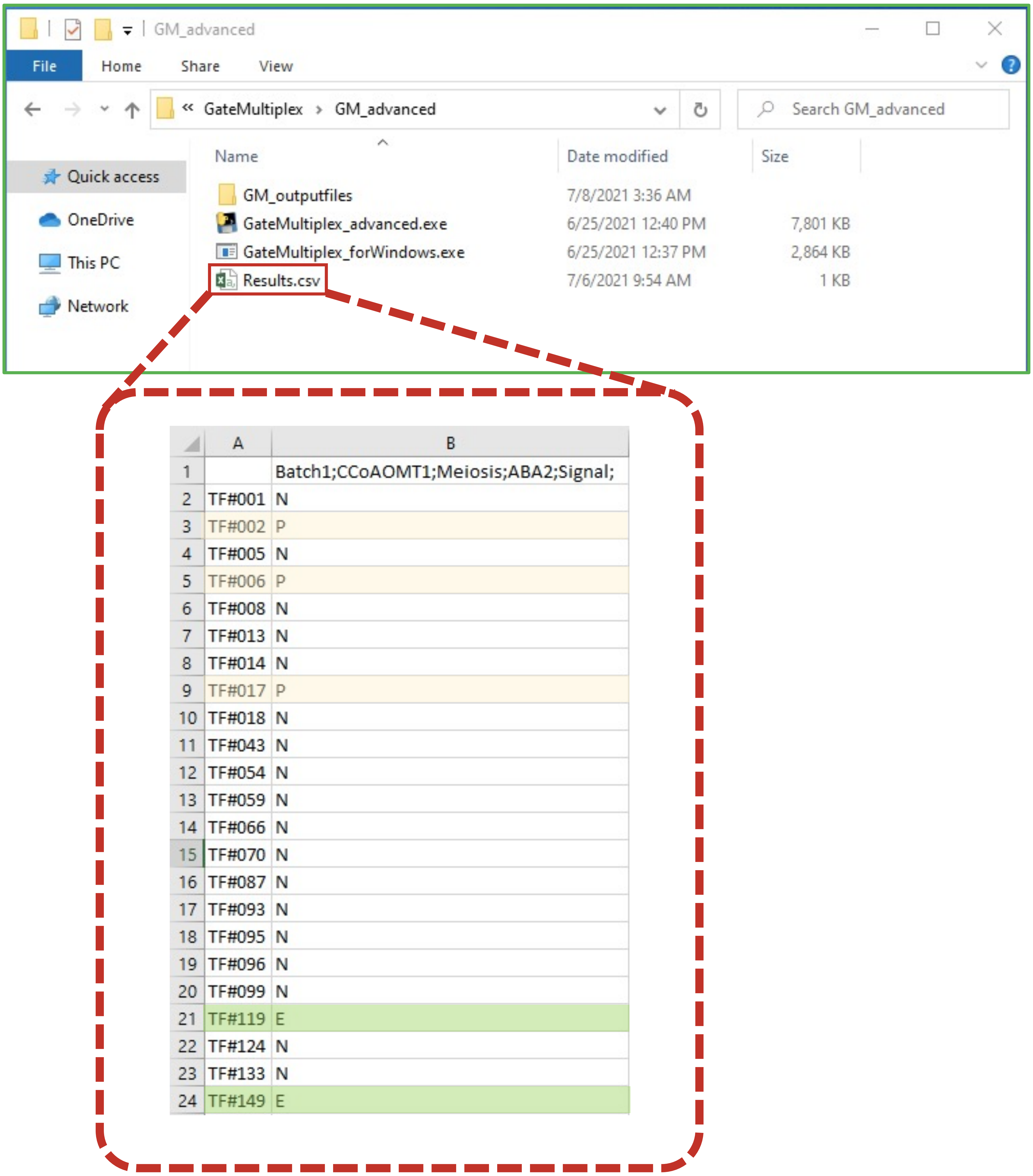

	A	B
1		Batch1;CCoAOMT1;Meiosis;ABA2;Signal;
2	TF#001	N
3	TF#002	P
4	TF#005	N
5	TF#006	P
6	TF#008	N
7	TF#013	N
8	TF#014	N
9	TF#017	P
10	TF#018	N
11	TF#043	N
12	TF#054	N
13	TF#059	N
14	TF#066	N
15	TF#070	N
16	TF#087	N
17	TF#093	N
18	TF#095	N
19	TF#096	N
20	TF#099	N
21	TF#119	E
22	TF#124	N
23	TF#133	N
24	TF#149	E

❖ The content of Fold change files

- The content of fold change file. Please see Figure L1-L3 for the concept of fold change file.
- The symbol ”nan” means “zero divided by zero”, and the symbol “inf” means “non-zero number divided by zero”. Please the “Symbols in output files” section in in Additional file 3.

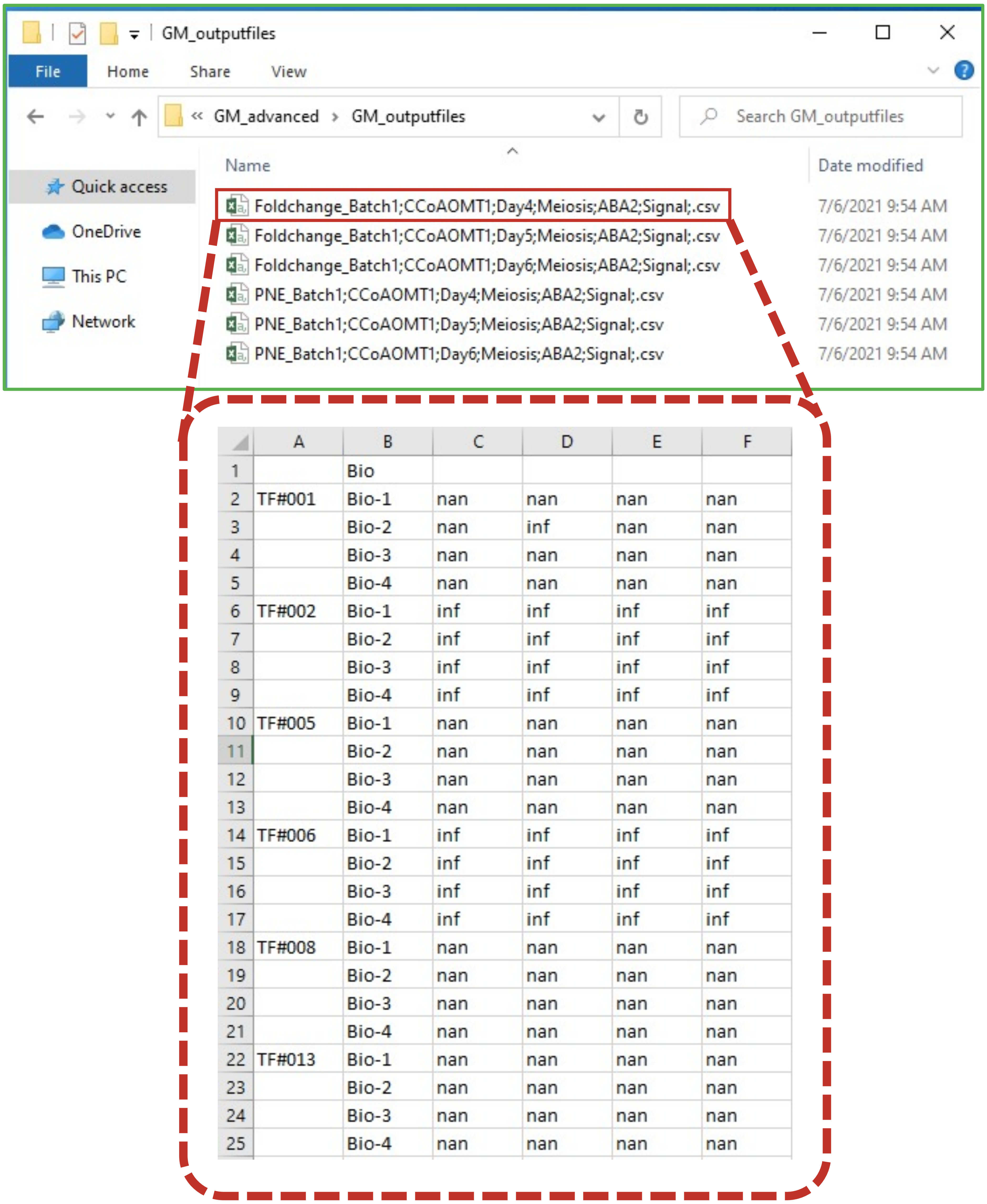

	A	B	C	D	E	F
1		Bio				
2	TF#001	Bio-1	nan	nan	nan	nan
3		Bio-2	nan	inf	nan	nan
4		Bio-3	nan	nan	nan	nan
5		Bio-4	nan	nan	nan	nan
6	TF#002	Bio-1	inf	inf	inf	inf
7		Bio-2	inf	inf	inf	inf
8		Bio-3	inf	inf	inf	inf
9		Bio-4	inf	inf	inf	inf
10	TF#005	Bio-1	nan	nan	nan	nan
11		Bio-2	nan	nan	nan	nan
12		Bio-3	nan	nan	nan	nan
13		Bio-4	nan	nan	nan	nan
14	TF#006	Bio-1	inf	inf	inf	inf
15		Bio-2	inf	inf	inf	inf
16		Bio-3	inf	inf	inf	inf
17		Bio-4	inf	inf	inf	inf
18	TF#008	Bio-1	nan	nan	nan	nan
19		Bio-2	nan	nan	nan	nan
20		Bio-3	nan	nan	nan	nan
21		Bio-4	nan	nan	nan	nan
22	TF#013	Bio-1	nan	nan	nan	nan
23		Bio-2	nan	nan	nan	nan
24		Bio-3	nan	nan	nan	nan
25		Bio-4	nan	nan	nan	nan

❖ The contents of PNE file

* The results in “PNE file” are the same as in “Result file” without being analyzed by positive cutoff. Please see Figure M in Additional file 2 for detailed explanation of “PNE file”.

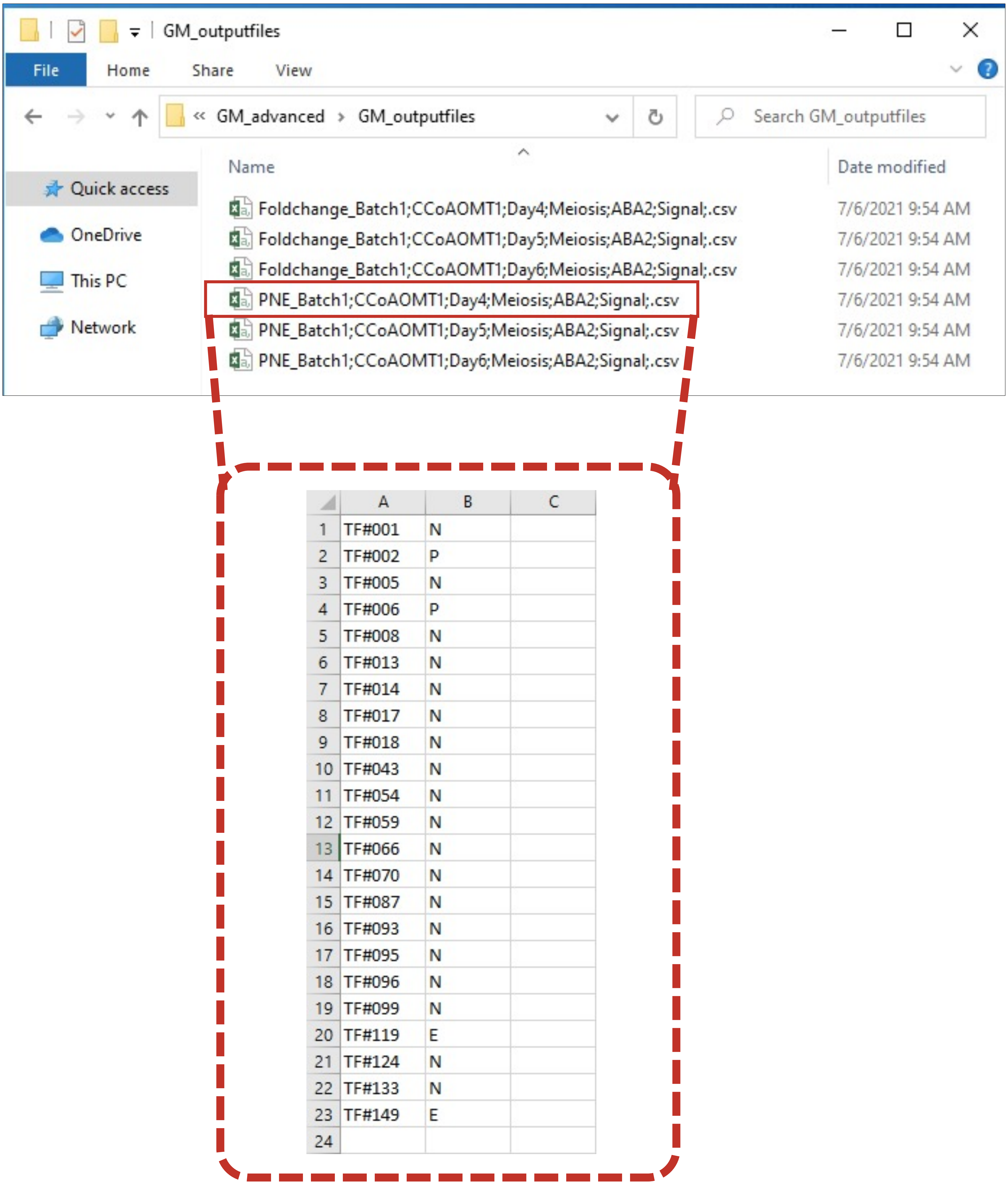

	A	B	C
1	TF#001	N	
2	TF#002	P	
3	TF#005	N	
4	TF#006	P	
5	TF#008	N	
6	TF#013	N	
7	TF#014	N	
8	TF#017	N	
9	TF#018	N	
10	TF#043	N	
11	TF#054	N	
12	TF#059	N	
13	TF#066	N	
14	TF#070	N	
15	TF#087	N	
16	TF#093	N	
17	TF#095	N	
18	TF#096	N	
19	TF#099	N	
20	TF#119	E	
21	TF#124	N	
22	TF#133	N	
23	TF#149	E	
24			

❖ Y1H (Large-Scale data)

- Except for the demo data for GM_Basic and GM_Advanced, a large scale data set with 407 files (406 files in “Files” folder and one file for the list of converter (“List_for_Converter.csv”) was also provided for the users to have more practice and to have a comprehensive understanding of the real data scale.

- *The provided large scale data set is from our previous study (PMID: 31186303, 2019 Genome Research). In this study, we used an updated version of PhenoBooth software to output the data.*

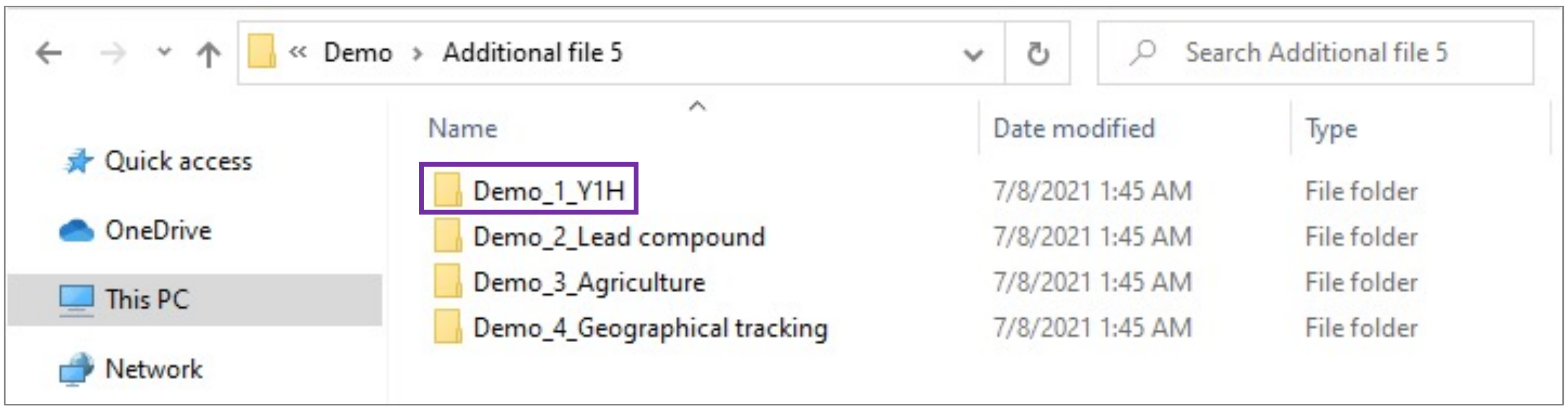

Enter the folder “Demo_1_Y1H” (purple frame)

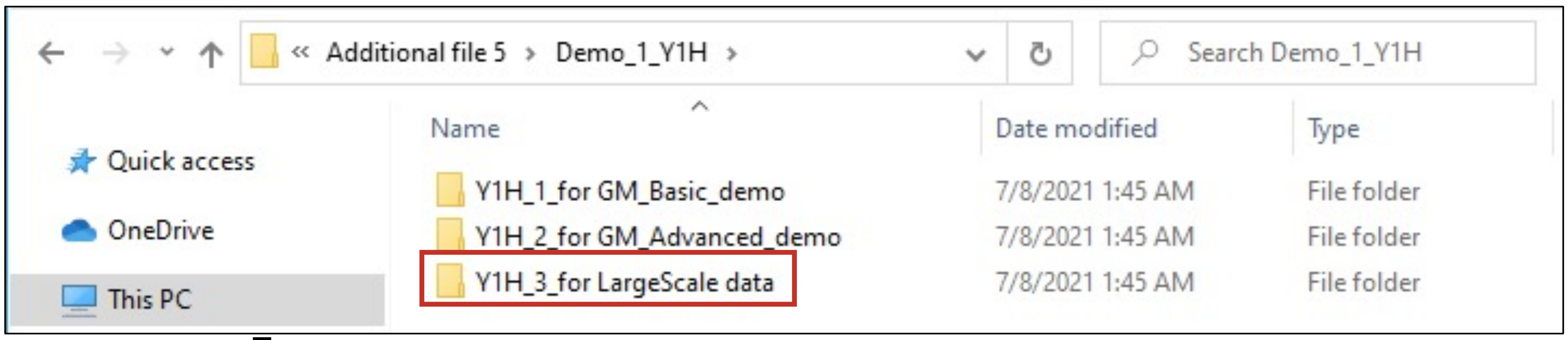

Enter the folder “Y1H_3_for LargeScale data” (red frame)

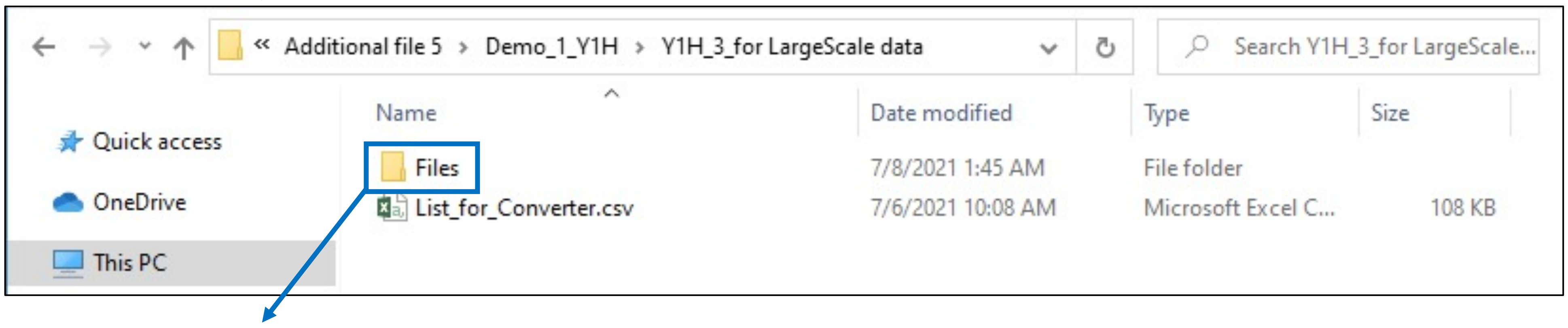

Partially shown (Total 406 files)

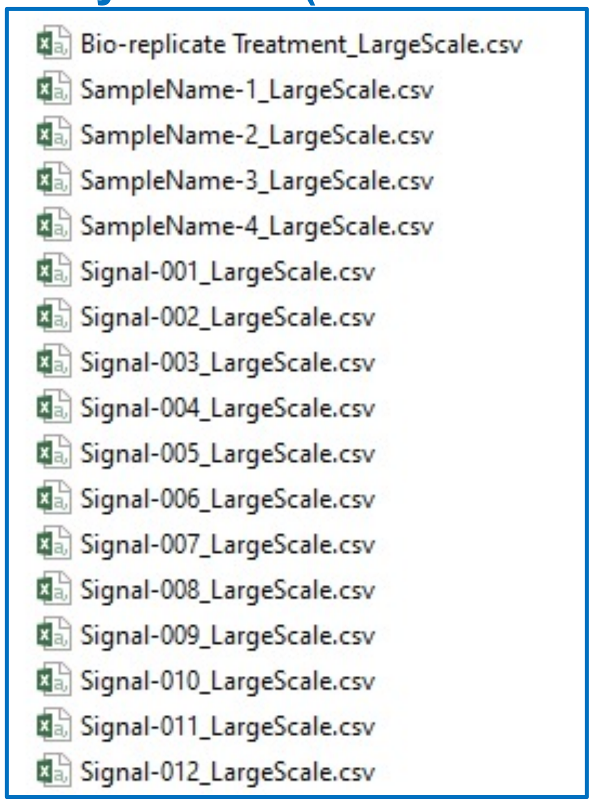

- 406 files:

Bio-replicate Treatment_LargeScale.csv

SampleName-1_LargeScale.csv to
SampleName-4_LargeScale.csv

Signal-001_LargeScale.csv to
Signal-384_LargeScale.csv

Treatment-1_TF batch#1_LargeScale.csv<br>Treatment-1_TF batch#2_LargeScale.csv<br>Treatment-1_TF batch#3_LargeScale.csv<br>Treatment-1_TF batch#4_LargeScale.csv	→	TF batch#1: Batch1<br>TF batch#2: Batch2<br>TF batch#3: Batch3<br>TF batch#4: Batch4
Treatment-2_DNA-bait#1_LargeScale.csv<br>Treatment-2_DNA-bait#2_LargeScale.csv<br>Treatment-2_DNA-bait#3_LargeScale.csv<br>Treatment-2_DNA-bait#4_LargeScale.csv	→	DNA-bait#1: CCoAOMT1<br>DNA-bait#2: CCoAOMT2<br>DNA-bait#3: CESA8<br>DNA-bait#4: HCT1
Treatment-3_Day#1_LargeScale.csv<br>Treatment-3_Day#2_LargeScale.csv<br>Treatment-3_Day#3_LargeScale.csv<br>Treatment-3_Day#4_LargeScale.csv	→	Day#1: Day4<br>Day#2: Day5<br>Day#3: Day6<br>Day#4: Day7
Treatment-4_Experimental method#1_LargeScale.csv<br>Treatment-4_Experimental method#2_LargeScale.csv<br>Treatment-4_Experimental method#3_LargeScale.csv	→	Experimental method#1: Meiosis<br>Experimental method#2: Diploid<br>Experimental method#3: Haploid
Treatment-5_Plate#1_LargeScale.csv<br>Treatment-5_Plate#2_LargeScale.csv	→	Plate#1: ABA2<br>Plate#2: SC

Warning of GateMultiplex

Warning of GM_Converter

❖ If the number in row or column (green frame) is not a positive integer, then the warning will pop-out after pressing “OK”.

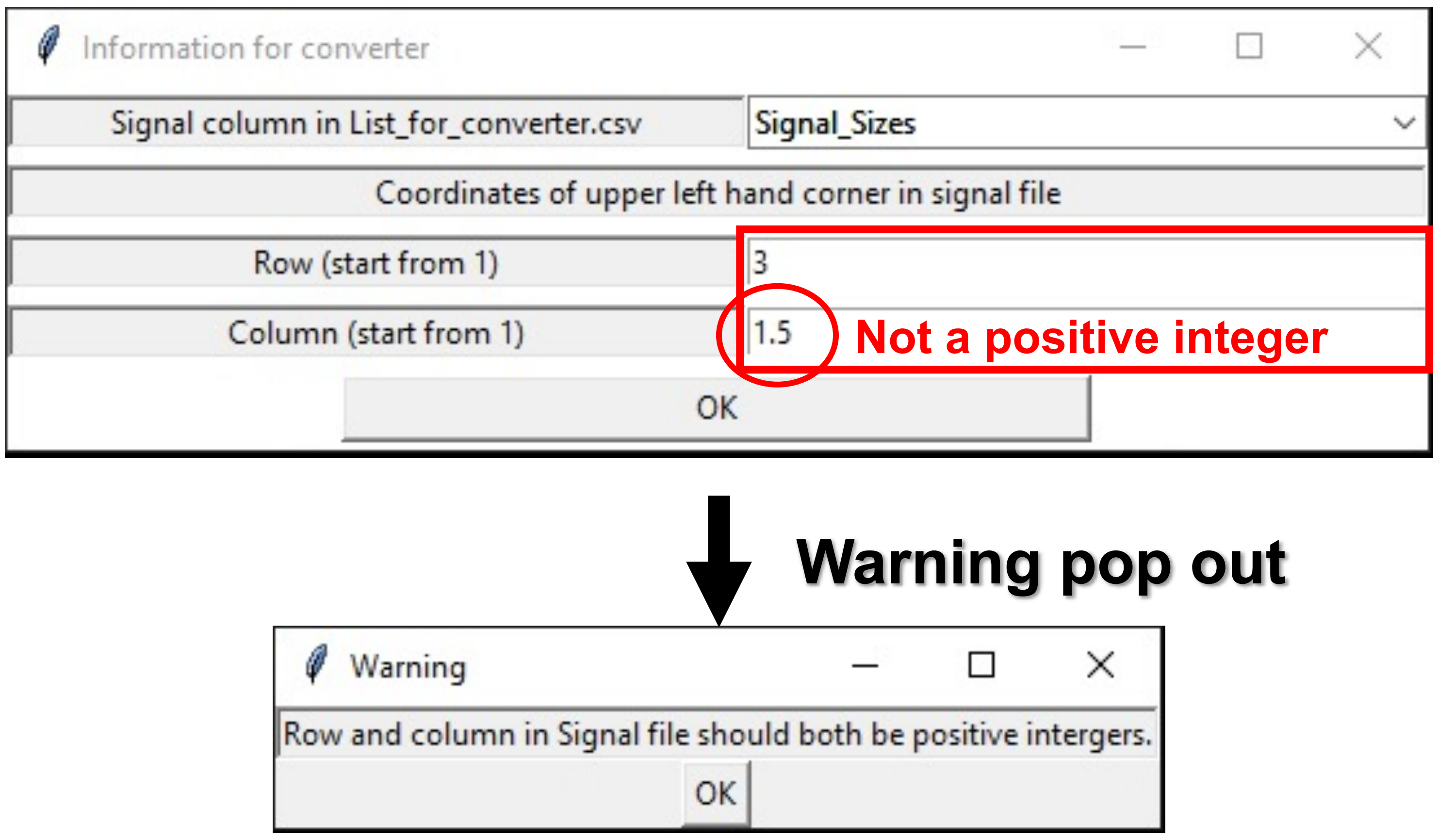

Warning of GM_Basic

❖ If titles of input files are inconsistent, then the warning will pop out after selecting the input folder.

- Take two title revised marine ecosystem data files for example (files are not provided).

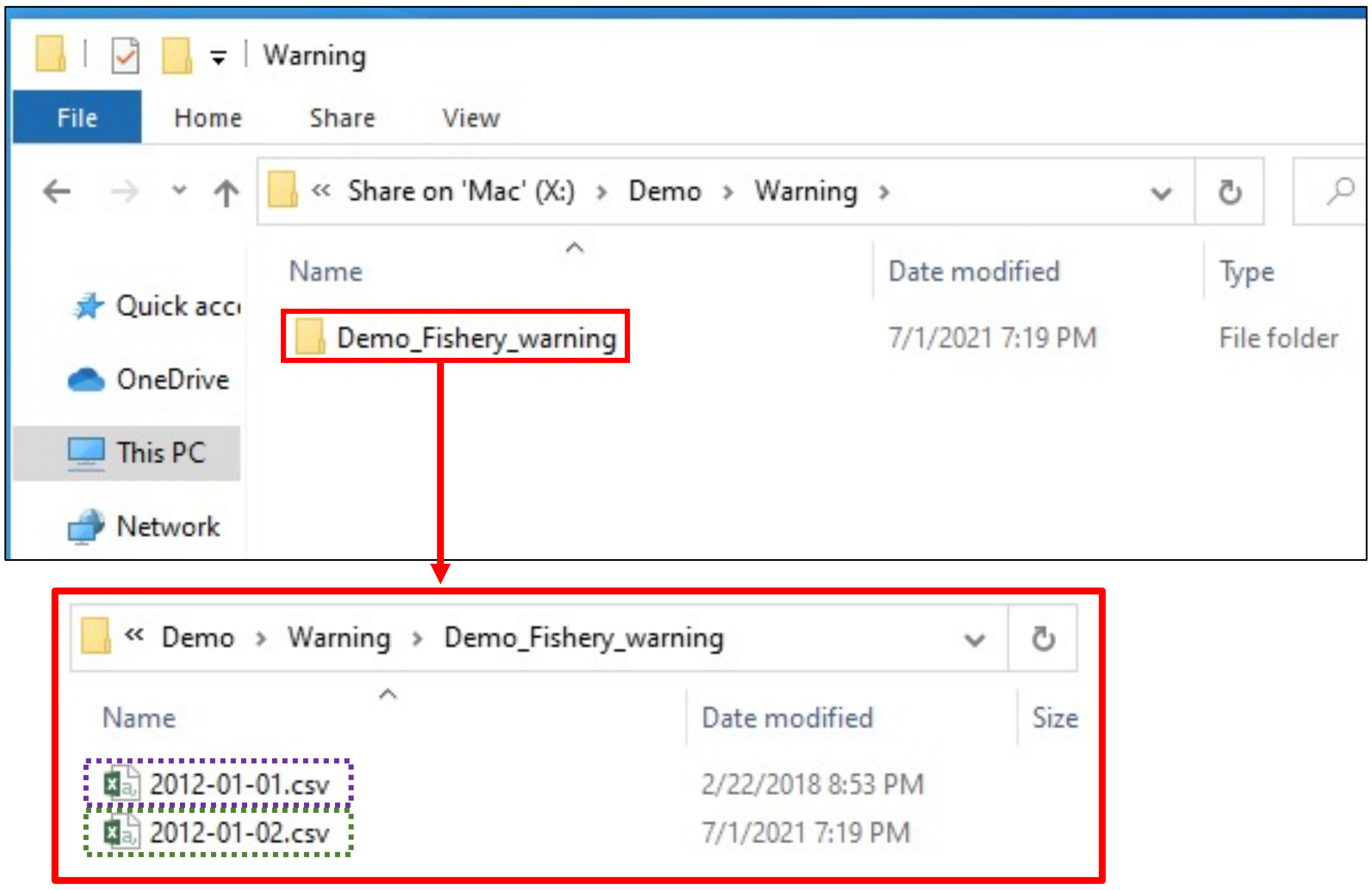

2012-01-01.csv

	A	B	C	D	E	F	G	H
1	Date	Latitude	Longitude	Country	Gear type	Vessel hour	Fishing hour	
2	1/1/2012	-4685	-6490	ARG	trawlers	1.59625	0	
3	1/1/2012	-4274	-6503	ARG	trawlers	28.69722222	0	
4	1/1/2012	-3463	-5836	ARG	trawlers	16.52763889	0	
5	1/1/2012	-4762	-6371	ARG	trawlers	0.805555556	0	

inconsistent

2012-01-02.csv

	A	B	C	D	E	F	G	H
1	Date	Latitude	Longitude	Country	Gear	Vessel hour	Fishing hour	
2	1/2/2012	-4598	-6615	ARG	fixed_gear	8.341666667	0	
3	1/2/2012	-4597	-6621	ARG	fixed_gear	0.770972222	0	
4	1/2/2012	-4597	-6622	ARG	fixed_gear	0.851666667	0	
5	1/2/2012	-4593	-6621	ARG	fixed_gear	1.606805556	0	

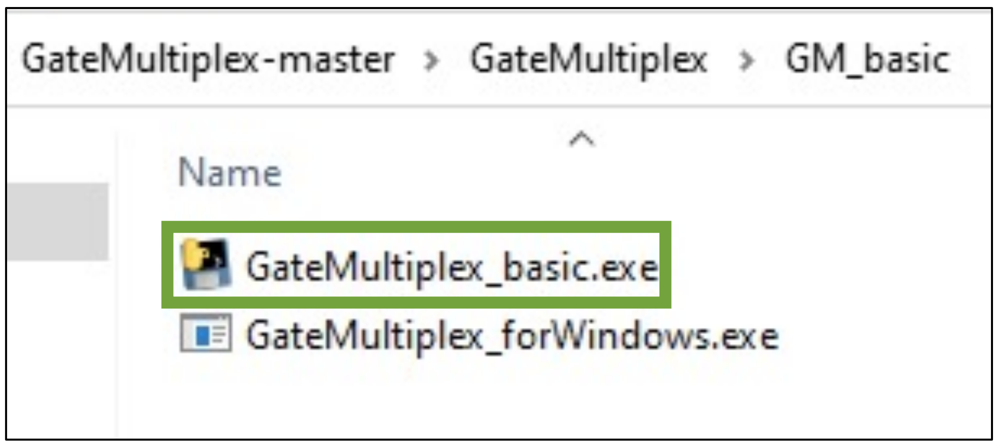

Activate the “GateMultiplex_basic.exe” (green frame).

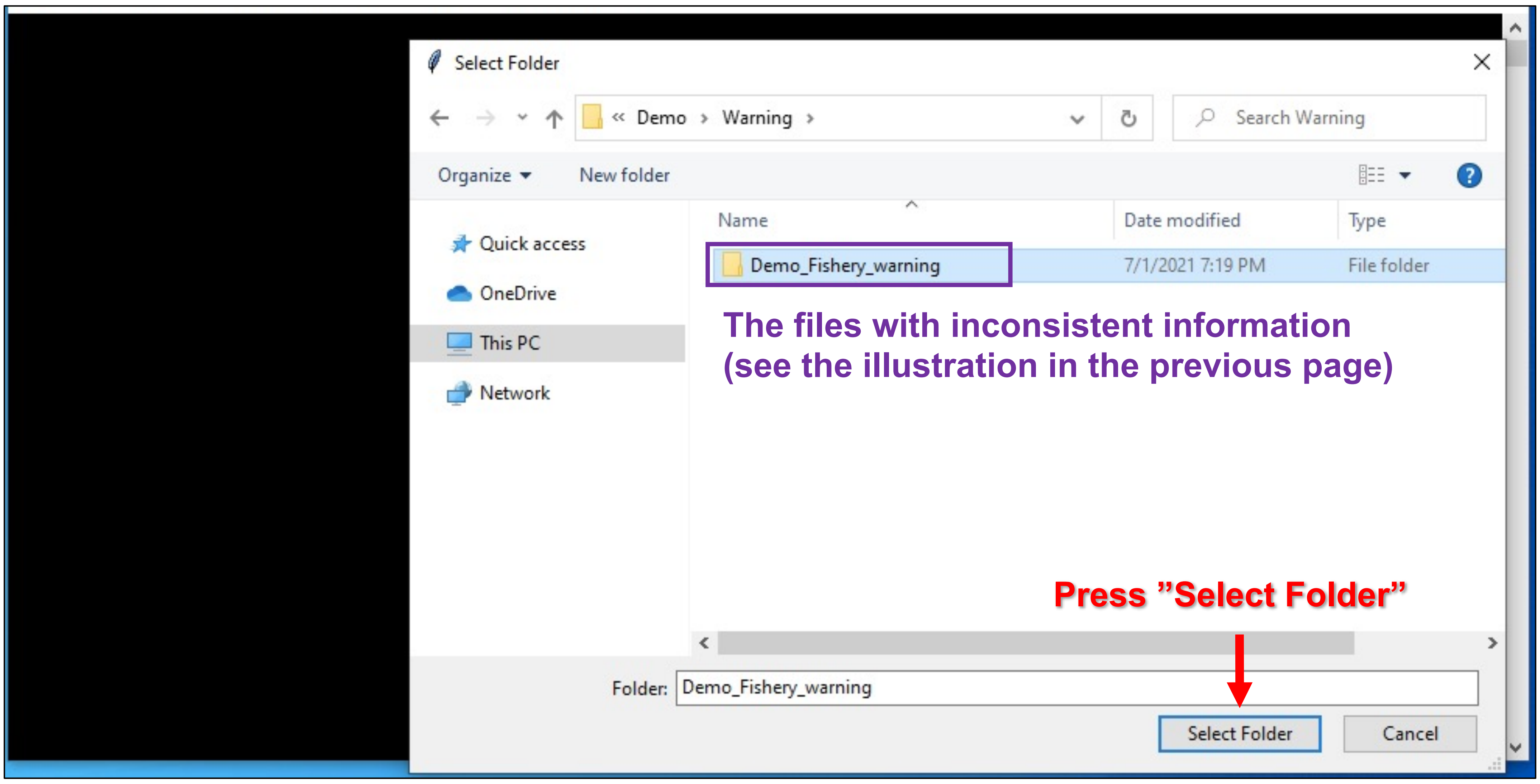

Warning pop out

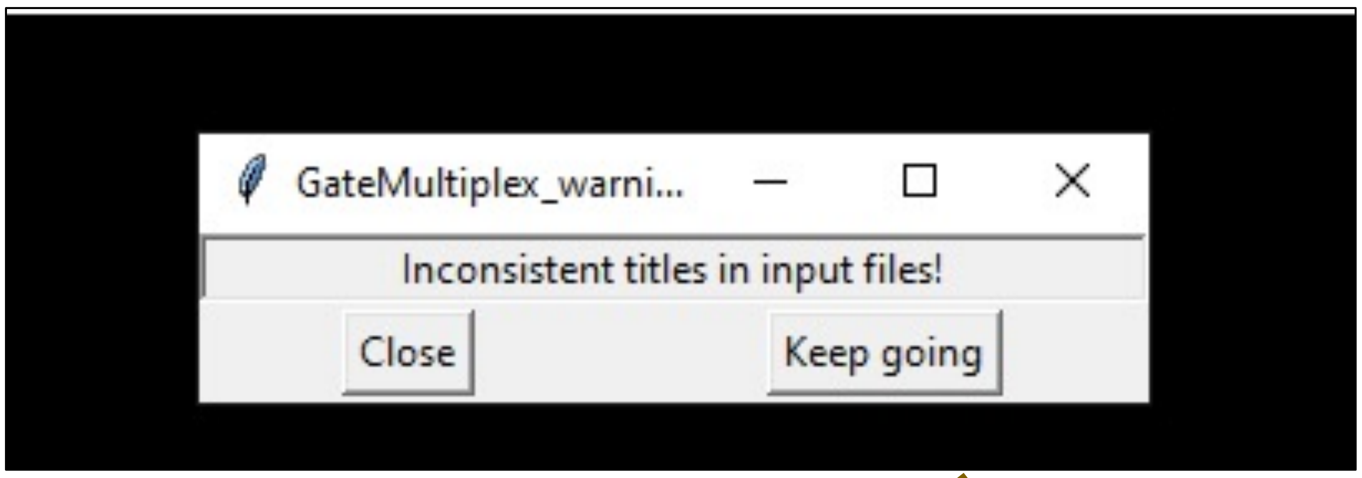

Press “Close”, then the GateMultiplex program will be closed

Press “Keep going”, then the warning will be ignored

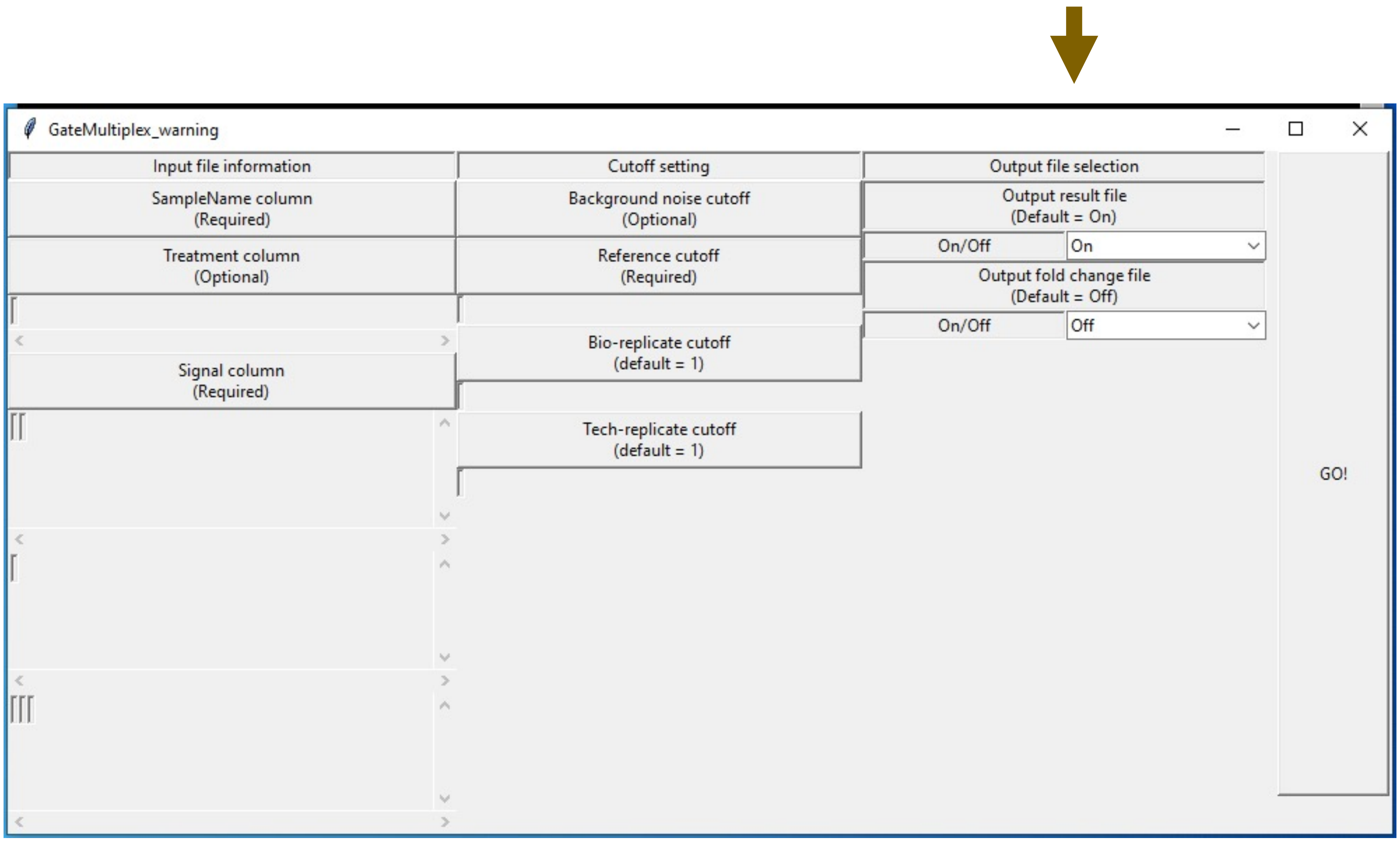

❖ If SampleName column, Signal column, or Reference cutoff (red frames) is not set, the warning will pop out after pressing "GO!".

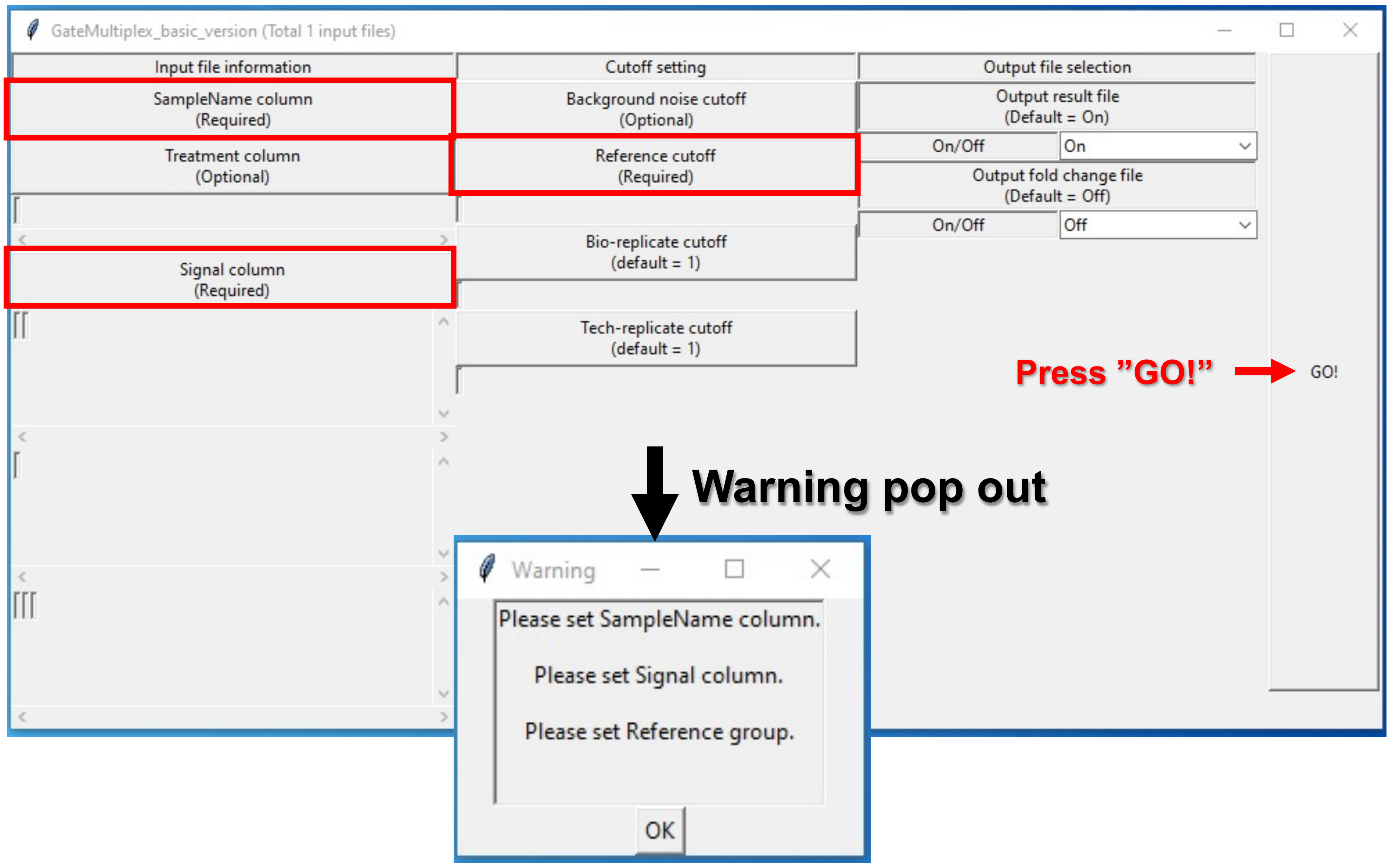

❖ If Signal column is not set, the warning will pop out after pressing the button of Background noise cutoff.

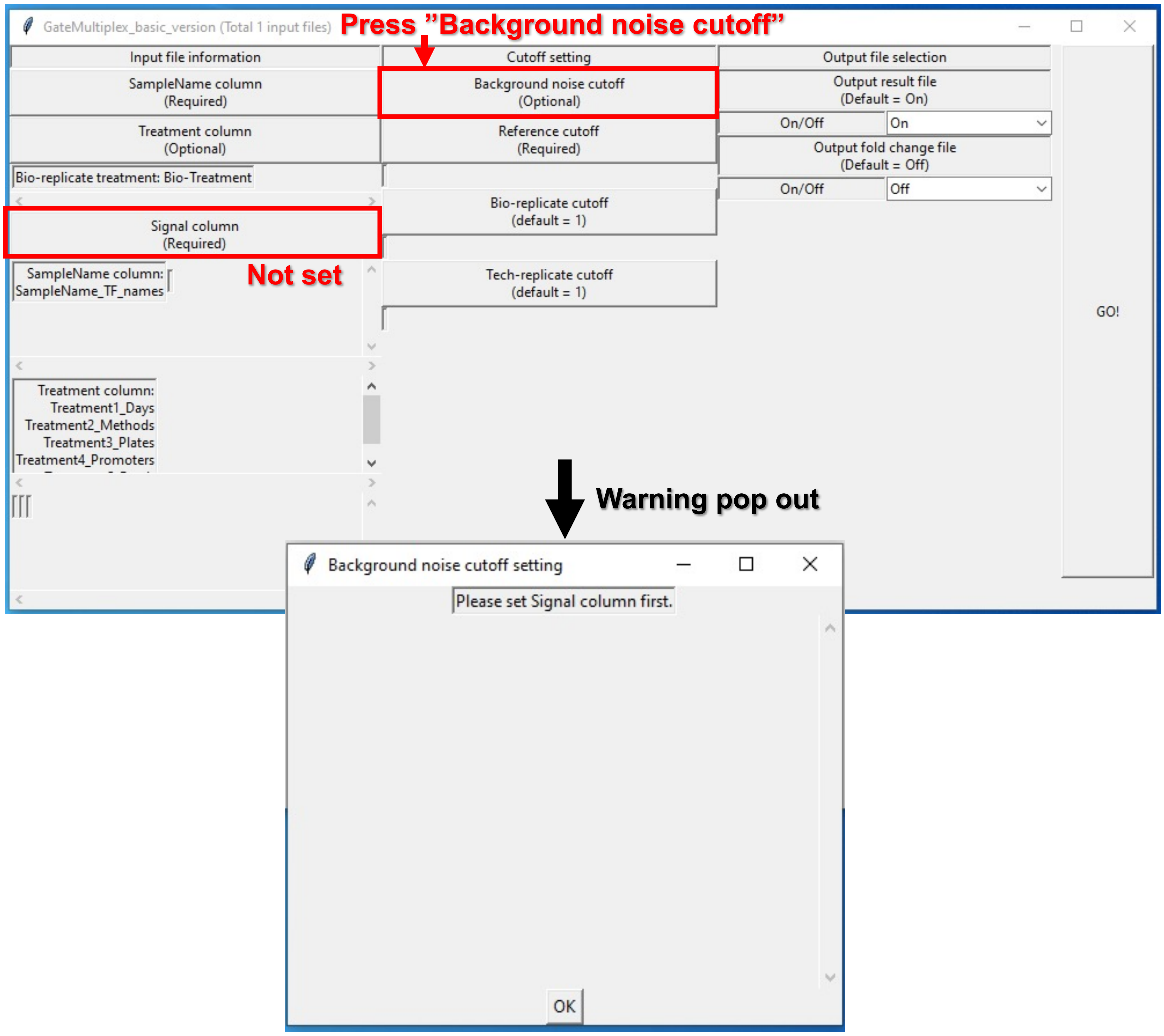

❖ If the value of Background noise cutoff is not a numeric value, the warning will pop out after pressing “OK”.

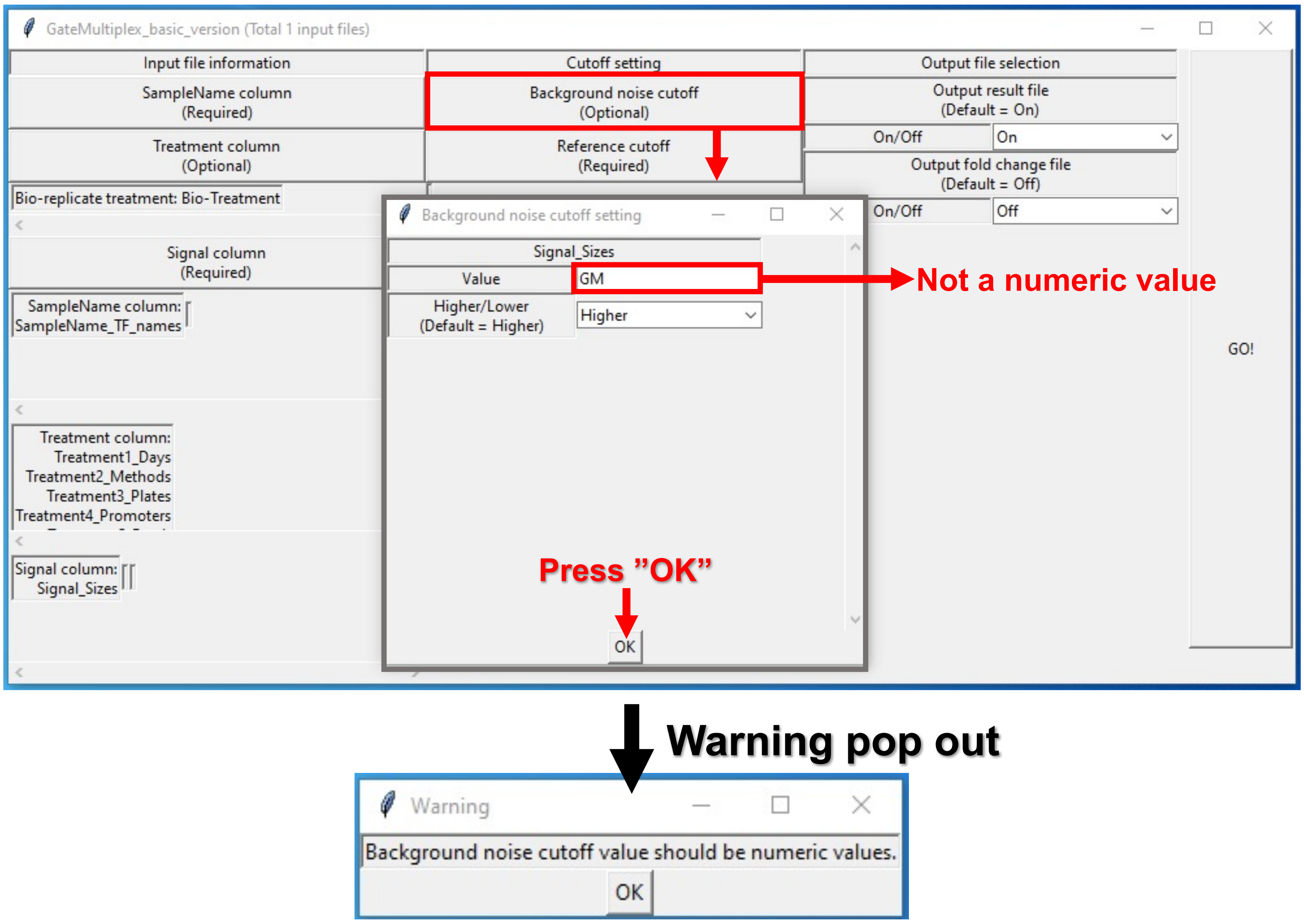

❖ If SampleName column is not set, the warning will pop out after pressing the button of Reference cutoff.

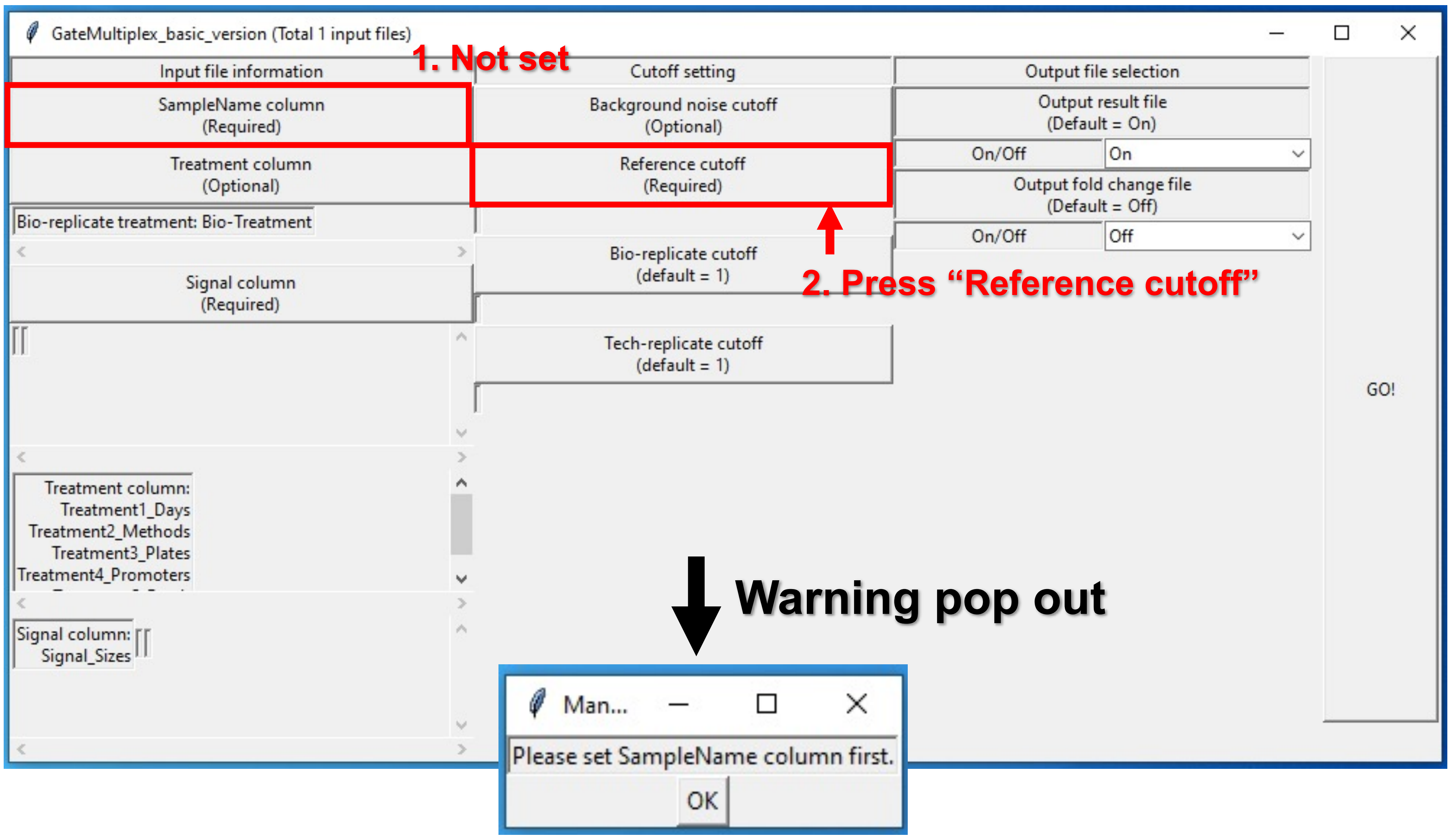

❖ If the sample name in Reference cutoff setting is not set, the warning will pop out after pressing “OK”.

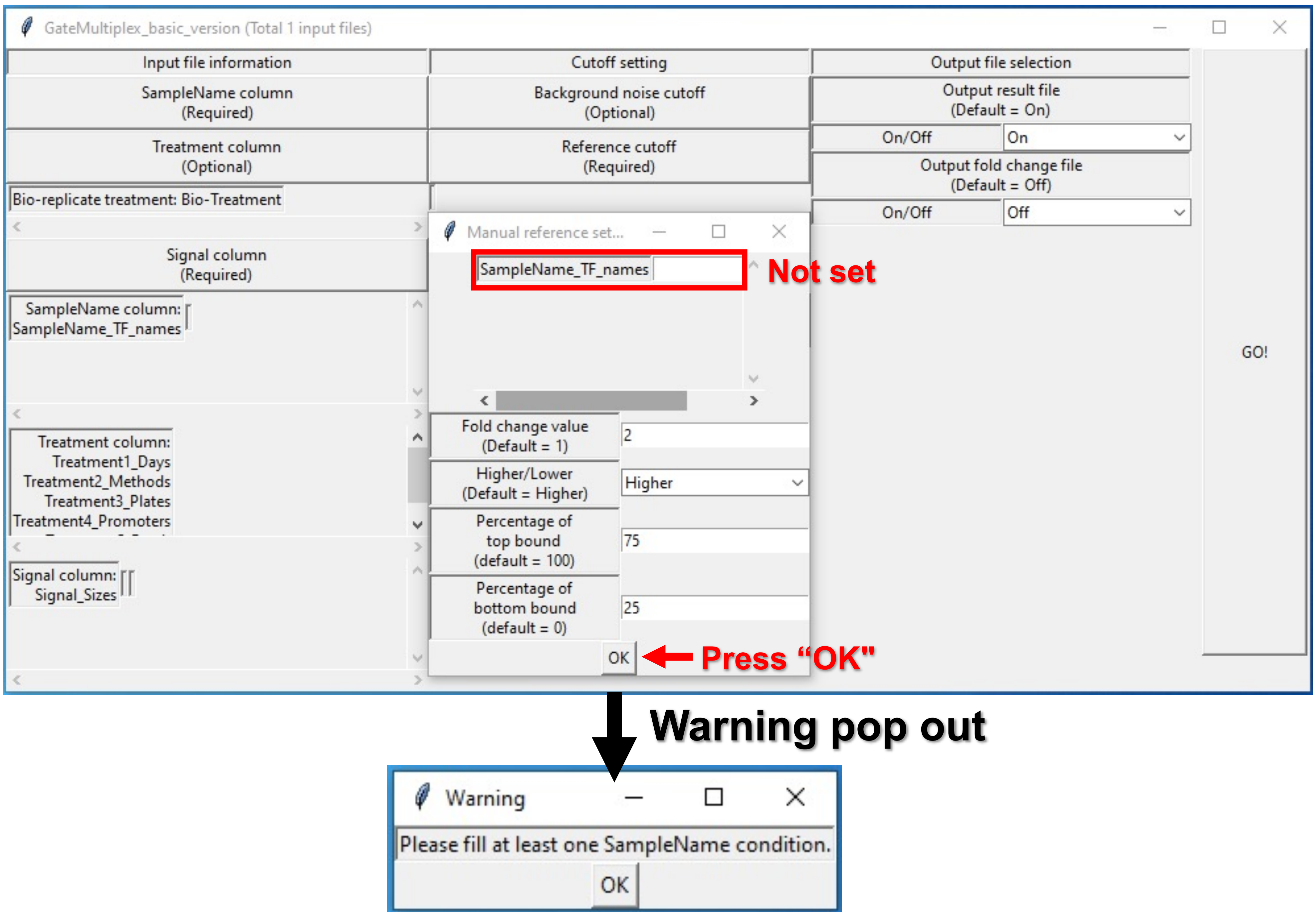

❖ If value of Bio-replicate cutoff is not a positive integer, the warning will pop out after pressing “OK”.

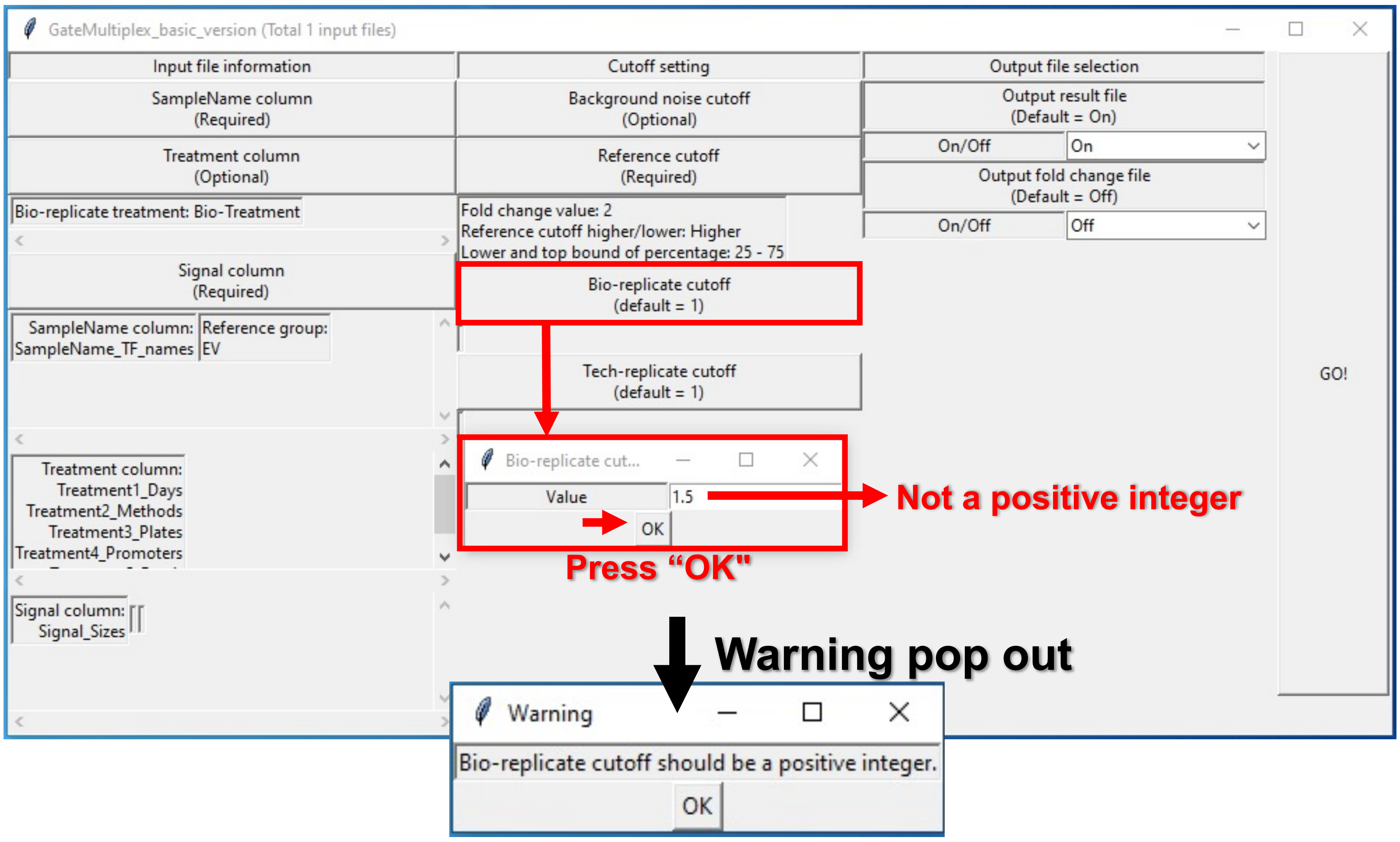

❖ If value of tech-replicate cutoff is not a positive integer, the warning will pop out after pressing “OK”.

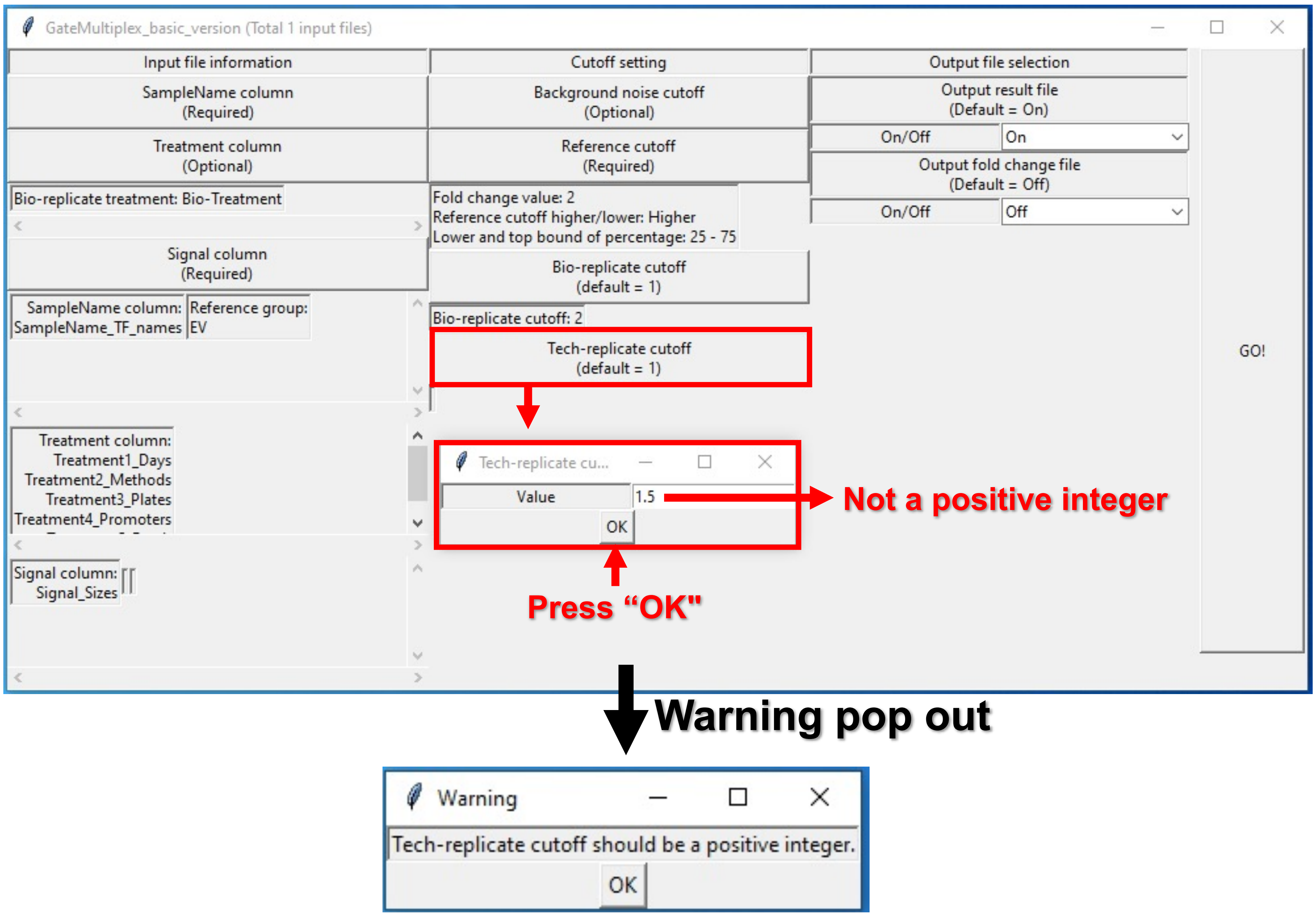

Warning of GM_Advanced

❖ The warning of GM_Advanced is similar to that of GM_Basic. Several overlapped warnings are skipped here. Please see the warning section of GM_Basic.

❖ In the manual setting of reference cutoff, if SampleName column is not set, the warning will pop out after selecting “manual setting” and pressing the button of Reference cutoff.

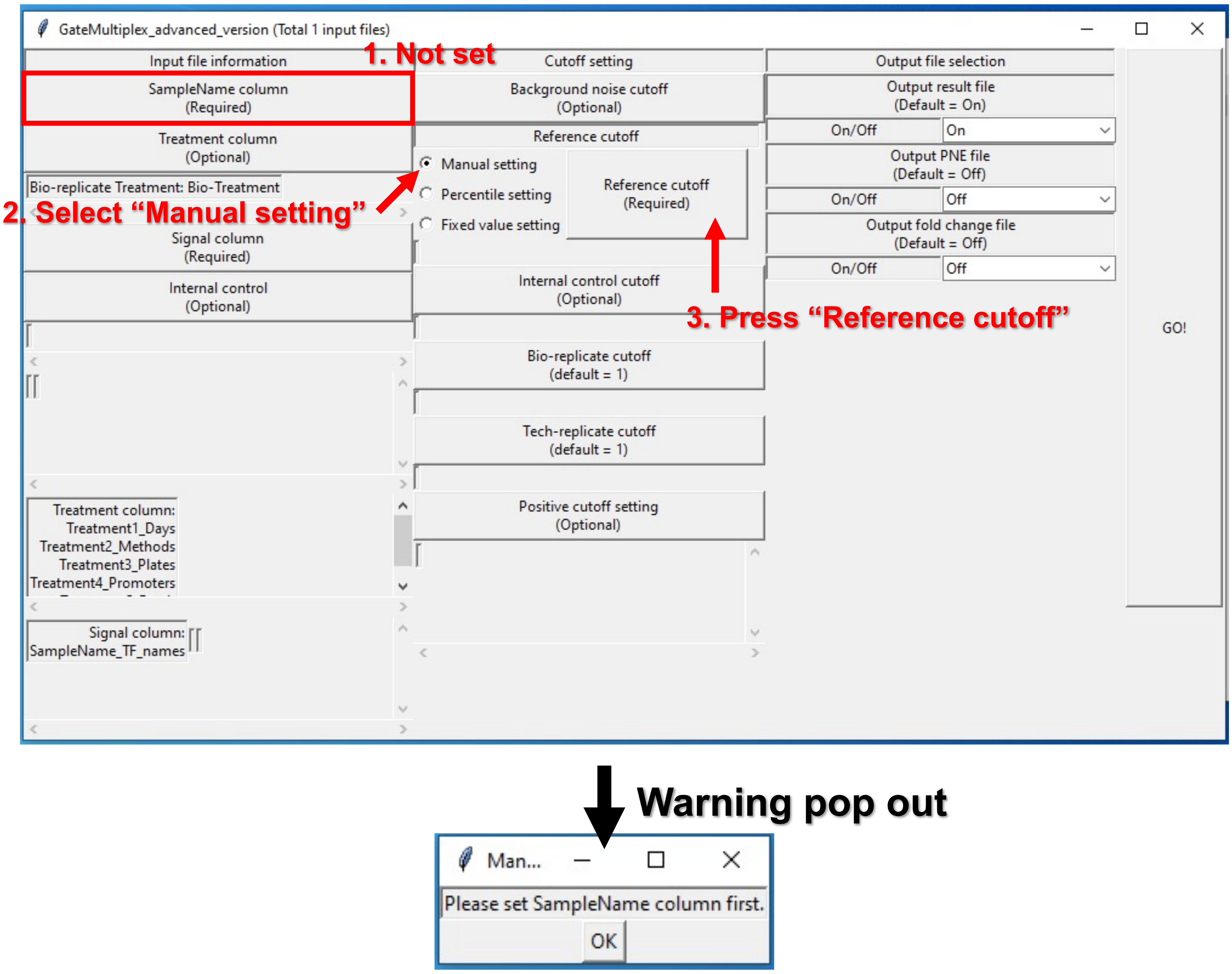

❖ In the manual setting of reference cutoff, if the sample name in reference cutoff is not set, the warning will pop out after pressing “OK”.

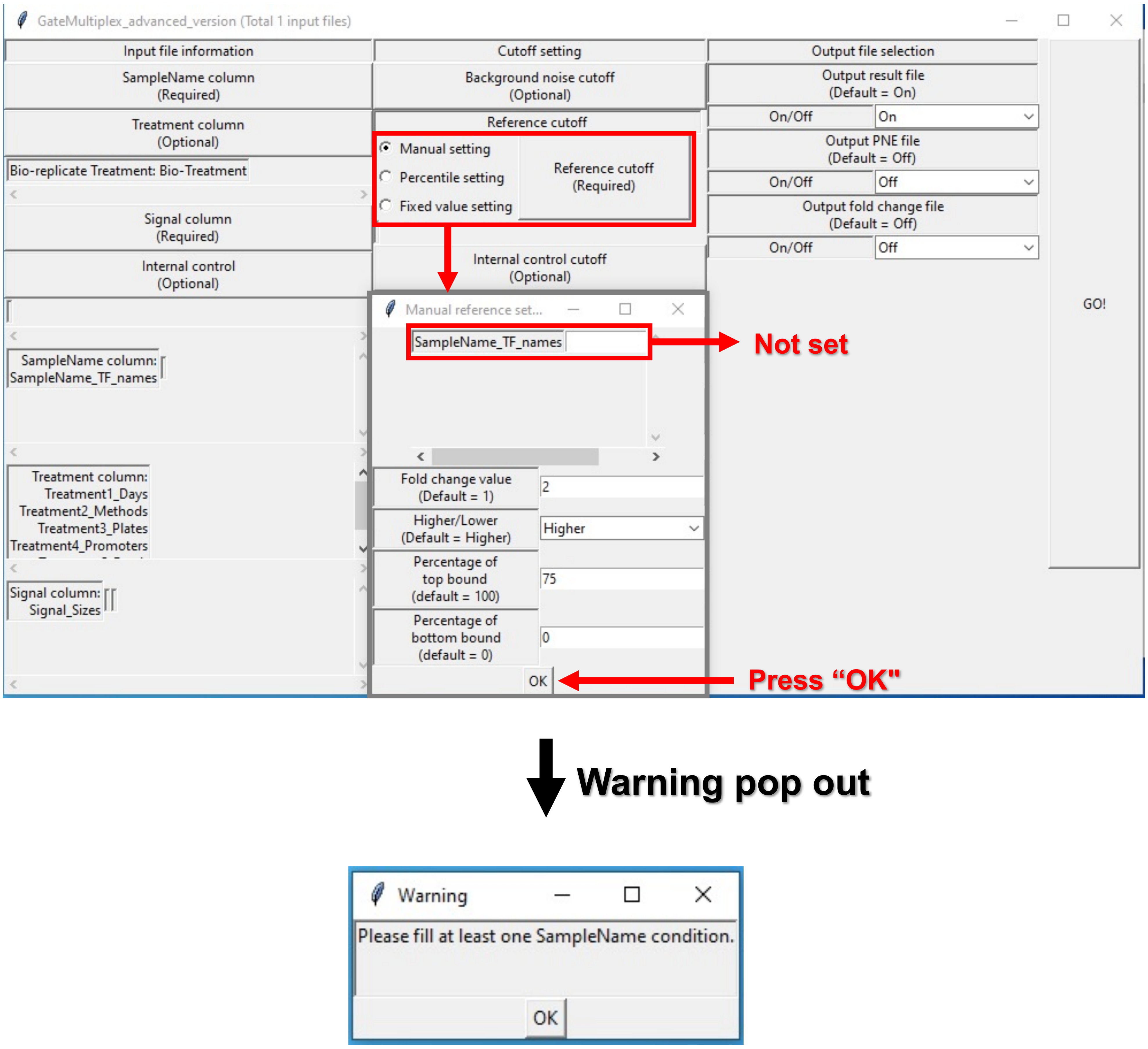

❖ In the percentile setting of reference cutoff, if the percentile value is not a numeric value between 0 and 100, the warning will pop out after pressing “OK”.

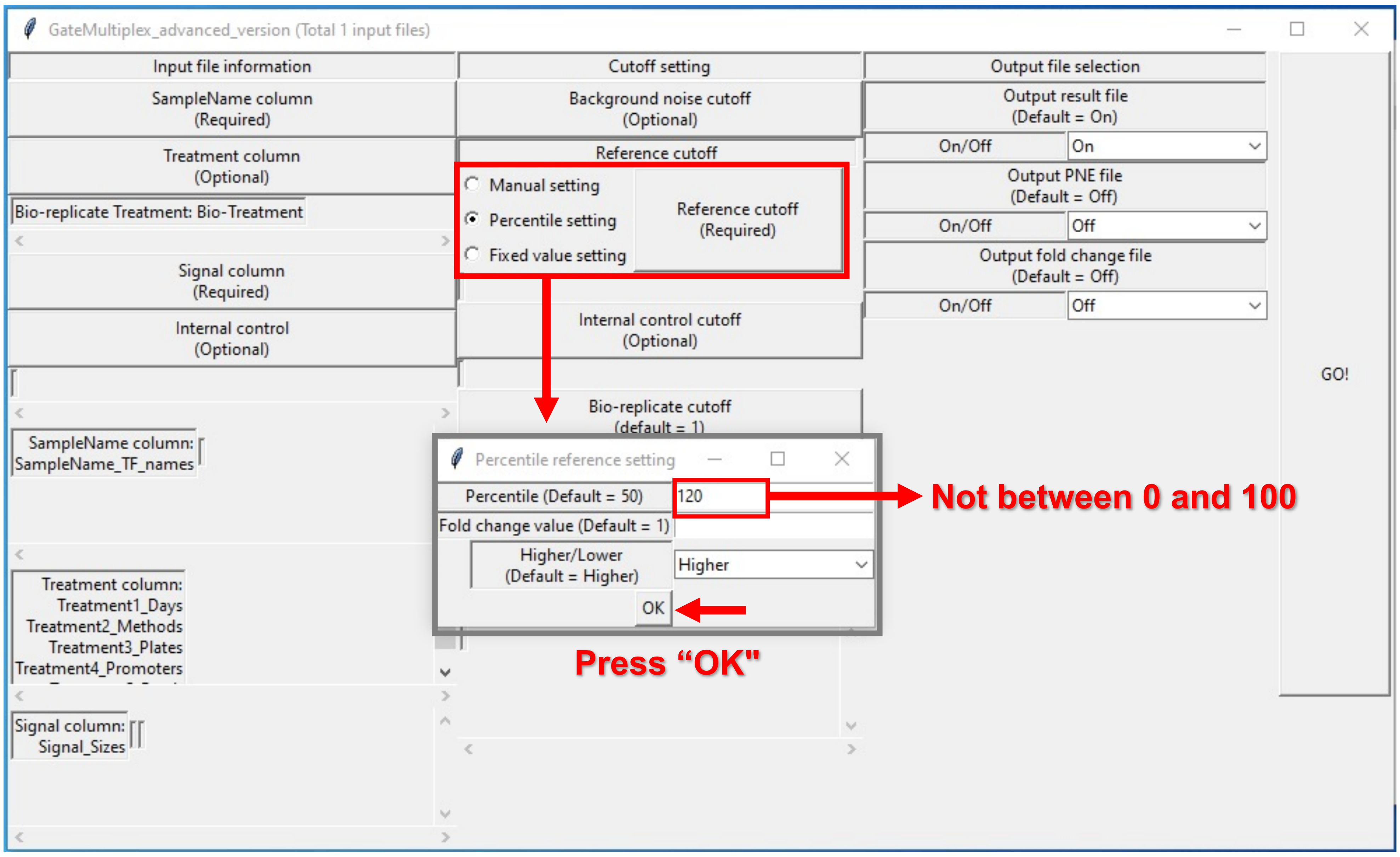

Warning pop out

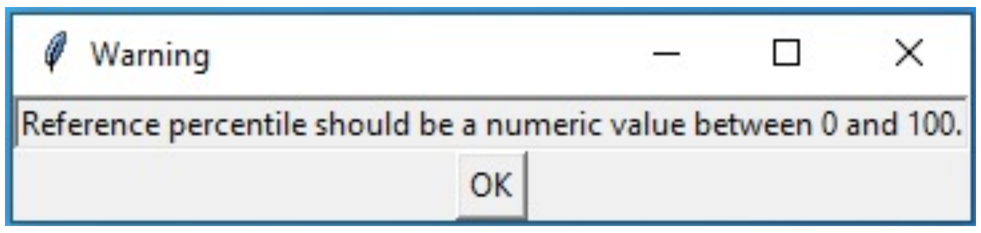

❖ In the fixed value setting of reference cutoff, if Signal column is not set, the warning will pop out after selecting ”fixed value setting” and pressing the button of Reference cutoff.

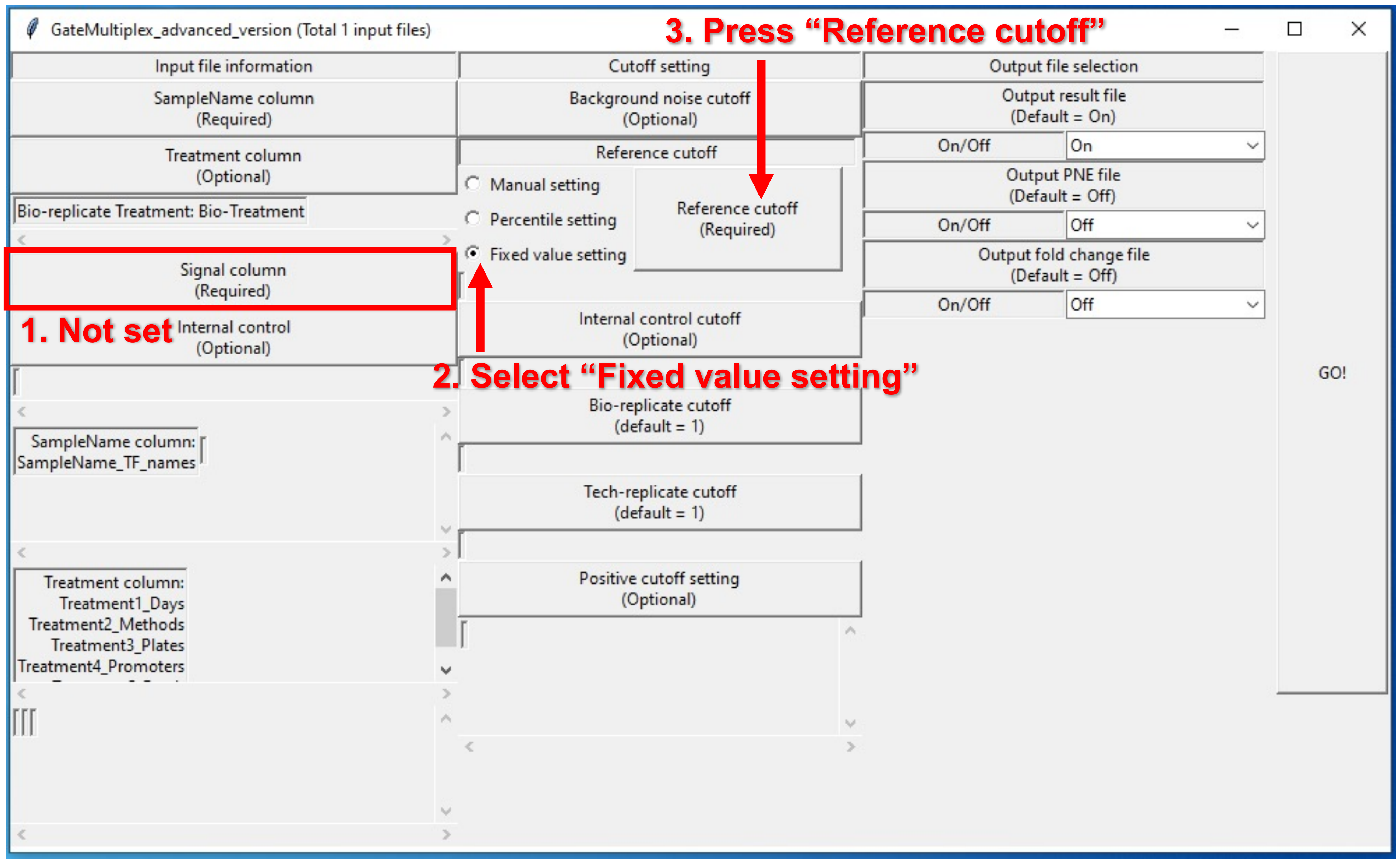

Warning pop out

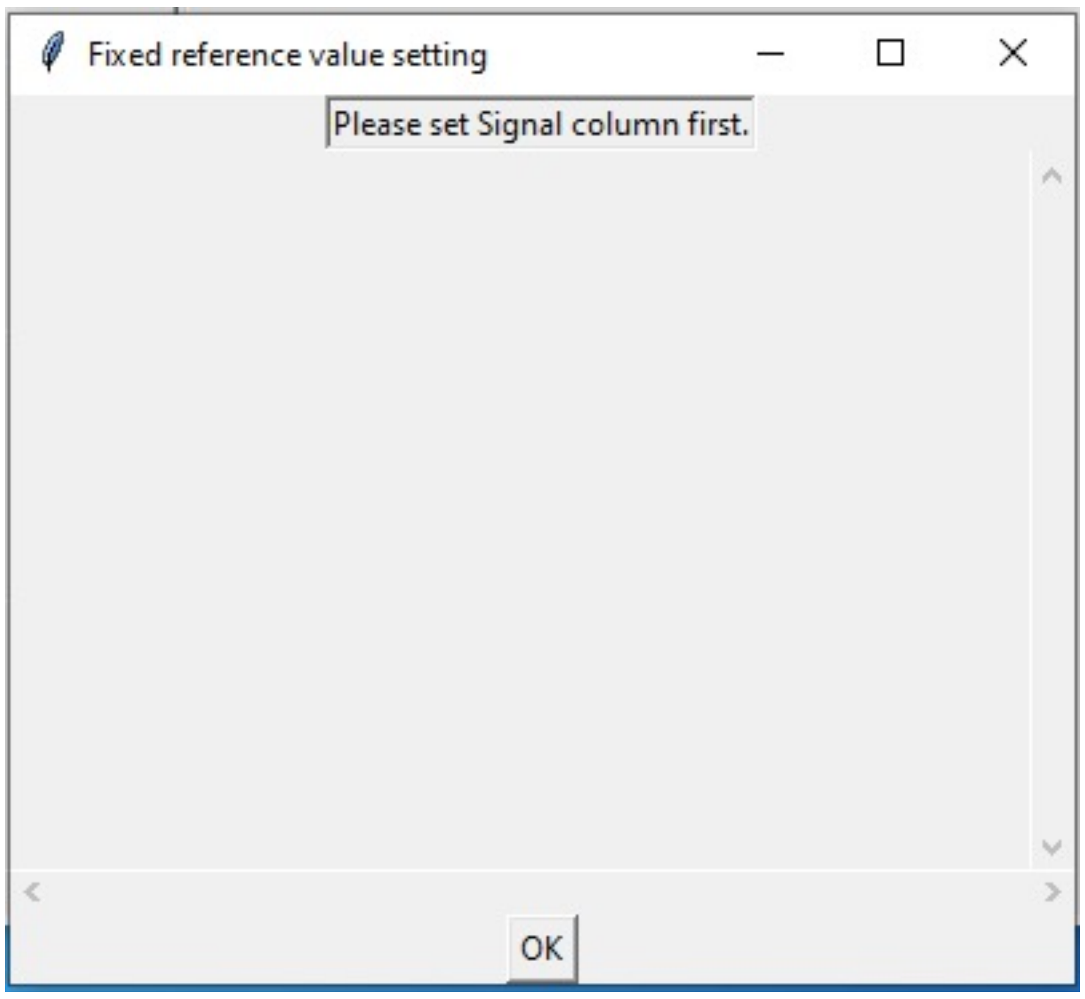

- In the fixed value setting of reference cutoff, if the fixed reference value is not a numeric value, the warning will pop out after pressing “OK”.

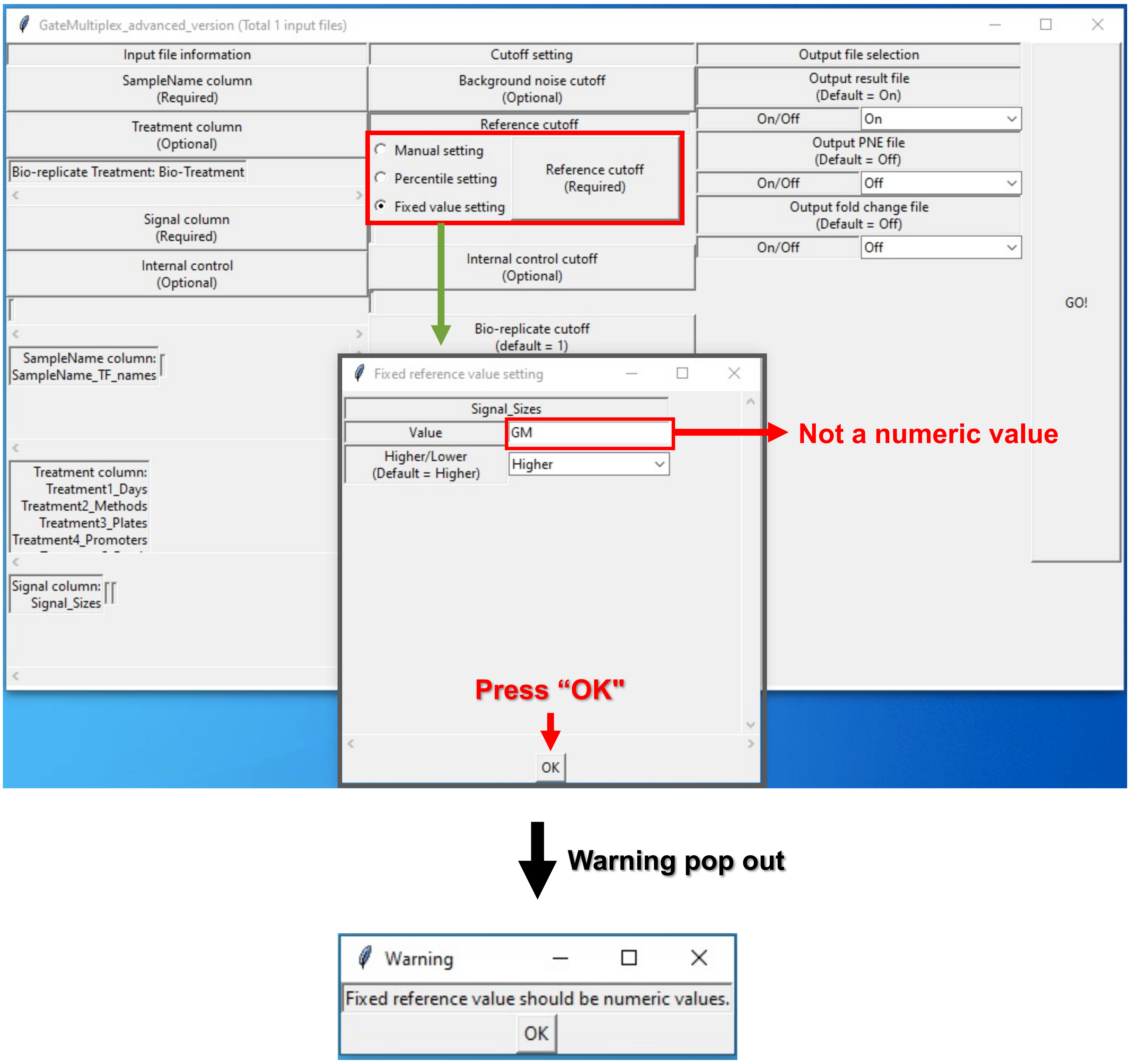

❖ If the internal control Treatment and internal control Condition are not both set or empty, the warning will pop out after pressing “OK”.

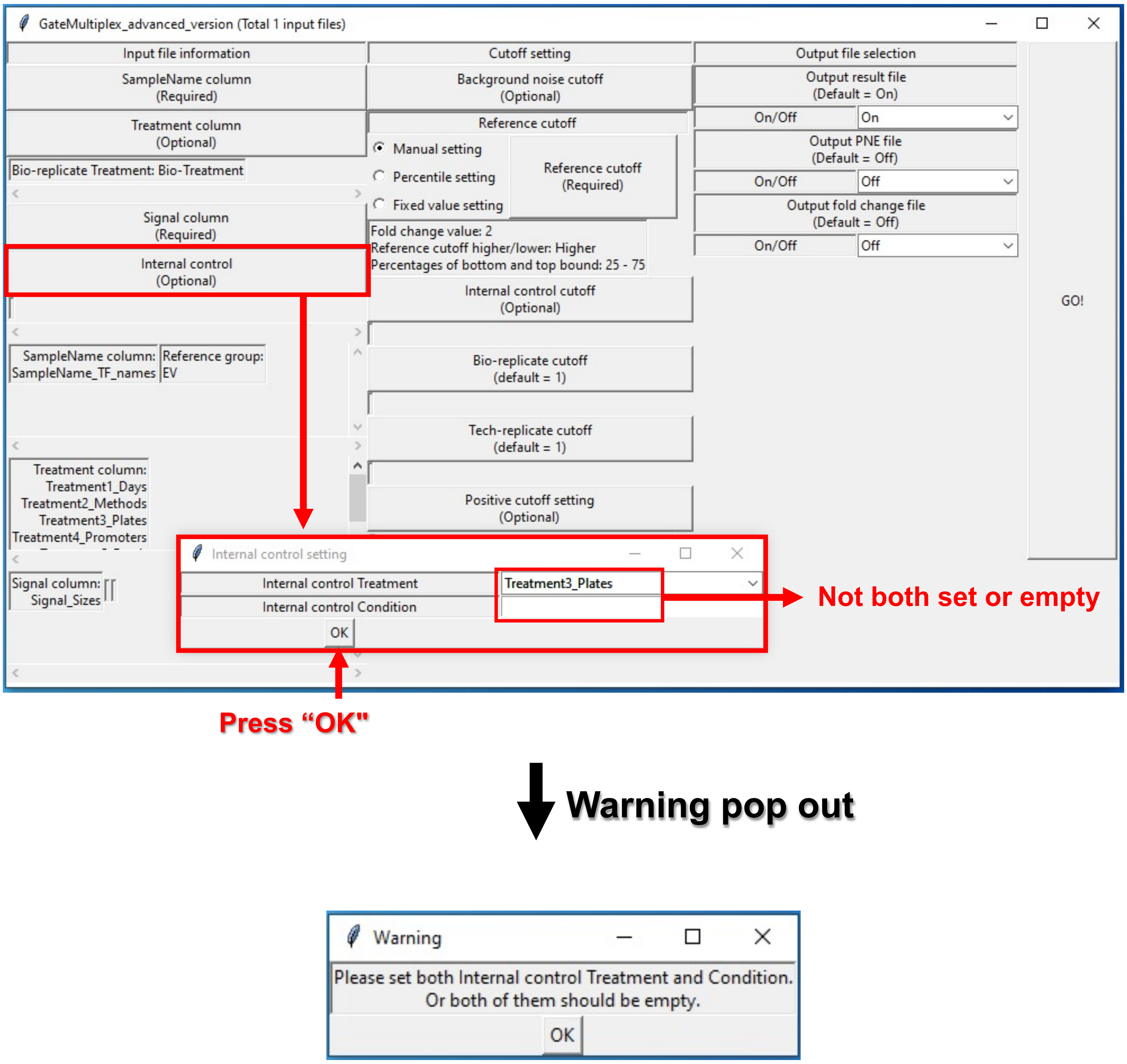

❖ If Internal control (internal control Treatment and internal control Condition) is set, but Internal control cutoff is not set, the warning will pop out after pressing “GO!”.

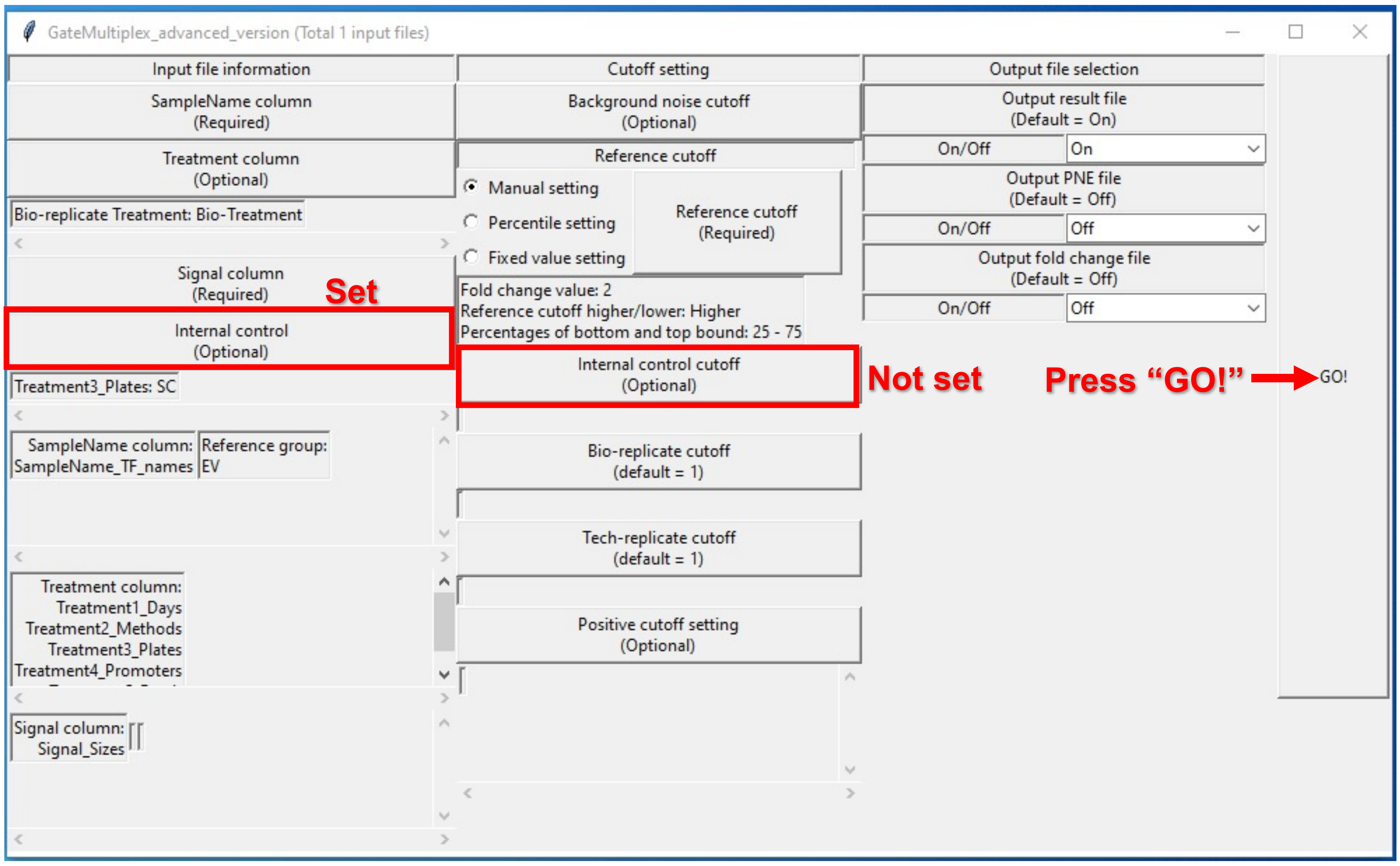

Warning pop out

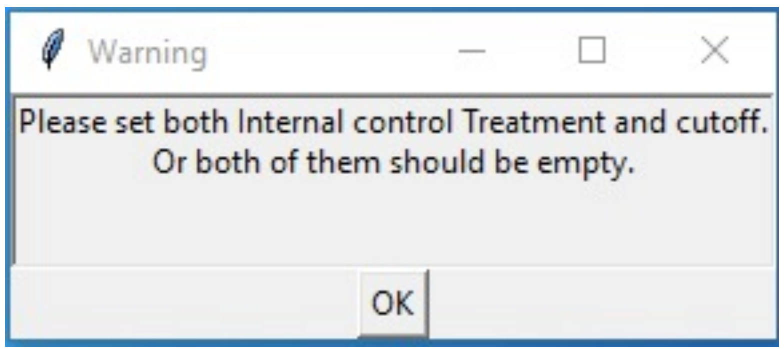

❖ If Internal control (internal control Treatment and internal control Condition) is not set, and Internal control cutoff is set, the warning will pop out after pressing "GO!".

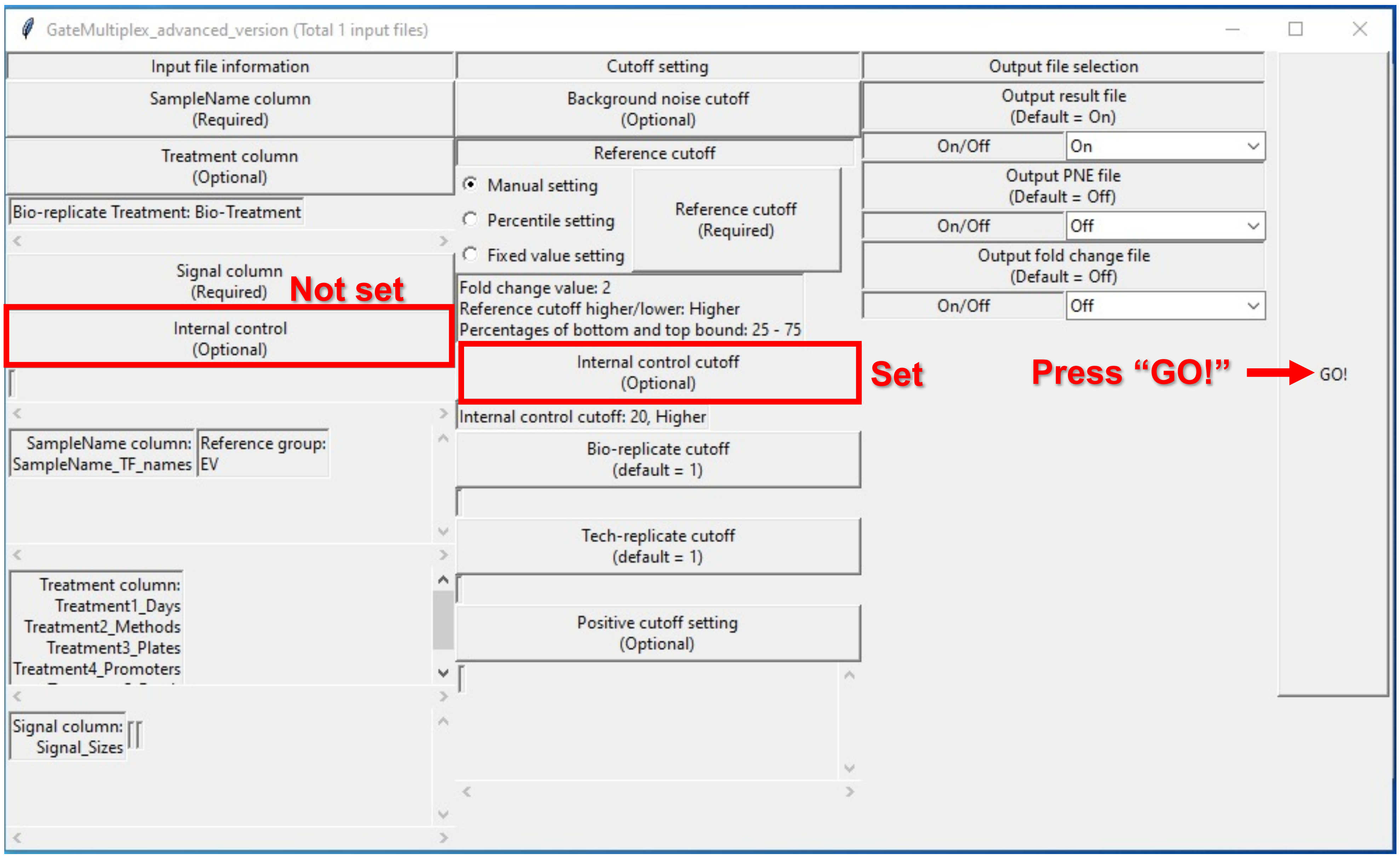

Warning pop out

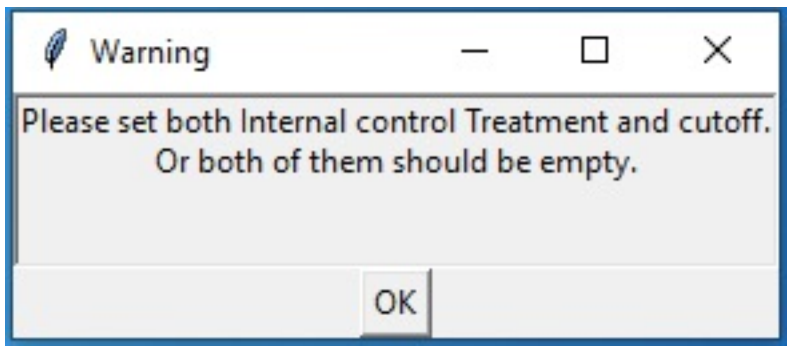

❖ If the value of positive cutoff is not a positive integer, the warning will pop out after pressing “OK”.

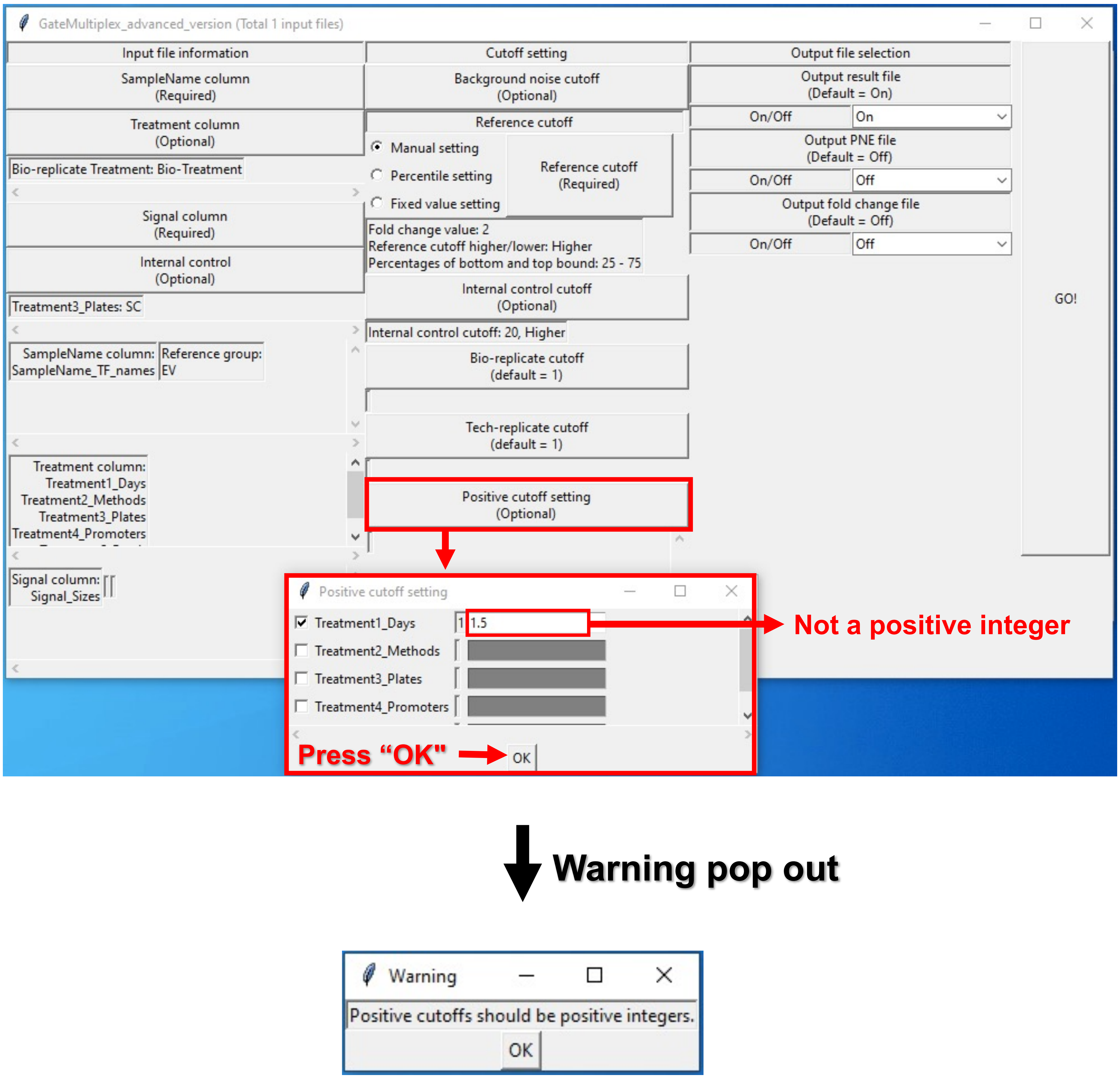

Supplement: Supplementary file 3 — Additional file 3. Manual-Y1H. A step-by-step manual for operating GateMultiplex on Y1H analysis. [file 12915_2021_1140_MOESM3_ESM.pdf]
