# Supplementary material for: Large-scale data analysis for robotic yeast one-hybrid platforms and multi-disciplinary studies using GateMultiplex
Source: BMC Biol. 2021 Sep 24;19:214. doi: 10.1186/s12915-021-01140-y (PMC8461970; doi:10.1186/s12915-021-01140-y)
Supplement: Supplementary file 4 — Additional file 4. Manual-Agriculture/Drug discovery/Geographical tracking. A step-by-step manual for operating GateMultiplex on Agriculture/Drug discovery/Geographical tracking analysis. [file 12915_2021_1140_MOESM4_ESM.pdf]

***NOTICE: To fully understand this manual, please read through the main text first**

— Index —

Preclinical lead compound identification (GM_Converter + GM_Basic) 2-52

Phenomic screening in precision agriculture (GM_Basic) 53-73

Geographical tracking for marine ecosystems (GM_Basic) 74-82

Preclinical lead compound identification
(GM_Converter + GM_Basic)

Preclinical lead compound identification (GM_Converter + GM_Basic)

❖ The location of Preclinical lead compound identification demo data

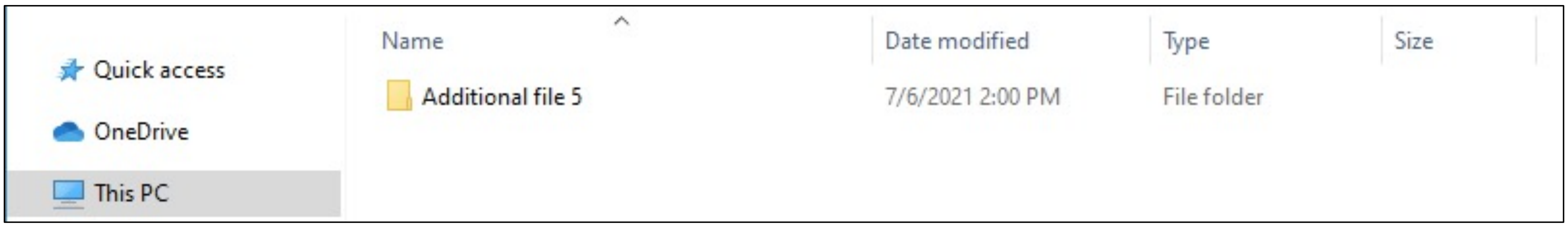

Open the "Additional file 5" folder

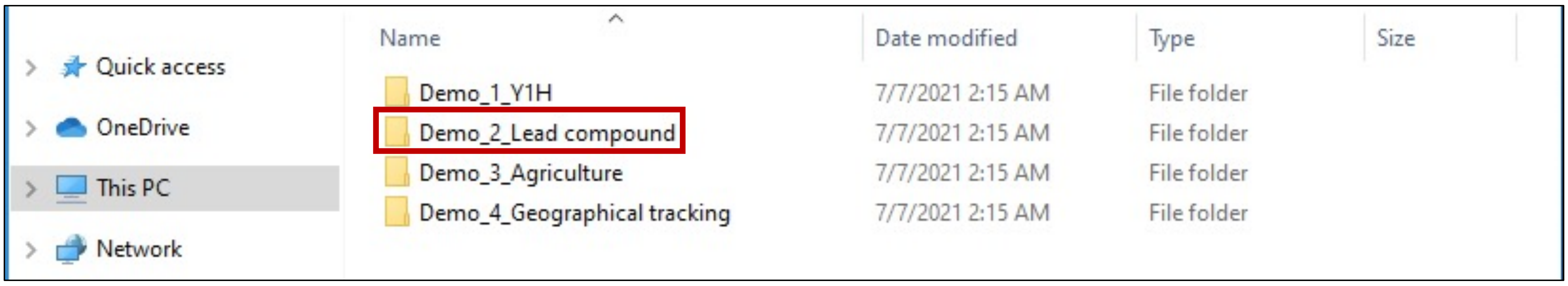

Open the "Demo_2_Lead compound" folder (red frame)

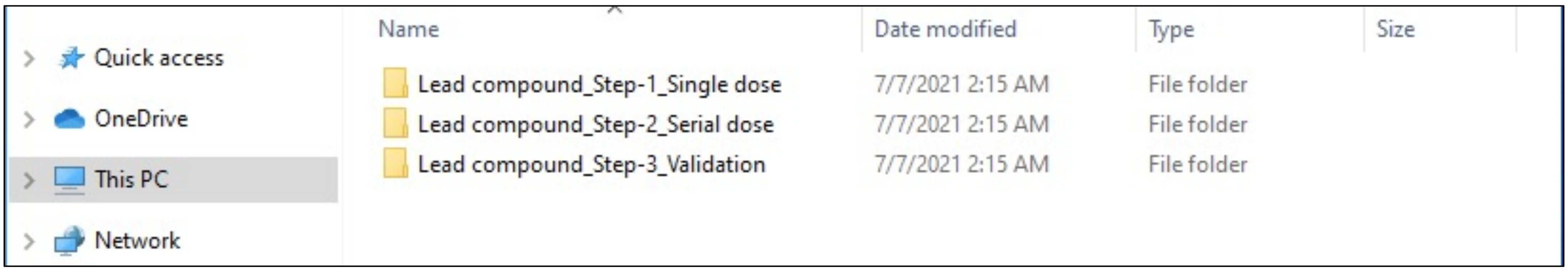

*The folder "Lead compound_Step-1_Single dose", "Lead compound_Step-2_Serial dose", and the " Lead compound_Step-3_Validation" are the demo data for Preclinical lead compound identification.

❖ Single dose (GM_Converter)

- The demo data of single dose is stored in “Lead compound_Step-1_Single dose” (green frame) folder of “Demo_2_Lead compound” folder (red frame).

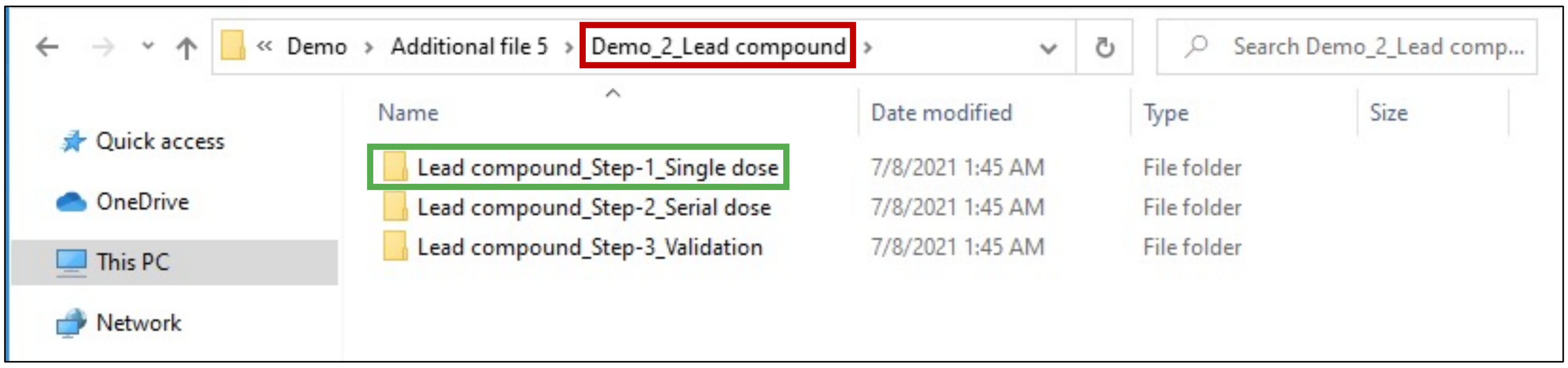

Open the “Lead compound_Step-1_Single dose” folder (green frame)

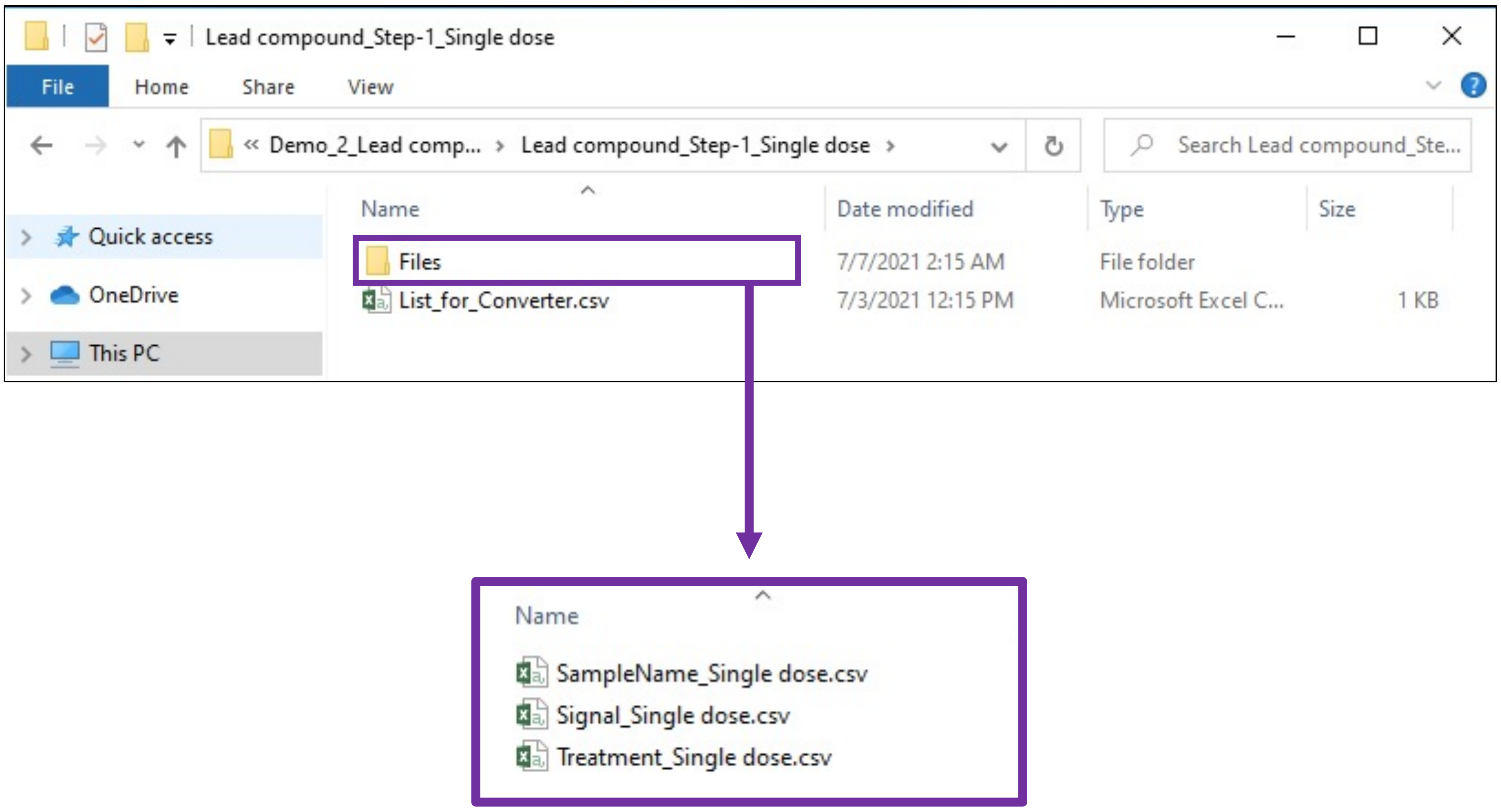

- Files in “Files” folder for using converter (Single dose)

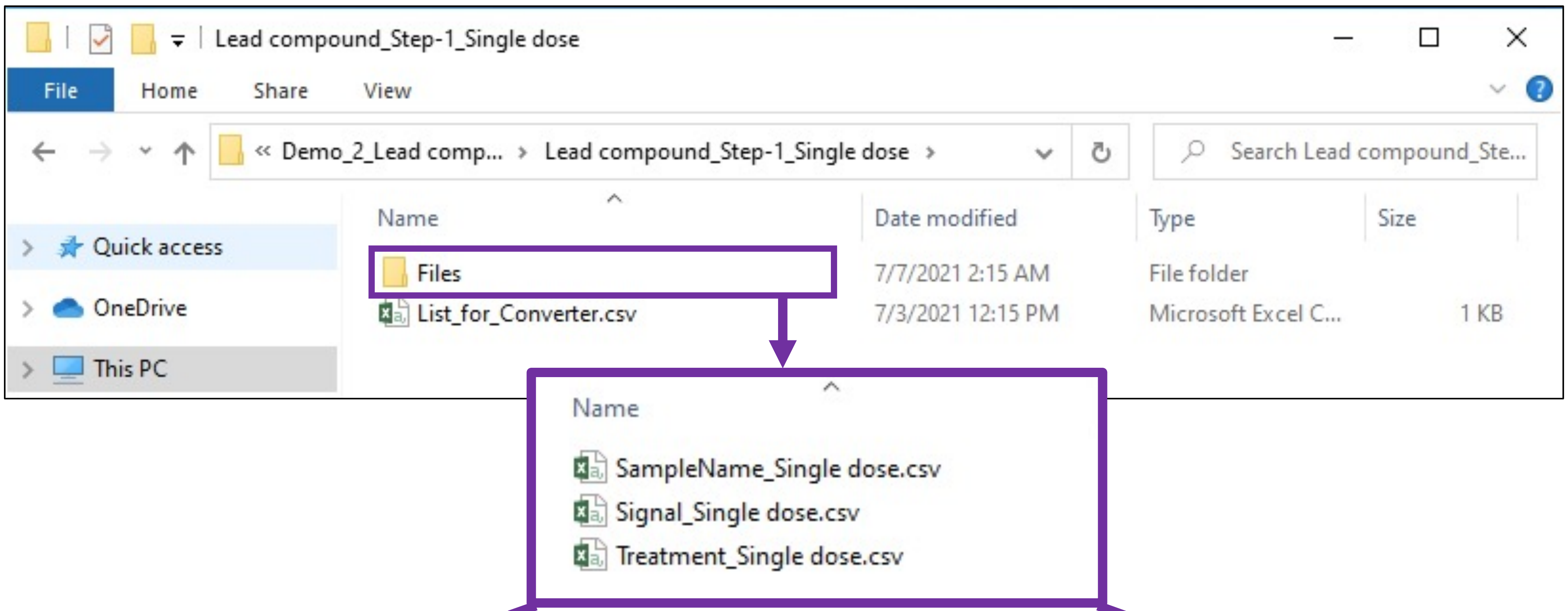

SampleName_Single dose.csv

	A	B	C	D	E	F	G	H	I	J	K
1	DMSO	sorafenib	lenvatinib	nilotinib	tranylcypromine hydrochloride	A769662	GSK2879552	GSK-LSD1	BIX01254	UNC0638	
2	DMSO	sorafenib	lenvatinib	nilotinib	tranylcypromine hydrochloride	A769662	GSK2879552	GSK-LSD1	BIX01254	UNC0638	
3	DMSO	sorafenib	lenvatinib	nilotinib	tranylcypromine hydrochloride	A769662	GSK2879552	GSK-LSD1	BIX01254	UNC0638	
4	DMSO	sorafenib	lenvatinib	nilotinib	tranylcypromine hydrochloride	A769662	GSK2879552	GSK-LSD1	BIX01254	UNC0638	
5	DMSO	sorafenib	lenvatinib	nilotinib	tranylcypromine hydrochloride	A769662	GSK2879552	GSK-LSD1	BIX01254	UNC0638	
6	DMSO	sorafenib	lenvatinib	nilotinib	tranylcypromine hydrochloride	A769662	GSK2879552	GSK-LSD1	BIX01254	UNC0638	
7											

SampleName

Treatment_Single dose.csv

	A	B	C	D	E	F	G	H	I	J
1	Compound plate-1	Compound plate-1	Compound plate-1	Compound plate-1	Compound plate-1	Compound plate-1	Compound plate-1	Compound plate-1	Compound plate-1	Compound plate-1
2	Compound plate-1	Compound plate-1	Compound plate-1	Compound plate-1	Compound plate-1	Compound plate-1	Compound plate-1	Compound plate-1	Compound plate-1	Compound plate-1
3	Compound plate-1	Compound plate-1	Compound plate-1	Compound plate-1	Compound plate-1	Compound plate-1	Compound plate-1	Compound plate-1	Compound plate-1	Compound plate-1
4	Compound plate-1	Compound plate-1	Compound plate-1	Compound plate-1	Compound plate-1	Compound plate-1	Compound plate-1	Compound plate-1	Compound plate-1	Compound plate-1
5	Compound plate-1	Compound plate-1	Compound plate-1	Compound plate-1	Compound plate-1	Compound plate-1	Compound plate-1	Compound plate-1	Compound plate-1	Compound plate-1
6	Compound plate-1	Compound plate-1	Compound plate-1	Compound plate-1	Compound plate-1	Compound plate-1	Compound plate-1	Compound plate-1	Compound plate-1	Compound plate-1
7										
8										

Treatment

Signal_Single dose.csv

	A	B	C	D	E	F	G	H	I	J	K	L	M	N
1		1	2	3	4	5	6	7	8	9	10	11	12	
2	A	0	0.03	0.033	0.032	0.033	0.033	0.035	0.034	0.033	0.033	0.034	-0.094	
3	B	0	0.194	0.048	0.146	0.117	0.173	0.157	0.165	0.163	0.094	0.127	-0.001	
4	C	0	0.176	0.055	0.172	0.118	0.173	0.164	0.182	0.171	0.097	0.122	-0.001	
5	D	0	0.162	0.055	0.16	0.117	0.181	0.177	0.189	0.185	0.112	0.123	-0.001	
6	E	0	0.174	0.055	0.173	0.132	0.18	0.168	0.177	0.169	0.106	0.126	-0.001	
7	F	0	0.175	0.049	0.145	0.115	0.153	0.142	0.153	0.15	0.08	0.115	-0.001	
8	G	0	0.177	0.053	0.145	0.101	0.145	0.13	0.148	0.142	0.09	0.116	-0.001	
9	H	0	0.031	0.031	0.032	0.032	.032	0.033	0.031	0.032	0.029	0.033	-0.001	
10														

Signal

List_for_Converter.csv"

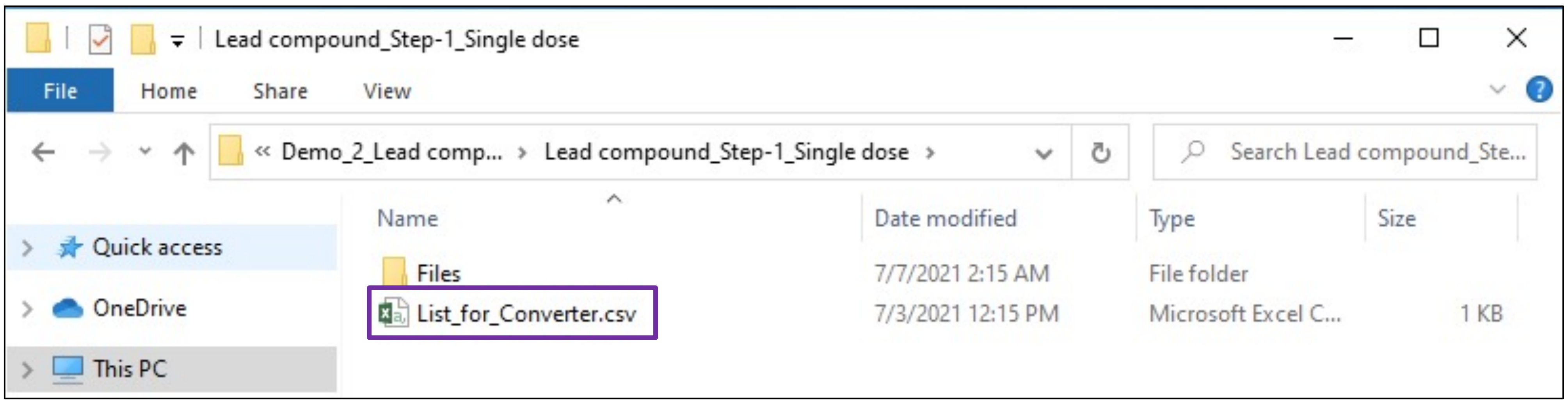

- The data in the first row of "Converted_data.csv" file (the result file output from GM_Converter) is from the first row of "List_for_Converter" file (red frame). Please see the Figure A1-A11 in Additional file 2 for the detailed concept of GM_Converter.

- The data of files listed in the same row of "List_for_Converter.csv" (circled by blue frame) will be integrated. Please see the Figure A1-A11 in Additional file 2 for the detailed concept of GM_Converter.

"Converted_data.csv"

First row →

	A	B	C
1	SampleName_Reagent	Treatment_Compound plate	Signal
2	DMSO	Compound plate-1	0.194
3	sorafenib	Compound plate-1	0.048
4	lenvatinib	Compound plate-1	0.146
5	nilotinib	Compound plate-1	0.117
6	tranylcypromine hydrochloride	Compound plate-1	0.173
7	A769662	Compound plate-1	0.157

"List_for_Converter.csv"

	A	B	C	D
1	SampleName_Reagent	Treatment_Compound plate	Signal	
2	SampleName_Single dose.csv	Treatment_Single dose.csv	Signal_Single dose.csv	
3				
4				

- Operation steps

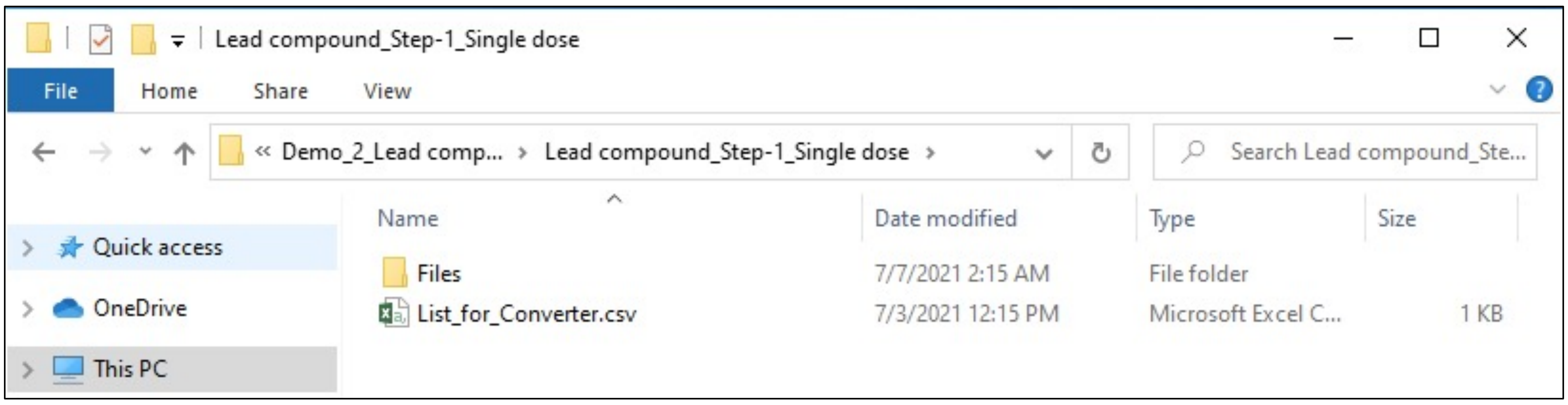

Copy and paste the two executive files of GM_Converter (GateMultiplex_converter.exe and Converter.exe) into "Lead compound_Step-1_Single dose" folder (purple frames and purple arrows)

*The two executive files should be placed in the same folder

- The "List_for_Converter.csv" (indicated by the green arrow) should be placed in the same folder with the two executive files of GM_Converter (GateMultiplex_converter.exe and Converter.exe).

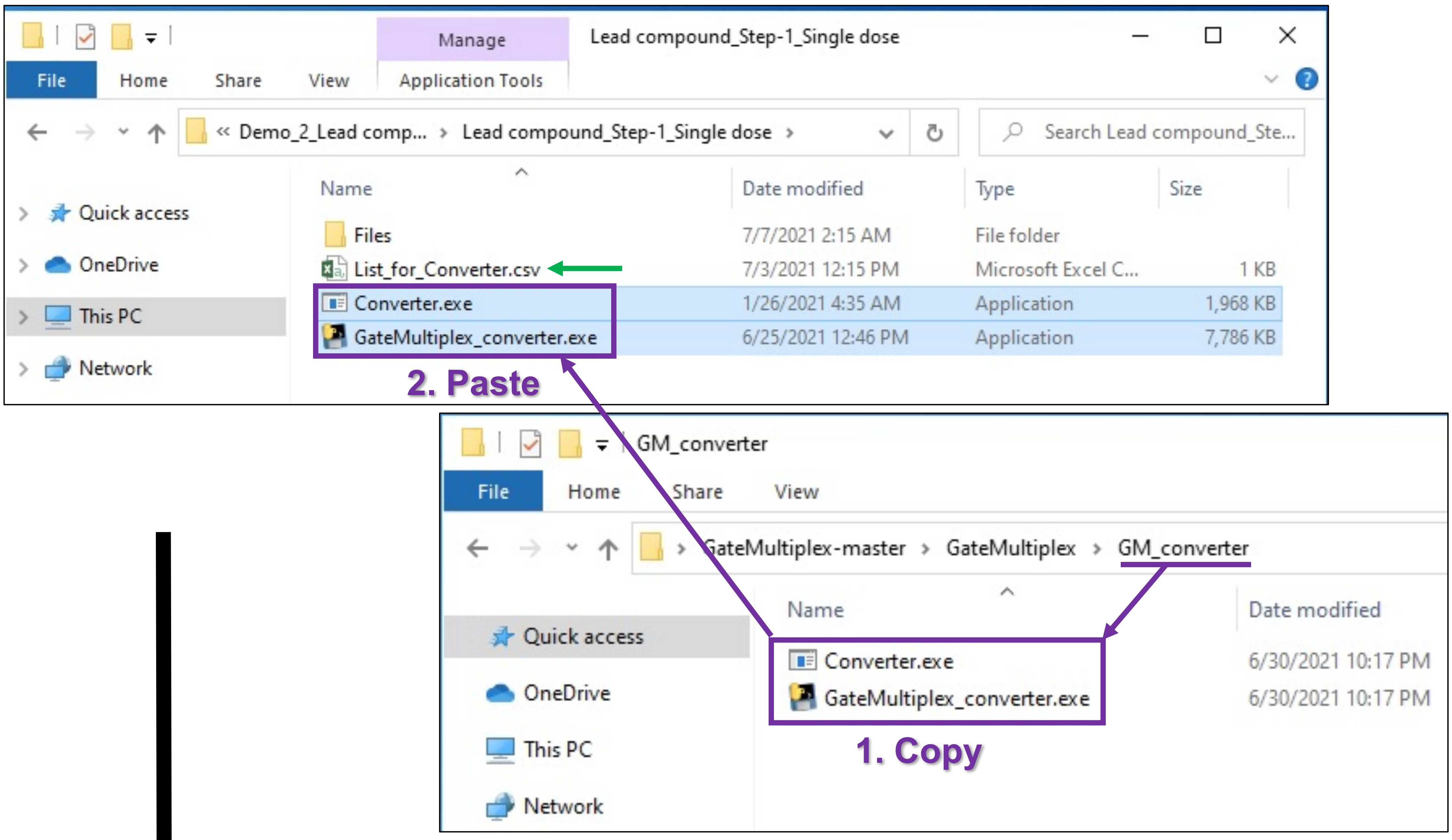

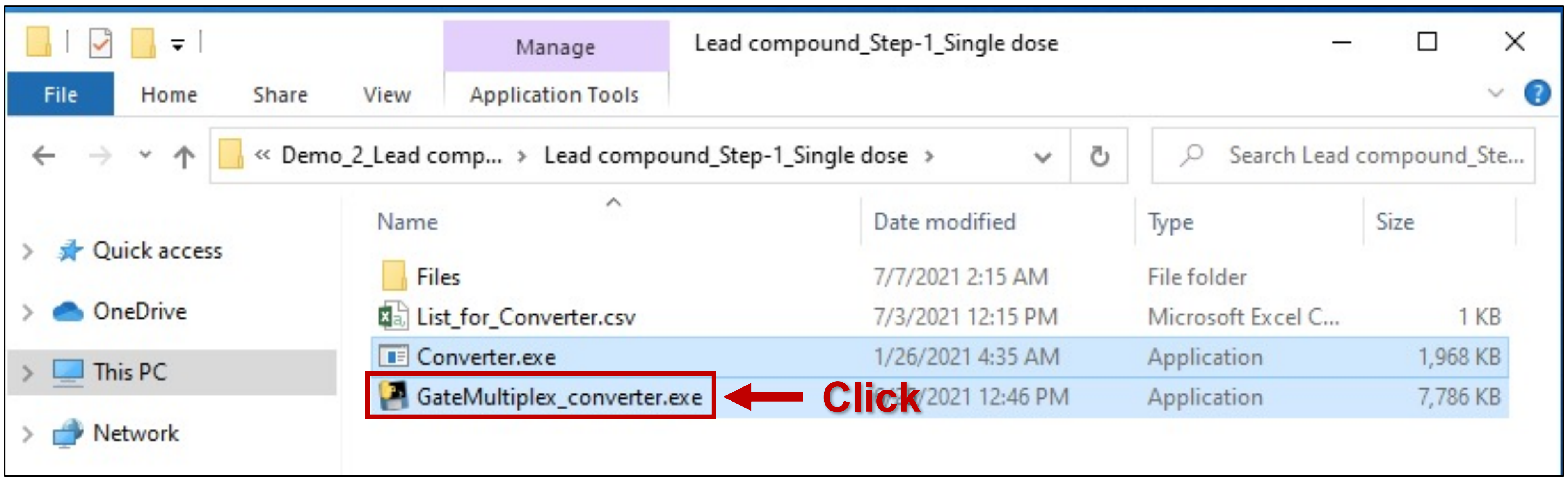

Activate the GM_Converter by a double-clicking on "GateMultiplex_converter.exe" (red frame)

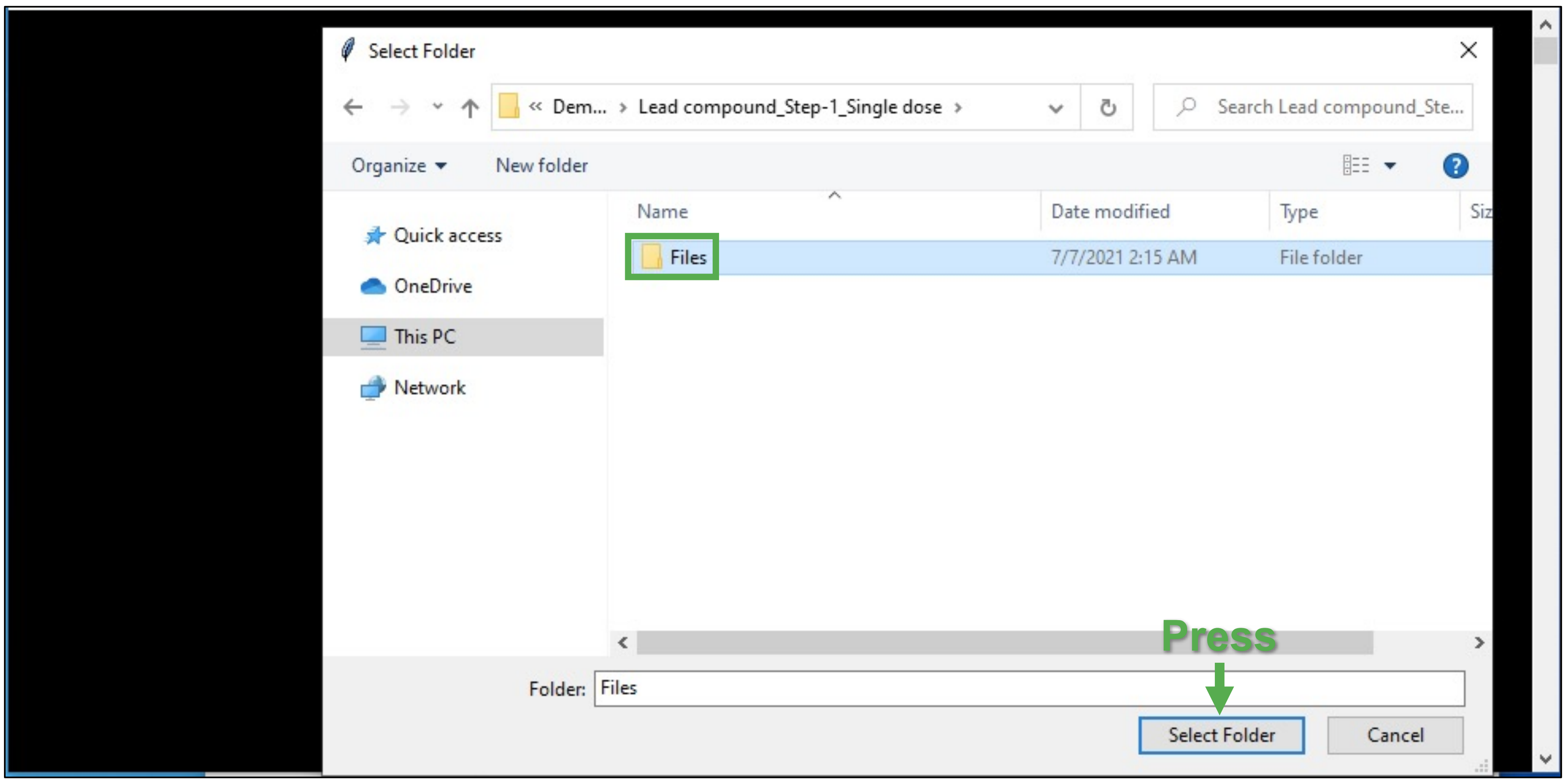

Select the folder "Files" (Additional file 5 > Demo_2_Lead compound > Lead compound_Step-1_Single dose > Files) (green frame) and press "Select Folder" (indicated by a green arrow)

- Please see the Figure A5-A11 in Additional file 2 for the detailed parameter setting of GM_Converter.

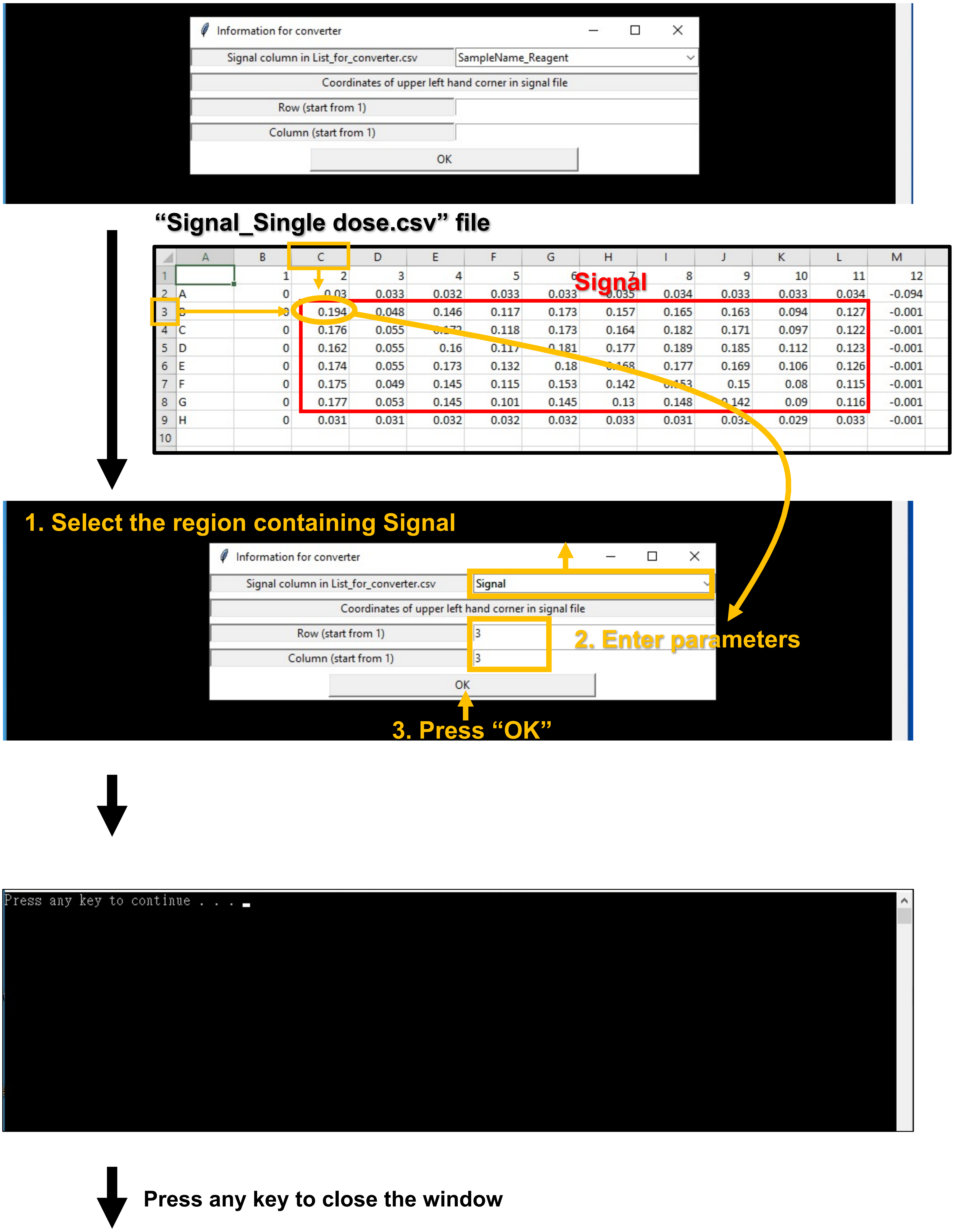

- The converted data “Converted_data.csv” (green frame) is stored in the “GM_converted_data” folder (red frame)

- The title of “Converted_data.csv” (circled by blue frame) is the same as the title in “List_for_Converter.csv”. Please see Figure A5-A11 in Additional file 2 for detailed explanation.

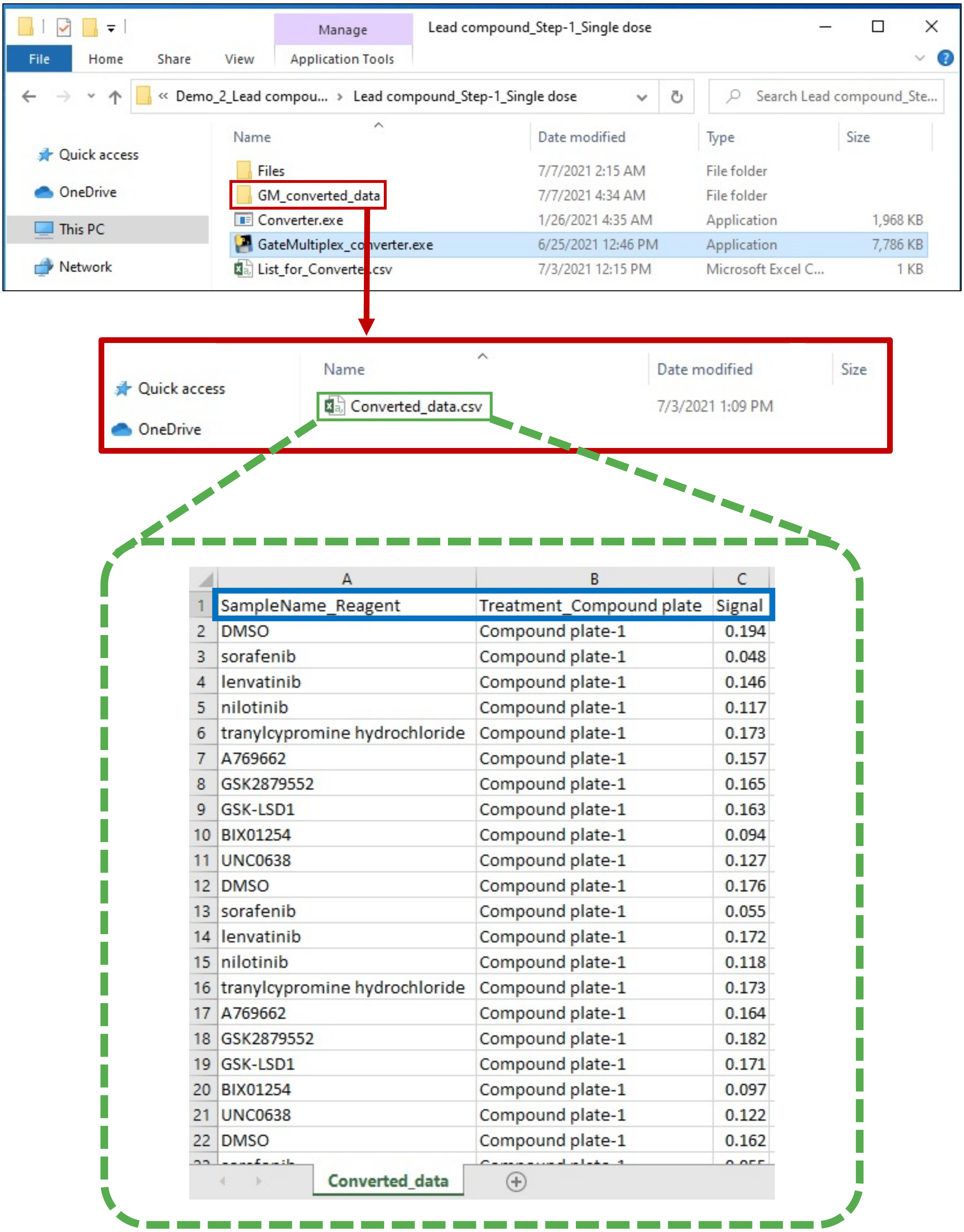

	A	B	C
1	SampleName_Reagent	Treatment_Compound plate	Signal
2	DMSO	Compound plate-1	0.194
3	sorafenib	Compound plate-1	0.048
4	lenvatinib	Compound plate-1	0.146
5	nilotinib	Compound plate-1	0.117
6	tranylcypromine hydrochloride	Compound plate-1	0.173
7	A769662	Compound plate-1	0.157
8	GSK2879552	Compound plate-1	0.165
9	GSK-LSD1	Compound plate-1	0.163
10	BIX01254	Compound plate-1	0.094
11	UNC0638	Compound plate-1	0.127
12	DMSO	Compound plate-1	0.176
13	sorafenib	Compound plate-1	0.055
14	lenvatinib	Compound plate-1	0.172
15	nilotinib	Compound plate-1	0.118
16	tranylcypromine hydrochloride	Compound plate-1	0.173
17	A769662	Compound plate-1	0.164
18	GSK2879552	Compound plate-1	0.182
19	GSK-LSD1	Compound plate-1	0.171
20	BIX01254	Compound plate-1	0.097
21	UNC0638	Compound plate-1	0.122
22	DMSO	Compound plate-1	0.162

❖ Single dose (GM_Basic)

- After Converted_data.csv completing, GM_Basic is further applied for analysis.
- The two executive files (GateMultiplex_basic.exe and GateMultiplex_forWindows.exe) of GM_Basic are both stored in the "GM_basic" folder (blue frame) and should be placed in the same folder for operation.
- Please see Fig. S8 and S9 in Additional file 1.

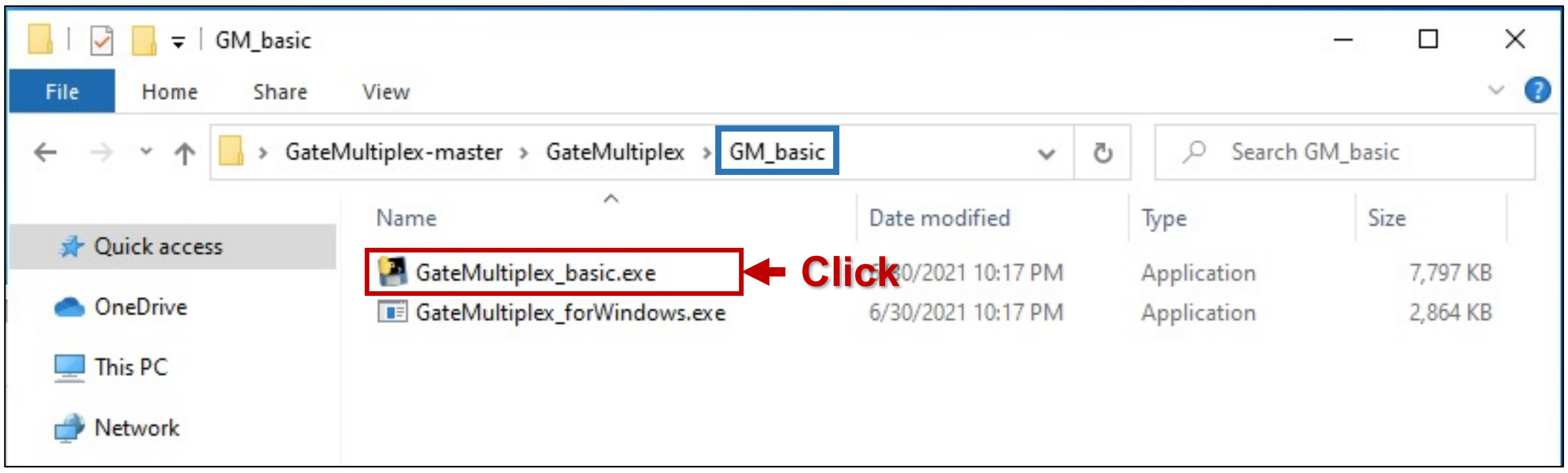

Activate the GM_basic by a double-clicking on "GateMultiplex_basic.exe" (red frame)

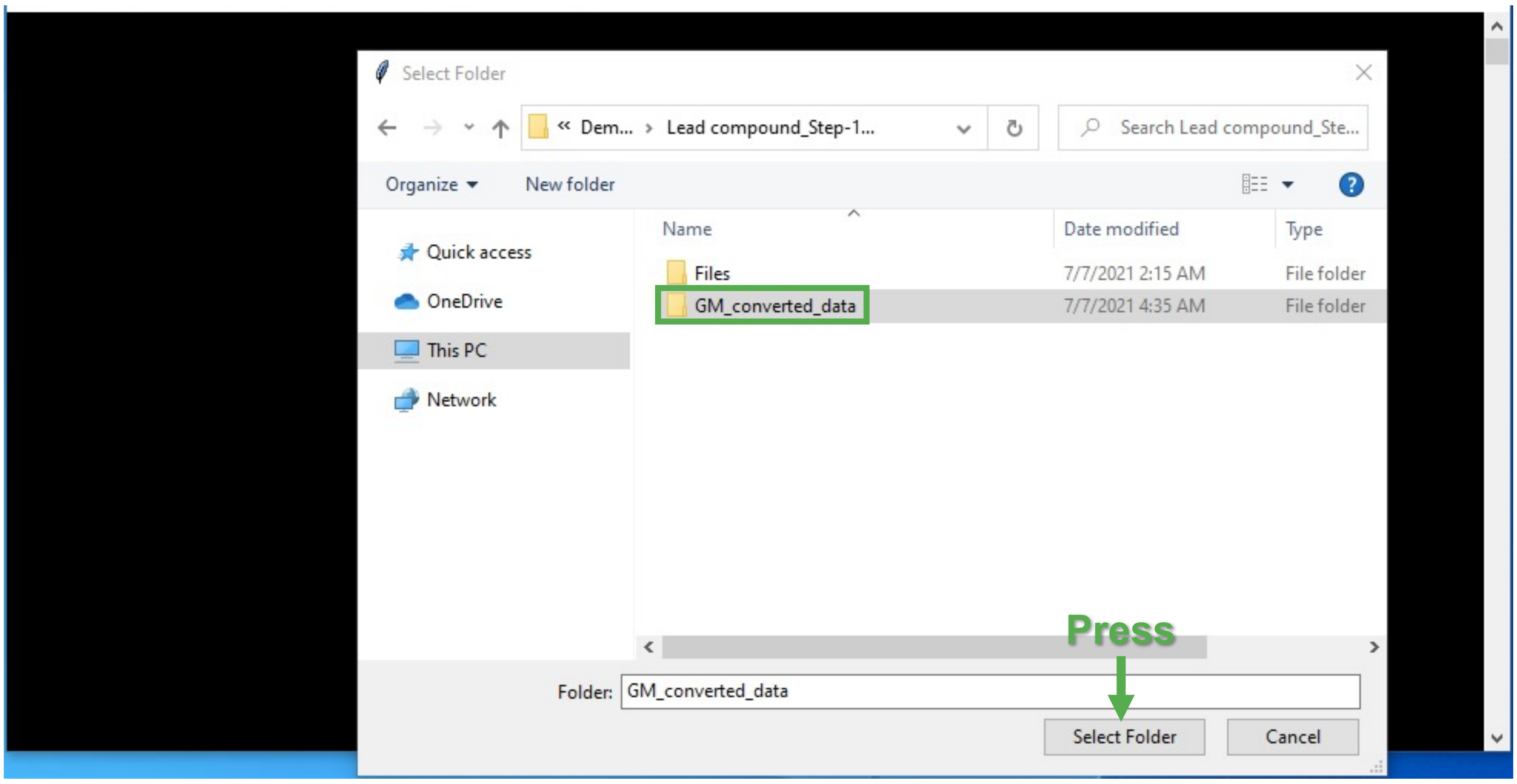

Select the folder "GM_converted_data" (Directory: Additional file 5 > Demo_2_Lead compound > Lead compound_Step-1_Single dose > GM_converted_data) (green frame) and press "Select Folder" (indicated by a green arrow)

✱ The GUI of GM_Basic

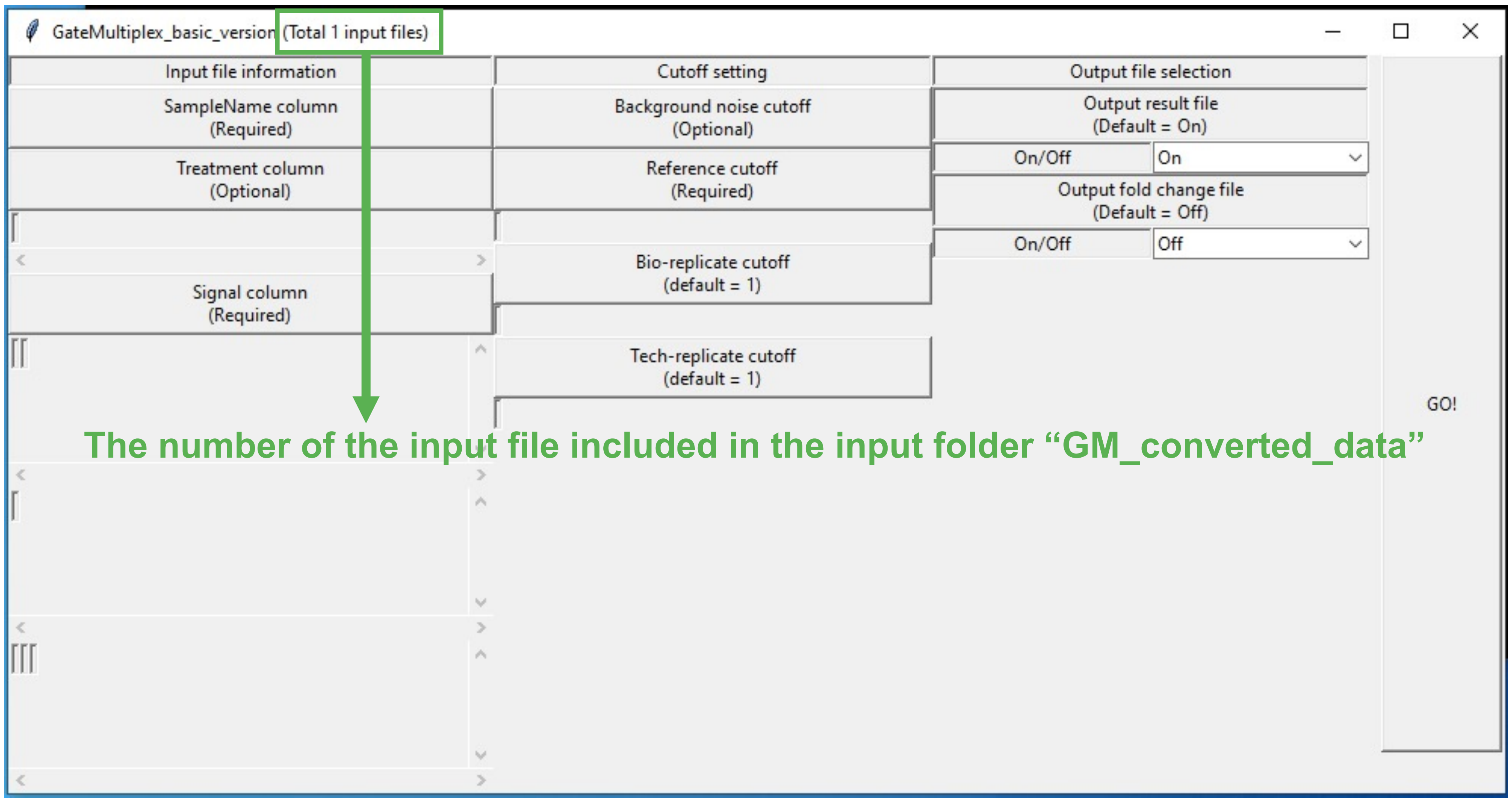

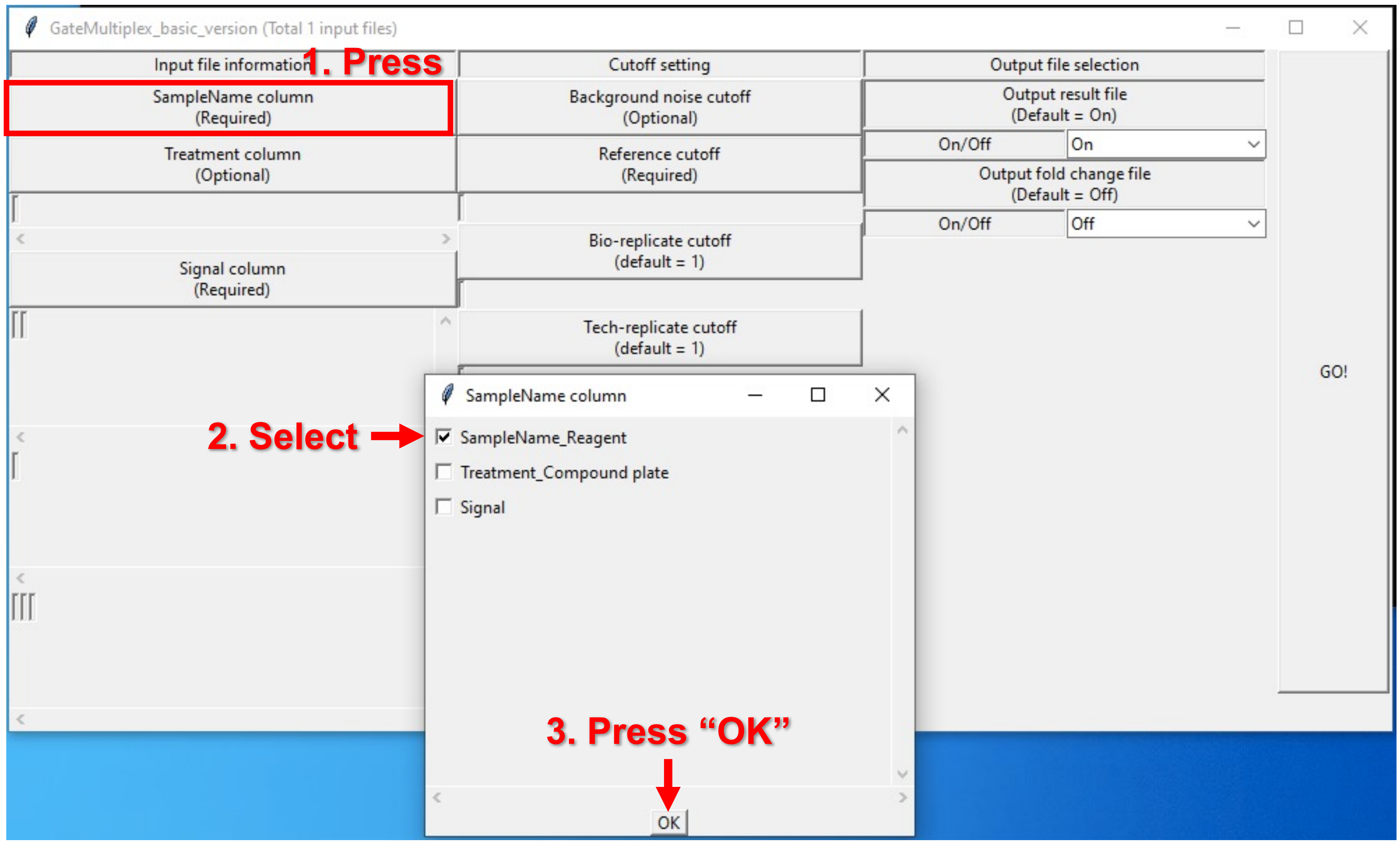

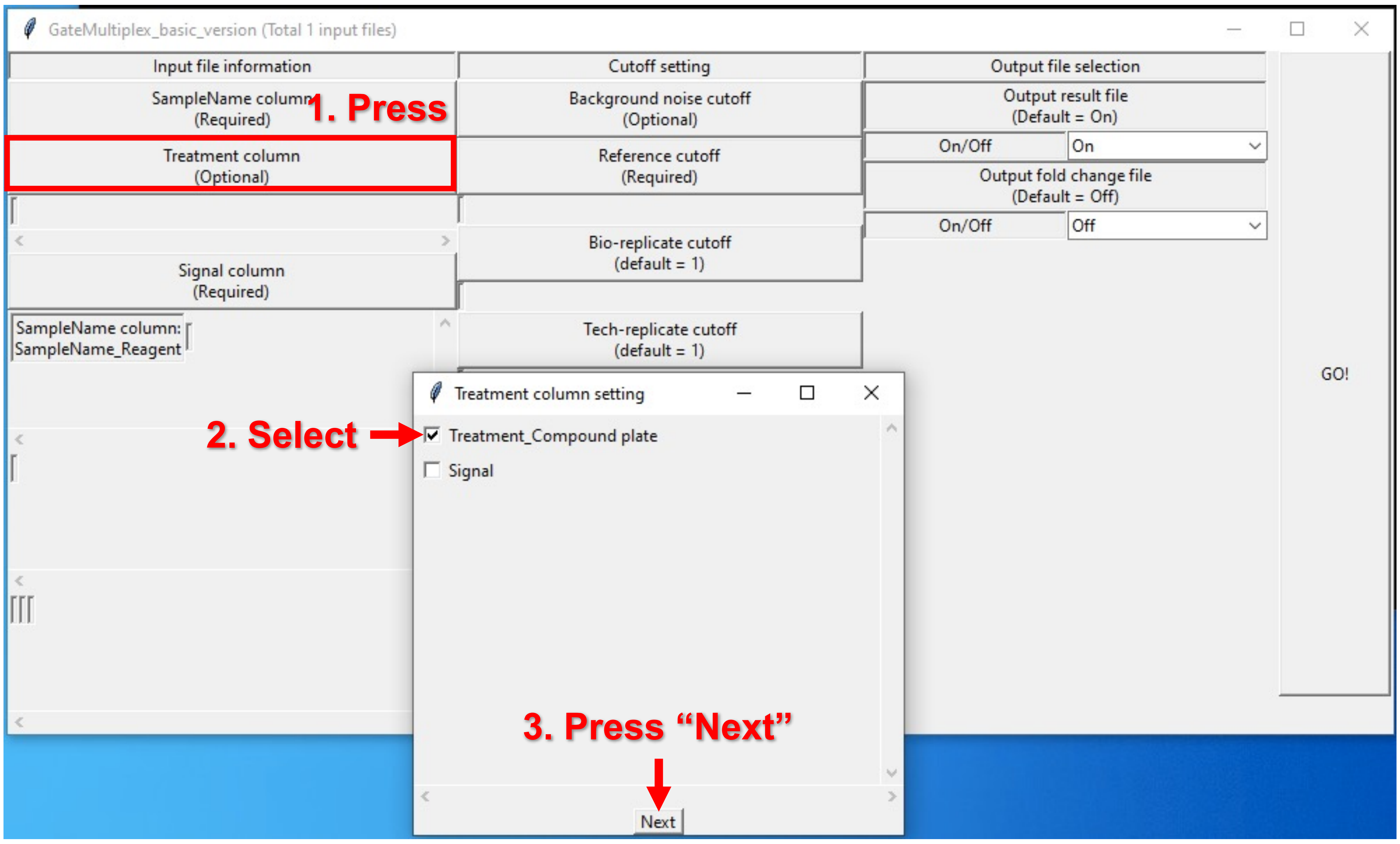

GateMultiplex_basic_version (Total 1 input files)
Input file information
SampleName column (Required)
1. Press
Treatment column (Optional)
Signal column (Required)
SampleName column: SampleName_Reagent
Cutoff setting
Background noise cutoff (Optional)
Reference cutoff (Required)
Bio-replicate cutoff (default = 1)
Tech-replicate cutoff (default = 1)
Output file selection
Output result file (Default = On)
On/Off
On
Output fold change file (Default = Off)
On/Off
Off
GO!
Treatment column setting
2. Select
Treatment_Compound plate
Signal
3. Press “Next”
Next

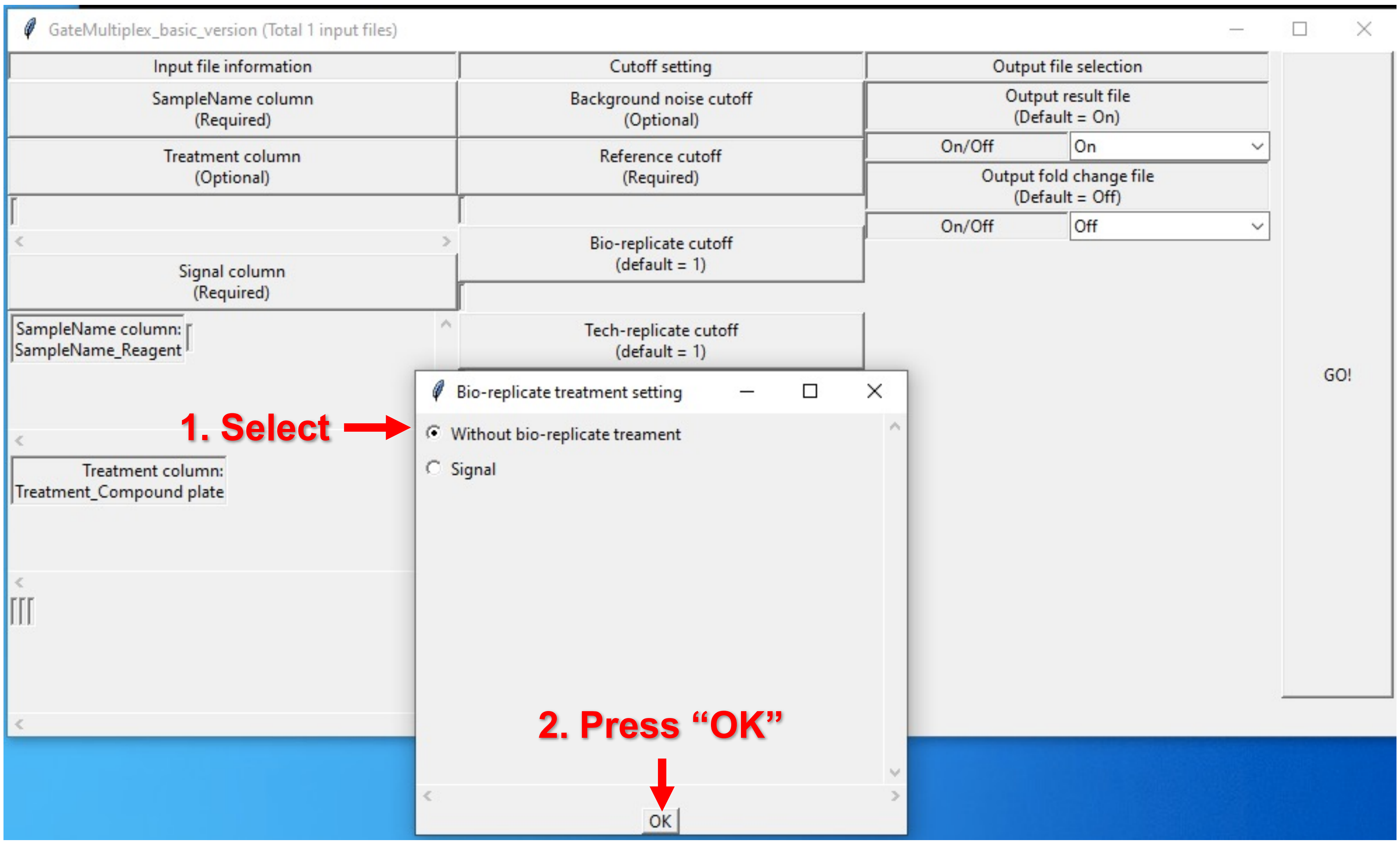

GateMultiplex_basic_version (Total 1 input files)
Input file information
SampleName column (Required)
Treatment column (Optional)
Signal column (Required)
SampleName column: SampleName_Reagent
Treatment column: Treatment_Compound plate
Cutoff setting
Background noise cutoff (Optional)
Reference cutoff (Required)
Bio-replicate cutoff (default = 1)
Tech-replicate cutoff (default = 1)
Output file selection
Output result file (Default = On)
On/Off
On
Output fold change file (Default = Off)
On/Off
Off
GO!
Bio-replicate treatment setting
1. Select
Without bio-replicate treament
Signal
2. Press “OK”
OK

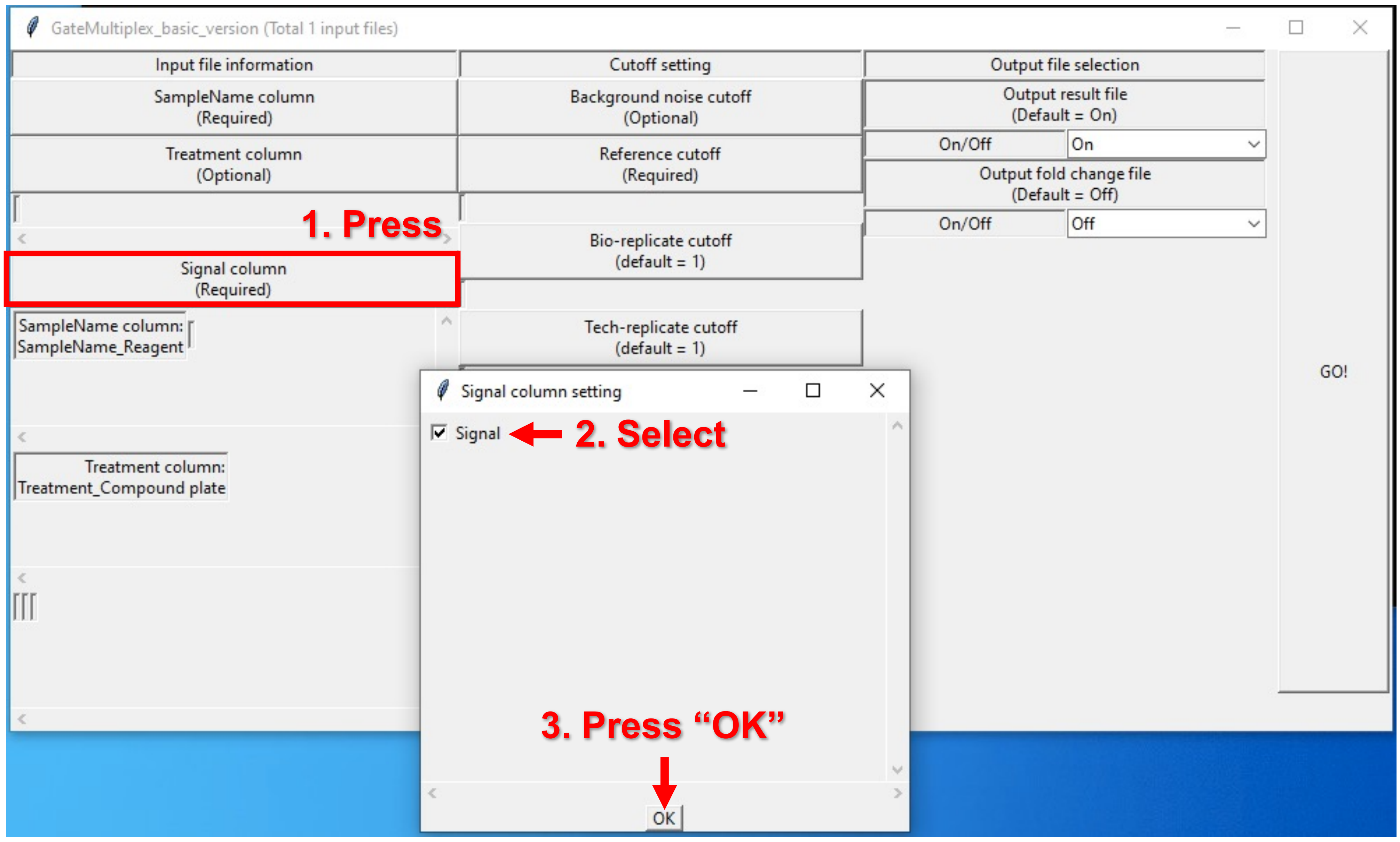
GateMultiplex_basic_version (Total 1 input files)
Input file information
SampleName column (Required)
Treatment column (Optional)
1. Press
Signal column (Required)
SampleName column: SampleName_Reagent
Treatment column: Treatment_Compound plate
Cutoff setting
Background noise cutoff (Optional)
Reference cutoff (Required)
Bio-replicate cutoff (default = 1)
Tech-replicate cutoff (default = 1)
Output file selection
Output result file (Default = On)
On/Off On
Output fold change file (Default = Off)
On/Off Off
GO!
Signal column setting
Signal
2. Select
3. Press "OK"
OK

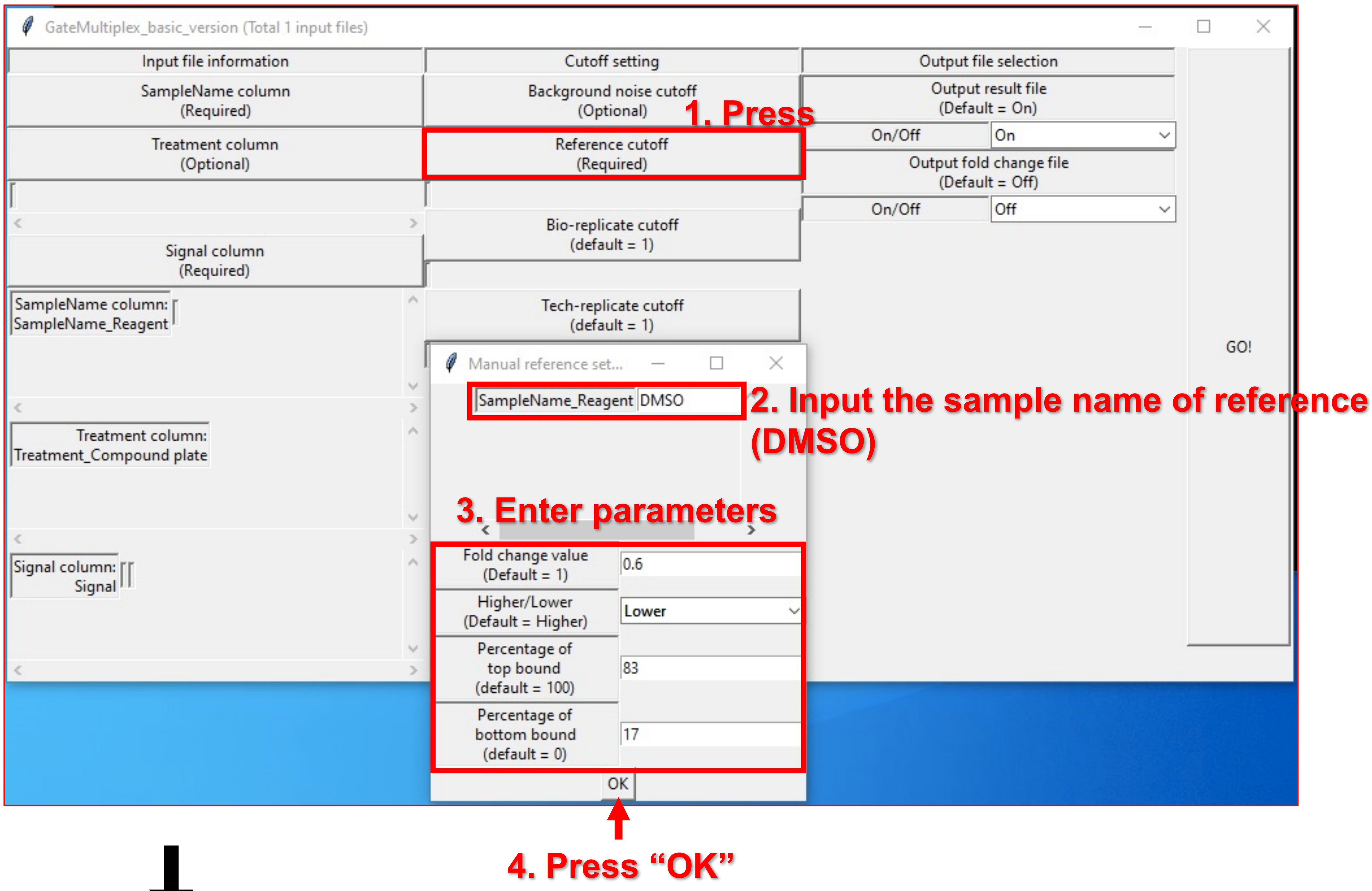
GateMultiplex_basic_version (Total 1 input files)
Input file information
SampleName column (Required)
Treatment column (Optional)
Signal column (Required)
SampleName column: SampleName_Reagent
Treatment column: Treatment_Compound plate
Signal column: Signal
Cutoff setting
Background noise cutoff (Optional)
1. Press
Reference cutoff (Required)
Bio-replicate cutoff (default = 1)
Tech-replicate cutoff (default = 1)
Output file selection
Output result file (Default = On)
On/Off On
Output fold change file (Default = Off)
On/Off Off
GO!
Manual reference set...
SampleName_Reagent DMSO
2. Input the sample name of reference (DMSO)
3. Enter parameters
Fold change value (Default = 1) 0.6
Higher/Lower (Default = Higher) Lower
Percentage of top bound (default = 100) 83
Percentage of bottom bound (default = 0) 17
OK
4. Press "OK"

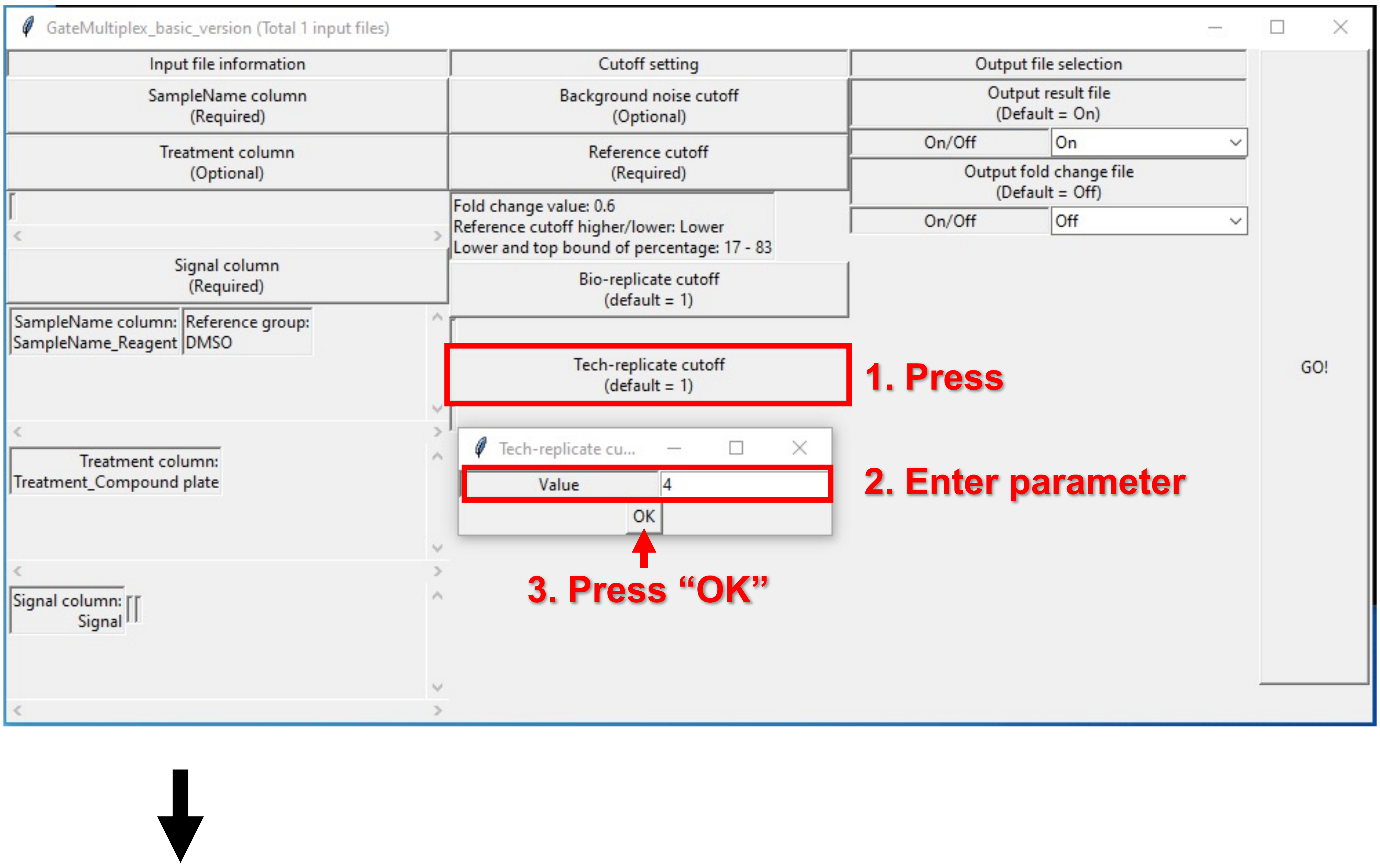
GateMultiplex_basic_version (Total 1 input files)
Input file information
SampleName column (Required)
Treatment column (Optional)
Signal column (Required)
SampleName column: SampleName_Reagent
Reference group: DMSO
Treatment column: Treatment_Compound plate
Signal column: Signal
Cutoff setting
Background noise cutoff (Optional)
Reference cutoff (Required)
Fold change value: 0.6
Reference cutoff higher/lower: Lower
Lower and top bound of percentage: 17 - 83
Bio-replicate cutoff (default = 1)
Tech-replicate cutoff (default = 1)
1. Press
Tech-replicate cu...
Value 4
OK
2. Enter parameter
3. Press “OK”
Output file selection
Output result file (Default = On)
On/Off On
Output fold change file (Default = Off)
On/Off Off
GO!

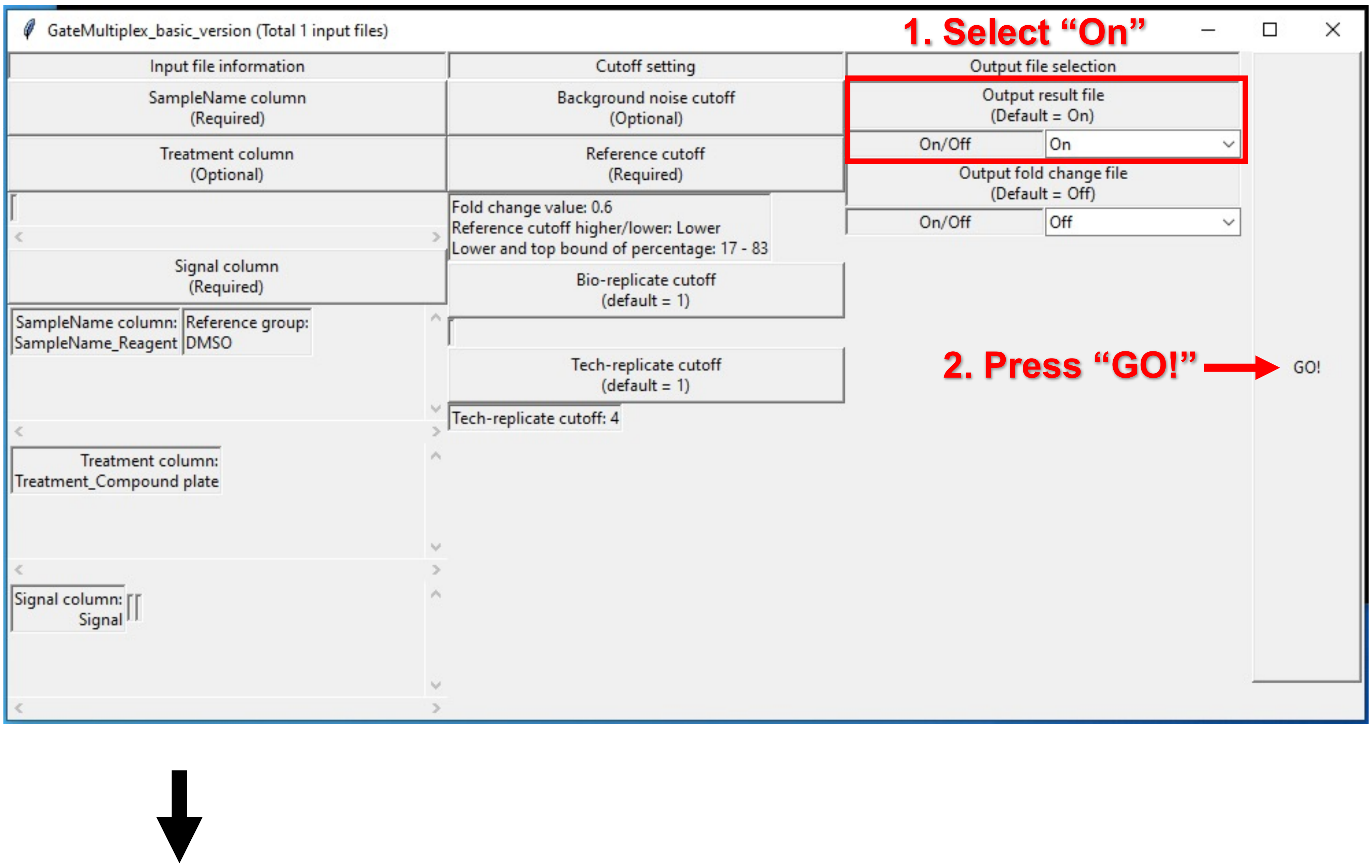
GateMultiplex_basic_version (Total 1 input files)
1. Select “On”
Input file information
SampleName column (Required)
Treatment column (Optional)
Signal column (Required)
SampleName column: SampleName_Reagent
Reference group: DMSO
Treatment column: Treatment_Compound plate
Signal column: Signal
Cutoff setting
Background noise cutoff (Optional)
Reference cutoff (Required)
Fold change value: 0.6
Reference cutoff higher/lower: Lower
Lower and top bound of percentage: 17 - 83
Bio-replicate cutoff (default = 1)
Tech-replicate cutoff (default = 1)
Tech-replicate cutoff: 4
Output file selection
Output result file (Default = On)
On/Off On
Output fold change file (Default = Off)
On/Off Off
2. Press “GO!”
GO!

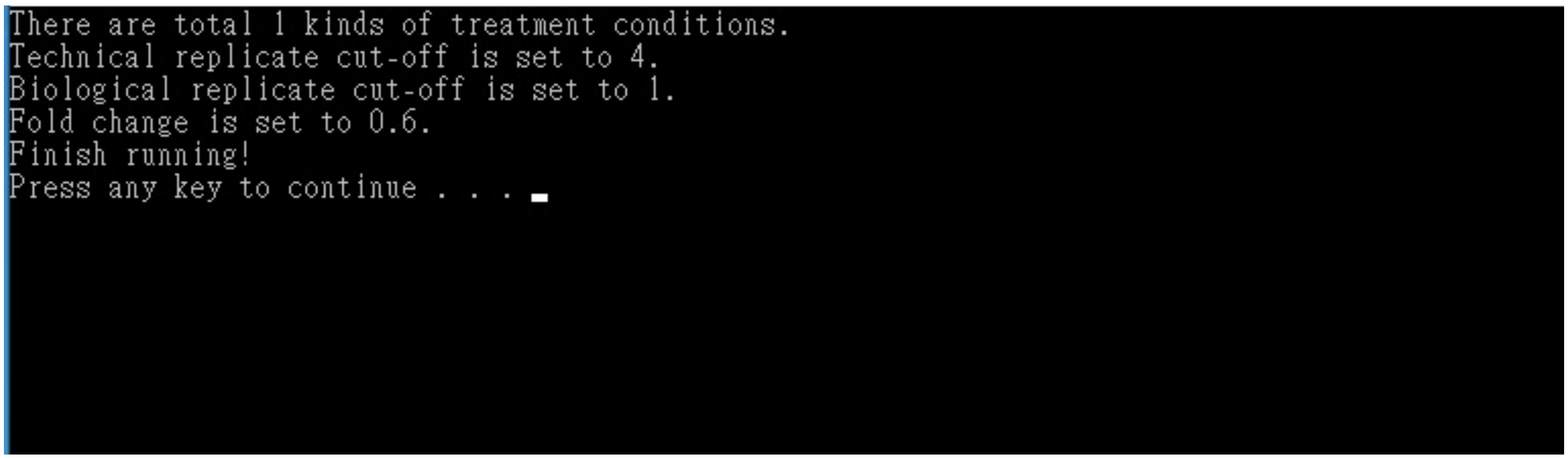

Press any key to close the window

- The result file, named "Results.csv" (red frame), is stored in the same folder with executive files of GM_Basic.
- The symbol "N" means negative and the symbol "P" means positive. Please see the "Symbols in output files" section in Additional file 3.

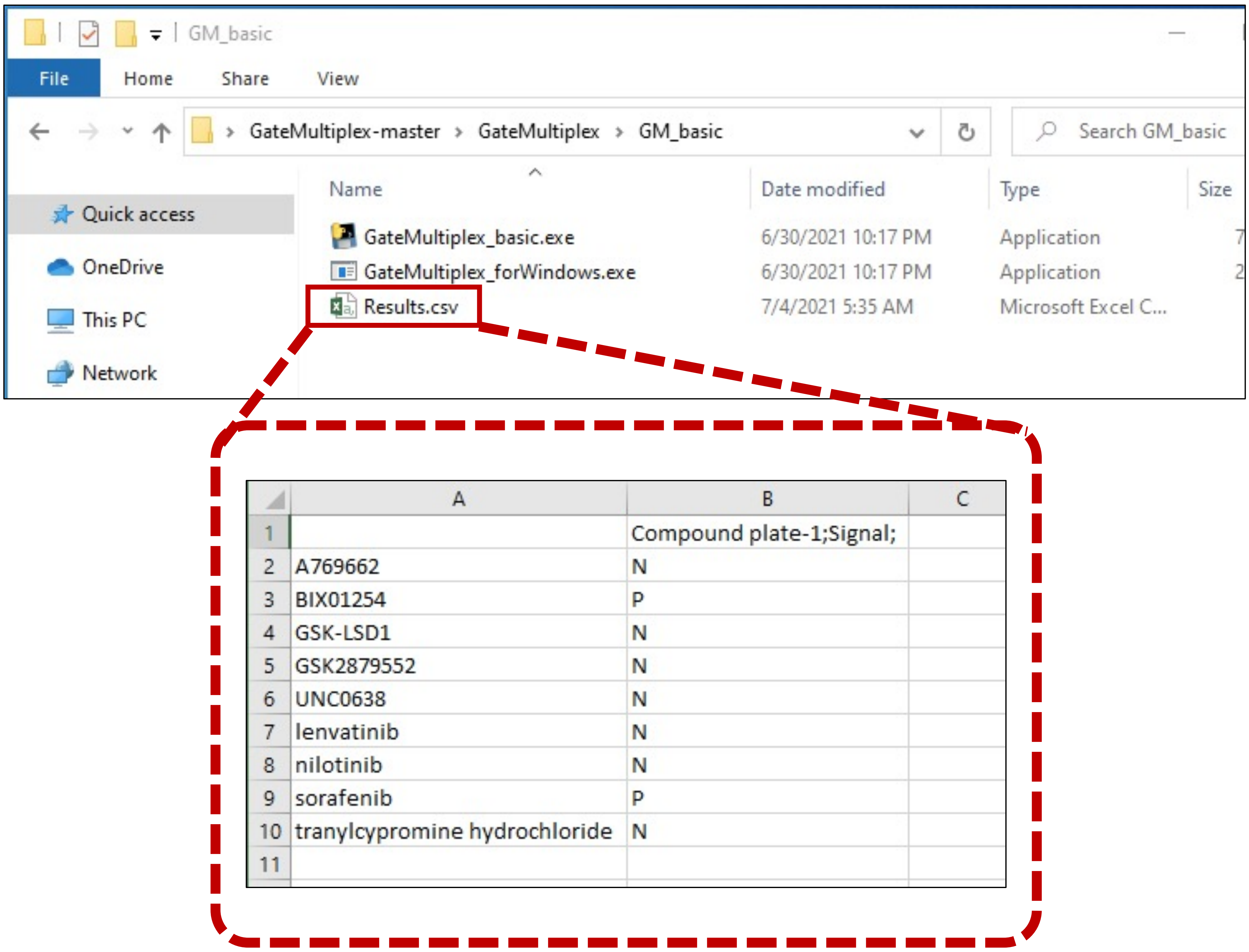

	A	B	C
1		Compound plate-1;Signal;	
2	A769662	N	
3	BIX01254	P	
4	GSK-LSD1	N	
5	GSK2879552	N	
6	UNC0638	N	
7	lenvatinib	N	
8	nilotinib	N	
9	sorafenib	P	
10	tranylcypromine hydrochloride	N	
11			

❖ Serial dose (GM_Converter)

- The demo data of serial dose is stored in “Lead compound_Step-2_Serial dose” (green frame) folder of “Demo_2_Lead compound” folder (red frame).

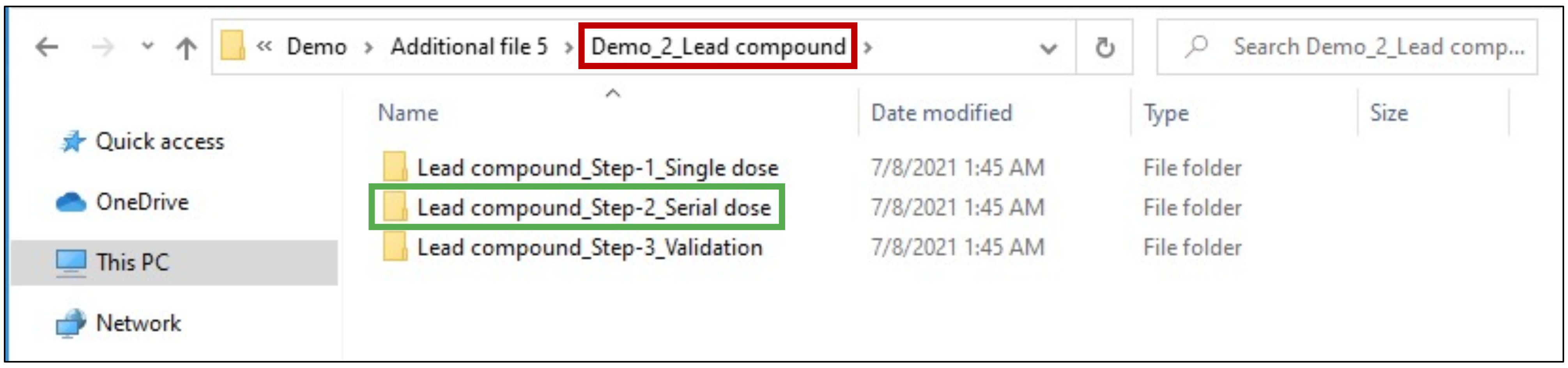

Open the “Lead compound_Step-2_Serial dose” folder (green frame)

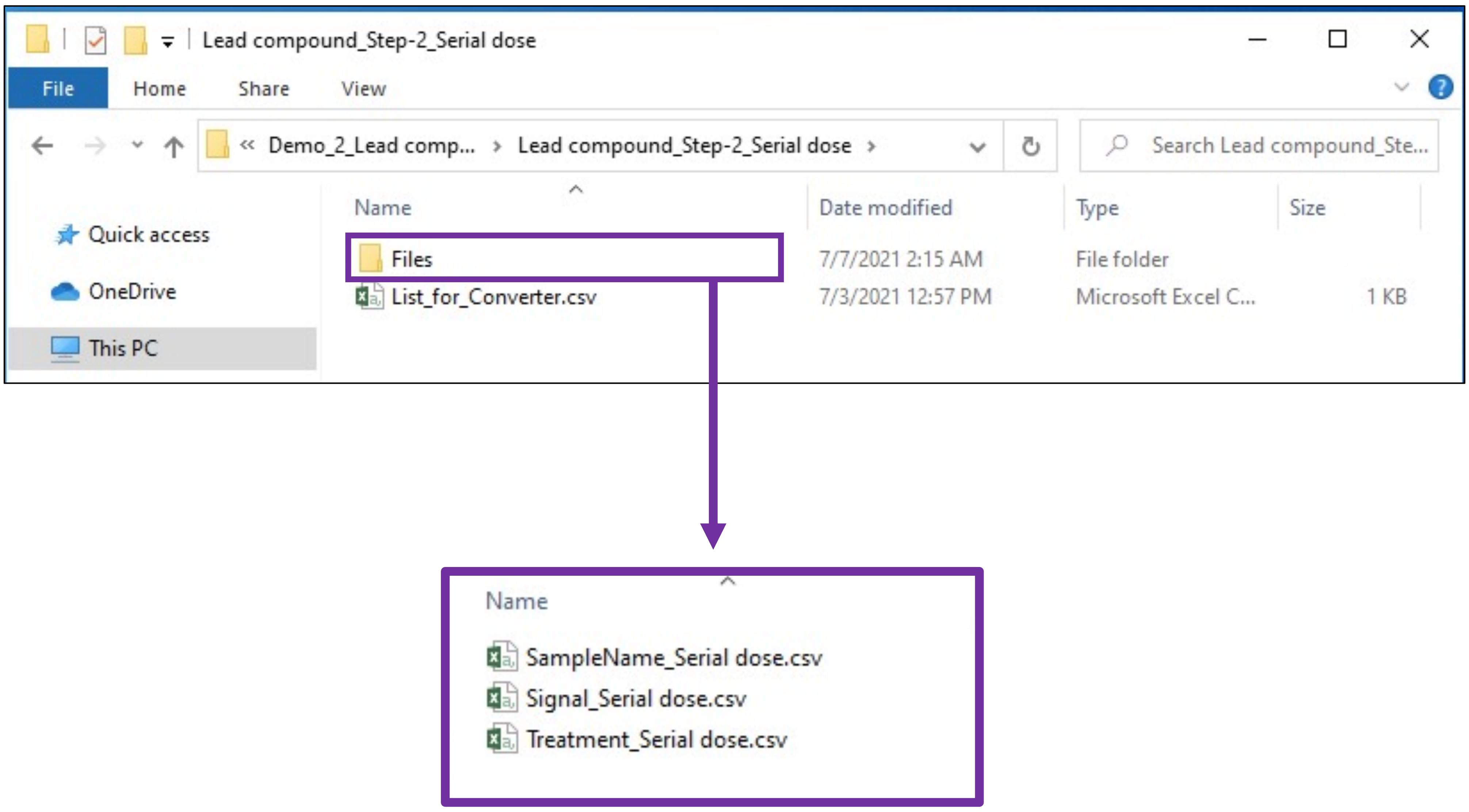

- Files in “Files” folder for using converter (Serial dose)

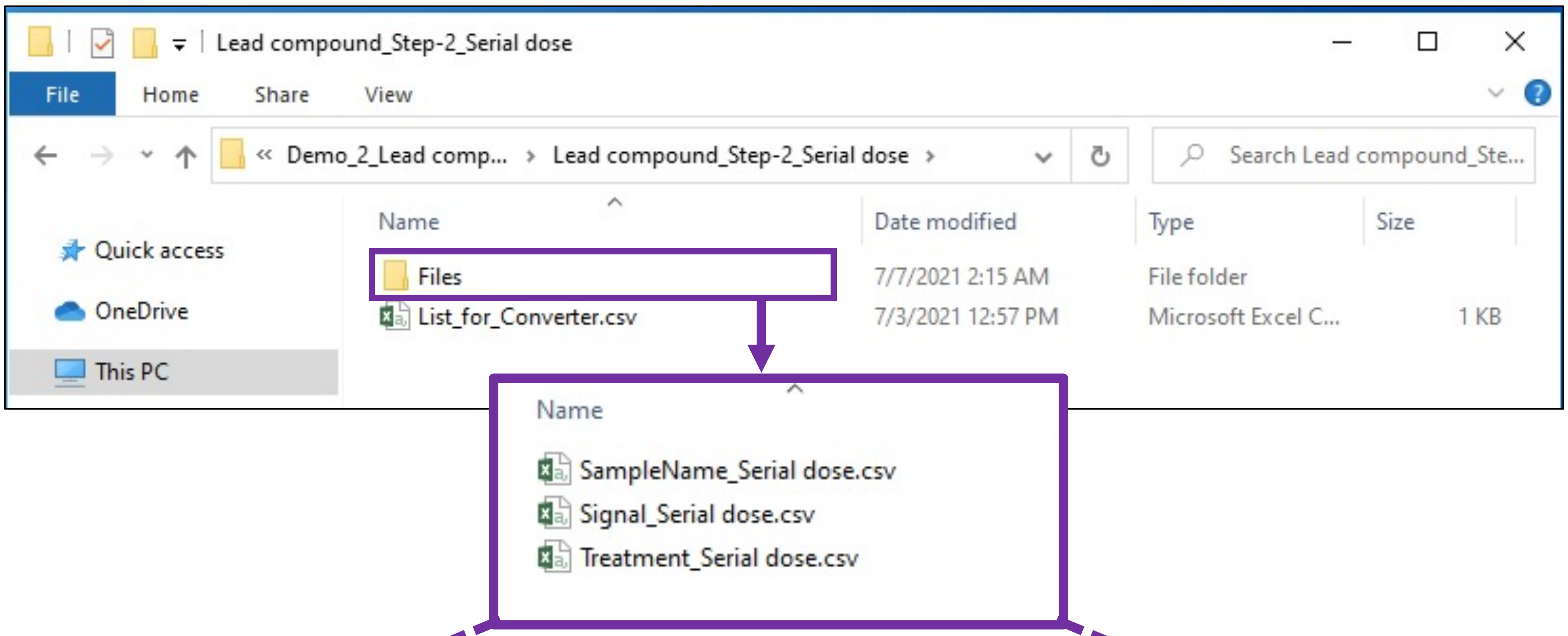

SampleName_Serial dose.csv

	A	B	C	D	E	F	G	H	I	J
1	0.1% DMSO	0.1% DMSO	0.1% DMSO	0.1% DMSO	0.1% DMSO	0.1% DMSO	0.1% DMSO	0.1% DMSO	0.1% DMSO	0.1% DMSO
2	0.9375 (micromolar)	0.9375 (micromolar)	0.9375 (micromolar)	0.9375 (micromolar)	0.9375 (micromolar)	0.9375 (micromolar)	0.9375 (micromolar)	0.9375 (micromolar)	0.9375 (micromolar)	0.9375 (micromolar)
3	1.875 (micromolar)	1.875 (micromolar)	1.875 (micromolar)	1.875 (micromolar)	1.875 (micromolar)	1.875 (micromolar)	1.875 (micromolar)	1.875 (micromolar)	1.875 (micromolar)	1.875 (micromolar)
4	3.75 (micromolar)	3.75 (micromolar)	3.75 (micromolar)	3.75 (micromolar)	3.75 (micromolar)	3.75 (micromolar)	3.75 (micromolar)	3.75 (micromolar)	3.75 (micromolar)	3.75 (micromolar)
5	7.5 (micromolar)	7.5 (micromolar)	7.5 (micromolar)	7.5 (micromolar)	7.5 (micromolar)	7.5 (micromolar)	7.5 (micromolar)	7.5 (micromolar)	7.5 (micromolar)	7.5 (micromolar)
6	15 (micromolar)	15 (micromolar)	15 (micromolar)	15 (micromolar)	15 (micromolar)	15 (micromolar)	15 (micromolar)	15 (micromolar)	15 (micromolar)	15 (micromolar)
7										

SampleName

Treatment_Serial dose.csv

	A	B	C	D	E	F	G	H	I	J	K
1	sorafenib	sorafenib	sorafenib	sorafenib	sorafenib	BIX01254	BIX01254	BIX01254	BIX01254	BIX01254	
2	sorafenib	sorafenib	sorafenib	sorafenib	sorafenib	BIX01254	BIX01254	BIX01254	BIX01254	BIX01254	
3	sorafenib	sorafenib	sorafenib	sorafenib	sorafenib	BIX01254	BIX01254	BIX01254	BIX01254	BIX01254	
4	sorafenib	sorafenib	sorafenib	sorafenib	sorafenib	BIX01254	BIX01254	BIX01254	BIX01254	BIX01254	
5	sorafenib	sorafenib	sorafenib	sorafenib	sorafenib	BIX01254	BIX01254	BIX01254	BIX01254	BIX01254	
6	sorafenib	sorafenib	sorafenib	sorafenib	sorafenib	BIX01254	BIX01254	BIX01254	BIX01254	BIX01254	
7											

Treatment

Signal_Serial dose.csv

	A	B	C	D	E	F	G	H	I	J	K	L	M	N
1		1	2	3	4	5	6	7	8	9	10	11	12	
2	A	0	0.032	0.033	0.033	0.034	0.035	0.035	0.034	0.034	0.033	0.034	-0.098	
3	B	0.03	0.153	0.135	0.129	0.111	0.129	0.118	0.124	0.128	0.13	0.11	0.033	
4	C	0.028	0.09	0.101	0.106	0.098	0.099	0.127	0.129	0.128	0.125	0.101	0.032	
5	D	0	0.087	0.094	0.092	0.082	0.097	0.127	0.135	0.127	0.127	0.093	0	
6	E	0	0.067	0.061	0.067	0.06	0.071	0.109	0.119	0.116	0.092	0.078	0.001	
7	F	0	0.038	0.032	0.036	0.036	0.031	0.079	0.071	0.068	0.07	0.053	0	
8	G	0.001	0.001	0.008	0.009	0.009	0.012	0.03	0.027	0.025	0.023	0.023	0	
9	H	0	0.033	0.033	0.033	0.033	0.03[illegible]	0.034	0.034	0.033	0.032	0.034	-0.001	
10														

Signal

List_for_Converter.csv”

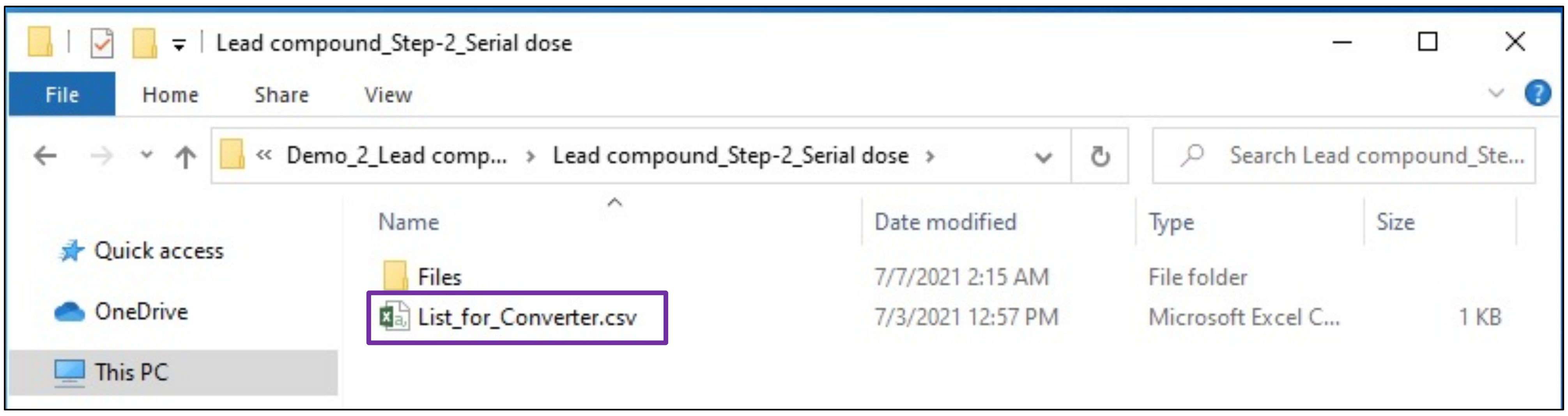

- The data in the first row of “Converted_data.csv” file (the result file output from GM_Converter) is from the first row of “List_for_Converter” file (circled by red frame). Please see the Figure A1-A11 in Additional file 2 for the detailed concept of GM_Converter.
- The data of files listed in the same row of “List_for_Converter.csv” (circled by blue frame) will be integrated. Please see the Figure A1-A11 in Additional file 2 for the detailed concept of GM_Converter.

“Converted_data.csv”

First row →

	A	B	C
1	SampleName_Dosage	Treatment_Compound name	Signal
2	0.1% DMSO	sorafenib	0.153
3	0.1% DMSO	sorafenib	0.135
4	0.1% DMSO	sorafenib	0.129
5	0.1% DMSO	sorafenib	0.111
6	0.1% DMSO	sorafenib	0.129
7	0.1% DMSO	BIX01254	0.118

“List_for_Converter.csv” file

	A	B	C	D
1	SampleName_Dosage	Treatment_Compound name	Signal	
2	SampleName_Serial dose.csv	Treatment_Serial dose.csv	Signal_Serial dose.csv	
3				

- Operation steps

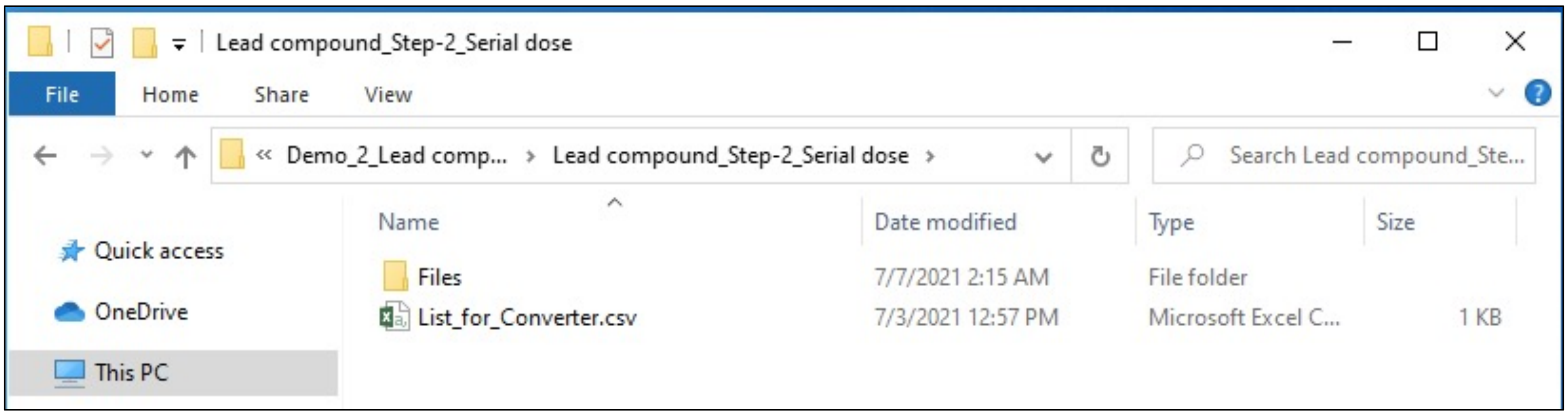

Copy and paste the two executive files of GM_Converter (GateMultiplex_converter.exe and Converter.exe) into "Lead compound_Step-2_Serial dose" folder (purple frames and purple arrows)

*The two executive files should be placed in the same folder

- The "List_for_Converter.csv" (indicated by the green arrow) should be placed in the same folder with the two executive files of GM_Converter (GateMultiplex_converter.exe and Converter.exe).

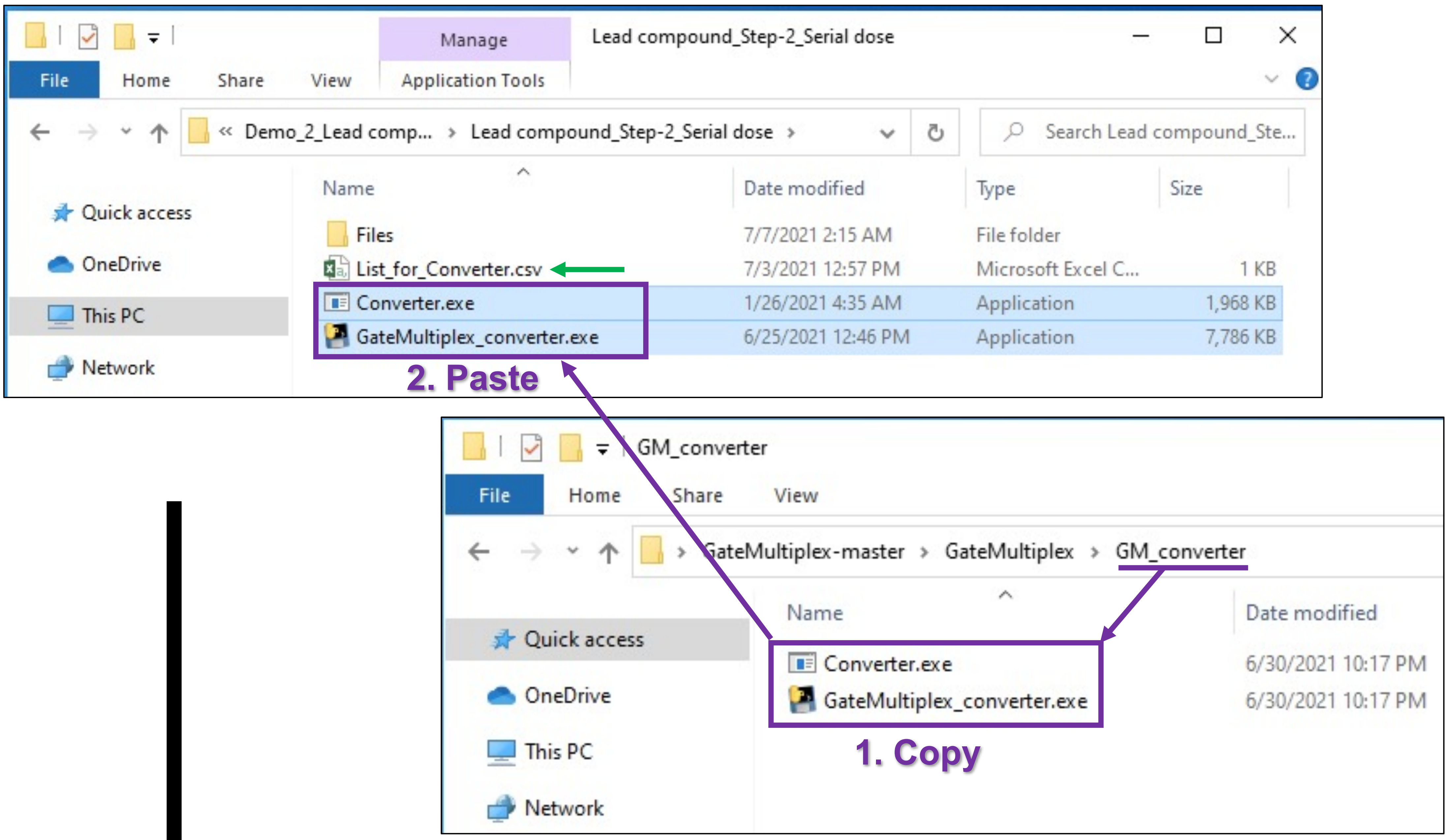

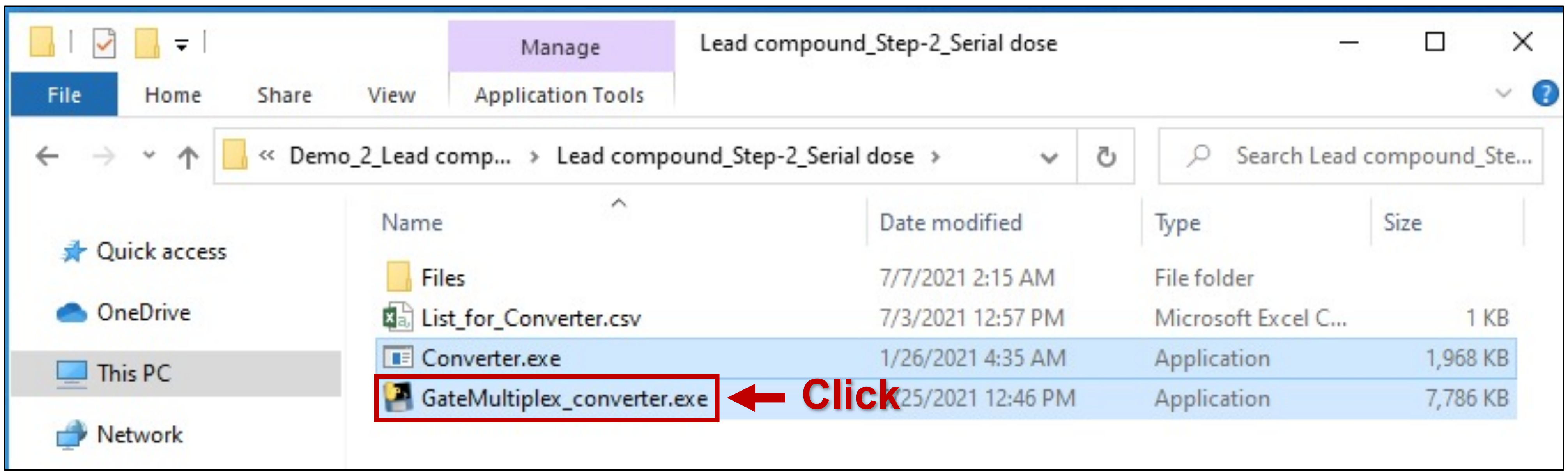

Activate the GM_Converter by a double-clicking on "GateMultiplex_converter.exe" (red frame)

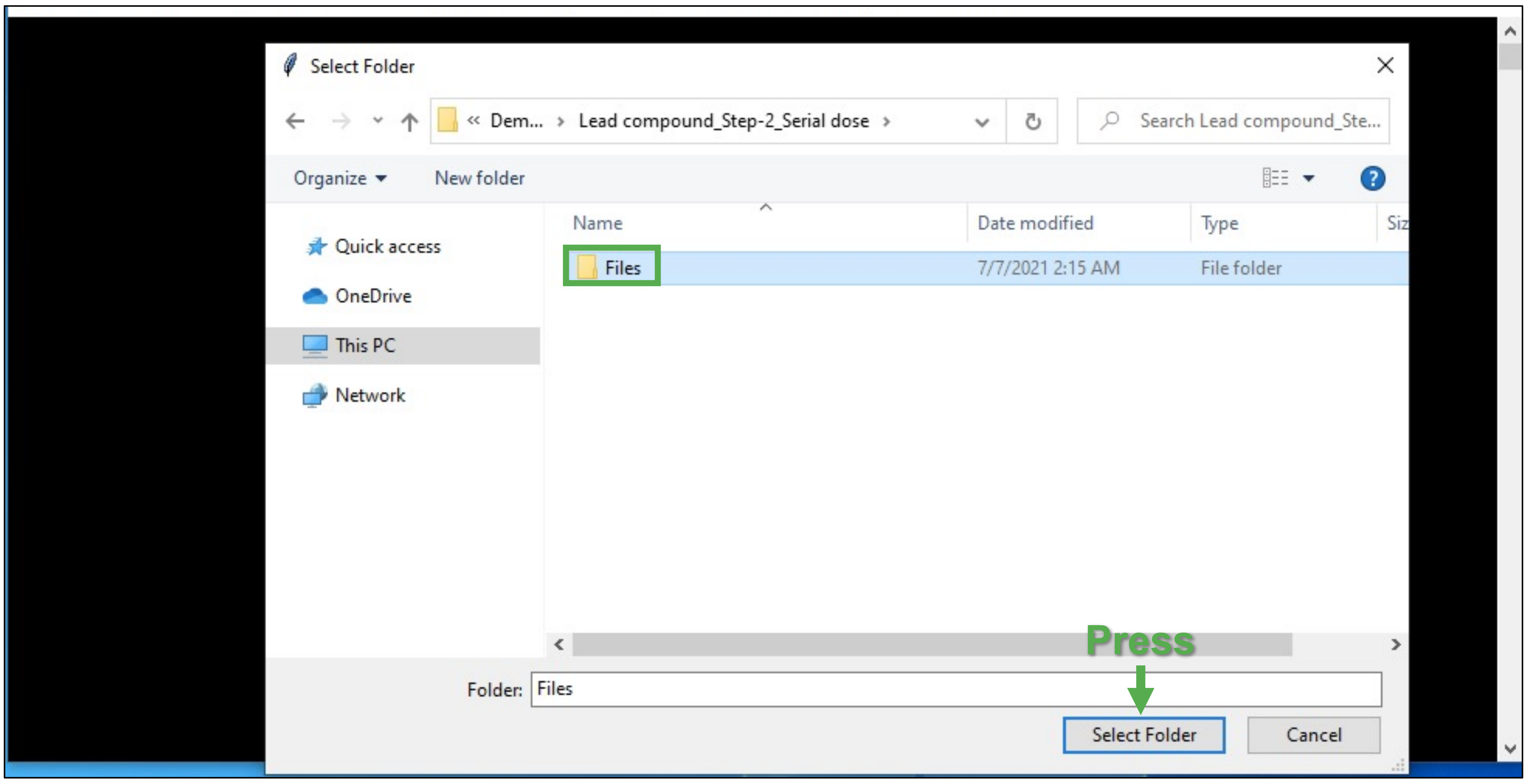

Select the folder "Files" (Directory: Additional file 5 > Demo_2_Lead compound > Lead compound_Step-2_Serial dose > Files) (green frame) and press "Select Folder" (indicated by a green arrow)

- Please see the Figure A5-A11 in Additional file 2 for the detailed parameter setting of GM_Converter.

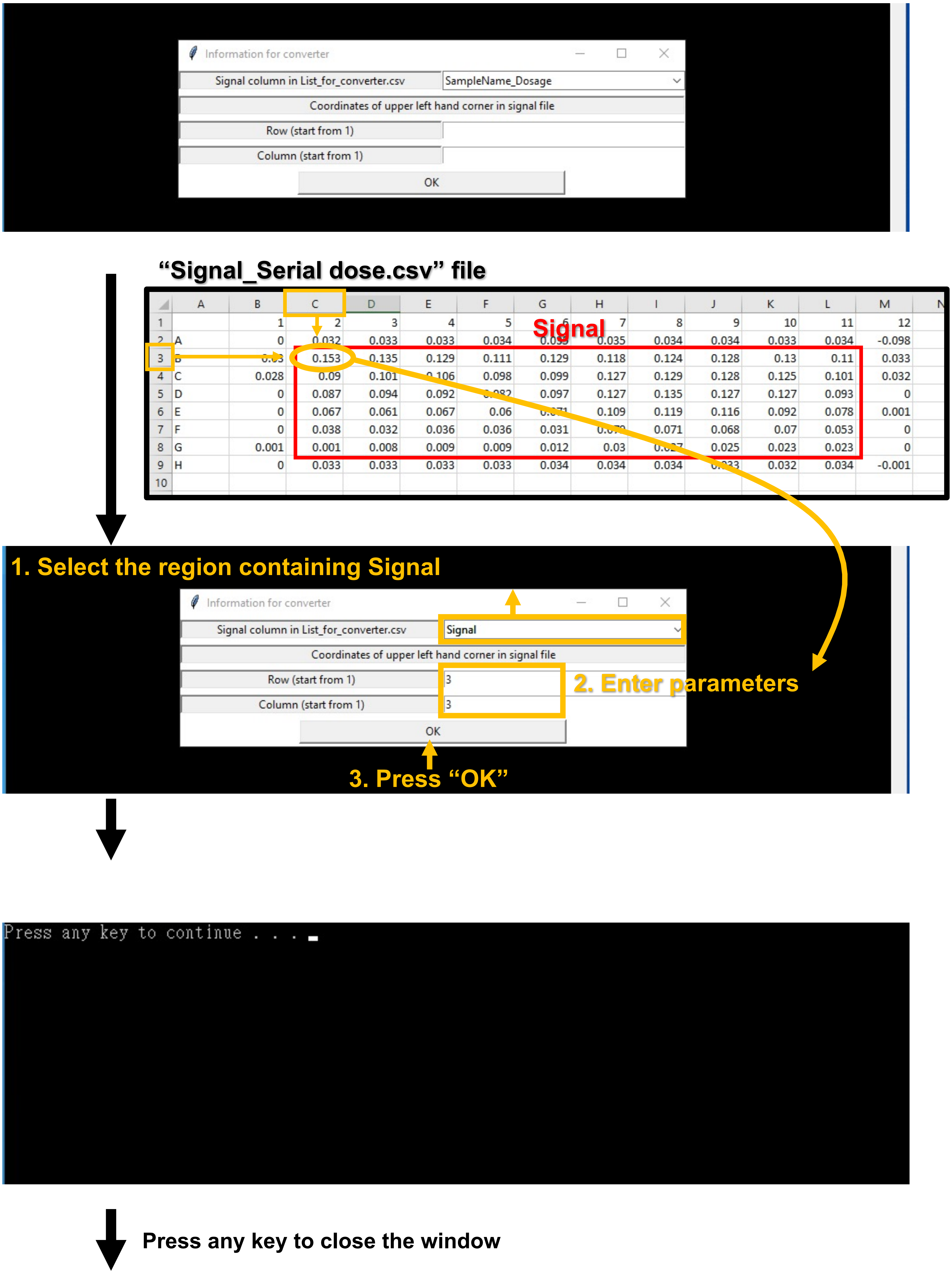

- The converted data “Converted_data.csv” (green frame) is stored in the “GM_converted_data” folder (red frame).

- The title of “Converted_data.csv” (circled by blue frame) is the same as the title in “List_for_Converter.csv”. Please see Figure A5-A11 in Additional file 2.

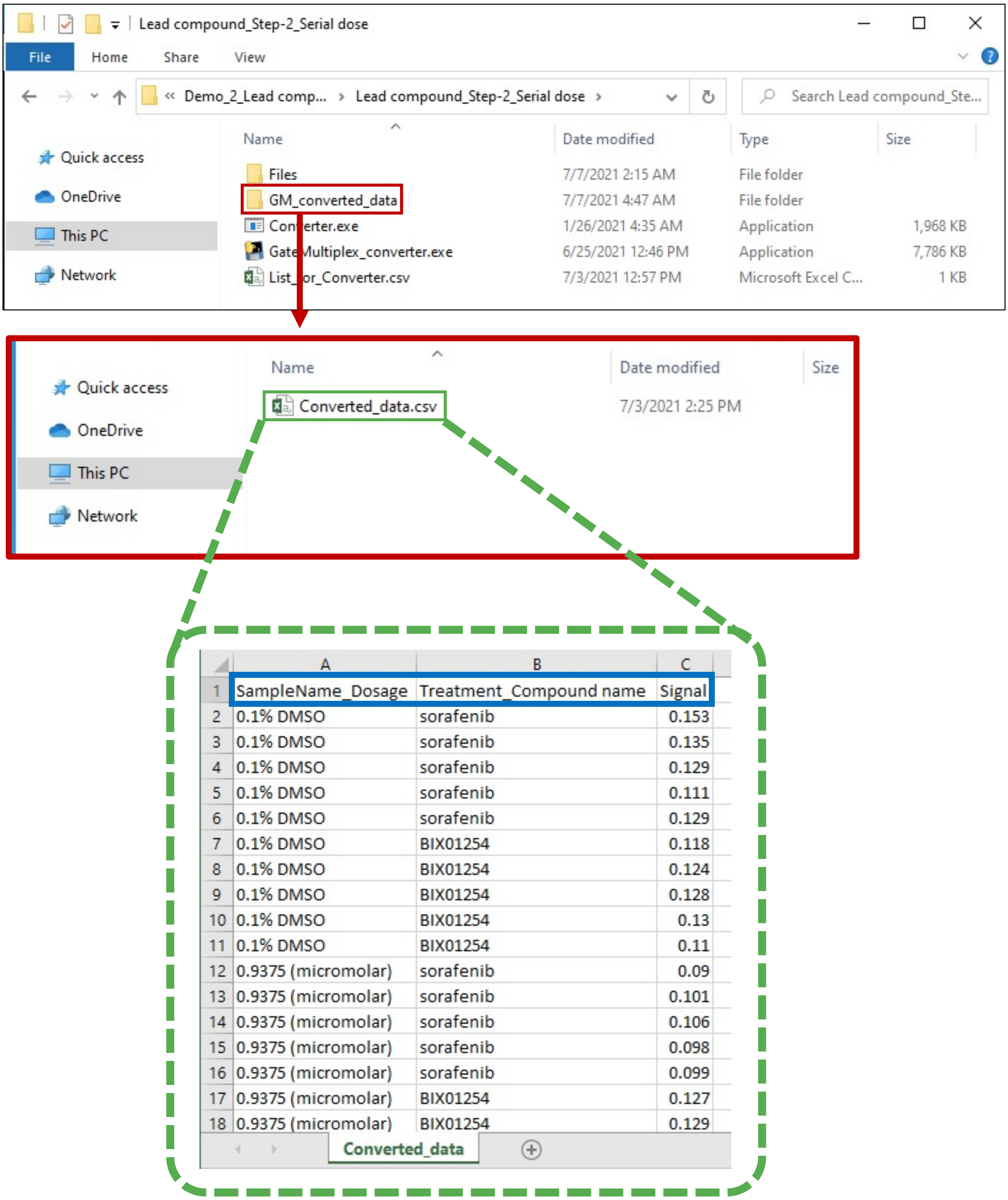

❖ Serial dose (GM_Basic)

- After Converted_data.csv completing, GM_Basic is further applied for analysis.
- The two executive files of GM_Basic (GateMultiplex_basic.exe and GateMultiplex_forWindows.exe) are both stored in the "GM_basic" folder (blue frame) and should be both placed in the same folder for operation.
- Please see Fig. S10 and 11 in the Additional file 1.

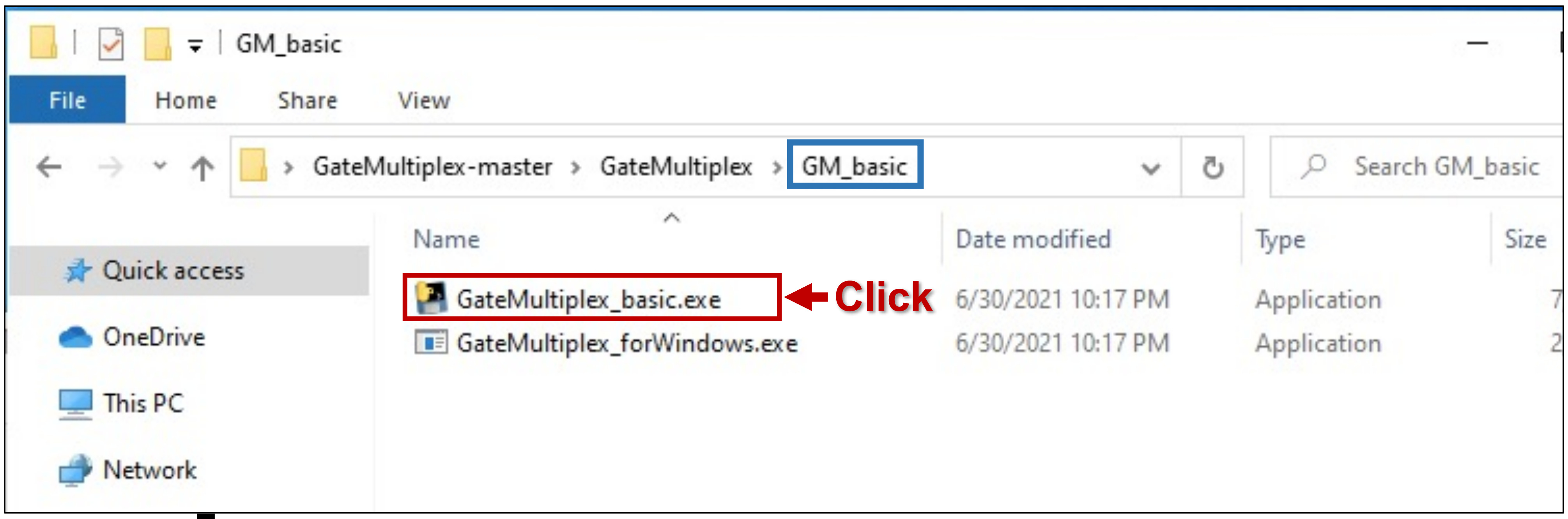

Activate the GM_basic by a double-clicking on "GateMultiplex_basic.exe" (red frame)

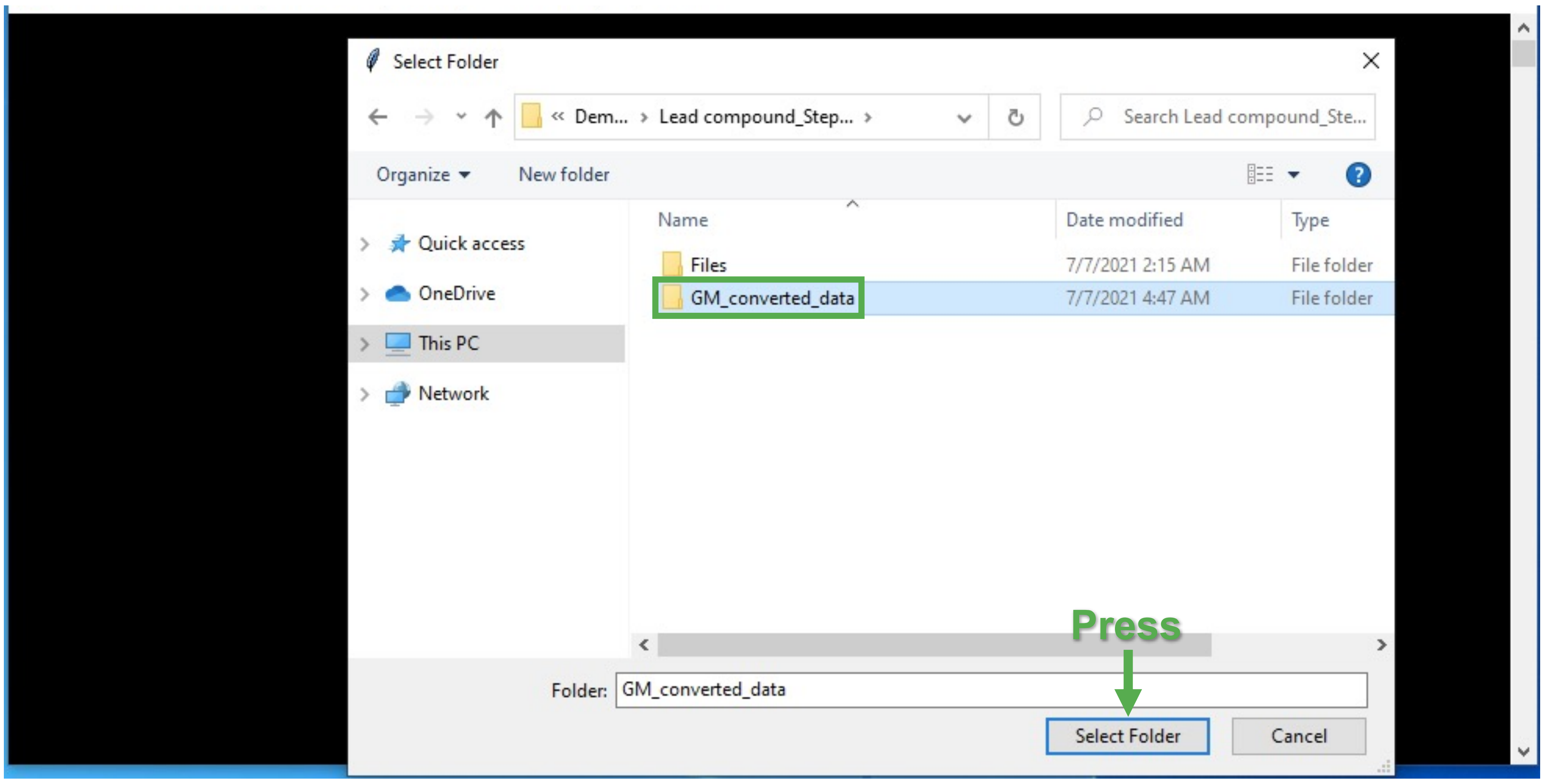

Select the folder "GM_converted_data" (Directory: Additional file 5 > Demo_2_Lead compound > Lead compound_Step-2_Serial dose > GM_converted_data) (green frame) and press "Select Folder" (indicated by a green arrow)

✱ The GUI of GM_Basic

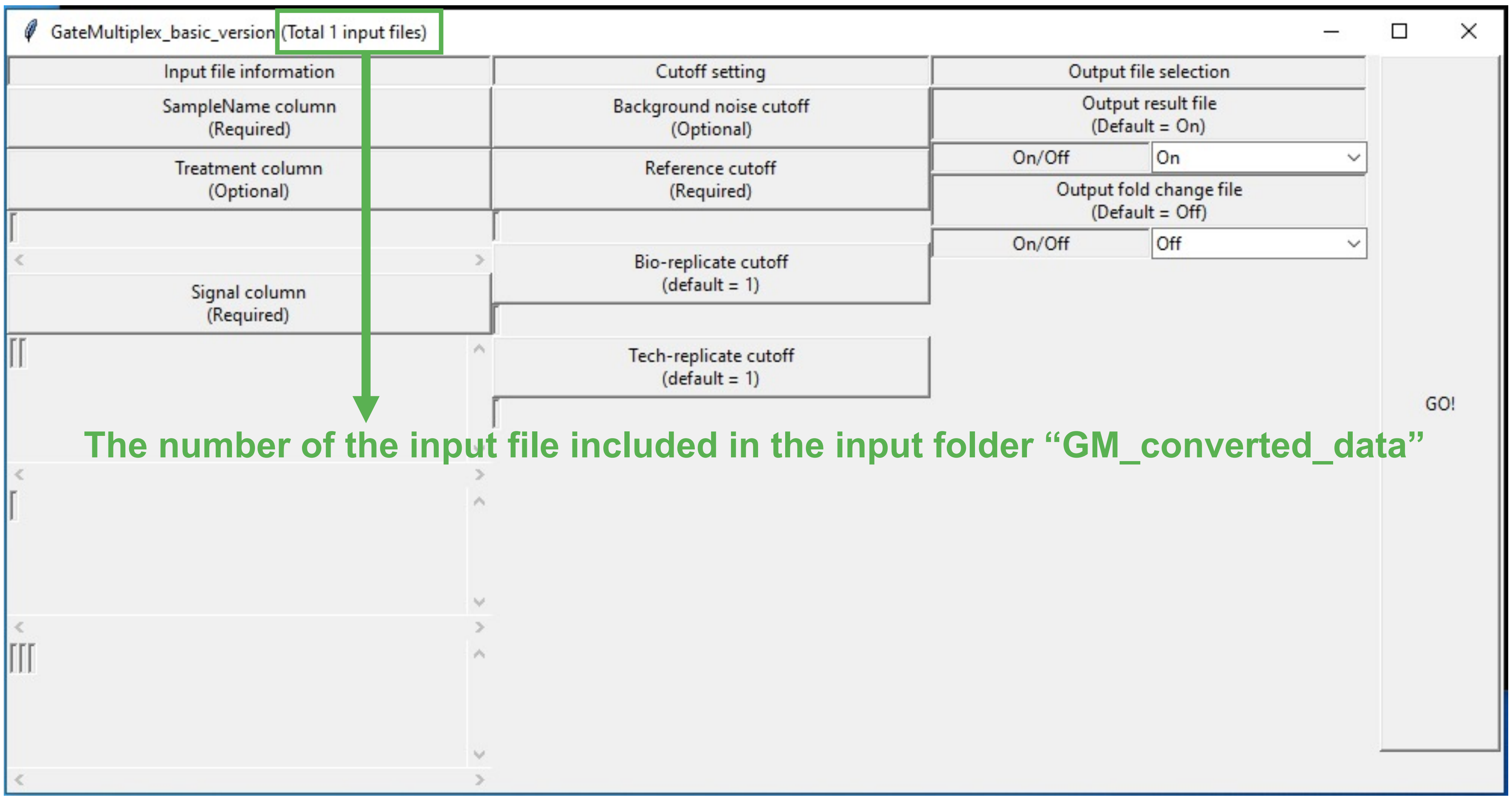

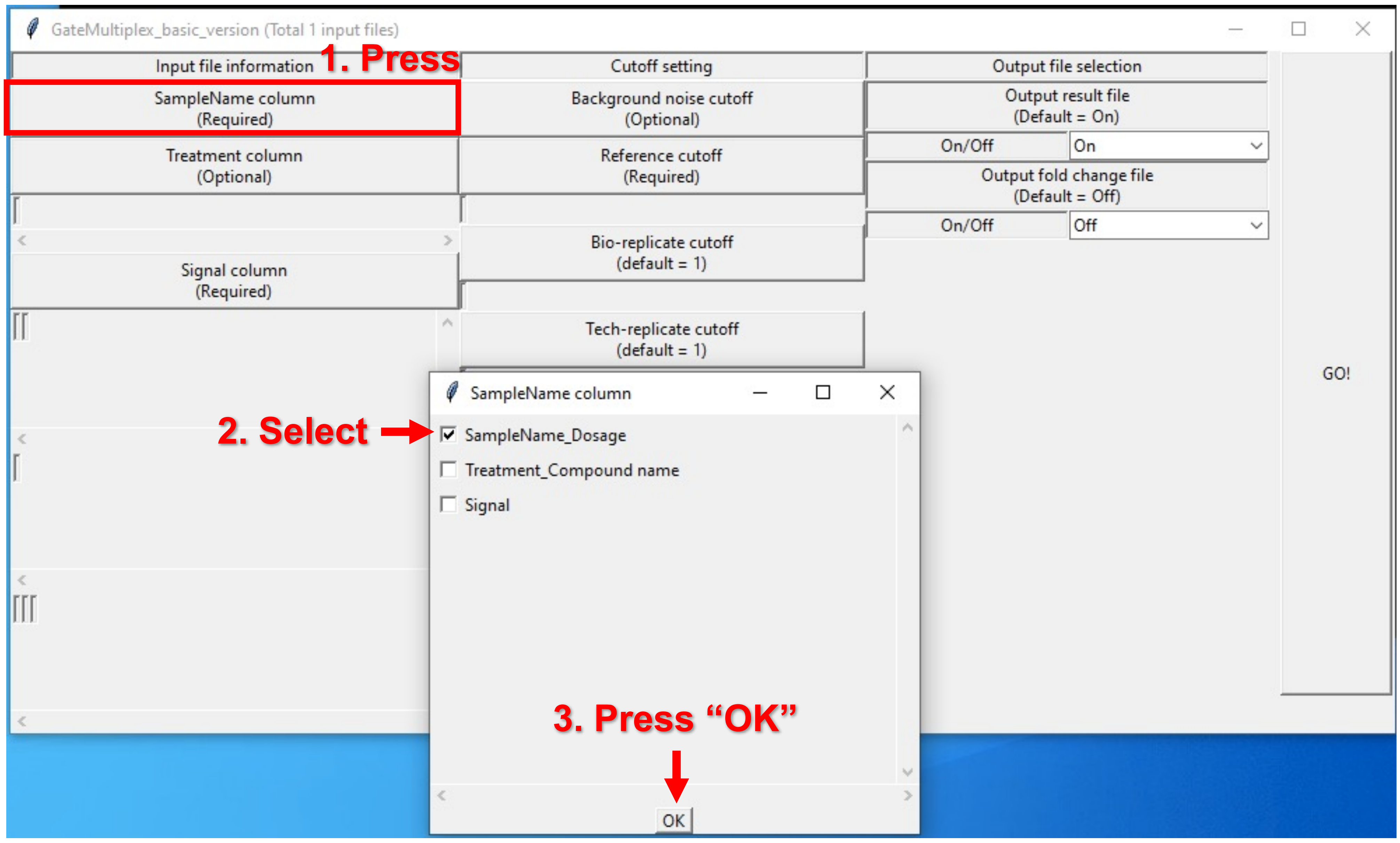

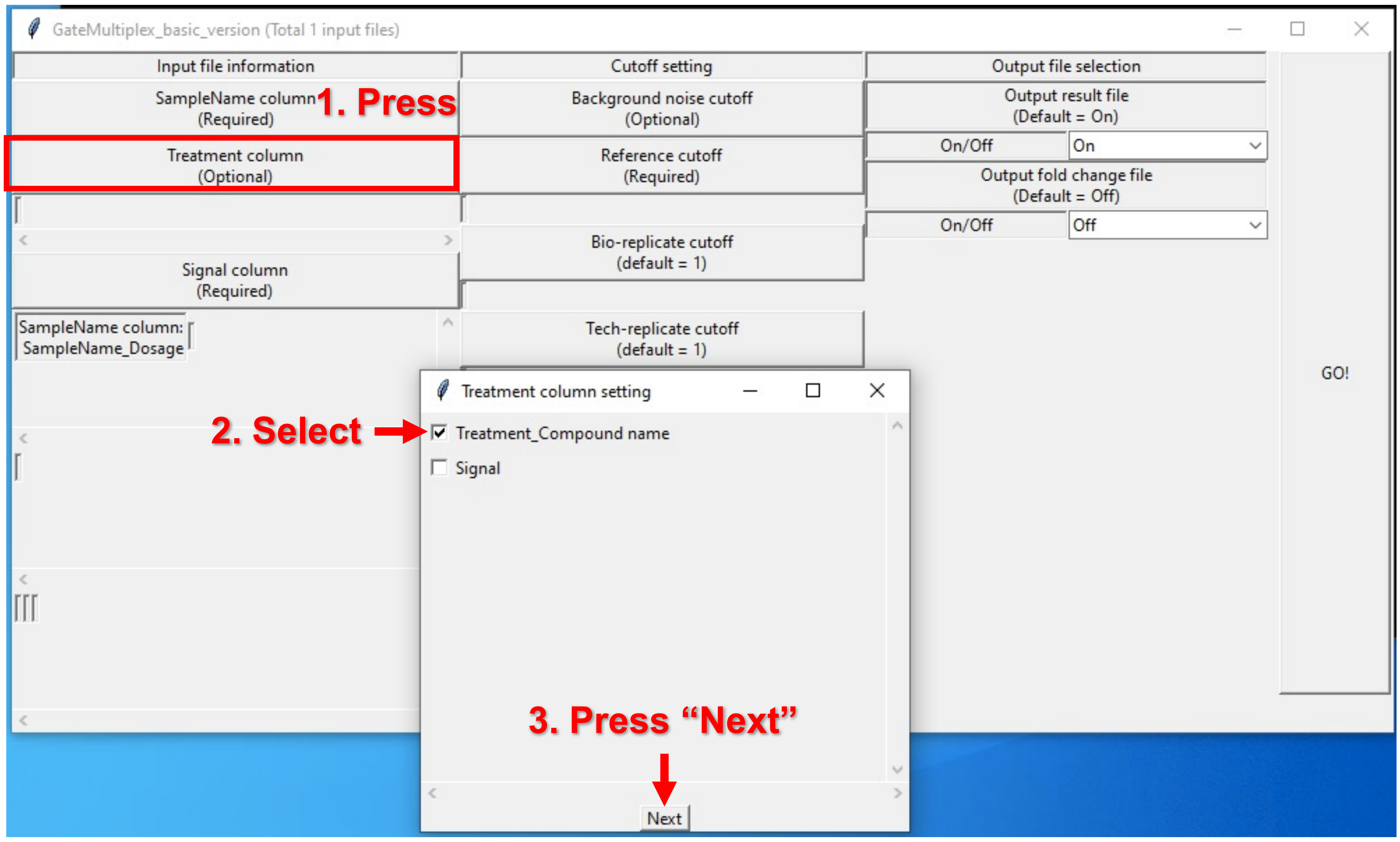
GateMultiplex_basic_version (Total 1 input files)
Input file information
SampleName column (Required)
1. Press
Treatment column (Optional)
Signal column (Required)
SampleName column: SampleName_Dosage
Cutoff setting
Background noise cutoff (Optional)
Reference cutoff (Required)
Bio-replicate cutoff (default = 1)
Tech-replicate cutoff (default = 1)
Output file selection
Output result file (Default = On)
On/Off
On
Output fold change file (Default = Off)
On/Off
Off
GO!
Treatment column setting
2. Select
Treatment_Compound name
Signal
3. Press "Next"
Next

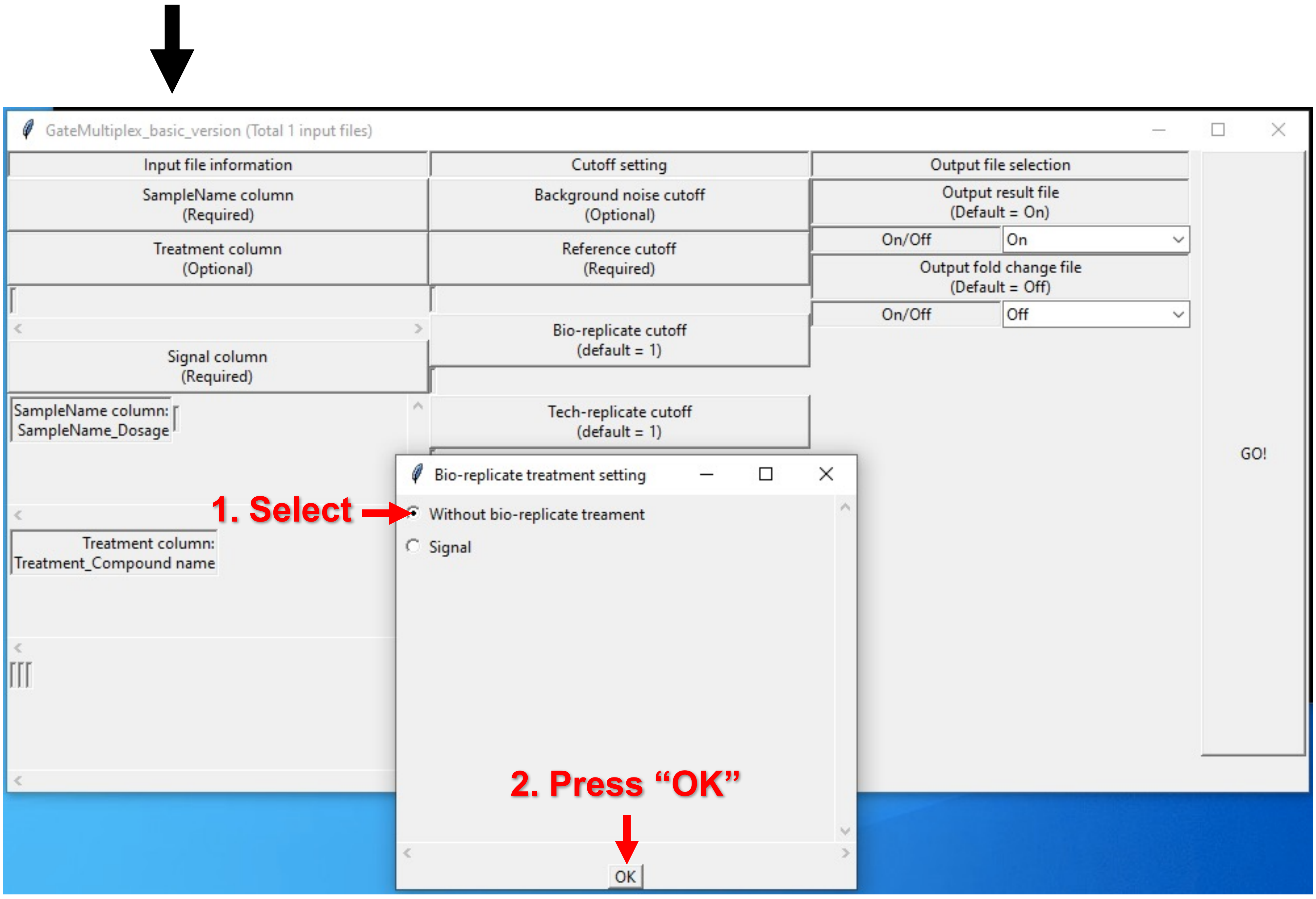
GateMultiplex_basic_version (Total 1 input files)
Input file information
SampleName column (Required)
Treatment column (Optional)
Signal column (Required)
SampleName column: SampleName_Dosage
Treatment column: Treatment_Compound name
Cutoff setting
Background noise cutoff (Optional)
Reference cutoff (Required)
Bio-replicate cutoff (default = 1)
Tech-replicate cutoff (default = 1)
Output file selection
Output result file (Default = On)
On/Off
On
Output fold change file (Default = Off)
On/Off
Off
GO!
Bio-replicate treatment setting
1. Select
Without bio-replicate treament
Signal
2. Press "OK"
OK

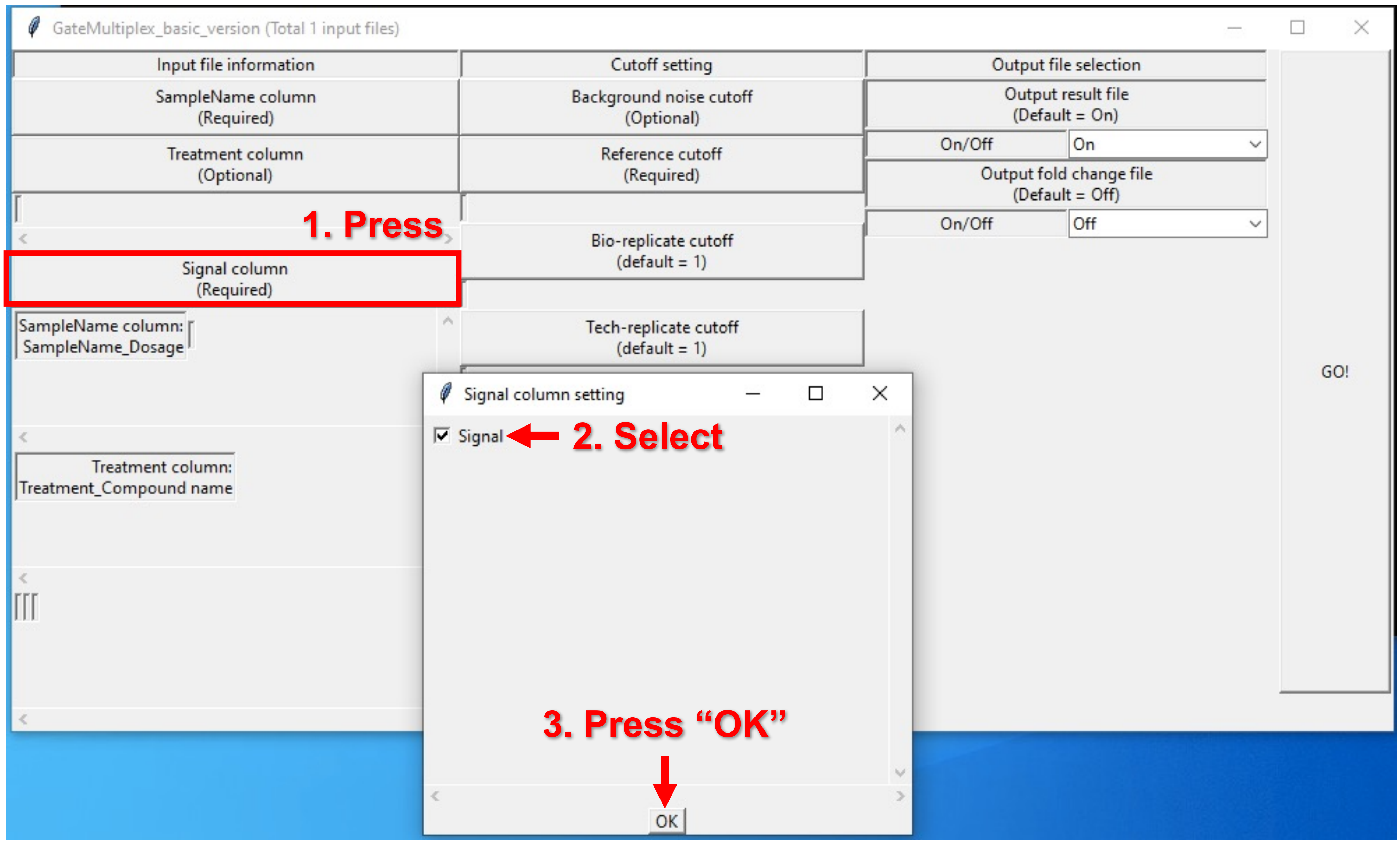

GateMultiplex_basic_version (Total 1 input files)
Input file information
SampleName column (Required)
Treatment column (Optional)
1. Press
Signal column (Required)
SampleName column: SampleName_Dosage
Treatment column: Treatment_Compound name
Cutoff setting
Background noise cutoff (Optional)
Reference cutoff (Required)
Bio-replicate cutoff (default = 1)
Tech-replicate cutoff (default = 1)
Output file selection
Output result file (Default = On)
On/Off
On
Output fold change file (Default = Off)
On/Off
Off
GO!
Signal column setting
Signal
2. Select
3. Press "OK"
OK

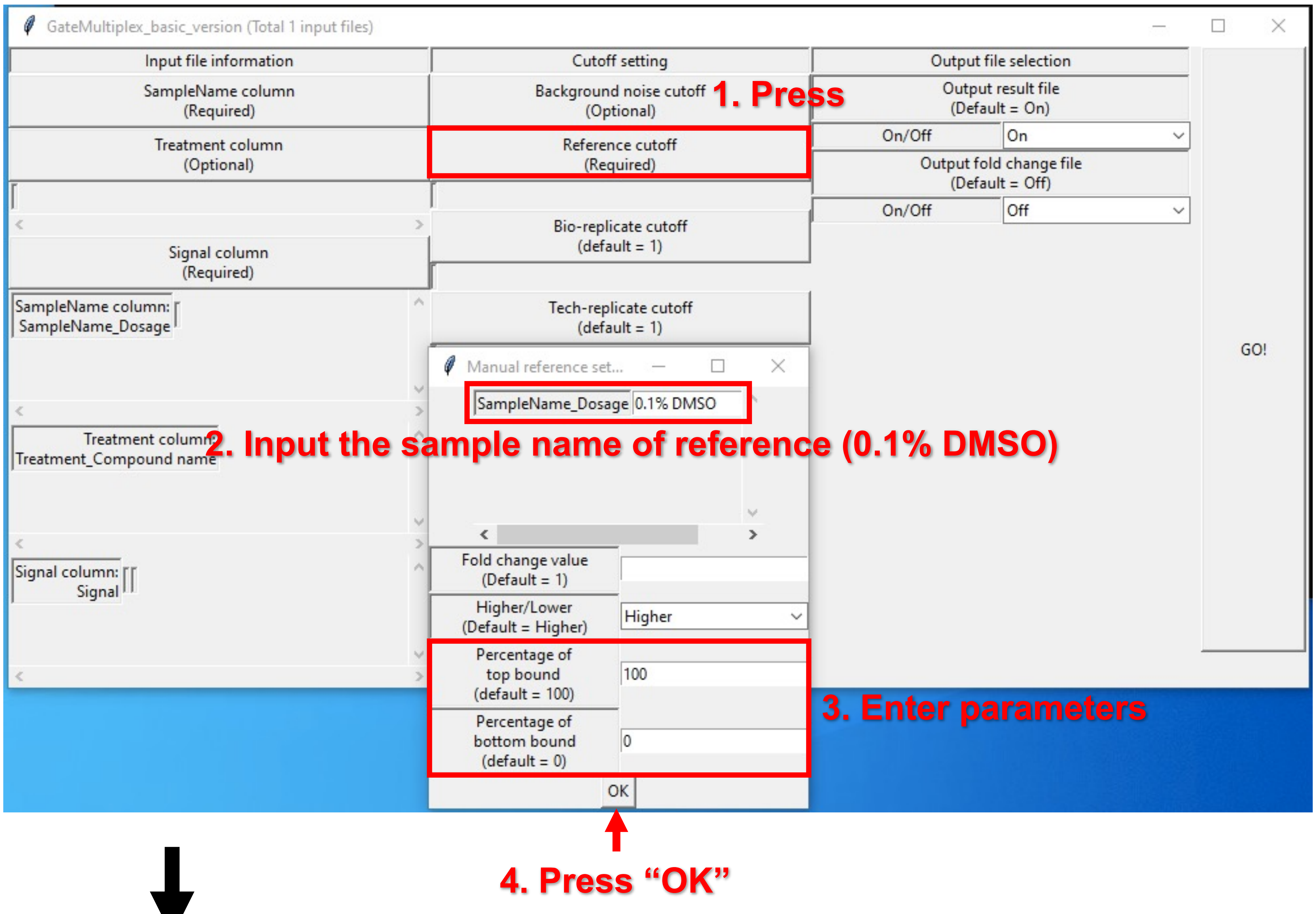

GateMultiplex_basic_version (Total 1 input files)
Input file information
SampleName column (Required)
Treatment column (Optional)
Signal column (Required)
SampleName column: SampleName_Dosage
Treatment column: Treatment_Compound name
Signal column: Signal
Cutoff setting
Background noise cutoff (Optional)
1. Press
Reference cutoff (Required)
Bio-replicate cutoff (default = 1)
Tech-replicate cutoff (default = 1)
Output file selection
Output result file (Default = On)
On/Off
On
Output fold change file (Default = Off)
On/Off
Off
GO!
Manual reference set...
SampleName_Dosage
0.1% DMSO
2. Input the sample name of reference (0.1% DMSO)
Fold change value (Default = 1)
Higher/Lower (Default = Higher)
Higher
Percentage of top bound (default = 100)
100
Percentage of bottom bound (default = 0)
0
3. Enter parameters
OK
4. Press "OK"

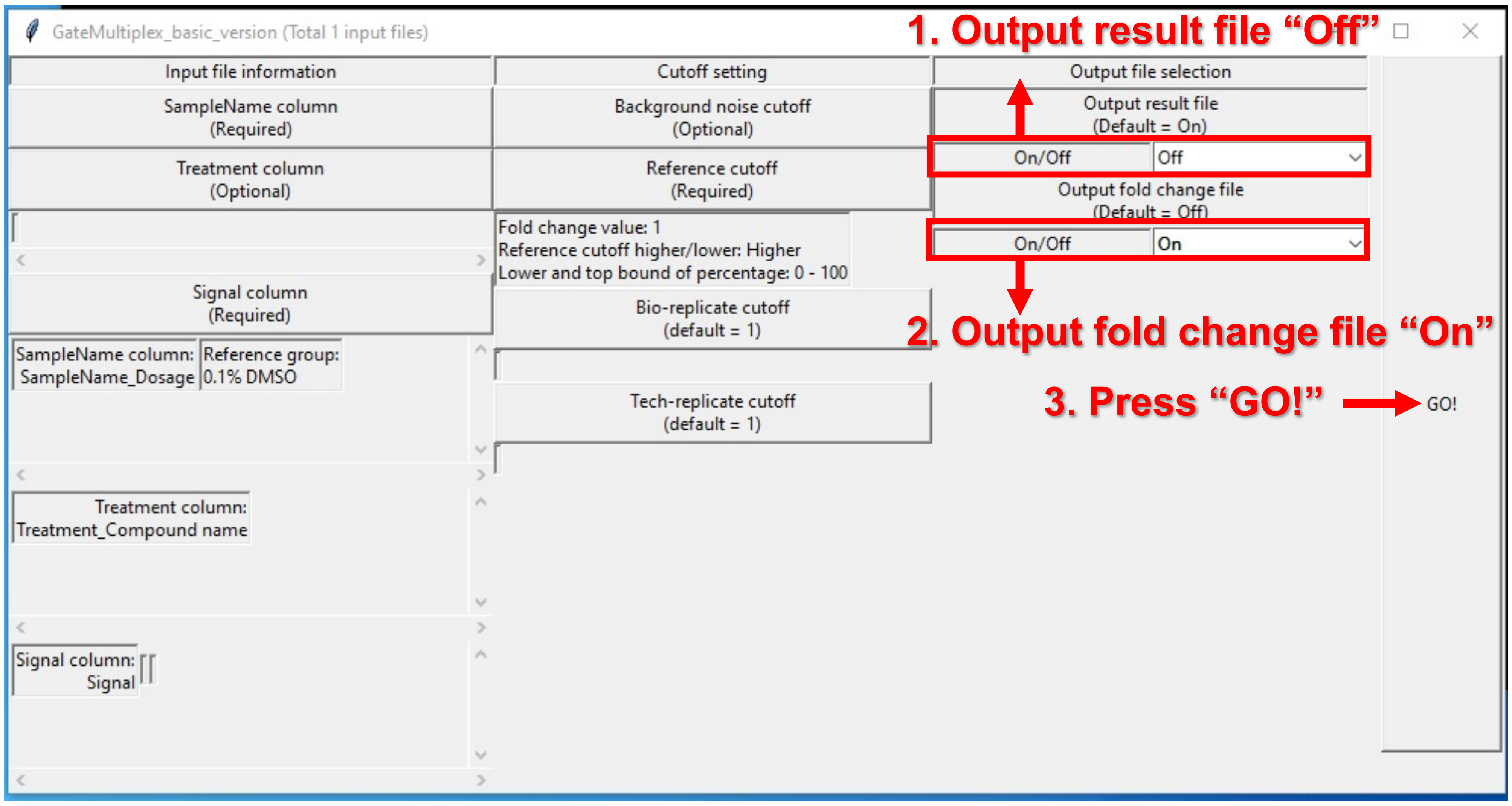

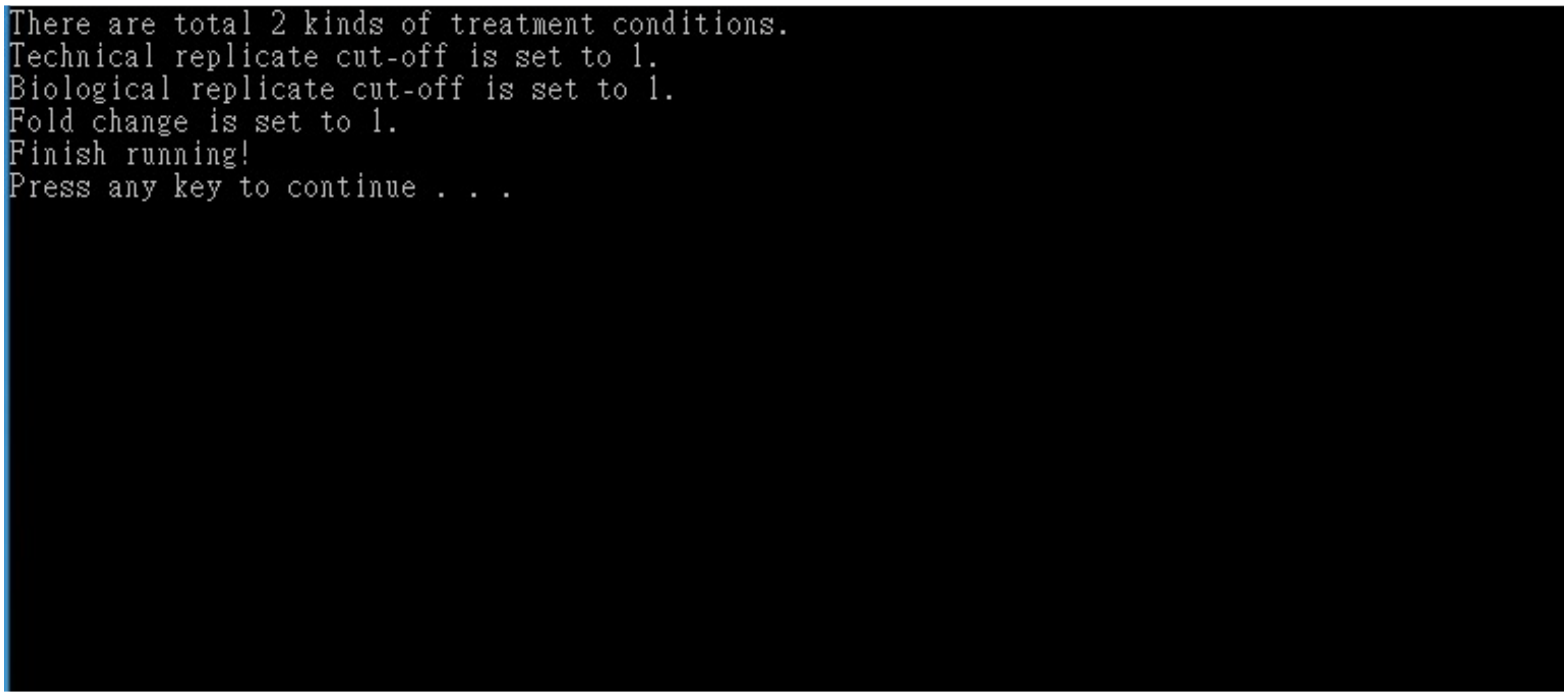

Press any key to close the window

- The fold change files are stored in the folder named “GM_outputfiles” (in green frame).
- Please see the Figure L1-L3 in Additional file 2 for the concept of mutiple fold change files.
- Please see the “Symbols in output files” section in Additional file 3 for the concept of symbols in fold change files

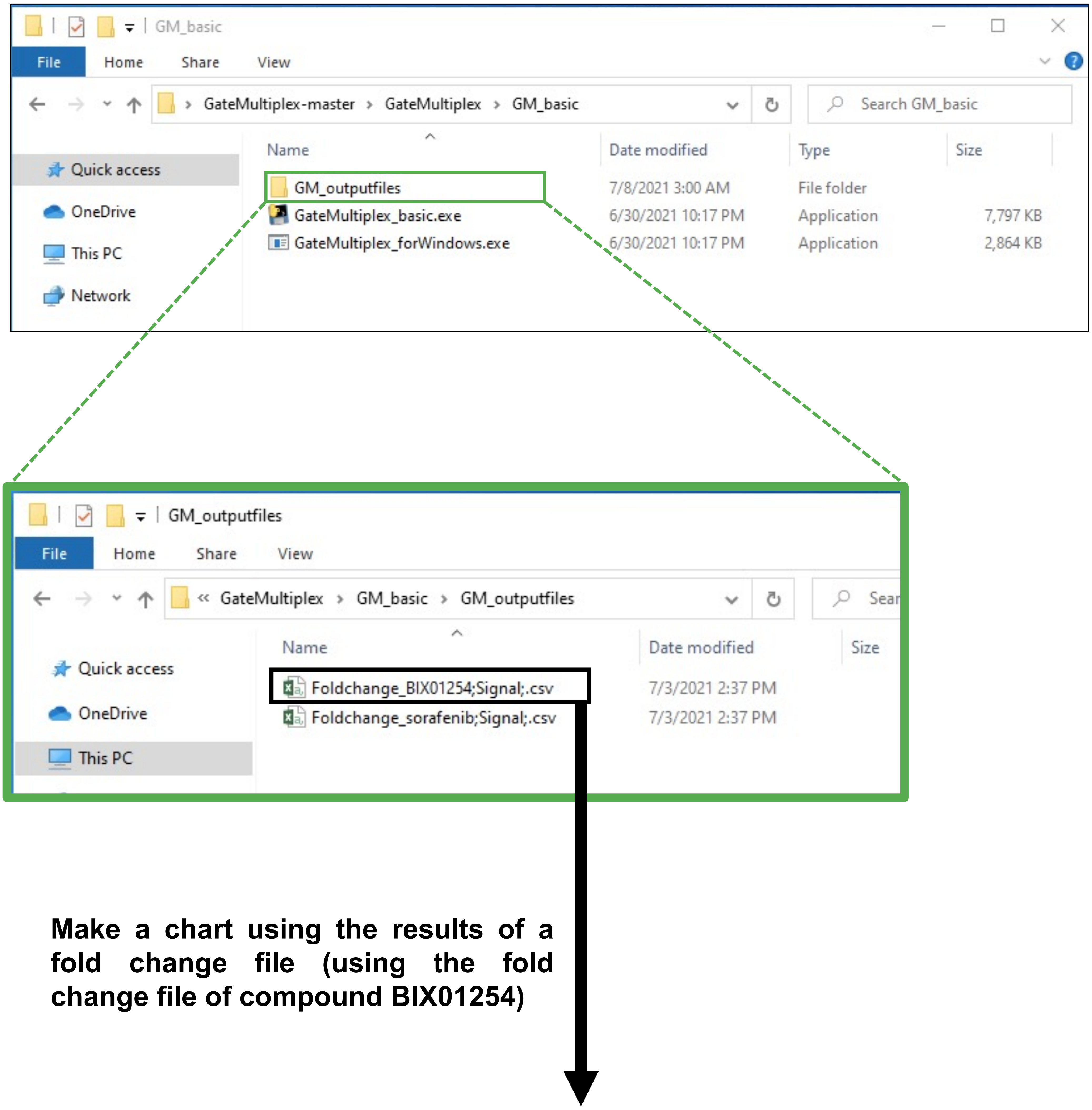

Make a chart using the results of a fold change file (using the fold change file of compound BIX01254)

“Foldchange_BIX01254; Signal;.csv” file

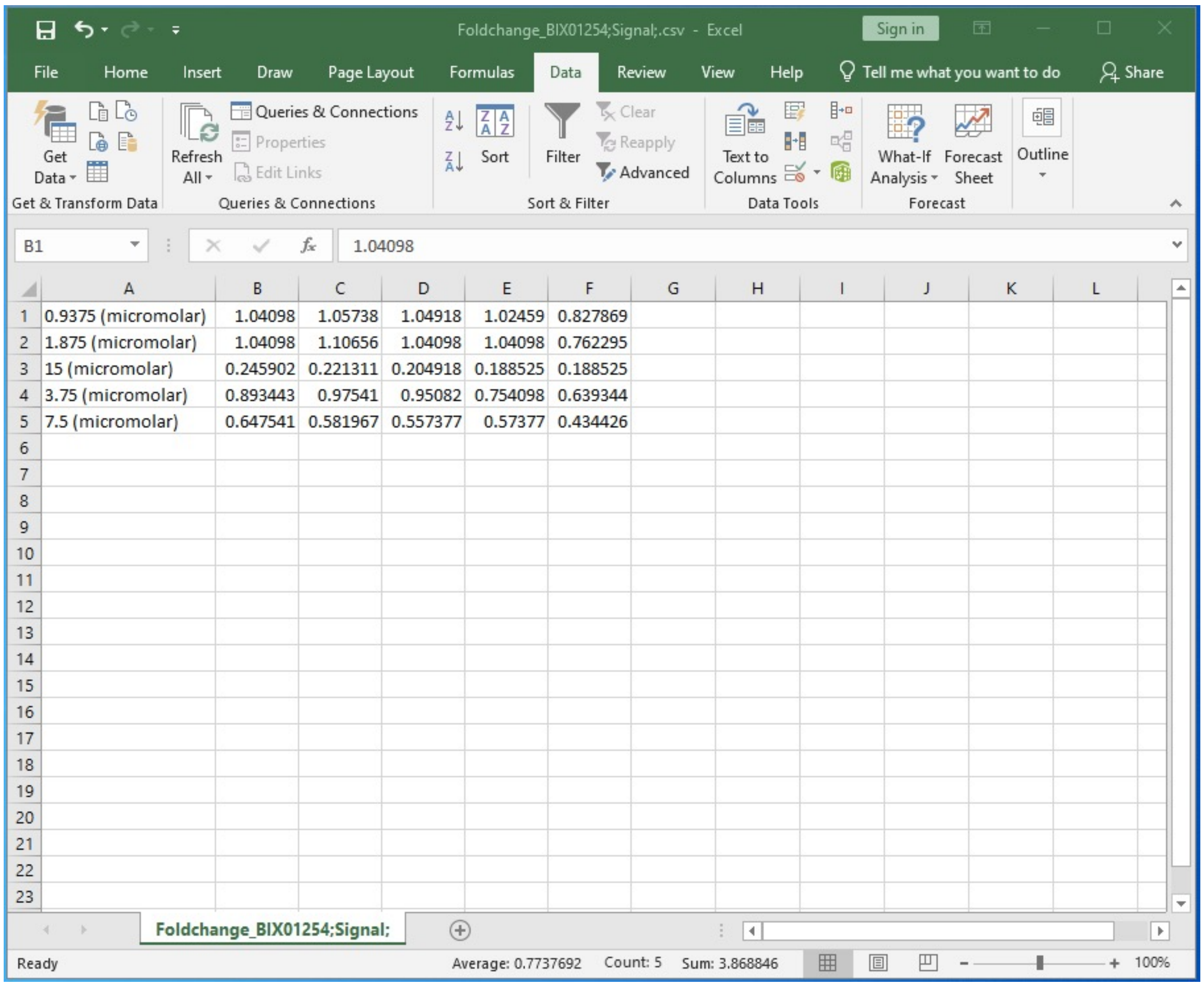

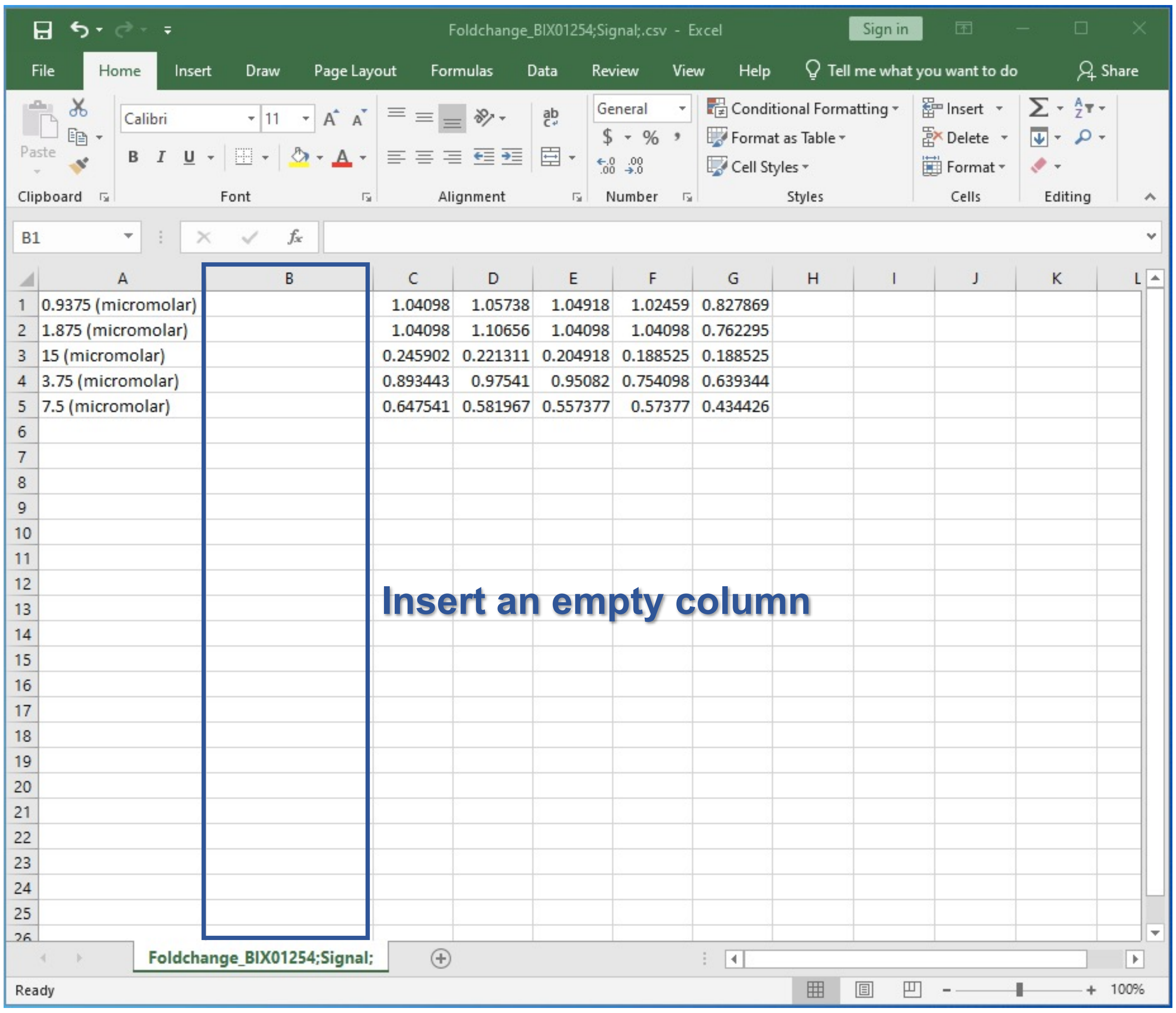

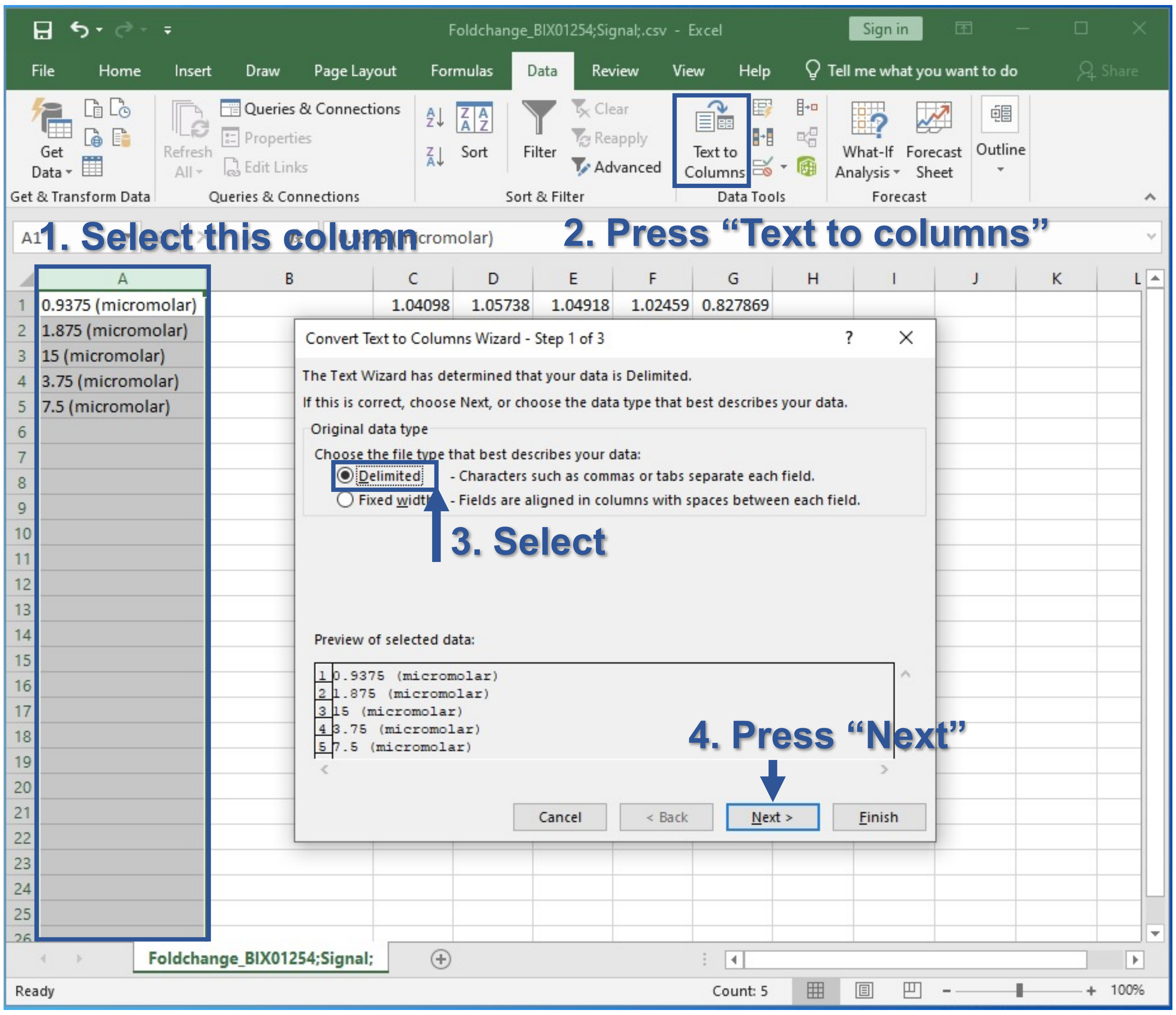
1. Select this column
2. Press "Text to columns"
Convert Text to Columns Wizard - Step 1 of 3
The Text Wizard has determined that your data is Delimited.
If this is correct, choose Next, or choose the data type that best describes your data.
Original data type
Choose the file type that best describes your data:
Delimited - Characters such as commas or tabs separate each field.
Fixed width - Fields are aligned in columns with spaces between each field.
3. Select
Preview of selected data:
4. Press "Next"
Cancel
< Back
Next >
Finish

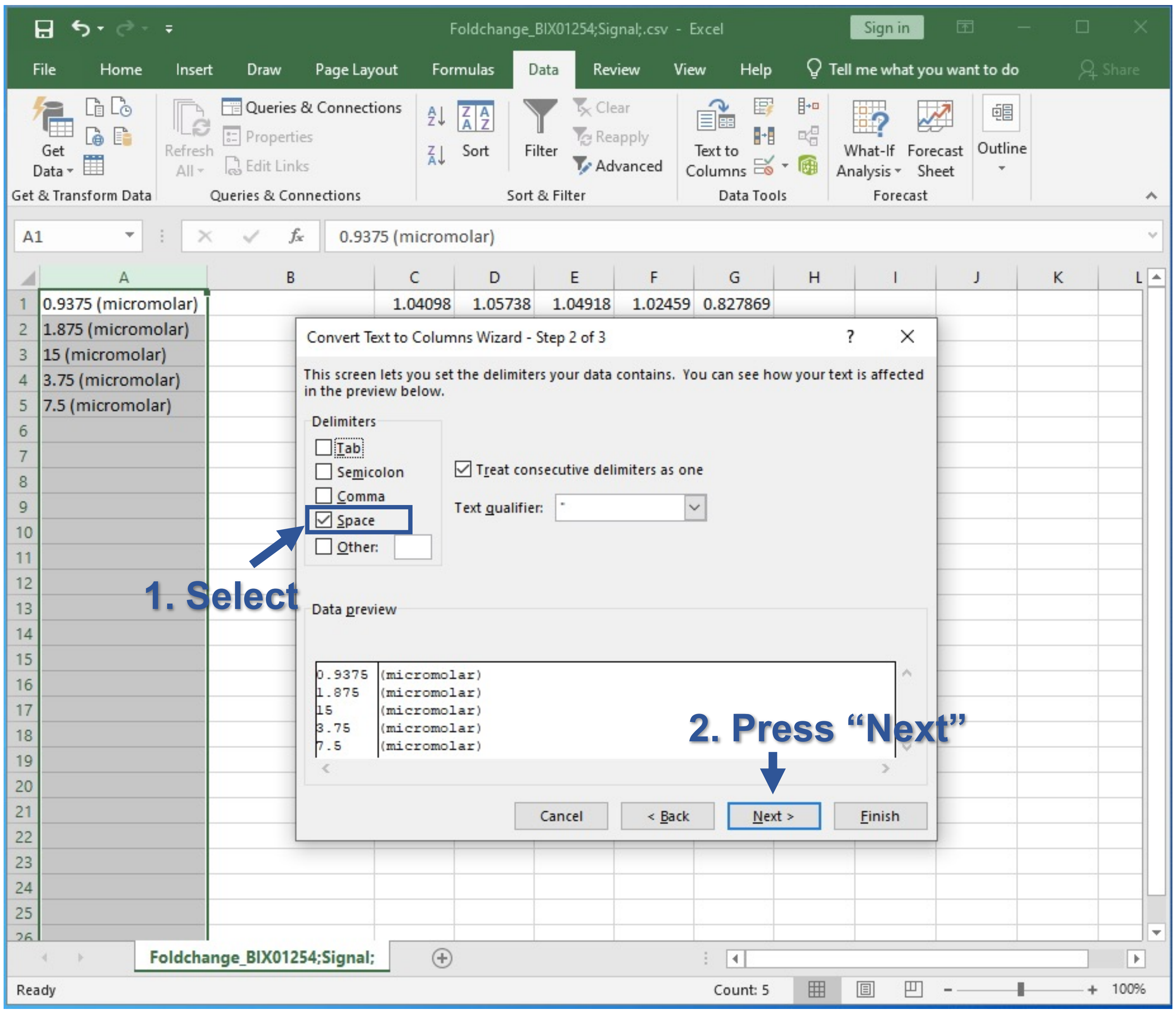
Convert Text to Columns Wizard - Step 2 of 3
This screen lets you set the delimiters your data contains. You can see how your text is affected in the preview below.
Delimiters
Tab
Semicolon
Comma
Space
Other:
Treat consecutive delimiters as one
Text qualifier:
1. Select
Data preview
2. Press "Next"
Cancel
< Back
Next >
Finish

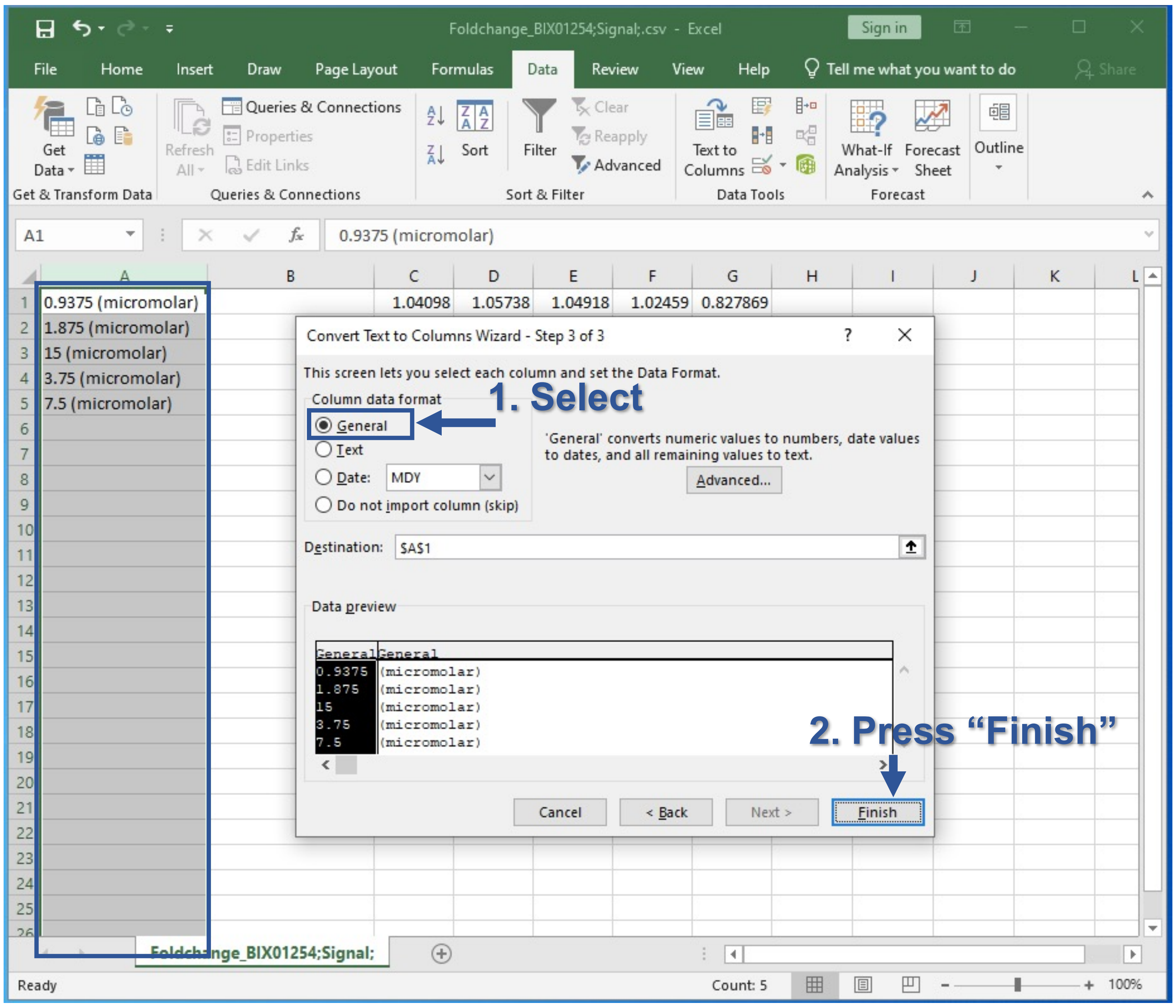
Foldchange_BIX01254;Signal;.csv - Excel
Convert Text to Columns Wizard - Step 3 of 3
This screen lets you select each column and set the Data Format.
Column data format
General
Text
Date: MDY
Do not import column (skip)
'General' converts numeric values to numbers, date values to dates, and all remaining values to text.
Advanced...
Destination: A1
Data preview
Cancel
< Back
Next >
Finish
1. Select
2. Press “Finish”

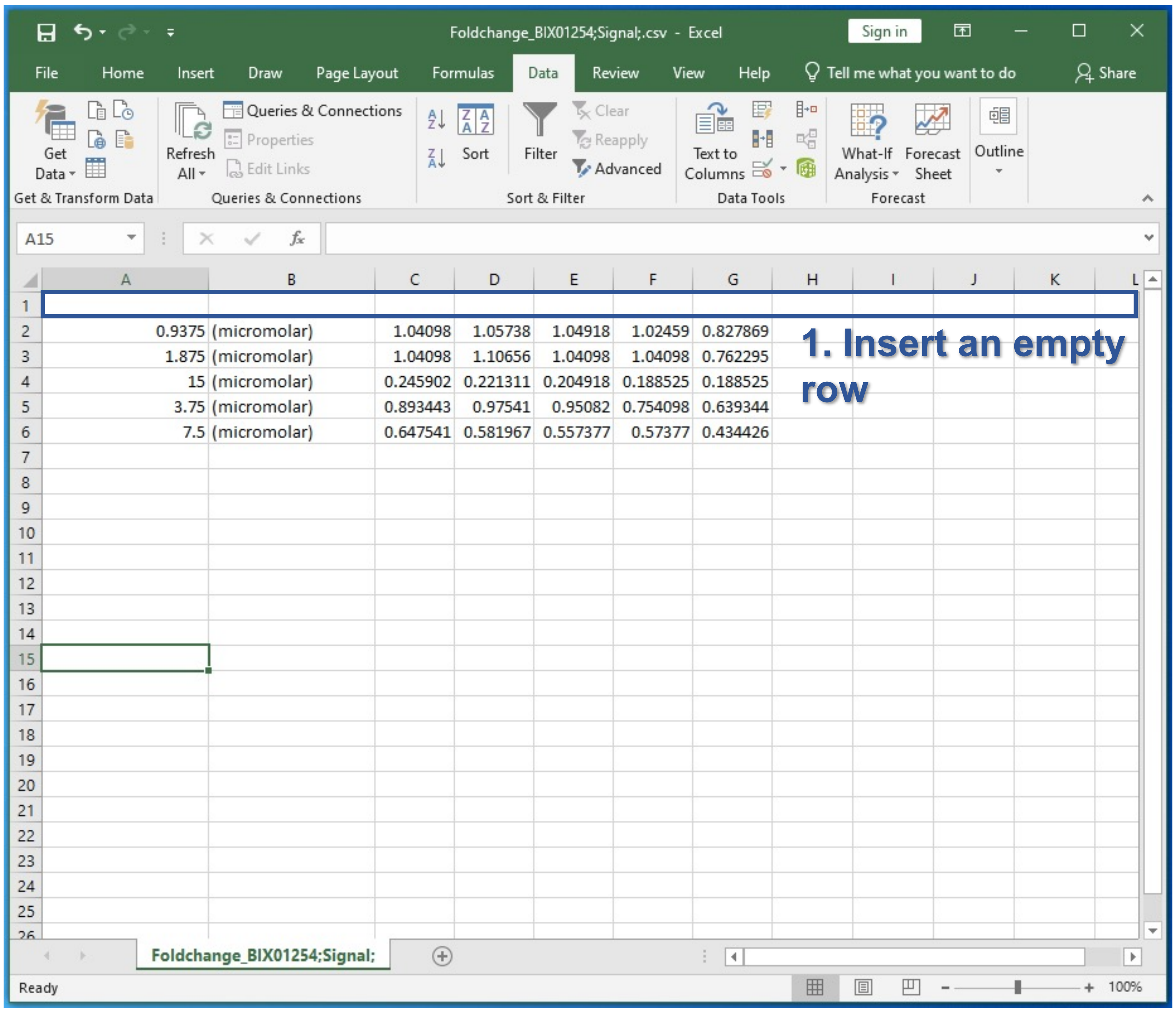
Foldchange_BIX01254;Signal;.csv - Excel
1. Insert an empty row

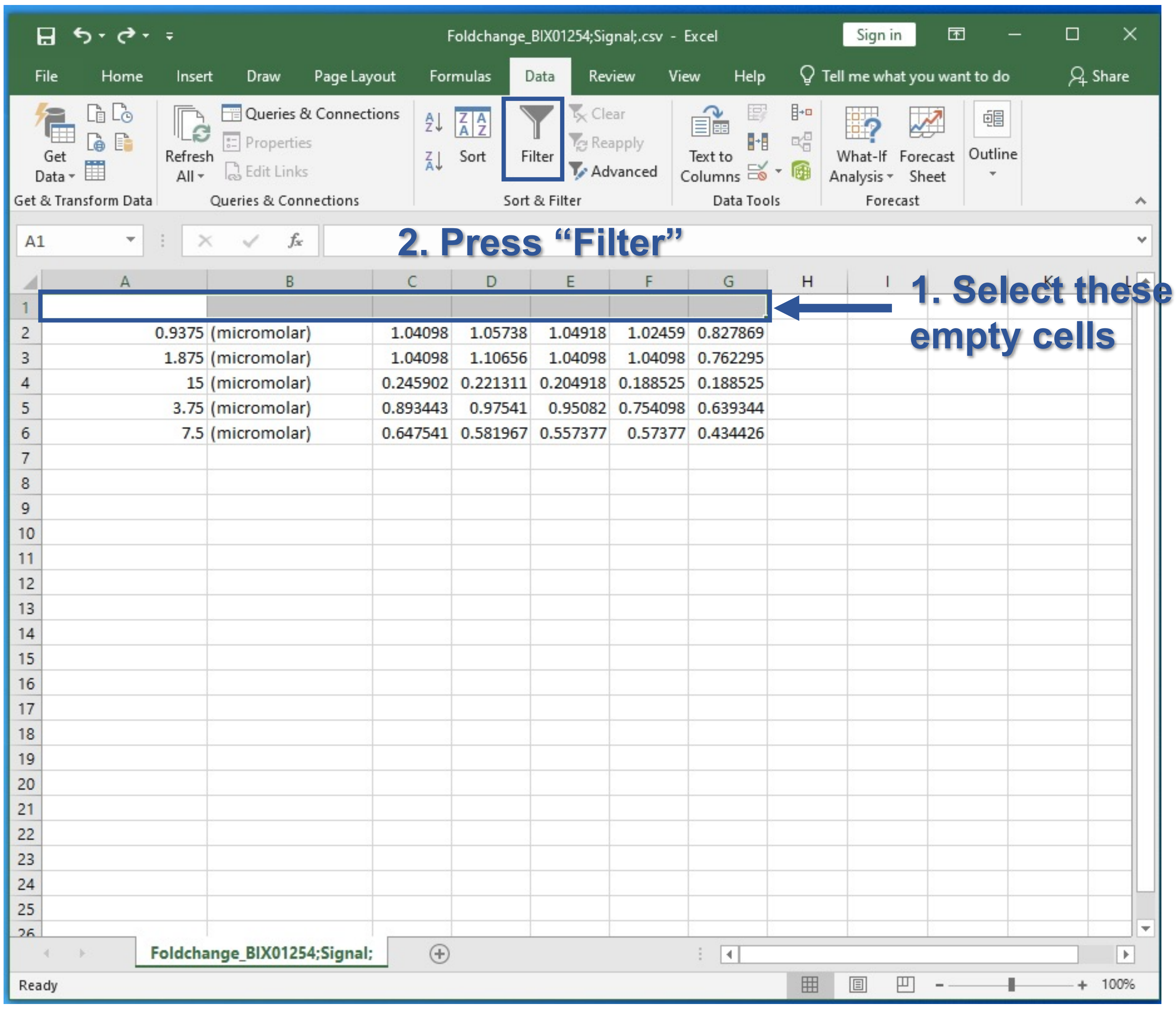
2. Press “Filter”
1. Select these empty cells

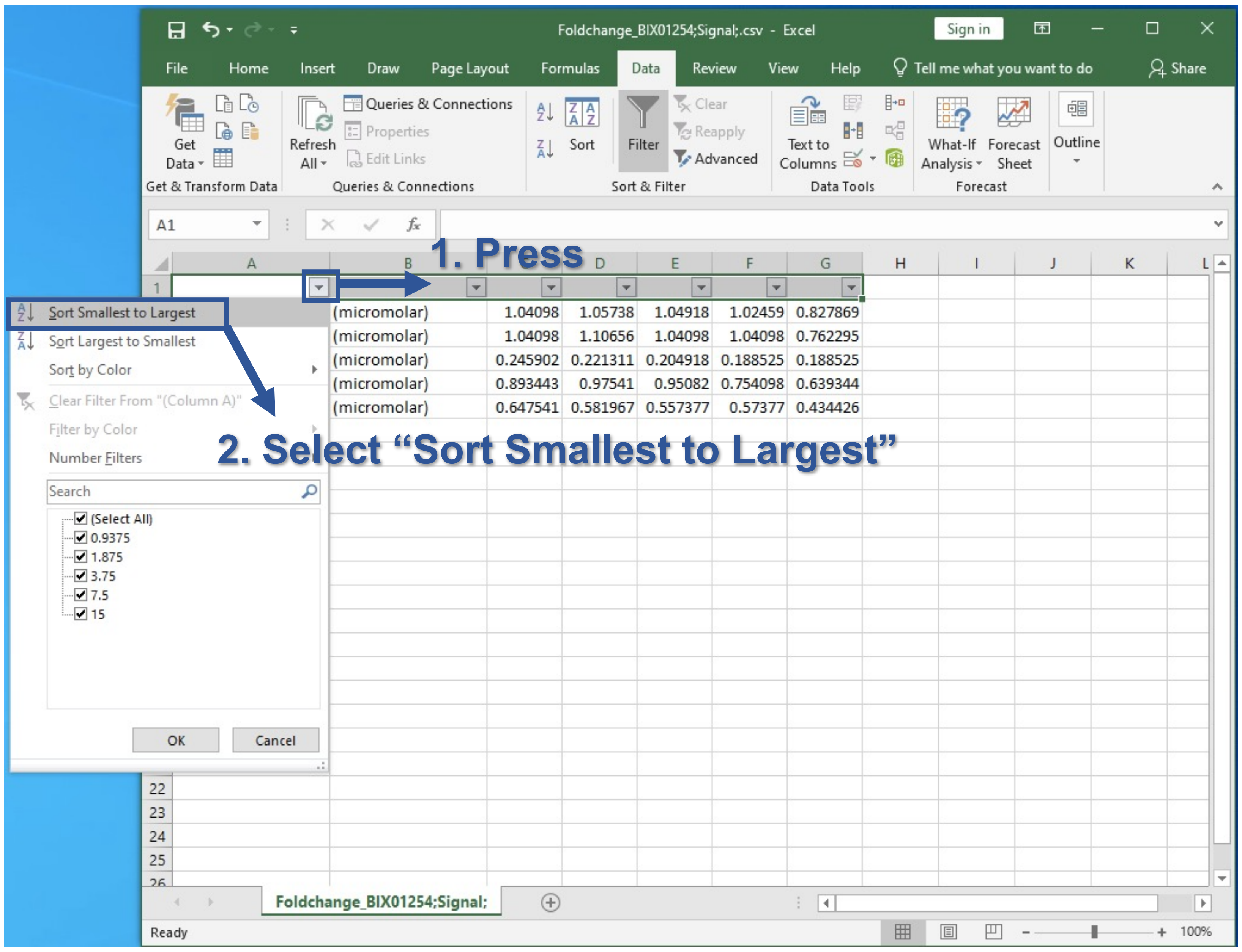
1. Press
2. Select “Sort Smallest to Largest”

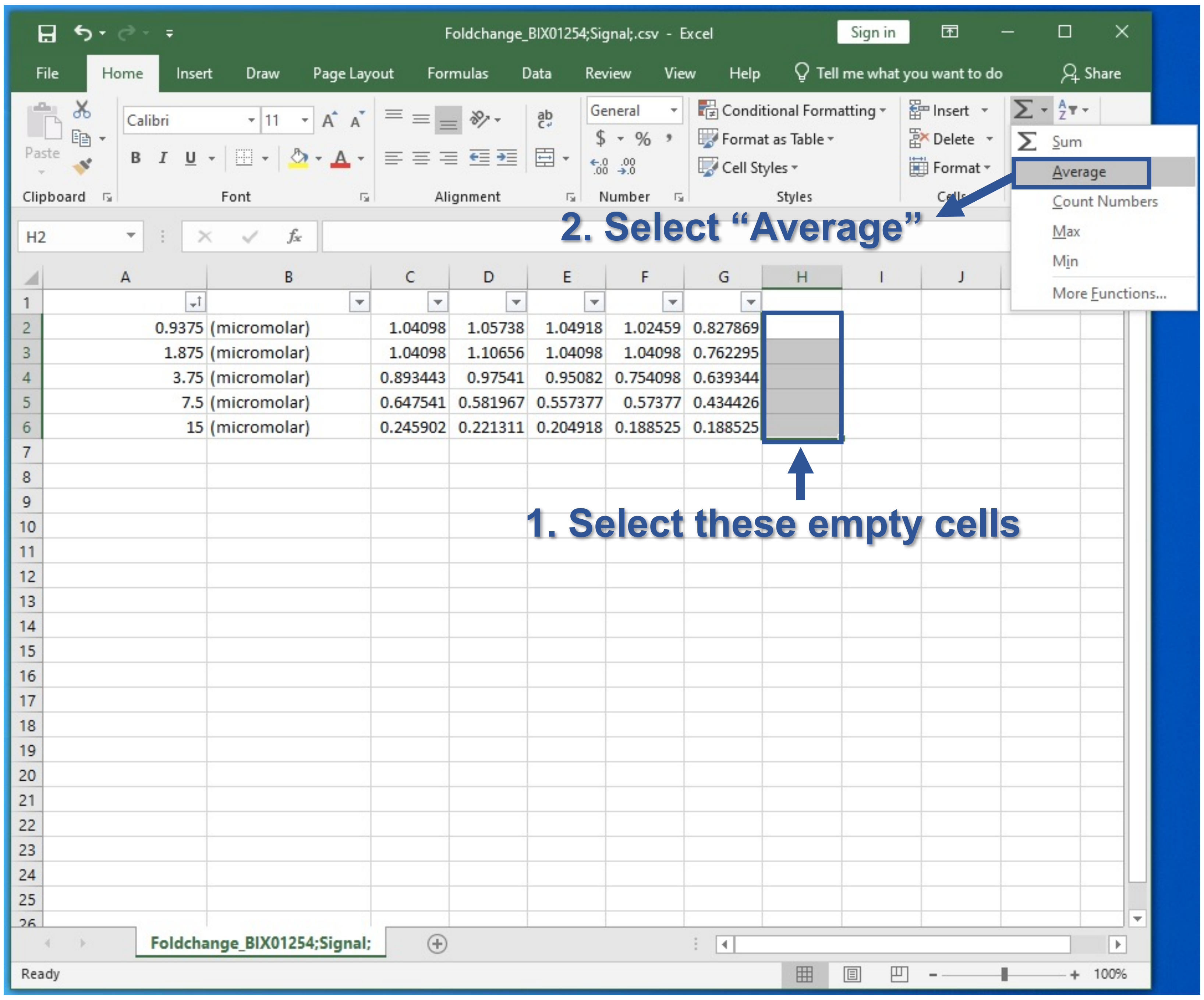
2. Select “Average”
1. Select these empty cells

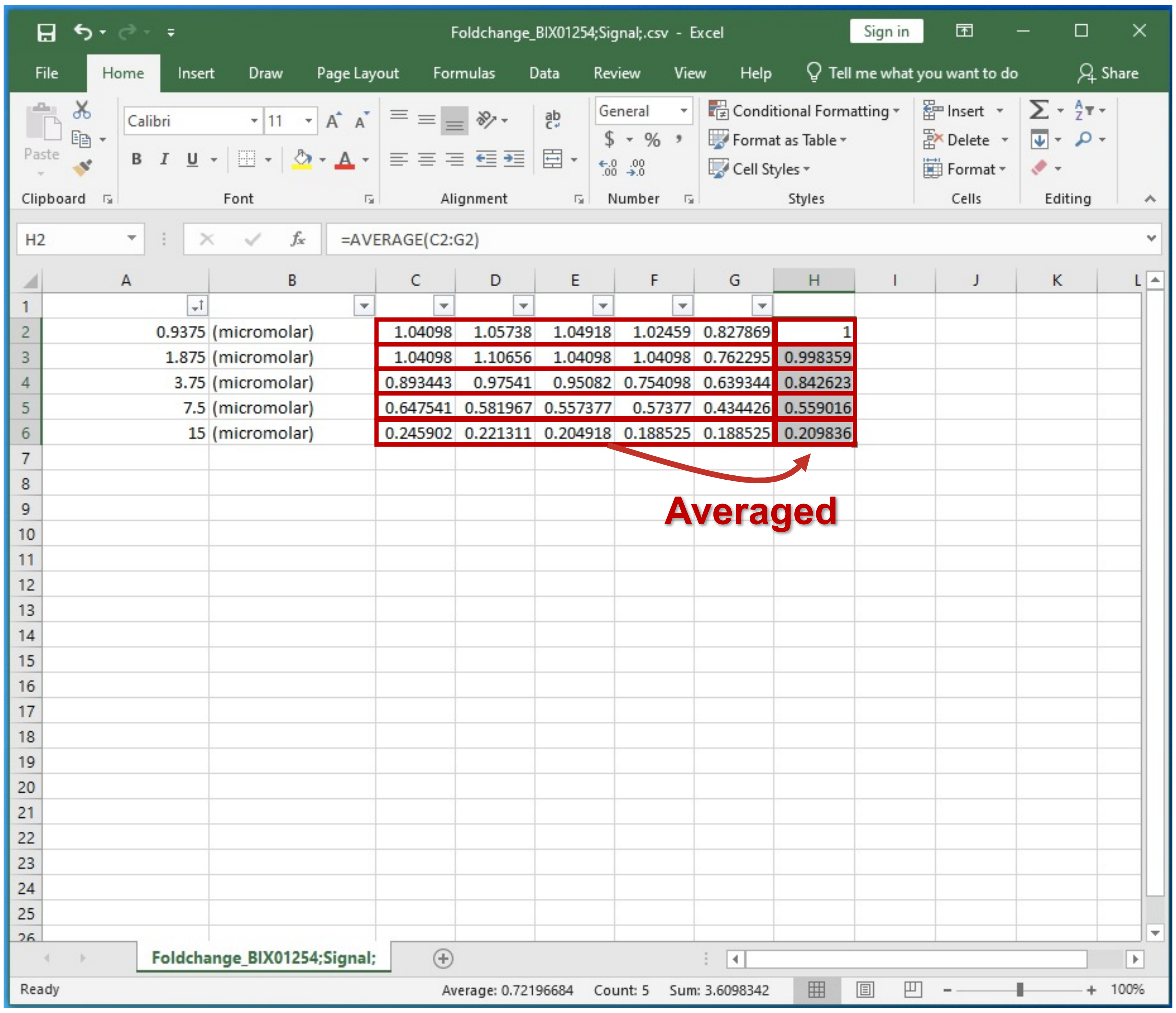
=AVERAGE(C2:G2)
Averaged

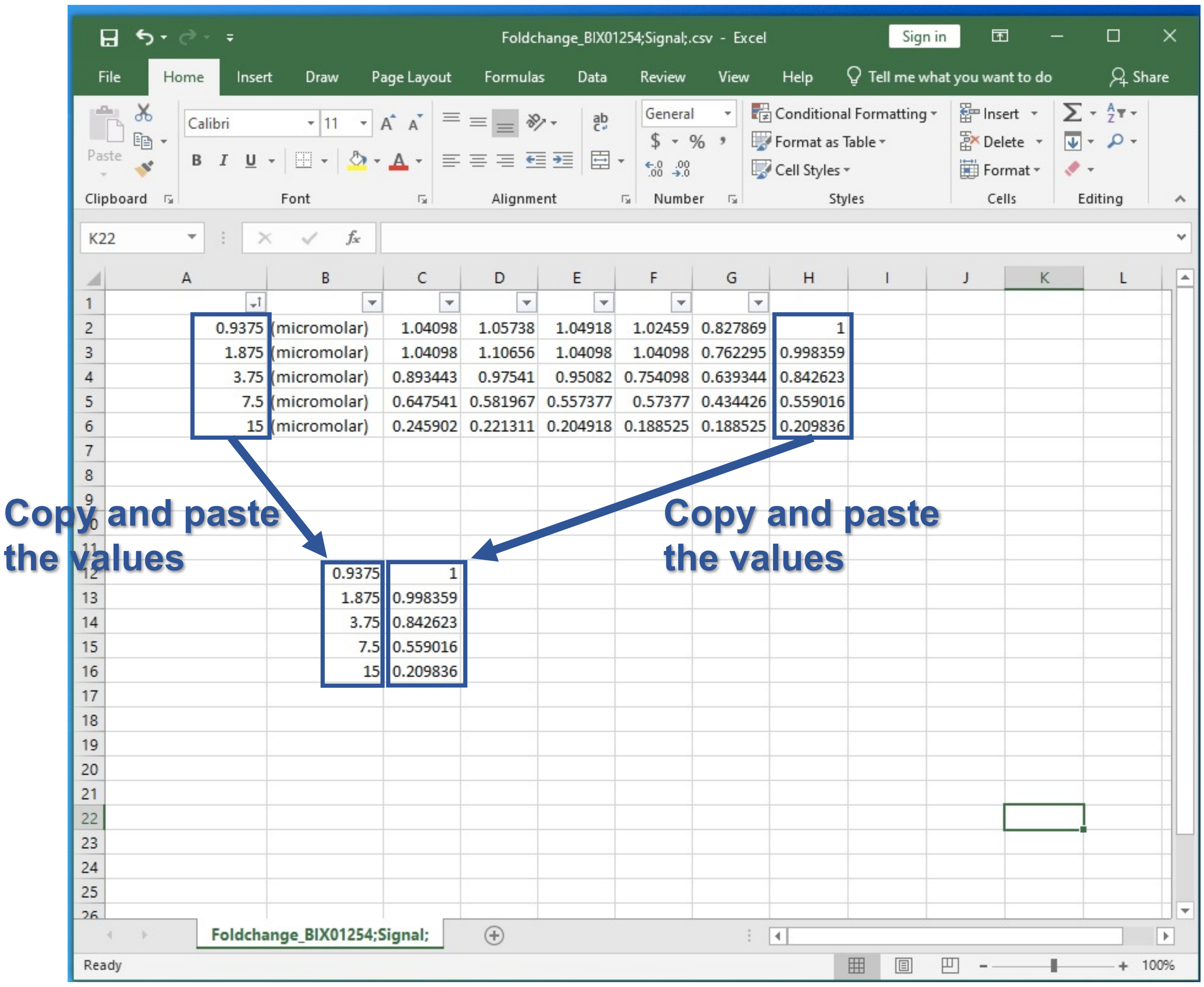
Foldchange_BIX01254;Signal;.csv - Excel
Copy and paste the values
Copy and paste the values
Foldchange_BIX01254;Signal;

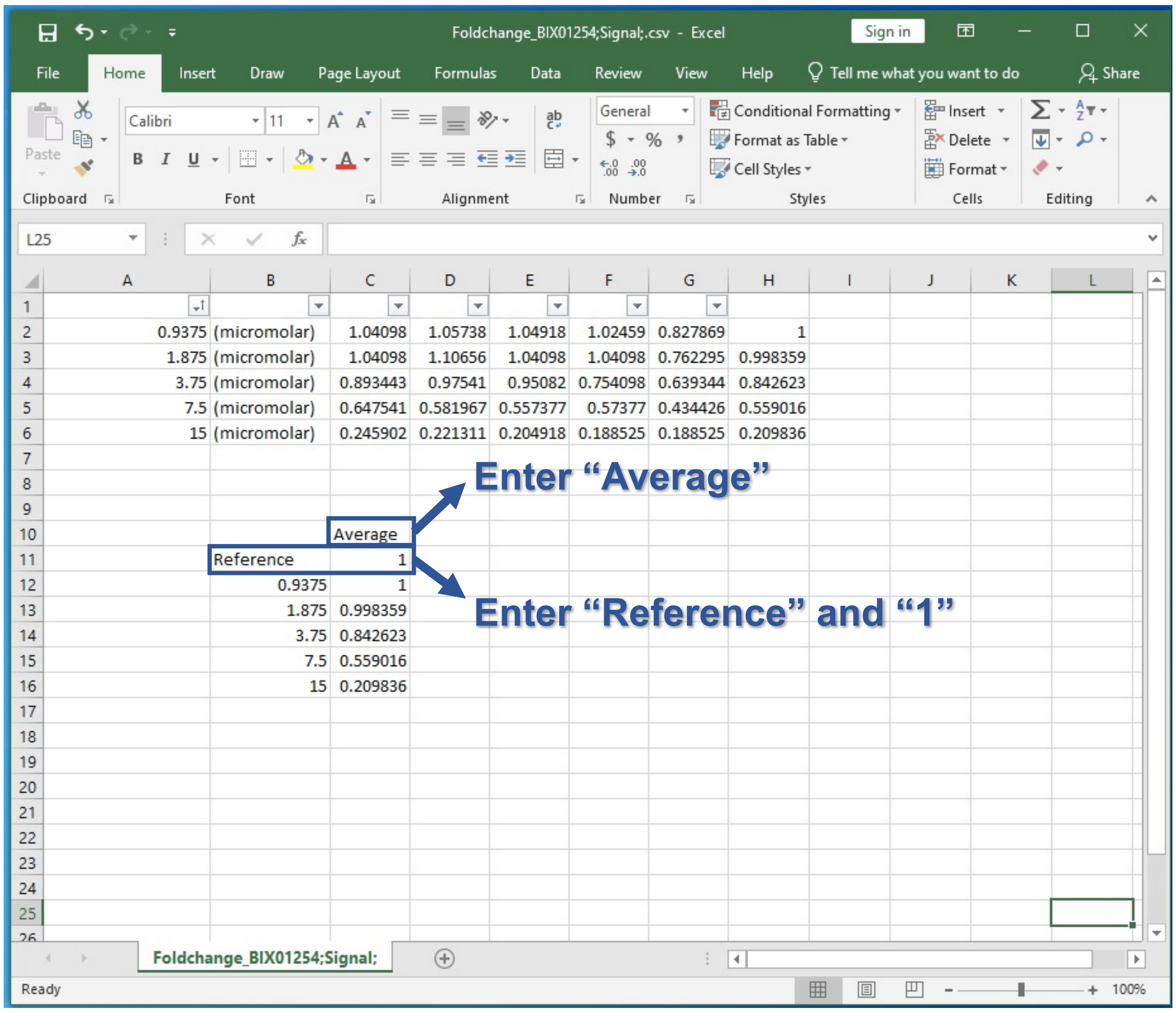
Foldchange_BIX01254;Signal;.csv - Excel
Average
Reference
Enter “Average”
Enter “Reference” and “1”
Foldchange_BIX01254;Signal;

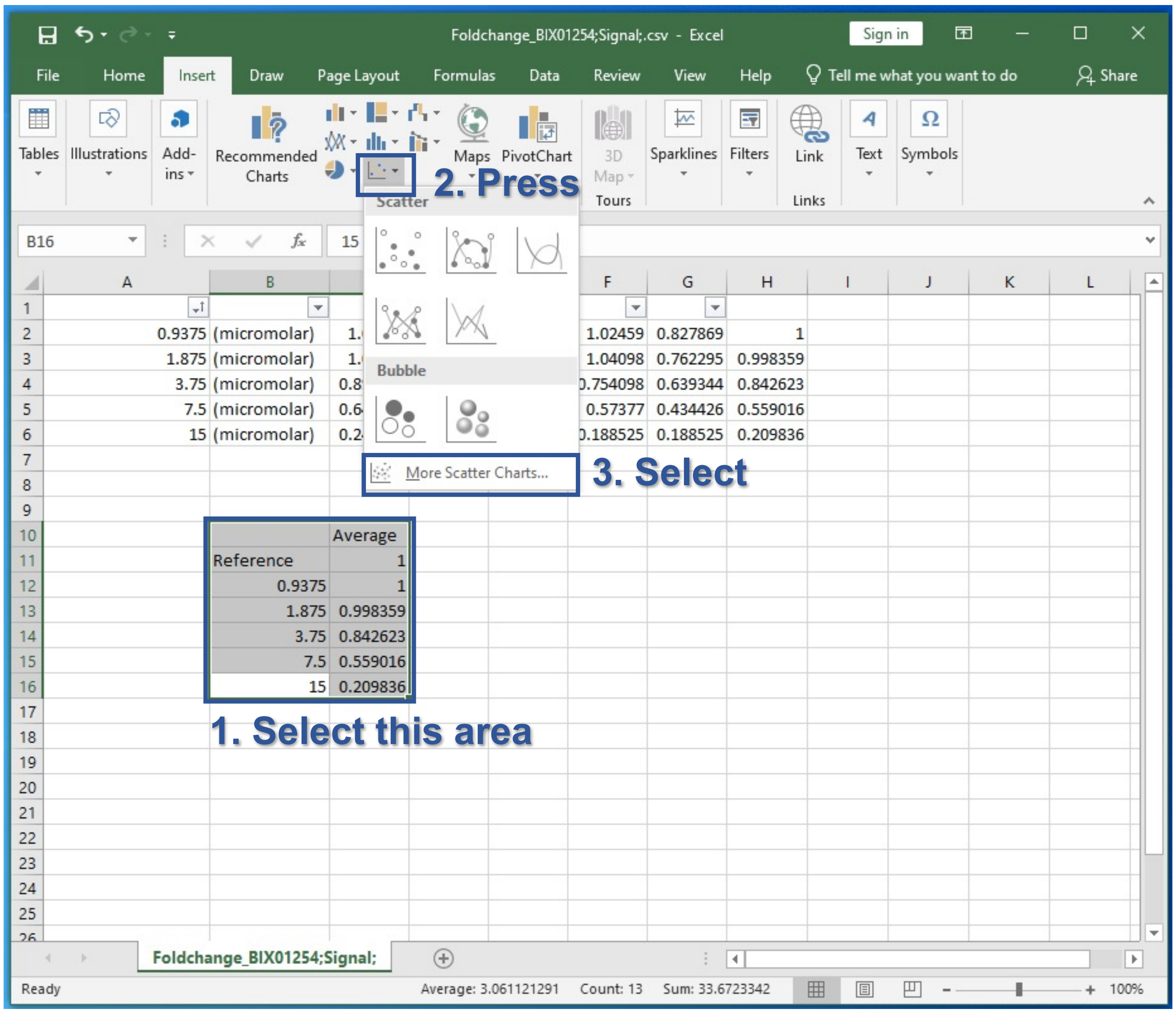
2. Press
3. Select
1. Select this area

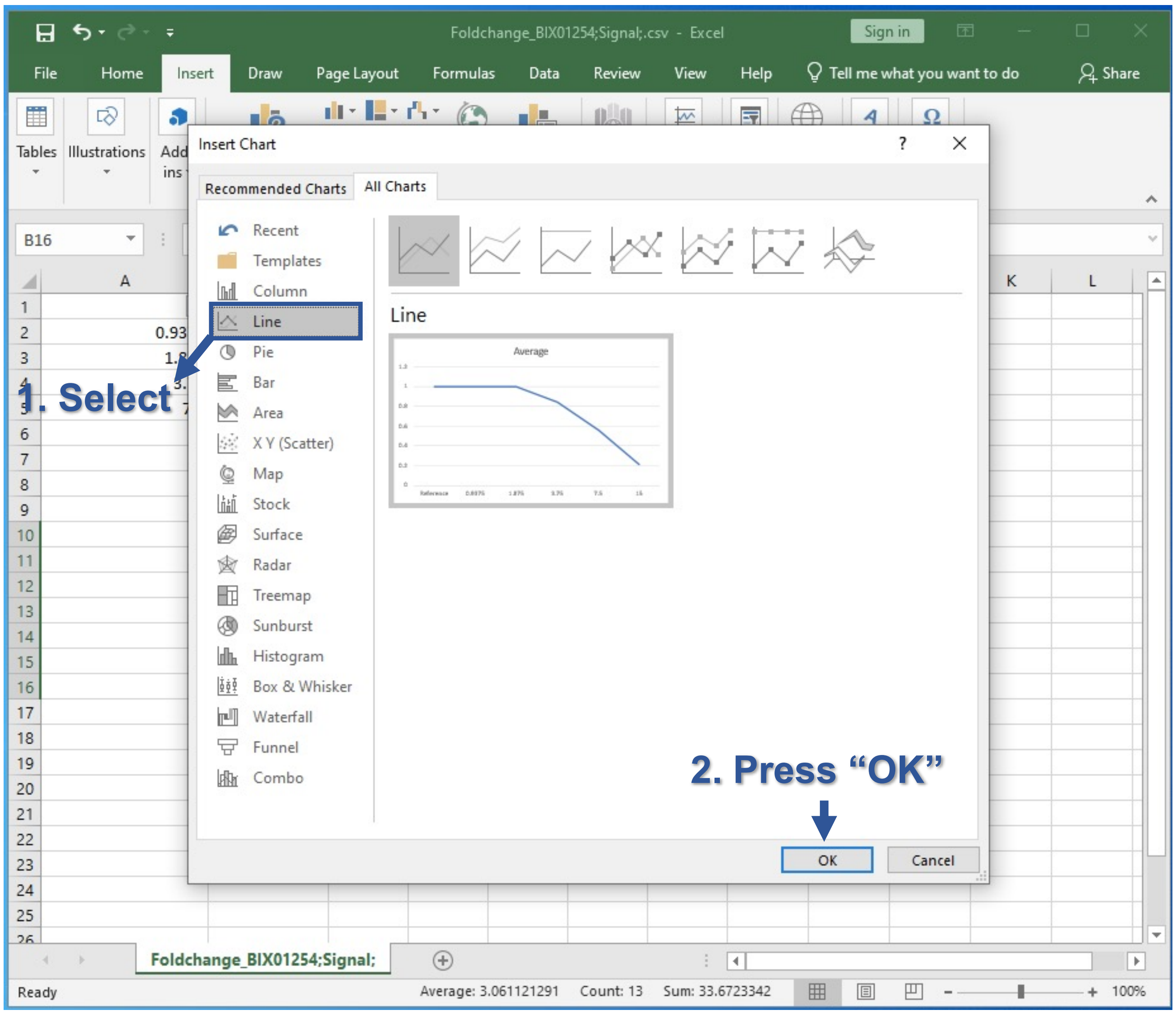
1. Select
2. Press “OK”

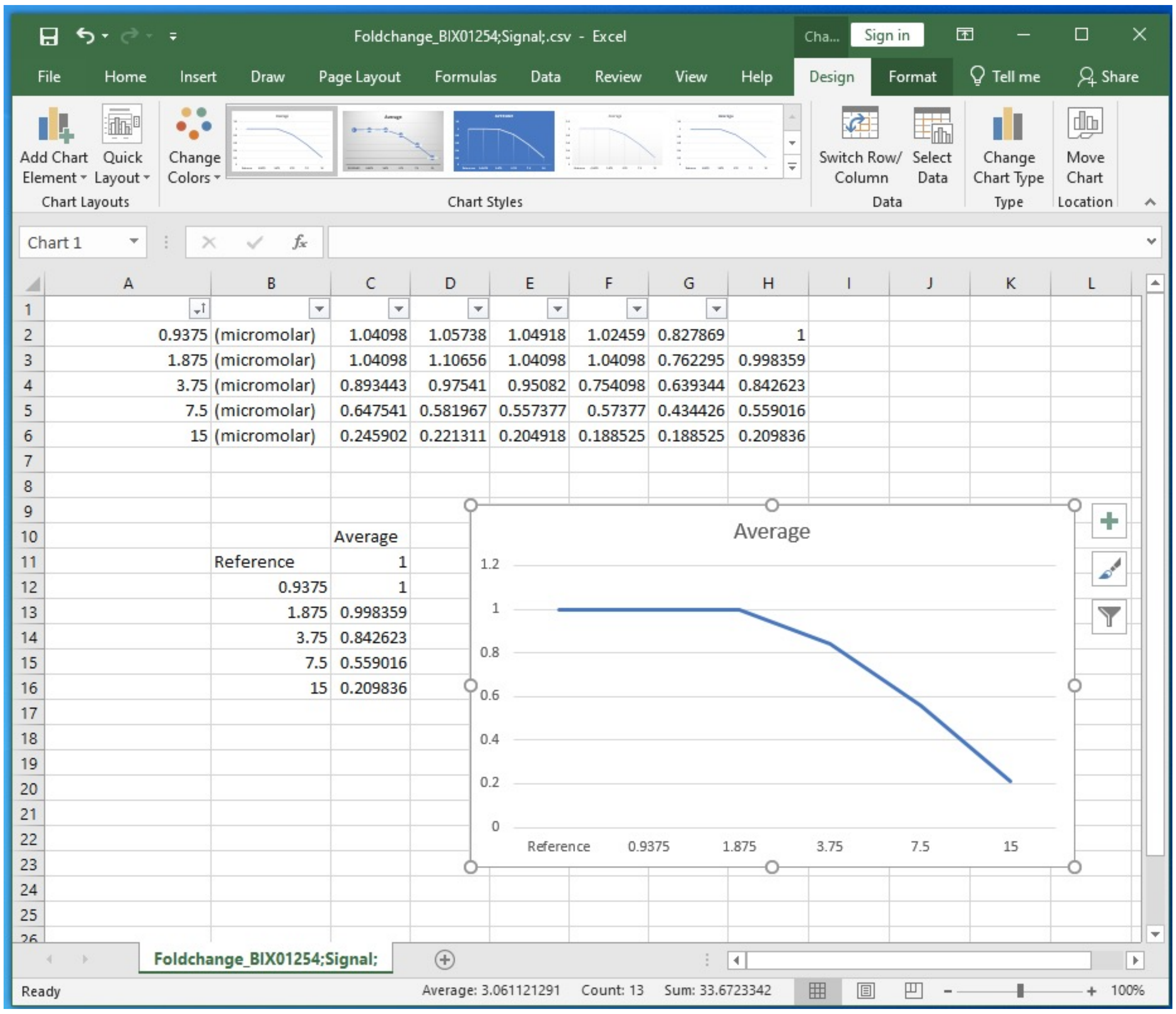
Foldchange_BIX01254;Signal;.csv - Excel
Design
Format
Chart Layouts
Chart Styles
Chart 1
Average
Reference
Foldchange_BIX01254;Signal;
Ready

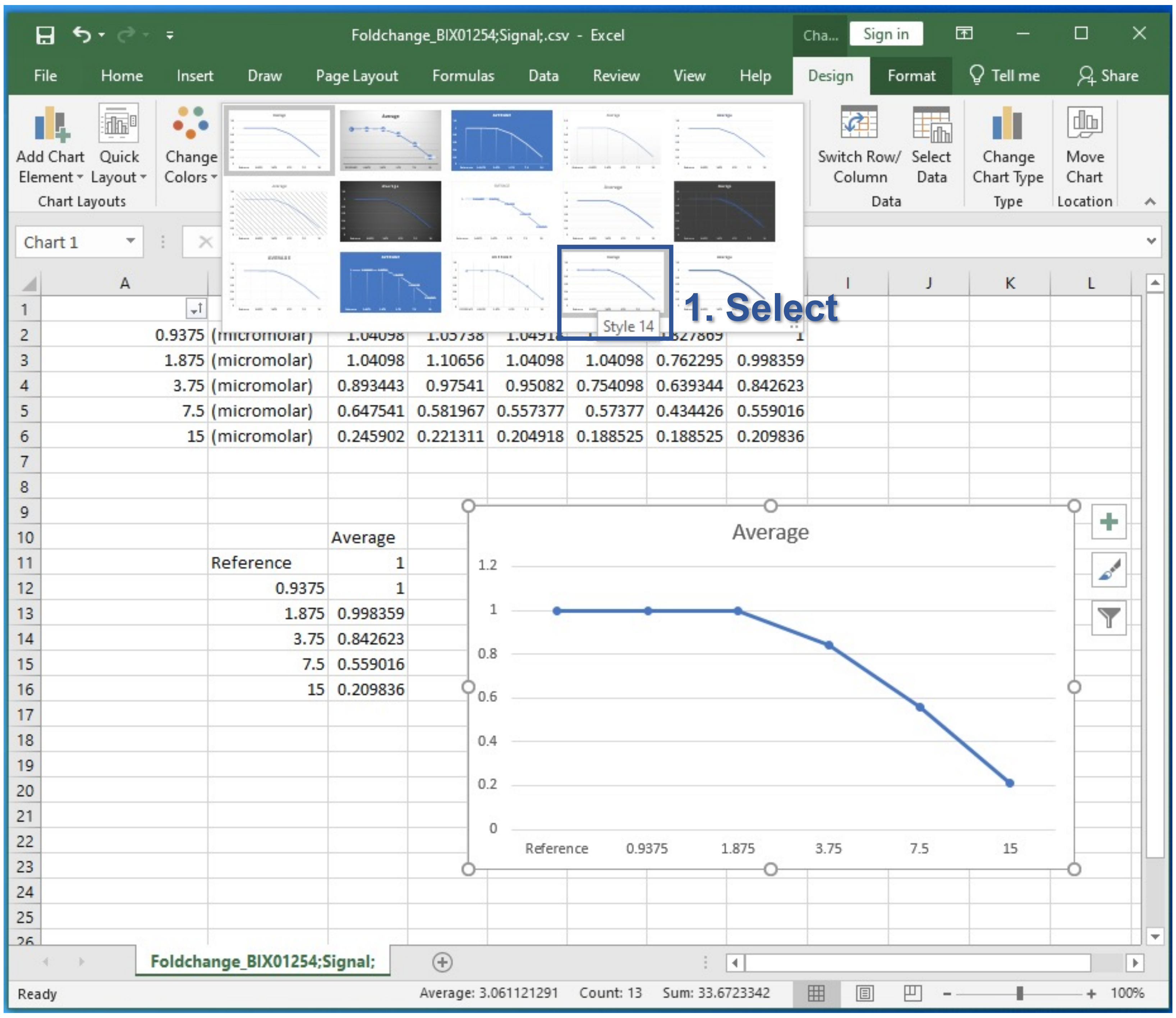
Foldchange_BIX01254;Signal;.csv - Excel
Style 14
1. Select
Average
Reference
Foldchange_BIX01254;Signal;
Ready

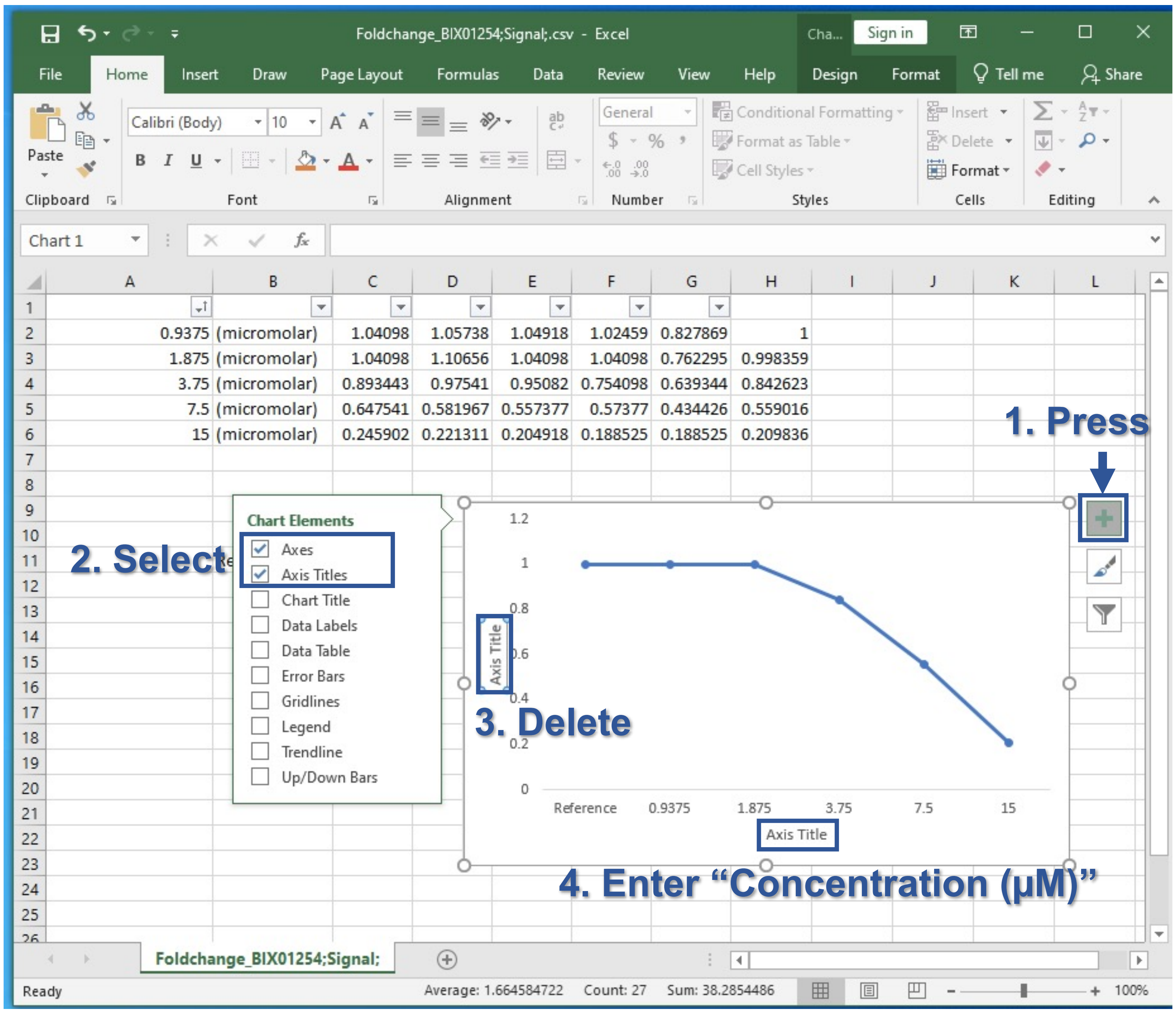
Foldchange_BIX01254;Signal;.csv - Excel
1. Press
2. Select
Chart Elements
Axes
Axis Titles
Chart Title
Data Labels
Data Table
Error Bars
Gridlines
Legend
Trendline
Up/Down Bars
Axis Title
3. Delete
Reference
0.9375
1.875
3.75
7.5
15
Axis Title
4. Enter “Concentration (µM)”
Foldchange_BIX01254;Signal;

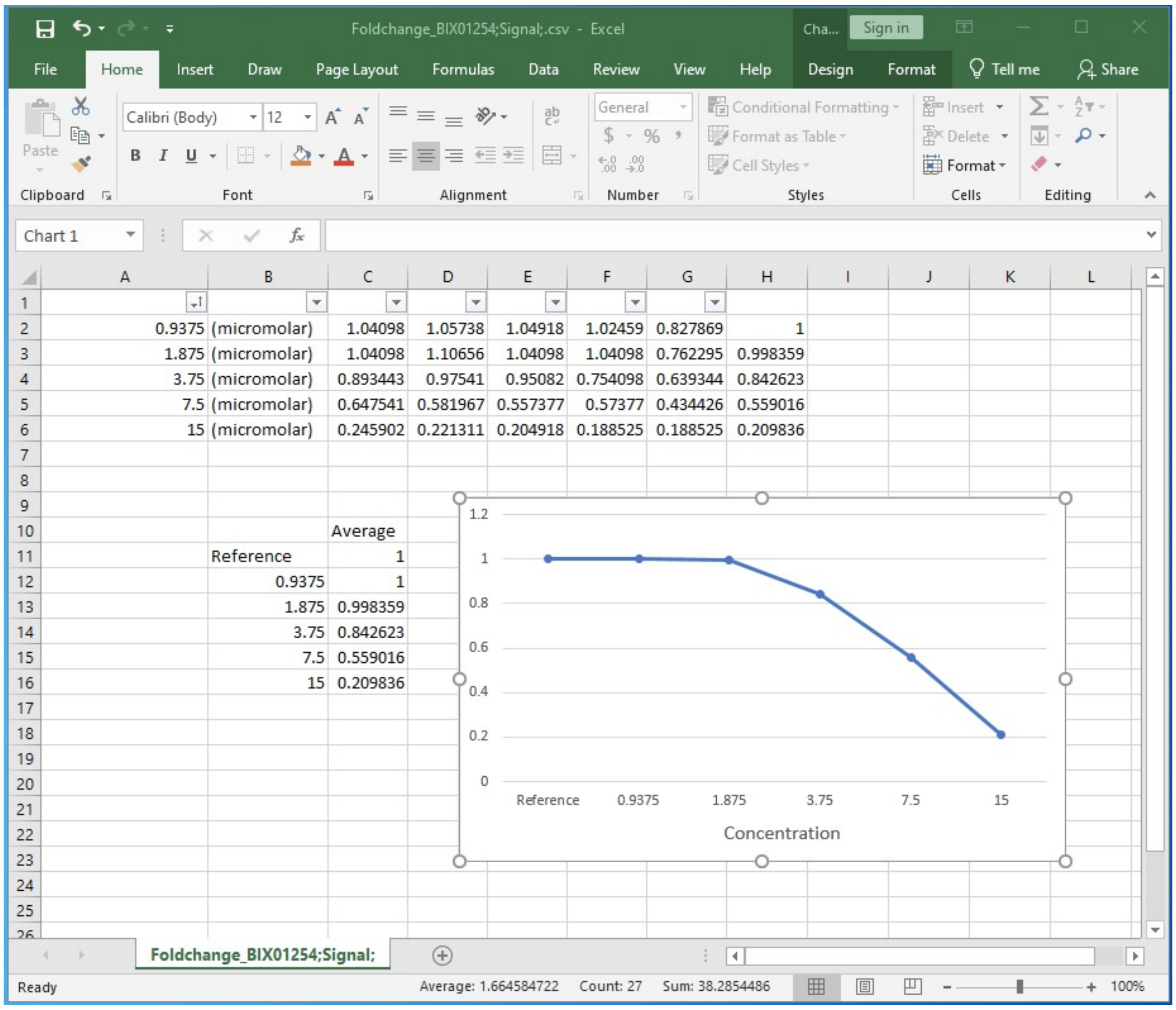
Foldchange_BIX01254;Signal;.csv - Excel
Average
Reference 1
0.9375 1
1.875 0.998359
3.75 0.842623
7.5 0.559016
15 0.209836
Reference
0.9375
1.875
3.75
7.5
15
Concentration
Foldchange_BIX01254;Signal;

❖ Validation (GM_Converter)

- The demo data of validation is stored in “Lead compound_Step-3_Validation” (purple frame) folder of “Demo_2_Lead compound” folder (red frame).

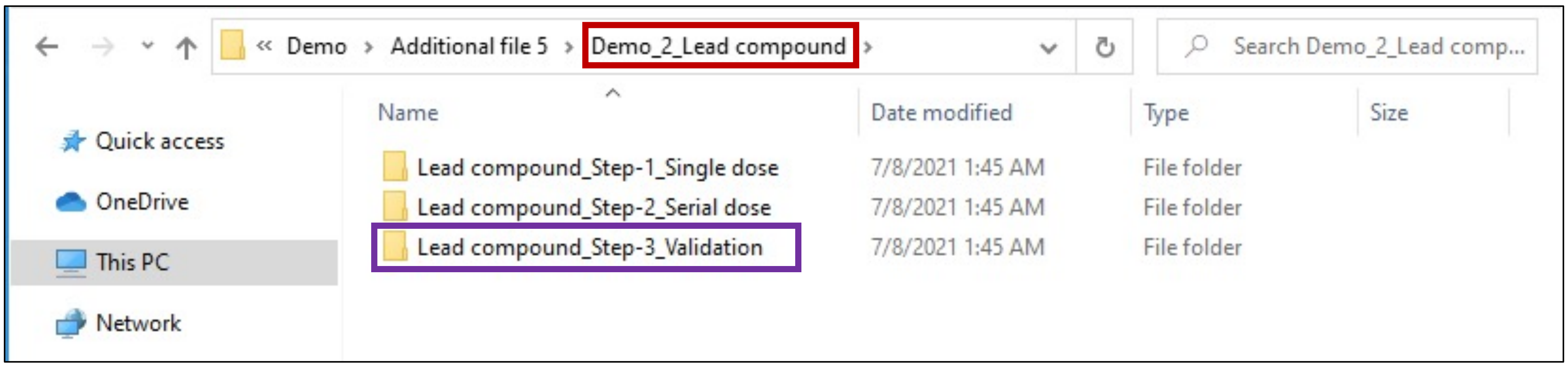

Open the “Lead compound_Step-3_Validation” folder (purple frame)

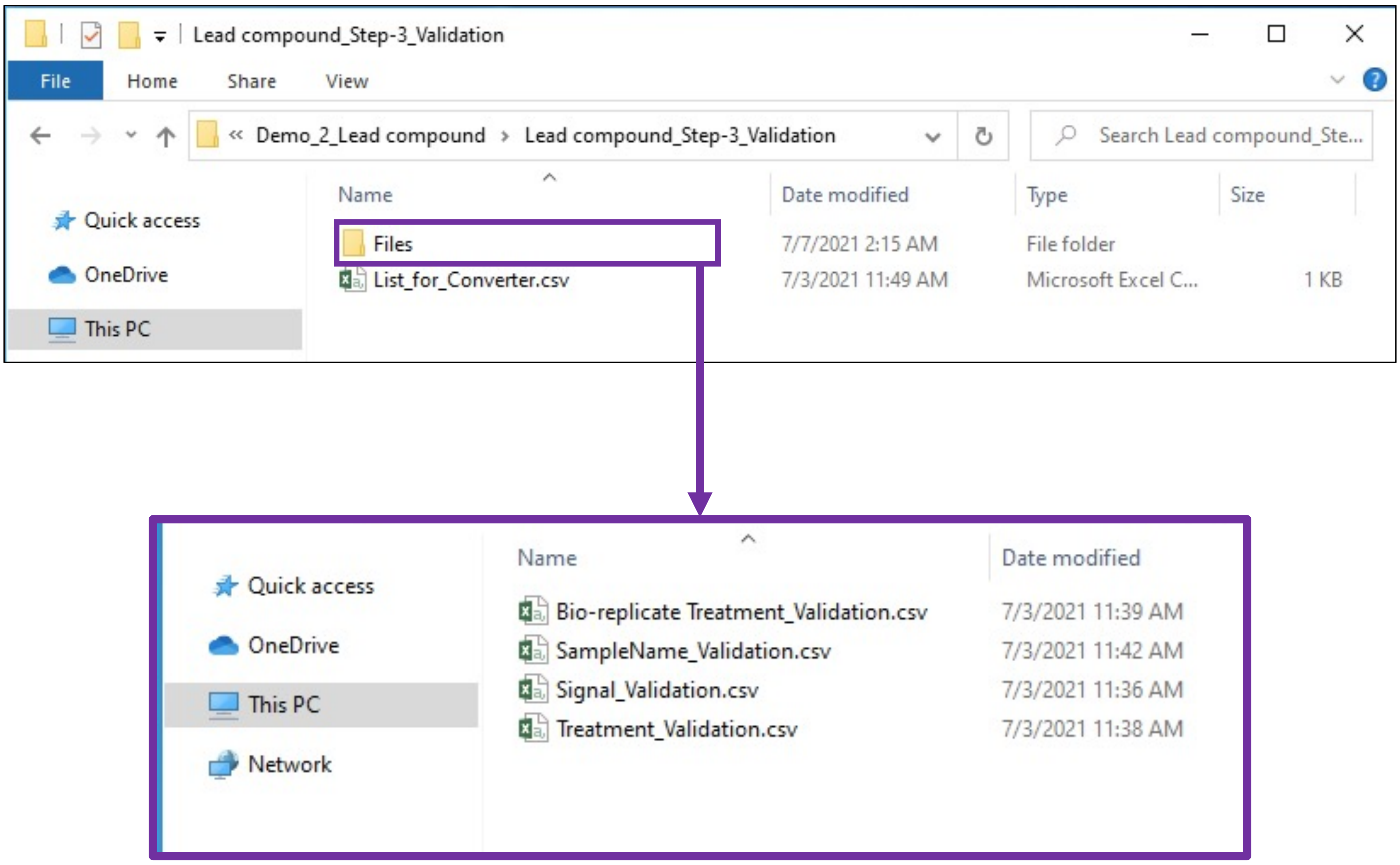

- Files in “Files” folder for using converter (Validation)

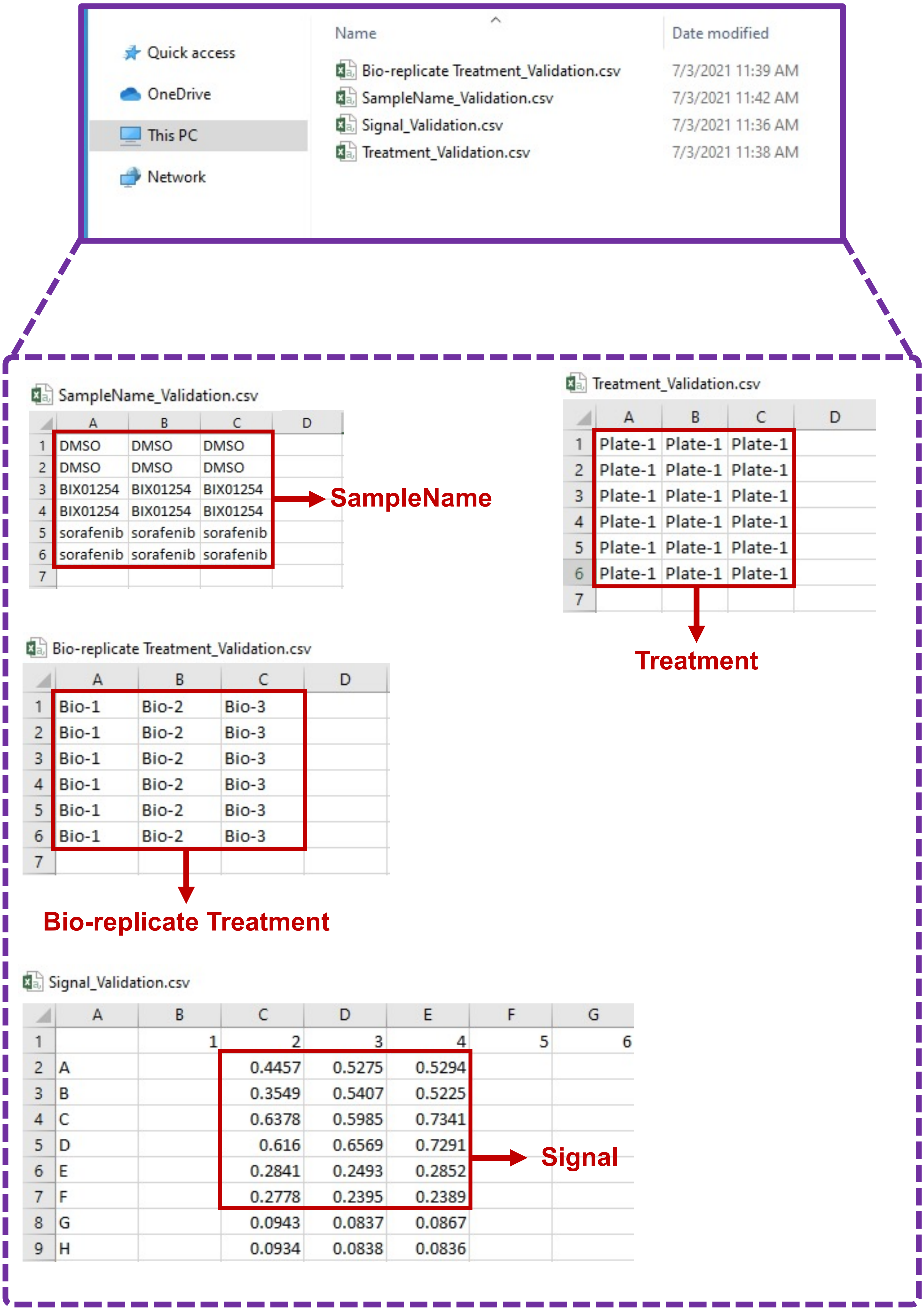

SampleName_Validation.csv

	A	B	C	D
1	DMSO	DMSO	DMSO	
2	DMSO	DMSO	DMSO	
3	BIX01254	BIX01254	BIX01254	
4	BIX01254	BIX01254	BIX01254	
5	sorafenib	sorafenib	sorafenib	
6	sorafenib	sorafenib	sorafenib	
7				

Treatment_Validation.csv

	A	B	C	D
1	Plate-1	Plate-1	Plate-1	
2	Plate-1	Plate-1	Plate-1	
3	Plate-1	Plate-1	Plate-1	
4	Plate-1	Plate-1	Plate-1	
5	Plate-1	Plate-1	Plate-1	
6	Plate-1	Plate-1	Plate-1	
7				

Bio-replicate Treatment_Validation.csv

	A	B	C	D
1	Bio-1	Bio-2	Bio-3	
2	Bio-1	Bio-2	Bio-3	
3	Bio-1	Bio-2	Bio-3	
4	Bio-1	Bio-2	Bio-3	
5	Bio-1	Bio-2	Bio-3	
6	Bio-1	Bio-2	Bio-3	
7				

Signal_Validation.csv

	A	B	C	D	E	F	G
1		1	2	3	4	5	6
2	A		0.4457	0.5275	0.5294		
3	B		0.3549	0.5407	0.5225		
4	C		0.6378	0.5985	0.7341		
5	D		0.616	0.6569	0.7291		
6	E		0.2841	0.2493	0.2852		
7	F		0.2778	0.2395	0.2389		
8	G		0.0943	0.0837	0.0867		
9	H		0.0934	0.0838	0.0836		

List_for_Converter.csv”

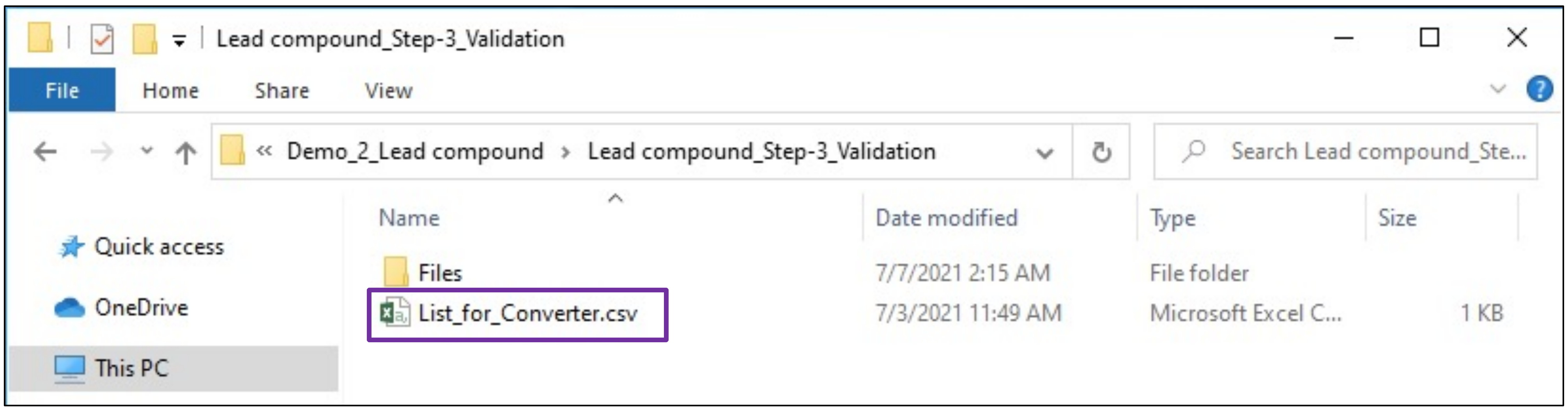

- The data in the first row of “Converted_data.csv” file (the result file output from GM_Converter) is from the first row of “List_for_Converter” file (red frame). Please see the Figure A1-A11 in Additional file 2 for the detailed concept of GM_Converter.

- The data of files listed in the same row of ”List_for_Converter.csv” (blue frame) will be integrated. Please see the Figure A1-A11 in Additional file 2 for the detailed concept of GM_Converter.

“Converted_data.csv”

First row →

	A	B	C	D	E
1	SampleName_Reagent	Treatment_Plate	Bio-replicate Treatment_Bio-rep	Signal	
2	DMSO	Plate-1	Bio-1	0.4457	
3	DMSO	Plate-1	Bio-2	0.5275	
4	DMSO	Plate-1	Bio-3	0.5294	
5	DMSO	Plate-1	Bio-1	0.3549	
6	DMSO	Plate-1	Bio-2	0.5407	
7	DMSO	Plate-1	Bio-3	0.5225	

“List_for_Converter.csv” file

	A	B	C	D	E
1	SampleName_Reagent	Treatment_Plate	Bio-replicate Treatment_Bio-rep	Signal	
2	SampleName_Validation.csv	Treatment_Validation.csv	Bio-replicate Treatment_Validation.csv	Signal_Validation.csv	
3					

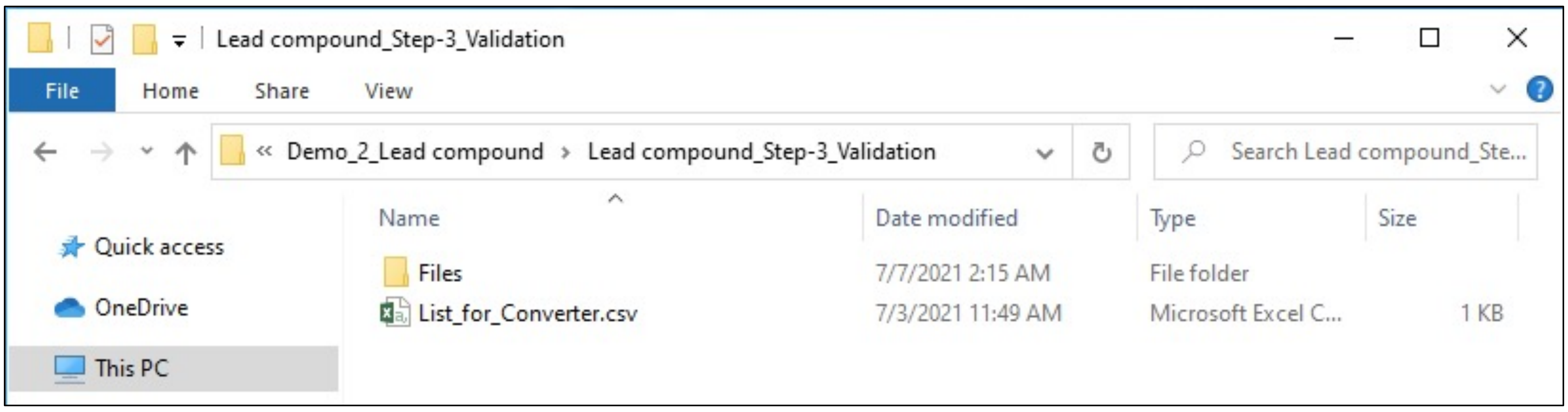

Copy and paste the two executive files of GM_Converter (GateMultiplex_converter.exe and Converter.exe) into "Lead compound_Step-3_Validation" folder (purple frames and purple arrows)

*The two executive files should be placed in the same folder

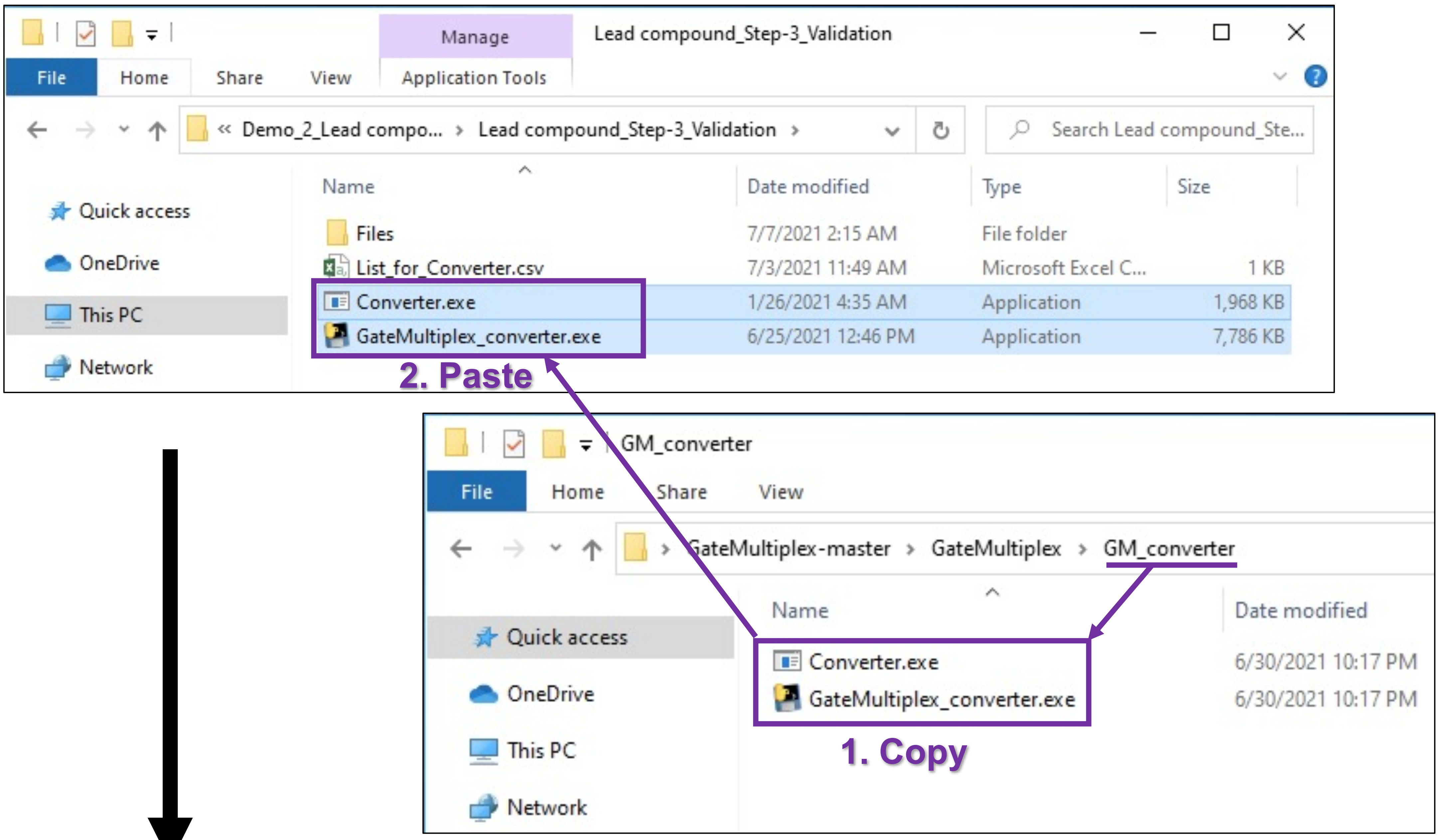

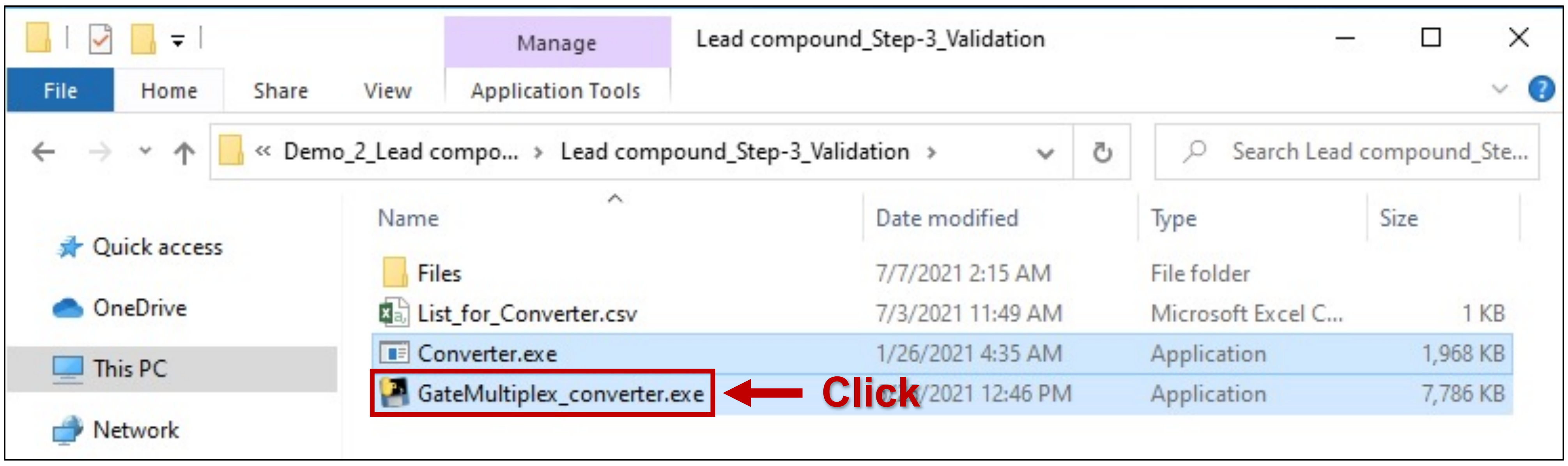

Activate the GM_Converter by a double-clicking on "GateMultiplex_converter.exe" (red frame)

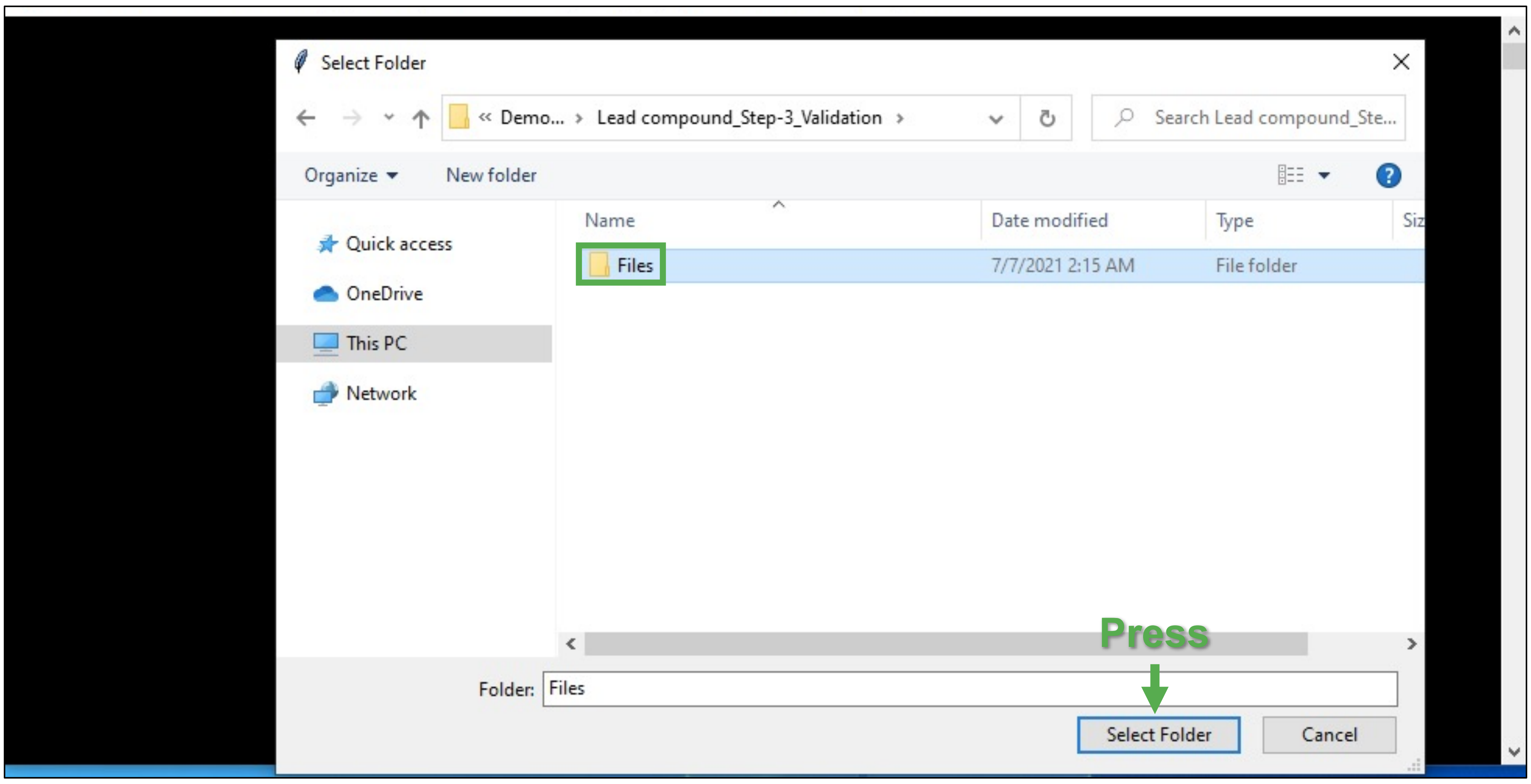

Select the folder "Files" (Additional file 5 > Demo_2_Lead compound > Lead compound_Step-3_Validation > Files) (green frame) and press "Select Folder" (indicated by a green arrow)

- Please see the Figure A5-A11 in Additional file 2 for the detailed parameter setting of GM_Converter.

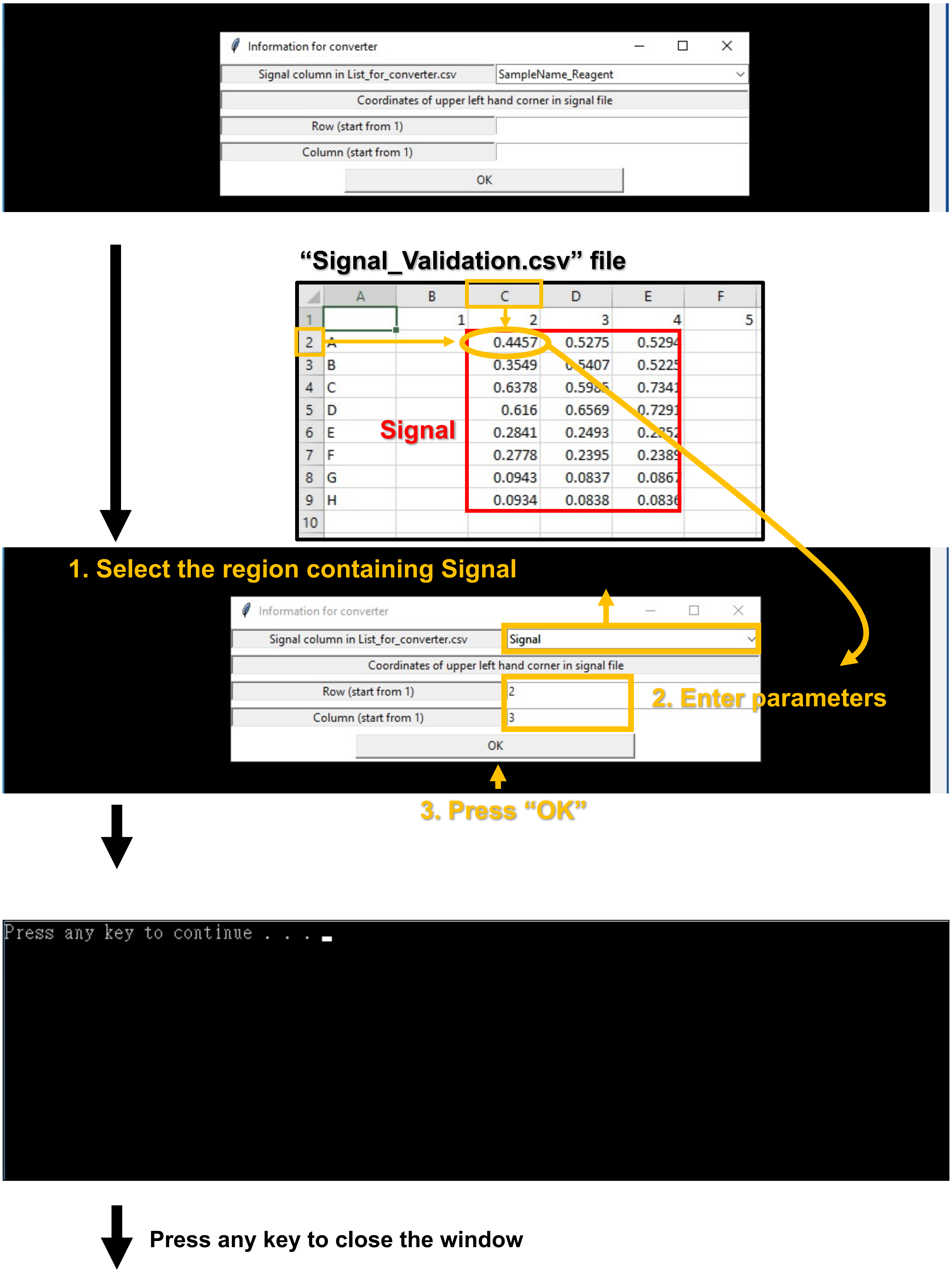

- The converted data “Converted_data.csv” (green frame) is stored in the “GM_converted_data” folder (red frame)

- The title of “Converted_data.csv” (blue frame) is the same as the title in “List_for_Converter.csv”. Please see Figure A5-A11 in Additional file 2.

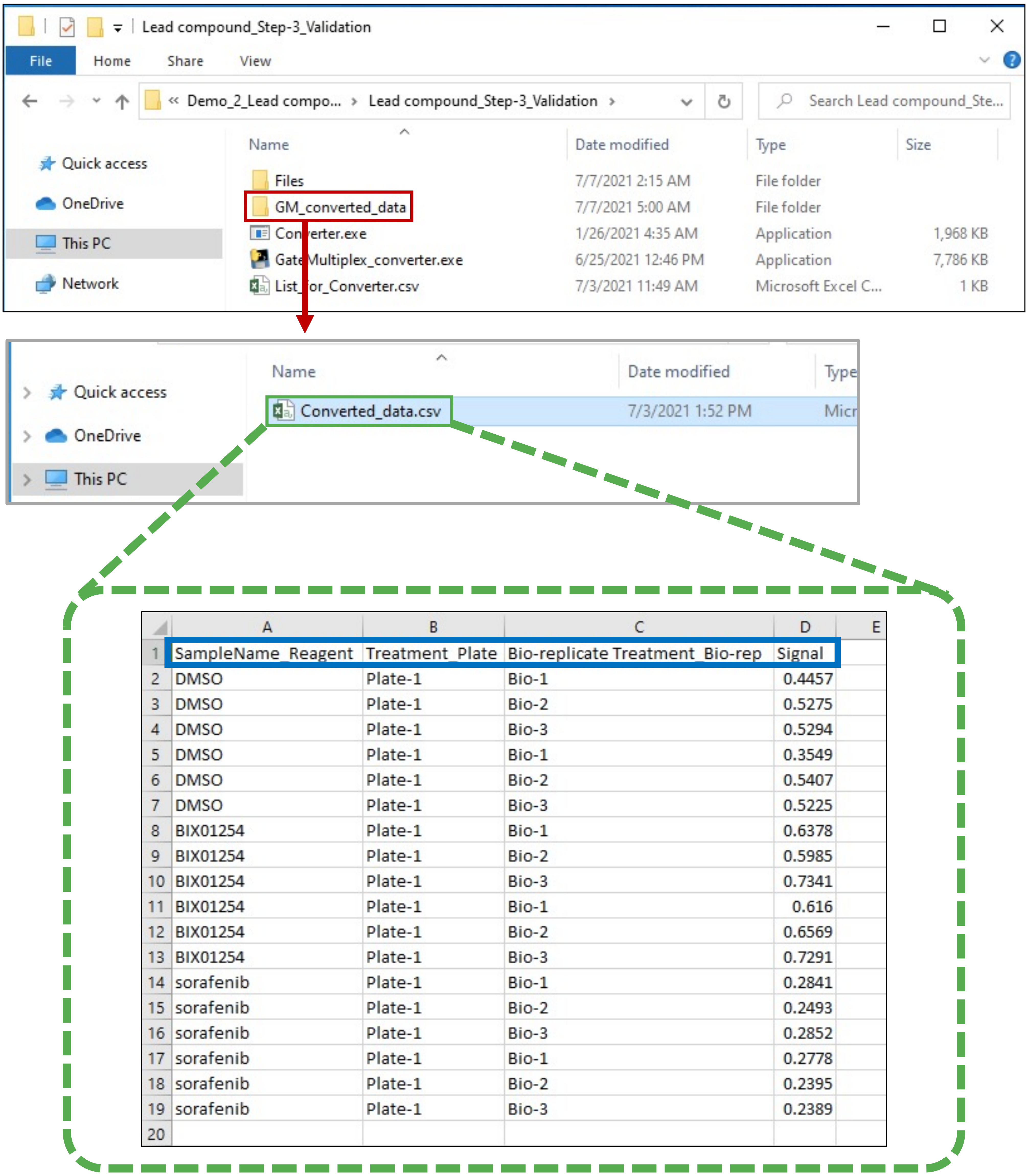

	A	B	C	D	E
1	SampleName_Reagent	Treatment_Plate	Bio-replicate Treatment_Bio-rep	Signal	
2	DMSO	Plate-1	Bio-1	0.4457	
3	DMSO	Plate-1	Bio-2	0.5275	
4	DMSO	Plate-1	Bio-3	0.5294	
5	DMSO	Plate-1	Bio-1	0.3549	
6	DMSO	Plate-1	Bio-2	0.5407	
7	DMSO	Plate-1	Bio-3	0.5225	
8	BIX01254	Plate-1	Bio-1	0.6378	
9	BIX01254	Plate-1	Bio-2	0.5985	
10	BIX01254	Plate-1	Bio-3	0.7341	
11	BIX01254	Plate-1	Bio-1	0.616	
12	BIX01254	Plate-1	Bio-2	0.6569	
13	BIX01254	Plate-1	Bio-3	0.7291	
14	sorafenib	Plate-1	Bio-1	0.2841	
15	sorafenib	Plate-1	Bio-2	0.2493	
16	sorafenib	Plate-1	Bio-3	0.2852	
17	sorafenib	Plate-1	Bio-1	0.2778	
18	sorafenib	Plate-1	Bio-2	0.2395	
19	sorafenib	Plate-1	Bio-3	0.2389	
20					

❖ Validation (GM_Basic)

- The two executive files of GM_Basic are both stored in the “GM_basic” folder (blue frame) and should be placed in the same folder for operation.

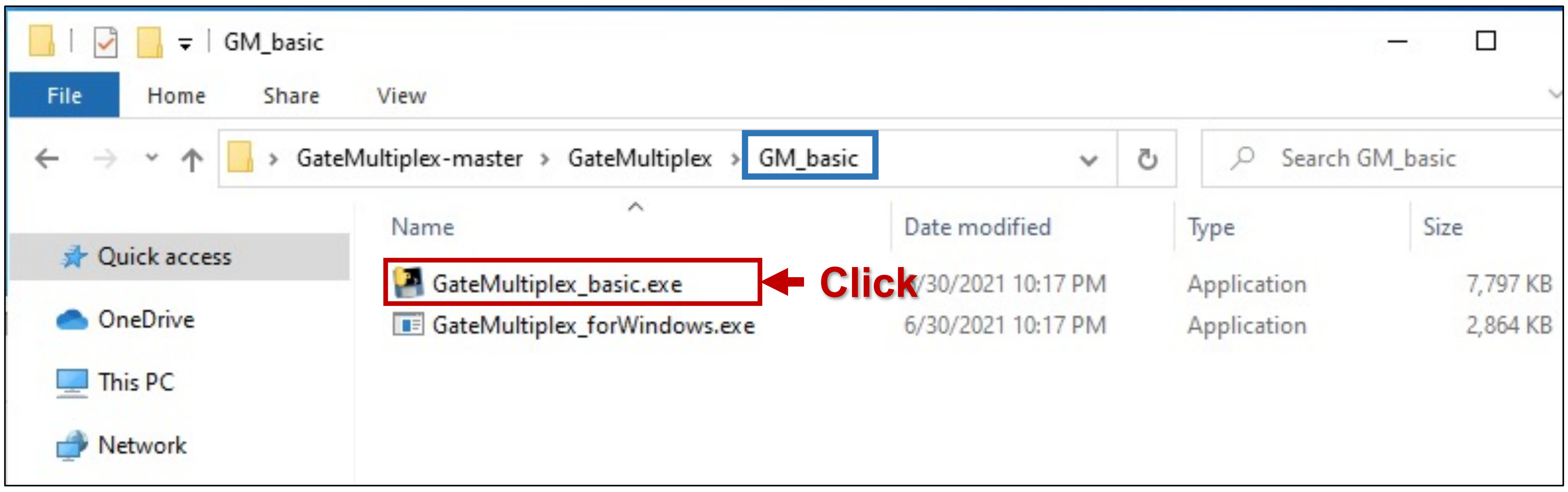

Activate the GM_basic by a double-clicking on “GateMultiplex_basic.exe” (red frame)

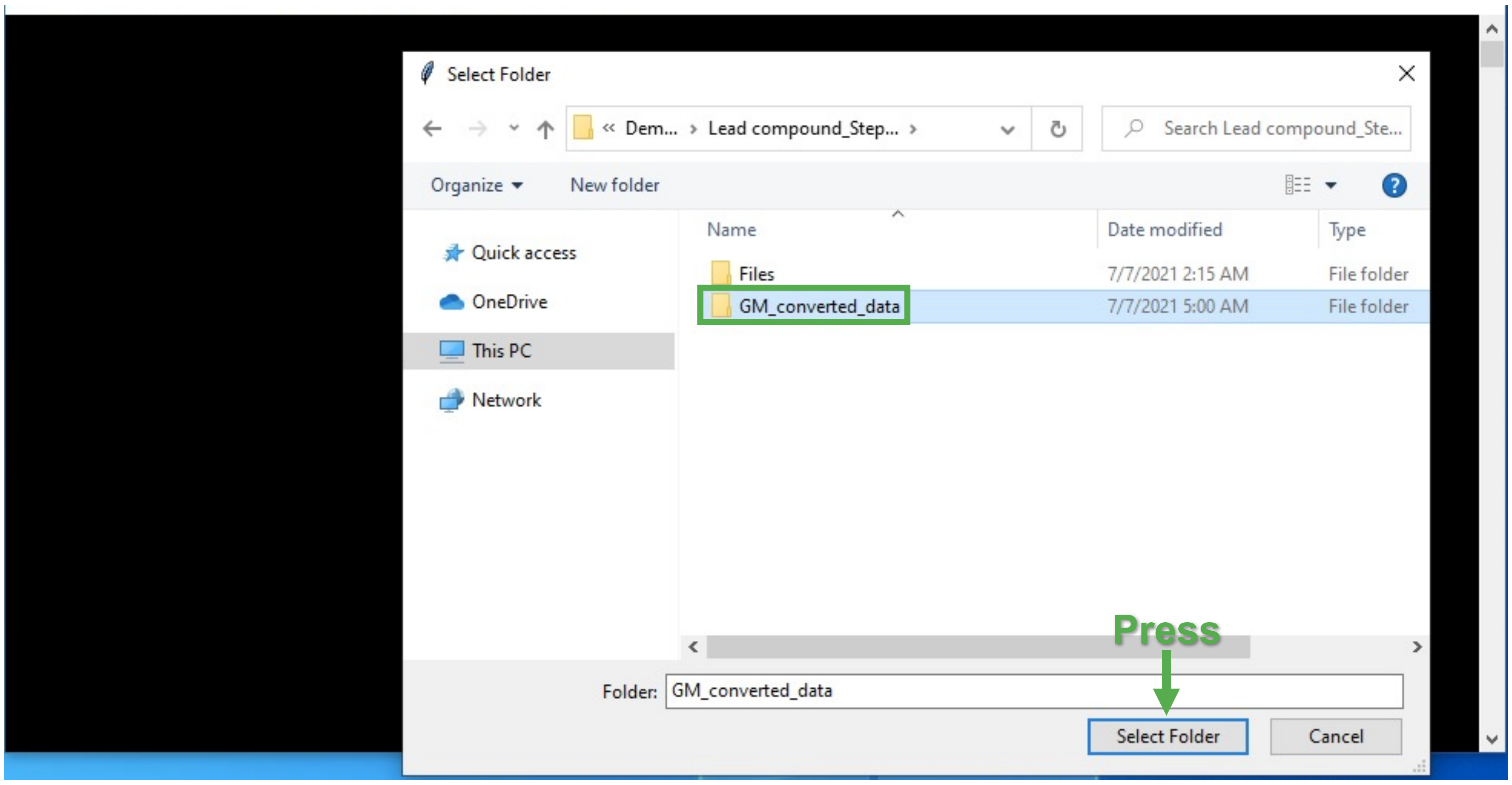

Select the folder “GM_converted_data” (Directory: Additional file 5 > Demo_2_Lead compound > Lead compound_Step-3_Validation > GM_converted_data) (green frame) and press “Select Folder” (indicated by a green arrow)

✱ The GUI of GM_Basic

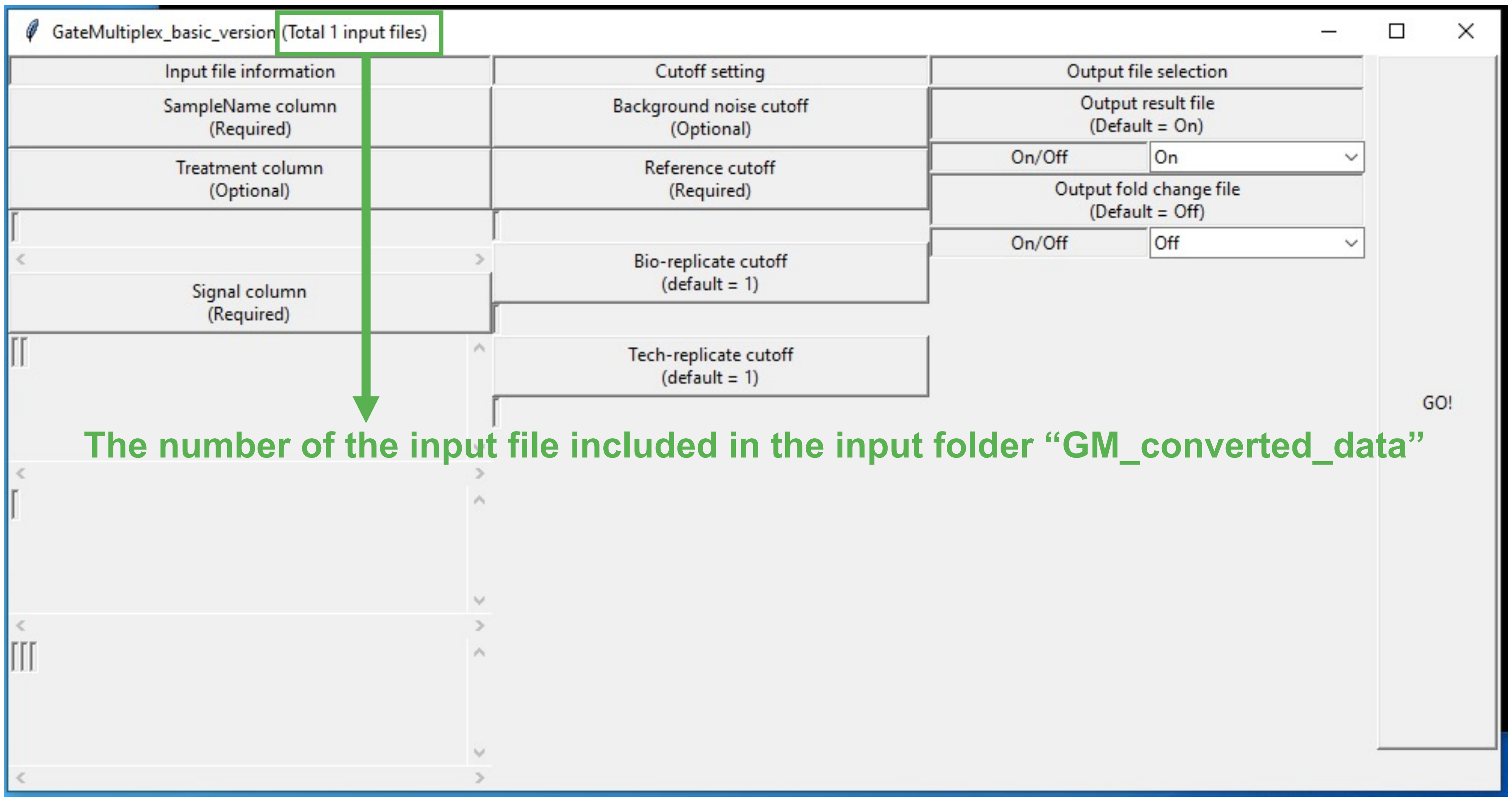

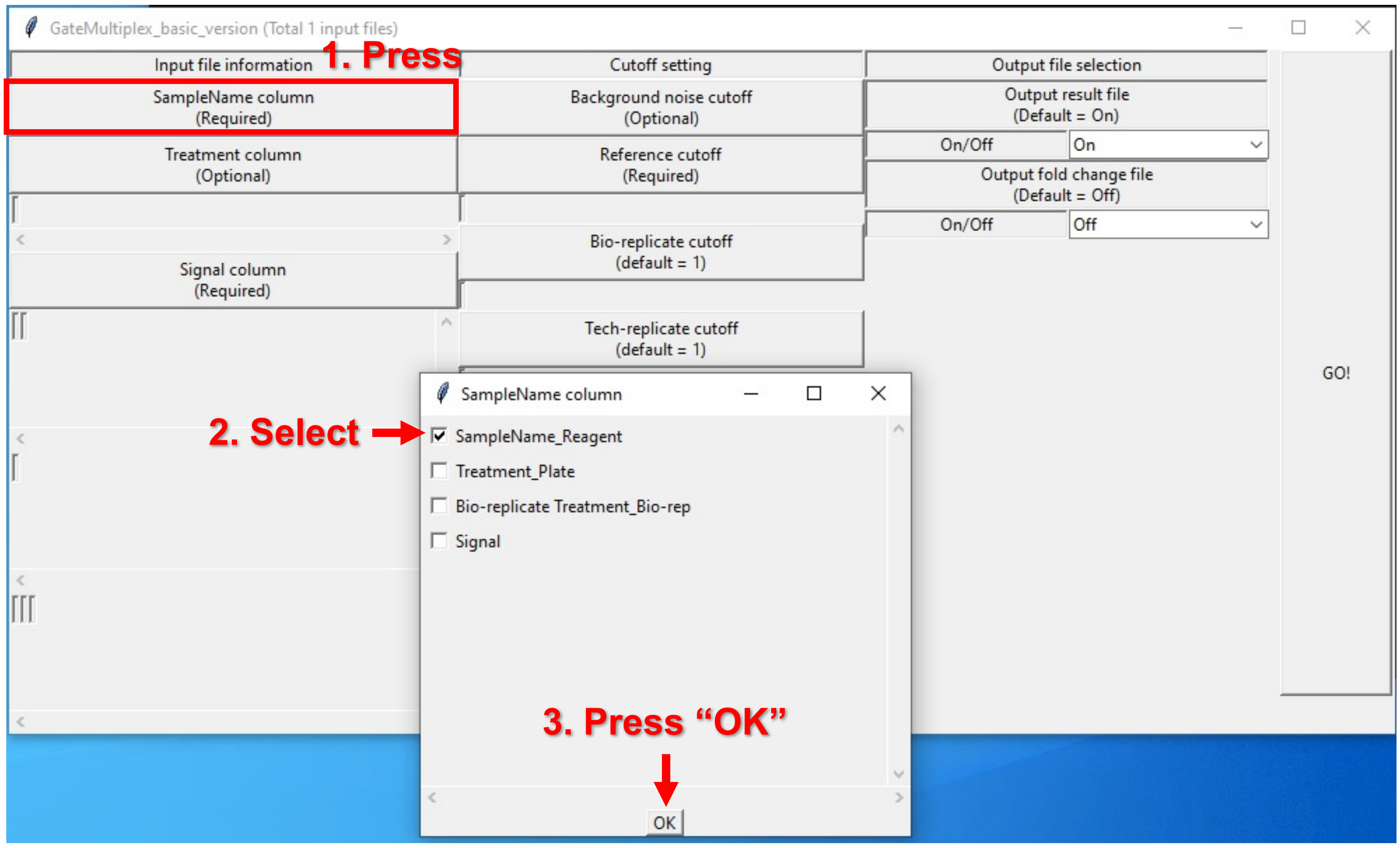

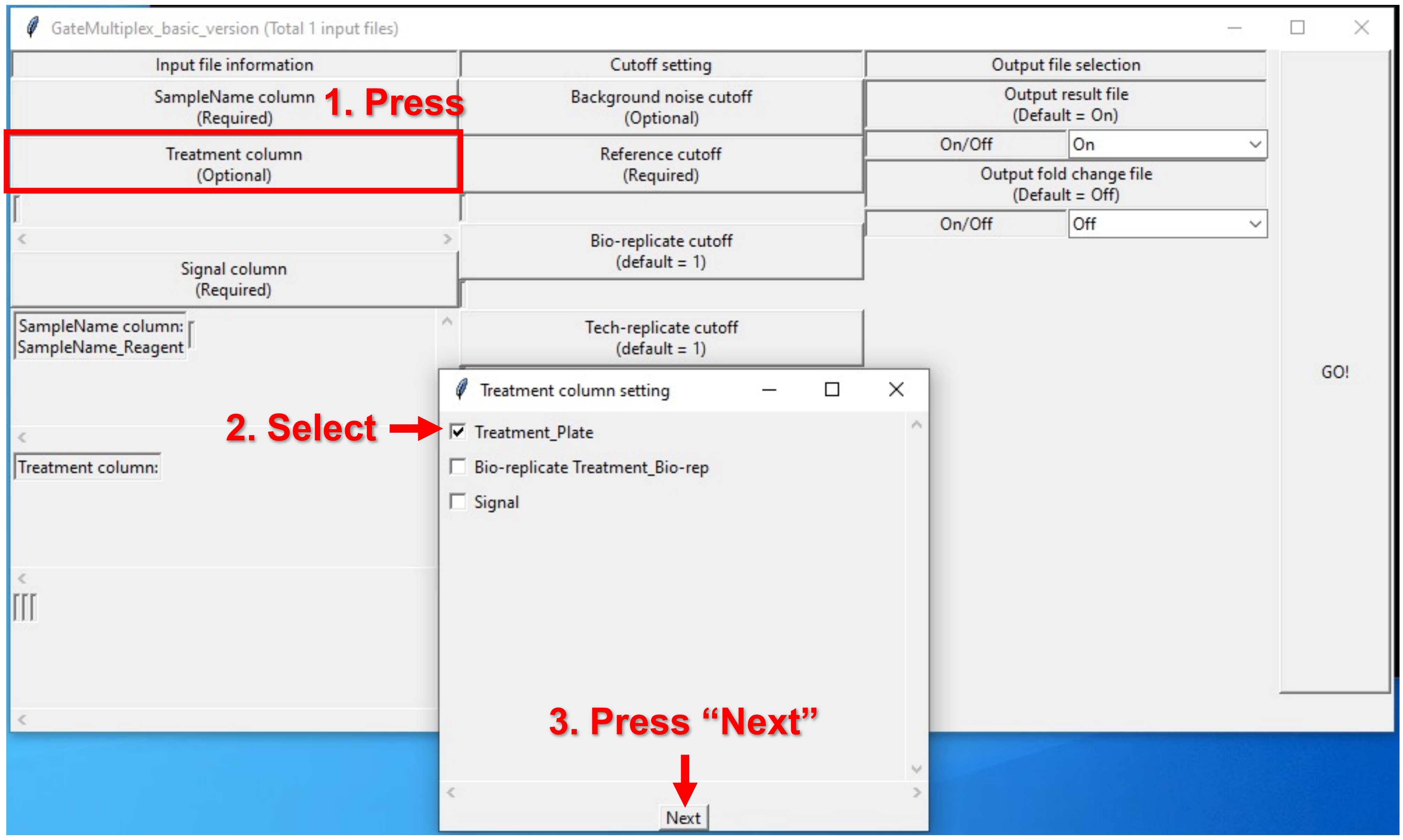
GateMultiplex_basic_version (Total 1 input files)
Input file information
Cutoff setting
Output file selection
SampleName column
(Required)
1. Press
Background noise cutoff
(Optional)
Output result file
(Default = On)
On/Off
On
Treatment column
(Optional)
Reference cutoff
(Required)
Output fold change file
(Default = Off)
On/Off
Off
Bio-replicate cutoff
(default = 1)
Signal column
(Required)
SampleName column:
SampleName_Reagent
Tech-replicate cutoff
(default = 1)
GO!
Treatment column setting
2. Select
Treatment_Plate
Bio-replicate Treatment_Bio-rep
Signal
Treatment column:
3. Press "Next"
Next

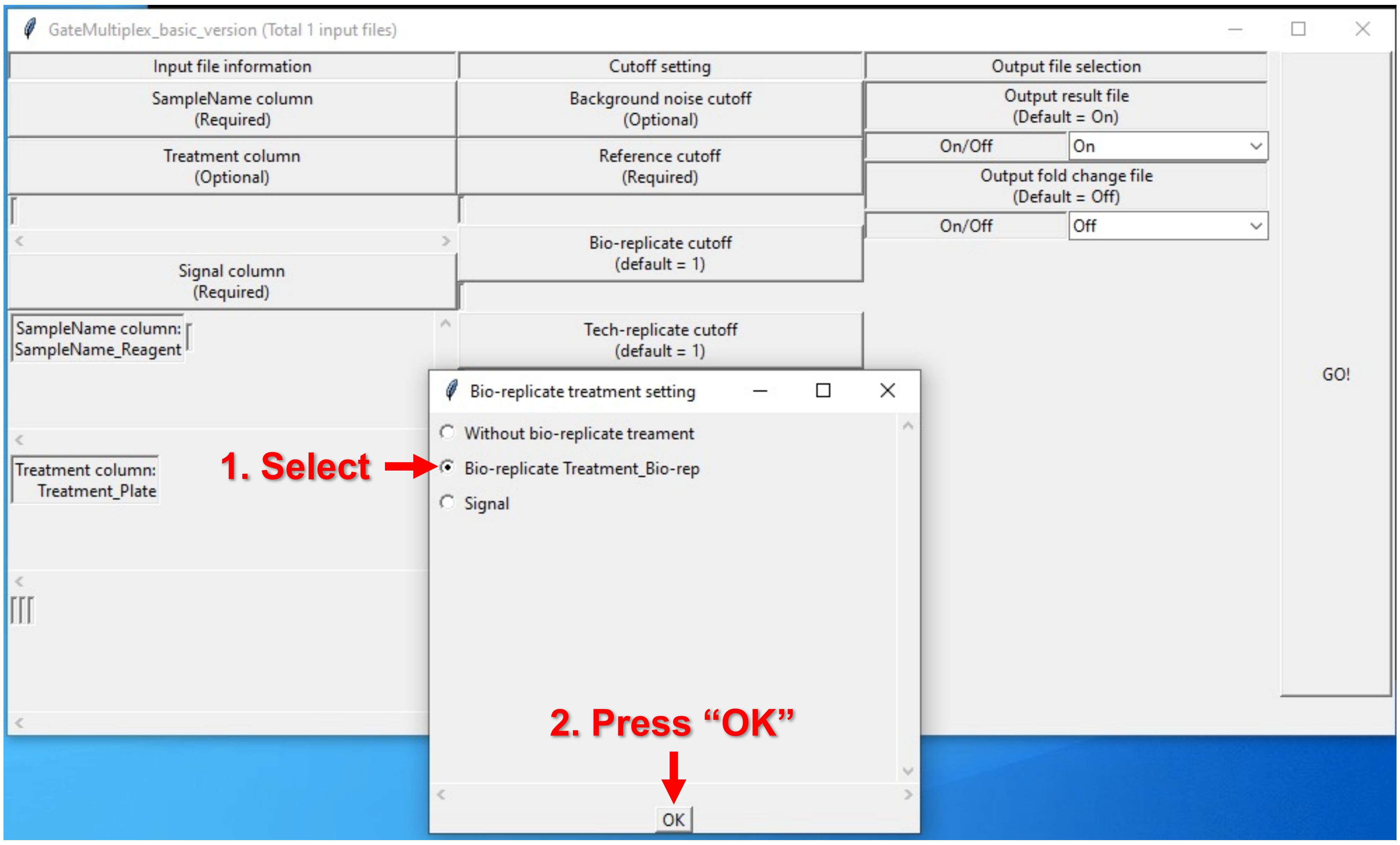
GateMultiplex_basic_version (Total 1 input files)
Input file information
Cutoff setting
Output file selection
SampleName column
(Required)
Background noise cutoff
(Optional)
Output result file
(Default = On)
On/Off
On
Treatment column
(Optional)
Reference cutoff
(Required)
Output fold change file
(Default = Off)
On/Off
Off
Bio-replicate cutoff
(default = 1)
Signal column
(Required)
SampleName column:
SampleName_Reagent
Tech-replicate cutoff
(default = 1)
GO!
Bio-replicate treatment setting
Without bio-replicate treament
1. Select
Bio-replicate Treatment_Bio-rep
Signal
Treatment column:
Treatment_Plate
2. Press "OK"
OK

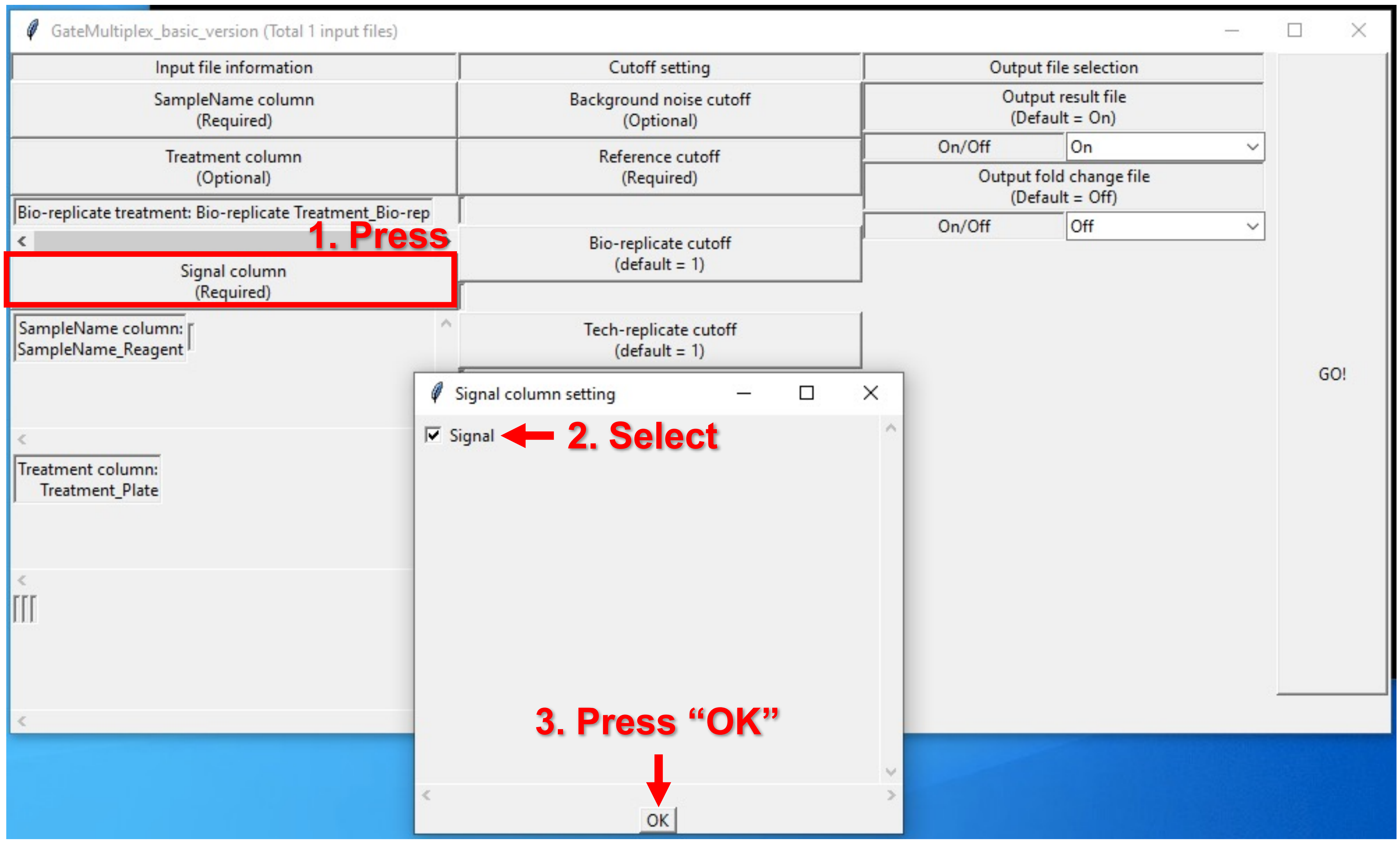
GateMultiplex_basic_version (Total 1 input files)
Input file information
SampleName column (Required)
Treatment column (Optional)
Bio-replicate treatment: Bio-replicate Treatment_Bio-rep
1. Press
Signal column (Required)
SampleName column: SampleName_Reagent
Treatment column: Treatment_Plate
Cutoff setting
Background noise cutoff (Optional)
Reference cutoff (Required)
Bio-replicate cutoff (default = 1)
Tech-replicate cutoff (default = 1)
Signal column setting
Signal
2. Select
3. Press "OK"
OK
Output file selection
Output result file (Default = On)
On/Off
On
Output fold change file (Default = Off)
On/Off
Off
GO!

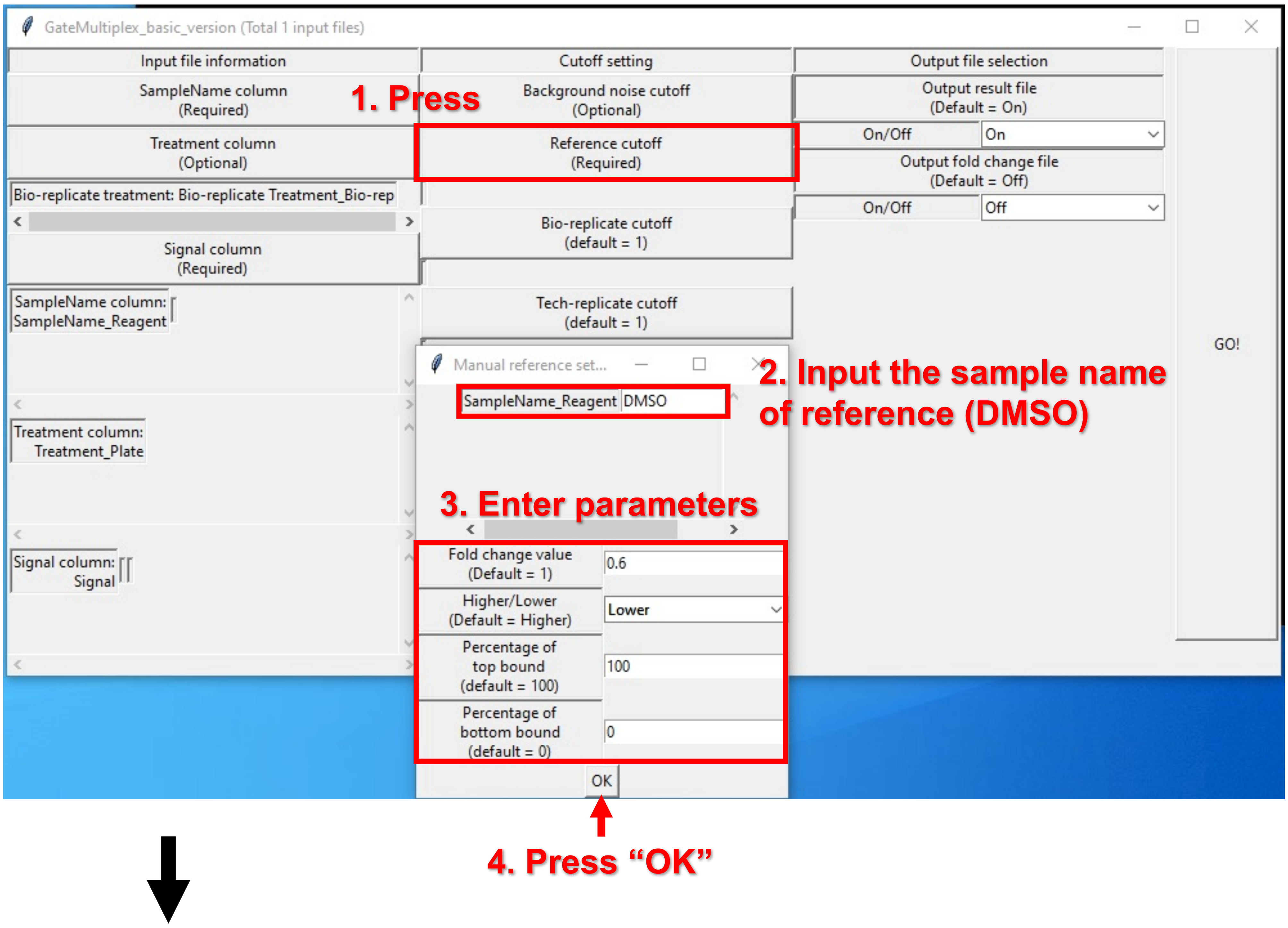
GateMultiplex_basic_version (Total 1 input files)
Input file information
SampleName column (Required)
Treatment column (Optional)
Bio-replicate treatment: Bio-replicate Treatment_Bio-rep
Signal column (Required)
SampleName column: SampleName_Reagent
Treatment column: Treatment_Plate
Signal column: Signal
Cutoff setting
1. Press
Background noise cutoff (Optional)
Reference cutoff (Required)
Bio-replicate cutoff (default = 1)
Tech-replicate cutoff (default = 1)
Manual reference set...
SampleName_Reagent
DMSO
2. Input the sample name of reference (DMSO)
3. Enter parameters
Fold change value (Default = 1)
0.6
Higher/Lower (Default = Higher)
Lower
Percentage of top bound (default = 100)
100
Percentage of bottom bound (default = 0)
0
OK
4. Press "OK"
Output file selection
Output result file (Default = On)
On/Off
On
Output fold change file (Default = Off)
On/Off
Off
GO!

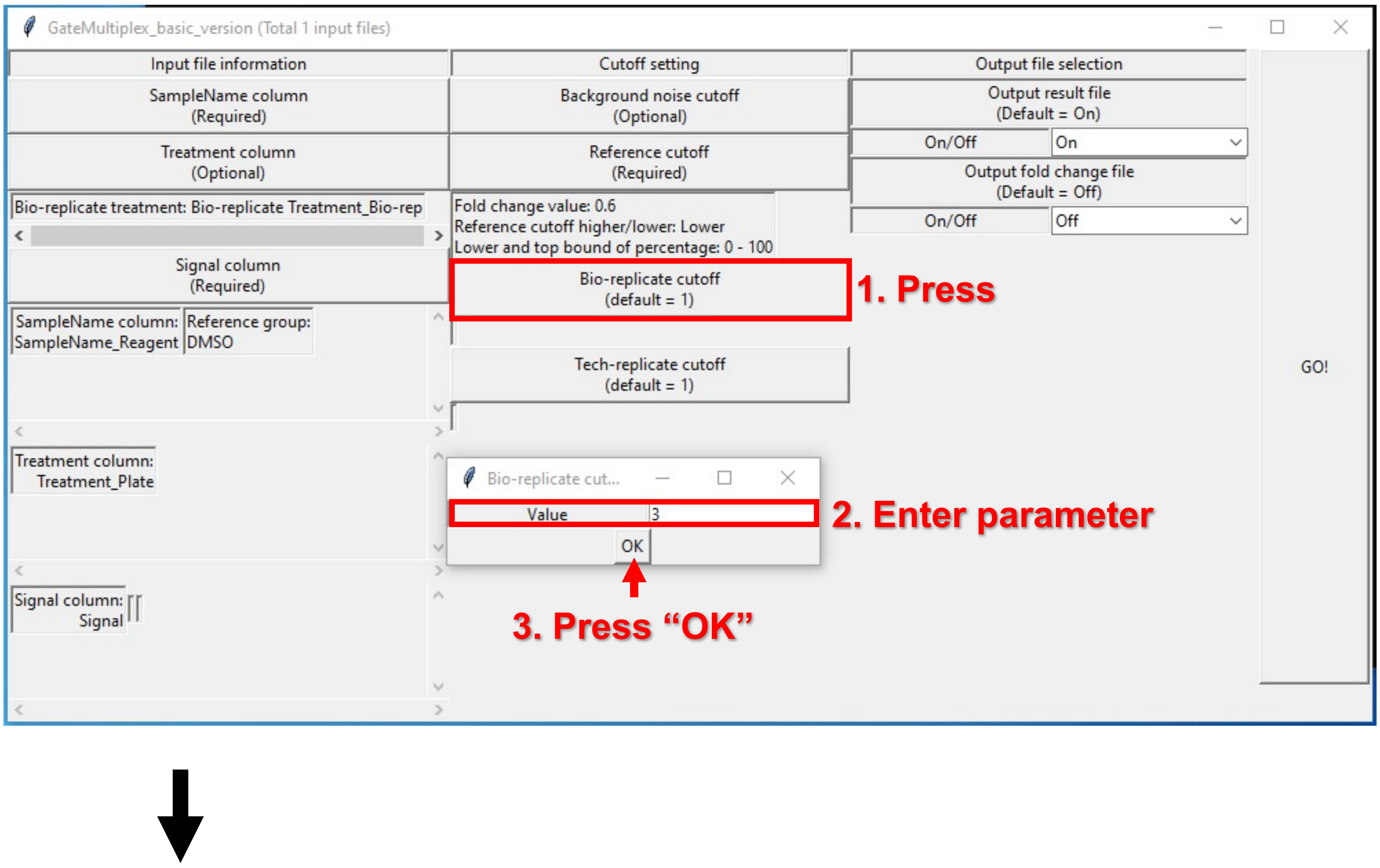
GateMultiplex_basic_version (Total 1 input files)
Input file information
SampleName column (Required)
Treatment column (Optional)
Bio-replicate treatment: Bio-replicate Treatment_Bio-rep
Signal column (Required)
SampleName column: SampleName_Reagent
Reference group: DMSO
Treatment column: Treatment_Plate
Signal column: Signal
Cutoff setting
Background noise cutoff (Optional)
Reference cutoff (Required)
Fold change value: 0.6
Reference cutoff higher/lower: Lower
Lower and top bound of percentage: 0 - 100
Bio-replicate cutoff (default = 1)
Tech-replicate cutoff (default = 1)
Output file selection
Output result file (Default = On)
On/Off
On
Output fold change file (Default = Off)
On/Off
Off
GO!
Bio-replicate cut...
Value
3
OK
1. Press
2. Enter parameter
3. Press "OK"

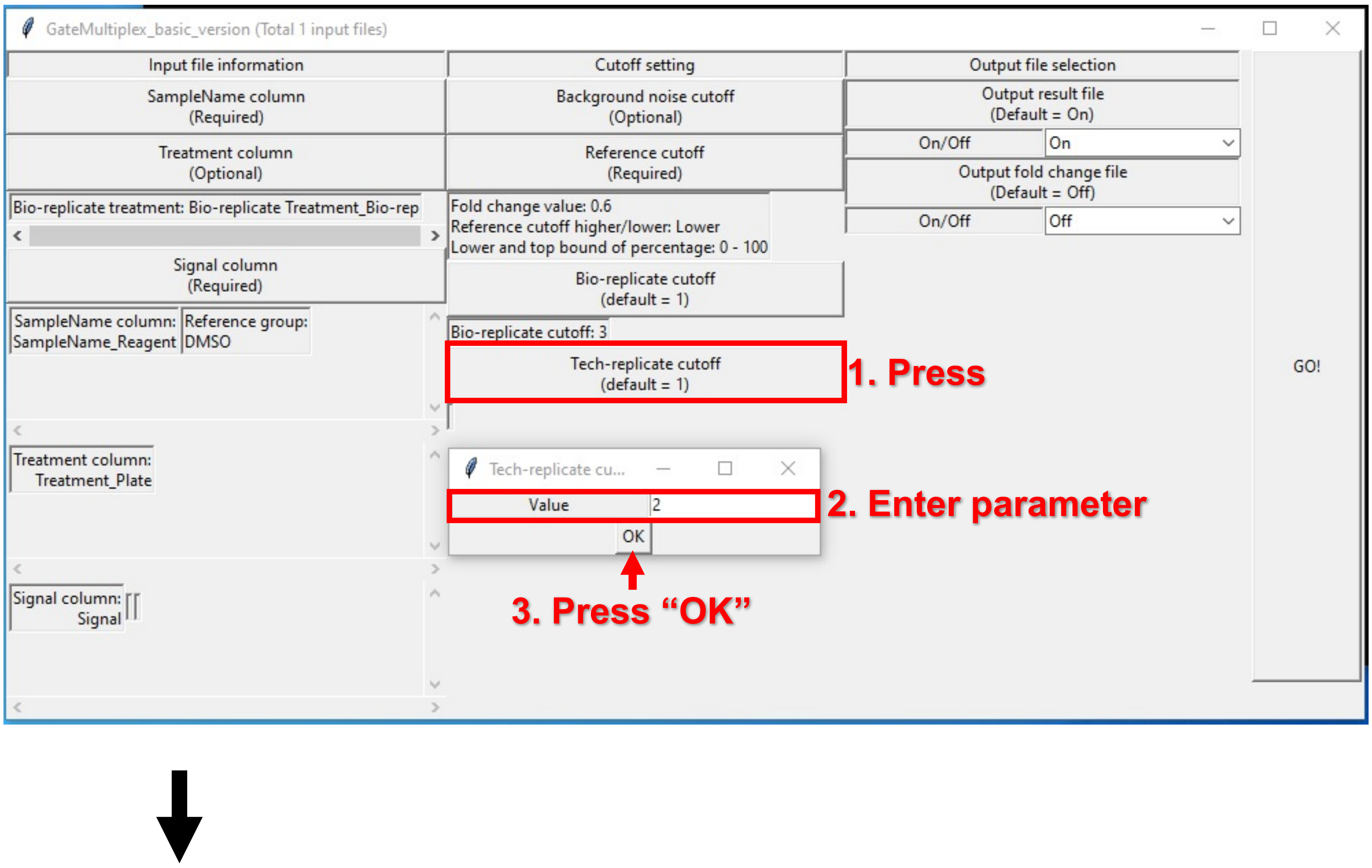
GateMultiplex_basic_version (Total 1 input files)
Input file information
SampleName column (Required)
Treatment column (Optional)
Bio-replicate treatment: Bio-replicate Treatment_Bio-rep
Signal column (Required)
SampleName column: SampleName_Reagent
Reference group: DMSO
Treatment column: Treatment_Plate
Signal column: Signal
Cutoff setting
Background noise cutoff (Optional)
Reference cutoff (Required)
Fold change value: 0.6
Reference cutoff higher/lower: Lower
Lower and top bound of percentage: 0 - 100
Bio-replicate cutoff (default = 1)
Bio-replicate cutoff: 3
Tech-replicate cutoff (default = 1)
Output file selection
Output result file (Default = On)
On/Off
On
Output fold change file (Default = Off)
On/Off
Off
GO!
Tech-replicate cu...
Value
2
OK
1. Press
2. Enter parameter
3. Press "OK"

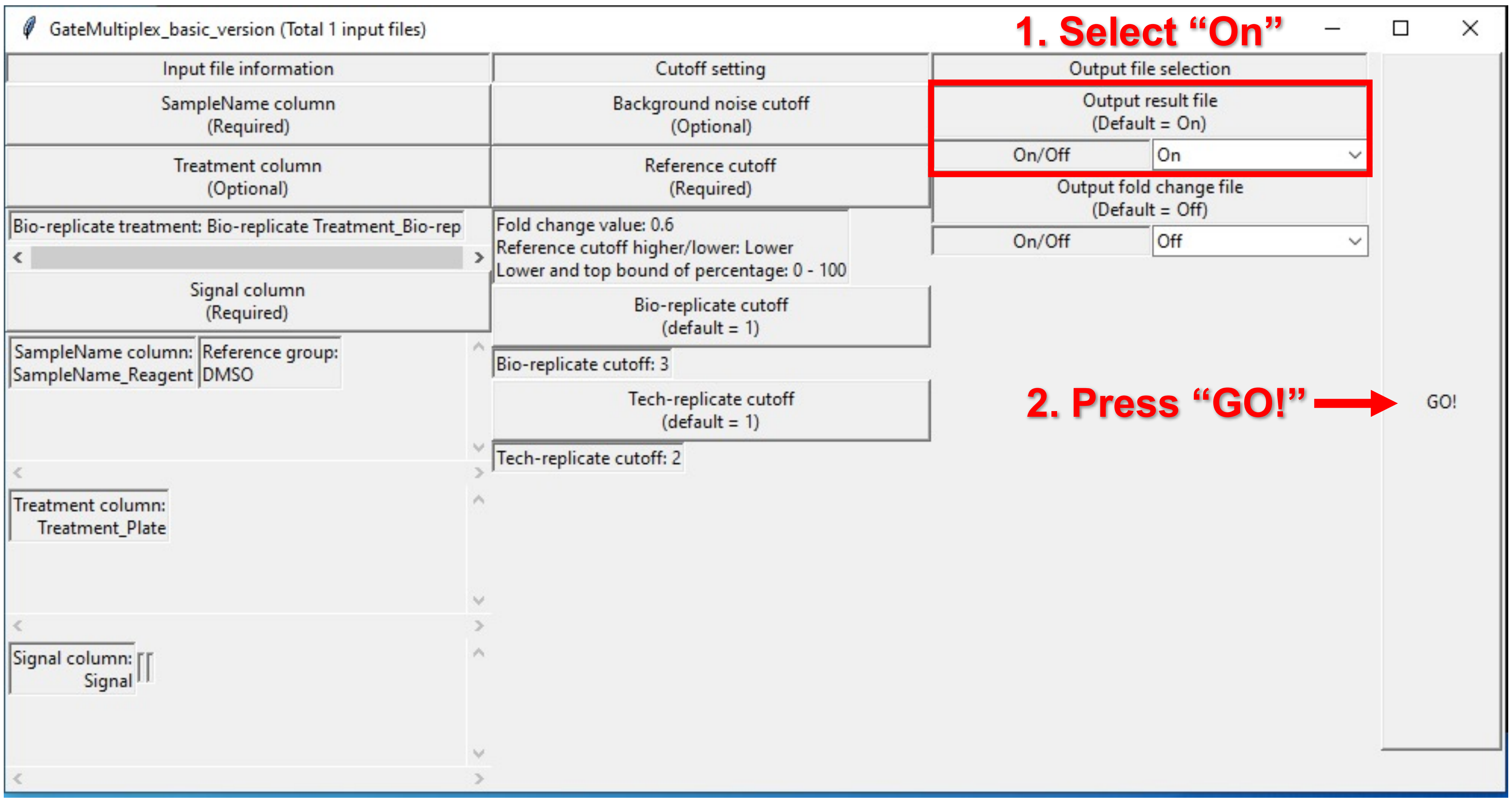

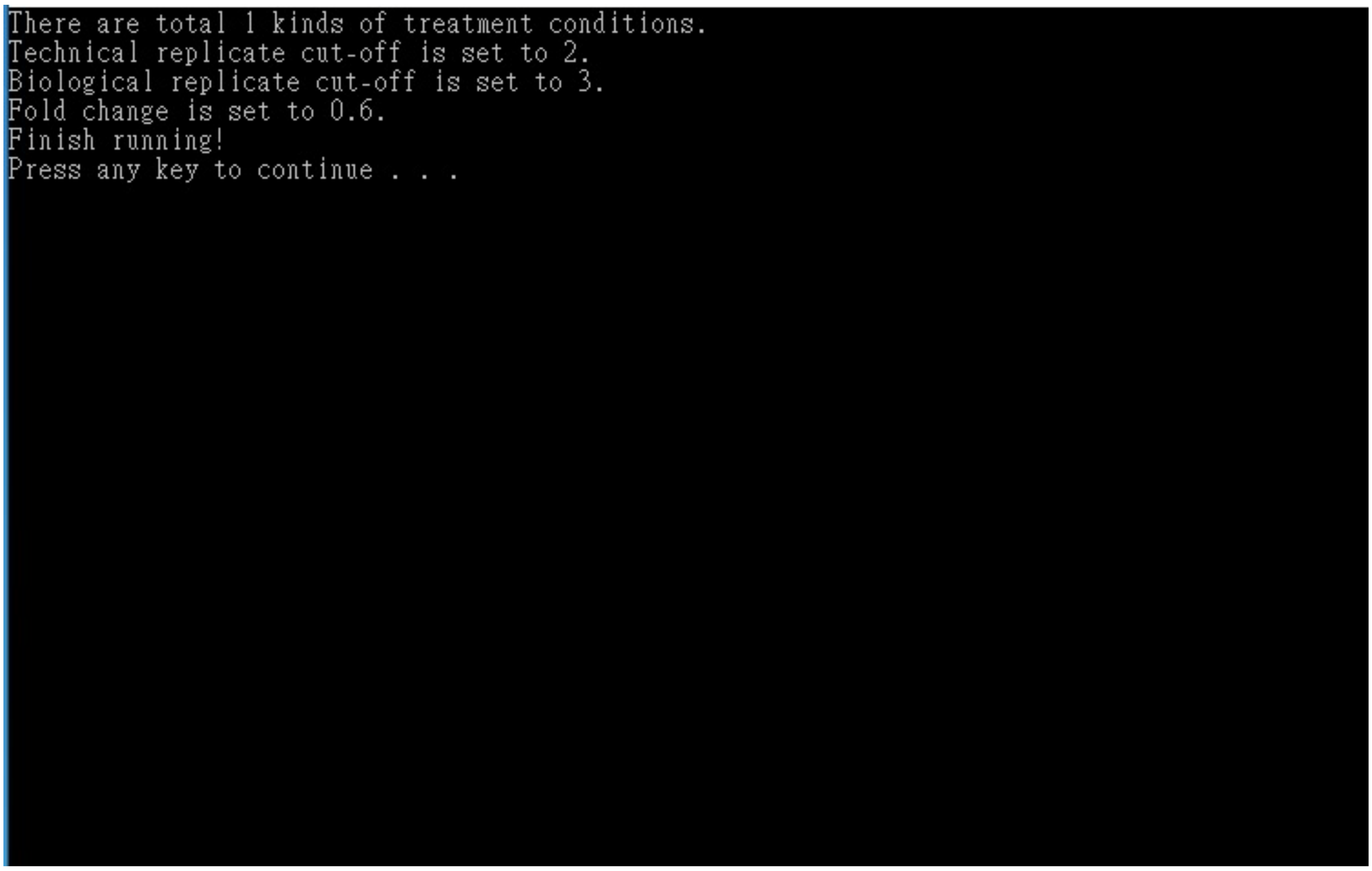

Press any key to close the window

- The result file, named “Results.csv”, (red frame) is stored in the same folder with executive files of GM_Basic.

- The symbol “N” means negative and the symbol “P” means positive. Please see the “Symbols in output files” section in Additional file 3.

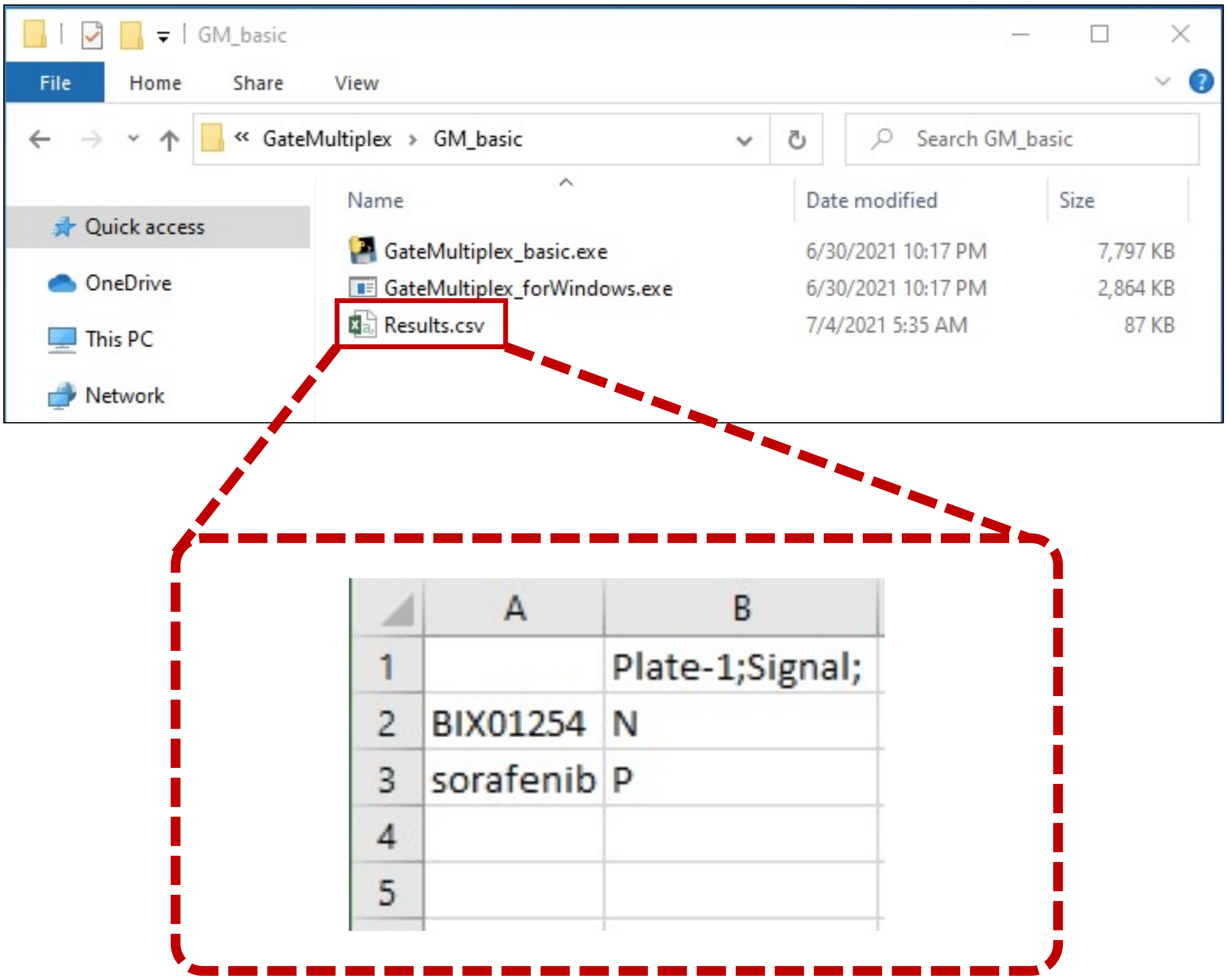

Phenomic screening in precision agriculture (GM_Basic)

Phenomic screening in precision agriculture (GM_Basic)

- The location of precision agriculture demo data.

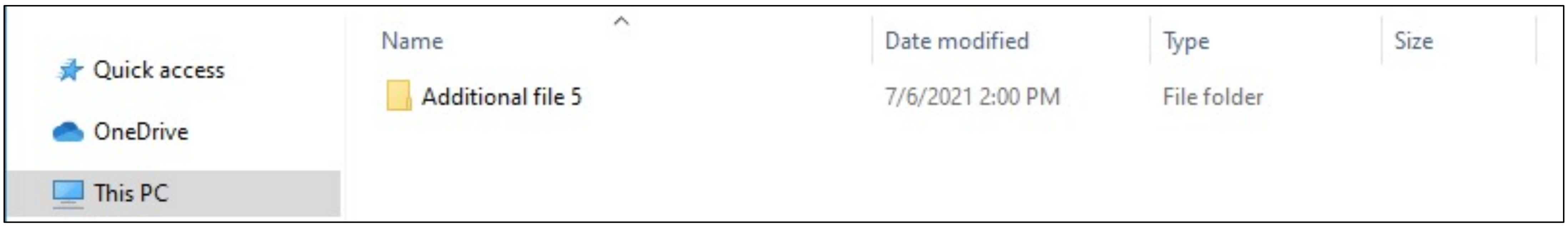

Open the "Additional file 5" folder

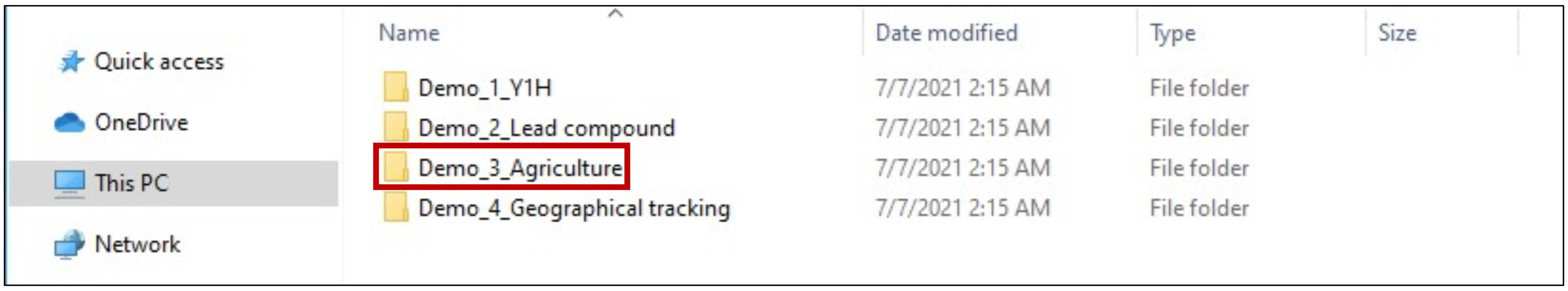

Open the "Demo_3_Agriculture" folder (red frame)

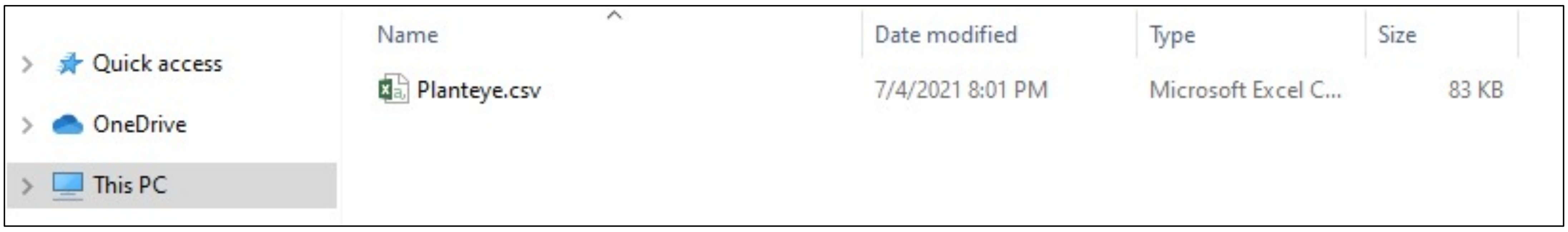

*The "Planteye.csv" file is the demo data for precision agriculture.

- The content of “Planteye.csv” (partially shown).

Plant line	Date	Time	Digital biomass	Greenness	Plant height	Hue	Leaf angle	Leaf area	Leaf area index
10-01-01 AM	2019_6_8	11-43-43 AM	1738210	0.34924	211.561	105.523	56.73	8216.09	0.051
10-01-01 AM	2019_6_9	11-43-47 AM	2240160	0.32839	225.05	106.143	51.481	9954.03	0.062
10-01-01 AM	2019_6_10	11-43-51 AM	3297400	0.37061	247.013	104.418	58.586	13349.1	0.083
10-01-01 AM	2019_6_11	11-43-55 AM	4186860	0.33275	275.108	106.928	56.453	15219	0.095
10-01-01 AM	2019_6_12	11-43-58 AM	4261940	0.37419	292.934	103.121	55.414	14549.2	0.091
10-01-01 AM	2019_6_13	11-44-02 AM	5245310	0.3362	314.395	105.517	59.179	16683.8	0.104
10-01-01 AM	2019_6_14	11-44-06 AM	6125460	0.3374	344.901	104.755	60.58	17760.1	0.111
10-01-01 AM	2019_6_15	11-44-09 AM	6791060	0.3379	341.243	103.708	57.448	19901	0.124
10-01-01 AM	2019_6_16	11-44-14 AM	8113610	0.35269	423.715	102.996	53.869	19148.7	0.12

*The information title of each column is circled by red frame.

*The data of each column is circled by a blue frame.

❖ Line selection (GM_Basic)

- The two executive files of GM_Basic (GateMultiplex_basic.exe and GateMultiplex_forWindows.exe) are both stored in the “GM_basic” folder (blue frame) and should be placed in the same folder for operation.
- Please see the Fig. S14 and S15 in Additional file 1 for the cutoff setting.

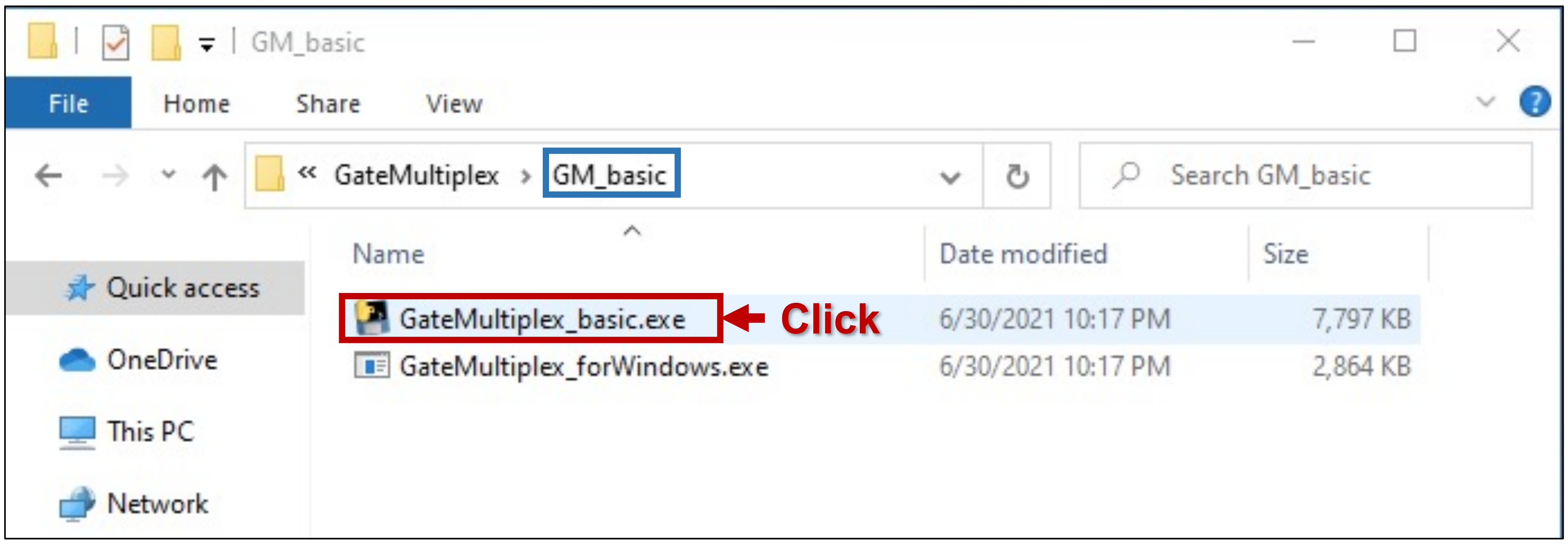

Activate the GM_basic by a double-clicking on “GateMultiplex_basic.exe” (red frame)

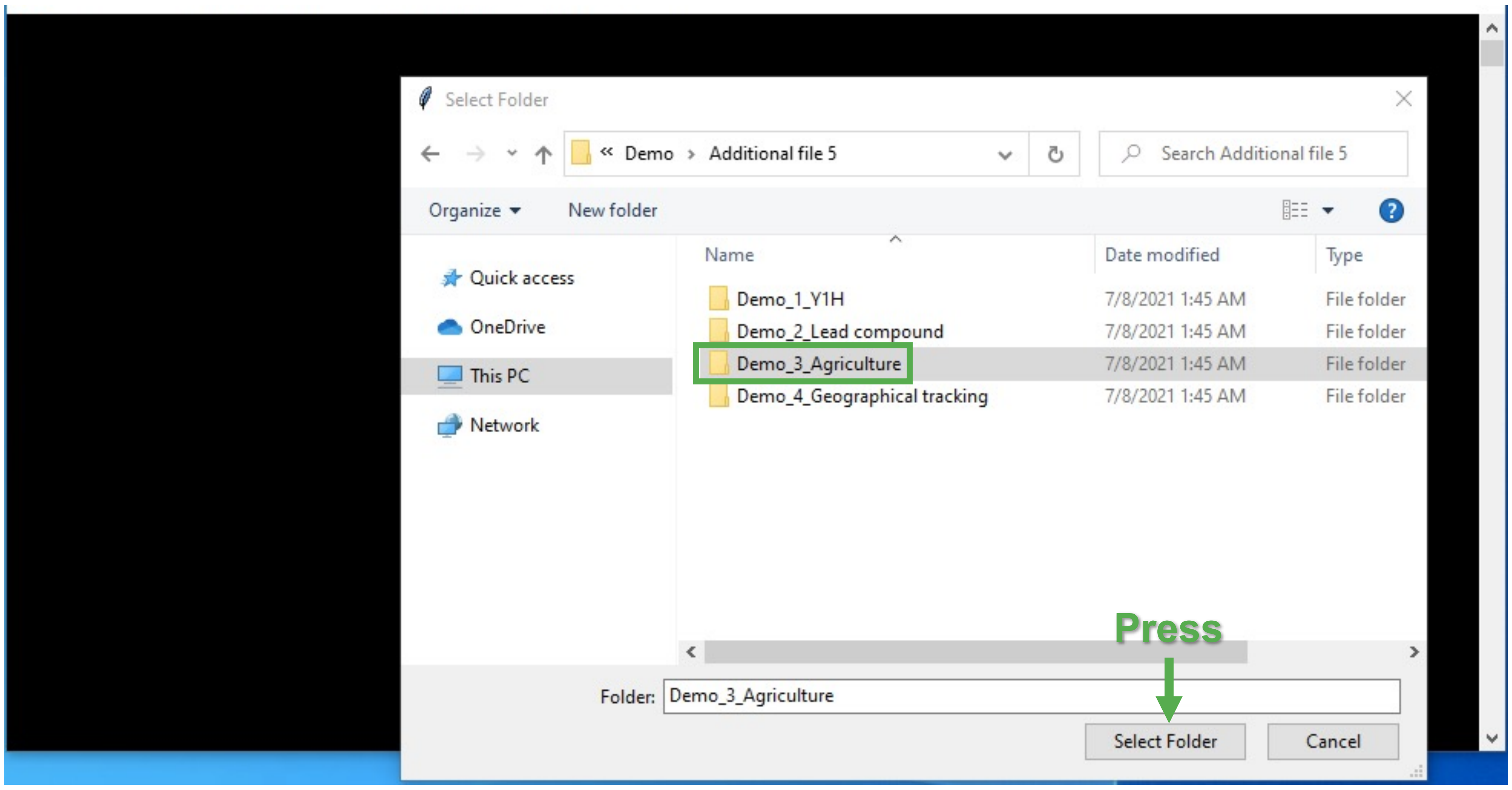

Select the folder "Demo_3_Agriculture" (Additional file 5 > Demo_3_Agriculture) (green frame) and press "Select Folder" (indicated by a green arrow)

✱ The GUI of GM_Basic

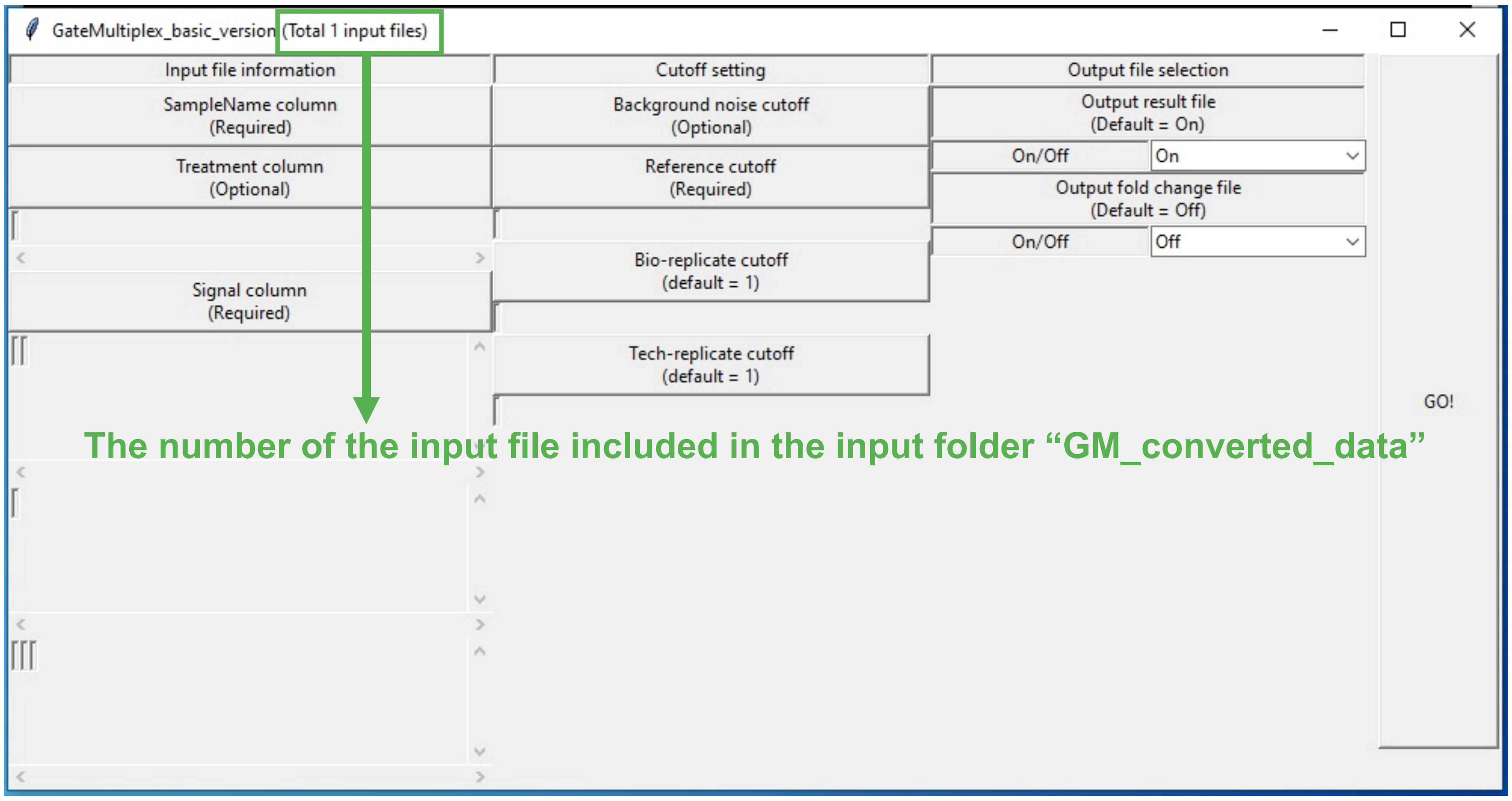

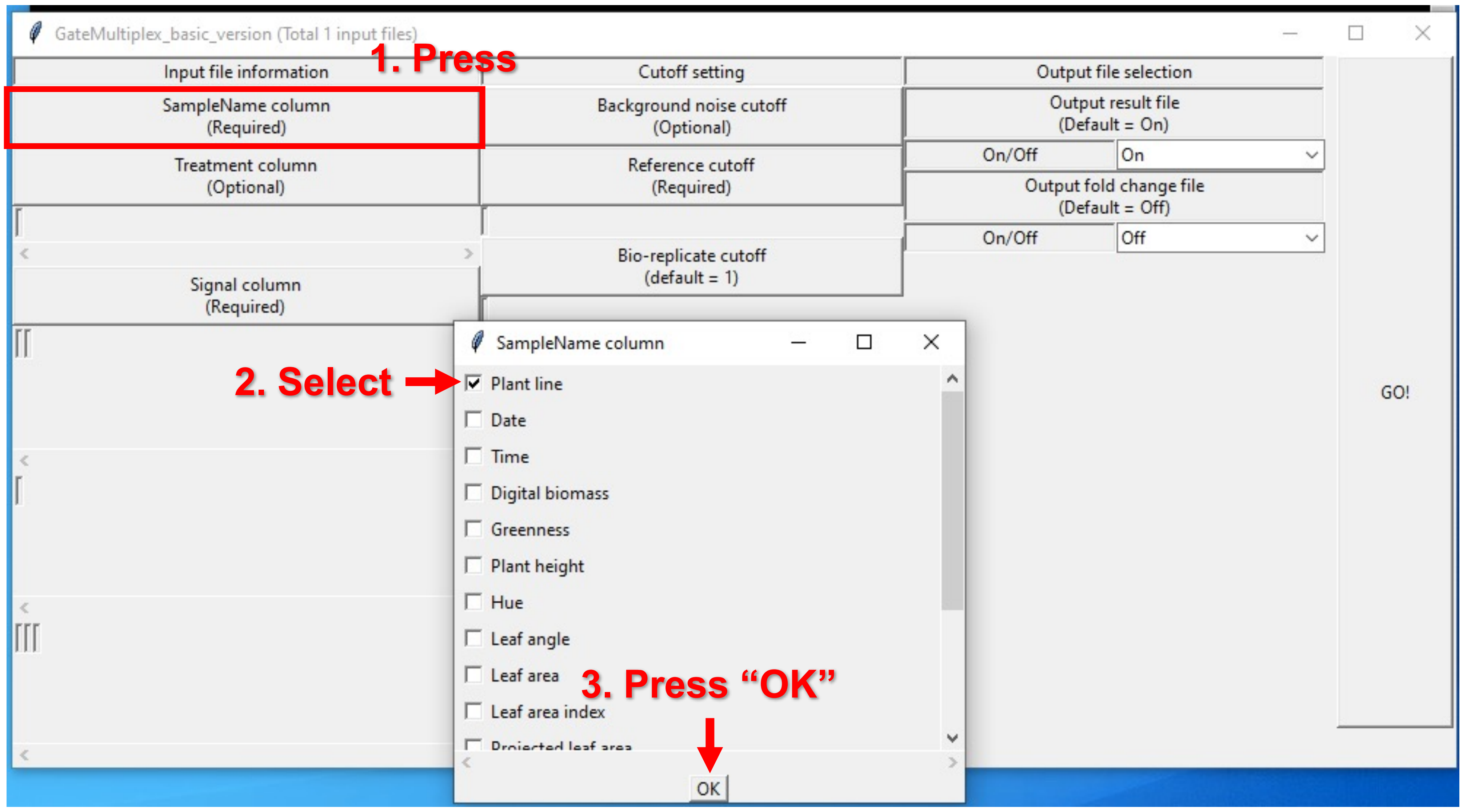
GateMultiplex_basic_version (Total 1 input files)
1. Press
Input file information
SampleName column
(Required)
Treatment column
(Optional)
Signal column
(Required)
Cutoff setting
Background noise cutoff
(Optional)
Reference cutoff
(Required)
Bio-replicate cutoff
(default = 1)
Output file selection
Output result file
(Default = On)
On/Off
On
Output fold change file
(Default = Off)
On/Off
Off
GO!
SampleName column
2. Select
Plant line
Date
Time
Digital biomass
Greenness
Plant height
Hue
Leaf angle
Leaf area
Leaf area index
3. Press "OK"
OK

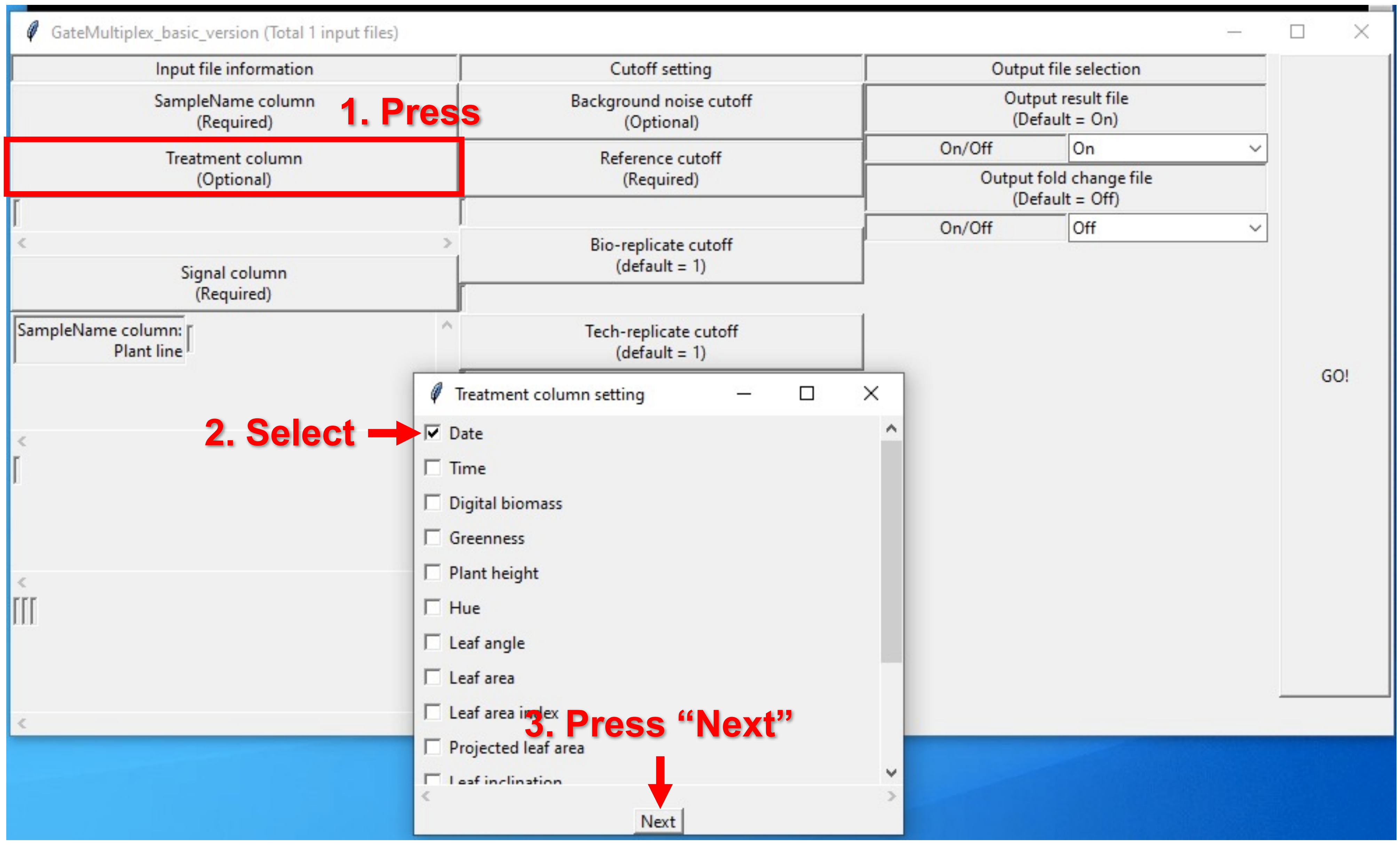
GateMultiplex_basic_version (Total 1 input files)
Input file information
SampleName column
(Required)
1. Press
Treatment column
(Optional)
Signal column
(Required)
SampleName column:
Plant line
Cutoff setting
Background noise cutoff
(Optional)
Reference cutoff
(Required)
Bio-replicate cutoff
(default = 1)
Tech-replicate cutoff
(default = 1)
Output file selection
Output result file
(Default = On)
On/Off
On
Output fold change file
(Default = Off)
On/Off
Off
GO!
Treatment column setting
2. Select
Date
Time
Digital biomass
Greenness
Plant height
Hue
Leaf angle
Leaf area
Leaf area index
Projected leaf area
3. Press "Next"
Next

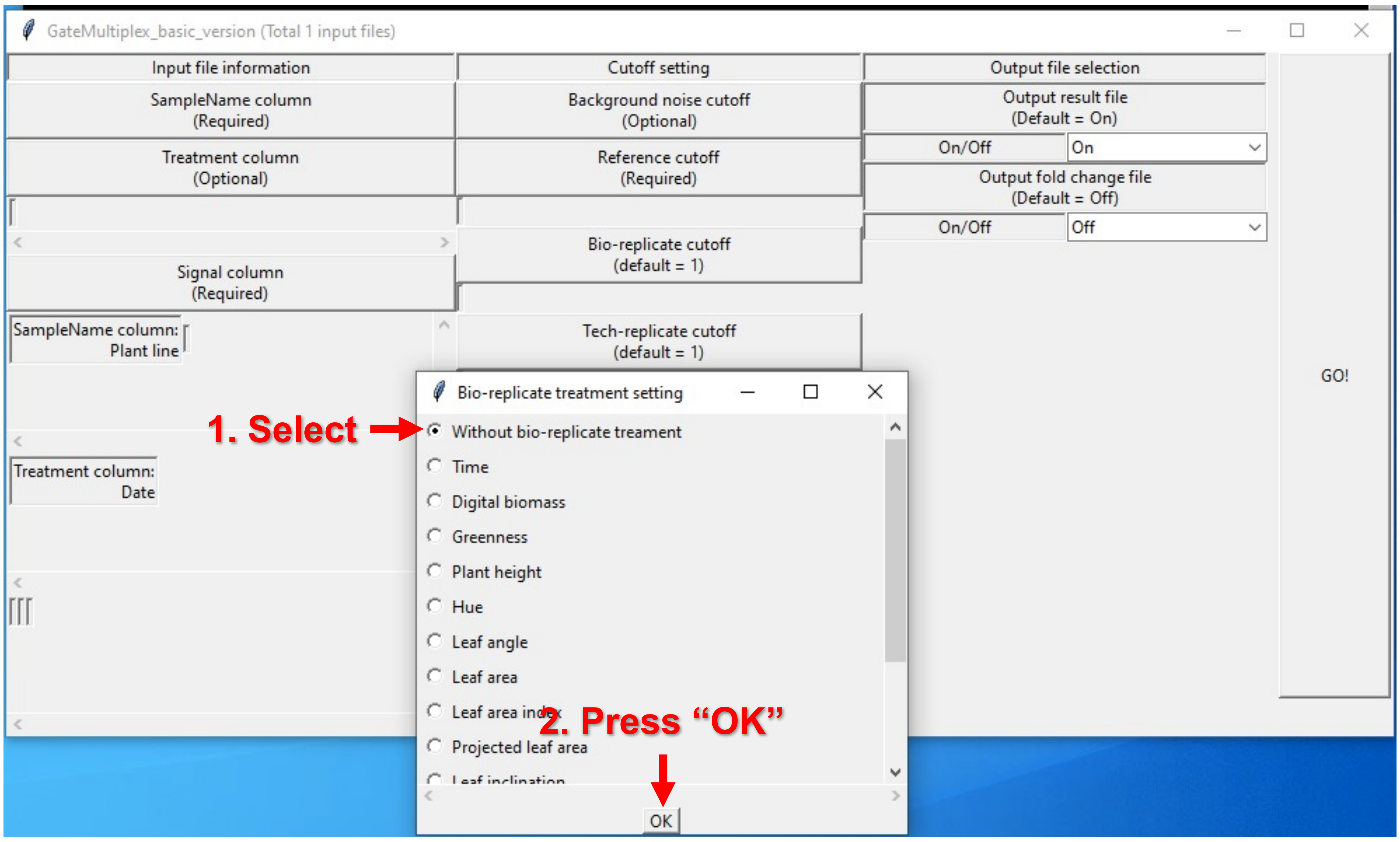
GateMultiplex_basic_version (Total 1 input files)
Input file information
SampleName column
(Required)
Treatment column
(Optional)
Signal column
(Required)
SampleName column:
Plant line
Treatment column:
Date
Cutoff setting
Background noise cutoff
(Optional)
Reference cutoff
(Required)
Bio-replicate cutoff
(default = 1)
Tech-replicate cutoff
(default = 1)
Output file selection
Output result file
(Default = On)
On/Off
On
Output fold change file
(Default = Off)
On/Off
Off
GO!
Bio-replicate treatment setting
1. Select
Without bio-replicate treament
Time
Digital biomass
Greenness
Plant height
Hue
Leaf angle
Leaf area
Leaf area index
Projected leaf area
2. Press "OK"
OK

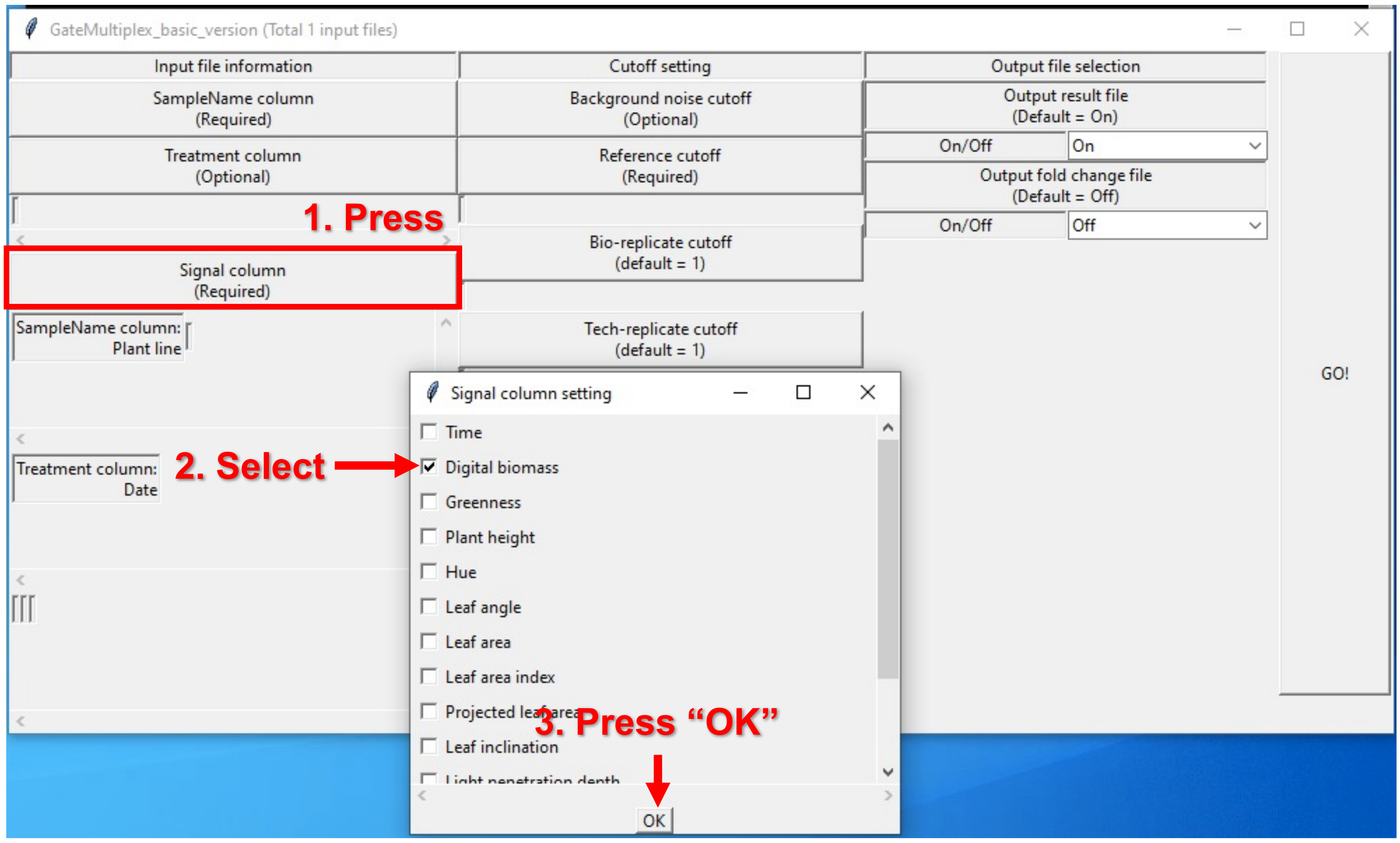
GateMultiplex_basic_version (Total 1 input files)
Input file information
SampleName column
(Required)
Treatment column
(Optional)
1. Press
Signal column
(Required)
SampleName column:
Plant line
Treatment column:
Date
Cutoff setting
Background noise cutoff
(Optional)
Reference cutoff
(Required)
Bio-replicate cutoff
(default = 1)
Tech-replicate cutoff
(default = 1)
Output file selection
Output result file
(Default = On)
On/Off
On
Output fold change file
(Default = Off)
On/Off
Off
GO!
Signal column setting
Time
2. Select
Digital biomass
Greenness
Plant height
Hue
Leaf angle
Leaf area
Leaf area index
Leaf inclination
3. Press "OK"
OK

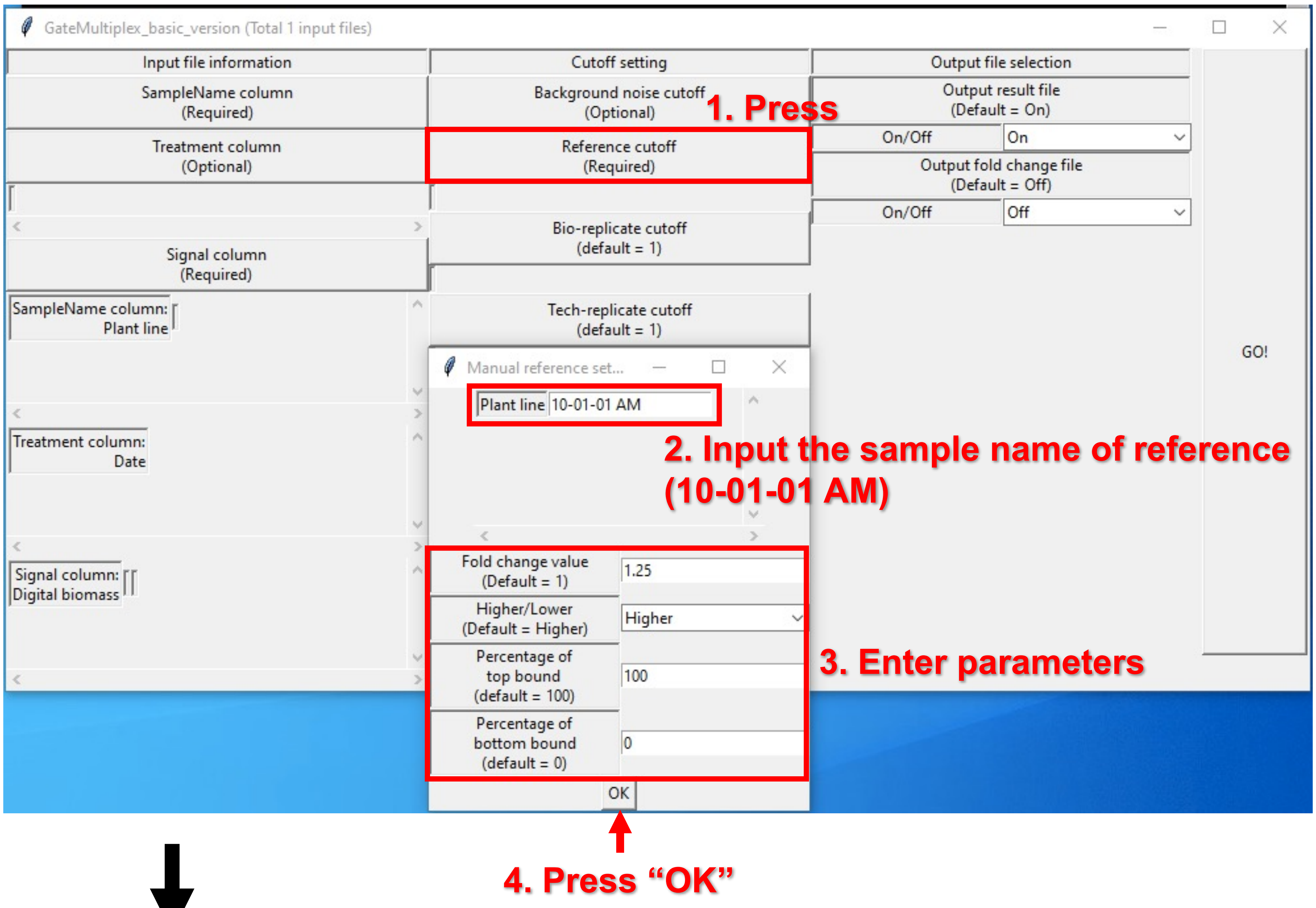
GateMultiplex_basic_version (Total 1 input files)
Input file information
SampleName column (Required)
Treatment column (Optional)
Signal column (Required)
SampleName column: Plant line
Treatment column: Date
Signal column: Digital biomass
Cutoff setting
Background noise cutoff (Optional)
1. Press
Reference cutoff (Required)
Bio-replicate cutoff (default = 1)
Tech-replicate cutoff (default = 1)
Manual reference set...
Plant line 10-01-01 AM
2. Input the sample name of reference (10-01-01 AM)
Fold change value (Default = 1)
1.25
Higher/Lower (Default = Higher)
Higher
Percentage of top bound (default = 100)
100
Percentage of bottom bound (default = 0)
0
OK
3. Enter parameters
4. Press "OK"
Output file selection
Output result file (Default = On)
On/Off
On
Output fold change file (Default = Off)
On/Off
Off
GO!

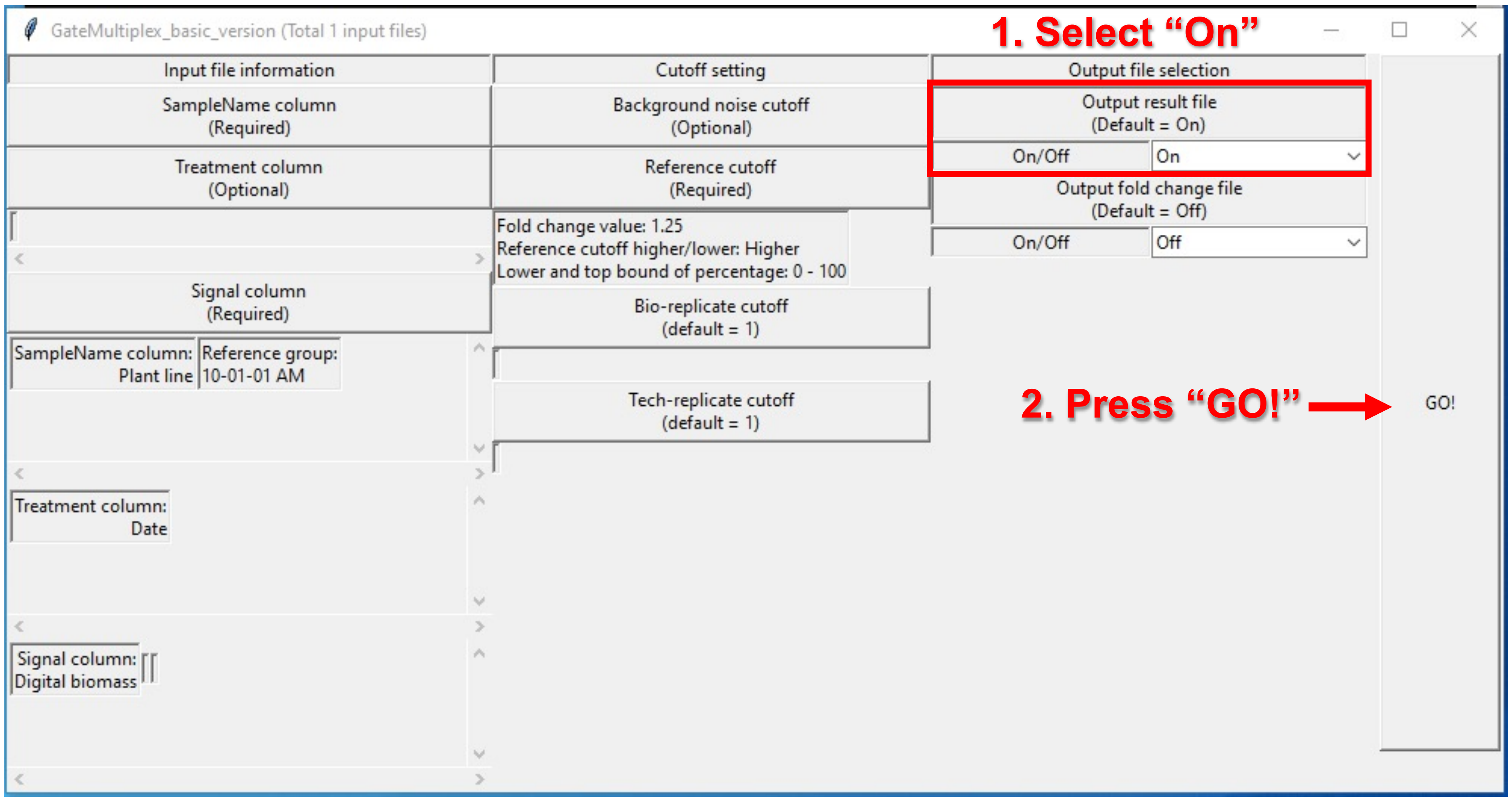
GateMultiplex_basic_version (Total 1 input files)
1. Select "On"
Input file information
SampleName column (Required)
Treatment column (Optional)
Signal column (Required)
SampleName column: Plant line
Reference group: 10-01-01 AM
Treatment column: Date
Signal column: Digital biomass
Cutoff setting
Background noise cutoff (Optional)
Reference cutoff (Required)
Fold change value: 1.25
Reference cutoff higher/lower: Higher
Lower and top bound of percentage: 0 - 100
Bio-replicate cutoff (default = 1)
Tech-replicate cutoff (default = 1)
Output file selection
Output result file (Default = On)
On/Off
On
Output fold change file (Default = Off)
On/Off
Off
2. Press "GO!"
GO!

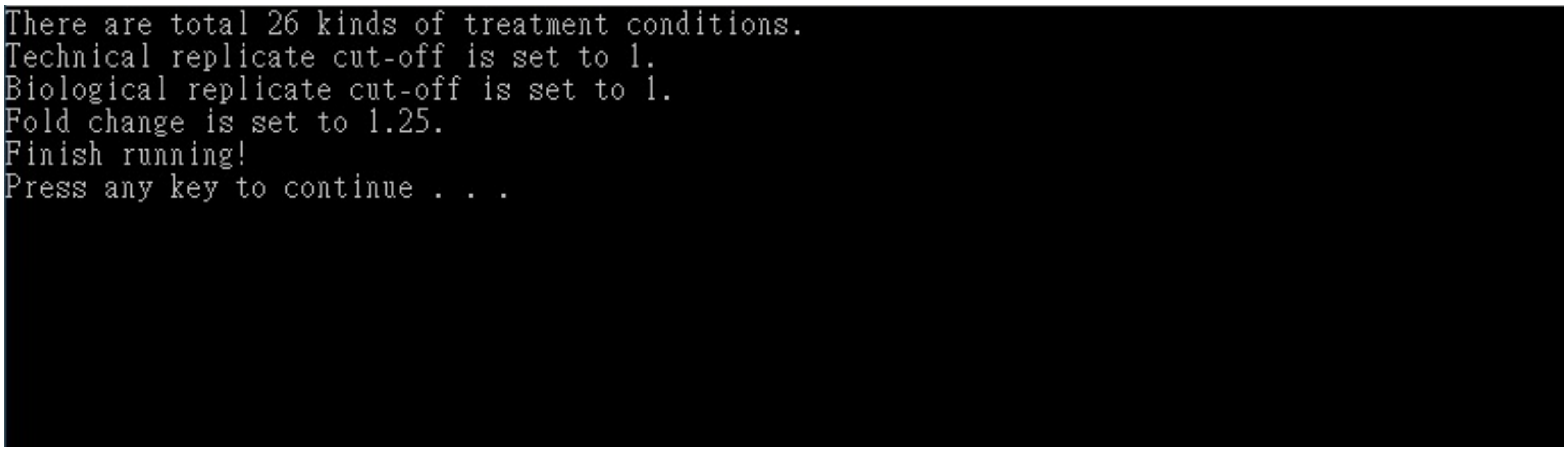

Press any key to close the window

- The result file, named "Results.csv" (red frame), is stored in the same folder with executive files of GM_Basic.
- Please see "Symbols in output files" section in Additional file 3 for the concept of P and N.

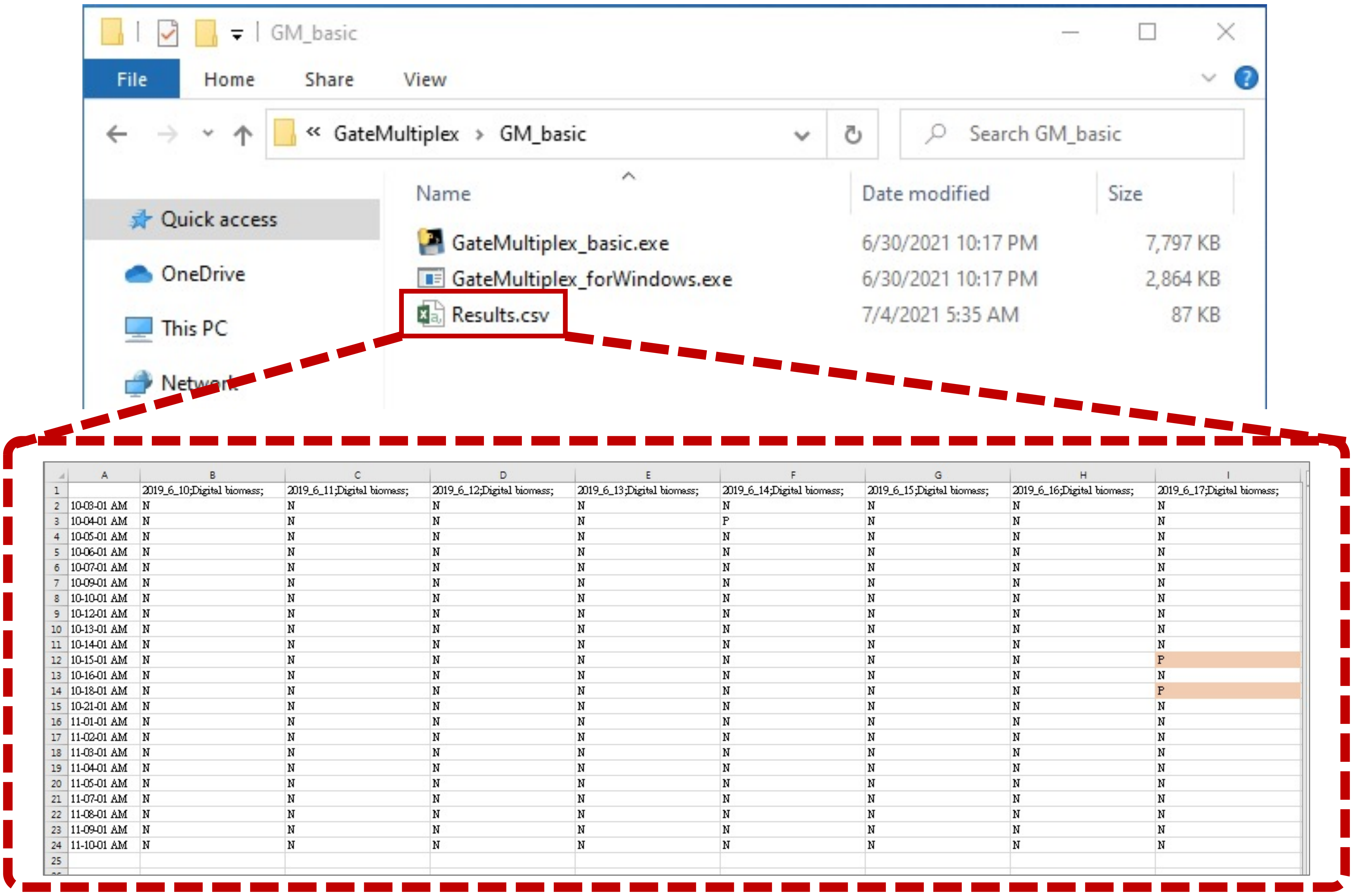

	A	B	C	D	E	F	G	H	I
1		2019_6_10;Digital biomass;	2019_6_11;Digital biomass;	2019_6_12;Digital biomass;	2019_6_13;Digital biomass;	2019_6_14;Digital biomass;	2019_6_15;Digital biomass;	2019_6_16;Digital biomass;	2019_6_17;Digital biomass;
2	10-03-01 AM	N	N	N	N	N	N	N	N
3	10-04-01 AM	N	N	N	N	P	N	N	N
4	10-05-01 AM	N	N	N	N	N	N	N	N
5	10-06-01 AM	N	N	N	N	N	N	N	N
6	10-07-01 AM	N	N	N	N	N	N	N	N
7	10-09-01 AM	N	N	N	N	N	N	N	N
8	10-10-01 AM	N	N	N	N	N	N	N	N
9	10-12-01 AM	N	N	N	N	N	N	N	N
10	10-13-01 AM	N	N	N	N	N	N	N	N
11	10-14-01 AM	N	N	N	N	N	N	N	N
12	10-15-01 AM	N	N	N	N	N	N	N	P
13	10-16-01 AM	N	N	N	N	N	N	N	N
14	10-18-01 AM	N	N	N	N	N	N	N	P
15	10-21-01 AM	N	N	N	N	N	N	N	N
16	11-01-01 AM	N	N	N	N	N	N	N	N
17	11-02-01 AM	N	N	N	N	N	N	N	N
18	11-03-01 AM	N	N	N	N	N	N	N	N
19	11-04-01 AM	N	N	N	N	N	N	N	N
20	11-05-01 AM	N	N	N	N	N	N	N	N
21	11-07-01 AM	N	N	N	N	N	N	N	N
22	11-08-01 AM	N	N	N	N	N	N	N	N
23	11-09-01 AM	N	N	N	N	N	N	N	N
24	11-10-01 AM	N	N	N	N	N	N	N	N
25									

Partial view on this file

❖ Harvesting time (GM_Basic)

- The two executive files of GM_Basic (GateMultiplex_basic.exe and GateMultiplex_forWindows.exe) are both stored in the "GM_basic" folder (blue frame) and should be placed in the same folder for operation.
- Please see Fig. S14 and S16 in Additional file 1.

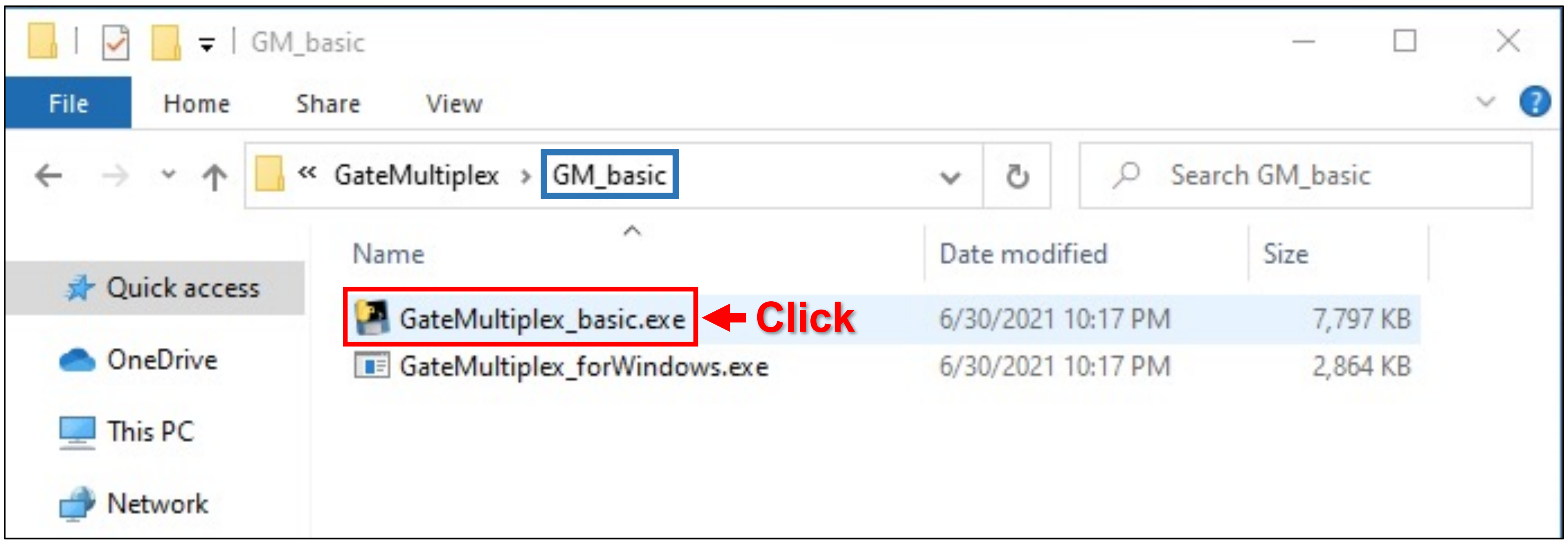

Activate the GM_basic by a double-clicking on "GateMultiplex_basic.exe" (red frame)

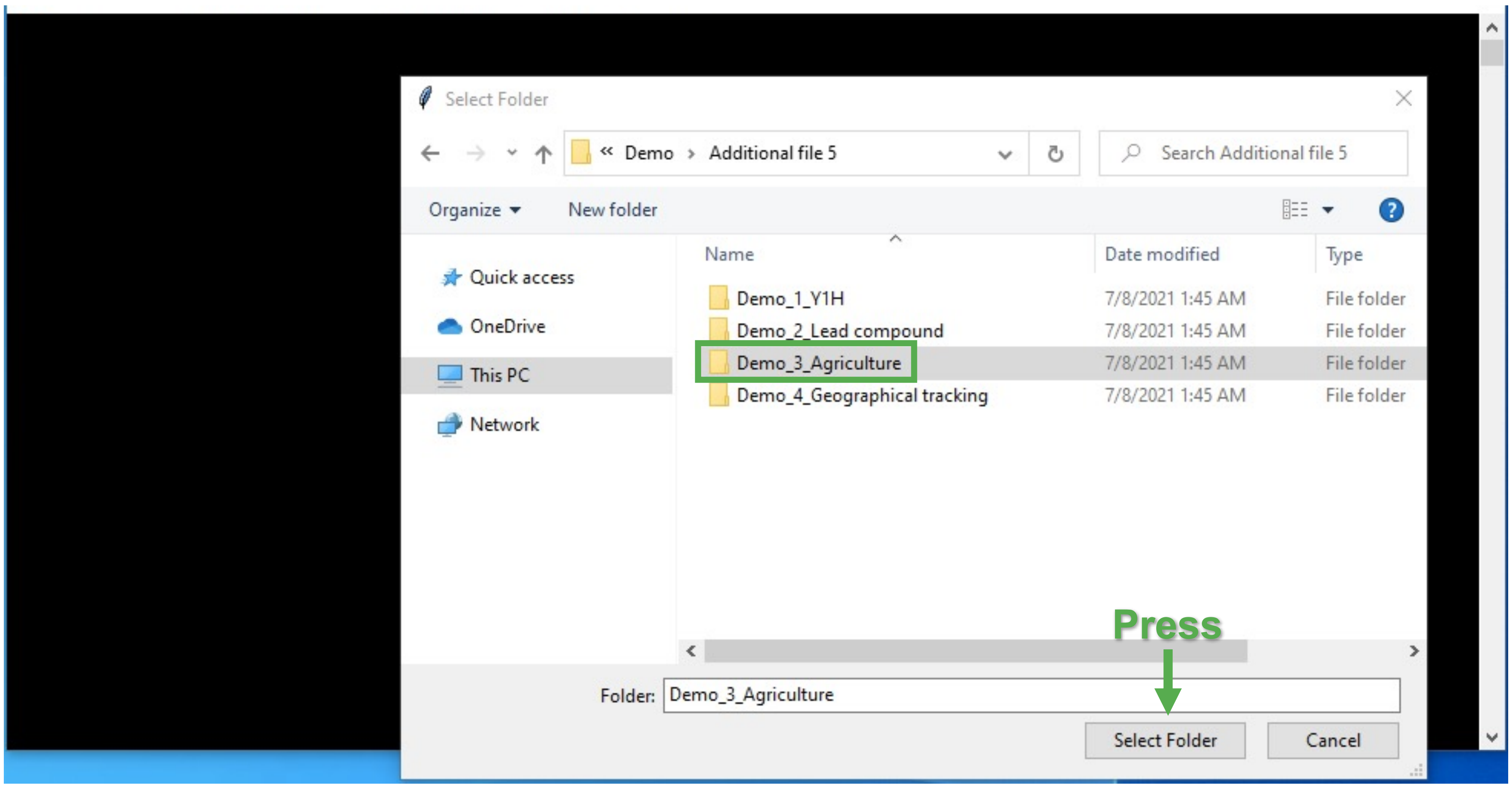

Select the folder "Demo_3_Agriculture" (green frame) from the folder "Additional file 5" and press "Select Folder" (indicated by a green arrow).

✱ The GUI of GM_Basic

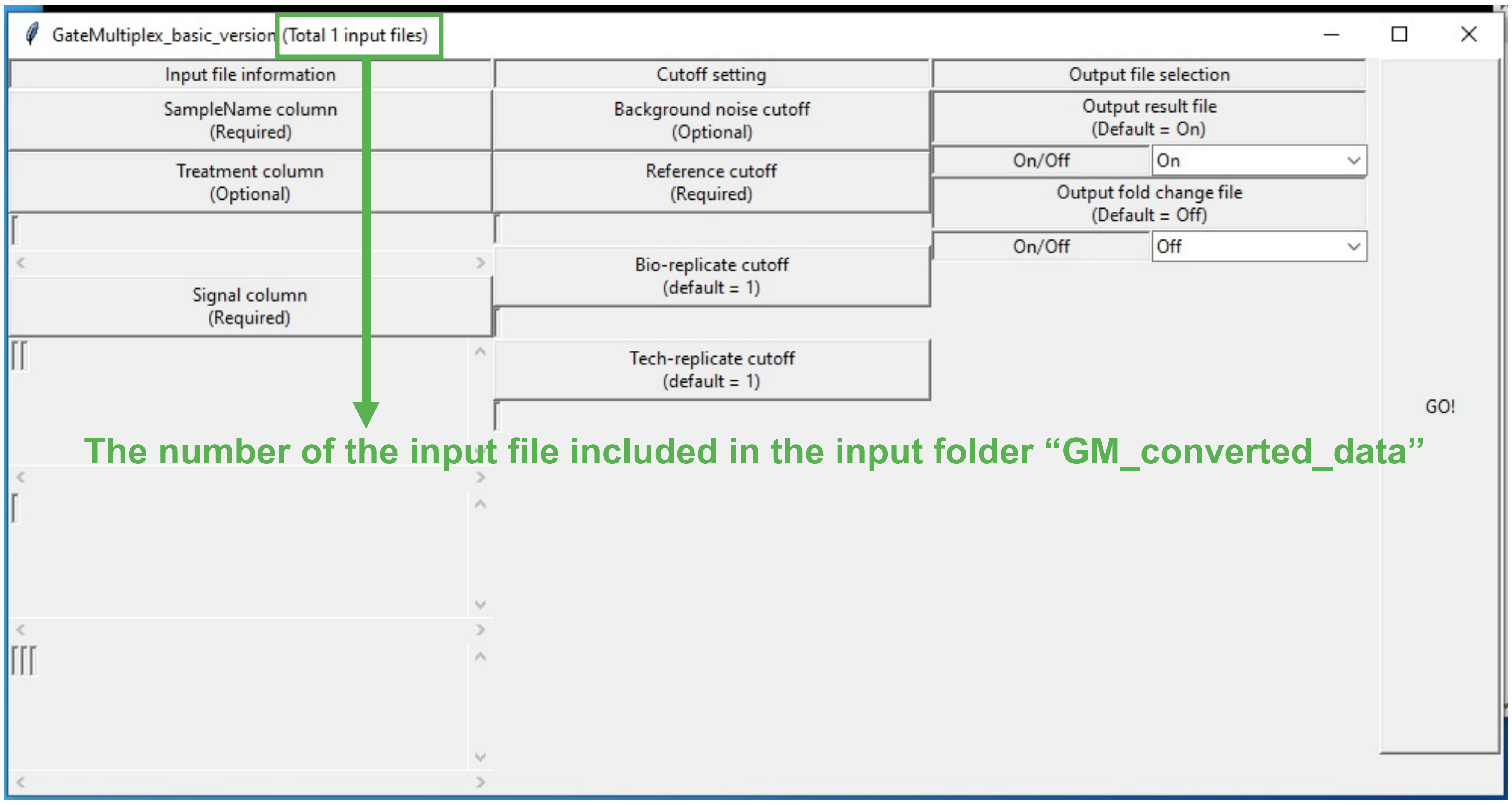

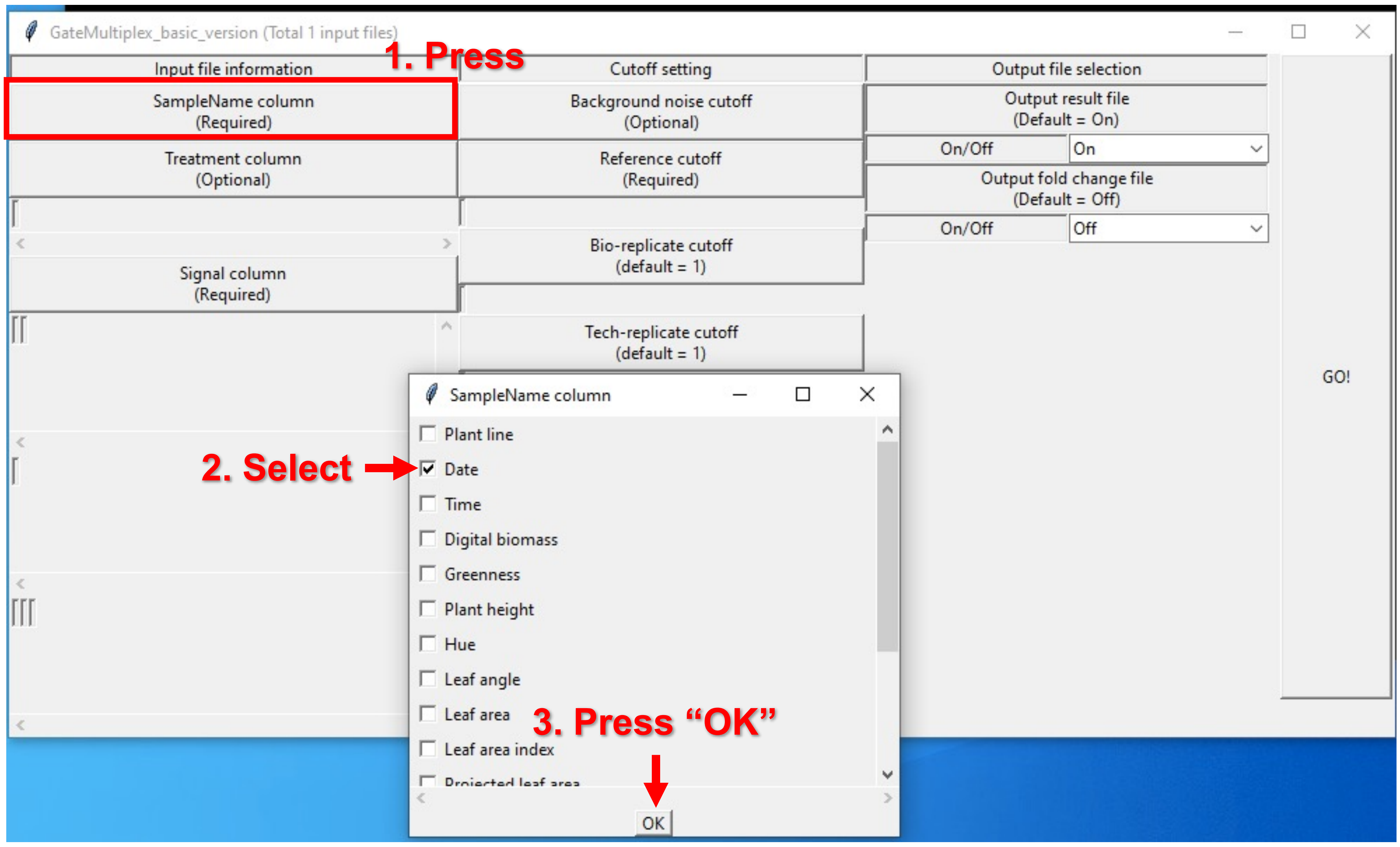

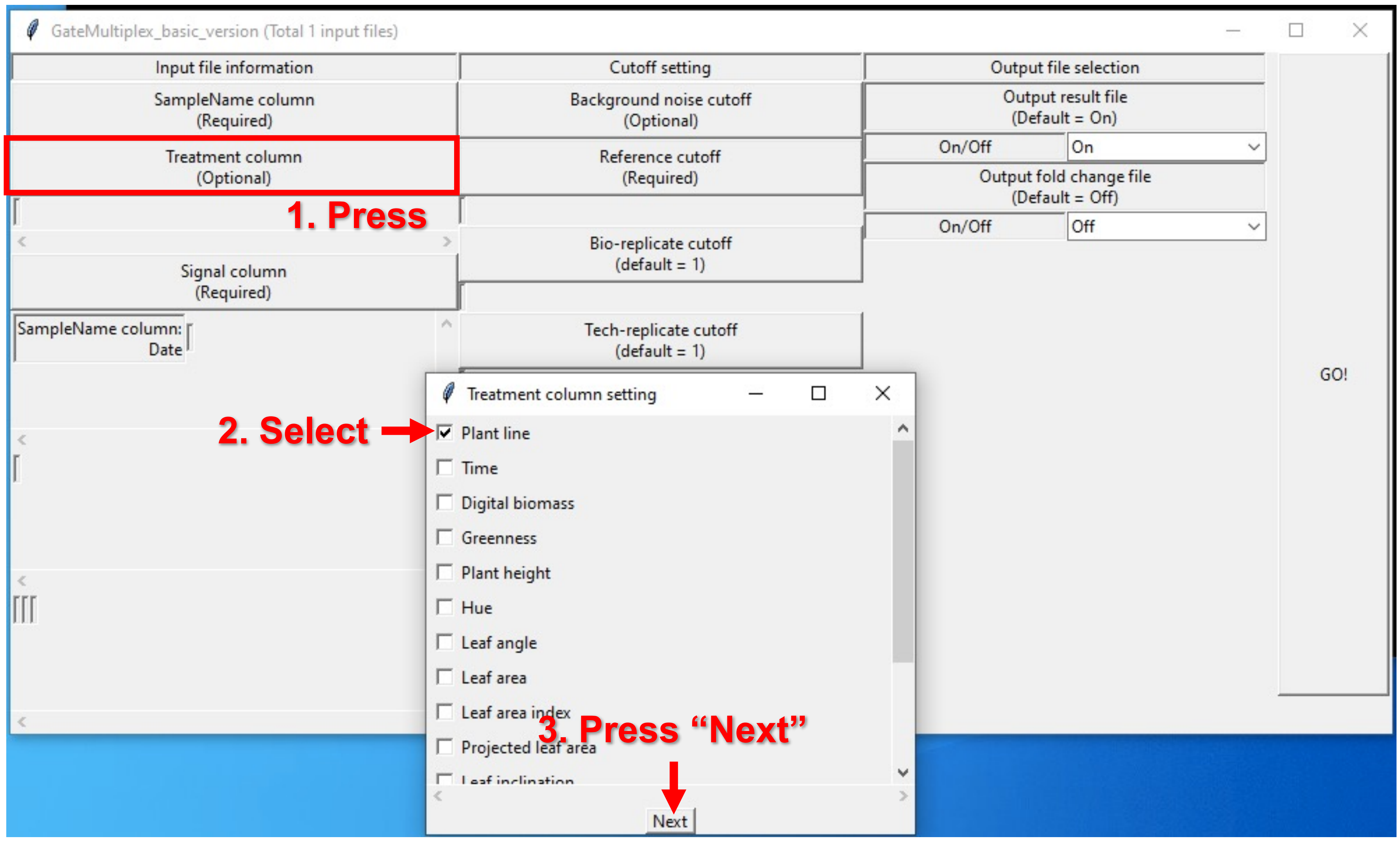
GateMultiplex_basic_version (Total 1 input files)
Input file information
SampleName column (Required)
Treatment column (Optional)
1. Press
Signal column (Required)
SampleName column: Date
Cutoff setting
Background noise cutoff (Optional)
Reference cutoff (Required)
Bio-replicate cutoff (default = 1)
Tech-replicate cutoff (default = 1)
Output file selection
Output result file (Default = On)
On/Off
On
Output fold change file (Default = Off)
On/Off
Off
GO!
Treatment column setting
2. Select
Plant line
Time
Digital biomass
Greenness
Plant height
Hue
Leaf angle
Leaf area
Leaf area index
Projected leaf area
3. Press "Next"
Next

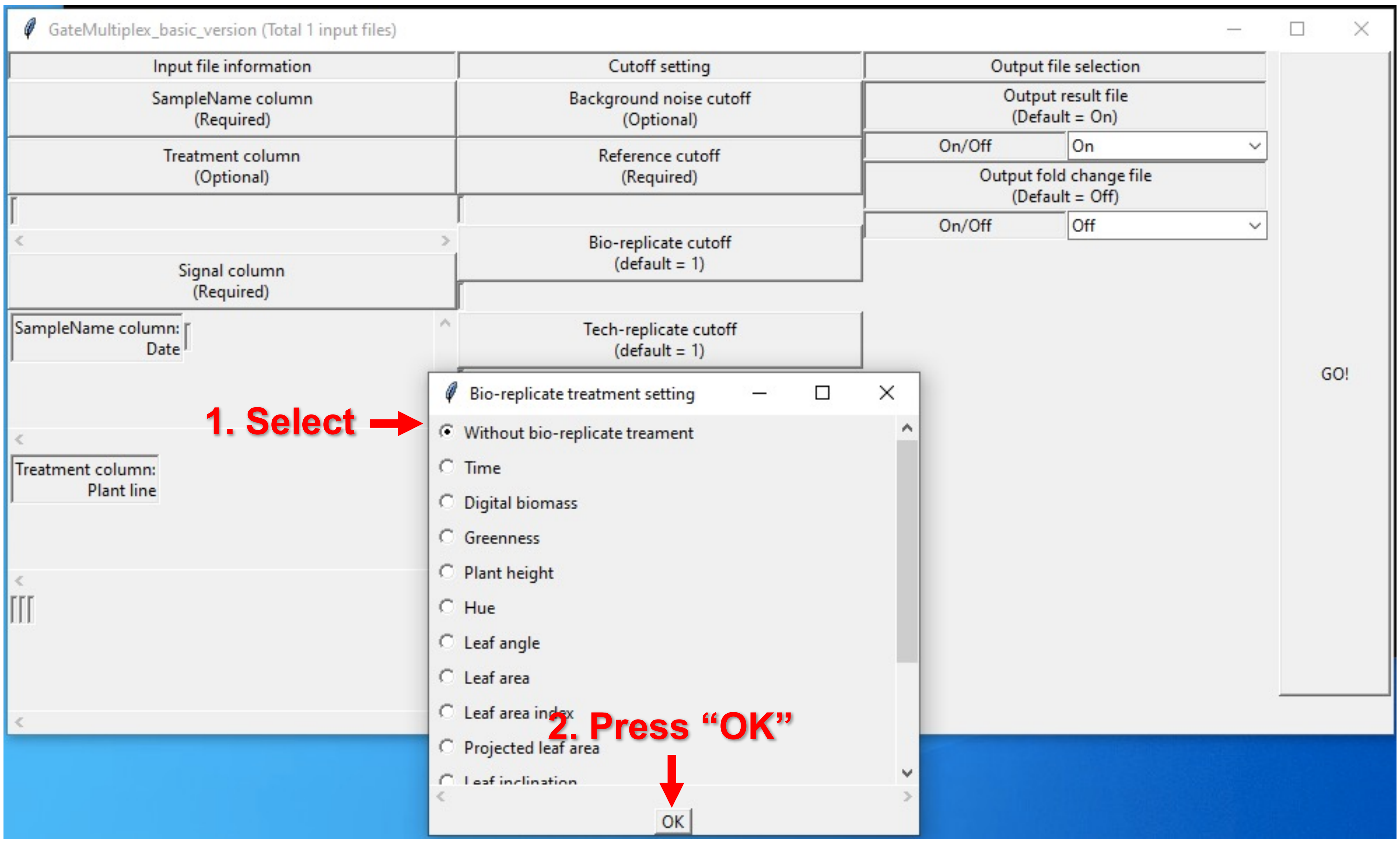
GateMultiplex_basic_version (Total 1 input files)
Input file information
SampleName column (Required)
Treatment column (Optional)
Signal column (Required)
SampleName column: Date
Treatment column: Plant line
Cutoff setting
Background noise cutoff (Optional)
Reference cutoff (Required)
Bio-replicate cutoff (default = 1)
Tech-replicate cutoff (default = 1)
Output file selection
Output result file (Default = On)
On/Off
On
Output fold change file (Default = Off)
On/Off
Off
GO!
Bio-replicate treatment setting
1. Select
Without bio-replicate treament
Time
Digital biomass
Greenness
Plant height
Hue
Leaf angle
Leaf area
Leaf area index
Projected leaf area
2. Press "OK"
OK

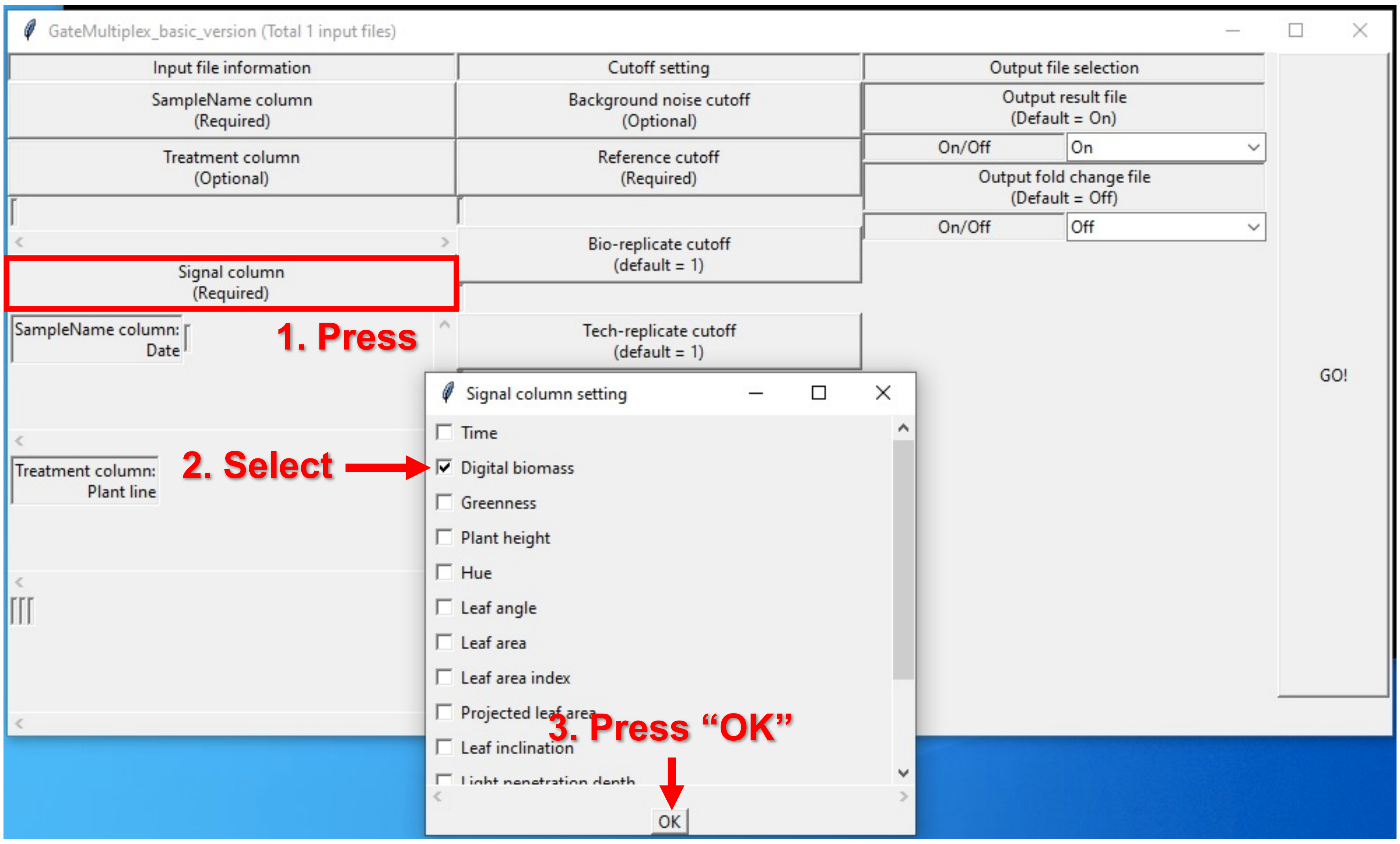
GateMultiplex_basic_version (Total 1 input files)
Input file information
Cutoff setting
Output file selection
SampleName column (Required)
Background noise cutoff (Optional)
Output result file (Default = On)
On/Off
On
Treatment column (Optional)
Reference cutoff (Required)
Output fold change file (Default = Off)
On/Off
Off
Bio-replicate cutoff (default = 1)
Signal column (Required)
SampleName column: Date
1. Press
Tech-replicate cutoff (default = 1)
GO!
Signal column setting
Time
Digital biomass
Greenness
Plant height
Hue
Leaf angle
Leaf area
Leaf area index
Projected leaf area
Leaf inclination
Treatment column: Plant line
2. Select
3. Press "OK"
OK

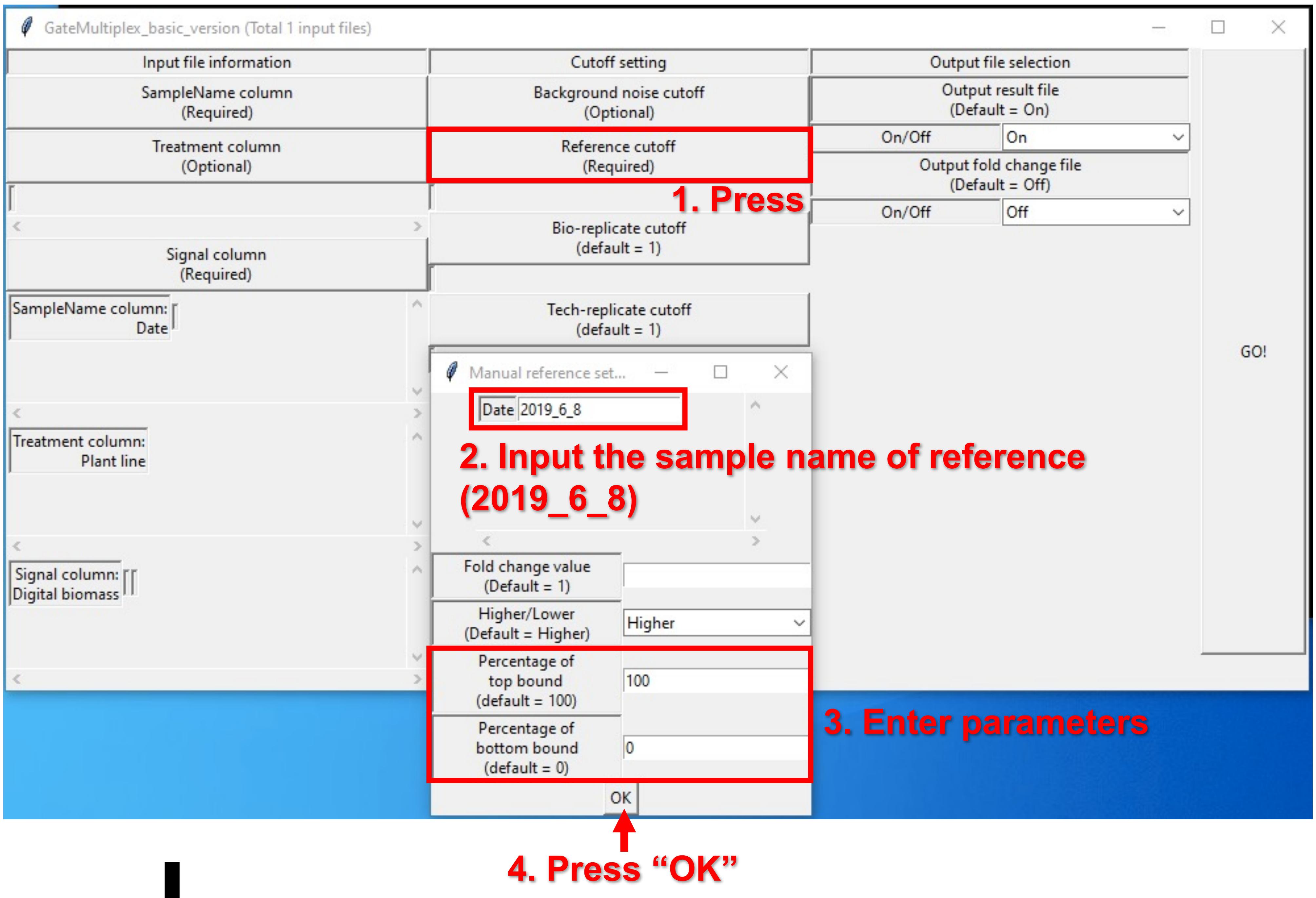
GateMultiplex_basic_version (Total 1 input files)
Input file information
Cutoff setting
Output file selection
SampleName column (Required)
Background noise cutoff (Optional)
Output result file (Default = On)
On/Off
On
Treatment column (Optional)
Reference cutoff (Required)
Output fold change file (Default = Off)
1. Press
On/Off
Off
Bio-replicate cutoff (default = 1)
Signal column (Required)
SampleName column: Date
Tech-replicate cutoff (default = 1)
GO!
Manual reference set...
Date 2019_6_8
Treatment column: Plant line
2. Input the sample name of reference (2019_6_8)
Signal column: Digital biomass
Fold change value (Default = 1)
Higher/Lower (Default = Higher)
Higher
Percentage of top bound (default = 100)
100
Percentage of bottom bound (default = 0)
0
3. Enter parameters
OK
4. Press "OK"

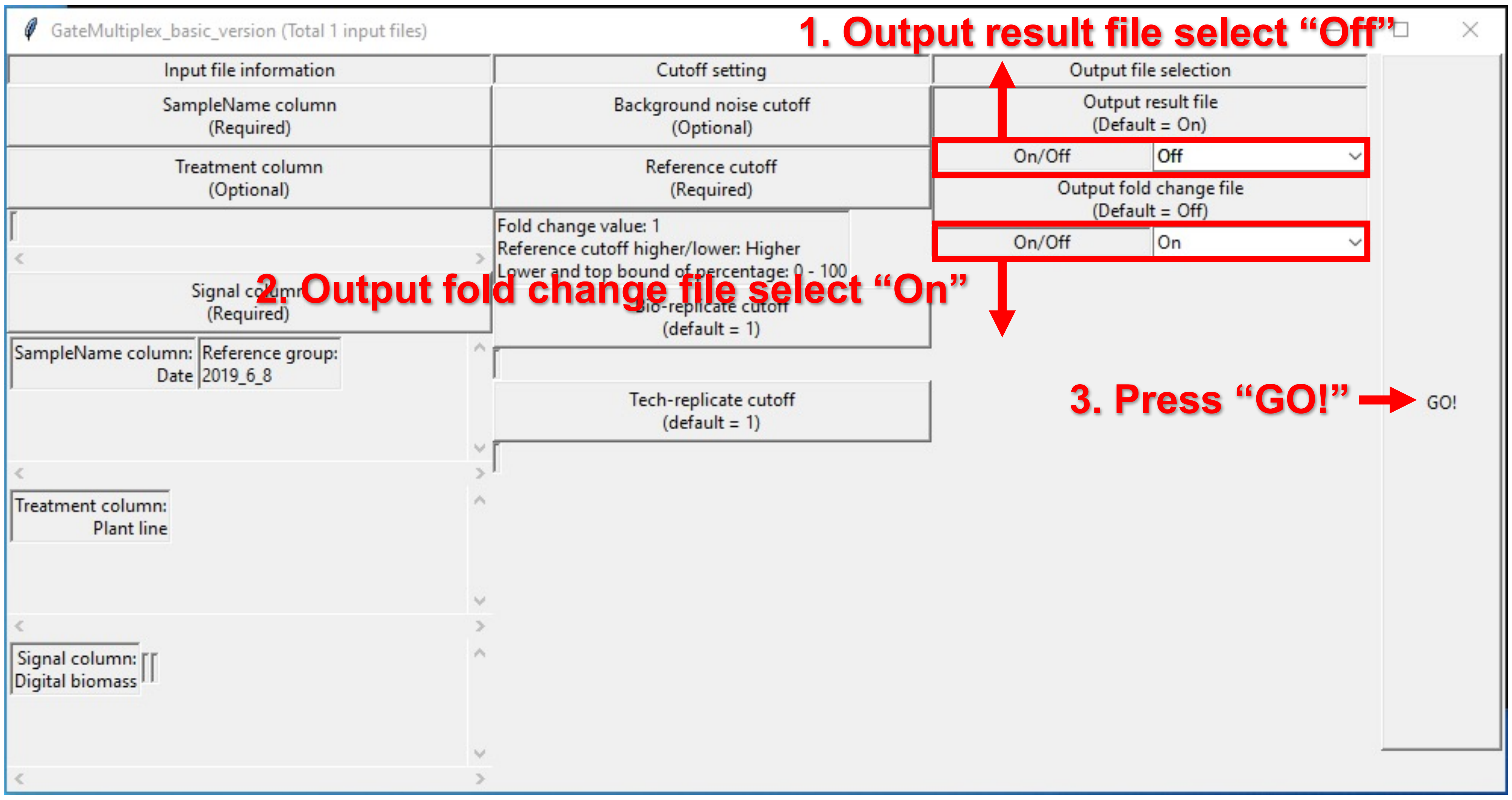

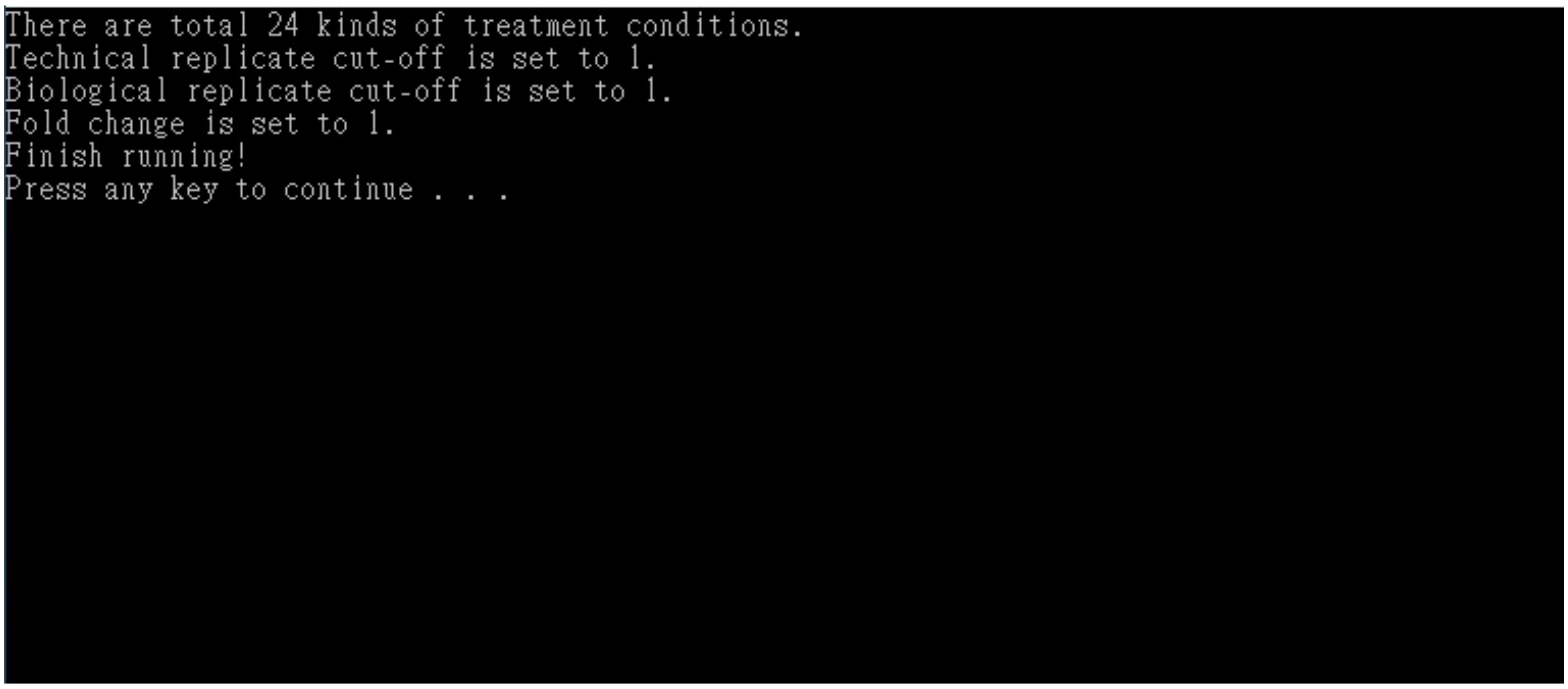

Press any key to close the window

- The fold change files are stored in the folder “GM_outputfiles” (in green frame).
- Please see the Figure L1-L3 in Additional file 2 for the concept of mutiple fold change files.
- Please see the “Symbols in output files” section in Additional file 3 for the concept of symbols in fold change files

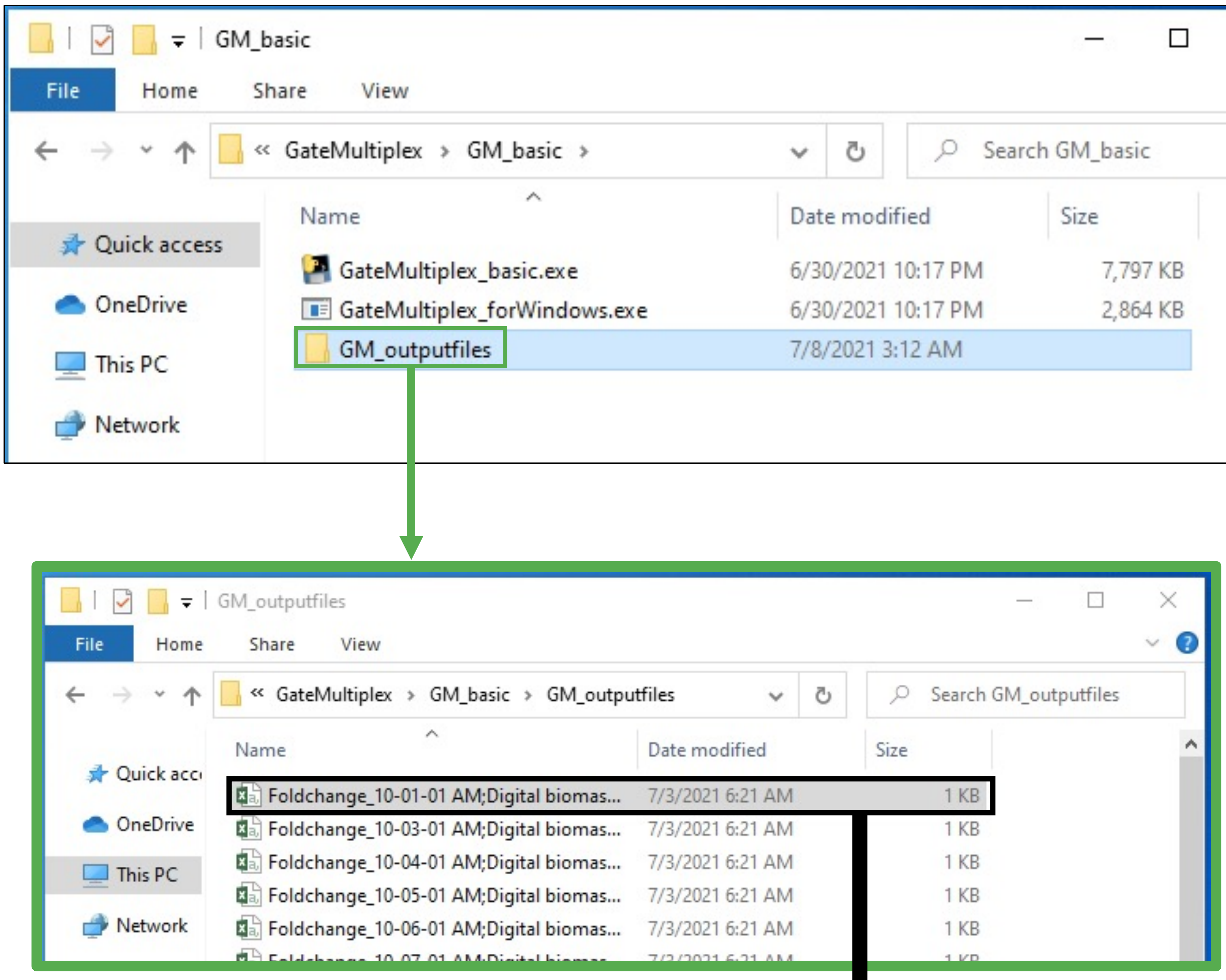

Make a chart using the results of a fold change file (the fold change file of plant line 10-01-01 AM is the example)

“Foldchange_10-01-01 AM;Digital biomass;.csv” file

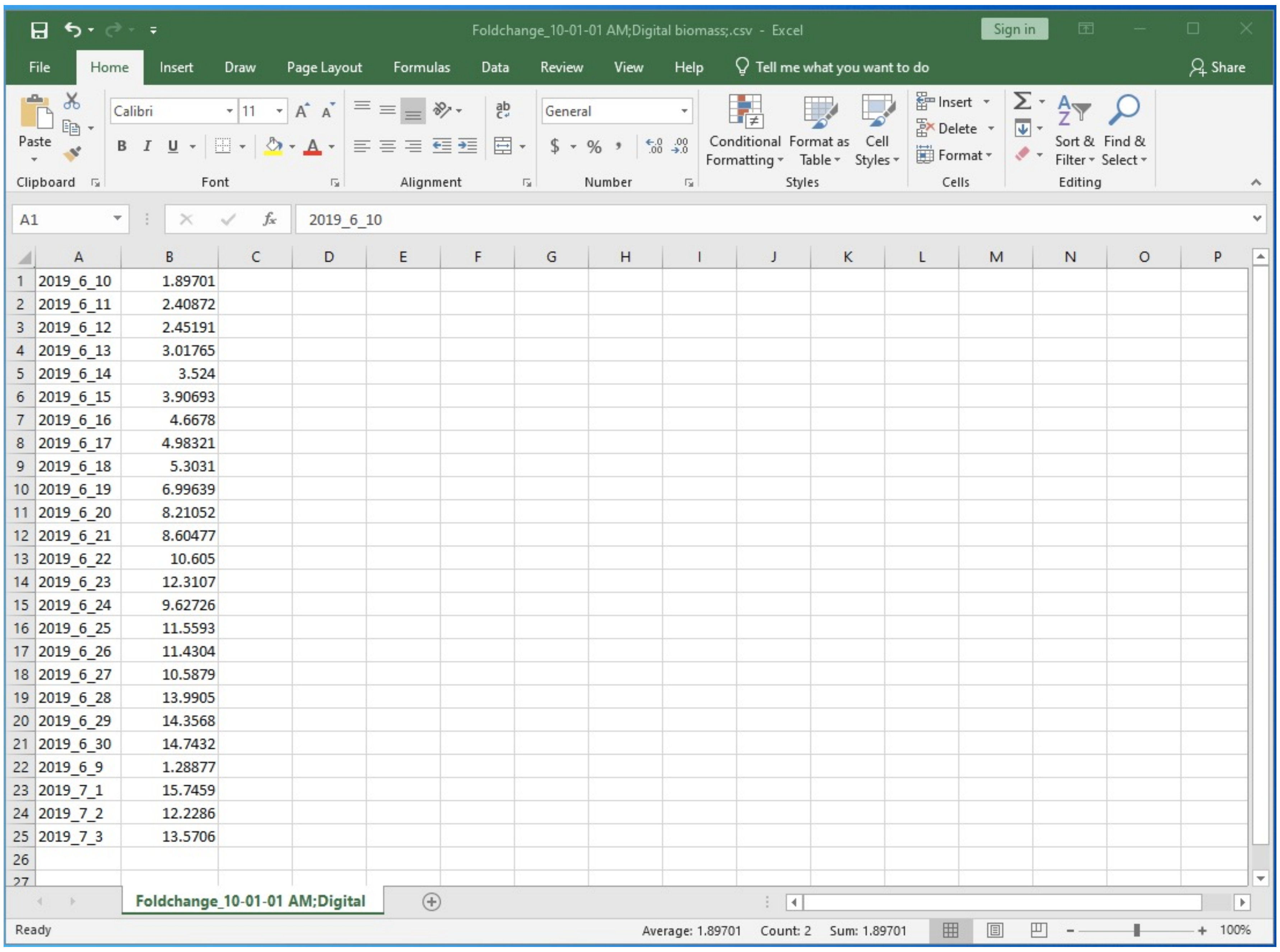

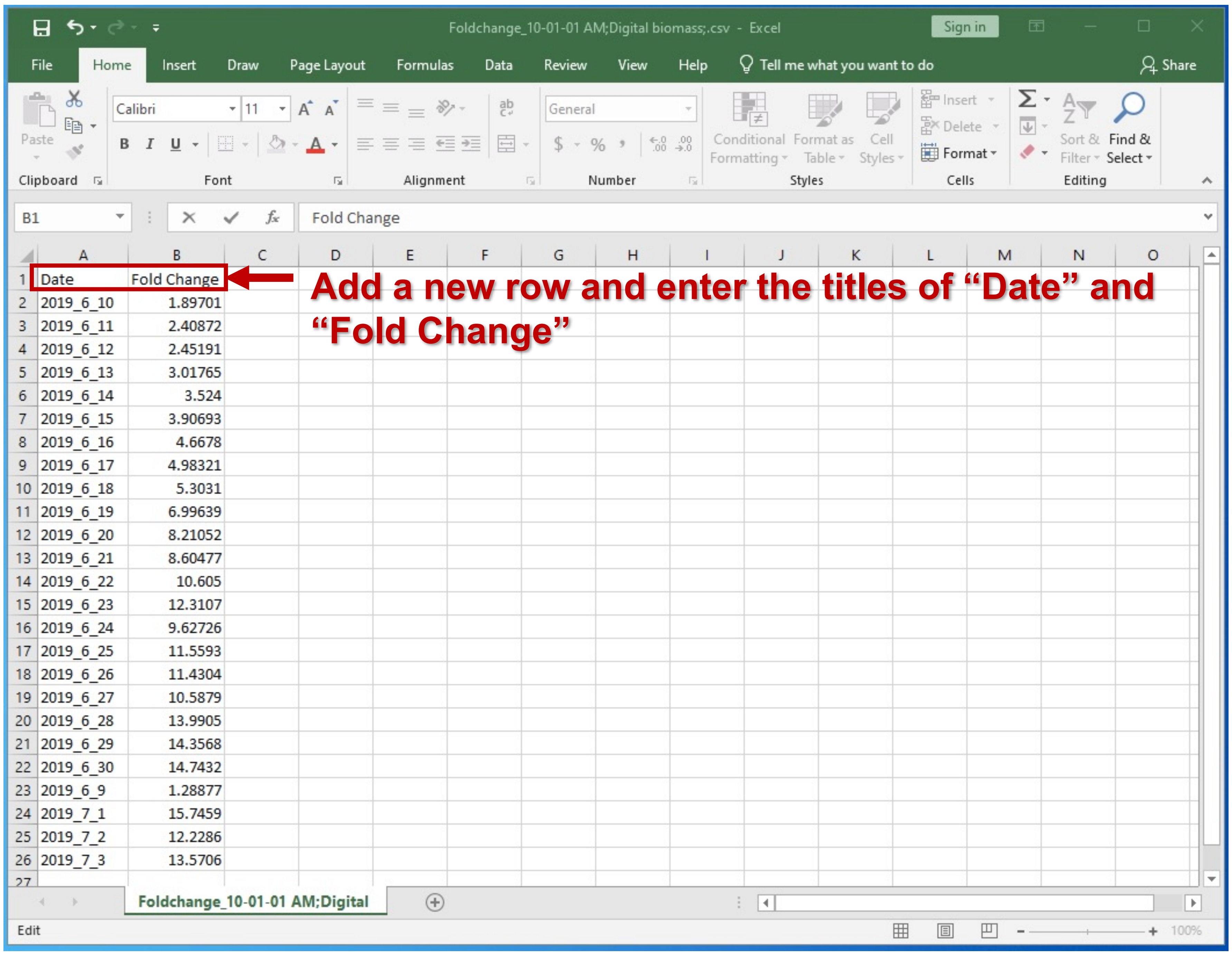

- Replace the "_" in dates with "/"

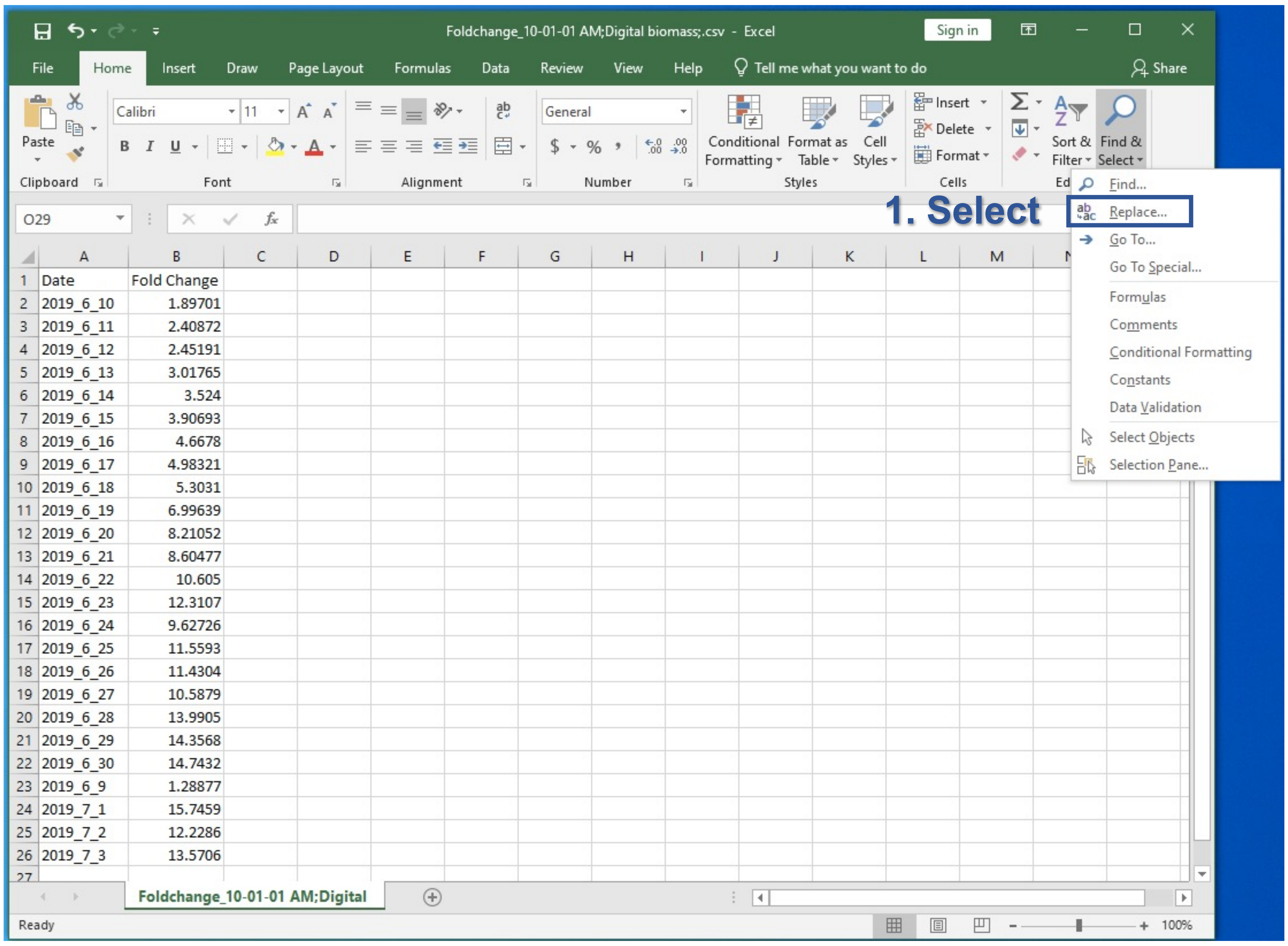

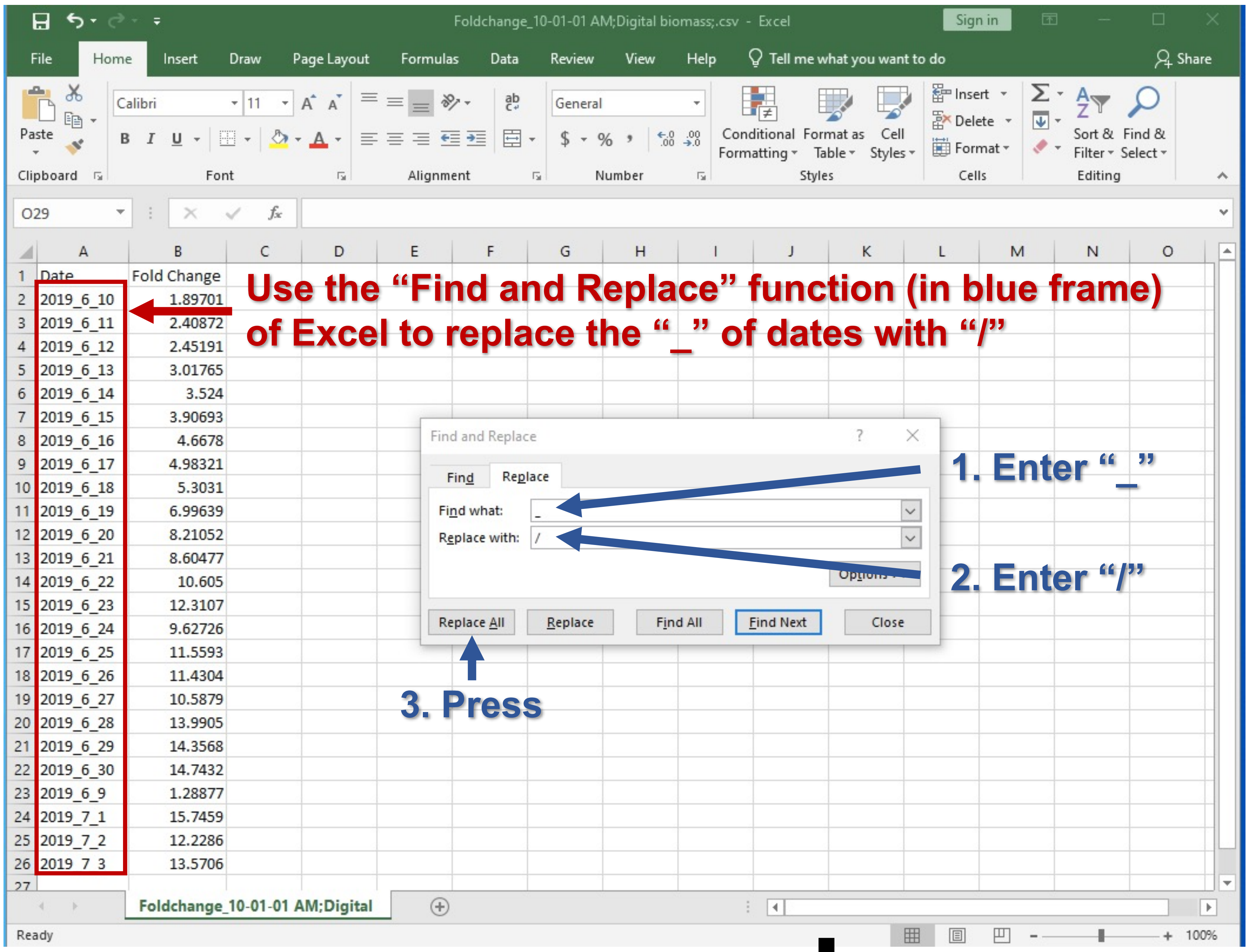

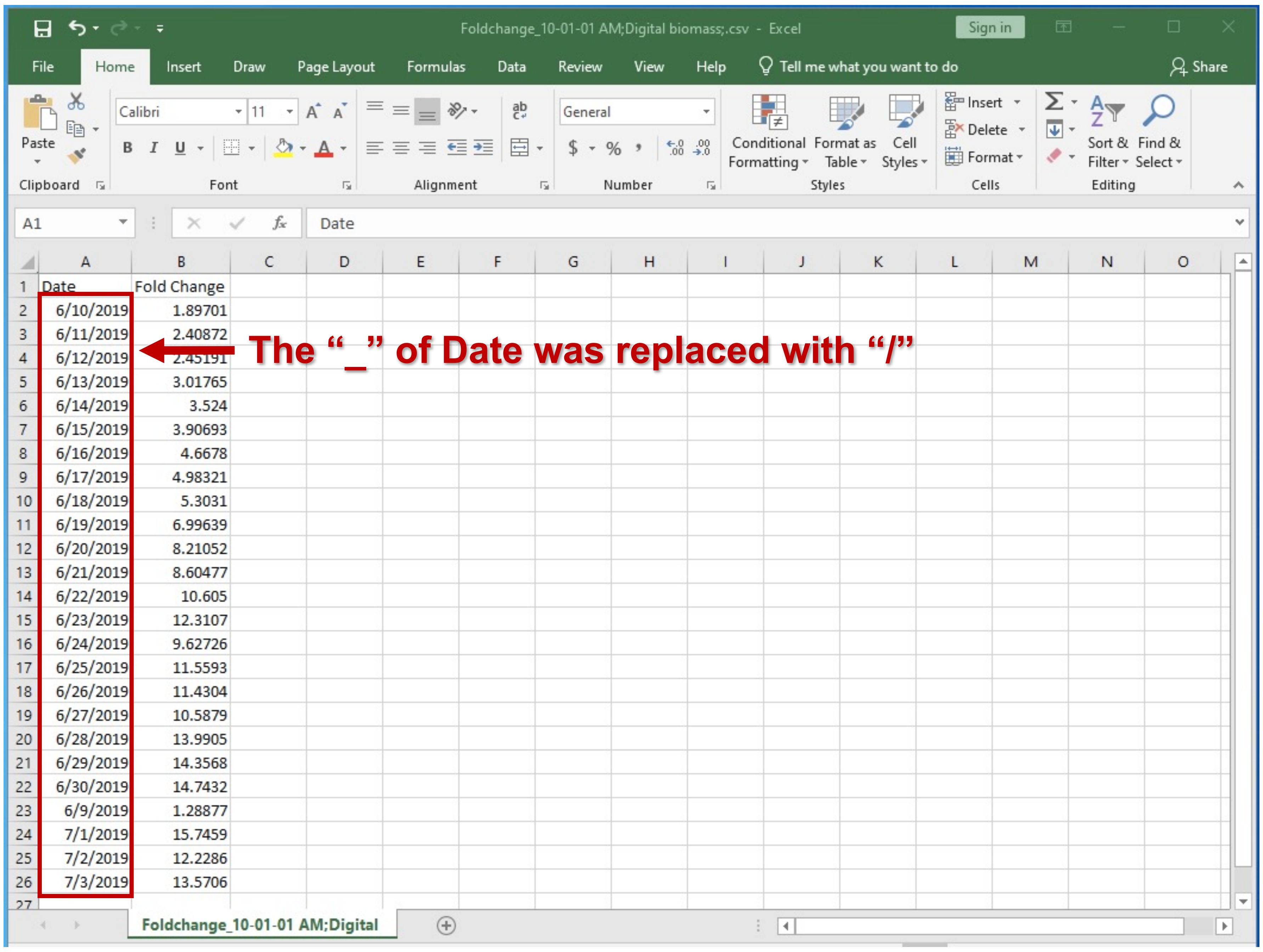

- Sort the dates from the oldest to the newest

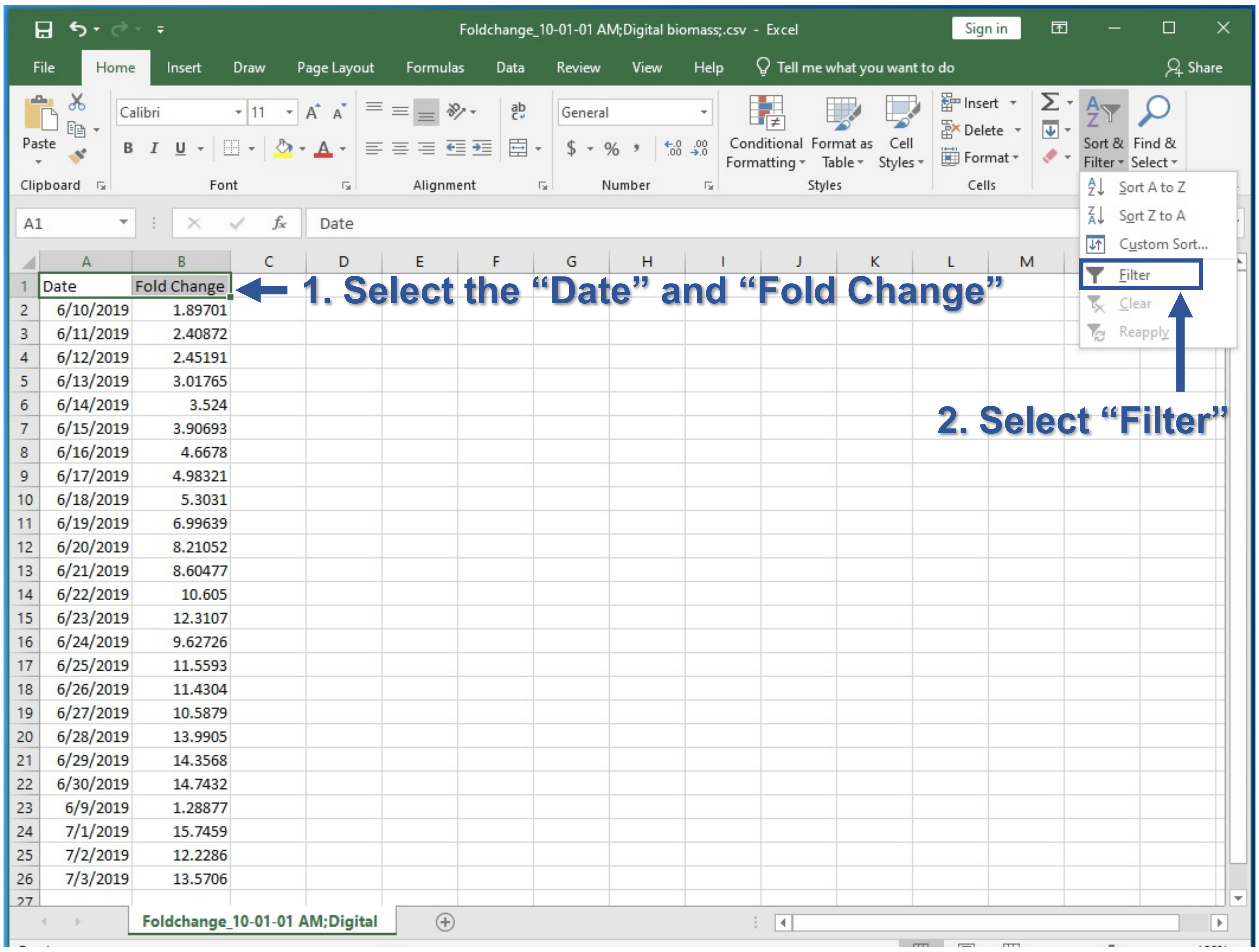

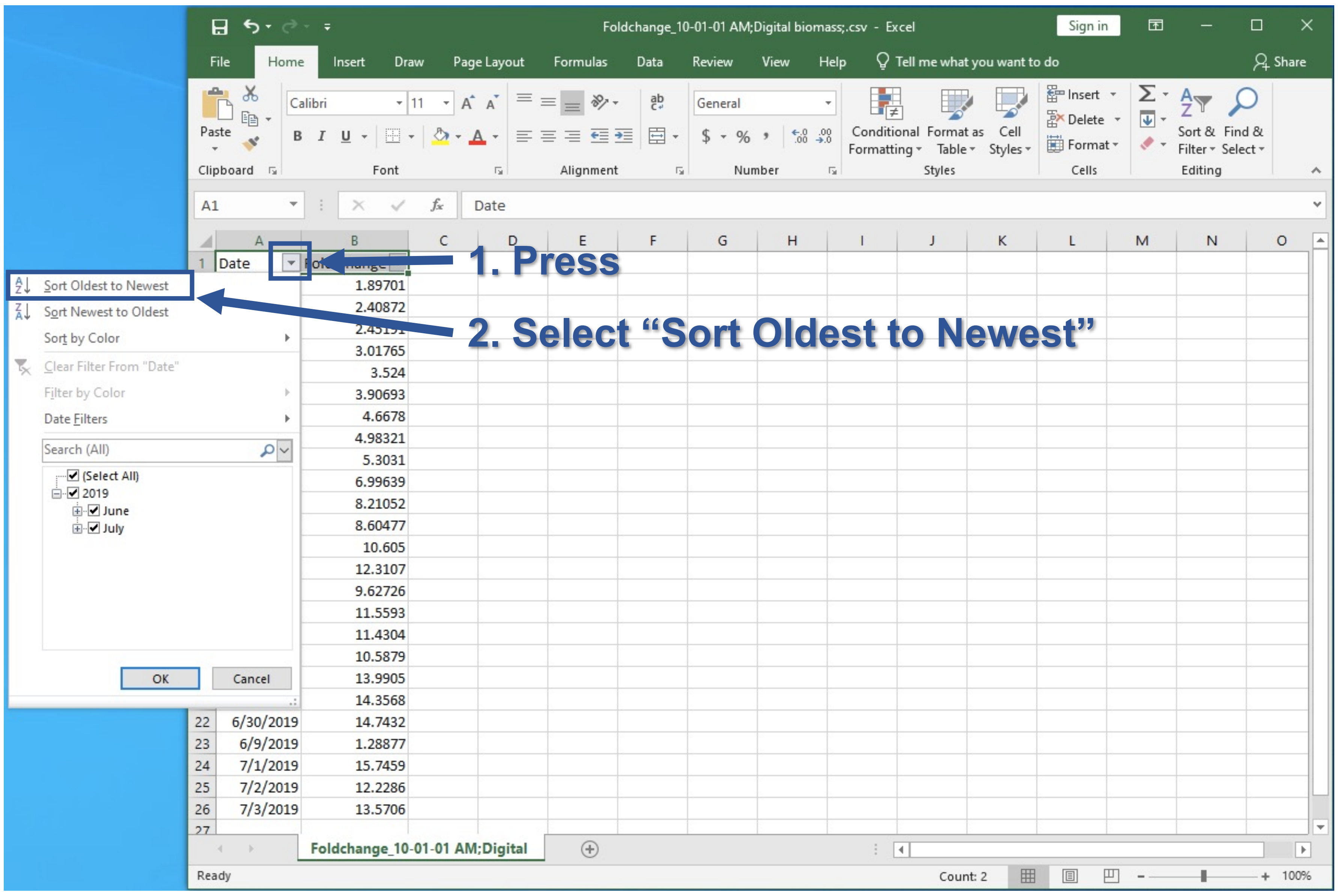
1. Press
2. Select "Sort Oldest to Newest"
Sort Oldest to Newest
Sort Newest to Oldest

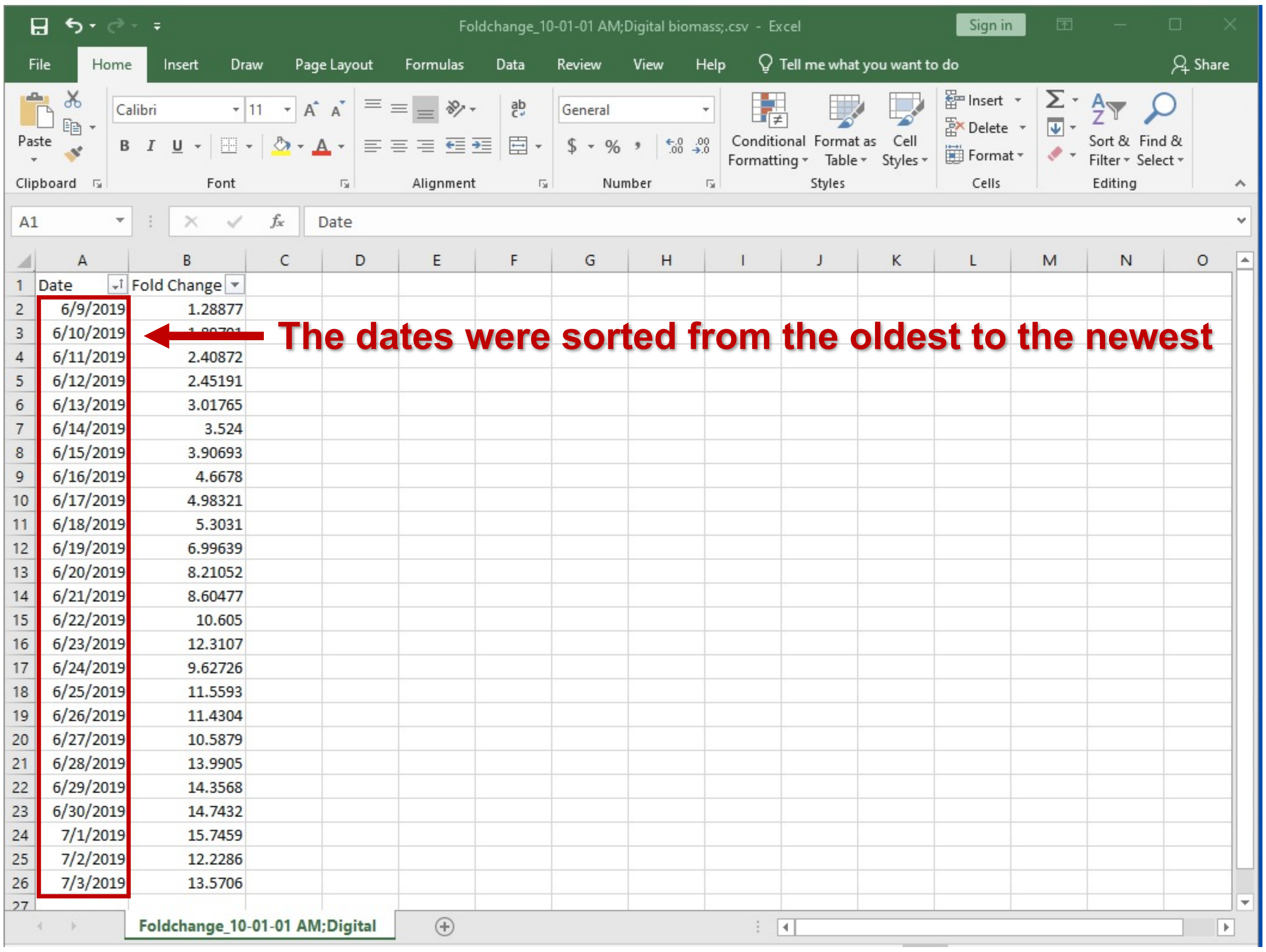
The dates were sorted from the oldest to the newest

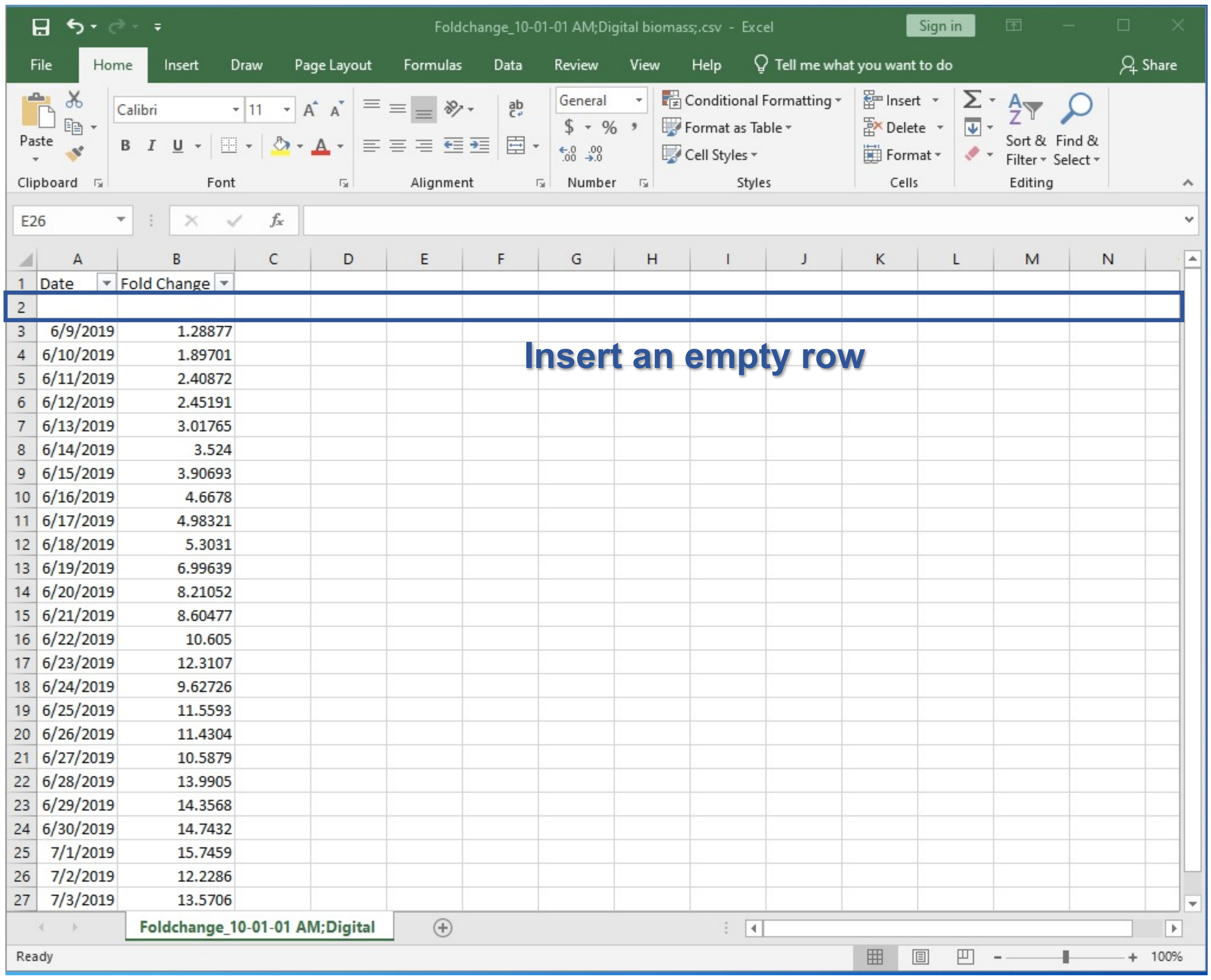
Insert an empty row

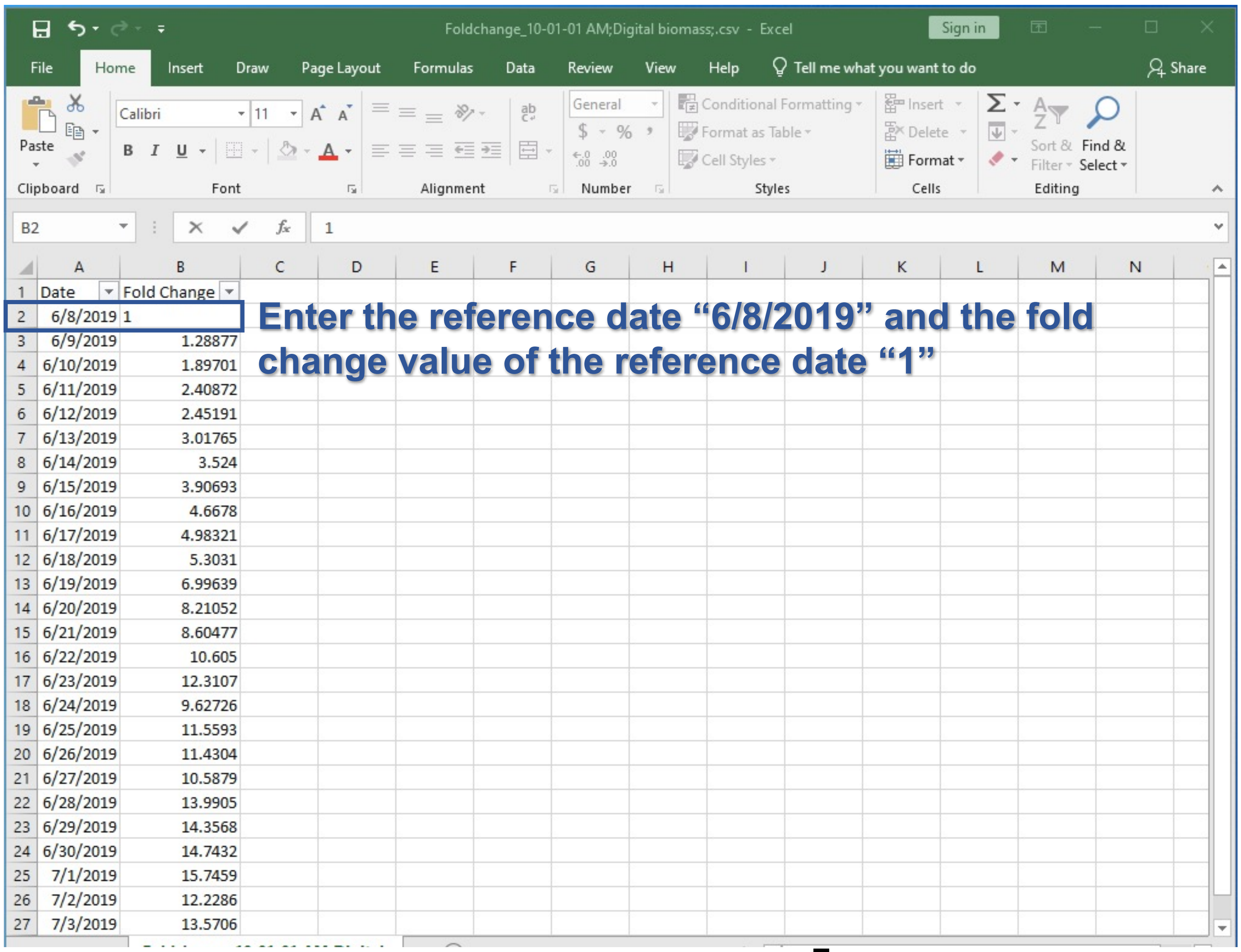
Enter the reference date “6/8/2019” and the fold change value of the reference date “1”

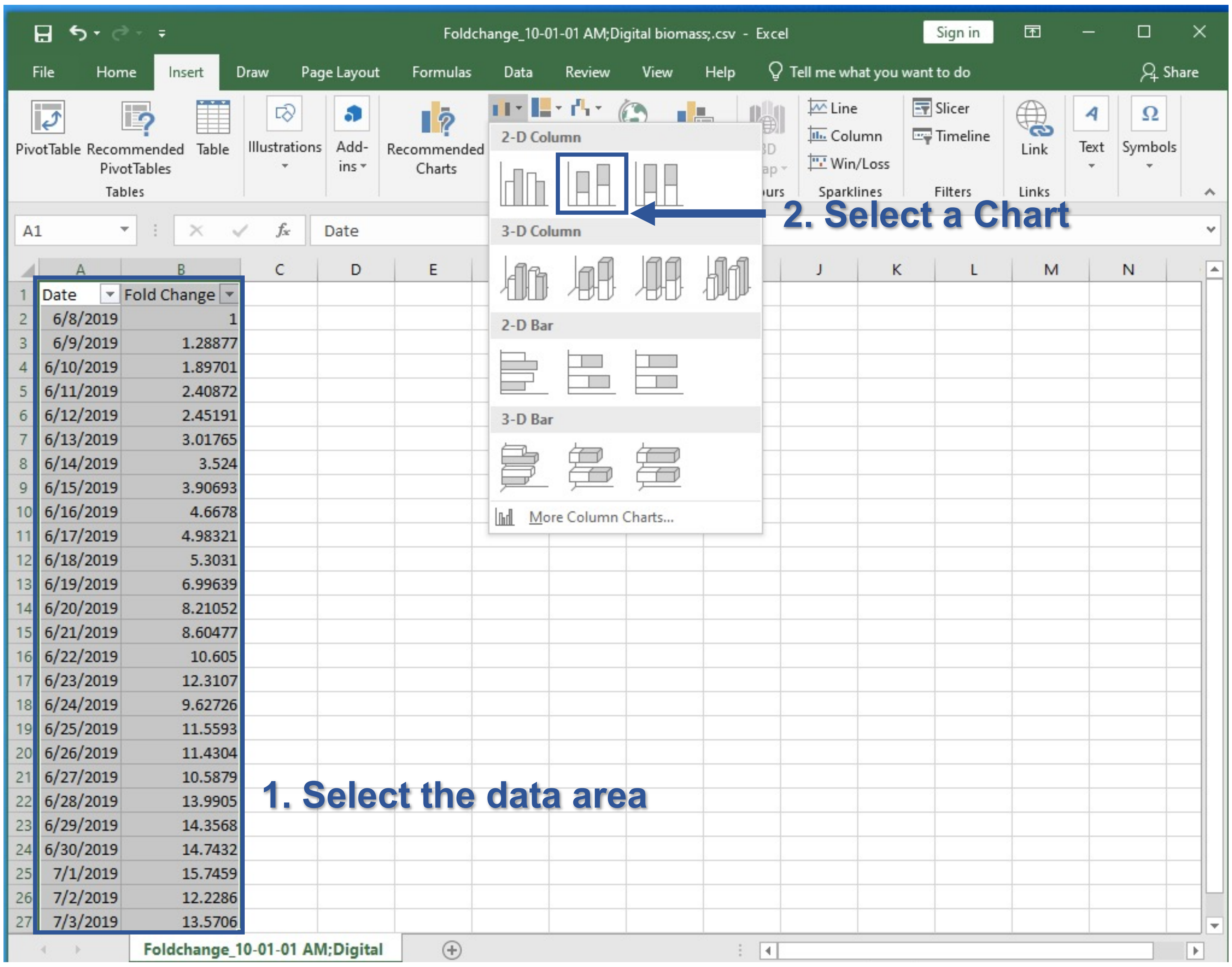
Foldchange_10-01-01 AM;Digital biomass;.csv - Excel
2-D Column
3-D Column
2-D Bar
3-D Bar
More Column Charts...
2. Select a Chart
1. Select the data area
Foldchange_10-01-01 AM;Digital

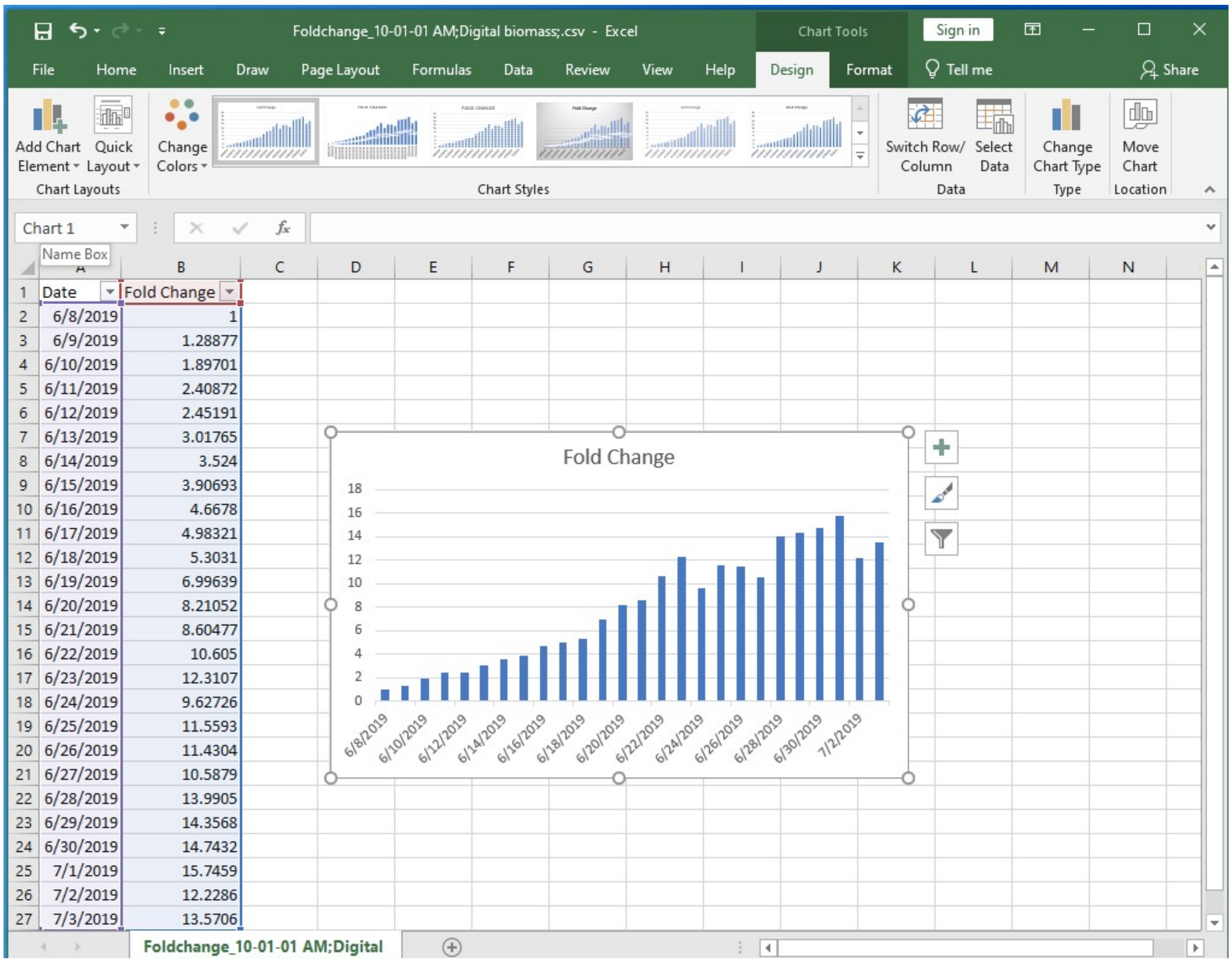
Foldchange_10-01-01 AM;Digital biomass;.csv - Excel
Chart Tools
Fold Change
Foldchange_10-01-01 AM;Digital

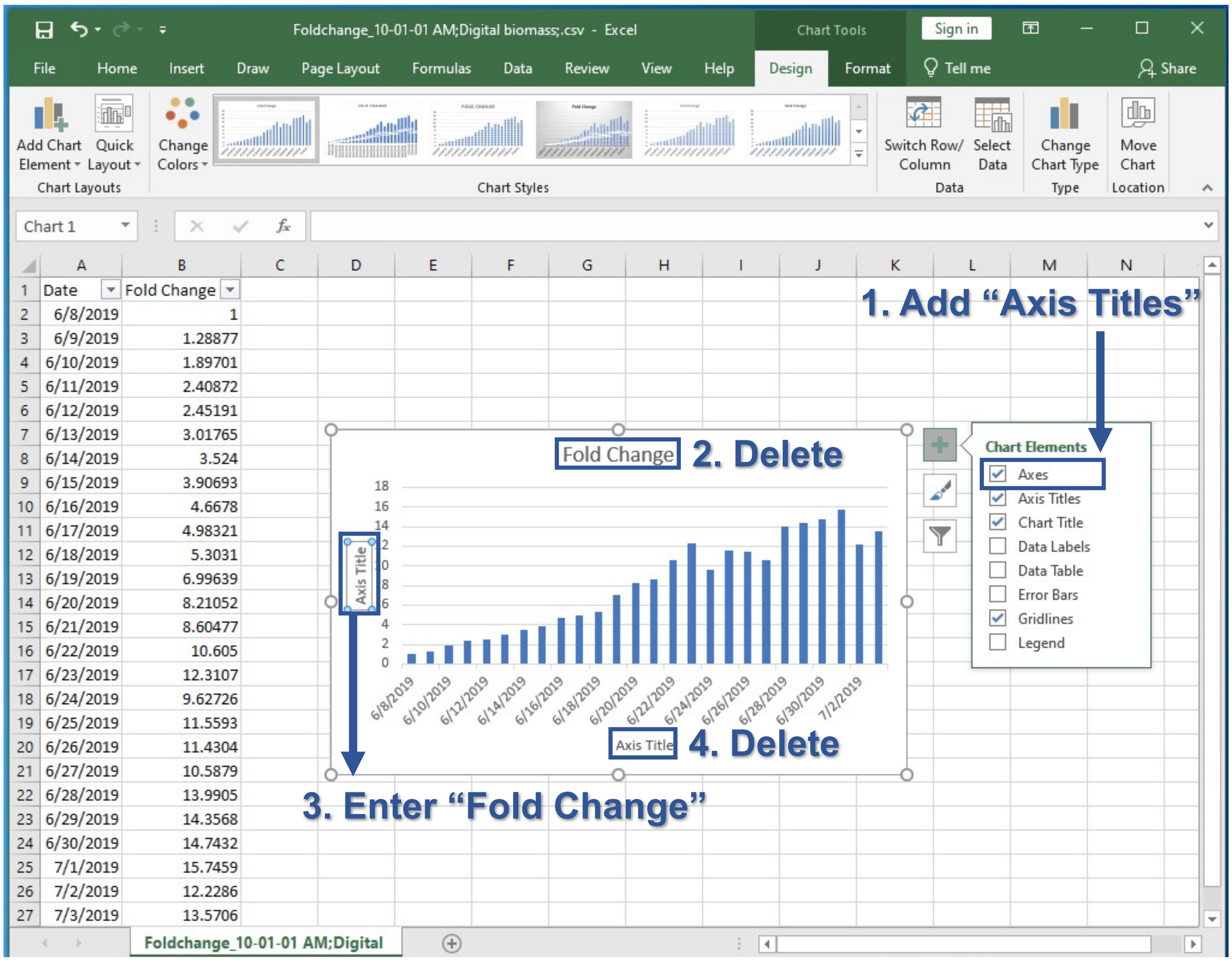

Resize the chart

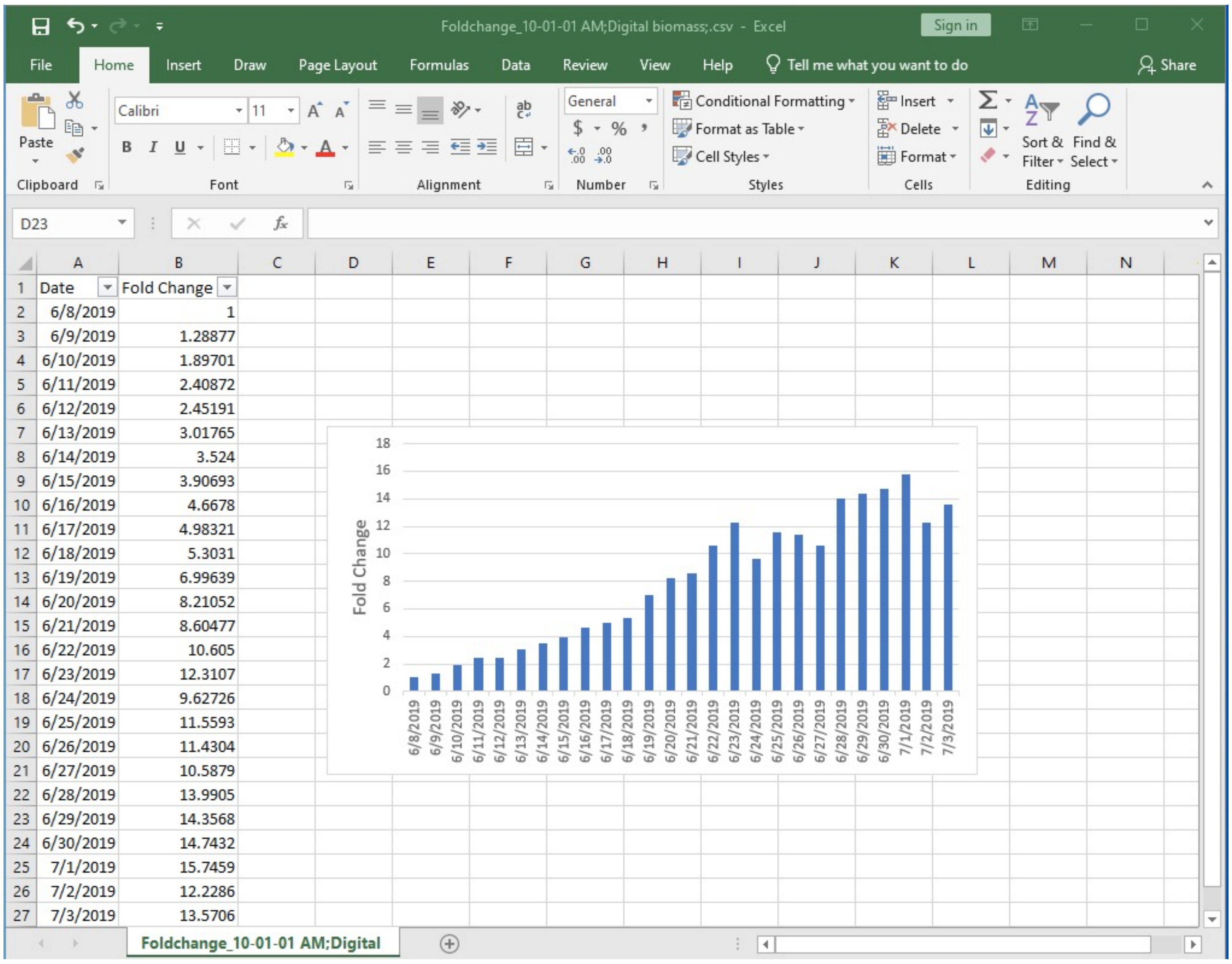

Geographical tracking for marine ecosystems (GM_Basic)

Geographical tracking for marine ecosystems (GM_Basic)

- The location of marine ecosystem demo data.

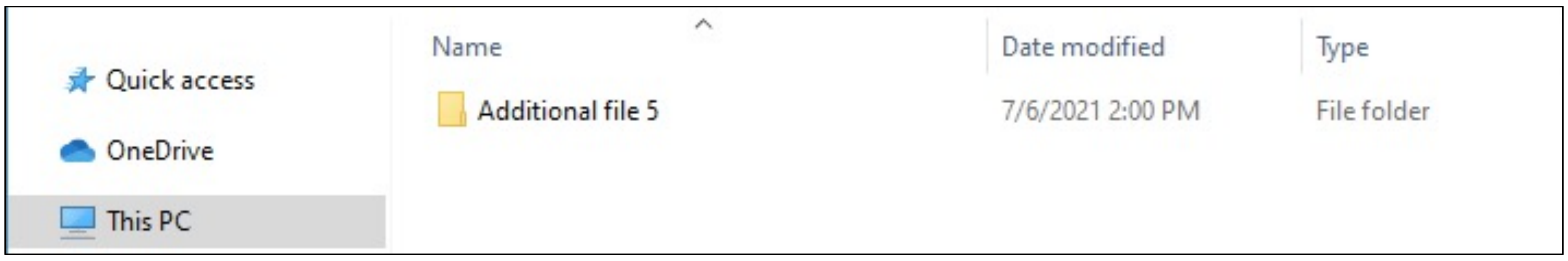

Open the “Additional file 5” folder

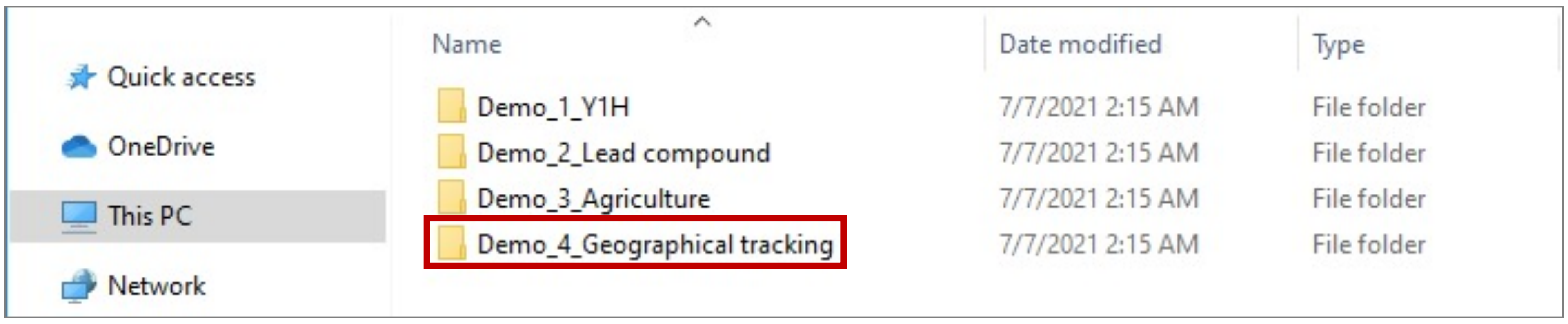

Open the “Demo_4_Geographical tracking” folder (red frame)

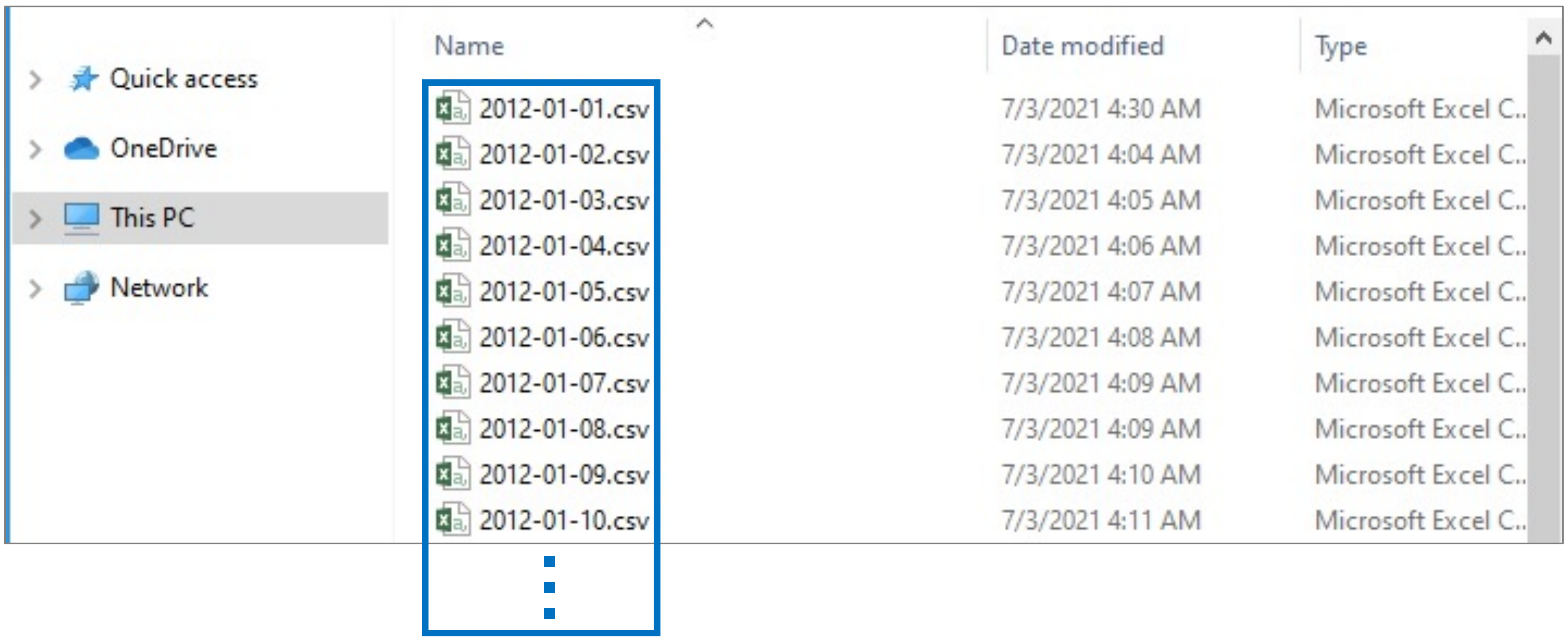

Total 31 files

*The 31 files (blue frame) included in the “Demo_4_Geographical tracking” folder are the demo data for marine ecosystems.

*Please see the Figure B1 in Additional file 2 for the concept of multiple input files.

- Take “2012-01-01.csv” (green frame) as the example for showing the content of the files (All files show similar contents).

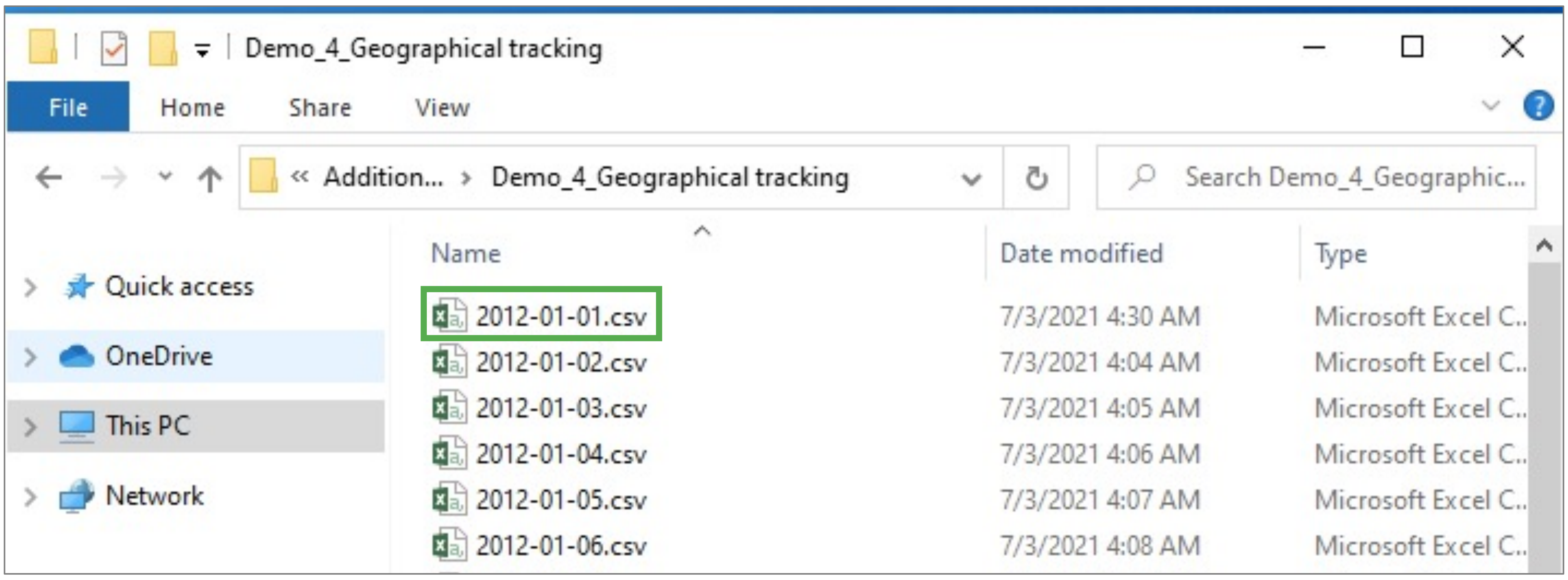

“2012-01-01.csv” file

	A	B	C	D	E	F	G	H
1	Date	Latitude	Longitude	Country	Gear type	Vessel hour	Fishing hour	
2	1/1/2012	-4685	-6490	ARG	trawlers	1.59625	0	
3	1/1/2012	-4274	-6503	ARG	trawlers	28.69722222	0	
4	1/1/2012	-3463	-5836	ARG	trawlers	16.52763889	0	
5	1/1/2012	-4762	-6371	ARG	trawlers	0.805555556	0	
6	1/1/2012	-4768	-6343	ARG	trawlers	0.049722222	0	
7	1/1/2012	-3458	-5839	ARG	trawlers	17.99902778	0	
8	1/1/2012	-4201	-5846	ARG	trawlers	0.81125	0	
9	1/1/2012	-4683	-6493	ARG	trawlers	1.605694444	0	
10	1/1/2012	-4771	-6356	ARG	trawlers	0.034583333	0	
11	1/1/2012	-4770	-6358	ARG	trawlers	0.048611111	0	
12	1/1/2012	-4761	-6386	ARG	trawlers	8.277916667	0	
13	1/1/2012	-4770	-6359	ARG	trawlers	0.733333333	0	

*The information title of each column is circled by red frame.

*The data of each column is circled by a blue frame.

❖ Marine ecosystems (GM_Basic)

- The two executive files of GM_Basic (GateMultiplex_basic.exe and GateMultiplex_forWindows.exe) are stored in the same "GM_basic" folder and should be placed in the same folder for operation.

- Please see Fig. S18 and S19 in Additional file 1.

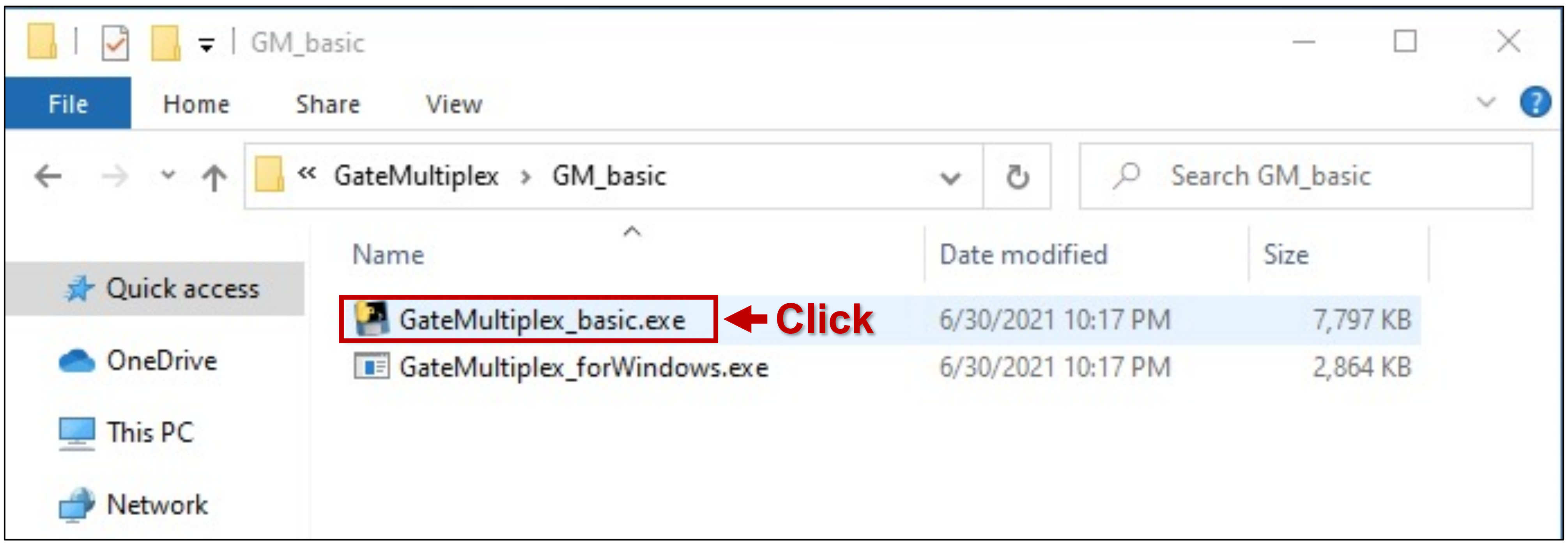

Activate the GM_basic by a double-clicking on "GateMultiplex_basic.exe" (red frame)

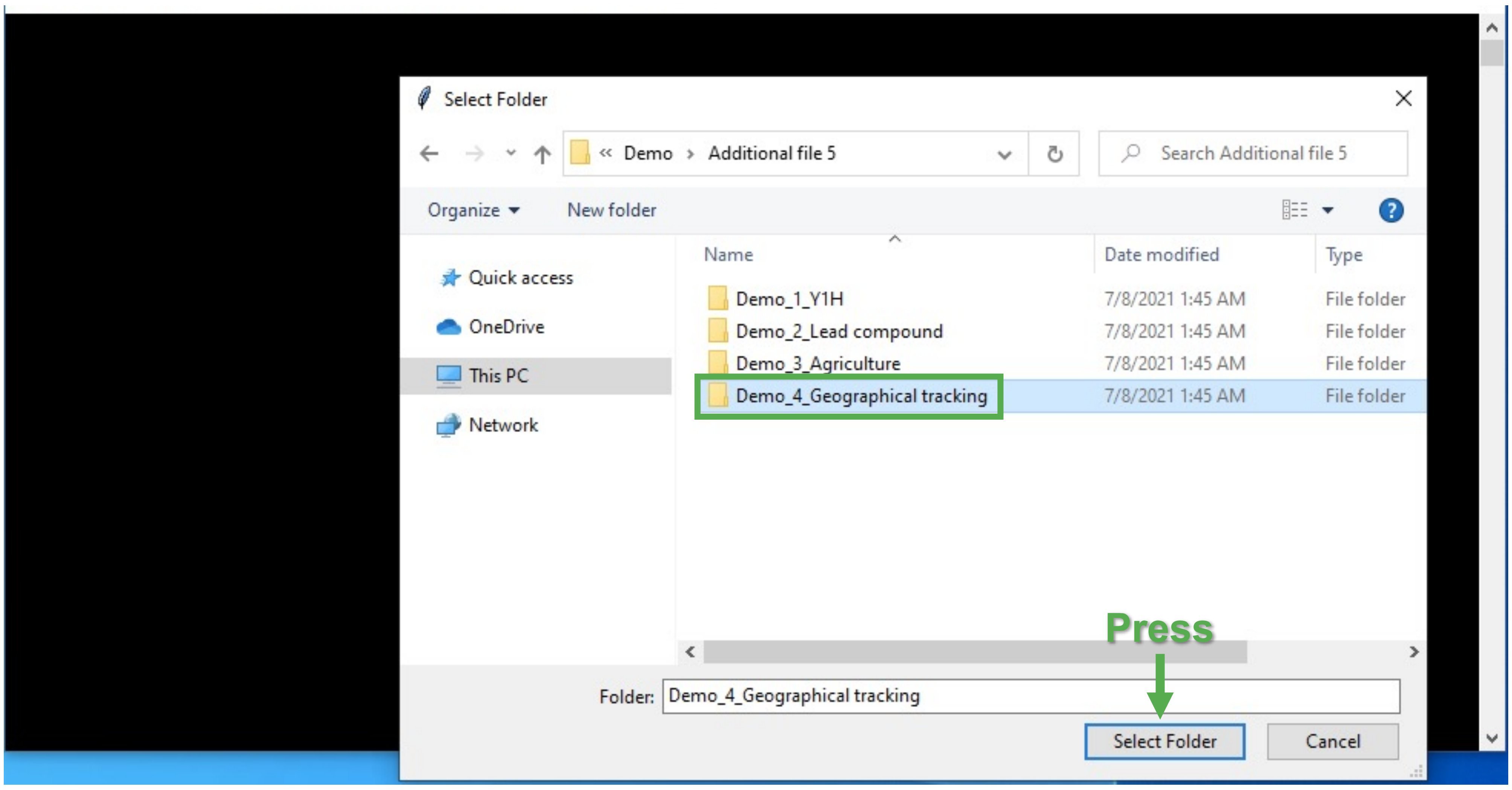

Select the folder "Demo_4_Geographical tracking" (green frame) and press "Select Folder" (indicated by a green arrow)

✱ The GUI of GM_Basic

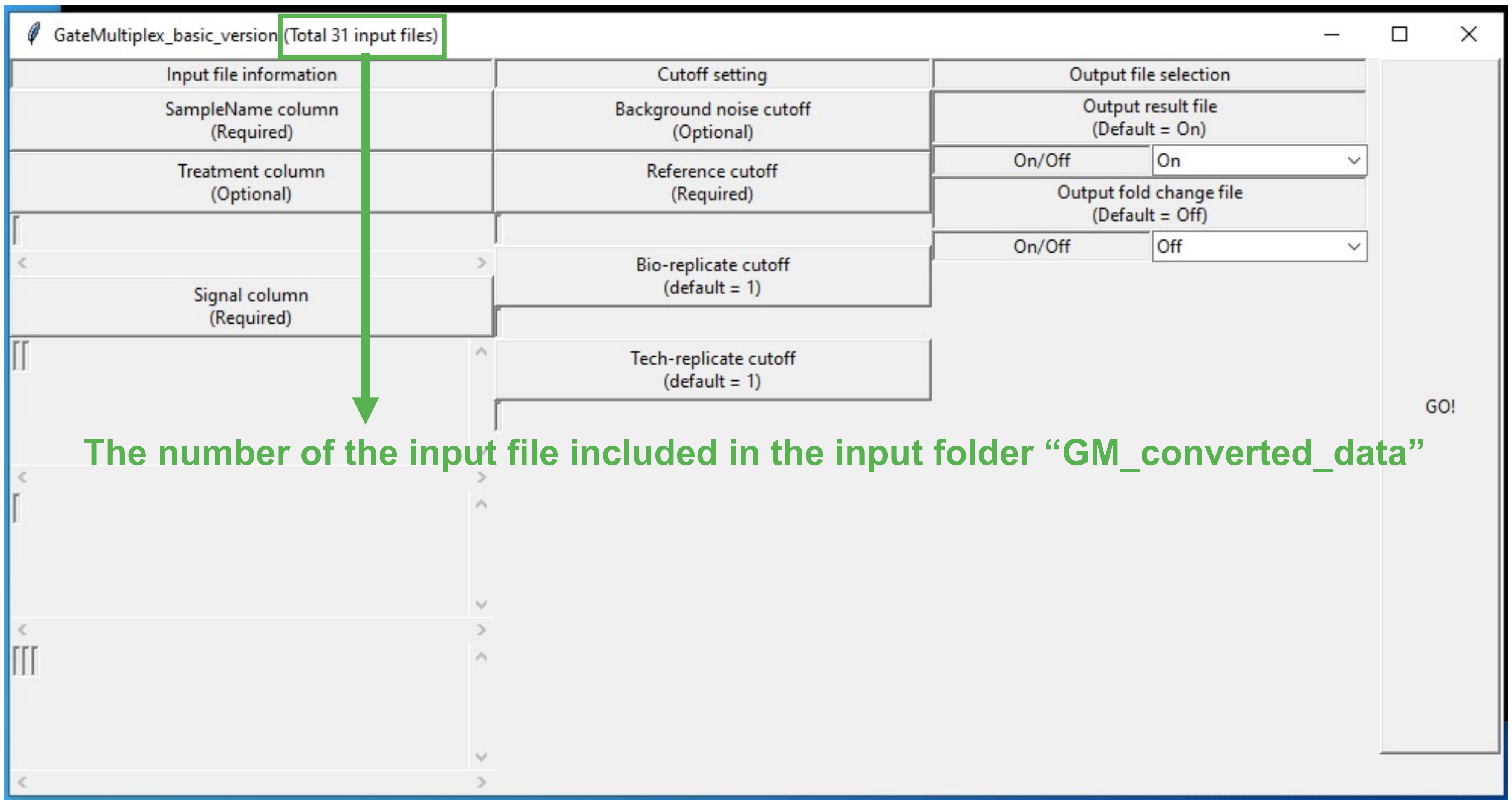

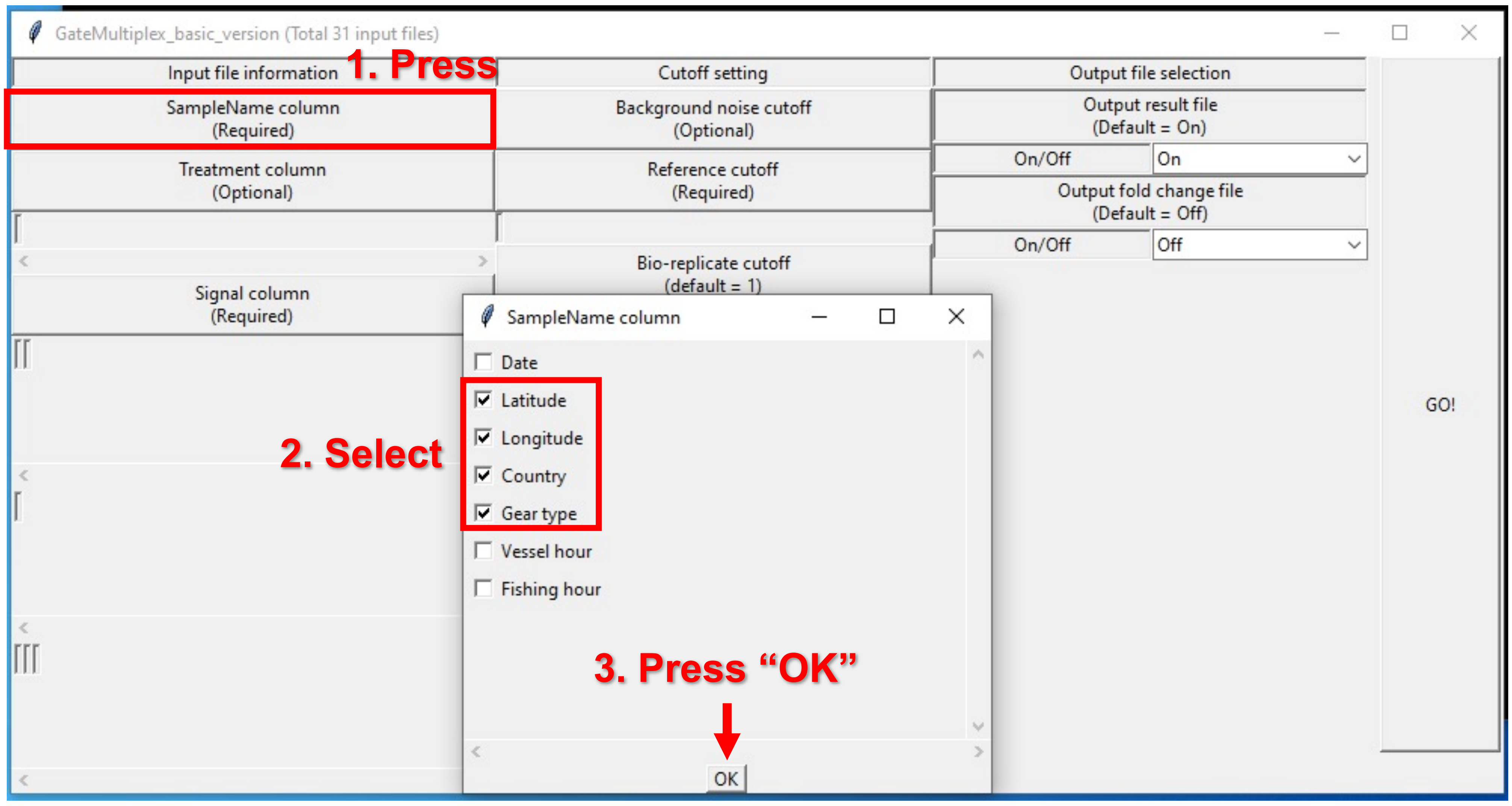

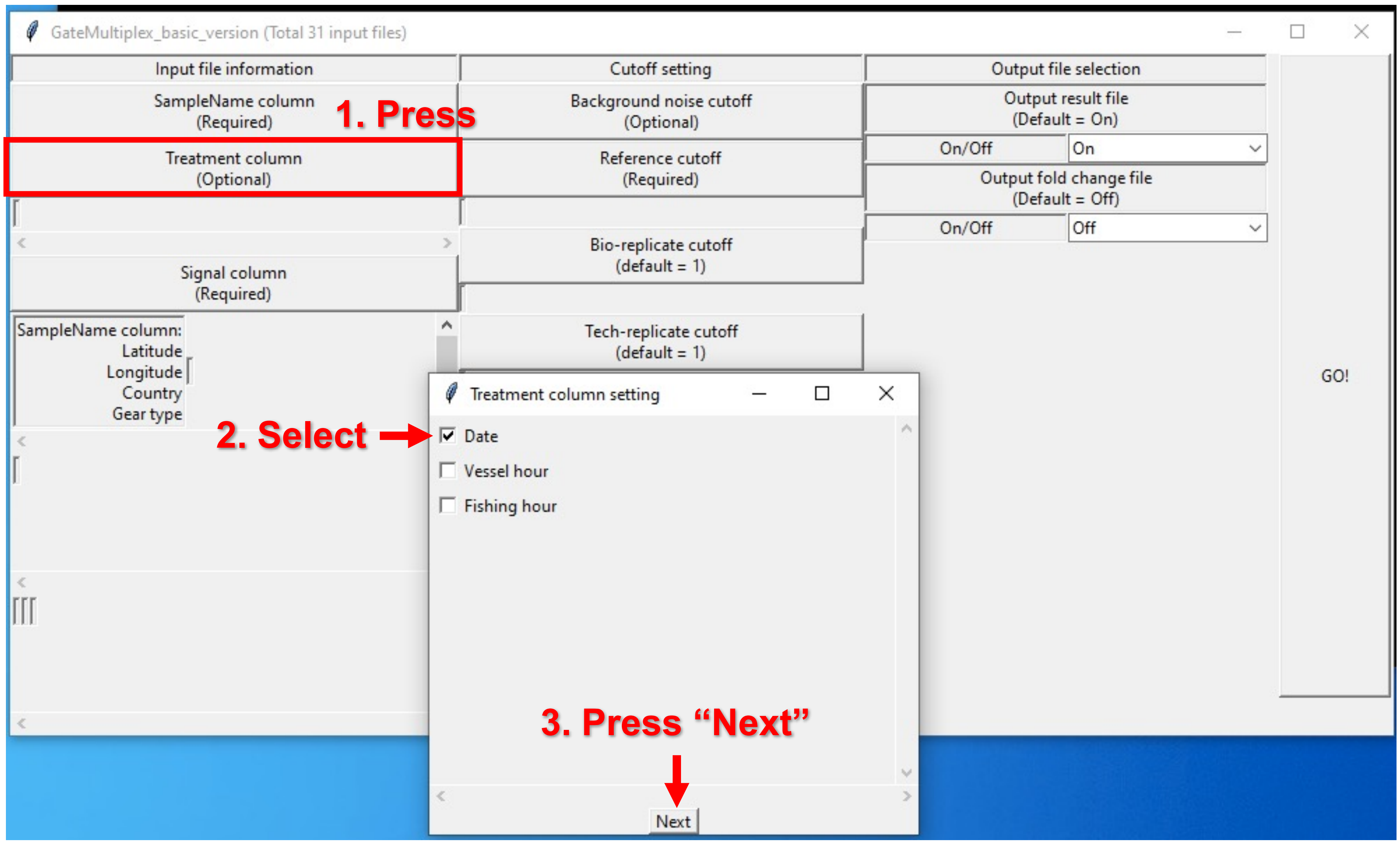
GateMultiplex_basic_version (Total 31 input files)
Input file information
Cutoff setting
Output file selection
SampleName column (Required)
1. Press
Background noise cutoff (Optional)
Output result file (Default = On)
On/Off
On
Treatment column (Optional)
Reference cutoff (Required)
Output fold change file (Default = Off)
On/Off
Off
Bio-replicate cutoff (default = 1)
Signal column (Required)
SampleName column: Latitude Longitude Country Gear type
Tech-replicate cutoff (default = 1)
GO!
Treatment column setting
2. Select
Date
Vessel hour
Fishing hour
3. Press "Next"
Next

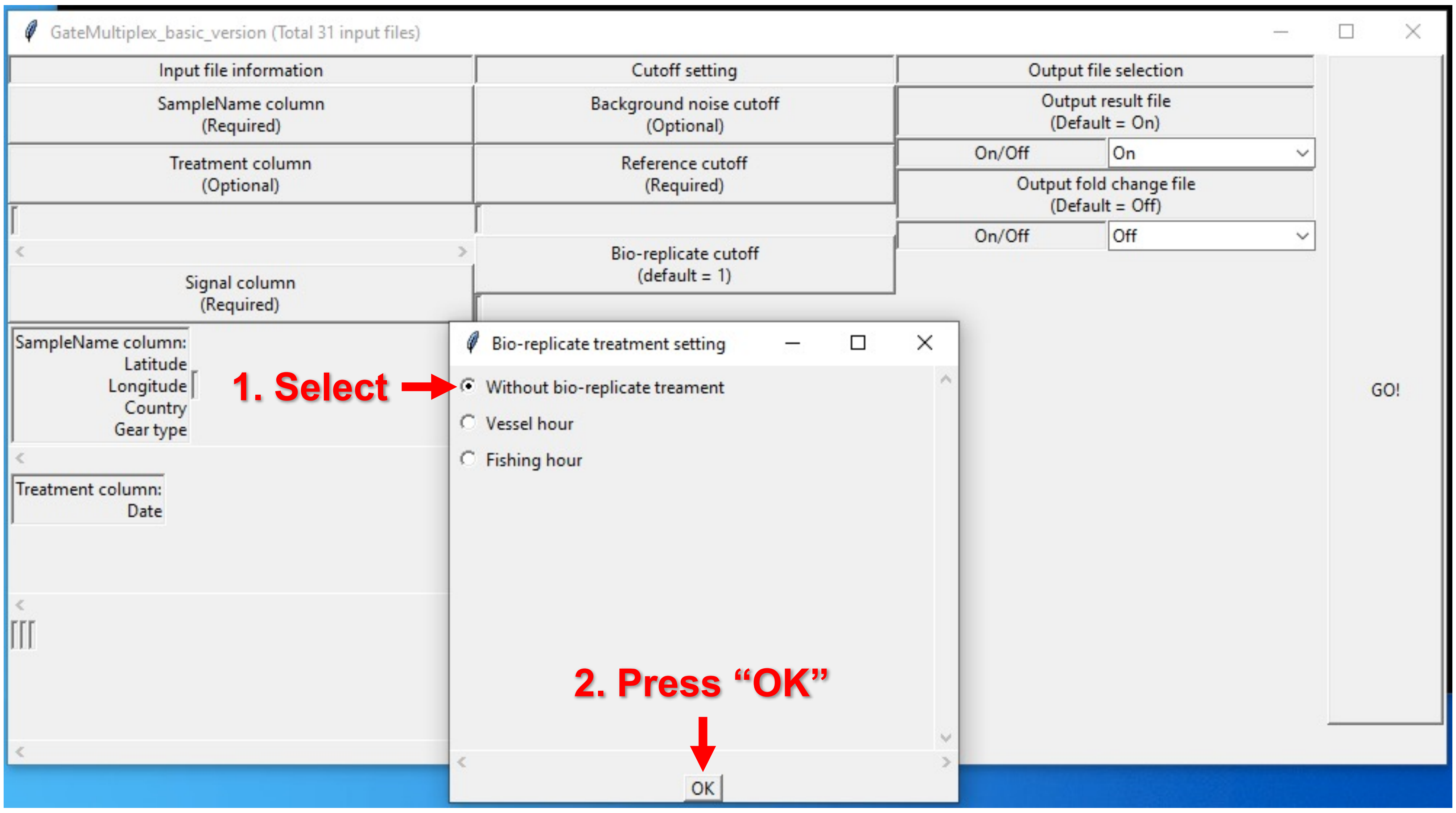
GateMultiplex_basic_version (Total 31 input files)
Input file information
Cutoff setting
Output file selection
SampleName column (Required)
Background noise cutoff (Optional)
Output result file (Default = On)
On/Off
On
Treatment column (Optional)
Reference cutoff (Required)
Output fold change file (Default = Off)
On/Off
Off
Bio-replicate cutoff (default = 1)
Signal column (Required)
SampleName column: Latitude Longitude Country Gear type
Treatment column: Date
GO!
Bio-replicate treatment setting
1. Select
Without bio-replicate treament
Vessel hour
Fishing hour
2. Press "OK"
OK

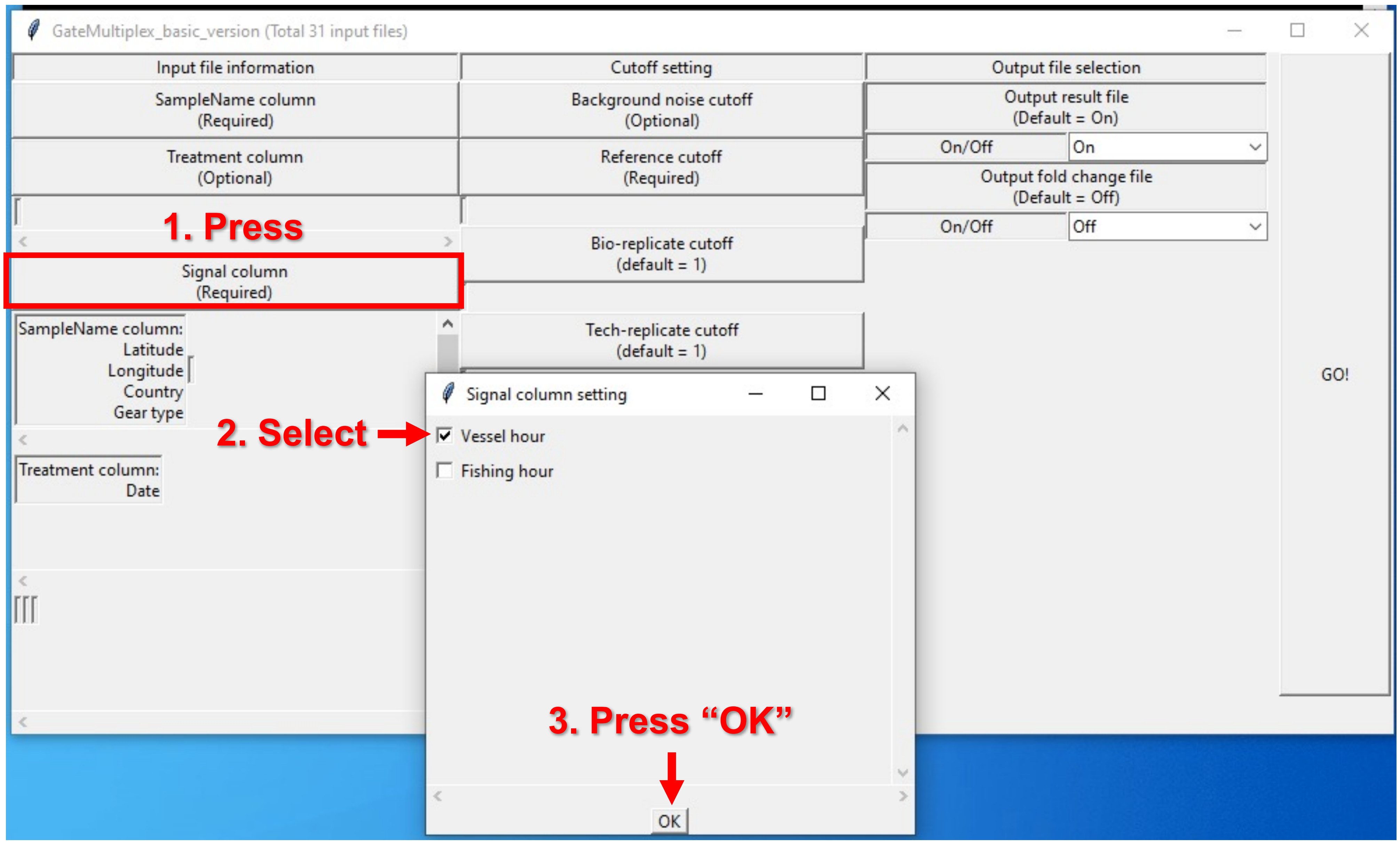

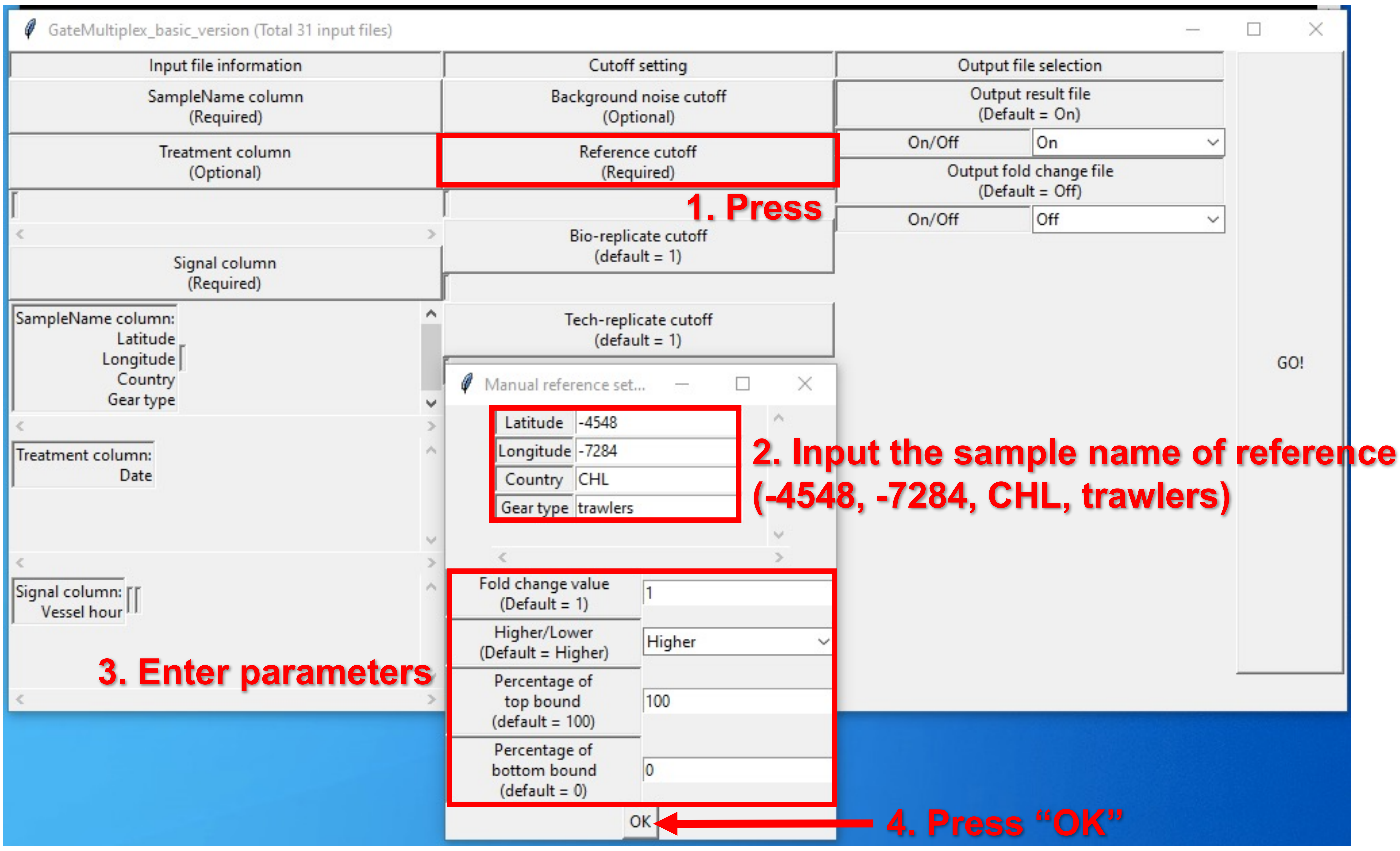

- In the reference cutoff, the sample name combination of -4548 (latitude), -7284 (longitude), CHL (country), trawlers (gear type) is used as the reference. This sample name combination is included in the data of each of the 31 days.

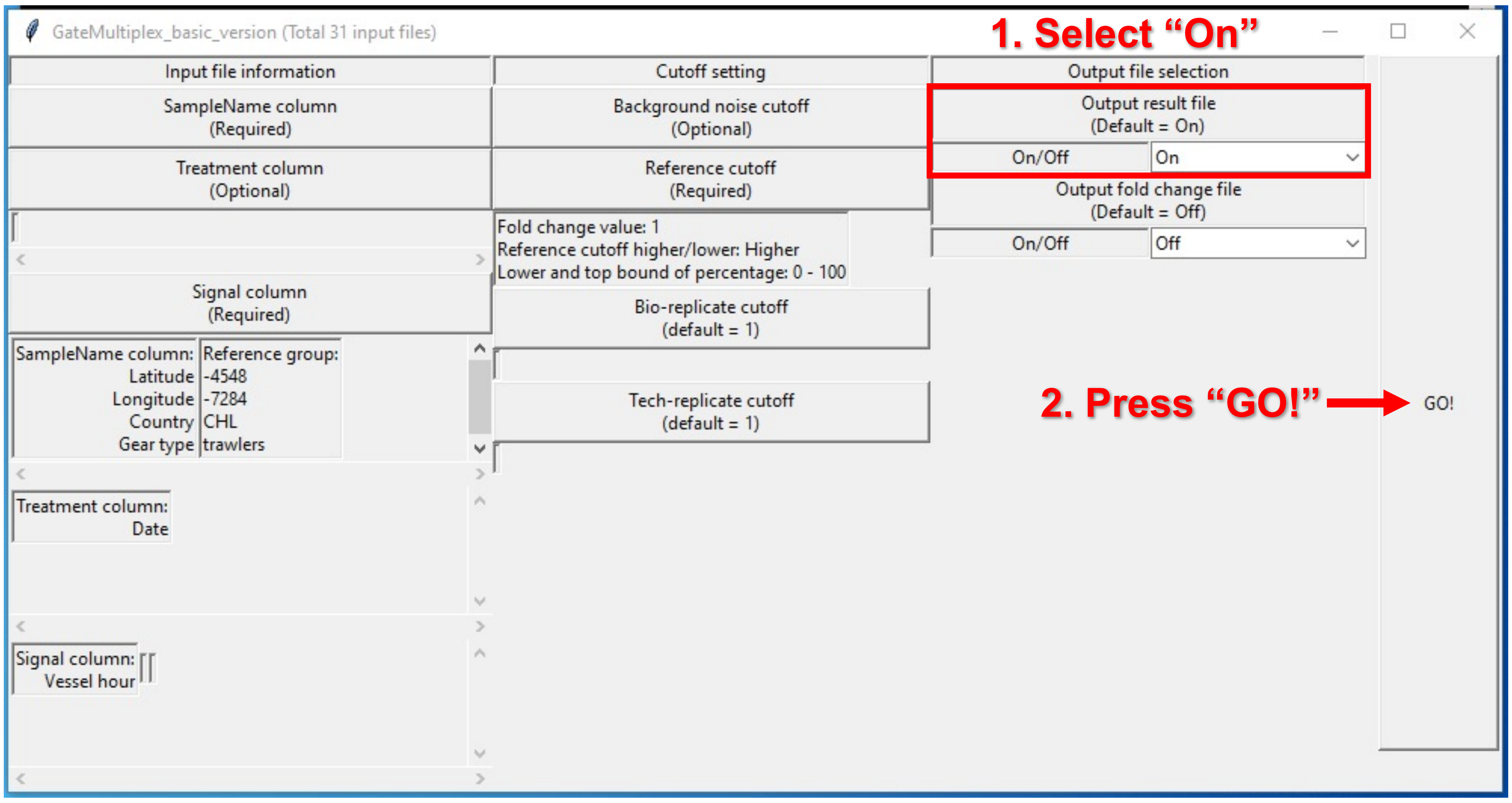

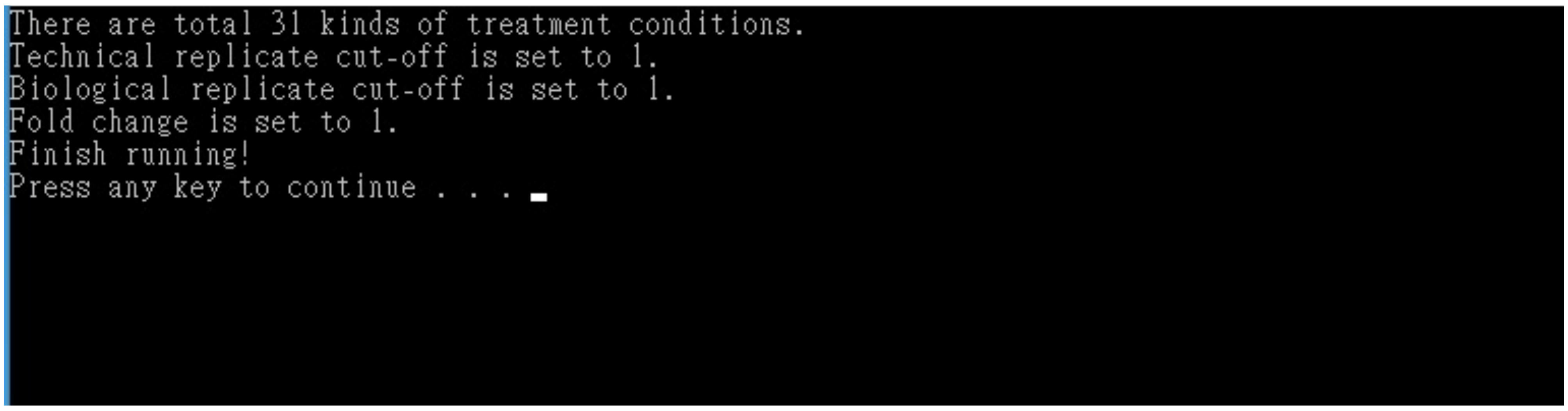

Press any key to close the window

- The result file, named “Results.csv” (red frame), is stored in the same folder with executive files of GM_Basic.

- The symbol “-” means that the data is not exist. The symbol “N” means negative. Please see the “Symbols in output files” section in Additional file 3.

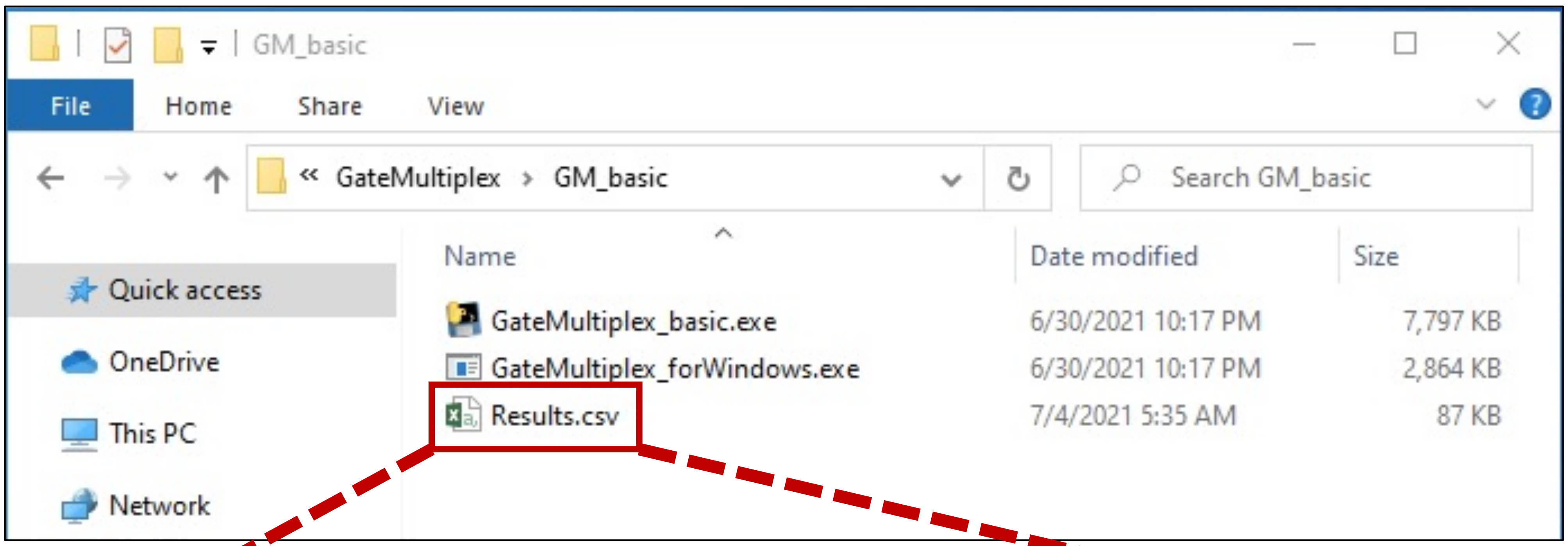

	A	B	C	D	E	F	G
1		1/1/2012;Vessel hour;	1/10/2012;Vessel hour;	1/11/2012;Vessel hour;	1/12/2012;Vessel hour;	1/13/2012;Vessel hour;	1/14/2012;Vessel hour;
2	-1000;-10354;JPN;drifting_longlines	-	-	-	-	-	N
3	-1000;-10;JPN;drifting_longlines	-	-	-	-	-	N
4	-1000;-11065;JPN;drifting_longlines	-	-	-	-	-	-
5	-1000;-11092;JPN;drifting_longlines	-	-	-	-	-	-
6	-1000;-11224;JPN;drifting_longlines	-	-	-	-	-	-
7	-1000;-11225;JPN;drifting_longlines	-	-	-	-	-	-
8	-1000;-11231;JPN;drifting_longlines	-	-	-	-	-	-
9	-1000;-11239;JPN;drifting_longlines	-	-	-	-	-	-
10	-1000;-11244;JPN;drifting_longlines	-	-	-	-	-	-
11	-1000;-11248;JPN;drifting_longlines	-	-	-	-	-	-
12	-1000;-11276;JPN;drifting_longlines	-	-	-	-	-	-
13	-1000;-11277;JPN;drifting_longlines	-	-	-	-	-	-
14	-1000;-11389;JPN;drifting_longlines	-	-	-	-	-	-
15	-1000;-11394;JPN;drifting_longlines	-	-	-	-	-	-
16	-1000;-11572;JPN;drifting_longlines	-	-	-	-	-	-
17	-1000;-11593;JPN;drifting_longlines	-	-	-	-	-	-

Partial view on this file

- In marine ecosystem data, the ships from the same country equip with the same gear type will not appear in the same grid in each day (Please see Fig. S18 and S19 in Additional file 1 for the schematic diagram illustration. Therefore, the combinations of sample names (latitude, longitude, country, and gear type) among 31 days are not the same.
- If the combinations of sample names is not shown on the indicated day, the results will be showed as the symbol “-”.
- If a sample name combination is selected as the reference in reference cutoff, but is not included in some of the 31 days, then data in the indicated day will all be regarded as negative (N).
